# Illuminating *anti*-hydrozirconation: controlled geometric isomerization of an organometallic species†‡

**DOI:** 10.1039/d1sc02454j

**Published:** 2021-07-09

**Authors:** Theresa Hostmann, Tomáš Neveselý, Ryan Gilmour

**Affiliations:** Organisch-Chemisches Institut, Westfälische Wilhelms-Universität Münster Corrensstraße 36 48149 Münster Germany ryan.gilmour@uni-muenster.de

## Abstract

A general strategy to enable the formal *anti*-hydrozirconation of arylacetylenes is reported that merges *cis*-hydrometallation using the Schwartz Reagent (Cp_2_ZrHCl) with a subsequent light-mediated geometric isomerization at *λ* = 400 nm. Mechanistic delineation of the *contra*-thermodynamic isomerization step indicates that a minor reaction product functions as an efficient *in situ* generated photocatalyst. Coupling of the *E*-vinyl zirconium species with an alkyne unit generates a conjugated diene: this has been leveraged as a selective energy transfer catalyst to enable *E* → *Z* isomerization of an organometallic species. Through an *Umpolung* metal–halogen exchange process (Cl, Br, I), synthetically useful vinyl halides can be generated (up to *Z* : *E* = 90 : 10). This enabling platform provides a strategy to access nucleophilic and electrophilic alkene fragments in both geometric forms from simple arylacetylenes.

## Abstract 2

A general strategy to enable the formal *anti*-hydrozirconation of arylacetylenes is reported that merges *cis*-hydrometallation using the Schwartz Reagent (Cp_2_ZrHCl) with a subsequent light-mediated geometric isomerization at *λ* = 400 nm.

## Graphical abstract

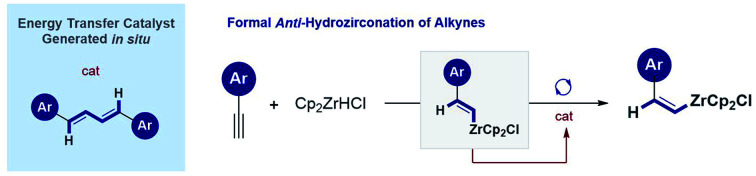

The venerable Schwartz reagent (Cp_2_ZrHCl) is totemic in the field of hydrometallation,^1^ where reactivity is dominated by *syn*-selective M–H addition across the π-bond.^2,3^ This mechanistic foundation can be leveraged to generate well-defined organometallic coupling partners that are amenable to stereospecific functionalization. Utilizing terminal alkynes as readily available precursors,^4^ hydrozirconation constitutes a powerful strategy to generate *E*-configured vinyl nucleophiles that, through metal–halogen exchange, can be converted to vinyl electrophiles in a formal *Umpolung* process.^5^ Whilst this provides a versatile platform to access the electronic antipodes of the *E*-isomer, the mechanistic course of addition renders access to the corresponding *Z*-isomer conspicuously challenging. To reconcile the synthetic importance of this transformation with the intrinsic challenges associated with *anti*-hydrometallation and metallometallation,^6^ it was envisaged that a platform to facilitate geometric isomerization^7^ would be of value. Moreover, coupling this to a metal–halogen exchange would provide a simple *Umpolung* matrix to access both stereo-isomers from a common alkyne precursor (Fig. 1).

**Fig. 1:**
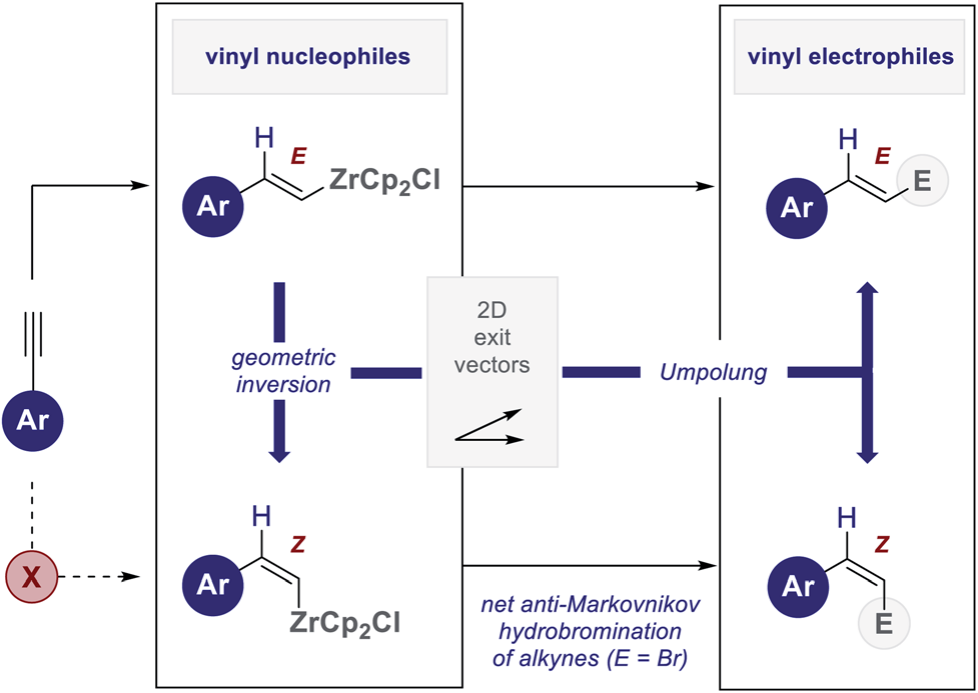
The stereochemical course of alkyne hydrometallation using the Schwartz reagent and an *Umpolung* platform to generate both stereo-isomers from a common alkyne precursor.

Confidence in this conceptual blueprint stemmed from a report by Erker and co-workers, in which irradiating the vinyl zirconium species derived from phenyl acetylene (0.5 M in benzene) with a mercury lamp (Philips HPK 125 and Pyrex filter) induced geometric isomerization.^8^ Whilst Hg lamps present challenges in terms of safety, temperature regulation, cost and wavelength specificity, advances in LED technology mitigate all of these points. Therefore, a process of reaction development was initiated to generalize the *anti*-hydrozirconation of arylacetylenes. Crucial to the success of this venture was identifying the light-based activation mode that facilitates alkene isomerization. Specifically, it was necessary to determine whether this process was enabled by direct irradiation of the vinyl zirconium species, or if the *E* → *Z* directionality results from a subsequent selective energy transfer process involving a facilitator. Several accounts of the incipient vinyl zirconium species reacting with a second alkyne unit to generate a conjugated diene have been disclosed.^9,10^ It was therefore posited that the minor by-product diene may be a crucial determinant in driving this isomerization (Fig. 2).

**Fig. 2:**
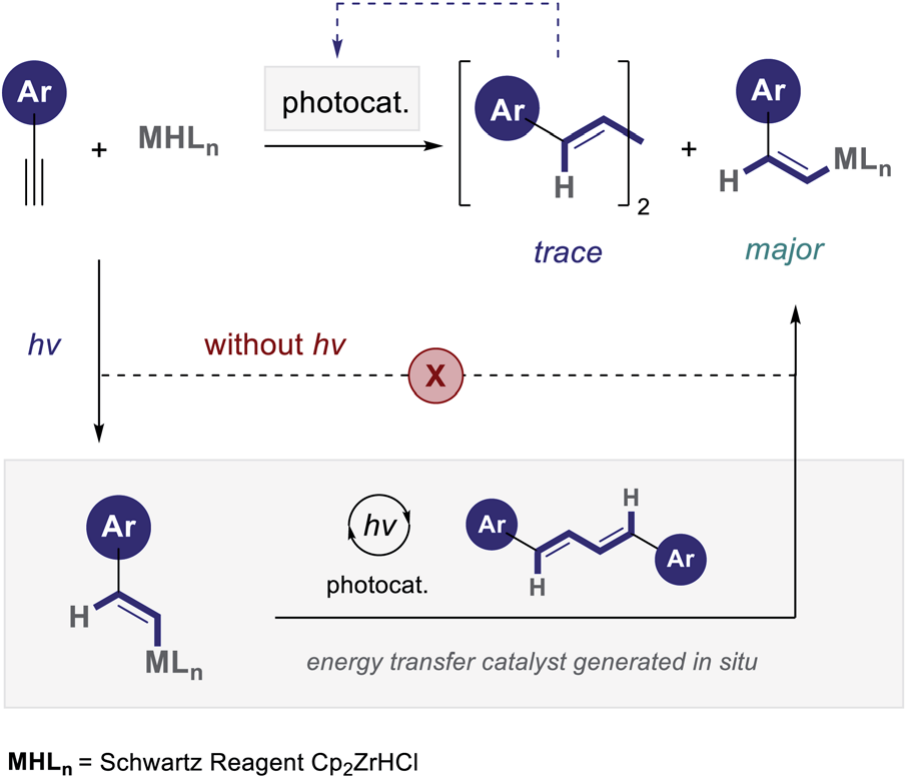
A working hypothesis for the light-mediated *anti*-hydrozirconation *via* selective energy transfer catalysis.

To advance this working hypothesis and generalize the formal *anti*-hydrozirconation process, the reaction of Cp_2_ZrHCl with 1-bromo-4-ethynylbenzene (**A-1**) in CH_2_Cl_2_ was investigated (Table 1). Following hydrozirconation, the vinyl zirconium species was irradiated at *λ* = 400 nm (Avonec, 3 W High Power LED) for the time indicated. To facilitate analysis, the post-isomerization reaction mixture was treated with NBS to generate the vinyl bromide following stereospecific metal–halogen exchange (see the ESI‡ for full details). This generates a versatile electrophile for downstream synthetic applications. Gratifyingly, after only 15 minutes, a *Z* : *E*-composition of 50 : 50 was reached (entry 1) and, following treatment with NBS, the desired vinyl bromide **(Z)-1** was obtained in 76% yield (isomeric mixture) over the two steps. Further increasing the irradiation by 15 minute increments (entries 2–4) revealed that the optimum reaction time for the isomerization is 45 minutes (74%, *Z* : *E* = 73 : 27, entry 3). Extending the reaction time to 60 minutes (entry 4, 54%) did not lead to an improvement in selectivity and this was further confirmed by irradiating the reaction mixture for 90 minutes (entry 5). In both cases, a notable drop in yield was observed and therefore the remainder of the study was performed using the conditions described in entry 3. Next, the influence of the irradiation wavelength on the isomerization process was examined (entries 6–11). From a starting wavelength of *λ* = 369 nm, which gave a *Z* : *E*-ratio of 27 : 73 (entry 6), a steady improvement was observed by increasing the wavelength to *λ* = 374 nm (*Z* : *E* = 44 : 56, entry 7) and *λ* = 383 nm (*Z* : *E* = 53 : 47, entry 8). The selectivity reached a plateau at *λ* = 400 nm, with higher wavelengths proving to be detrimental (*Z* : *E* = 60 : 40 at *λ* = 414 nm, entry 9; *Z* : *E* = 26 : 74 at *λ* = 435 nm, entry 10). It is interesting to note that at *λ* = 520 nm, **Z-1** was not detected by ^1^H NMR (entry 11).

**Table 1:** Reaction optimization^a^

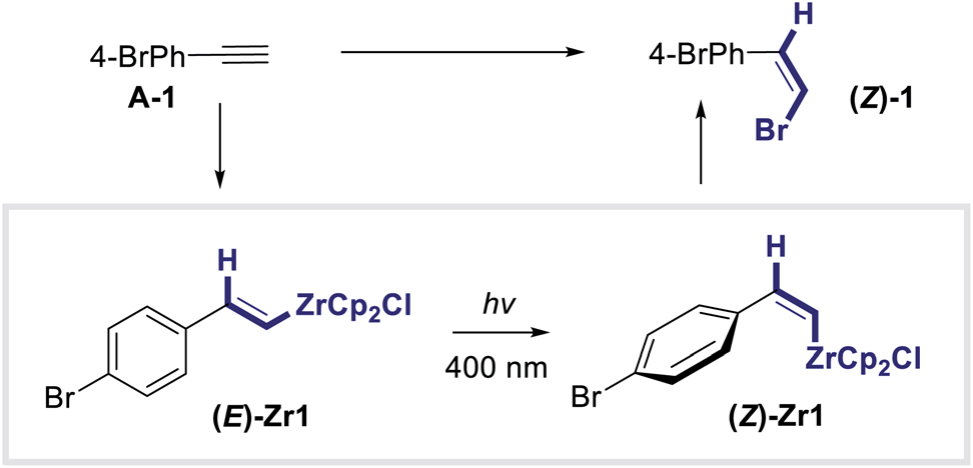				
Entry	*λ* [nm]	Time [min]	Yield^b^	*Z* : *E* ratio^b^
1	400	15	76%	50 : 50
2	400	30	72%	68 : 32
3	400	45	74% (74%)	74 : 26 (73 : 27)
4	400	60	54%	73 : 27
5	400	90	49%	73 : 27
6	369	45	66%	27 : 73
7	374	45	61%	44 : 56
8	383	45	64%	53 : 47
9	414	45	67%	60 : 40
10	435	45	72%	26 : 74
11	520	45	67%	<5 : 95

a (i) Cp_2_ZrHCl (62 mg, 0.24 mmol, 1.2 eq.), CH_2_Cl_2_ (1.5 mL), alkyne **A-1** (36 mg, 0.2 mmol, 1.0 eq.) in CH_2_Cl_2_ (0.5 mL); (ii) irradiation; (iii) NBS (39 mg, 0.22 mmol, 1.1 eq.).

b average yield and *Z* : *E* ratio of two reactions determined by ^1^H-NMR with DMF as internal standard; isolated yield of the *Z* : *E*-mixture and *Z* : *E*-ratio in parentheses.

Having identified standard conditions to enable a hydrozircononation/isomerization/bromination sequence, the scope and limitations of the method was explored using a range of electronically and structurally diverse phenylacetylenes (Fig. 3). This constitutes a net *anti*-Markovnikov hydrobromination of alkynes.^11^

**Fig. 3:**
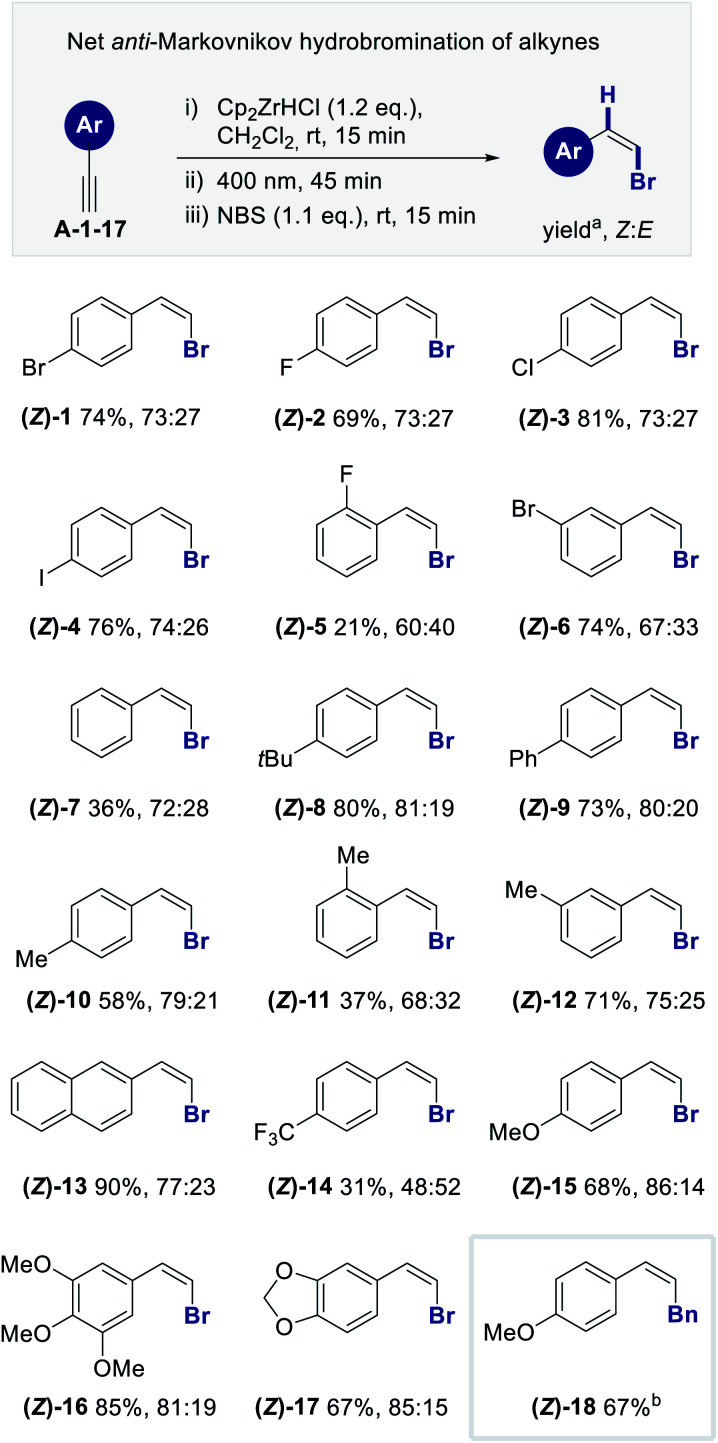
Aromatic scope for the formal *anti*-hydrozirconation of terminal alkynes; reaction conditions: (i) Cp_2_ZrHCl (62 mg, 0.24 mmol, 1.2 eq.), CH_2_Cl_2_ (1.5 mL), alkyne **A-1-17** (0.2 mmol, 1.0 eq.) in CH_2_Cl_2_ (0.5 mL), 15 min; (ii) irradiation (*λ* = 400 nm), 45 min; (iii) NBS (39 mg, 0.22 mmol, 1.1 eq.), 15 min; ^a^isolated yield of *Z* : *E*-mixture as average of two reactions; ^b^(i) Cp_2_ZrHCl (62 mg, 0.24 mmol, 1.2 eq.), CH_2_Cl_2_ (1.5 mL), alkyne **A-15** (26 mg, 0.2 mmol, 1.0 eq.) in CH_2_Cl_2_ (0.5 mL); (ii) irradiation (*λ* = 400 nm), 45 min; (iii) PdPPh_3_ (7 mg, 0.006 mmol, 0.03 eq.) in THF (0.4 mL), BnBr (24 μL, 0.2 mmol, 1.0 eq.), rt, 18 h.^12^

The introduction of halogen substituents in the 4-position proved to be compatible with the reaction conditions, enabling the formation of **(Z)-1-4** in up to 81% yield (up to *Z* : *E* = 74 : 26). Interestingly, the introduction of the *o*-F **(Z)-5** substituent led to a drop in the yield and selectivity: this is in stark contrast to cinnamoyl derivatives that have previously been examined in this laboratory.^12^ The *m*-Br proved to be less challenging enabling **(Z)-6** to be generated smoothly (74%, *Z* : *E* = 67 : 33). The parent phenylacetylene (**A-7**) could be converted with a similar *Z* : *E*-ratio to **(Z)-7** albeit less efficiently (36%, *Z* : *E* = 72 : 28). Electron donating groups in the *para* position such as **(Z)-8-10** led to a general improvement in selectivity (up to 80%, *Z* : *E* = 81 : 19). Whereas methylation at the *ortho*-position compromised efficiency [**(Z)-11**, 37%, *Z* : *E* = 68 : 32], translocation to the meta-position led to a recovery in terms of yield and *Z* : *E*-ratio [**(Z)-12**, 71%, *Z* : *E* = 75 : 25]. Extending the π-system from phenyl to naphthyl enabled the generation of **(Z)-13** 90% and with a *Z* : *E*-ratio of 77 : 23. To enable a direct comparison of strongly and weakly donating groups on the reaction outcome the *p*-CF_3_ and *p*-OMe derivatives were examined. In the trifluoromethyl derivative **(Z)-14** a decrease in yield (31%) and selectivity (*Z* : *E* = 48 : 52) was noted. In contrast, the *para* methoxy group in **(Z)-15** led to an enhanced *Z* : *E* ratio of 86 : 14 (68% yield). This behavior was also observed with the trimethoxy derivative **(Z)-16** (*Z* : *E*-ratio of 81 : 19). The piperonyl derivative performing similarly to the *para* methoxy derivative thereby enabling the formation of **(Z)-17** with a *Z* : *E*-ratio of 85 : 15 (67% yield). Finally, to demonstrate the utility of the method, a direct transmetallation protocol was performed to intercept the *Z*-vinyl zirconium species with benzyl bromide.^13^ This enabled the synthesis of **(Z)-18** in 67% yield.

To demonstrate the compatibility of this platform with other common electrophiles, the deuterated, chlorinated and iodinated systems **(Z)-19**, -**20** and -**21** were prepared (Fig. 4). Yields and selectivities that are fully comparable with Fig. 3 were observed (up to 80% yield and *Z* : *E* = 80 : 20). Finally, to augment the photostationary composition further, a process of structural editing was conducted. It was envisaged that integrating a stabilizing non-covalent interaction in the *Z*-vinyl zirconium species may bias isomerization selectivity. Recent studies from this laboratory have established that a stabilizing interaction between the boron p-orbital and an adjacent non-bonding electron pair can be leveraged to induce a highly selective geometric isomerization of *β*-borylacrylates (Fig. 5, top).^14^

**Fig. 4:**
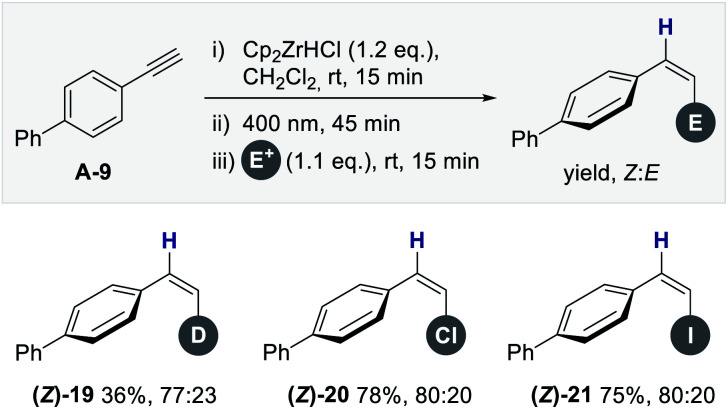
Scope of electrophiles for the formal *anti*-hydrozirconation; reaction conditions: (i) Cp_2_ZrHCl (62 mg, 0.24 mmol, 1.2 eq.), CH_2_Cl_2_ (1.5 mL), **A-9** (36 mg, 0.2 mmol, 1.0 eq.) in CH_2_Cl_2_ (0.5 mL); (ii) irradiation (*λ* = 400 nm), 45 min; (iii) *E*^+^ (DCl, NCS or NIS) (0.22 mmol, 1.1 eq.), 15 min; isolated yields of the *Z* : *E*-mixture are reported.

**Fig. 5:**
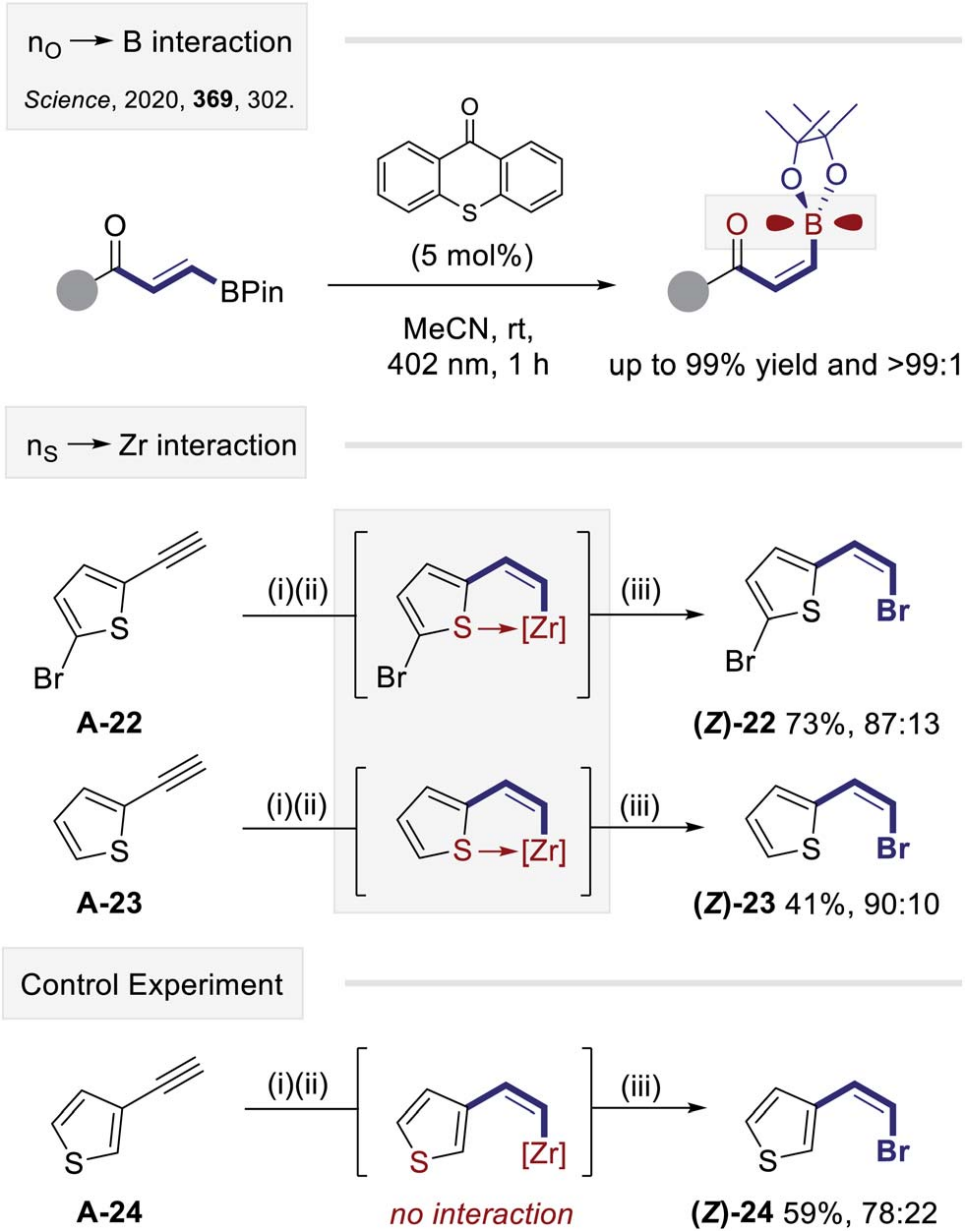
Enhancing the selectivity of *anti*-hydrozirconation by leveraging a postulated *n*_S_ → Zr interaction. Reaction conditions: (i) Cp_2_ZrHCl (62 mg, 0.24 mmol, 1.2 eq.), CH_2_Cl_2_ (1.5 mL), alkyne **A-22-24** (0.2 mmol, 1.0 eq.) in CH_2_Cl_2_ (0.5 mL), rt, 15 min; (ii) irradiation (*λ* = 400 nm), 45 min; (iii) NBS (39 mg, 0.22 mmol, 1.1 eq.), rt, 15 min.

Gratifyingly, the 5-bromo thiophenyl derivative **(Z)-22** was generated with a *Z* : *E* ratio of 87 : 13 in 73% yield, and the unsubstituted derivative **(Z)-23** was obtained in 41% yield higher selectivity (*Z* : *E* = 90 : 10). As a control experiment, the regioisomeric product **(Z)-24** was prepared in which the sulfur atom is distal from the zirconium center. This minor alteration resulted in a conspicuous drop of selectivity (*Z* : *E* = 78 : 22), which is in line with the phenyl derivatives. Given the prominence of Frustrated-Lewis-Pairs (FLPs) in small molecule activation,^15^ materials such as **(Z)-22** and **(Z)-23** may provide a convenient starting point for the development of future candidates.

To provide structural support for the formation of a *Z*-vinyl zirconium species upon irradiation at *λ* = 400 nm, the standard experiment was repeated in deuterated dichloromethane and investigated by ^1^H NMR spectroscopy. The spectra shown in Fig. 6 confirm the formation of transient *E*- and *Z*-vinyl zirconium species **(E)-Zr1** and **(Z)-Zr1** and are in good agreement with literature values.^8^ Diagnostic resonances of **(E)-Zr1** include H^1^ at 7.76 ppm, whereas the analogous signal in **(Z)-Zr1** is high field shifted to 6.33 ppm (Δ*δ*(H^1^_Z−E_) = −1.43 ppm). In contrast, the H^2^ signal for **(Z)-Zr1** appears at 7.56 ppm, which is at lower field compared to the H^2^ signal for **(E)-Zr1** at 6.64 ppm (Δ*δ*(H^2^_Z−E_) = 0.92 ppm). In the ^13^C-NMR spectra (see the ESI‡) the carbon signal of C1 and C2 are both low field shifted for **(Z)-Zr1** compared to **(E)-Zr1** (Δ*δ*(C1_Z−E_) = 10.5 ppm and Δ*δ*(C1_Z-E_) = 5.6 ppm).

**Fig. 6:**
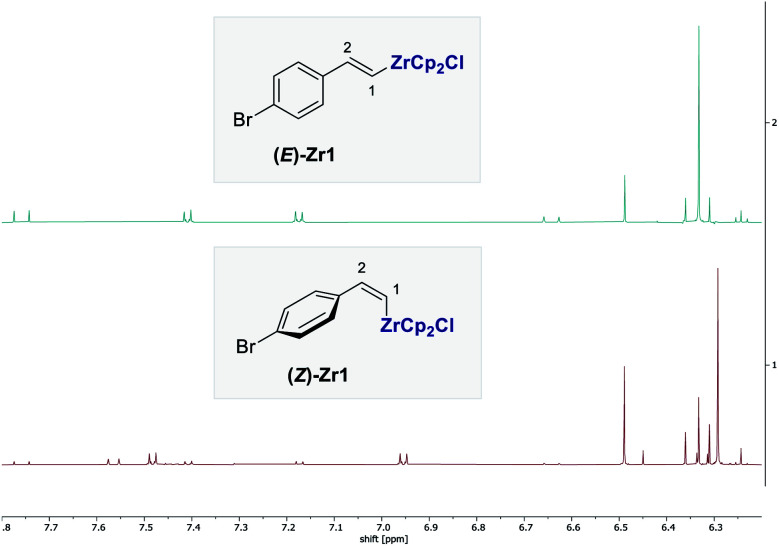
^1^H-NMR of the transient vinylzirconium species **(E)-Zr1** (top) and **(Z)-Zr1** (bottom).

A computational analysis of the vinyl zirconium isomers **(E)-Zr1** and **(Z)-Zr1** revealed two low energy conformers for each geometry (Fig. 7. For full details see the ESI‡). These optimized structures served as a basis for more detailed excited state calculations using a time-dependent density functional theory (TDDFT) approach. These data indicate that isomerization of the styrenyl zirconium species by direct irradiation is highly improbable using *λ* = 400 nm LEDs. However, upon measuring the absorption spectrum of the reaction mixture (Fig. 8, bottom), the shoulder of a band reaching to the visible part of the spectrum is evident (for more details see the ESI‡). Furthermore, the fluorescence spectrum (Fig. 8, top) clearly shows light emission from the reaction mixture. Collectively, these data reinforce the working hypothesis that a minor reaction product functions as a productive sensitizer, thereby enabling the isomerization to occur *via* selective energy transfer.

**Fig. 7:**
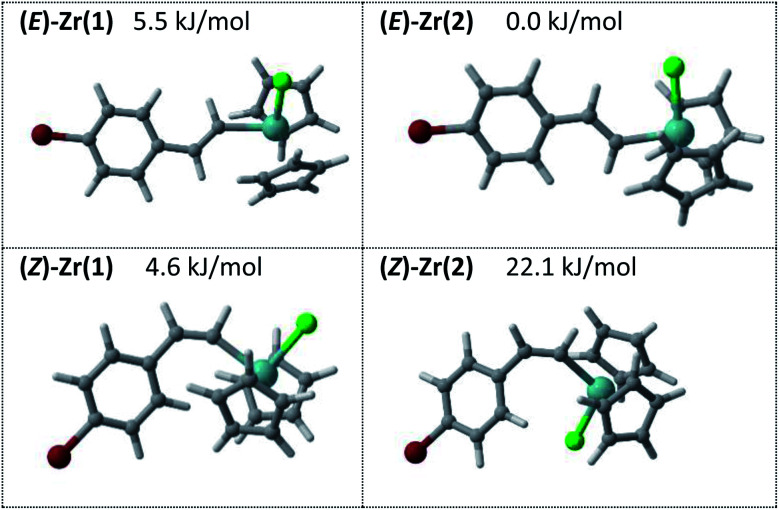
A comparative analysis of **(E)-Zr1** and **(Z)-Zr1**.

**Fig. 8:**
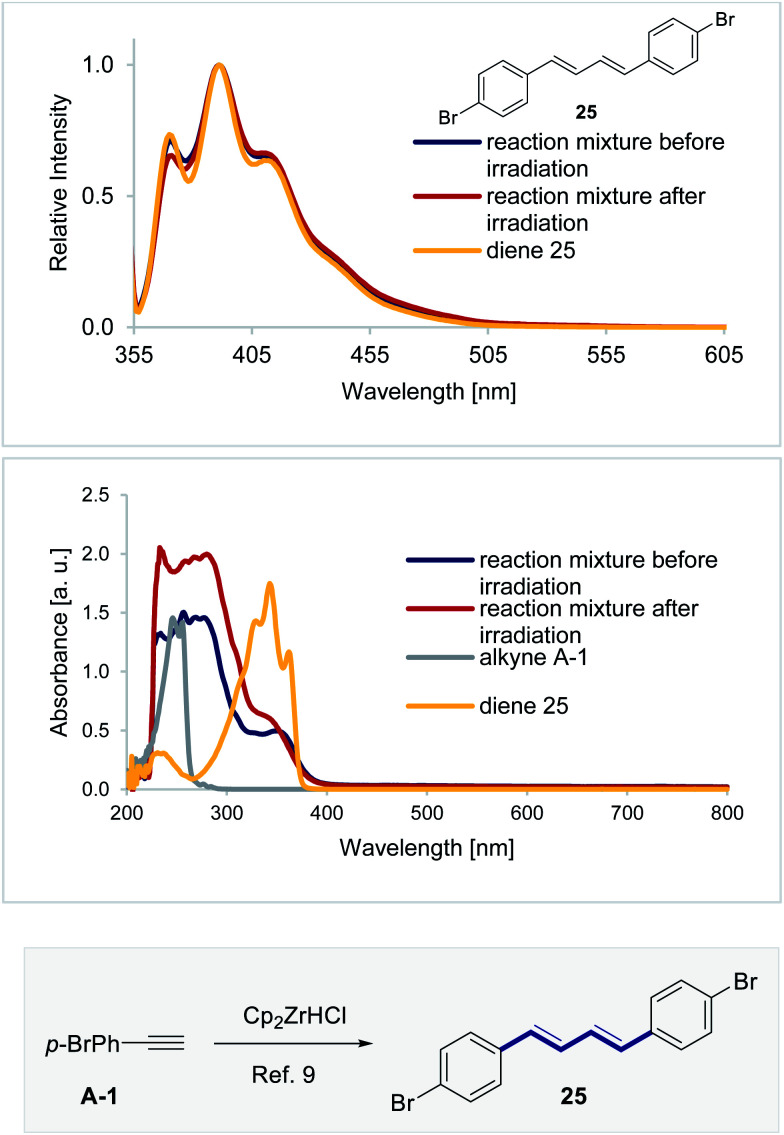
(Top) Fluorescence spectra of the reaction mixture before and after irradiation, and the diene **25** (*c* = 0.1 mm, irradiation at *λ* = 350 nm). (Bottom) Absorption spectra of the reaction mixture before and after irradiation (*c* = 0.1 mm), the alkyne **A-1** and the diene **25** (*c* = 0.05 mm).

As previously highlighted, phenylacetylenes are known to dimerize in the presence of Cp_2_Zr* based complexes.^9,16^ Therefore, to provide support for the involvement of such species, diene **25** was independently prepared and its absorption and emission spectra were compared with those of the reaction mixture (Fig. 8). The emission spectra of the reaction mixture and of diene **25** are closely similar. It is also pertinent to note that diene **25** was also detected in the crude reaction mixture by HRMS (see the ESI‡).

Whilst the spectral measurements in Fig. 8 are in line with diene **25** functioning as an *in situ* photocatalyst, more direct support was desirable. Frustratingly, efforts to subject **(E)-Zr-1** and **(Z)-Zr-1** to standard Stern–Volmer quenching studies were complicated by difficulties in removing diene **25** from the samples. It was therefore envisaged that doping reactions with increasing quantities of diene **25** might be insightful. To that end, the hydrozirconation/isomerization sequence was performed with 0.5, 1.0 and 2.5 mol% of diene **25** and the reactions were shielded from light after 5 minutes. Analysis of the mixture by ^1^H NMR spectroscopy revealed a positive impact of **25** on the *Z* : *E* selectivity, (*Z* : *E* = 23 : 77, 24 : 76 and 30 : 70, respectively. Fig. 9, top). To further demonstrate the ability of diene **25** to act as an energy transfer catalyst for geometric isomerization, two model alkenes containing the styrenyl chromophore were exposed to the standard reaction conditions and the photostationary composition was measured after 45 min. Exposing *trans*-stilbene **(E)-26** to the isomerization conditions furnished a *Z* : *E* photostationary composition of 44 : 56. Similarly, *trans*-β-methyl styrene **(E)-27** could be isomerized to the *cis*-β-methyl styrene **(Z)-27** with a *Z* : *E* ratio of 47 : 53. No isomerization was observed at *λ* = 400 nm in the absence of the catalyst. Whilst direct comparison with the isomerization of vinyl zirconium species must be made with caution, these experiments demonstrate that dienes such as **25** have the capacity to act as photosensitizers with styrenyl chromophores.

**Fig. 9:**
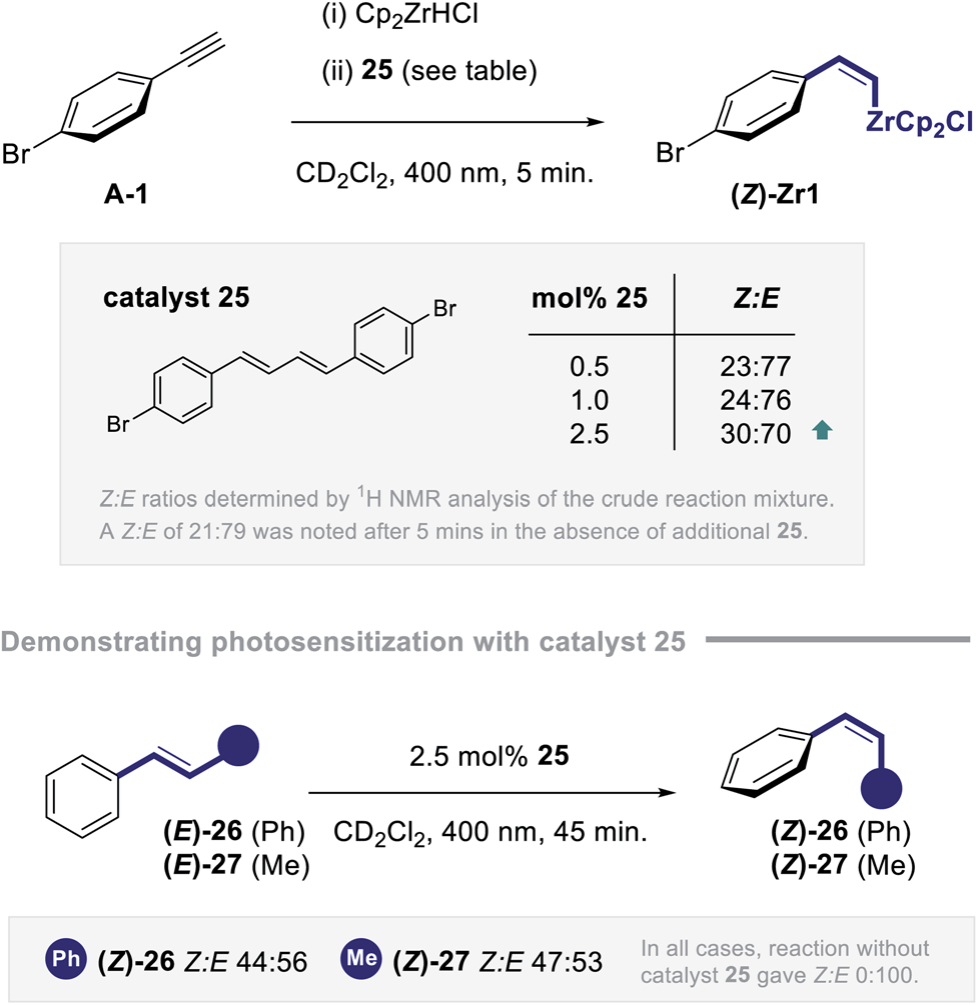
(Top) Exploring the impact of adding diene **25** as an external photocatalyst. (Bottom) Validating photosensitization of the styrenyl chromophore using diene **25**.

Collectively, these data support the hypothesis that isomerization does not result from direct irradiation alone,^17^ but that conjugated dienes, which are produced in small amounts, function as *in situ* energy transfer catalysts (Fig. 10). This antenna undergoes rapid *inter-system crossing* (ISC)^18^ to generate the triplet state and, upon energy transfer to the alkene fragment, returns to the ground state.^19^ This mechanistic study has guided the development of an operationally simple *anti*-hydrozirconation of alkynes that relies on inexpensive LED irradiation. Merging this protocol with a sequential metal–halogen exchange enables the formal *anti*-Markovnikov hydrobromination of alkynes^11^ and provides a sterodivergent platform to access defined alkene vectors from simple alkynes. This complements existing strategies to isomerize vinyl bromides,^20^ and circumvents the risks of vinyl cation formation and subsequent degradation.^21^ Finally, the selectivity of this geometric isomerization can be further augmented through the judicious introduction of stabilizing non-covalent interactions (up to *Z* : *E* = 90 : 10). It is envisaged that this selective, controlled geometric isomerization of an organometallic species will find application in contemporary synthesis. Furthermore, it contributes to a growing body of literature that describes the *in situ* formation of photoactive species upon irradiation.^22^

**Fig. 10:**
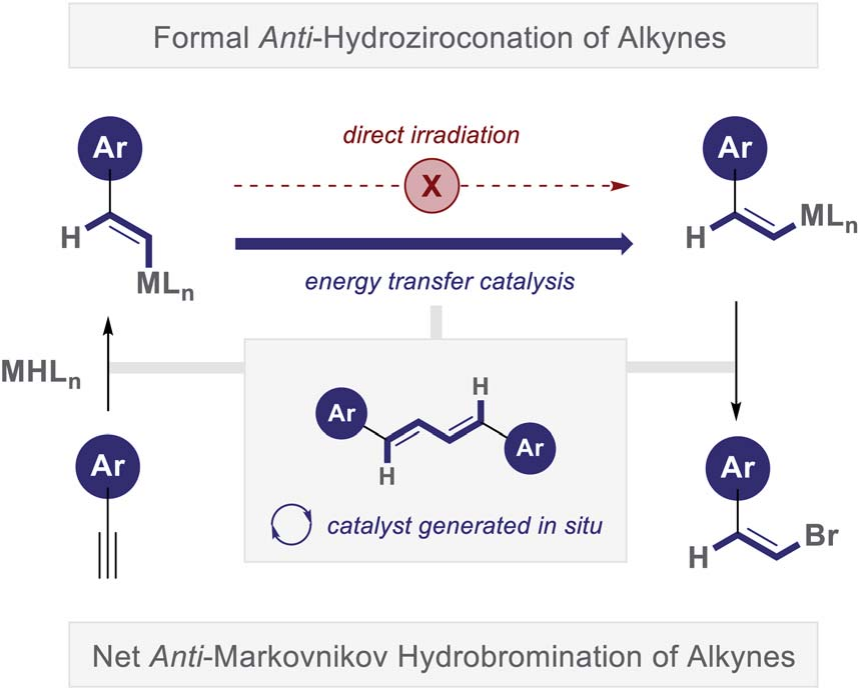
Postulated energy transfer catalysis cycle predicated on *in situ* formation of a conjugated diene photocatalyst.

## Data availability

All data is available in the ESI.

## Author contributions

TH, TN and RG designed the project and TH and TN conducted the experimental work. All authors contributed to writing the manuscript.

## Conflicts of interest

There are no conflicts to declare.

## Supplementary Material

SC-012-D1SC02454J-s001
