# Supplementary material for: Illuminating anti-hydrozirconation: controlled geometric isomerization of an organometallic species
Source: Chem Sci. 2021 Jul 9;12(31):10643–8. doi: 10.1039/d1sc02454j (PMC8356810; doi:10.1039/d1sc02454j)

Electronic Supplementary Material (ESI) for Chemical Science.
This journal is © The Royal Society of Chemistry 2021

Illuminating *Anti*-Hydrozirconation: Controlled Geometric Isomerization of an Organometallic Species by *in situ* Photocatalyst Generation

Theresa Hostmann, Tomáš Neveselý, Ryan Gilmour*

Organisch-Chemisches Institut, Westfälische Wilhelms-Universität Münster, Corrensstraße 36, 48149 Münster, Germany

- General Information 2
- General Procedure A: Hydrozirconation-Isomerisation-Bromination Sequence 4
- General Procedure B: Synthesis of (*E*)-Vinyl Bromides 4
- General Procedure C: Synthesis of (*Z*)-Vinyl Bromides 4
- Synthesis and Characterisation of the Vinyl Bromides 5
- Scope of Electrophiles 17
- Hydrozirconation-Isomerisation-Transmetallation Sequence 18
- NMR Analysis of the Transient Vinyl Zirconium Species 18
- Independent Synthesis of the diene 19
- Reaction Optimisation 20
- Synthesis of Alkynes 22
- Mechanistic Investigations 24
- UV/Vis-Absorption Spectra 28
- Fluorescence Spectra 29
- Computational Details 30
- References 35
- NMR Spectra 38

General Information

All chemicals were purchased as reagent grade and used without further purification. Solvents for purification (extraction and column chromatography) were purchased as technical grade and distilled using a rotary evaporator prior to use, with the exception of diethyl ether which was purchased as reagent grade and used as received. Dry solvents for reactions were taken from a Grubbs purification system that included columns packed with molecular sieves and aluminium oxide and were stored on molecular sieves under argon. Prior to use, the dry solvents were degassed by bubbling argon through them for ca. 15 min. The stationary phase for column chromatography was SiO_2-60 (230-400 mesh ASTM; *Fluka*). Analytical thin layer chromatography (TLC) was performed on glass plates pre-coated with SiO_2-60 F254 (*Merck*) and visualised with a UV-lamp (254 nm) and/or a $KMnO_4$ solution or I_2 on silica gel. Concentration under reduced pressure was performed at ca. 10 mbar and 40 °C, drying *in vacuo* at ca. 10^{-2} mbar and room temperature. NMR spectra were measured by the NMR service of the Organisch-Chemisches Institut, Westfälische Wilhelms-Universität Münster on a *Bruker Avance II 300*, a *Bruker Avance II 400*, an *Agilent DD2 500* or an *Agilent DD2 600* spectrometer at room temperature. ^{1}H NMR spectra are reported as follows: chemical shift δ in ppm (multiplicity, coupling constant *J* in Hz, number of protons, assignment of proton). The deuterated solvent residual peak was used as an internal reference: $CDCl_3$ (δ_H = 7.26 ppm), DMSO-d_6 (δ_H = 3.33 ppm). ^{13}C NMR spectra are reported as follows: chemical shift δ in ppm (multiplicity if different from singlet, coupling constant *J* in Hz, assignment of carbon). The solvent residual peak was used as an internal reference: $CDCl_3$ (δ_C = 77.16 ppm), DMSO-d_6 (δ_C = 39.52 ppm). ^{19}F NMR spectra are reported unreferenced as follows: chemical shift δ in ppm (multiplicity if different from singlet, coupling constant *J* in Hz). The resonance multiplicity is abbreviated as: s (singlet), d (doublet), t (triplet), q (quartet), m (multiplet). Assignments of compounds are based on COSY (HH), HMBC, HSQC, NOESY, TOCSY spectra. Melting points were measured on a *Büchi B-545* melting-point apparatus in open capillaries and are uncorrected. IR spectra were recorded on a *Perkin-Elmer 100 FT-IR* spectrometer, selected adsorption bands are reported in wavenumbers v in cm^{-1} and intensities are reported as w (weak), m (medium) and s (strong). Mass spectra (HR-ESI, GC-EI) were measured by the MS service of the Organisch-Chemisches Institut, Westfälische Wilhelms-Universität Münster on a *Bruker Daltonics MicrOTof ESI*, a *Thermo Fisher Scientific Orbitrap LTQ XL* or a *Thermo Fisher Scientific ISQ* 7000 *GC-MS*. The standard isomerisation reactions at 400 nm were performed using the set up shown in Figure 1 a) and b) with the LEDs specified in Table 1. Optimisation reactions at 375 nm, 385 nm, 431 nm and 435 nm were performed using a similar set up (Figure 1 a) and b)) and reactions at 365 nm and 525 nm using the set up shown in Figure 1 c). Specifications of all LEDs are listed in Table 1 and emission spectra are shown in Figure 2. In all cases the distance between the LED and the reaction vessel was set to ca. 1.5 cm. Determination of the *Z*:*E*-ratio was accomplished by integration of the ^{1}H NMR signal of the proton β to the aromatic ring (labelled as C1 in the NMR data). UV absorption spectra were recorded on an *Agilent Cary* 60 *UV-Vis* spectrophotometer and fluorescence spectra on a *Jasco FP-8300* spectrofluorometer. The Schwartz reagent was purchased from Tokyo Chemical Industry Co., Ltd. and the alkynes **A-1-3**, **A-5-15** and **A-22-24** from *Fluorochem Ltd.*, *Sigma-Aldrich Chemie GmbH or BLD-Pharmatech Ltd*.

Table 1: Specification of the LEDs.

**λ**	**supplier**	**article number**[a]	**power**	**forward current per chip**	**forward voltage**	**radiation flux**	**beam angle**
**400 nm**	**Avonec**	**3W390400m**	**3 W**	750 mA	3.5 - 4.5 V	900-1000 mW	120 °
369 nm	-	-	3 W	750 mA	3.3 – 3.7 V	-	-
374 nm	Avonec	3W370380m	3 W	750 mA	3.5 - 4.5 V	900-1000 mW	120 °
383 nm	Avonec	3W380390m	3 W	750 mA	3.5 - 4.5 V	900-1000 mW	120 °
414 nm	Avonec	3W430435m	3 W	750 mA	3.5 - 4.5 V	900-1000 mW	120 °
435 nm	Avonec	3W440450m	3 W	750 mA	3.5 - 4.5 V	20-40 lm[b]	120 °
520 nm	-	-	5 W	1330 mA	3.7 – 4.2 V	-	-

[a]3 W High Power LED auf Starplatine; [b]luminous flux

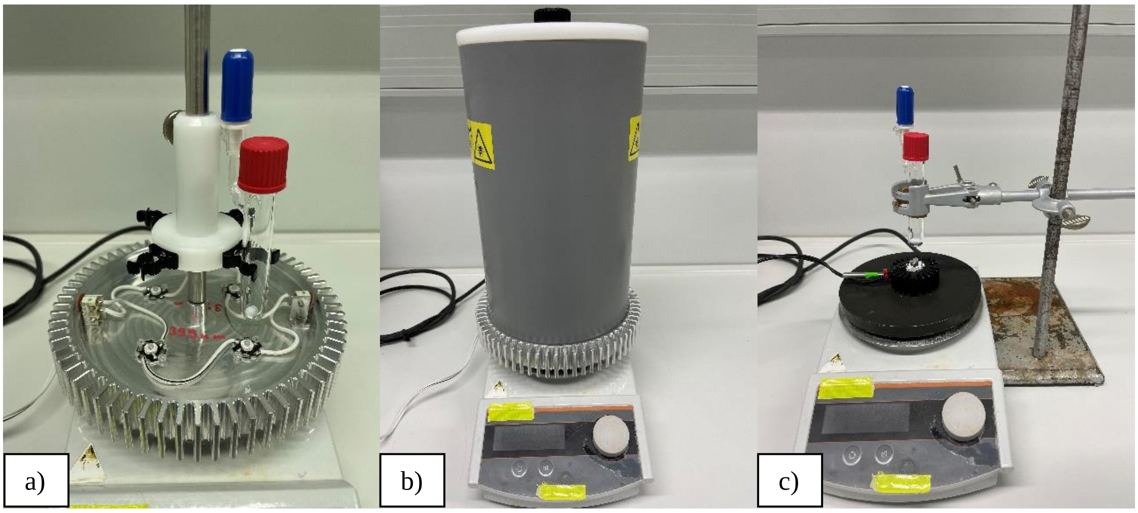

Figure 1: Exemplary LED set ups: a) open set up for irradiation at 374 nm, 383 nm, 400 nm, 414 nm and 435 nm; b) closed set up for irradiation at 374 nm, 383 nm, 400 nm, 414 nm and 435 nm; c) set up for irradiation at 369 nm and 520 nm.

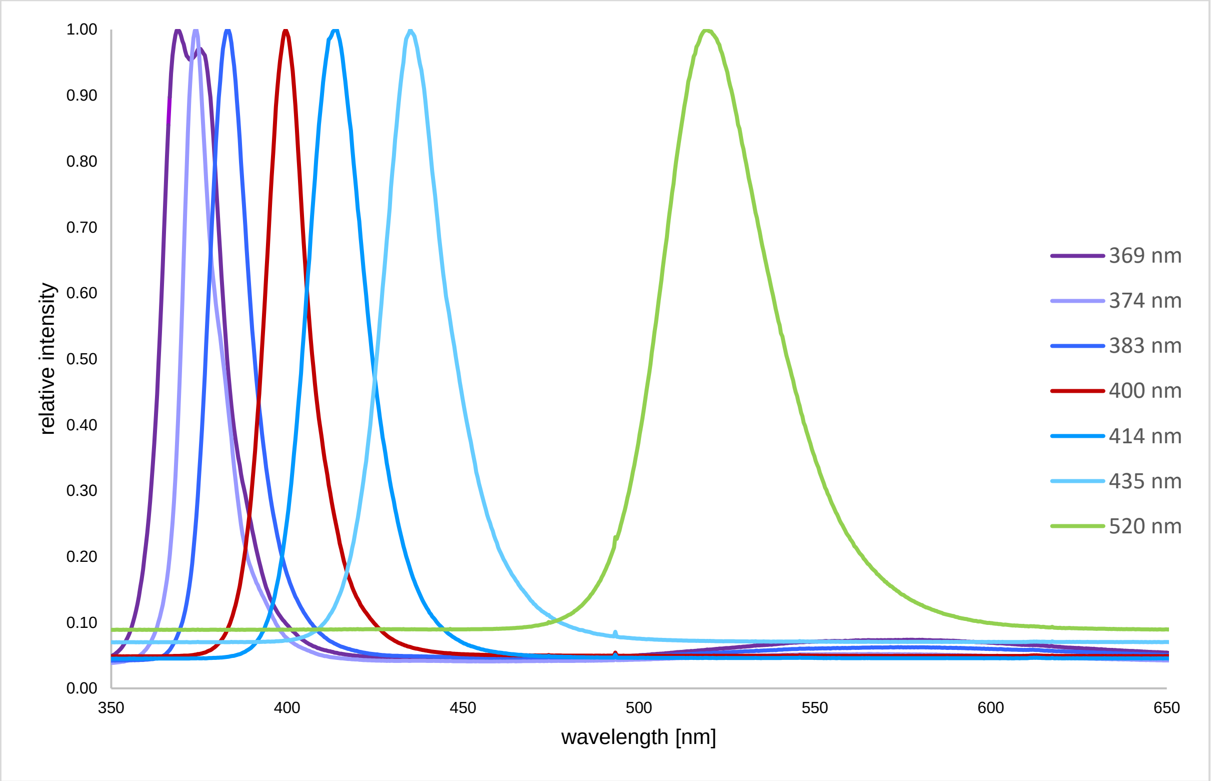

Figure 2: Emission spectra of the utilised LEDs.

General Procedure A: Hydrozirconation-Isomerisation-Bromination Sequence
An oven-dried pressure tube (10 mL, cooled to room temperature under vacuum and refilled with argon) was charged with Cp_2ZrHCl (62 mg, 0.24 mmol, 1.2 eq.), evacuated and refilled with argon. Dry CH_2Cl_2 (1.5 mL) and the alkyne (0.20 mmol, 1.0 eq.), dissolved in CH_2Cl_2 (0.5 mL, 0.1 M in total), were added under argon at room temperature. The mixture was stirred at room temperature for 15 min. (By this time the colourless suspension had turned into an yellow, clear solution, an indication of complete hydrozirconation.[1]) The pressure tube was placed in the LED set up (Figure 1) and irradiated for 45 min at 400 nm. The yellow reaction mixture was quenched by addition of NBS (39 mg, 0.22 mmol, 1.1 eq.) at room temperature and stirred for 15 min at room temperature. Upon NBS addition the colour of the reaction mixture was brighten up significantly, a sign for the replacement of the Zr by a Br atom. Water (10 mL) was added and the separated aqueous layer was extracted with CH_2Cl_2 (3 x 10 mL). The combined organic layers were washed with water (10 mL), dried over $MgSO_4$, filtered and concentrated under reduced pressure. The crude mixture was filtered over a large plug of silica rinsed with *n*-pentane to give the isomeric mixture of the *Z* and *E* vinyl bromide after removal of the solvent under reduced pressure. Drying under high vacuum was avoided due to the compounds' volatility. Determination of the *Z*:*E* ratio was accomplished by integrating the proton signals β to the aromatic ring in the ^{1}H NMR.
Attempts to separate the *Z* and *E* isomer by column chromatography were not successful. To verify the outcome of the photoisomerisation, both isomers were synthesised individually (see spectra at the end of this document). The NMR data are listed for the isomeric mixture resulting from the photoisomerisation but assignments and further analysis of the pure *E*- and *Z*-isomer were performed using these individually-synthesised isomers.

General Procedure B: Synthesis of (*E*)-Vinyl Bromides
An oven dried round bottom flask (cooled to room temperature under vacuum and refilled with argon) was charged with Cp_2ZrHCl (77 mg, 0.30 mmol, 1.5 eq.), evacuated and refilled with argon. Dry CH_2Cl_2 (1.5 mL) and the alkyne (0.20 mmol, 1.0 eq.), dissolved in CH_2Cl_2 (0.5 mL, 0.1 M in total), were added under argon at room temperature. The mixture was stirred at room temperature for 15 min. By this time the colourless suspension had turned into an orange, clear solution, an indication of complete hydrozirconation.[1] The reaction mixture was quenched by addition of NBS (39 mg, 0.22 mmol, 1.1 eq.) at room temperature and stirred for 15 min at room temperature. Upon NBS addition the colour of the reaction mixture was diluted significantly, a sign for the replacement of the Zr by a Br atom. The solvent was removed under reduced pressure and the (*E*)-vinyl bromide was afforded after column chromatography on SiO_2 with *n*-pentane as solvent. (N.b.: High vacuum drying should be avoided for most of the compounds due to their volatility.)

General Procedure C: Synthesis of (*Z*)-Vinyl Bromides
In accordance with a modified literature procedure,[2,3] an oven-dried round bottom flask cooled *in vacuo* and refilled with argon was charged under a flow of argon with the corresponding aldehyde (1.0 mmol, 1.0 eq) and CH_2Cl_2 (2.5 mL, 0.4 M), and cooled to 0 °C. At 0 °C CBr_4 (663 mg, 2.0 mmol, 2.0 eq.) was added in one portion followed by portionwise addition of PPh_3 (1.03 g, 4.0 mmol, 4.0 eq.) over 15 min. The ice bath was removed and stirring continued for 90 min. *n*-Pentane and SiO_2 were added and the dry loaded crude was purified by filtration over a plug of silica rinsed with *n*-pentane. The resulting dibromide was sufficiently pure to be used in the following step without further analysis or purification. An oven-dried vial (3 mL), closed with a septum screw cap, was cooled *in vacuo*, refilled with argon and was charged with $Pd(PPh_3)_4$ (29 mg, 0.05 mmol, 0.025 eq.) under a flow of argon. The corresponding dibromide (0.5 mmol, 1.0 eq.) dissolved in CH_2Cl_2 (1 mL, 0.5 M) was added (under a flow of argon) and the mixture was cooled to 0 °C. At 0 °C, $SnBu_3H$ (0.16 mL, 0.6 mmol, 1.2 eq.) was added dropwise through the septum and stirring was continued at room temperature for 90 min. The solvent was removed under reduced pressure and purification by column chromatography (*n*-pentane) afforded the (*Z*)-vinyl bromide.

Synthesis and Characterisation of the Vinyl Bromides

(*Z*,*E*)-1-Bromo-4-(2-bromovinyl)benzene ((*Z*,*E*)-1)

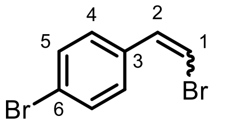

Prepared according to General Procedure A, 1-bromo-4-ethynylbenzene (**A-1**) (36 mg, 0.20 mmol, 1.0 eq.) was converted to an isomeric mixture of **(*Z*,*E*)-1**, a colourless solid (1st experiment: 38 mg, 0.15 mmol, 73%, *Z*:*E* = 73:27; 2nd experiment: 39.0 mg, 0.15 mmol, 74% *Z*:*E* = 72:28 , average: 74%, *Z*:*E* = 73:27).
^{1}H NMR (400 MHz, $CDCl_3$): δ = 7.58-7.43 (m, 3.46H, H_{Ar}), 7.19-7.14 (m, 0.54H, $H4_E$), 7.07-6.98 (m, 1H, H2), 6.78 (d, $^3J_{HH}$ = 14.0 Hz, 0.27H, $H1_E$), 6.47 (d, $^3J_{HH}$ = 8.1 Hz, 0.73H, $H1_Z$) ppm.

(*E*)-1-Bromo-4-(2-bromovinyl)benzene ((*E*)-1)

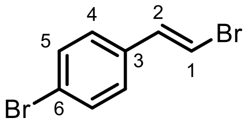

Prepared according to General Procedure B, 1-bromo-4-ethynylbenzene (**A-1**) (36 mg, 0.20 mmol, 1.0 eq.) was converted to **(*E*)-1**, a colourless solid (38 mg, 0.15 mmol, 73%).
R_f = 0.66 (*n*-pentane); **M.p.:** 68 °C; **^{1}H NMR** (400 MHz, $CDCl_3$): δ = 7.49-7.42 (m, 2H, H5), 7.20-7.15 (m, 2H, H4), 7.04 (d, $^3J_{HH}$ = 14.0 Hz, 1H, H2), 6.78 (d, $^3J_{HH}$ = 14.0 Hz, 1H, H1) ppm; **^{13}C{^{1}H} NMR** (101 MHz, $CDCl_3$): δ = 136.2 (C2), 134.9 (C3), 132.1 (C5), 127.7 (C4), 122.3 (C6), 107.5 (C1) ppm; **IR (ATR):** ν = 3074 (w), 1885 (w), 1717 (w), 1701 (w), 1606 (m), 1587 (m), 1562 (w), 1484 (m), 1397 (m), 1312 (w), 1291 (w), 1275 (w), 1225 (m), 1194 (m), 1182 (w), 1113 (w), 1091 (w), 1073 (s), 1008 (s), 944 (s), 928 (s), 823 (m), 768 (s), 745 (s), 693 (w) cm^{-1}; **GC-EI-MS:** *m/z*: 259.91 (55), 261.91 (100) 263.91 (52) ($[M]^+$, calcd. for $C_8H_6Br_2^+$: 259.88 (51), 261.88 (100), 263.88 (49)). The analytical data are in agreement with the literature data.[4]

(*Z*)-1-Bromo-4-(2-bromovinyl)benzene ((*Z*)-1)

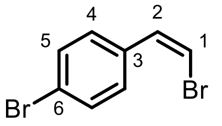

Prepared according to the second part of General Procedure C, 1-bromo-4-(2,2-dibromovinyl)benzene (170 mg, 0.50 mmol, 1.0 eq.) was converted to **(*Z*)-1**, a colourless liquid (39 mg, 0.15 mmol, 30%).
R_f = 0.66 (*n*-pentane); **^{1}H NMR** (400 MHz, $CDCl_3$): δ = 7.58-7.53 (m, 2H, H4), 7.52-7.48 (m, 2H, H5), 7.01 (d, $^3J_{HH}$ = 8.1 Hz, 1H, H2), 6.47 (d, $^3J_{HH}$ = 8.1 Hz, 1H, H1) ppm; **^{13}C{^{1}H} NMR** (101 MHz, $CDCl_3$): δ = 133.9 (C3), 131.6 (C5), 131.4 (C2), 130.6 (C4), 122.4 (C6), 107.5 (C1) ppm; **IR (ATR):** ν = 3076 (w), 1611 (w), 1587 (m), 1485 (s), 1400 (m), 1324 (m), 1308 (m), 1283 (w), 1216 (w), 1181 (w), 1109 (w), 1070 (m), 1010 (s), 946 (w), 919 (w), 836 (s), 815 (s), 775 (m), 729 (m), 710 (s), 677 (s) cm^{-1}; **GC-EI-MS:** *m/z*: 259.90 (55), 261.91 (100), 263.91 (50) ($[M]^+$, calcd. for $C_8H_6Br_2^+$: 259.88 (51), 261.88 (100), 263.88 (49)). The analytical data are in agreement with the literature data.[5]

(*Z*,*E*)-1-(2-Bromovinyl)-4-fluorobenzene ((*Z*,*E*)-2)

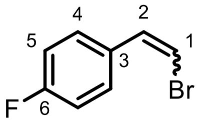

Prepared according to General Procedure A, 1-ethynyl-4-fluorobenzene (**A-2**) (24 mg, 0.20 mmol, 1.0 eq.) was converted to an isomeric mixture of **(*Z*,*E*)-2**, a colourless liquid (1st experiment: 28 mg, 0.14 mmol, 69%, *Z*:*E* = 74:26; 2nd experiment: 28 mg, 0.14 mmol, 68% *Z*:*E* = 72:28, average: 69%, *Z*:*E* = 73:27).
^{1}H NMR (400 MHz, $CDCl_3$): δ = 7.71-7.64 (m, 1.48H, $H4_Z$), 7.30-7.24 (m, 0.52H, $H4_E$), 7.10-6.99 (m, 3H, H2+H5), 6.69 (d, $^3J_{HH}$ = 14.0 Hz, 0.27H, $H1_E$), 6.42 (d, $^3J_{HH}$ = 8.1 Hz, 0.74H, $H1_Z$) ppm.

(*E*)-1-(2-Bromovinyl)-4-fluorobenzene ((*E*)-2)

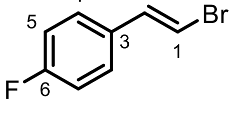

Prepared according to General Procedure B, 1-ethynyl-4-fluorobenzene (**A-2**) (24 mg, 0.20 mmol, 1.0 eq.) was converted to **(*E*)-2**, a colourless liquid (17 mg, 0.09 mmol, 43%).
R_f = 0.76 (*n*-pentane); **^{1}H NMR** (599 MHz, $CDCl_3$): δ = 7.29-7.25 (m, 2H, H4), 7.07 (d, $^3J_{HH}$ = 14.0 Hz, 1H, H2), 7.04-6.99 (m, 2H, H5), 6.70 (d, $^3J_{HH}$ = 14.0 Hz, 1H, H1) ppm; **^{13}C{^{1}H} NMR** (151 MHz, $CDCl_3$): δ = 162.8 (d, $^1J_{CF}$ = 248.2 Hz, C6), 136.1 (d, $^5J_{CF}$ = 0.6 Hz, C2), 132.3 (d, $^4J_{CF}$ = 3.5 Hz, C3), 127.9 (d, $^3J_{CF}$ = 8.1 Hz, C4), 116.0 (d, $^2J_{CF}$ = 21.7 Hz, C5), 106.3 (d, $^6J_{CF}$ = 2.3 Hz, C1) ppm; **^{19}F NMR** (564 MHz, $CDCl_3$): δ = -113.01 (tt, J_{HF} = 8.6, 5.3 Hz) ppm; **IR (ATR):** ν = 1600 (m), 1588 (w), 1506 (s), 1236 (m), 1223 (s), 1185 (m), 1158 (m), 1095 (w), 945 (m), 927 (s), 856 (w), 837 (s), 787 (s), 775 (s), 729 (w), 700 (w) cm^{-1}; **GC-EI-MS:** *m/z*: 199.99 (100) 202.04 (92) ($[M]^+$, calcd. for $C_8H_6F^+$:199.96 (100) 201.96 (98)). The analytical data are in agreement with the literature data.[6]

(*Z*)-1-(2-Bromovinyl)-4-fluorobenzene ((*Z*)-2)

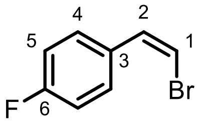

Prepared according to General Procedure C, 4-fluorobenzaldehyde (124 mg, 1.00 mmol, 1.0 eq.) yielded 1-(2,2-dibromovinyl)-4-fluorobenzene (192 mg, 0.69 mmol, 69%), of which 140 mg, 0.50 mmol, 1.0 eq. was converted to **(*Z*)-2**, a colourless liquid (7 mg, 0.03 mmol, 3%).

R_f = 0.76 (*n*-pentane); **^{1}H NMR** (599 MHz, $CDCl_3$): δ = 7.70-7.65 (m, 2H, H4), 7.09-7.04 (m, 2H, H5), 7.03 (d, $^3J_{HH}$ = 8.1 Hz, 1H, H2), 6.42 (d, $^3J_{HH}$ = 8.1 Hz, 1H, H1) ppm; **^{13}C{^{1}H} NMR** (151 MHz, $CDCl_3$): δ = 162.5 (d, $^1J_{CF}$ = 248.8 Hz, C6), 131.4 (C2), 131.2 (d, $^4J_{CF}$ = 3.5 Hz, C3), 131.0 (d, $^3J_{CF}$ = 8.1 Hz, C4), 115.4 (d, $^2J_{CF}$ = 21.7 Hz, C5), 106.4 (d, $^6J_{CF}$ = 2.0 Hz) ppm; **^{19}F NMR** (376 MHz): δ = -112.41 (tt, J_{HF} = 8.5 , 5.4 Hz) ppm; **IR (ATR):** ν = 1601 (m), 1505 (s), 1464 (w), 1410 (w), 1326 (m), 1294 (w), 1232 (s), 1159 (m), 1095 (w), 1014 (w), 945 (w), 929 (w), 854 (m), 836 (s), 781 (m), 731 (m), 684 (m); **GC-EI-MS:** *m/z*: 200.04 (92), 202.00 (100) ([M]$^+$, calcd. for $C_8H_6F^+$:199.96 (100) 201.96 (98)). The analytical data are in agreement with the literature data.[5]

(*Z*,*E*)-1-(2-Bromovinyl)-4-chlorobenzene ((*Z*,*E*)-3)

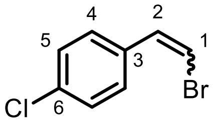

Prepared according to General Procedure A, 1-chloro-ethynylbenzene (**A-3**) (27 mg, 0.20 mmol, 1.0 eq.) was converted to an isomeric mixture of **(*Z*,*E*)-3**, a colourless liquid (1st experiment: 36 mg, 0.16 mmol, 82%, *Z*:*E* = 73:27; 2nd experiment: 35 mg, 0.16 mmol, 80% *Z*:*E* = 73:27, average: 81%, *Z*:*E* = 73:27).

^{1}H NMR (400 MHz, $CDCl_3$): δ = 7.62 (d, $^3J_{HH}$ = 8.5 Hz, 1.46H, H4$_Z$), 7.39-7.19 (m, 2.54H, H4$_E$, H5), 7.10-6.99 (m, 1H, H2), 6.77 (d, $^3J_{HH}$ = 14.0 Hz, 0.27H, H1$_E$), 6.46 (d, $^3J_{HH}$ = 8.1 Hz, 0.73H, H1$_Z$) ppm.

(*E*)-1-(2-Bromovinyl)-4-chlorobenzene ((*E*)-3)

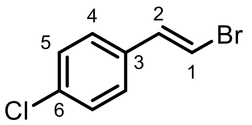

Prepared according to General Procedure B, 1-chloro-4-ethynylbenzene (**A-3**) (27 mg, 0.20 mmol, 1.0 eq.) was converted to **(*E*)-3**, a colourless solid (26 mg, 0.12 mmol, 59%).

R_f = 0.66 (*n*-pentane); **M.p.:** 46 °C; **^{1}H NMR** (599 MHz, $CDCl_3$): δ = 7.32-7.27 (m, 2H, H5), 7.25-7.21 (m, 2H, H4), 7.06 (d, $^3J_{HH}$ = 14.0 Hz, 1H, H2), 6.77 (d, $^3J_{HH}$ = 14.0 Hz, 1H, H1) ppm; **^{13}C{^{1}H} NMR** (126 MHz, $CDCl_3$): δ = 136.1 (C2), 134.5 (C3), 134.2 (C6), 129.2 (C5), 127.4 (C4), 107.3 (C1) ppm; **IR (ATR):** ν = 3075 (w), 2924 (w), 2853 (w), 1899 (w), 1883 (w), 1718 (w), 1702 (w), 1644 (w), 1609 (w), 1591 (w), 1566 (w), 1488 (m), 1401 (m), 1273 (w), 1227 (m), 1193 (w), 1179 (w), 1113 (w), 1098 (s), 1081 (m), 1011 (m), 945 (m), 928 (s), 830 (m), 770 (s) 749 (s), 695 (w), 655 (w); **GC-EI-MS:** *m/z*: 215.97 (80), 217.97 (100), 219.97 (25) ([M]$^+$, calcd. for $C_8H_6ClBr^+$: 215.93 (77), 217.93 (100), 219.93 (24)). The analytical data are in agreement with the literature data.[4]

(*Z*)-1-(2-Bromovinyl)-4-chlorobenzene ((*Z*)-3)

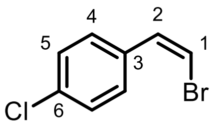

Prepared according to General Procedure C, 4-chlorobenzaldehyde (140 mg, 1.00 mmol, 1.0 eq.) yielded 1-chloro-4-(2,2-dibromovinyl)benzene (216 mg, 0.74 mmol, 74%), of which 148 mg, 0.50 mmol, 1.0 eq. was converted to **(*Z*)-3**, a colourless liquid (91 mg, 0.42 mmol, 84%).

R_f = 0.66 (*n*-pentane); **^{1}H NMR** (500 MHz, $CDCl_3$): δ = 7.64-7.60 (m, 2H, H4), 7.37-7.33 (m, 2H, H5), 7.02 (d, $^3J_{HH}$ = 8.2 Hz, 1H, H2), 6.46 (d, $^3J_{HH}$ = 8.2 Hz, 1H, H1) ppm; **^{13}C{^{1}H} NMR** (126 MHz, $CDCl_3$): δ = 134.2 (C6), 133.5 (C3), 131.4 (C2), 130.4 (C4), 128.6 (C5), 107.3 (C1) ppm; **IR (ATR):** ν = 1614 (w), 1588 (w), 1488 (s), 1401 (m), 1325 (s), 1309 (m), 1285 (w), 1110 (w), 1093 (s), 1013 (s), 946 (w), 919 (w), 839 (s), 820 (s), 719 (s), 679 (s) cm^{-1}; **GC-EI-MS:** *m/z*: 215.97 (80), 217.97 (100), 219.97 (20) ([M]$^+$, calcd. for $C_8H_6ClBr^+$: 215.93 (77), 217.93 (100), 219.03 (24)). The analytical data are in agreement with the literature data.[5]

(*Z*,*E*)-1-(2-Bromovinyl)-4-iodobenzene ((*Z*,*E*)-4)

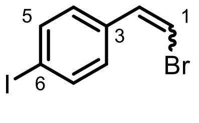

Prepared according to General Procedure A, 1-ethynyl-4-iodobenzene (**A-4**) (46 mg, 0.20 mmol, 1.0 eq.) was converted to an isomeric mixture of **(*Z*,*E*)-4**, a colourless liquid (1st experiment: 47 mg, 0.15 mmol, 76%, *Z*:*E* = 75:25; 2nd experiment: 47 mg, 0.15 mmol, 76% *Z*:*E* = 73:27, average: 76%, *Z*:*E* = 74:26).

^{1}H NMR (400 MHz, $CDCl_3$): δ = 7.75-7.62 (m, 2H, H5), 7.42 (d, $^3J_{HH}$ = 8.3 Hz, 1.50H, H4$_Z$), 7.06-6.97 (m, 1.50H, H4$_E$, H2), 6.80 (d, $^3J_{HH}$ = 14.0 Hz, 0.25H, H1$_E$), 6.47 (d, $^3J_{HH}$ = 8.1 Hz, 0.75H, H1$_Z$) ppm.

(*E*)-1-(2-Bromovinyl)-4-iodobenzene ((*E*)-4)

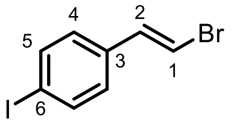

Prepared according to General Procedure B, 1-ethynyl-4-iodobenzene (**A-4**) (46 mg, 0.20 mmol, 1.0 eq.) was converted to **(*E*)-4**, a colourless solid (22 mg, 0.07 mmol, 36%).

R_f = 0.66 (*n*-pentane); **M.p.:** 82 °C; **^{1}H NMR** (500 MHz, $CDCl_3$): δ = 7.68-7.63 (m, 2H, H5), 7.06-6.99 (m, 3H, H4+H2), 6.80 (d, $^3J_{HH}$ = 14.0 Hz, 1H, H1) ppm; **^{13}C{^{1}H} NMR** (126 MHz, $CDCl_3$): δ = 138.1 (C5), 136.3 (C2), 135.5 (C3), 127.9 (C4), 107.6 (C1), 93.9 (C4) ppm; **IR (ATR):** ν = 3067 (w), 1702 (w), 1607 (m), 1580 (m), 1553 (w), 1481 (m), 1392 (m), 1315 (w), 1292 (w), 1272 (w), 1221 (w), 1193 (w), 1180 (m), 1112 (w), 1060 (m), 1003 (m), 948 (m), 929 (s), 830 (m), 768 (s), 743 (s), 694 (w); **GC-EI-MS:** *m/z*: 307.88 (100), 309.90 (92) ([M]$^+$, calcd. for $C_8H_6IBr^+$: 307.87 (100), 309,87 (98)). The analytical data are in agreement with the literature data.[7]

(*Z*,*E*)-1-(2-Bromovinyl)-4-iodobenzene ((*Z*)-4)

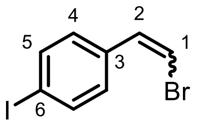

The application of General Procedure C resulted in an inseparable mixture of the desired **(*Z*)-4** and the deiodinated compound **(*Z*)-4**. Therefore, characterisation is based on the isomeric mixture resulting from the photoisomerisation (General Procedure A) and NMR data are listed for the *Z* isomer only.

R_f = 0.66 (*n*-pentane); **M.p.:** 31 °C; **^{1}H NMR** (599 MHz, $CDCl_3$): δ = 7.73-7.69 (m, 2H, H5), 7.44-7.40 (m, 2H, H4), 6.99 (d, $^3J_{HH}$ 8.2 Hz, 1H, H2), 6.47 (d, $^3J_{HH}$ = 8.1 Hz, 1H, H1) ppm; **^{13}C{^{1}H} NMR** (151 MHz, $CDCl_3$): δ = 137.5 (C5), 134.5 (C3), 131.5 (C2), 130.8 (C4), 107.6 (C1), 94.2 (C6) ppm; **IR (ATR):** ν = 3075 (w), 2918 (w),1917 (w), 1845 (w), 1793 (w), 1761 (w), 1654 (w), 1606 (w), 1577 (w), 1551 (w), 1481 (m), 1414 (w), 1393 (m), 1367 (w), 1329 (m), 1311 (m), 1287 (w), 1272 (w), 1247 (w), 1216 (w), 1194 (w), 1180 (w), 1116 (w), 1061 (w), 1003 (m), 949 (w), 933 (m), 894 (w), 827 (s), 817 (s), 772 (s), 746 (m), 734 (w), 702 (m), 682 (s) cm^{-1}; **GC-EI-MS:** *m/z*: 307.88 (100), 309.89 (94) ([M]$^+$, calcd. for $C_8H_6IBr^+$: 307.87 (100), 309.87 (98)). The analytical data are in agreement with the literature data.[8]

(*Z*,*E*)-1-(2-Bromovinyl)-2-fluorobenzene((*Z*,*E*)-5)

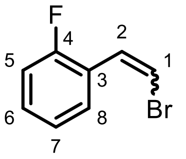

Prepared according to General Procedure A, 1-ethynyl-2-fluorobenzene (**A-5**) (24 mg, 0.20 mmol, 1.0 eq.) was converted to an isomeric mixture of **(*Z*,*E*)-5**, a colourless liquid (1st experiment: 8 mg, 0.04 mmol, 20%, *Z*:*E* = 59:41; 2nd experiment: 9 mg, 0.04 mmol, 22% *Z*:*E* = 60:40, average: 21%, *Z*:*E* = 60:40).

^{1}H NMR (400 MHz, $CDCl_3$): δ = 8.03-7.95 (m, 0.60H, $H8_Z$), 7.35-7.01 (m, 3.40H, H5+H6+H7+$H8_E$), 6.94 (d, $^3J_{HH}$ = 14.1 Hz, 0.40H, $H1_E$), 6.56 (d, $^3J_{HH}$ = 8.1 Hz, 0.60H, $H1_Z$) ppm.

(*E*)-1-(2-Bromovinyl)-2-fluorobenzene((*E*)-5)

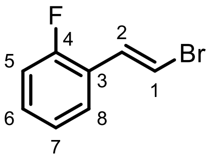

Prepared according to General Procedure B, 1-ethynyl-2-fluorobenzene (**A-5**) (24 mg, 0.20 mmol, 1.0 eq.) was converted to **(*E*)-5**, a colourless liquid (3 mg, 0.02 mmol, 8%).

R_f = 0.55 (*n*-hexane); **^{1}H NMR** (500 MHz, $CDCl_3$): δ = 7.33-7.29 (m, 1H, H8), 7.29-7.24 (m, 2H, H6), 7.19 (d, $^3J_{HH}$ = 14.1 Hz, 1H, H2), 7.14-7.08 (m, 1H, H7), 7.08-7.03 (m, 1H, H5), 6.94 (d, $^3J_{HH}$ = 14.1 Hz, 1H, H1) ppm; **^{13}C{^{1}H} NMR** (101 MHz, $CDCl_3$): δ = 159.9 (d, $^1J_{CF}$ = 253.4 Hz, C4), 130.6 (d, $^3J_{CF}$ = 2.3 Hz, C2), 129.7 (d, $^3J_{CF}$ = 8.5 Hz, C6), 128.1 (d, $^3J_{CF}$ = 3.6 Hz, C8), 124.5 (d, $^4J_{CF}$ = 3.6 Hz, C7), 123.8 (d, $^3J_{CF}$ = 12.2 Hz, C2), 116.2 (d, $^2J_{CF}$ = 22.0 Hz, C5), 109.9 (d, $^4J_{CF}$ = 8.1 Hz) ppm; **^{19}F NMR** (470 MHz, $CDCl_3$): δ = -116.25 ppm; **IR (ATR):** ν = 2926 (w), 1603 (w), 1575 (w), 1492 (m), 1485 (s), 1455 (m), 1326 (w), 1271 (w), 1234 (m), 1214 (s), 1189 (w), 1163 (w), 1128 (w), 1108 (w), 1093 (w), 1068 (w), 1035 (w), 943 (s), 934 (s), 879 (w), 856 (w), 810 (w), 769 (w), 750 (s), 729 (s), 708 (m) cm^{-1}; **GC-EI-MS:** *m/z*: 199.99 (100), 202.00 (93) ([M]$^+$, calcd. $C_8H_6F^+$:199.96 (100) 201.96 (98)). The analytical data are in agreement with the literature data.[4]

(*Z*)-1-(2-Bromovinyl)-2-fluorobenzene ((*Z*)-5)

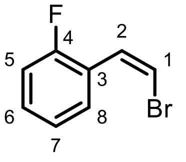

Prepared according to General Procedure C, 2-fluorobenzaldehyde (149 mg, 1.00 mmol, 1.0 eq.) yielded (2,2-dibromovinyl)benzene (272 mg, 0.98 mmol, 98%), of which 140 mg, 0.50 mmol, 1.0 eq. was converted to **(*Z*)-5**, a colourless liquid (9 mg, 0.05 mmol, 9%).

R_f = 0.55 (*n*-hexane); **^{1}H NMR** (500 MHz, $CDCl_3$): δ = 8.01-7.96 (m, 1H, H8), 7.35-7.29 (m, 1H, H6), 7.22 (d, $^3J_{HH}$ = 8.2 Hz, 1H, H2), 7.19-7.15 (m, 1H, H7), 7.13-7.04 (m, 1H, H5), 6.57 (d, $^3J_{HH}$ = 8.2 Hz, 1H, H1) ppm; **^{13}C{^{1}H} NMR** (126 MHz, $CDCl_3$): δ = 160.2 (d, $^1J_{CF}$ = 249.9 Hz, C4), 130.1 (d, $^3J_{CF}$ = 8.6 Hz, C6), 129.8 (d, $^3J_{CF}$ = 2.9 Hz, C8), 125.4 (d, $^3J_{CF}$ = 5.7 Hz, C2), 123.7 (d, $^4J_{CF}$ = 3.8 Hz, C7), 123.1 (d, $^2J_{CF}$ = 12.5 Hz, C3), 115.5 (d, $^2J_{CF}$ = 21.9 Hz, C5), 109.3

(C1) ppm; **^{19}F NMR** (470 MHz, $CDCl_3$): δ = -115.22--115.28 (m) ppm; **IR (ATR):** ν = 1483 (m), 1456 (s), 1377 (m), 1306 (w), 1232 (m), 1193 (w), 1152 (m), 1096 (m), 1036 (w), 989 (m), 941 (w), 862 (w), 839 (w), 807 (w), 796 (w), 753 (s), 724 (w), 690 (w), 671 (w), 661 (w) cm^{-1}; **GC-EI-MS:** *m/z*: 200.02 (100), 202.00 (98) ($[M]^+$, calcd. $C_8H_6F^+$:199.96 (100) 201.96 (98)). The analytical data are in agreement with the literature data.[9]

(*Z*,*E*)-1-Bromo-3-(2-bromovinyl)benzene ((*Z*,*E*)-6)

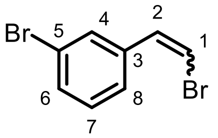

Prepared according to General Procedure A, 3-bromo-1-ethynylbenzene (**A-6**) (36 mg, 0.20 mmol, 1.0 eq.) was converted to an isomeric mixture of **(*Z*,*E*)-6**, a colourless liquid (1st experiment: 38 mg, 0.14 mmol, 72%, *Z*:*E* = 66:34; 2nd experiment: 39 mg, 0.15 mmol, 75% *Z*:*E* = 68:32, average: 74%, *Z*:*E* = 67:33).
^{1}H NMR (400 MHz, $CDCl_3$): δ = 7.82 (t, $^3J_{HH}$ = 1.9 Hz, 0.68H, $H4_Z$), 7.61-7.57 (m, 0.68H, $H8_Z$), 7.47-7.39 (m, 1.32H, $H4_E$+H6), 7.27-7.16 (m, 1.32H, H7+$H8_E$), 7.05-6.98 (m, 1H, H2), 6.80 (d, $^3J_{HH}$ = 14.0 Hz, 0.32H, $H1_E$), 6.49 (d, $^3J_{HH}$ = 8.2 Hz, 0.68H,$H1_Z$) ppm.

(*E*)-1-Bromo-3-(2-bromovinyl)benzene ((*E*)-6)

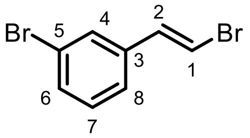

Prepared according to General Procedure B, 3-bromo-1-ethynylbenzene (**A-6**) (36 mg, 0.20 mmol, 1.0 eq.) was converted to **(*E*)-6**, a colourless liquid (29 mg, 0.11 mmol, 56%).
R_f = 0.67 (*n*-pentane); **^{1}H NMR** (599 MHz, $CDCl_3$): δ = 7.46-7.43 (m, 1H, H4), 7.43-7.40 (m, 1H, H6), 7.24-7.17 (m, 2H, H7+H8) , 7.04 (d, $^3J_{HH}$ = 14.0 Hz, 1H, H2), 6.81 (d, $^3J_{HH}$ = 14.0 Hz, 1H, H1) ppm; **^{13}C{^{1}H} NMR** (151 MHz, $CDCl_3$): δ = 138.0 (C3), 135.9 (C2), 131.3 (C6), 130.4 (C7), 129.1 (C4), 124.9 (C8), 123.1 (C5), 108.4 (C1) ppm; **IR (ATR):** ν = 1604 (m), 1592 (m), 1560 (m), 1489 (w), 1476 (m), 1469 (m), 1411 (m), 1401 (w), 1221 (m), 1188 (m), 1168 (w), 1091 (m), 1071 (m), 995 (m), 934 (s), 901 (w), 878 (m), 859 (m), 833 (w), 799 (m), 759 (s), 677 (s), 667 (s) cm^{-1}; **GC-EI-MS:** *m/z*: 259.90 (51), 261.91 (100), 263.91 (49) ($[M]^+$, calcd. for $C_8H_6Br_2^+$: 259.88 (51), 261.88 (100), 263.88 (49)). The analytical data are in agreement with the literature data.[4]

(*Z*)-1-Bromo-3-(2-bromovinyl)benzene ((*Z*)-6)

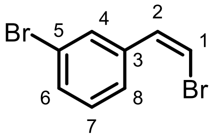

Prepared according to General Procedure C, 3-bromobenzaldehyde (185 mg, 1.00 mmol, 1.0 eq.) yielded 1-bromo-3-(2,2-dibromovinyl)benzene (264 mg, 0.78 mmol, 78%), of which 170 mg, 0.50 mmol, 1.0 eq. was converted to **(*Z*)-6**, a colourless liquid (99 mg, 0.38 mmol, 76%).
R_f = 0.67 (*n*-pentane); **^{1}H NMR** (500 MHz, $CDCl_3$): δ = 7.83 (t, $^4J_{HH}$ = 1.8 Hz, 1H, H4), 7.64-7.58 (m, 1H, H8), 7.48-7.44 (m, 1H, H6), 7.25 (t, $^3J_{HH}$ = 7.9 Hz, 1H, H7), 7.01 (d, $^3J_{HH}$ = 8.2 Hz, 1H, H2), 6.50 (d, $^3J_{HH}$ = 8.2 Hz, 1H, H1) ppm; **^{13}C{^{1}H} NMR** (126 MHz, $CDCl_3$): δ = 137.0 (C3), 131.9 (C4), 131.4 (C6), 131.2 (C2), 129.9 (C7), 127.7 (C8), 122.4 (C5), 108.2 (C1) ppm; **IR (ATR):** ν = 1615 (w), 1591 (w), 1558 (m), 1476 (m), 1470 (m), 1421 (w), 1404 (m), 1325 (m), 1164 (w), 1076 (m), 997 (m), 882 (m), 861 (m), 785 (s), 731 (s), 705 (m), 670 (s) cm^{-1}; **GC-EI-MS:** *m/z*: 259.91 (50), 261.90 (100), 263.90 (48) ($[M]^+$, calcd. for $C_8H_6Br_2^+$: 259.88 (51), 261.88 (100), 263.88 (49)). The analytical data are in agreement with the literature data.[10]

(*Z*,*E*)-(2-Bromovinyl)benzene ((*Z*,*E*)-7)

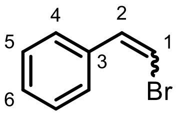

Prepared according to General Procedure A, phenylacetylene (**A-7**) (20 mg, 0.20 mmol, 1.0 eq.) was converted to an isomeric mixture of **(*Z*,*E*)-1**, a colourless liquid (1st experiment: 13 mg, 0.07 mmol, 36%, *Z*:*E* = 71:29; 2nd experiment: 13 mg, 0.07 mmol, 35%, *Z*:*E* = 73:27 , average: 36%, *Z*:*E* = 72:28).
^{1}H NMR (400 MHz, $CDCl_3$): δ = 7.72-7.65 (m, 1.46H, $H4_Z$), 7.43-7.25 (m, 3.54, H_{Ar}), 7.15-7.05 (m, 1H, H2), 6.77 (d, $^3J_{HH}$ = 14.0 Hz, 0.27H, $H1_E$), 6.44 (d, $^3J_{HH}$ = 8.1 Hz, 0.73H, $H1_Z$) ppm.

(*E*)-(2-Bromovinyl)benzene ((*E*)-7)

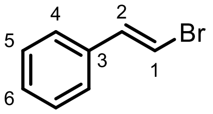

Prepared according to General Procedure B, phenylacetylene (**A-7**) (20 mg, 0.20 mmol, 1.0 eq.) was converted to **(*E*)-7**, a colourless liquid (25 mg, 0.14 mmol, 68%).
R_f = 0.52 (*n*-hexane); **^{1}H NMR** (500 MHz, $CDCl_3$): δ = 7.37-7.29 (m, 5H, H4-6), 7.12 (d, $^3J_{HH}$ = 14.0 Hz, 1H, H2), 6.78 (d, $^3J_{HH}$ = 14.0 Hz, 1H, H1) ppm; **^{13}C{^{1}H} NMR** (126 MHz, $CDCl_3$): δ = 137.3 (C2), 136.1 (C3), 128.9 (C5), 128.4 (C6), 126.2 (C4), 106.7 (C1) ppm; **IR (ATR):** ν = 3075 (w), 3024 (w), 1607 (m), 1574 (w), 1496 (m), 1445 (m), 1327 (w), 1280 (w), 1221 (m), 1185 (w), 1176 (w), 1072 (w), 1030 (w), 1001 (w), 983 (w), 937 (s), 837 (w), 772 (w), 727 (s), 688 (s) cm^{-1}; **GC-EI-MS:** *m/z*: 182.00 (100), 184.01 (96) ($[M]^+$, calcd. for $C_8H_7Br^+$: 181.97 (100), 183.97 (98)). The analytical data are in agreement with the literature data.[4]

(*Z*)-(2-Bromovinyl)benzene ((*Z*)-7)

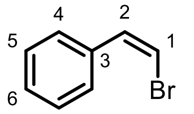

Prepared according to General Procedure C, benzaldehyde (0.1 mL, 1.00 mmol, 1.0 eq.) yielded (2,2-dibromovinyl)benzene (250 mg, 0.96 mmol, 96%), of which 131 mg, 0.50 mmol, 1.0 eq. was converted to **(*Z*)-7**, a colourless liquid (50 mg, 0.27 mmol, 55%).

R_f = 0.52 (*n*-hexane); **^{1}H NMR** (599 MHz, $CDCl_3$): δ = 7.71-7.65 (m, 2H, H4), 7.41-7.36 (m, 2H, H5), 7.35-7.30 (m, 1H, H6), 7.08 (d, $^3J_{HH}$ = 8.1 Hz, 1H, H2), 6.44 (d, $^3J_{HH}$ = 8.1 Hz, 1H, H1) ppm; **^{13}C{^{1}H} NMR** (151 MHz, $CDCl_3$): δ = 135.1 (C3), 132.5 (C2), 129.1 (C4), 128.5 (C6), 128.4 (C5), 106.5 (C1) ppm; **IR (ATR):** ν = 3025 (w), 1614 (w), 1490 (m), 1445 (m), 1316 (m), 1169 (w), 1073 (w), 1028 (w), 924 (m), 827 (m), 766 (s), 691 (s), 678 (s) cm^{-1}; **GC-EI-MS:** *m/z*: 182.01 (100), 184.01(95) ([M]$^+$, calcd. for $C_8H_7Br^+$: 181.97 (100), 183.97 (98)). The analytical data are in agreement with the literature data.[5]

(*Z*,*E*)-1-(2-Bromovinyl)-4-(*tert*-butyl)benzene ((*Z*,*E*)-8)

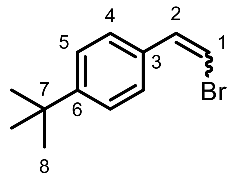

Prepared according to General Procedure A, 1-ethynyl-4-(*tert*)-butylbenzene (**A-8**) (32 mg, 0.20 mmol, 1.0 eq.) was converted to an isomeric mixture of **(*Z*,*E*)-8**, a colourless liquid (1st experiment: 39 mg, 0.16 mmol, 81%, *Z*:*E* = 81:19; 2nd experiment: 37 mg, 0.16 mmol, 78% *Z*:*E* = 81:19, average: 80%, *Z*:*E* = 81:19).

^{1}H NMR (400 MHz, $CDCl_3$): δ = 7.69-7.63 (m, 1.62H, $H4_Z$), 7.43-7.31 (m, 2H, H5), 7.26-7.22 (m, 0.38H, $H4_E$), 7.11-7.01 (m, 1H, H2), 6.72 (d, $^3J_{HH}$ = 13.9 Hz, 0.19H, $H1_E$), 6.38 (d, $^3J_{HH}$ = 8.1 Hz, 0.81H, $H1_Z$), 1.34 (s, 2.43H, $H8_Z$), 1.32 (s, 0.57H, $H1_E$) ppm.

(*E*)-1-(2-Bromovinyl)-4-(*tert*-butyl)benzene ((*E*)-8)

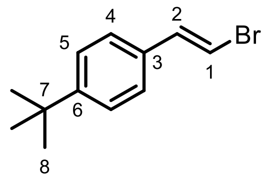

Prepared according to General Procedure B, 1-ethynyl-4-(*tert*)-butylbenzene (**A-8**) (32 mg, 0.20 mmol, 1.0 eq.) was converted to **(*E*)-8**, a colourless solid (26 mg, 0.11 mmol, 54%).

R_f = 0.52 (*n*-pentane); **M.p.:** 28 °C; **^{1}H NMR** (599 MHz, $CDCl_3$): δ = 7.38-7.32 (m, 2H, H5), 7.26-7.22 (m, 2H, H4), 7.09 (d, $^3J_{HH}$ = 14.0 Hz, 2H), 6.72 (d, $^3J_{HH}$ = 14.0 Hz, 1H, H1), 1.32 (s, 9H, H8) ppm; **^{13}C{^{1}H} NMR** (151 MHz, $CDCl_3$): δ = 151.6 (C6), 137.1 (C2), 133.3 (C3), 126.0 (C4), 125.9 (C5), 105.8 (C1), 34.8 (C7), 31.3 (C8) ppm; **IR (ATR):** ν = 2959 (m), 2864 (w), 1603 (w), 1517 (w), 1505 (w), 1459 (m), 1411 (w), 1389 (w), 1365 (m), 1360 (m), 1268 (m), 1228 (m), 1204 (w), 1183 (m), 1105 (m), 1021 (w), 956 (m), 934 (s), 854 (w), 838 (s), 827 (m), 780 (s), 749 (s), 724 (s), 666 (s) cm^{-1}; **GC-EI-MS:** *m/z*: 238.07 (100), 240.08 (92) ([M]$^+$, calcd. for $C_{12}H_{15}Br^+$: 238.04 (100), 240.03 (98)). The analytical data are in agreement with the literature data.[4]

(*Z*)-1-(2-Bromovinyl)-4-(*tert*-butyl)benzene ((*Z*)-8)

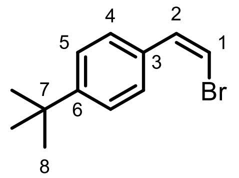

Prepared according to General Procedure C, 4-(*tert*-butyl)benzaldehyde (162 mg, 1.00 mmol, 1.0 eq.) yielded 1-(*tert*-butyl)-4-(2,2-dibromovinyl)benzene (240 mg, 0.75 mmol, 75%), of which 159 mg, 0.50 mmol, 1.0 eq. was converted to **(*Z*)-8**, a colourless liquid (43 mg, 0.18 mmol, 36%).

R_f = 0.52 (*n*-pentane); **^{1}H NMR** (599 MHz, $CDCl_3$): δ = 7.69-7.64 (m, 2H, H4), 7.43-7.40 (m, 2H, H5), 7.04 (d, $^3J_{HH}$ = 8.1 Hz, 1H, H2), 6.38 (d, $^3J_{HH}$ = 8.1 Hz, 1H, H1), 1.34 (s, 9H, H8) ppm; **^{13}C{^{1}H} NMR** (151 MHz, $CDCl_3$): δ = 151.6 (C6), 132.23 (C3), 132.21 (C2), 128.9 (C4), 125.3 (C5), 105.6 (C1), 34.9 (C7), 31.4 (C8) ppm; **IR (ATR):** ν = 2962 (m), 2905 (w), 2868 (w), 1609 (w), 1510 (m), 1462 (m), 1407 (w), 1363 (m), 1318 (s), 1269 (m), 1202 (w), 1125 (w), 1107 (m), 1017 (w), 852 (s), 839 (s), 818 (m), 753 (w), 720 (s), 691 (m) cm^{-1}; **GC-EI-MS:** *m/z*: 238.05 (100), 240.05 (96) ([M]$^+$, calcd. for $C_{12}H_{15}Br^+$: 238.04 (100), 240.03 (98)). The analytical data are in agreement with the literature data.[11]

(*Z*,*E*)-4-(2-Bromovinyl)-1,1'-biphenyl ((*Z*,*E*)-9)

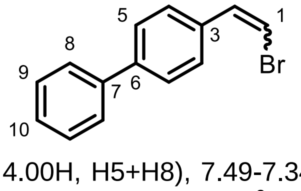

Prepared according to General Procedure A, 4-ethynyl-1,1'-biphenyl (**A-9**) (36 mg, 0.20 mmol, 1.0 eq.) was converted to an isomeric mixture of **(*Z*,*E*)-9**, a colourless liquid (1st experiment: 39 mg, 0.15 mmol, 75%, *Z*:*E* = 80:20; 2nd experiment: 37 mg, 0.14 mmol, 71% *Z*:*E* = 80:20, average: 73%, *Z*:*E* = 80:20).

^{1}H NMR (400 MHz, $CDCl_3$): δ = 7.83-7.77 (m, 1.60H, $H4_Z$), 7.66-7.55 (m, 4.00H, H5+H8), 7.49-7.34 (m, 3.20H, $H4_E$+H9+H10), 7.19-7.09 (m, 1H, H2), 6.82 (d, $^3J_{HH}$ = 14.0 Hz, 0.20H, $H1_E$), 6.47 (d, $^3J_{HH}$ = 8.1 Hz, 0.80H, $H1_Z$) ppm.

(*E*)-4-(2-Bromovinyl)-1,1'-biphenyl ((*E*)-9)

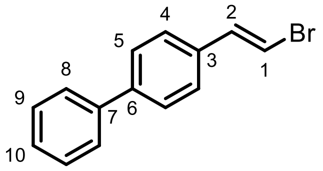

Prepared according to General Procedure B, 4-ethynyl-1,1'-biphenyl (**A-9**) (36 mg, 0.20 mmol, 1.0 eq.) was converted to **(*E*)-9**, a colourless solid (39 mg, 0.15 mmol, 76%).

R$_f$ = 0.36 (*n*-pentane); **M.p.:** 124 °C; **^{1}H NMR** (400 MHz, $CDCl_3$): δ = 7.65-7.56 (m, 4H, H8+H5), 7.50-7.43 (m, 2H, H9), 7.42-7.35 (m, 3H, H4+H10), 7.17 (d, $^3J_{HH}$ = 14.0 Hz, 1H, H2), 6.84 (d, $^3J_{HH}$ = 14.0 Hz, 1H, H1) ppm; **^{13}C{^{1}H} NMR** (126 MHz, $CDCl_3$): δ = 141.2 (C6), 140.6 (C7), 136.9 (C2), 135.1 (C3), 129.0 (C9), 127.7 (C19), 127.6 (C5), 127.1 (C8), 126.7 (C4), 106.7 (C1) ppm; **IR (ATR):** ν = 3064 (w), 3032 (w), 2921 (w), 1595 (w), 1580 (w), 1554 (w), 1483 (w), 1449 (w), 1408 (w), 1281 (w), 1226 (w), 1207 (w), 1186 (w), 1078 (w), 1038 (w), 1005 (w), 993 (w), 957 (m), 937 (s), 917 (w), 844 (m), 791 (w), 754 (s), 738 (m), 707 (m) cm^{-1}; **GC-EI-MS:** *m/z*: 258.06 (99), 260.06 (100) ([M]$^+$, calcd. for $C_{14}H_{11}Br^+$: 258.00 (100), 260.00 (98)). The analytical data are in agreement with the literature data.[4]

(*Z*)-4-(2-Bromovinyl)-1,1'-biphenyl ((*Z*)-9)

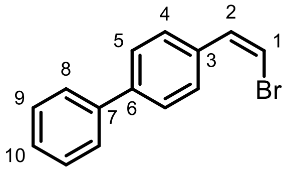

Prepared according to General Procedure C, [1,1'-biphenyl]-4-carbaldehyde (183 mg, 1.00 mmol, 1.0 eq.) yielded 4-(2,2-dibromovinyl)-1,1'-biphenyl (157 mg, 0.47 mmol, 47%), of which 67 mg, 0.20 mmol, 1.0 eq. was converted with $Pd(PPh_3)_4$ (11 mg, 0.01 mmol, 0.05 eq.) and $SnBu_3H$ (0.06 mL) in CH_2Cl_2 (0.4 mL) to **(*Z*)-9**, a colourless solid (42 mg, 0.16 mmol, 81%).

R$_f$ = 0.36 (*n*-pentane); **M.p.:** 51 °C; **^{1}H NMR** (500 MHz, $CDCl_3$): δ = 7.83-7.77 (m, 2H, H4), 7.66-7.60 (m, 4H, H5+H8), 7.50-7.42 (m, 2H, H9), 7.39-7.35 (m, 1H, H10), 7.12 (d, $^3J_{HH}$ = 8.2 Hz, 1H, H2), 6.47 (d, $^3J_{HH}$ = 8.1 Hz, 1H, H1) ppm; **^{13}C{^{1}H} NMR** (126 MHz, $CDCl_3$): δ = 141.2 (C6), 140.7 (C7), 134.0 (C3), 132.1 (C2), 129.6 (C4), 129.0 (C9), 127.7 (C10), 127.2 (C8), 127.0 (C5), 106.5 (C1) ppm; **IR (ATR):** ν = 1604 (w), 1483 (m), 1449 (w), 1404 (m), 1332 (m), 1318 (m), 1168 (w), 1128 (w), 1039 (w), 1004 (m), 84766 (s), 740 (m), 731 (m), 680 (s) cm^{-1}; **GC-EI-MS:** *m/z*: 258.05 (98), 260.05 (100) ([M]$^+$, calcd. for $C_{14}H_{11}Br^+$: 258.00 (100), 260.00 (98)).

(*Z*,*E*)-1-(2-Bromovinyl)-4-methylbenzene ((*Z*,*E*)-10)

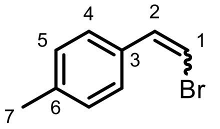

Prepared according to General Procedure A, 1-ethynyl-4-methylbenzene (**A-10**) (23 mg, 0.20 mmol, 1.0 eq.) was converted to an isomeric mixture of **(*Z*,*E*)-8**, a colourless liquid (1st experiment: 23 mg, 0.12 mmol, 58%, *Z*:*E* = 79:21; 2nd experiment: 23 mg, 0.12 mmol, 58% *Z*:*E* = 79:21, average: 58%, *Z*:*E* = 79:21).

^{1}H NMR (400 MHz, $CDCl_3$): δ = 7.60 (d, $^3J_{HH}$ = 8.3 Hz, 1.58H, H4$_Z$), 7.24-7.02 (m, 3.42H, H2+H4$_E$+H5), 6.71 (d, $^3J_{HH}$ = 13.9 Hz, 0.21H, H1$_E$), 6.38 (d, $^3J_{HH}$ = 8.0 Hz, 1H, H1$_Z$), 2.36 (s, 2.37H, H7$_Z$), 2.34 (s, 0.63H, H7$_E$) ppm.

(*E*)-1-(2-Bromovinyl)-4-methylbenzene ((*E*)-10)

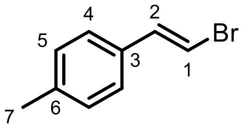

Prepared according to a procedure by Rueping *et al.*[12] 1-(2,2-dibromovinyl)-4-methylbenzene (276 mg, 1.00 mmol, 1.0 eq.) and diethylphosphite (0.39 mL, 3.00 mmol, 3.0 eq.) were dissolved in DMF (0.5 mL) and cooled to 0 °C. At 0 °C, NEt_3 (0.42 mL, 3.00 mmol, 3.0 eq.) was added and the reaction mixture was stirred for 18 h while warming to room temperature. Water (5 mL) was added and the crude product was extracted with CH_2Cl_2 (3 x 5 mL). The separated organic layer was washed with water (10 mL), dried over $MgSO_4$, filtered and concentrated under reduced pressure. Purification by column chromatography (SiO_2, *n*-pentane) afforded **(*E*)-10**, a colourless solid (24 mg, 0.12 mmol, 12%). **R$_f$** = 0.56 (*n*-hexane); **M.p.:** 44 °C; **^{1}H NMR** (400 MHz, $CDCl_3$): δ = 7.20 (d, $^3J_{HH}$ = 8.1 Hz, 2H, H4), 7.14 (d, $^3J_{HH}$ = 8.1 Hz, 2H, H5), 7.08 (d, $^3J_{HH}$ = 14.0 Hz, 1H, H2), 6.71 (d, $^3J_{HH}$ = 14.0 Hz, 1H, H1), 2.34 (s, 3H, H7) ppm; **^{13}C{^{1}H} NMR** (101 MHz, $CDCl_3$): δ = 138.4 (C6), 137.2 (C2), 133.3 (C3), 129.6 (C5), 126.1 (C4), 105.6 (C1), 21.4 (C7) ppm; **IR (ATR):** ν = 3069 (w), 3027 (w), 2914 (w), 2854 (w), 1602 (w), 1567 (w), 1543 (w), 1509 (m), 1447 (w), 1410 (w), 1374 (w), 1324 (w), 1300 (w), 1278 (w), 1226 (w), 1195 (w), 1180 (w), 1121 (w), 1108 (w), 1040 (w), 1020 (w), 964 (w), 949 (m), 937 (s), 826 (s), 769 (s), 724 (s) cm^{-1}; **GC-EI-MS:** *m/z*: 195.94 (100), 197.99 (88) ([M]$^+$, calcd. for $C_9H_9Br^+$: 195.99 (100), 197.99 (98)). The analytical data are in agreement with the literature data.[4]

(*Z*)-1-(2-Bromovinyl)-4-methylbenzene ((*Z*)-10)

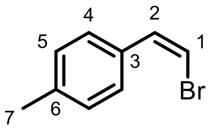

Prepared according the second part of General Procedure C, 1-(2,2-dibromovinyl)-4-methylbenzene (138 mg, 0.50 mmol, 1.0 eq.) was converted to **(*Z*)-10**, a colourless liquid (49 mg, 0.25 mmol, 50%).

R$_f$ = 0.56 (*n*-hexane); **^{1}H NMR** (500 MHz, $CDCl_3$): δ = 7.62-7.57 (m, 2H, H4), 7.22-7.16 (m, 2H, H5), 7.04 (d, $^3J_{HH}$ = 8.1 Hz, 1H, H2), 6.38 (d, $^3J_{HH}$ = 8.1 Hz, 1H, H1),

2.36 (s, 3H, H7) ppm; **^{13}C{^{1}H} NMR** (126 MHz, $CDCl_3$): δ = 138.5 (C6), 132.3 (C2), 132.2 (C3), 129.07 (C4+C5), 105.6 (C1), 21.5 (C7) ppm; **IR (ATR):** ν = 2920 (w), 1605 (w), 1509 (m), 1449 (w), 1410 (w), 1325 (m), 1316 (s), 1295 (w), 1186 (w), 1166 (w), 1118 (w), 1039 (w), 1021 (w), 946 (w), 843 (m), 819 (s), 768 (m), 725 (m), 685 (s) cm^{-1}; **GC-EI-MS:** *m/z*: 196.03 (100), 198.03 (98) ($[M]^+$, calcd. for $C_9H_9Br^+$: 195.99 (100), 197.99 (98)). The analytical data are in agreement with the literature data.[2]

(*Z*,*E*)-1-(2-Bromovinyl)-2-methylbenzene ((*Z*,*E*)-11)

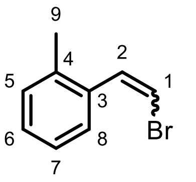

Prepared according to General Procedure A, 1-ethynyl-2-methylbenzene (**A-11**) (23 mg, 0.20 mmol, 1.0 eq.) was converted to an isomeric mixture of **(*Z*,*E*)-11**, a colourless liquid (1st experiment: 14 mg, 0.07 mmol, 36%, *Z*:*E* = 67:33; 2nd experiment: 15 mg, 0.08 mmol, 38% *Z*:*E* = 69:31, average: 37%, *Z*:*E* = 68:32).
^{1}H NMR (400 MHz, $CDCl_3$): δ = 7.59-7.54 (m, 0.69H, $H8_Z$), 7.35-7.13 (m, 3.31H, H5+H6+H7+$H8_E$), 6.64 (d, $^3J_{HH}$ = 13.9 Hz, 0.31H, $H1_E$), 6.53 (d, $^3J_{HH}$ = 7.9 Hz, 0.69H, $H1_Z$), 2.34 (s, 0.93H, $H9_E$), 2.28 (s, 2.07H, $H9_Z$) ppm.

(*E*)-1-(2-Bromovinyl)-2-methylbenzene ((*E*)-11)

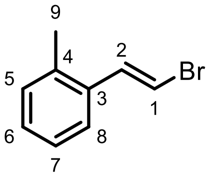

Prepared according to General Procedure B, 1-ethynyl-2-methylbenzene (**A-11**) (23 mg, 0.20 mmol, 1.0 eq.) was converted to **(*E*)-11**, a colourless liquid (25 mg, 0.13 mmol, 63%).
R_f = 0.67 (*n*-pentane); **^{1}H NMR** (500 MHz, $CDCl_3$): δ = 7.34-7.30 (m, 2H, H2+H6), 7.23-7.19 (m, 1H, H8), 7.19-7.15 (m, 2H, H5+H7), 6.64 (d, $^3J_{HH}$ = 13.8 Hz, 2H, H1), 2.34 (s, 3H, H9) ppm; **^{13}C{^{1}H} NMR** (126 MHz, $CDCl_3$): δ = 135.7 (C2), 135.33 (C4), 135.27 (C3), 130.6 (C5), 128.4 (C8), 126.4 (C7), 126.0 (C6), 107.4 (C1), 20.0 (C9) ppm; **IR (ATR):** ν = 3071 (w), 1609 (m), 1595 (m), 1569 (w), 1482 (m), 1459 (m), 1380 (w), 1280 (w), 1225 (m), 1206 (m), 1182 (w), 1160 (w), 1102 (m), 1034 (m), 944 (m), 932 (s), 866 (w), 797 (m), 740 (s), 717 (s) cm^{-1}; **GC-EI-MS:** *m/z*: 196.01 (100), 198.02 (94) ($[M]^+$, calcd. for $C_9H_9Br^+$: 195.99 (100), 197.99 (98)). The analytical data are in agreement with the literature data.[4]

(*Z*)-1-(2-Bromovinyl)-2-methylbenzene ((*Z*)-11)

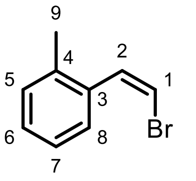

Prepared according to General Procedure C, 2-methylbenzaldehyde (0.12 mL, 1.00 mmol, 1.0 eq.) yielded 1-(2,2-dibromovinyl)-2-methylbenzene (202 mg, 0.74 mmol, 74%), of which 138 mg, 0.50 mmol, 1.0 eq. was converted to **(*Z*)-11**, a colourless liquid (15 mg, 0.08 mmol, 38%).
R_f = 0.67 (*n*-pentane); **^{1}H NMR** (599 MHz, $CDCl_3$): δ = 7.59-7.55 (m, 1H, H8), 7.26-7.20 (m, 3H, H5+H6+H7), 7.16 (d, $^3J_{HH}$ = 7.9 Hz, 1H, H2), 6.54 (d, $^3J_{HH}$ = 7.9 Hz, 1H, H1), 2.29 (s, 3H, H9) ppm;**^{13}C{^{1}H} NMR** (151 MHz, $CDCl_3$): δ = 136.3 (C4), 134.5 (C3), 132.2 (C2), 130.1 (C5), 128.8 (C8), 128.3 (C6), 125.6 (C7), 108.6 (C1), 19.9 (C9) ppm; **IR (ATR):** ν = 1616 (w), 1481 (m), 1458 (m), 1379 (w), 1314 (m), 1288 (w), 1159 (w), 1105 (w), 1047 (w), 1034 (w), 944 (w), 868 (w), 837 (m), 796 (m), 755 (s), 727 (s), 691 (s), 669 (s); **GC-EI-MS:** *m/z*: 196.02 (100), 198.01 (96) ($[M]^+$, calcd. for $C_9H_9Br^+$: 195.99 (100), 197.99 (98)). The analytical data are in agreement with the literature data.[8]

(*Z*,*E*)-1-(2-Bromovinyl)-3-methylbenzene ((*Z*,*E*)-12)

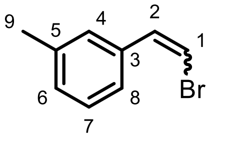

Prepared according to General Procedure A, 1-ethynyl-3-methylbenzene (**A-12**) (23 mg, 0.20 mmol, 1.0 eq.) was converted to an isomeric mixture of **(*Z*,*E*)-12**, a colourless liquid (1st experiment: 28 mg, 0.14 mmol, 72%, *Z*:*E* = 75:25; 2nd experiment: 27 mg, 0.14 mmol, 69% *Z*:*E* = 75:25, average: 71%, *Z*:*E* = 75:25).
^{1}H NMR (400 MHz, $CDCl_3$): δ = 7.54-7.43 (m, 1.5H, $H8_Z$+$H4_Z$), 7.29-6.99 (m, 3.5H, H2+$H4_E$+H6+H7+$H8_E$), 6.74 (d, $^3J_{HH}$ = 13.9 Hz, 0.25H, $H1_E$), 6.40 (d, $^3J_{HH}$ = 8.1 Hz, 0.75H, $H1_Z$), 2.37 (s, 2.25H, $H9_Z$), 2.33 (s, 0.75H, $H9_E$) ppm.

(*E*)-1-(2-Bromovinyl)-3-methylbenzene ((*E*)-12)

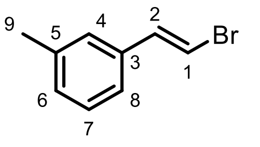

Prepared according to General Procedure B, 1-ethynyl-3-methylbenzene (**A-12**) (23 mg, 0.20 mmol, 1.0 eq.) was converted to **(*E*)-12**, a colourless liquid (27 mg, 0.14 mmol, 68%).
R_f = 0.67 (*n*-pentane); **^{1}H NMR** (599 MHz, $CDCl_3$): δ = 7.23-7.19 (m, 1H, H7), 7.12-7.09 (m, 3H, H4+H6+H8), 7.08 (d, $^3J_{HH}$ = 14.0 Hz, 1H, H2), 6.75 (d, $^3J_{HH}$ = 14.0 Hz, 1H, H1), 2.35 (s, 3H, H9) ppm; **^{13}C{^{1}H} NMR** (151 MHz, $CDCl_3$): δ = 138.6 (C5), 137.4 (C2), 136.0 (C3), 129.2 (C6), 128.8 (C7), 127.0 (C4), 123.4 (C8), 106.4 (C1), 21.5 (C9) ppm; **IR (ATR):** ν = 1613 (w), 1602 (w), 1255 (w), 1198 (w), 937 (s), 788 (w), 757 (s), 718 (m), 687 (s) cm^{-1}; **GC-EI-MS:** *m/z*: 196.02 (100), 199.05 (98) ($[M]^+$, calcd. for $C_9H_9Br^+$: 195.99 (100), 197.99 (98)). The analytical data are in agreement with the literature data.[4]

(*Z*)-1-(2-Bromovinyl)-3-methylbenzene ((*Z*)-12)

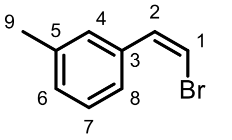

Prepared according to General Procedure C, 3-methylbenzaldehyde (120 mg, 1.00 mmol, 1.0 eq.) yielded 1-(2,2-dibromovinyl)-3-methylbenzene (200 mg, 0.73 mmol, 73%), of which 138 mg, 0.50 mmol, 1.0 eq. was converted to **(*Z*)-12**, a colourless liquid (28 mg, 0.14 mmol, 29%).

R_f = 0.67 (*n*-pentane); **^{1}H NMR** (599 MHz, $CDCl_3$): δ = 7.50 (d, $^3J_{HH}$ = 7.7 Hz, 1H, H8), 7.46 (s, 1H, H4), 7.28-7.23 (m, 1H, H7), 7.15-7.11 (m, 1H, H6), 7.03 (d, $^3J_{HH}$ = 8.1 Hz, 1H, H2), 6.40 (d, $^3J_{HH}$ = 8.1 Hz, 1H, H1), 2.37 (s, 3H) ppm; **^{13}C{^{1}H} NMR** (151 MHz, $CDCl_3$): δ = 138.0 (C5), 135.0 (C3), 132.6 (C2), 129.8 (C4), 129.2 (C6), 128.3 (C7), 126.2 (C8), 106.3 (C1), 21.6 (C9) ppm; **IR (ATR):** ν = 1618 (w), 1602 (m), 1583 (w), 1482 (m), 1455 (w), 1412 (w), 1322 (m), 1143 (w), 1094 (w), 1040 (w), 924 (m), 905 (w), 880 (m), 794 (s), 771 (m), 753 (m), 714 (m), 676 (s) cm^{-1}; **GC-EI-MS:** *m/z*: 196.01 (100), 198.03 (98) ($[M]^+$, calcd. for $C_9H_9Br^+$: 195.99 (100), 197.99 (98)).

(*Z*,*E*)-2-(2-Bromovinyl)naphthalene ((*Z*,*E*)-13)

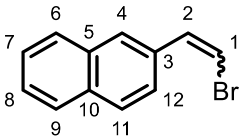

Prepared according to General Procedure A, 2-ethynylnaphthalene (**A-13**) (30 mg, 0.20 mmol, 1.0 eq.) was converted to an isomeric mixture of **(*Z*,*E*)-13**, a colourless solid (1st experiment: 41 mg, 0.18 mmol, 89%, *Z*:*E* = 77:23; 2nd experiment: 42 mg, 0.18 mmol, 90% *Z*:*E* = 77:23, average: 90%, *Z*:*E* = 77:23).

^{1}H NMR (400 MHz, $CDCl_3$): δ = 8.16 (s, 0.77H, $H4_Z$), 7.88-7.77 (m, 3.77H, $H6_E$ +$H7_E$ +$H11_E$+$4H_{Ar,\,Z}$), 7.69 (s, 0.23H, $H4_E$), 7.53-7.45 (m, 2.23H, $H9_E$ +$H8_E$ +$H12_E$+$2H_{Ar,\,Z}$), 7.29-7.22 (m, 1H, H2), 6.90 (d, $^3J_{HH}$ = 14.0 Hz, 0.23H, $H1_Z$), 6.52 (d, $^3J_{HH}$ = 8.1 Hz, 0.77H, $H1_Z$) ppm.

(*E*)-2-(2-Bromovinyl)naphthalene ((*E*)-13)

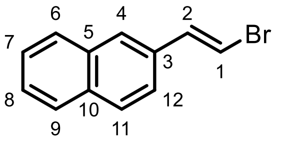

Prepared according to General Procedure B, 2-ethynylnaphthale (**A-13**) (30 mg, 0.20 mmol, 1.0 eq.) was converted to **(*E*)-15**, a colourless solid (32 mg, 0.14 mmol, 69%).

R_f = 0.58 (*n*-pentane); **M.p.:** 87 °C; **^{1}H NMR** (400 MHz, $CDCl_3$): δ = 7.86-7.76 (m, 3H, H6+H7+H11), 7.68 (s, 1H, H4), 7.53-7.43 (m, 3H,H9+H8+H12), 7.27 (d, $^3J_{HH}$ = 14.0 Hz, 1H, H2), 6.90 (d, $^3J_{HH}$ = 14.0 Hz, 1H, H1) ppm; **^{13}C{^{1}H} NMR** (151 MHz, $CDCl_3$): δ = 137.4 (C2), 133.6 (C5), 133.5 (C3), 133.2 (C10), 128.7 (C11), 128.2 (C6), 127.9 (C7), 126.7 (C9), 126.5 (C8), 126.4 (C4), 123.0 (C12), 107.0 (C1) ppm; **IR (ATR):** ν = 3076 (m), 3059 (m), 1725 (w), 1687 (w), 1624 (w), 1611 (m), 1594 (m), 1568 (w), 1507 (m), 1465 (w), 1435 (m), 1364 (w), 1293 (w), 1273 (m), 1249 (w), 1218 (m), 1202 (m), 1176 (w), 1158 (w), 1145 (w), 1125 (m), 1018 (w), 966 (w), 943 (s), 893 (m), 862 (m), 822 (s), 780 (s), 762 (s), 746 (s), 705 (m) cm^{-1}; **GC-EI-MS:** *m/z*: 232.03 (100), 234.01 (92) ($[M]^+$, calcd. for $C_{12}H_9Br^+$: 231.99 (100), 233.99 (98)). The analytical data are in agreement with the literature data.[13]

(*Z*)-2-(2-Bromovinyl)naphthalene ((*Z*)-13)

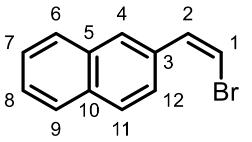

Prepared according to General Procedure C, 2-naphthaldehyde (156 mg, 1.00 mmol, 1.0 eq.) yielded 2-(2,2-dibromovinyl)naphthalene (240 mg, 0.77 mmol, 77%), of which 156 mg, 0.50 mmol, 1.0 eq. was converted to **(*Z*)-13**, a colourless liquid (35 mg, 0.15 mmol, 76%).

R_f = 0.58 (*n*-pentane); **M.p.:** 79 °C; **^{1}H NMR** (500 MHz, $CDCl_3$): δ = 8.16 (s, 1H, H4), 7.88-7.80 (m, 4H, H_{Ar}), 7.53-7.47 (m, 2H, H_{Ar}), 7.24 (d, $^3J_{HH}$ = 8.1 Hz, 1H, H2), 6.52 (d, $^3J_{HH}$ = 8.1 Hz, 1H, H1) ppm; **^{13}C{^{1}H} NMR** (126 MHz, $CDCl_3$): δ = 133.21 (C5), 133.17 (C10), 132.58 (C3), 132.56 (C2), 128.7 (C4), 128.4 (C_{Ar}), 127.9 (C_{Ar}), 127.8 (C_{Ar}), 126.58 (C_{Ar}), 126.56 (C_{Ar}), 126.4 (C_{Ar}), 106.8 (C1) ppm; **IR (ATR):** ν = 3069 (w), 1591 (w), 1508 (w), 1464 (w), 1371 (w), 1358 (w), 1320 (s), 1275 (m), 1231 (w), 1162 (w), 1146 (w), 1121 (w), 1016 (w), 971 (w), 951 (w), 930 (w), 899 (m), 885 (w), 863 (s), 822 (s), 780 (m), 766 (m), 751 (s), 712 (s) cm^{-1}; **GC-EI-MS:** *m/z*: 232.04 (100), 234.09 (96) ($[M]^+$, calcd. for $C_{12}H_9Br^+$: 231.99 (100), 233.99 (98)). The analytical data are in agreement with the literature data.[2]

(*Z*,*E*)-1-(2-Bromovinyl)-4-(trifluoromethyl)benzene ((*Z*,*E*)-14)

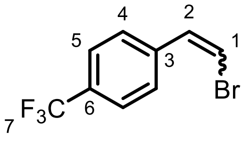

Prepared according to General Procedure A, 1-ethynyl-4-(trifluoromethyl)benzene (**A-14**) (34 mg, 0.20 mmol, 1.0 eq.) was converted to an isomeric mixture of **(*Z*,*E*)-14**, a colourless liquid (1st experiment: 15 mg, 0.06 mmol, 30%, *Z*:*E* = 48:52; 2nd experiment: 16 mg, 0.06 mmol, 32% *Z*:*E* = 48:52, average: 31%, *Z*:*E* = 48:52).

^{1}H NMR (400 MHz, $CDCl_3$): δ = 7.77 (d, $^3J_{HH}$ = 8.8 Hz, 0.96H, $H4_Z$), 7.67-7.54 (m, 2H, H5), 7.40 (d, $^3J_{HH}$ = 8.1 Hz, 1.04H, $H4_E$), 7.19-7.08 (m, 1H, H2), 6.91 (d, $^3J_{HH}$ = 14.0 Hz, 0.52H, $H1_E$), 6.58 (d, $^3J_{HH}$ = 8.3 Hz, 0.48H, $H1_Z$) ppm.

(*E*)-1-(2-Bromovinyl)-4-(trifluoromethyl)benzene ((*E*)-14)

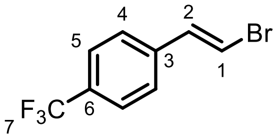

Prepared according to General Procedure B, 1-ethynyl-4-(trifluoromethyl)benzene (**A-14**) (34 mg, 0.20 mmol, 1.0 eq.) was converted to **(*E*)-14**, a colourless liquid (15 mg, 0.06 mmol, 30%).

R$_f$ = 0.50 (*n*-hexane); **^{1}H NMR** (500 MHz, $CDCl_3$): δ = 7.60-7.56 (m, 2H, H5), 7.42-7.38 (m, 2H, H4), 7.14 (d, $^3J_{HH}$ = 14.0 Hz, 1H, H2), 6.91 (d, $^3J_{HH}$ = 14.0 Hz, 1H, H1) ppm; **^{13}C{^{1}H} NMR** (126 MHz, $CDCl_3$): δ = 139.4 (q, $^5J_{CF}$ = 1.4 Hz, C3), 136.1 (q, $^6J_{CF}$ = 0.7 Hz, C2), 130.3 (q, $^2J_{CF}$ = 32.7 Hz, C6), 126.4 (C4), 126.0 (q, $^3J_{CF}$ = 3.8 Hz, C5), 124.2 (q, $^1J_{CF}$ = 272.0 Hz, C7), 109.5 (C1) ppm; **^{19}F NMR** (470 MHz, $CDCl_3$): δ = -62.72 ppm; **IR (ATR):** ν = 1616 (w), 1605 (w), 1574 (w), 1411 (w), 1321 (s), 1286 (w), 1163 (m), 1119 (s), 1106 (s), 1066 (s), 1017 (m), 957 (w), 933 (s), 845 (m), 784 (m), 738 (s), 723 (m); **GC-EI-MS:** *m/z*: 249.97 (100), 251.99 (94) ([M]$^+$, calcd. for $C_9H_6F_3Br^+$: 249.96 (100), 251.96 (98)). The analytical data are in agreement with the literature data.[9]

(*Z*)-1-(2-Bromovinyl)-4-(trifluoromethyl)benzene ((*Z*)-14)

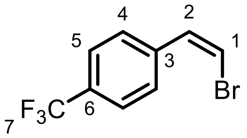

Prepared according to the second part of General Procedure C, 1-(2,2-dibromovinyl)-4-(trifluoromethyl)benzene (165 mg, 0.50 mmol, 1.0 eq.) was converted to **(*Z*)-14**, a colourless liquid (52 mg, 0.21 mmol, 41%).

R$_f$ = 0.50 (*n*-hexane); **^{1}H NMR** (500 MHz, $CDCl_3$): δ = 7.80-7.75 (m, 2H, H4), 7.65-7.61 (m, 2H, H5), 7.12 (d, $^3J_{HH}$ = 8.2 Hz, 1H, H2), 6.58 (d, $^3J_{HH}$ = 8.2 Hz, 1H, H1) ppm; **^{13}C{^{1}H} NMR** (126 MHz, $CDCl_3$): δ = 138.5 (q, $^5J_{CF}$ = 1.3 Hz), 131.4 (C2), 130.2 (q, $^2J_{CF}$ = 32.7 Hz), 129.3 (C4), 125.3 (q, $^3J_{CF}$ = 3.8 Hz, C5), 124.2 (q, $^1J_{CF}$ = 272.0 Hz, C7), 109.1 (C1) ppm; **^{19}F{^{1}H} NMR** (376 MHz, $CDCl_3$): δ = -62.72 ppm; **IR (ATR):** ν = 1620 (w), 1406 (w), 1322 (s), 1163 (m), 1111 (s), 1066 (s), 1016 (m), 851 (s), 826 (m), 766 (w), 749 (w), 705 (m) cm^{-1}; **GC-EI-MS:** *m/z*: 249.97 (100), 251.99 (99) ([M]$^+$, calcd. for $C_9H_6F_3Br^+$: 249.96 (100), 251.96 (98)). The analytical data are in agreement with the literature data.[9]

(*Z*,*E*)-1-(2-Bromovinyl)-4-methoxybenzene ((*Z*,*E*)-15)

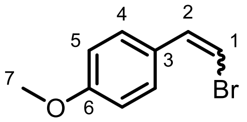

Prepared according to General Procedure A, 1-ethynyl-4-methoxybenzene (**A-15**) (27 mg, 0.20 mmol, 1.0 eq.) was converted to an isomeric mixture of **(*Z*,*E*)-7**, a colourless liquid (1st experiment: 29 mg, 0.14 mmol, 68%, *Z*:*E* = 86:14; 2nd experiment: 28 mg, 0.13 mmol, 67% *Z*:*E* = 86:14, average: 68%, *Z*:*E* = 86:14).

^{1}H NMR (400 MHz, $CDCl_3$): δ = 7.72-7.63 (m, 1.72H, H4$_Z$), 7.25-7.21 (m, 0.28H, H4$_E$), 7.07-6.98 (m, 1H, H2), 6.95-6.89 (m, 1.72H, H5$_Z$), 6.88-6.82 (m, 0.28H, H5$_E$), 6.61 (d, $^3J_{HH}$ = 13.9 Hz, 0.14H, H1$_E$), 6.31 (d, $^3J_{HH}$ = 8.1 Hz, 0.86H, H1$_Z$), 3.83 (s, 2.58H, H7$_Z$), 3.81 (s, 0.42H, H7$_E$) ppm.

(*E*)-1-(2-Bromovinyl)-4-methoxybenzene ((*E*)-15)

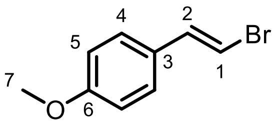

Prepared according to General Procedure B, 1-ethynyl-4-methoxybenzene (**A-15**) (26 mg, 0.20 mmol, 1.0 eq.) was converted to **(*E*)-15**, a colourless solid (25 mg, 0.12 mmol, 59%).

R$_f$ = 0.17 (*n*-hexane); **M.p.:** 47 °C; **^{1}H NMR** (599 MHz, $CDCl_3$): δ = 7.25-7.21 (m, 2H, H4), 7.04 (d, $^3J_{HH}$ = 14.0 Hz, 1H, H2), 6.87-6.84 (m, 2H, H5), 6.61 (d, $^3J_{HH}$ = 14.0 Hz, 1H, H1), 3.81 (s, 3H, H7) ppm; **^{13}C{^{1}H} NMR** (151 MHz, $CDCl_3$): δ = 159.8 (C6), 136.7 (C2), 128.9 (C3), 127.5 (C4), 114.3 (C5), 104.1 (C1), 55.4 (C7) ppm; **IR (ATR):** ν = 3068 (w), 3032 (w), 2958 (w), 2932 (w), 2837 (w), 1603 (m), 1569 (w), 1509 (m), 1457 (m), 1440 (m), 1417 (w), 1322 (w), 1303 (w), 1285 (w), 1252 (m), 1222 (m), 1191 (m), 1175 (m), 1110 (m), 1027 (m), 950 (s), 928 (m), 834 (s), 775 (s), 712 (m) cm^{-1}; **GC-EI-MS:** *m/z*: 212.02 (98), 214.03 (100) ([M]$^+$, calcd. for $C_9H_9OBr^+$: 211.98 (100), 213.98 (98)). The analytical data are in agreement with the literature data.[14]

(*Z*)-1-(2-Bromovinyl)-4-methoxybenzene ((*Z*)-15)

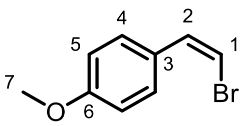

Prepared according to a procedure by Wu *et al.*[11] (*E*)-3-(4-methoxyphenyl)acrylic acid (891 mg, 5.0 mmol, eq.) was dissolved in CH_2Cl_2 (25 mL) and cooled to 0 °C. At 0 °C, Br_2 (0.31 mL, 6.0 mmol, 1.2 eq.) was added dropwise and the resulting dark orange mixture was stirred at room temperature for 2 h. The solvent and excess of Br_2 were removed by distillation and the lactone (a white solid) was redissolved in THF (25 mL) and cooled to 0 °C. At 0 °C, DBU (1.5 mL, 10.0 mmol, 2.0 eq.) was added and the reaction mixture was stirred for 18 h at room temperature. *n*-Hexane (20 mL) and water (20 mL) were added and the separated aqueous layer was extracted with Et_2O:*n*-hexane (10:90) (3 x 20 mL). The combined organic layers were dried over $MgSO_4$, filtered and

concentrated under reduced pressure. Column chromatography (SiO_2, Et_2O:*n*-pentane 2:98) afforded **(*Z*)-15**, a colourless liquid (901 mg, 4.3 mmol, 85%).

R$_f$ = 0.17 (*n*-hexane); **^{1}H NMR** (599 MHz, $CDCl_3$): δ = 7.71-7.66 (m, 2H, H4), 7.00 (d, $^3J_{HH}$ = 8.1 Hz, 1H, H2), 6.94-6.90 (m, 2H, H5), 6.32 (d, $^3J_{HH}$ = 8.1 Hz, 1H, H1), 3.83 (s, 3H, H7) ppm; **^{13}C{^{1}H} NMR** (151 MHz, $CDCl_3$): δ = 159.6 (C6), 131.8 (C2), 130.6 (C4), 127.7 (C3), 113.7 (C5), 104.3 (C1), 55.4 (C7) ppm; **IR (ATR):** ν = 2836 (w), 1603 (m), 1573 (w), 1507 (s), 1456 (w), 1441 (w), 1418 (w), 1327 (w), 1317 (w), 1303 (m), 1250 (s), 1178 (m), 1166 (m), 1116 (w), 1030 (s), 940 (w), 831 (s), 769 (w), 735 (w), 686 (m), 659 (w); **GC-EI-MS:** *m/z*: 212.05 (100), 214.05 (98) ([M]$^+$, calcd. for $C_9H_9OBr^+$: 211.98 (100), 213.98 (98)). The analytical data are in agreement with the literature data.[2]

(*Z*,*E*)-5-(2-Bromovinyl)-1,2,3-trimethoxybenzene ((*Z*,*E*)-16)

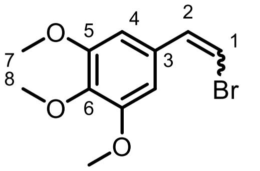

Prepared according to General Procedure A, 5-ethynyl-1,2,3-trimethoxybenzene (**A-16**) (38 mg, 0.20 mmol, 1.0 eq.) was converted to an isomeric mixture of **(*Z*,*E*)-16**, a yellow liquid (1st experiment: 47 mg, 0.17 mmol, 86%, *Z*:*E* = 81:19; 2nd experiment: 46 mg, 0.17 mmol, 84% *Z*:*E* = 81:19, average: 85%, *Z*:*E* = 81:19).

^{1}H NMR (400 MHz, $CDCl_3$): δ = 7.05-6.96 (m, 1.81H, H2+H4$_Z$), 6.69 (d, $^3J_{HH}$ = 14.0 Hz, 0.19H, H1$_Z$), 6.51 (s, 0.19H, H4$_E$), 6.39 (d, $^3J_{HH}$ = 8.0 Hz, 0.81H, H1$_Z$), 3.89-3.84 (m, 9H, H7+H8) ppm.

(*E*)-5-(2-Bromovinyl)-1,2,3-trimethoxybenzene ((*E*)-16)

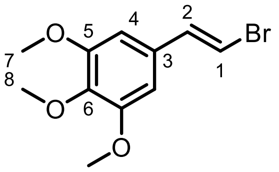

Prepared according to General Procedure B, 5-ethynyl-1,2,3-trimethoxybenzene (**A-16**) (38 mg, 0.20 mmol, 1.0 eq.) was converted to **(*E*)-16**, a yellow liquid (41 mg, 0.15 mmol, 75%).

R$_f$ = 0.65 (Et_2O:*n*-pentane 30:70); **^{1}H NMR** (599 MHz, $CDCl_3$): δ = 7.03 (d, $^3J_{HH}$ = 13.9 Hz, 1H, H2), 6.69 (d, $^3J_{HH}$ = 13.9 Hz, 1H, H1), 6.51 (s, 2H, H4), 3.87 (s, 6H, H7), 3.85 (s, 3H, H8) ppm; **^{13}C{^{1}H} NMR** (126 MHz, $CDCl_3$): δ = 153.6 (C5), 138.6 (C6), 137.2 (C2), 131.8 (C3), 106.0 (C1), 103.5 (C4), 61.0 (C8), 56.2 (C7) ppm; **IR (ATR):** ν = 1612 (w), 1576 (m), 1505 (m), 1463 (m), 1452 (m), 1431 (w), 1415 (m), 1332 (m), 1288 (w), 1239 (s), 1201 (m), 1184 (w), 1150 (m), 1121 (s), 1041 (w), 1003 (m), 986 (m), 935 (m), 837 (w), 772 (m), 656 (w) cm^{-1}; **HR-ESI-MS:** *m/z*: 294.99412 ([M+Na]$^+$, calcd. for $C_{11}H_{13}O_3Na^+$: 294.99458). The analytical data are in agreement with the literature data.[16]

(*Z*)-5-(2-Bromovinyl)-1,2,3-trimethoxybenzene ((*Z*)-16)

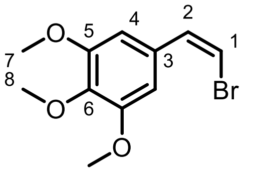

Prepared according to General Procedure C, 3,4,5-trimethoxybenzaldehyde (589 mg, 3.0 mmol, 1.0 eq.) CBr_4 (1.99 g, 6.0 mmol, 2.0 eq.) and PPh_3 (3.15 g, 12.0 mmol, 4.0 eq.) in CH_2Cl_2 (7.5 mL) yielded 5-(2,2-dibromovinyl)-1,2,3-trimethoxybenzene (570 mg, 1.6 mmol, 54%), of which 176 mg, 0.50 mmol, 1.0 eq. was converted to **(*Z*)-16**, a colourless liquid (28 mg, 0.10 mmol, 21%).

R$_f$ = 0.65 (Et_2O:*n*-pentane 30:70); **^{1}H NMR** (500 MHz, $CDCl_3$): δ = 6.99-6.97 (m, 3H, H2+H4), 6.38 (d, $^3J_{HH}$ = 8.1 Hz, 1H, H1), 3.88 (s, 6H, H7), 3.87 (s, 3H, H8) ppm; **^{13}C{^{1}H} NMR** (126 MHz, $CDCl_3$): δ = 153.0 (C5), 138.4 (C6), 132.2 (C2), 130.0 (C3), 106.5 (C4), 105.7 (C1), 61.0 (C8), 56.3 (C7) ppm; **IR (ATR):** ν = 2937 (w), 2836 (w), 1613 (w), 1580 (m), 1504 (m), 1450 (m), 1431 (w), 1414 (s), 1334 (m), 1298 (w), 1237 (s), 1185 (w), 1122 (s), 1038 (w), 1003 (m), 970 (w), 924 (w), 840 (m), 785 (w), 757 (w), 733 (w), 656 (w) cm^{-1}; **HR-ESI-MS:** *m/z*: 294.99400 ([M+Na]$^+$, calcd. for $C_{11}H_{13}O_3BrNa^+$: 294.99403). The analytical data are in agreement with the literature data.[17]

(*Z*,*E*)-5-(2-Bromovinyl)benzo[*d*][1,3]dioxole ((*Z*,*E*)-17)

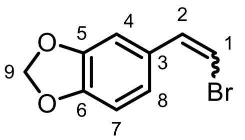

Prepared according to General Procedure A, 5-ethynylbenzo[*d*][1,3]dioxole (**A-17**) (29 mg, 0.20 mmol, 1.0 eq.) was converted to an isomeric mixture of **(*Z*,*E*)-17**, a yellow liquid (1st experiment: 30 mg, 0.13 mmol, 67%, *Z*:*E* = 85:15; 2nd experiment: 31 mg, 0.13 mmol, 67% *Z*:*E* = 84:16, average: 67%, *Z*:*E* = 85:15).

^{1}H NMR (400 MHz, $CDCl_3$): δ = 7.39 (d, $^4J_{HH}$ = 1.6 Hz, 0.85H, H4$_Z$), 7.13-7.05 (m, 6H, 0.85H, H8$_Z$), 7.04-6.93 (m, 1H, H2), 6.86-6.71 (m, 1.15H, H7+H4$_E$), 6.59 (d, $^3J_{HH}$ = 13.9 Hz, 0.15H, H1$_E$), 6.31 (d, $^3J_{HH}$ = 8.1 Hz, 0.85H, H1$_Z$), 6.03-5.91 (m, 2H, H9) ppm.

(*E*)-5-(2-Bromovinyl)benzo[*d*][1,3]dioxole ((*E*)-17)

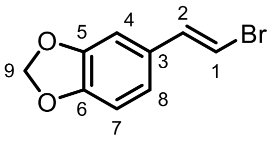

Prepared according to General Procedure B, (with 39 mg, 0.15 mmol, 1.5 eq. Cp_2ZrHCl and 20 mg, 0.11 mmol, 1.1 eq. NBS in 1 mL CH_2Cl_2) 5-ethynylbenzo[*d*][1,3]dioxole (**A-17**) (15 mg, 0.10 mmol, 1.0 eq.) was converted to **(*E*)-17**, a yellow liquid (11 mg, 0.05 mmol, 47%).

R_f = 0.11 (*n*-pentane); **^{1}H NMR** (500 MHz, $CDCl_3$): δ = 7.00 (d, $^3J_{HH}$ = 13.9 Hz, 1H, H2), 6.82-6.80 (m, 1H, H8), 6.77-6.73 (m, 2H, H7+H4), 6.59 (d, $^3J_{HH}$ = 13.9 Hz, 1H, H1), 5.96 (s, 2H, H9) ppm; **^{13}C{^{1}H} NMR** (126 MHz, $CDCl_3$): δ = 148.3 (C6), 147.9 (C5), 136.9 (C2), 130.5 (C3), 121.1 (C4), 108.6 (C7), 105.6 (C8), 104.7 (C1), 101.4 (C9) ppm; **IR (ATR):** ν = 1501 (s), 1488 (s), 1445 (s), 1434 (m), 1350 (w), 1278 (w), 1243 (s), 1182 (m), 1121 (w), 1100 (w), 1036 (s), 927 (s), 856 (w), 815 (m), 795 (w), 767 (s), 738 (m), 712 (w), 659 (w) cm^{-1}; **GC-EI-MS:** *m/z*: 226.01 (100), 228.00 (98) ($[M]^+$, calcd. for $C_9H_7O_2Br^+$: 225.96 (100), 227.96 (98)). The analytical data are in agreement with the literature data.[18]

(*Z*)-5-(2-Bromovinyl)benzo[*d*][1,3]dioxole ((*Z*)-17)

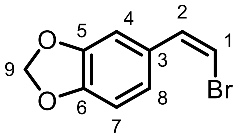

Prepared according to General Procedure C, piperonal (**S-17**) (75 mg, 0.50 mmol, 1.0 eq.), CBr_4 (332 mg, 1.00 mmol, 2.0 eq.) and PPh_3 (525 mg, 2.00 mmol, 4.0 eq.) in CH_2Cl_2 (1.5 mL) yielded 5-(2,2-dibromovinyl)benzo[*d*][1,3]dioxole (115 mg, 0.38 mmol, 76%), of which 61 mg, 0.20 mmol, 1.0 eq. was converted with $Pd(PPh_3)_4$ (11 mg, 0.01 mmol, 0.05 eq.) and $SnBu_3H$ (0.06 mL) in CH_2Cl_2 (0.4 mL) to **(*Z*)-17**, a colourless liquid (43 mg, 0.19 mmol, 95%).
R_f = 0.11 (*n*-pentane); **^{1}H NMR** (599 MHz, $CDCl_3$): δ = 7.39 (d, $^4J_{HH}$ = 1.8 Hz, 1H, H4), 7.11-7.06 (m, 1H, H8), 6.96 (d, $^3J_{HH}$ = 8.1 Hz, 1H, H2), 6.81 (d, $^3J_{HH}$ = 8.1 Hz, 1H, H7), 6.31 (d, $^3J_{HH}$ = 8.1 Hz, 1H, H1), 5.99 (s, 2H, H9) ppm; **^{13}C{^{1}H} NMR** (151 MHz, $CDCl_3$): δ = 147.7 (C5), 147.6 (C6), 131.9 (C2), 129.1 (C3), 124.0 (C8), 108.9 (C4), 108.3 (C7), 104.7 (C1), 101.4 (C9) ppm; **IR (ATR):** ν = 2894 (w), 1625 (w), 1612 (w), 1501 (m), 1486 (s), 1444 (m), 1360 (w), 1321 (m), 1255 (s), 1239 (s), 1208 (m), 1120 (w), 1090 (m), 1036 (s), 925 (m), 862 (m), 812 (s), 784 (m), 729 (w), 705 (m), 675 (m) cm^{-1}; **GC-EI-MS:** *m/z*: 226.01 (96), 228.00 (100) ($[M]^+$, calcd. for $C_9H_7O_2Br^+$: 225.96 (100), 227.96 (98)). The analytical data are in agreement with the literature data.[8]

(*Z*,*E*)-2-Bromo-5-(2-bromovinyl)thiophene ((*Z*,*E*)-22)

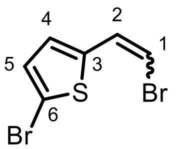

Prepared according to General Procedure A, 2-bromo-5-ethynylthiophene (**A-22**) (37 mg, 0.20 mmol, 1.0 eq.) was converted to an isomeric mixture of **(*Z*,*E*)-22**, a yellow sticky liquid (1st experiment: 39 mg, 0.14 mmol, 72%, *Z*:*E* = 87:13; 2nd experiment: 39 mg, 0.15 mmol, 73% *Z*:*E* = 87:13, average: 73%, *Z*:*E* = 87:13).

^{1}H NMR (400 MHz, $CDCl_3$): δ = 7.22 (d, $^3J_{HH}$ = 7.9 Hz, 0.87H, H_Z), 7.07 (d, $^3J_{HH}$ = 13.9 Hz, 0.13H, $H2_E$) 7.04-6.98 (m, 1.74H, H_Z+H_Z), 6.93 (d, $^3J_{HH}$ = 3.4 Hz, 0.13H, $H4_E$), 6.70 (d, $^3J_{HH}$ = 13.9 Hz, 0.13H, $H5_E$), 6.55 (d, $^3J_{HH}$ = 14.0 Hz, 0.13H, $H1_E$), 6.32 (d, $^3J_{HH}$ = 7.9 Hz, 0.87H, H_Z) ppm.

(*E*)-2-Bromo-5-(2-bromovinyl)thiophene ((*E*)-22)

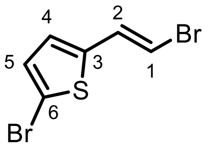

Prepared according to General Procedure B, (with 39 mg, 0.15 mmol, 1.5 eq. Cp_2ZrHCl and 20 mg, 0.11 mmol, 1.1 eq. NBS in 1 mL CH_2Cl_2) 2-bromo-5-ethynylthiophene (**A-22**) (19 mg, 0.1 mmol, 1.0 eq.) was converted to **(*E*)-22**, a yellow sticky liquid (23 mg, 0.08 mmol, 84%).

R_f = 0.48 (*n*-pentane); **^{1}H NMR** (599 MHz, $CDCl_3$): δ = 7.07 (dd, $^3J_{HH}$ = 13.9 Hz, $^5J_{HH}$ = 0.6 Hz, 1H, H2), 6.93 (d, $^3J_{HH}$ = 3.8 Hz, 1H, H4), 6.70 (d, $^3J_{HH}$ = 3.8 Hz, 1H, H5), 6.55 (dd, $^3J_{HH}$ = 13.9 Hz, $^5J_{HH}$ = 0.5 Hz, 1H, H1) ppm; **^{13}C NMR** (151 MHz, $CDCl_3$): δ = 141.6 (C3), 130.5 (C4), 129.8 (C2), 126.4 (C5) 112.1 C6), 106.0 (C1) ppm; **IR (ATR):** ν = 3089 (w), 3059 (w), 1751 (w), 1673 (w), 1602 (w), 1562 (w), 1516 (w), 1427 (s), 1359 (w), 1319 (w), 1276 (w), 1223 (s), 1201 (m), 1176 (w), 1129 (w), 1060 (w), 1049 (w), 968 (m), 918 (s), 887 (m), 799 (s), 770 (s), 756 (s), 724 (s), 678 (m) cm^{-1}; **GC-EI-MS:** *m/z*: ($[M]^+$, calcd. for $C_6H_4Br_2^+$: 267.84 (50), 267.84 (100), 269.84 (52)). The analytical data are in agreement with the literature data.[19]

(*Z*)-2-Bromo-5-(2-bromovinyl)thiophene ((*Z*)-22)

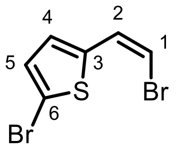

This compound could be isolated from the isomeric mixture obtained in the isomerisation reaction (*vide supra*) after column chromatography on SiO_2 with *n*-pentane as solvent. **(*Z*)-22** was received as yellow sticky oil (11 mg, 0.04 mmol, 21%).
R_f = 0.48 (*n*-pentane); **^{1}H NMR** ((599 MHz, $CDCl_3$): δ = 7.21 (dd, $^3J_{HH}$ = 7.7 Hz, $^5J_{HH}$ = 0.5 Hz, 1H, H2), 7.02 (d, $^3J_{HH}$ = 3.8 Hz, 1H, H4), 7.00 (d, $^3J_{HH}$ = 3.8 Hz, 1H, H5), 6.32 (dd, $^3J_{HH}$ = 7.7 Hz, $^5J_{HH}$ = 0.4 Hz, 1H, H1) ppm; **^{13}C NMR** (126 MHz, $CDCl_3$): δ = 140.0 (C3), 130.4 (C5), 129.2 (C4), 126.4 (C2), 114.6 (C6). 104.8 (C1) ppm; **IR (ATR):** ν = 3083 (w), 2925 (w), 1607 (m), 1507 (w), 1422 (m), 1330 (m), 1296 (m), 1250 (w), 1196 (w), 1137 (w), 1120 (w), 1058 (m), 965 (m),

912 (w), 880 (w), 797 (s), 757 (m), 704 (s), 662 (m) cm^{-1}; **GC-EI-MS:** *m/z*: ([M]$^+$, calcd. for $C_6H_4Br_2^+$: 267.84 (50), 267.84 (100), 269.84 (52)).

(*Z*,*E*)-2-(2-Bromovinyl)thiophene ((*Z*,*E*)-23)

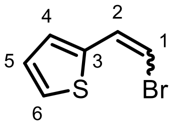

Prepared according to General Procedure A, 2-ethynylthiophene (**A-23**) (22 mg, 0.20 mmol, 1.0 eq.) was converted to an isomeric mixture of **(*Z*,*E*)-23**, a colourless liquid (1st experiment: 15 mg, 0.08 mmol, 40%, *Z*:*E* = 90:10; 2nd experiment: 15 mg, 0.08 mmol, 41% *Z*:*E* = 90:10, average: 41%, *Z*:*E* = 90:10).

^{1}H NMR (400 MHz, $CDCl_3$): δ = 7.39 (d, $^3J_{HH}$ = 5.3 Hz, 0.9H, H1$_Z$), 7.34-7.29 (m, 1.8H, H2$_Z$+H4$_Z$), 7.22-7.17 (m, 0.2H, H6$_E$+H2$_E$), 7.08-7.05 (m, 0.9H, H5$_Z$), 6.99-6.95 (m, 0.2H, H5$_E$+H4$_E$), 6.63 (d, $^3J_{HH}$ = 13.8 Hz, 0.1H, H1$_E$), 6.32 (d, $^3J_{HH}$ = 8.0 Hz, 0.9H, H1$_Z$) ppm.

(*E*)-2-(2-Bromovinyl)thiophene ((*E*)-23)

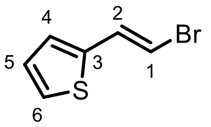

Prepared according to General Procedure B, 2-ethynylthiophene (**A-23**) (22 mg, 0.20 mmol, 1.0 eq.) was converted to **(*E*)-18**, a yellow liquid (18 mg, 0.10 mmol, 49%).

R$_f$ = 0.65 (*n*-pentane); **^{1}H NMR** (599 MHz, $CDCl_3$): δ = 7.22-7.17 (m, 2H, H6+H2), 6.99-6.95 (m, 2H, H5+H4), 6.63 (d, $^3J_{HH}$ = 13.8 Hz, 1H, H1) ppm; **$^{13}C\{^1H\}$ NMR** (151 MHz, $CDCl_3$): δ = 140.1 (C3), 130.4 (C2), 127.6 (C5), 126.2 (C4), 125.2 (C6), 105.3 (C1) ppm; **IR (ATR):** ν = 2922 (m), 2852 (m), 1463 (m), 1431 (m), 1377 (w), 1239 (s), 1213 (m), 1204 (m), 1179 (w), 1079 (w), 1042 (m), 925 (s), 855 (m), 827 (m), 765 (s), 734 (m), 693 (s) cm^{-1}; **GC-EI-MS:** *m/z*: 187.98 (100), 189.96 (82) ([M]$^+$, calcd. for $C_6H_5SBr^+$: 187.92 (98), 189.93 (100)). The analytical data are in agreement with the literature data.[7]

(*Z*)-2-(2-Bromovinyl)thiophene ((*Z*)-23)

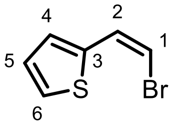

Prepared according to General Procedure C, thiophen-2-carbaldehyde (112 mg, 1.00 mmol, 1.0 eq.) yielded 2-(2,2-dibromovinyl)thiophene (198 mg, 0.74 mmol, 74%), of which 134 mg, 0.5 mmol, 1.0 eq. was converted to **(*Z*)-23**, a colourless liquid (70 mg, 0.37 mmol, 75%).

R$_f$ = 0.65 (*n*-pentane); **^{1}H NMR** (599 MHz, $CDCl_3$): δ = 7.39 (d, $^3J_{HH}$ = 5.2 Hz, 1H, H6), 7.33-7.30 (m, 2H, H2+H4), 7.09-7.06 (m, 1H, H5), 6.33 (d, $^3J_{HH}$ = 7.9 Hz, 1H, H1) ppm; **$^{13}C\{^1H\}$ NMR** (151 MHz, $CDCl_3$): δ = 138.3 (C3), 130.3 (C2), 127.0 (C6), 126.4 (C4+C5), 104.3 (C1) ppm; **IR (ATR):** ν = 3082 (m), 1607 (m), 1537 (w), 1503 (w), 1421 (m), 1352 (m), 1327 (m), 1306 (s), 1254 (m), 1209 (m), 1185 (m), 1135 (m), 1115 (m), 1082 (m), 1049 (m), 911 (m), 856 (s), 829 (s), 812 (m), 760 (m), 744 (w), 693 (s) cm^{-1}; **GC-EI-MS:** *m/z*: 187.97 (94), 189.97 (100) ([M]$^+$, calcd. for $C_6H_5SBr^+$: 187.92 (98), 189.93 (100)). The analytical data are in agreement with the literature data.[20]

(*Z*,*E*)- 3-(2-bromovinyl)thiophene ((*Z*,*E*)-24)

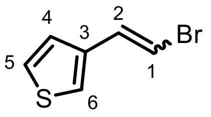

Prepared according to General Procedure A, 3-ethynylthiophene (**A-24**) (22 mg, 0.20 mmol, 1.0 eq.) was converted to an isomeric mixture of **(*Z*,*E*)-24**, a colourless liquid (1st experiment: 22 mg, 0.12 mmol, 59%, *Z*:*E* = 78:22; 2nd experiment: 22 mg, 0.12 mmol, 58% *Z*:*E* = 78:22, average: 59%, *Z*:*E* = 78:22). The analytical data were obtained from the isomeric mixture of the first experiment.

R$_f$ = 0.52 (*n*-pentane);

^{1}H NMR (500 MHz, $CDCl_3$): δ = 7.83-7.79 (m, 0.78H, H6$_Z$), 7.52-7.47 (m, 0.78H, H4$_Z$), 7.33-7.31 (m, 0.78H, H5$_Z$), 7.32-7.27 (m, 0.22H, H5$_E$), 7.18-7.16 (m, 0.22H, H4$_E$), 7.15-7.07 (m, 1.22H, H2+H6$_E$), 6.66 (d, $^3J_{HH}$ = 13.8 Hz, 0.22H, H1$_E$), 6.34 (d, $^3J_{HH}$ = 8.0 Hz, 0.78H, H1$_Z$) ppm; **$^{13}C\{^1H\}$ NMR** (126 MHz, $CDCl_3$): δ = 138.0 (C3$_E$), 136.2 (C3$_Z$), 131.6 (C2$_E$), 128.5 (C4$_Z$), 126.9 (C2$_Z$), 126.7 (C$_E$), 125.9 (C6$_Z$), 125.1 (C5$_Z$), 124.5 (C6$_E$), 122.8 (C4$_E$), 106.3 (C1$_E$), 105.5 (C1$_Z$) ppm; **IR (ATR):** ν = 1614 (w), 1412 (w), 1363 (w), 1314 (m), 1255 (w), 1230 (w), 1202 (w), 1184 (w), 1141 (w), 1084 (w), 935 (w), 875 (w), 838 (w), 829 (w), 788 (s), 753 (s), 733 (m), 711 (w), 688 (m), 668 (w) cm^{-1}; **GC-EI-MS:** *m/z*: 187.98 (98), 189.99 (100) ([M]$^+$, calcd. for $C_6H_5SBr^+$: 187.92 (98), 189.93 (100)). The analytical data are in agreement with the literature data.[21]

Scope of Electrophiles

(*Z*,*E*)-4-(Vinyl-2-*d*)-1,1'-biphenyl ((*Z*,*E*)-19)

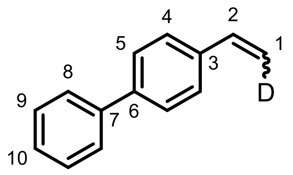

4-Ethynyl-1,1'-biphenyl (**A-9**) (36 mg, 0.20 mmol, 1.0 eq.) was submitted to the conditions of General Procedure A, but after isomerisation the vinyl zirconium species was trapped with deuterium chloride (35% in D_2O, D ≥99%, 24 µL, 0.22 mmol, 1.1 eq.) to afford **(*Z*,*E*)-19**, a colourless solid as an isomeric mixture (13 mg, 0.07 mmol, 36%, *Z*:*E* = 77:23). The isomers were not separated or synthesised separately. NMR assignments were made from the mixture and by comparison with literature values.[22]

R_f = 0.38 (*n*-pentane); **M.p.:** 117 °C; **^{1}H NMR** (599 MHz, $CDCl_3$): δ = 7.63-7.60 (m, 2H, H8), 7.60-7.57 (m, 2H, H5), 7.52-7.48 (m, 2H, H4), 7.47-7.43 (m, 2H, H9), 7.38-7.34 (m, 1H, H10), 6.80-6.74 (m, 1H, H2), 5.79 (d, $^3J_{HH}$ = 17.6 Hz, 0.23H, H1$_E$), 5.28 (d, $^3J_{HH}$ = 10.9 Hz, 1H, H1$_Z$) ppm; **^{13}C NMR** (151 MHz, $CDCl_3$): δ = 140.9 (C7), 140.7 (C6), 136.7 (C3), 136.5 (C2), 128.9 (C9), 127.5 (C10), 127.4 (C5), 127.1 (C8), 126.8 (C4), 114.0-113.6 (m, C1) ppm; **IR (ATR):** ν = 3058 (w), 3034 (w), 3008 (w), 1604 (w), 1580 (w), 1485 (m), 1449 (w), 1403 (m), 1367 (w), 1341 (w), 1228 (w), 1182 (w), 1126 (w), 1040 (w), 1005 (w), 982 (m), 945 (w), 910 (w), 844 (s), 829 (m), 799 (s), 763 (s), 727 (s), 688 (s) cm^{-1}; **GC-EI-MS:** *m/z*: 181.14 (100), 182.14 (15) ($[M+]^+$, calcd. for $C_{14}H_{11}D^+$: 181.10 (100), 182.10 (15)).

(*Z*,*E*)-4-(2-Chlorovinyl)-1,1'-biphenyl ((*Z*,*E*)-20)

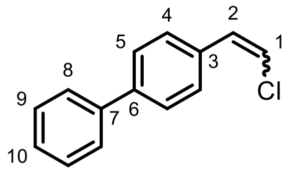

4-Ethynyl-1,1'-biphenyl (**A-9**) (36 mg, 0.20 mmol, 1.0 eq.) was submitted to the conditions of General Procedure A, but after isomerisation the vinyl zirconium species was trapped with NCS (29 mg, 0.22 mmol, 1.1 eq.) to afford **(*Z*,*E*)-20**, a colourless solid, as an isomeric mixture (33 mg, 0.16 mmol, 78%, *Z*:*E* = 80:20). The isomers were not separated or synthesised separately. NMR assignments were made from the mixture and by comparison with literature values.[23]

R_f = 0.28 (*n*-pentane); **M.p.:** 73 °C; **^{1}H NMR** (599 MHz, $CDCl_3$): δ = 7.79-7.76 (m, 1.6H, H4$_Z$), 7.64-7.55 (m, 4H, H9+H5$_Z$+H4$_E$), 7.48-7.43 (m, 2H, H8), 7.40-7.34 (m, 1H, H10), 6.88 (d, $^3J_{HH}$ = 13.7 Hz, 0.2H, H1$_E$), 6.71-6.66 (m, 1H, H2), 6.30 (d, $^3J_{HH}$ = 8.1 Hz, 0.8H, H1$_Z$) ppm; **^{13}C NMR** (151 MHz, $CDCl_3$): δ = 141.1 (C6$_E$), 141.0 (C6$_Z$), 140.7 (C7$_Z$), 140.6 (C7$_E$), 134.0 (C3$_E$), 133.3 (C3$_Z$), 133.0 (C4$_E$), 129.9 (C4$_Z$), 129.03 (C2$_Z$), 128.99 (C2$_E$), 128.98 (C2$_E$), 127.7 (C10$_Z$), 127.6 (C8$_E$), 127.2 (C9$_Z$), 127.10 (C5$_Z$), 127.08 (C5$_E$), 126.7 (C10$_E$), 118.9 (C1$_E$), 117.8 (C1$_Z$) ppm; **IR (ATR):** ν = 3065 (w), 3034 (w),1624 (w), 1606 (w), 1582 (w), 1556 (w), 1483 (m), 1449 (w), 1406 (m), 1349 (w), 1339 (w), 1324 (w), 1247 (w), 1176 (w), 1162 (w), 1130 (w), 1077 (w), 1040 (w), 1005 (w), 954 (w), 936 (m), 912 (w), 841 (s), 798 (w), 769 (s), 758 (s), 737 (m), 722 (m), 687 (s) cm^{-1}; **GC-EI-MS:** *m/z*: 214.09 (100), 215.09 (15), 216.10 (35) ($[M]^+$, calcd. for $C_{14}H_{11}Cl^+$: 214.05 (100), 215.06 (15), 216.05 (33)).

(*Z*,*E*)-4-(2-Iodovinyl)-1,1'-biphenyl ((*Z*,*E*)-21)

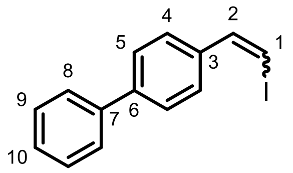

4-Ethynyl-1,1'-biphenyl (**A-9**) (36 mg, 0.20 mmol, 1.0 eq.) was submitted to the conditions of General Procedure A, but after isomerisation the vinyl zirconium species was trapped with NIS (49 mg, 0.22 mmol, 1.1 eq.) to afford **(*Z*,*E*)-21**, a colourless solid as an isomeric mixture (46 mg, 0.15 mmol, 75%, *Z*:*E* = 80:20). The isomers were not separate or synthesised separately. NMR assignments were made from the mixture and by comparison with literature values.[24]

R_f = 0.38 (*n*-pentane); **M.p.:** 68 °C; **^{1}H NMR** (599 MHz, $CDCl_3$): δ = 7.76-7.73 (m, 1.6H, H4$_Z$), 7.65-7.55 (m, 4H, H9+H8$_E$+H5$_Z$), 7.49-7.42 (m, 2.2H, H8$_Z$+H5$_E$+H2$_E$), 7.39-7.34 (m, 2.2H, H10+H4$_E$+H2$_Z$), 6.88 (d, $^3J_{HH}$ = 14.9 Hz, 0.2H, H1$_E$), 6.60 (d, $^3J_{HH}$ = 8.6 Hz, 0.8H, H1$_Z$) ppm; **^{13}C NMR** (151 MHz, $CDCl_3$): δ = 144.7 (C2$_E$), 141.3 (C6$_Z$), 141.3 (C6$_E$), 140.7 (C7$_Z$), 140.5 (C7$_E$), 138.3 (C2$_Z$), 136.8 (C3$_E$), 135.7 (C3$_Z$), 128.99 (C4$_Z$), 128.98 (C8$_Z$+C5$_E$), 127.7 (C10$_E$), 127.7 (C10$_Z$), 127.5 (C8$_E$), 127.2 (C9$_Z$), 127.1 (C9$_E$), 127.0 (C5$_Z$), 126.6 (C6$_E$), 79.2 (C1$_Z$), 76.7 (C1$_E$) ppm; **IR (ATR):** ν = 3053 (w), 3031 (w), 1598 (w), 1484 (m), 1448 (w), 1404 (m), 1328 (w), 1306 (s), 1291 (m), 1277 (w), 1208 (w), 1174 (w), 1158 (w), 1128 (w), 1077 (w), 1005 (m), 959 (w), 943 (w), 911 (w), 841 (s), 767 (s), 755 (s), 736 (s), 727 (s), 695 (m), 670 (s) cm^{-1}; **GC-EI-MS:** *m/z*: 306.01 (100), 307.07 (15) ($[M]^+$, calcd. for $C_{14}H_{11}I^+$: 305.99 (100), 306.99 (C15)).

Hydrozirconation-Isomerisation-Transmetallation Sequence

(*Z*)-1-Methoxy-4-(3-phenylprop-1-en-1-yl)benzene ((*Z*)-18)

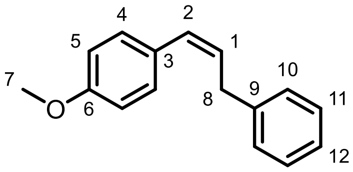

1-Ethynyl-4-methoxybenzene (**A-15**) (26 mg, 0.20 mmol, 1.0 eq.) was submitted to the conditions of General Procedure A, but after isomerisation the vinyl zirconium species was trapped with $Pd(PPh_3)_4$ (7 mg, 0.006 mmol, 0.03 eq.) in THF (0.4 mL). Benzyl bromide (24 µL, 0.20 mmol, 1.0 eq.) was then added, in analogy to the procedure of Race *et al*.[25]. This mixture was stirred at room temperature for 18 h before the solvents were removed under reduced pressure. The residue was dissolved in EtOAc:*n*-pentane (10:90) (10 mL) and filtered over Celite®. Purification by column chromatography (SiO_2, Et_2O:*n*-pentane 0:100 to 3:97) yielded **(*Z*)-18** as a colourless liquid (30 mg, 0.13 mmol, 67%).

R_f = 0.17 (*n*-pentane); **^{1}H NMR** (500 MHz, $CDCl_3$): δ = 7.34-7.19 (m, 7H, H4+H10+H11+H12), 6.93-6.88 (m, 2H, H5), 6.54 (d, $^3J_{HH}$ = 11.5 Hz, 1H, H2), 5.79 (dt, $^3J_{HH}$ = 11.5, 7.4 Hz, 1H, H1), 3.83 (s, 3H, H7), 3.69 (d, $^3J_{HH}$ = 7.4 Hz, 2H, H8) ppm; **^{13}C NMR** (126 MHz, $CDCl_3$): δ = 158.6 (C6), 141.1 (C9), 130.1 (C4), 130.0 (C3), 129.6 (C2), 129.3 (C1), 128.6 (C11), 128.5 (C12), 126.2 (C10), 113.8 (C5), 55.4 (C7), 34.8 (C8) ppm; **IR (ATR):** ν = 1606 (m), 1574 (w), 1509 (s), 1495 (m), 1453 (m), 1441 (w), 1401 (w), 1302 (w), 1249 (s), 1173 (s), 1110 (w), 1075 (w), 1032 (s), 967 (w), 933 (w), 833 (s), 817 (m), 757 (m), 725 (s), 697 (s) cm^{-1}; **GC-EI-MS:** *m/z*: 224.14 (100), 225.17 (20) ($[M]^+$, calcd. for $C_{16}H_{16}O^+$: 224.12 (100), 225.12 (18)).

NMR Analysis of the Vinyl Zirconium Intermediate

The vinyl zirconium intermediates were observed by NMR. Therefore the reaction was conducted similarly to General Procedure A with the following modifications: The reaction was done by suspending Cp_2ZrHCl (51 mg, 0.20 mmol, 1.2 eq.) in CD_2Cl_2 (1.2 mL) and the addition of 1-bromo-4-ethynylbenzene **A-1** (30 mg, 0.17 mmol, 1.0 eq.) in CD_2Cl_2 (0.4 mL). After stirring at room temperature for 15 min, half of the reaction mixture (*i.e.* 0.8 mL) was transferred to an oven dried, vacuum cooled NMR tube under argon. The remaining reaction mixture was irradiated at 400 nm for 45 min and then transferred to an oven dried, vacuum cooled NMR tube under argon.

The not irradiated sample shows only one set of signals belonging to the *E* configured vinyl zirconium species **(*E*)-Zr1**, whereas the irradiated sample shows two sets of signals. The major one for the *Z* configured vinyl zirconium species **(*Z*)-Zr1** and the minor for the *E* configured **(*E*)-Zr1**. All data are in agreement with the literature values.[26]

(*E*)-(4-bromostyryl)dicyclopentadienylzirconium(IV) chloride ((*E*)-Zr1)

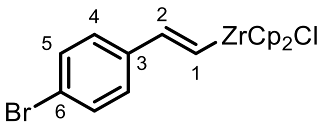

^{1}H NMR (599 MHz, CD_2Cl_2): δ = 7.76 (d, $^3J_{HH}$ = 19.0 Hz, 1H, H1), 7.42-7.40 (m, 2H, H5), 7.20-7.15 (m, 2H, H4), 6.64 (d, $^3J_{HH}$ = 19.0 Hz, 1H, H2), 6.33 (m, 10H, Cp) ppm; **^{13}C NMR** (151 MHz, CD_2Cl_2): δ = 179.1 (C1), 139.6 (C2), 138.7 (C3), 131.8 (C5), 127.8 (C4), 120.4 (C6), 113.7 (Cp) ppm.

Evidence of the in situ formed diene **25**: **HR-APCI-MS:** *m/z*: 361.92988 (55), 363.92794 (100), 365.92586 (45) (calcd. for $C_{16}H_{12}Br_2^+$: 361.9300 (51), 363.92804 (100), 365.92619 (50)).

(*Z*)-(4-bromostyryl)dicyclopentadienylzirconium(IV) chloride ((*Z*)-Zr1)

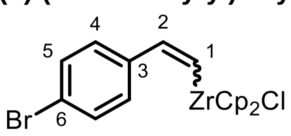

^{1}H NMR (599 MHz, CD_2Cl_2): δ = 7.76 (d, $^3J_{HH}$ = 19.0 Hz, 0.23H, $H1_E$), 7.56 (d, $^3J_{HH}$ = 13.4 Hz, 0.77H, $H2_Z$), 7.51-7.46 (m, 4H, $H5_Z$), 7.42-7.40 (m, 2H, $H5_E$), 7.19-7.16 (m, 2H, $H4_E$), 6.97-6.94 (m, 5H, $H4_Z$), 6.64 (d, $^3J_{HH}$ = 19.0 Hz, 0.77H, $H2_E$), 6.33 (d, $^3J_{HH}$ = 13.4 Hz, 3H, $H1_Z$ [overlaps with Cp_E signal]), 6.33 (s, 2.3H, Cp_E), 6.29 (s, 7.3H, Cp_Z) ppm; **^{13}C NMR** (151 MHz, CD_2Cl_2): δ = 189.6 ($C1_Z$), 179.1 ($C1_E$), 145.2 ($C2_Z$), 141.3 ($C3_Z$), 139.6 ($C2_E$), 138.7 ($C3_E$), 131.8 ($C5_E$), 131.5 ($C5_Z$), 128.7 ($C4_Z$), 127.7 ($C4_E$), 120.8 ($C6_Z$), 120.4 ($C6_E$) 113.7 (Cp_E), 112.8 (Cp_Z) ppm.

Table 2: Comparison of ^{1}H NMR and ^{13}C NMR shifts of **(*Z*)-Zr1** and **(*E*)-Zr1**.

#	δ(H_E) [ppm]	δ(H_Z) [ppm]	Δδ(H_E)-δ(H_Z) [ppm]	δ(C_E) [ppm]	δ(C_Z) [ppm]	Δδ(C_E)-δ(Z_Z) [ppm]
1	7.76	6.33	1.43	179.1	189.6	-10.5
2	6.64	7.56	-0.92	139.6	145.2	-5.6
3	-	-	-	138.7	141.3	-2.6
4	7.17	6.96	0.21	127.8	128.7	-0.9
5	7.41	7.48	-0.07	131.8	131.5	0.3
6	-	-	-	120.4	120.8	-0.4
Cp	6.33	6.29	0.04	113.7	112.8	0.9

Independent Synthesis of the Diene

(1*E*,3*E*)-1,4-Bis(4-bromophenyl)buta-1,3-diene (25)

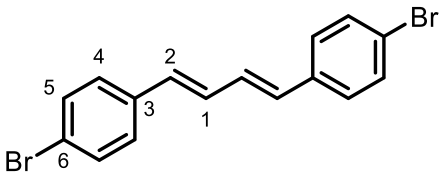

A solution of 4-benzyl bromide (2.0 mmol, 0.50 g, 1.0 eq.) in $CHCl_3$ (30 mL) was heated to 50 °C in a water bath and PPh_3 (2.0 mmol, 0.52 g, 1.0 eq.) was added. The reaction mixture was stirred for 2 h and cooled to room temperature before Et_2O (60 mL) was added. Over several minutes a crystalline product precipitated which was filtered off and washed with additional Et_2O (50 mL). The phosphonium salt was dried and used without further purification. To the suspension of the phosphonium salt in THF (10 mL) NaO*t*Bu (2.0 mmol, 0.19 g, 1.0 eq.) was added and the reaction mixture was stirred for 10 min before (*E*)-3-(4-bromophenyl)acrylaldehyde (2.0 mmol, 0.42 g, 1.0 eq.) was added. The reaction mixture was stirred for 3 h and then it was diluted with EtOAc (20 mL), washed with a sat. aq. solution of NH_4Cl (30 mL) and brine (30 mL). The combined organic layers were dried over $MgSO_4$ and concentrated under reduced pressure. Purification by crystallisation from a boiling mixture of toluene and EtOH (1:2) yielded **25** as a light yellow solid (15 mg, 0.04 mmol, 2%).

M.p.: 212 °C;**^{1}H NMR** (599 MHz, CD_2Cl_2): δ = 7.54-7.43 (m, 2H, H5), 7.37-7.26 (m, 2H, H4), 7.00-6.92 (m, 1H, H2), 6.72-6.61 (m, 1H, H1) ppm; **^{13}C NMR** (151 MHz, CD_2Cl_2): δ = 136.7 (C3), 132.4 (C1), 132.2 (C5), 130.7 (C2), 128.3 (C4), 121.7 (C6) ppm; **IR (ATR):** ν = 2922 (w), 2852 (w), 1581 (w), 1484 (m), 1399 (m), 1283 (w), 1102 (w), 1071 (m), 1007 (m), 991 (s), 959 (w), 851 (s), 829 (w), 796 (s), 747 (w), 702 (w), 667 (w) cm^{-1}; **GC-EI-MS:** *m/z*: 361.9 (53), 363.9 (100), 365.9 (50) ($[M]^+$, calcd. for $C_{16}H_{12}Br_2^+$: 361.93 (51), 363.93 (100), 365.93 (50)). The analytical data are in agreement with the literature data.[27]

Reaction Optimisation
Hydrozirconation

Hydrozirconation conditions were optimised using the conditions specified in Table 3: An oven dried pressure tube (10 mL, cooled to room temperature under vacuum and refilled with argon) was charged with Cp_2ZrHCl (X eq.), evacuated and refilled with argon. Dry solvent (1.5 mL) and 1-Bromo-4-ethynylalkyne (36 mg, 0.20 mmol, 1.0 eq.) dissolved in an additional portion of the solvent (0.5 mL, 0.1 M in total) were added under argon at room temperature. The mixture was stirred at room temperature for the given time. The reaction mixture was then quenched by the addition of NBS (X eq.) and stirred for 15 min at room temperature. Water (10 mL) was added and the separated aqueous layer was extracted with CH_2Cl_2 (3 x 10 mL). The combined organic layers were washed with water (10 mL), dried over $MgSO_4$, filtered and concentrated under reduced pressure. The crude mixture (containing the excess reagents and decomposition products) was filtered over a short plug of silica and rinsed with *n*-pentane to afford the *E* vinyl bromide after removal of the solvents under reduced pressure. DMF was added as internal standard to calculate the yield using the methyl signals of the DMF.

Table 3: Optimisation of the hydrozirconation.[a]

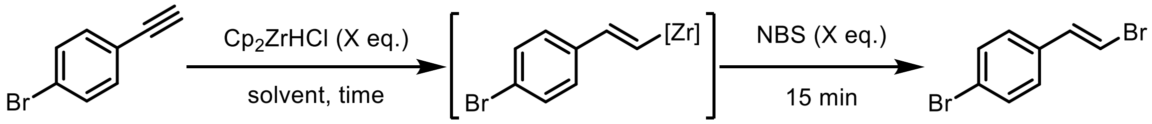

A-1 **(*E*)-Zr1** **(*E*)-1**

[Zr] = $ZrCp_2Cl$

entry	time with Cp_2ZrHCl	eq. of Cp_2ZrHCl	eq. of NBS	solvent	yield exp. 1[b]	yield exp. 2[b]	Ø yield[b]
1	15 min	1.0	1.0	CH_2Cl_2	63%	63%	63%
2	30 min	1.0	1.0	CH_2Cl_2	62%	61%	62%
3	60 min	1.0	1.0	CH_2Cl_2	62%	64%	63%
4	120 min	1.0	1.0	CH_2Cl_2	62%	59%	61%
5	240 min	1.0	1.0	CH_2Cl_2	56%	57%	57%
6	15 min	1.1	1.0	CH_2Cl_2	62%	56%	59%
7	15 min	1.2	1.0	CH_2Cl_2	69%	71%	70%
8	15 min	1.3	1.0	CH_2Cl_2	62%	69%	66%
9	15 min	1.0	1.1	CH_2Cl_2	69%	65%	67%
10	15 min	1.2	1.1	CH_2Cl_2	79%	82%	81%
11	90 min[c]	1.2	1.1	MeCN	-[d]	-[d]	-[d]
12	15 min	1.2	1.1	THF	38%	35%	37%
13	90 min[c]	1.2	1.1	toluene	61%	63%	62%
14	90 min[c]	1.2	1.1	cyclohexane	-[d]	-[d]	-[d]
15	15 min	1.2	1.1	DCE	75%	70%	73%

[a]Cp_2ZrHCl (X eq.), solvent (1.5 mL), alkyne (36 mg, 0.20 mmol, 1.0 eq.) in solvent (0.5 mL) quenched with NBS (X eq.); [b]determined by ^{1}H-NMR with DMF as internal standard; [c]reaction time prolonged due to inhomogeneous reaction mixture; [d]no homogeneous reaction mixture and no product observed.

Isomerisation

The isomerisation step was optimised using the conditions specified in Table 4: An oven dried pressure tube (10 mL, cooled to room temperature under vacuum and refilled with argon) was charged with Cp_2ZrHCl (62 mg, 0.24 mmol, 1.2 eq.), evacuated and refilled with argon. Dry CH_2Cl_2 (1.5 mL) and the alkyne (0.20 mmol, 1.0 eq.) dissolved in an additional portion of CH_2Cl_2 (0.5 mL, 0.1 M in total) were added under argon at room temperature. The mixture was stirred at room temperature for 15 min. Then, if applicable, a photocatalyst (PC) (0.01 mmol, 0.05 eq.) was added under argon and the pressure tube was placed in the LED set up (Figure 1) and irradiated for the given time at the given wavelength. The reaction mixture was then quenched by addition of NBS (39 mg, 0.22 mmol, 1.1 eq.) and stirred for 15 min at room temperature. Water (10 mL) was added and the separated aqueous layer was extracted with CH_2Cl_2 (3 x 10 mL). The combined organic layers were washed with water (10 mL), dried over $MgSO_4$, filtered and concentrated under reduced pressure. The crude mixture (containing the excess reagents and decomposition products) was filtered over a large plug of silica and rinsed with *n*-pentane to afford the isomeric mixture of the *Z* and *E* vinyl bromide after removal of the solvent under reduced pressure. DMF was added as internal standard to calculate the yield using the methyl signals of the DMF. The *Z*:*E* ratio, which was determined by integrating the proton signals *β* to the aromatic ring in the ^{1}H NMR.

Table 4: Optimisation of the isomerisation.[a]

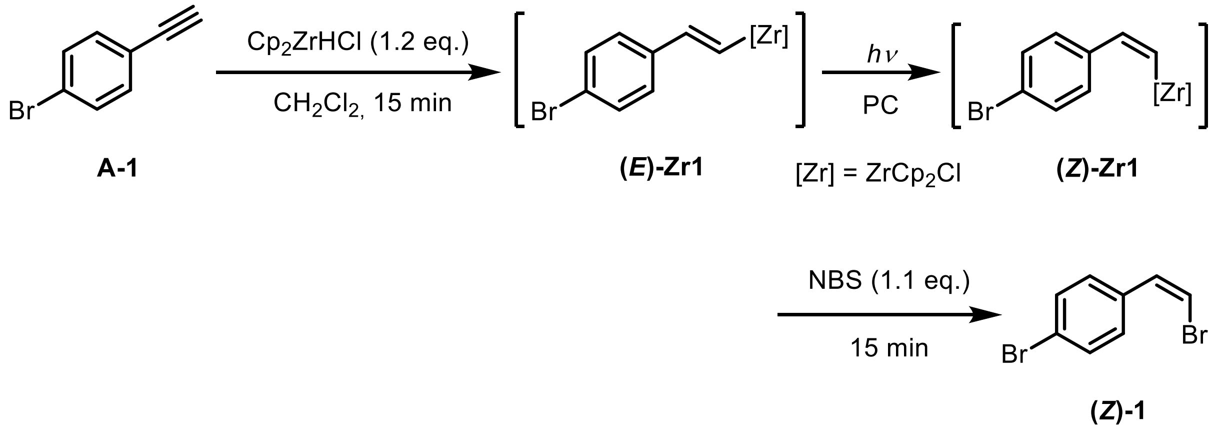

entry	photocatalyst (PC) (5 mol%)	λ [nm]	time [min]	yield exp. 1[b]	*Z*:*E* ratio exp. 1[b]	yield exp. 2[b]	*Z*:*E* ratio exp. 2[b]	Ø yield[b]	Ø *Z*:*E* ratio[b]
1	-	400	15	80%	51:49	72%	50:50	76%	50:50
2	-	400	30	72%	69:31	71%	67:33	72%	68:32
3	-	400	45	73% (73%)	74:26 73:27	75% (74%)	73:27 72:28	74% (74%)	74:26 (73:27)
4	-	400	60	53%	73:27	55%	73:27	54%	73:27
5	-	400	90	48%	73:27	49%	72:28	49%	73:27
6	-	369	45	66%	30:70	69%	23:77	66%	27:73
7	-	374	45	62%	43:57	59%	45:55	61%	44:56
8	-	383	45	63%	53:47	65%	52:48	64%	53:47
9	-	414	45	64%	58:42	69%	61:39	67%	60:40
10	-	435	45	70%	26:74	73%	25:75	72%	26:74
11	-	520	45	64%	<5:95	70%	<5:95	67%	<5:95
12	anthracene	369	45	50%	33:67	47%	37:63	48%	35:65
13	(-)-riboflavin tetra acetate	400	45	49%	52:48	52%	52:48	50%	52:48
14	benzil	400	45	49%	73:27	52%	73:27	51%	73:27
15	$Ir(ppy)_3$	400	45	58%	71:29	57%	70:30	58%	71:29
16	$Ir(L_1)(L_2)PF_6$[d]	400	45	70%	56:44	66%	56:44	68%	56:44
17	xanthone	400	45	64%	73:27	61%	73:27	63%	73:27
18	methylene blue	520	45	33%	0:100	35%	0:100	34%	0:100

[a]Cp_2ZrHCl (62 mg, 0.24 mmol, 1.2 eq.), CH_2Cl_2 (1.5 mL), alkyne (36 mg, 0.20 mmol, 1.0 eq.) in CH_2Cl_2 (0.5 mL) quenched with NBS (39 mg, 0.22 mmol, 1.1 eq.); [b]determined by ^{1}H-NMR with DMF as internal standard, isolated yield and *Z*:*E*-ratio in parentheses; [d]$Ir(L_1)L_2)PF_6$ = $Ir([dF(CF_3)ppy]_2(dtbppy))PF_6$.

Synthesis of Alkynes

1-Ethynyl-4-iodobenzene (A-4)

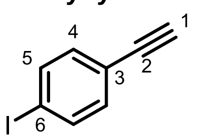

Prepared according to a procedure by Echavarren *et al*.[28] An oven dried Schlenk flask was charged with DIPA (0.84 mL, 6.0 mmol, 1.2 eq.) and dry THF (40 mL), and cooled to 0 °C before *n*-BuLi (1.6 M in hexane, 3.75 mL, 6.0 mmol, 1.2 eq.) was added. After 15 min stirring at 0 °C, TMS-diazomethane (2.0 M in hexane, 3.0 mL, 6.0 mmol, 1.2 eq.) was added and stirring at 0 °C continued for 20 min. Subsequently, 4-iodobenzaldehyde (1.16 g, 5.0 mmol, 1.0 eq.) in dry THF (10 mL) was added and the reaction mixture was stirred for 18 h while warming to room temperature. After quenching with water (10 mL), the crude product was extracted with Et_2O (3 x 10 mL). The combined organic layers were washed with HCl (1 M) and brine and dried over $MgSO_4$. After filtration and concentration under reduced pressure, purification by column chromatography (SiO_2, *n*-pentane) yielded **A-4** as a colourless solid (250 mg, 1.1 mmol, 22%).

R_f = 0.56 (*n*-pentane); **M.p.:** 75 °C; **^{1}H NMR** (500 MHz, $CDCl_3$): δ = 7.70-7.64 (m, 2H, H5), 7.23-7.18 (m, 2H, H4), 3.13 (s, 1H, H1) ppm; **^{13}C NMR** (126 MHz, $CDCl_3$): δ = 137.7 (C5), 133.7 (C4), 121.8 (C3), 95.0 (C6), 82.9 (C2), 78.7 (C1) ppm; **IR (ATR):** ν = 3262 (m), 1903 (w), 1579 (w), 1479 (m), 1467 (m), 1388 (m), 1343 (w), 1298 (w), 1263 (w), 1251 (w), 1228 (w), 1192 (w), 1180 (w), 1111 (w), 1094 (w), 1059 (w), 1049 (w), 1005 (s), 958 (w), 949 (w), 815 (s), 764 (w), 705 (m), 695 (m), 673 (m) cm^{-1}; **GC-EI-MS:** *m/z*: 227.98 ($[M]^+$, calcd. for $C_8H_5IBr^+$: 227.94). The analytical data are in agreement with the literature data.[29]

5-Ethynyl-1,2,3-trimethoxybenzene (A-16)

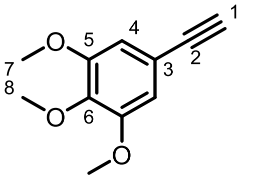

Prepared according to a literature procedure by Echavarren *et al*.[28] An oven dried Schlenk flask was charged with DIPA (0.50 mL, 3.6 mmol, 1.2 eq.) and dry THF (25 mL), and cooled to 0 °C before *n*-BuLi (1.6 M in hexane, 2.25 mL, 3.6 mmol, 1.2 eq.) was added. After 15 min stirring at 0 °C, TMS-diazomethane (2.0 M in hexane, 1.8 mL, 3.6 mmol, 1.2 eq.) was added and stirring at 0 °C continued for 20 min. Subsequently, 3,4,5-trimethoxybenzaldehyde (589 mg, 3.0 mmol, 1.0 eq.) in dry THF (5 mL) was added and the reaction mixture was stirred for 18 h while warming to room temperature. After quenching with water (20 mL), the crude product was extracted with Et_2O (3 x 20 mL). The combined organic layers were washed with HCl (1 M, 20 mL) and brine (20 mL) and dried over $MgSO_4$. After filtration and concentrated under reduced pressure purification by column chromatography (SiO_2, Et_2O:*n*-pentane 40:60) yielded **A-16** as a colourless solid (258 mg, 1.3 mmol, 45%).

R_f = 0.69 (Et_2O:*n*-pentane = 30:70); **M.p.:** 70 °C; **^{1}H NMR** (599 MHz, $CDCl_3$): δ = 6.72 (s, 2H, H4), 3.85 (s, 3H, H8), 3.85 (s, 6H, H7), 3.03 (s, 1H, H1) ppm; **^{13}C NMR** (126 MHz, $CDCl_3$): δ = 153.2 (C5), 139.5 (C6), 117.2 (C3), 109.5 (C4), 83.8 (C2), 76.3 (C1), 61.1 (C8), 56.3 (C7) ppm; **IR (ATR):** ν = 3243 (m), 2989 (w), 2941 (w), 1577 (s), 1502 (m), 1470 (w), 1463 (w), 1450 (m), 1428 (w), 1411 (s), 1331 (m), 1232 (s), 1193 (w), 1181 (w), 1125 (s), 1035 (w), 998 (s), 957 (w), 919 (w), 831 (s), 774 (w), 744 (w), 715 (m), 672 (m) cm^{-1}; **HR-ESI-MS:** *m/z*: 215.06776 ($[M+Na]^+$, calcd. for $C_{11}H_{12}O_3Na^+$: 215.06787). The analytical data are in agreement with the literature data.[30]

Piperonal (S-17)

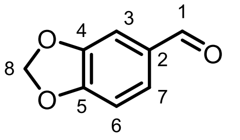

Prepared according to a procedure by Zhu et al.[31] In an oven dried Schlenk flask, vanillin (1.52 g, 10 mmol, 1.0 eq.) was dissolved in dry CH_2Cl_2 (20 mL) and cooled to 0 °C. At 0 °C, BBr_3 (1 M in CH_2Cl_2, 20 mL, 20.0 mmol, 2.0 eq.) was added slowly. After stirring at room temperature for 8 h, water (20 mL) was added and the crude 3,4-dihydroxy benzaldehyde was extracted with CH_2Cl_2 (3 x 20 mL) and EtOAc (3 x 20 mL). The combined organic layers were washed with. sat. aq. $NaHCO_3$ solution, dried over $MgSO_4$, filtered and concentrated under reduced pressure. The residual crude 3,4-dihydroxy benzaldehyde (1.17 g, ca. 8.5 mmol, ca. 85%, 1.0 eq. for the next step) was transferred to an oven dried Schlenk flask and dissolved in MeCN (17 mL). K_2CO_3 (3.52 g, 25.5 mmol, 3.0 eq.) and dibromomethane (0.72 mL, 10.2 mmol, 1.2 eq.) were added and the reaction mixture was stirred at 90 °C for 24 h. Then, water (10 mL) was added and the crude piperonal was extracted with EtOAc (3 x 10 mL). The combined organic layers were dried over $MgSO_4$, filtered and concentrated under reduced pressure. Purification by column chromatography (SiO2, Et_2O:*n*-pentane 0:100 to 10:90 to 30:70) yielded piperonal (**S-17**) as a white solid (665 mg, 4.4 mmol, 52% (44% over 2 steps)).

R_f = 0.62 (Et_2O:*n*-pentane = 30:70); **M.p.:** 36 °C; **^{1}H NMR** (500 MHz, $CDCl_3$): δ = 9.81 (d, $^4J_{HH}$ = 0.6 Hz, 1H, H1), 7.41 (ddd, $^3J_{HH}$ = 7.9 Hz, $^4J_{HH}$ = 1.6 Hz, $^4J_{HH}$ = 0.6 Hz, 1H, H7), 7.33 (d, $^4J_{HH}$ = 1.6 Hz, 1H, H3), 6.93 (d, $^3J_{HH}$ = 7.9 Hz, 1H, H6), 6.07 (s, 1H, H8) ppm; **^{13}C NMR** (126 MHz, $CDCl_3$): δ = 191.0 (C1), 153.3 (C5), 149.4 (C4), 132.0 (C2), 128.8 (C7), 108.0 (C6), 107.1 (C4), 102.2 (C8) ppm; **IR (ATR):**

ν = 2919 (w), 2853 (w), 2795 (w), 1672 (s), 1624 (w), 1600 (m), 1493 (m), 1447 (m), 1419 (m), 1400 (m), 1276 (w), 1254 (m), 1127 (w), 1116 (m), 1096 (m), 1035 (m), 1014 (m), 945 (m), 929 (s), 864 (m), 858 (s), 812 (s), 785 (m), 727 (w), 719 (m), 666 (w) cm^{-1}; **HR-ESI-MS:** *m/z*: 151.03896 ([M+H]$^+$, calcd. for $C_8H_7O_3^+$: 151.03897). The analytical data are in agreement with the literature data.[32]

5-Ethynylbenzo[*d*][1,3]dioxole (A-17)

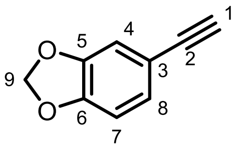

Prepared according to a literature procedure by Echavarren *et al*.[7] An oven dried Schlenk flask was charged with DIPA (0.51 mL, 3.6 mmol, 1.2 eq.) and dry THF (25 mL), and cooled to 0 °C before *n*-BuLi (1.6 M in hexane, 2.25 mL, 3.6 mmol, 1.2 eq.) was added. After 15 min stirring at 0 °C, TMS-diazomethane (2.0 M in Et_2O, 1.8 mL, 3.6 mmol, 1.2 eq.) was added and stirring at 0 °C continued for 20 min. Subsequently, piperonal (**S-17**) (450 mg, 3.0 mmol, 1.0 eq.) in dry THF (5 mL) was added and the reaction mixture was stirred for 18 h while warming up to room temperature. After quenching with water (5 mL), the crude product was extracted with Et_2O (3 x 10 mL). The combined organic layers were washed with HCl (1 M, 10 mL) and brine (10 mL) and dried over $MgSO_4$. After filtration, the crude material was concentrated under reduced pressure and purification by column chromatography (SiO_2, Et_2O:*n*-pentane 15:85) yielded **A-17** as a colourless liquid (92 mg, 0.6 mmol, 21%).
R_f = 0.37 (Et_2O:n-pentane = 20:80); **^{1}H NMR** (500 MHz, $CDCl_3$): δ = 7.03 (dd, $^3J_{HH}$ = 8.0 Hz, $^4J_{HH}$ = 1.6 Hz, 1H, H8), 6.93 (d, $^4J_{HH}$ = 1.6 Hz, 1H, H4), 6.75 (d, $^3J_{HH}$ = 8.1 Hz, 1H, H7), 5.97 (s, 2H, H9), 2.97 (s, 1H, H1) ppm; **^{13}C NMR** (126 MHz, $CDCl_3$): δ = 148.4 (C6), 147.5 (C5), 127.0 (C8), 115.4 (C3), 112.2 (C4), 108.6 (C7), 101.5 (C9), 83.7 (C2), 75.7 (C1) ppm; **IR (ATR):** ν = 3287 (w), 1604 (w), 1502 (m), 1478 (s), 1435 (m), 1330 (w), 1242 (s), 1188 (m), 1121 (m), 1092 (m), 1036 (s), 937 (m), 920 (m), 861 (m), 809 (s), 738 (w), 723 (w), 712 (w), 655 (m) cm^{-1}; **GC-EI-MS:** *m/z*: 146.08 ([M]$^+$, calcd. for $C_9H_6O_2Br^+$: 146.04). The analytical data are in agreement with the literature data.[30]

Mechanistic investigation

Diene 25 as photosensitiser in the isomerisation of *E*-configured alkenes

An oven dried pressure tube (10 mL, cooled to room temperature under vacuum and refilled with argon) was charged with an, in Table 5 specified, *E*-configured alkene (0.2 mmol, 1.0 eq.), evacuated, refilled with argon. Dry CD_2Cl_2 (1.5 mL) and the diene **25** (0.5 mL from a 0.01 mM stock solution in CD_2Cl_2 (0.1 M in total)), 0.005 mmol, 2.5 mol%) were added and the reaction mixture was placed in the LED set up (Figure 1) and irradiated at λ = 400 nm for 45 min. Then a part of the reaction mixture (ca. 0.7 mL) were transferred to an oven dried, vacuum cooled NMR tube under argon for analysis (*cf*. Table 5 and Figure 3 and 4).

Table 5: Diene **25** as possible photosensitiser in the isomerisation of *E*-configured alkenes.[a]

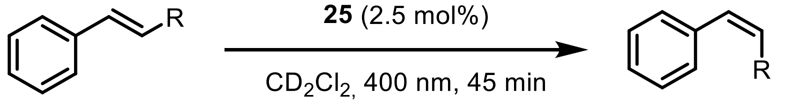

R = Ph: **(*E*)-26** R = Ph: **(*Z*)-26**
R = Me: **(*E*)-27** R = Me: **(*Z*)-27**

entry	R[1]	R[2]	*Z*:*E*[b]
1	Ph	Ph	44:56
2[c]	Ph	Ph	<5:95
3	Ph	Me	47:53
4[c]	Ph	Me	<5:95

[a] Reaction conditions: see text above; [b] determined by ^{1}H-NMR of the crude reaction mixture; [c] no diene **25** was added.

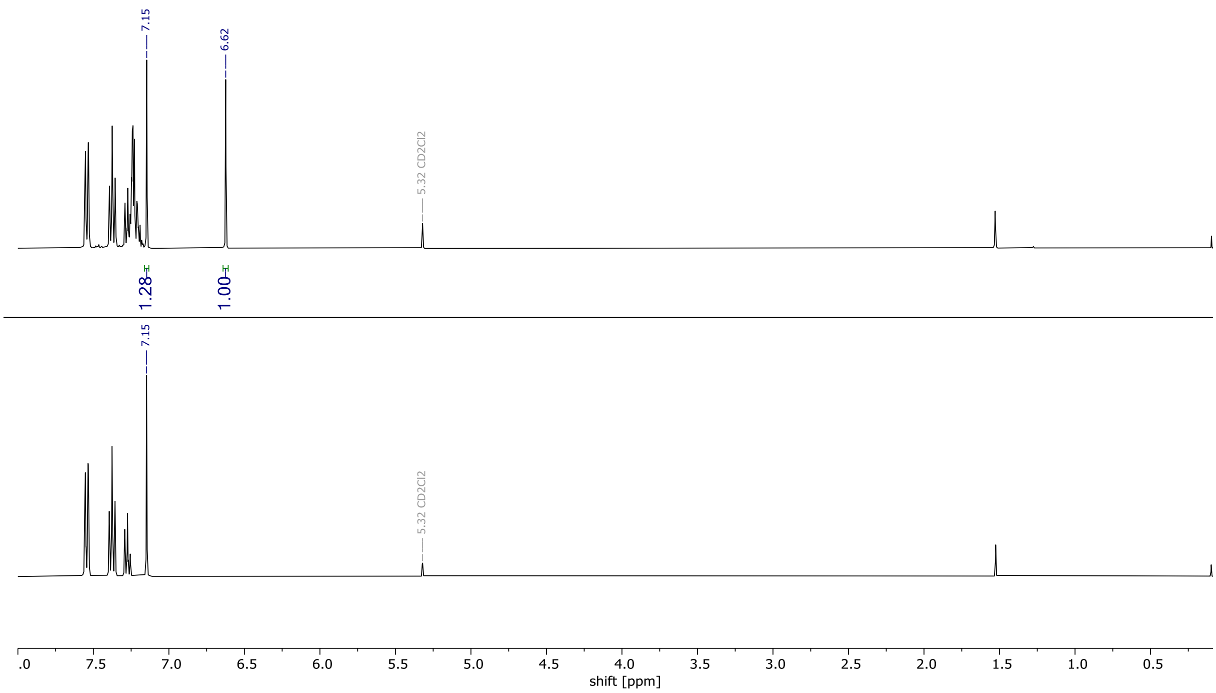

Figure 3: ^{1}H-NMR spectra of the reaction mixture after 45 min irradiation at λ = 400 nm; top: Table 5, entry 1; bottom: Table 5, entry 2.

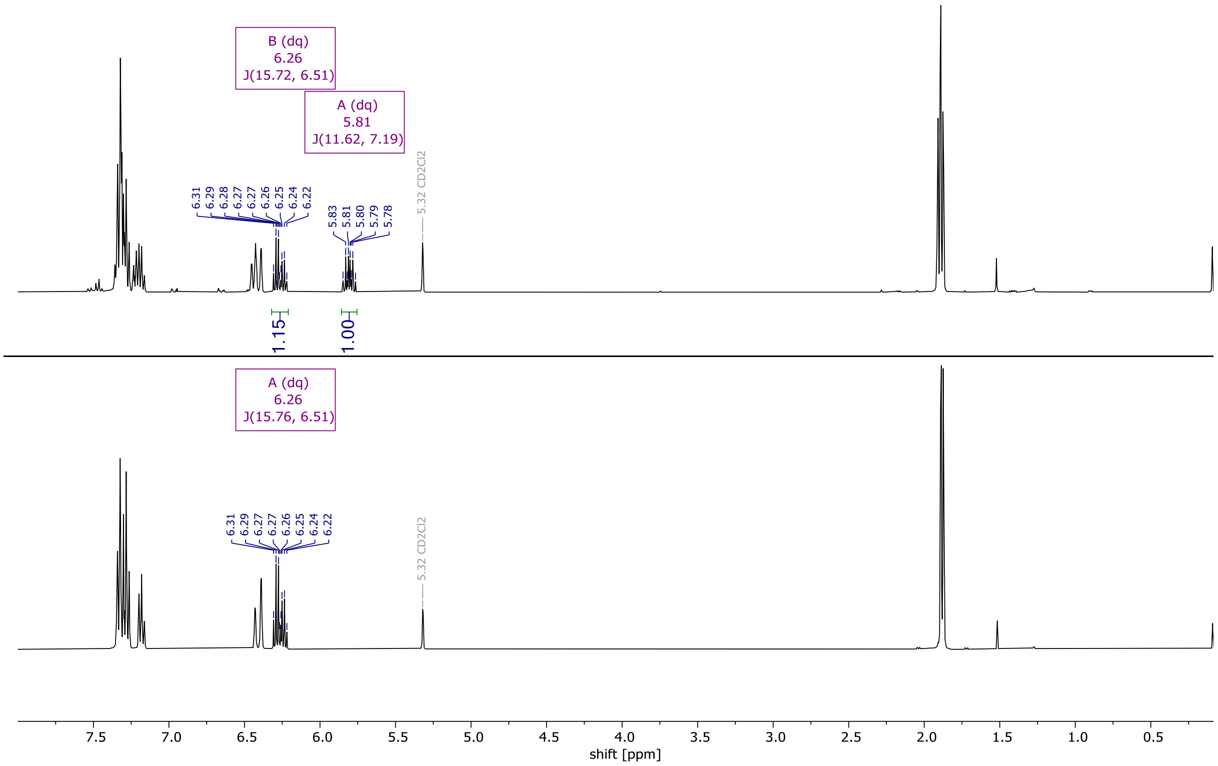

Figure 4: ^{1}H-NMR spectra of the reaction mixture after 45 min irradiation at λ = 400 nm; top: Table 5, entry 3; bottom: Table 5, entry 4.

Standard reaction doped with diene 25

An oven dried pressure tube (10 mL, cooled to room temperature under vacuum and refilled with argon) was charged with Cp_2ZrHCl (62 mg, 0.24 mmol 1.2 eq.), evacuated and refilled with argon. Dry CD_2Cl_2 (1.5 mL - X mL) and the alkyne (36 mg, 0.20 mmol, 1.0 eq.) dissolved in an additional portion of CH_2Cl_2 (0.5 mL) were added under argon at room temperature. The mixture was stirred at room temperature for 15 min. Then, diene **25** (X mL from a 0.01 mM stock solution in CD_2Cl_2 (0.1 M in total)) was added under argon and the pressure tube was placed in the LED set up (Figure 1) and irradiated for 5 min at λ = 400 nm before a part of the reaction mixture (ca. 0.7 mL) were transferred to an oven dried, vacuum cooled NMR tube under argon for analysis (*cf.* Table 6 and Figure 5).

Table 6: Standard reaction doped with diene **25** stopped after Y min.[a]

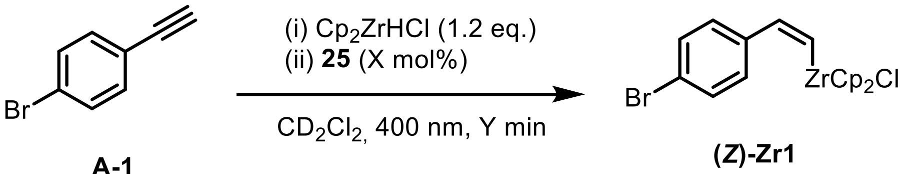

A-1 **(*Z*)-Zr1**

entry	amount of diene 25 added	*Z*:*E* after 5 min[b]
1	-	21:79
2	0.5 mol%	23:77
3	1.0 mol%	24:76
4	2.5 mol%	30:70

[a] Reaction conditions: see text above; [b] determined by ^{1}H-NMR of the crude reaction mixture.

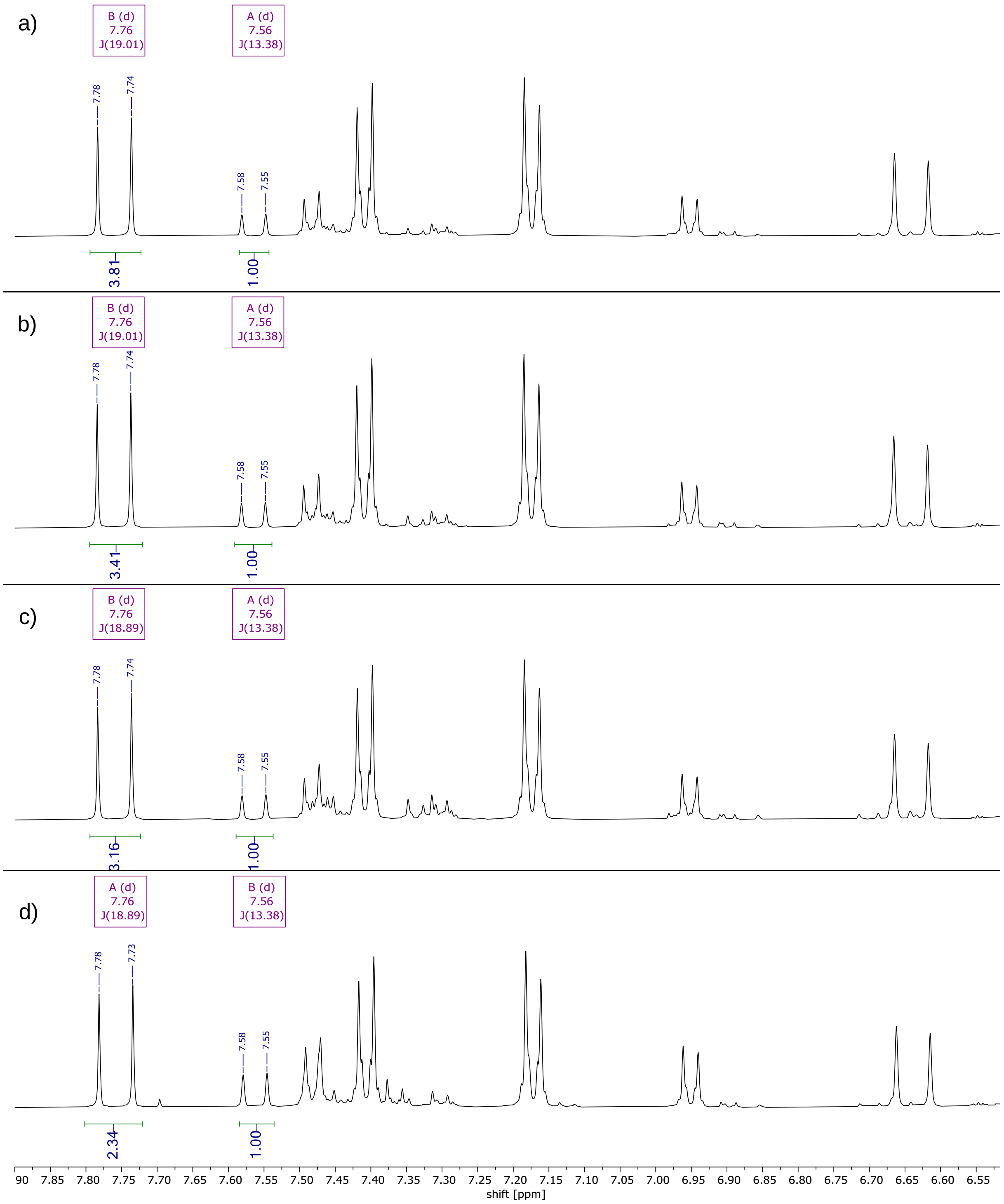

Figure 5: Selected area of the ^{1}H-NMR spectra of the standard reaction mixture after 5 min irradiation at λ = 400 nm; a) no diene **25** added; b) 0.5 mol% diene added; c) 1.0 mol% diene added; d) 2.5 mol% diene added (*cf.* Table 6).

UV/Vis-Absorption Spectra

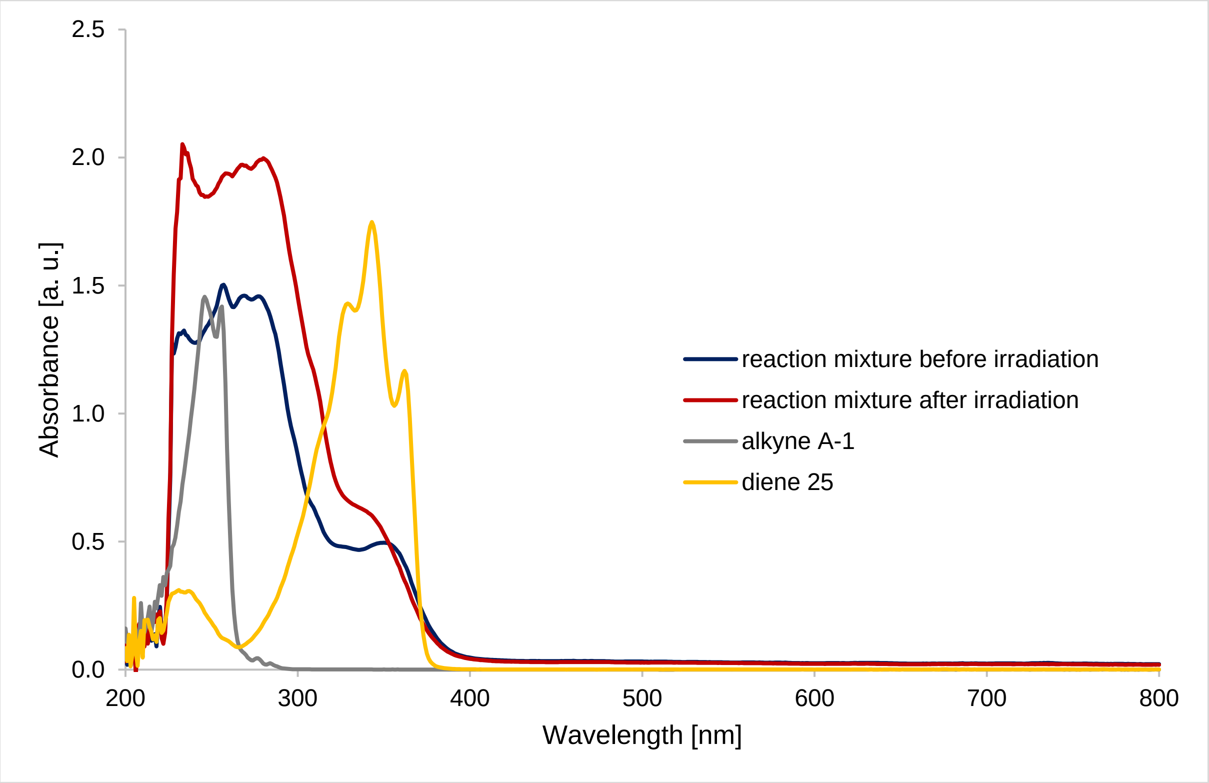

Figure 6: Absorption spectra. Blue: Reaction mixture before irradiation; red: reaction mixture after irradiation at 400 nm; grey: alkyne **A-1**; yellow: diene **25**.

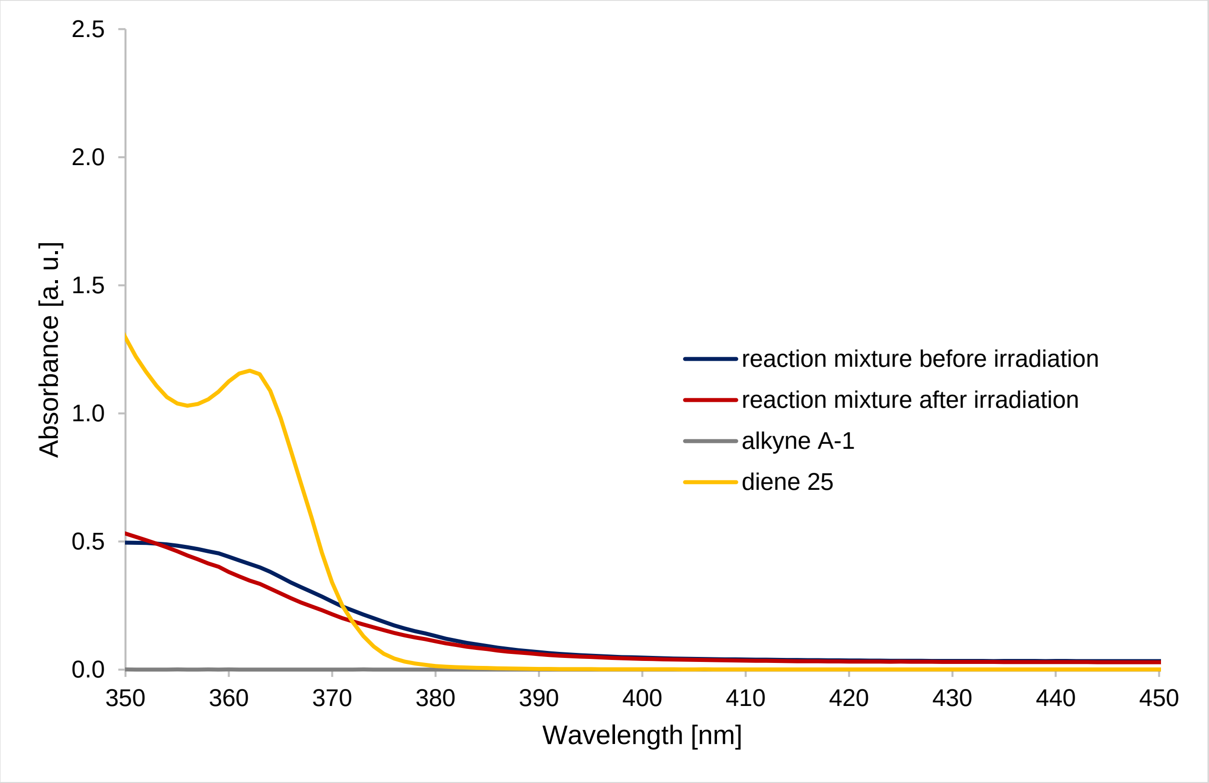

Figure 7: Absorption spectra. Blue: Reaction mixture before irradiation; red: reaction mixture after irradiation at λ = 400 nm; grey: alkyne **A-1**; yellow: diene **25**.

Fluorescence Spectra

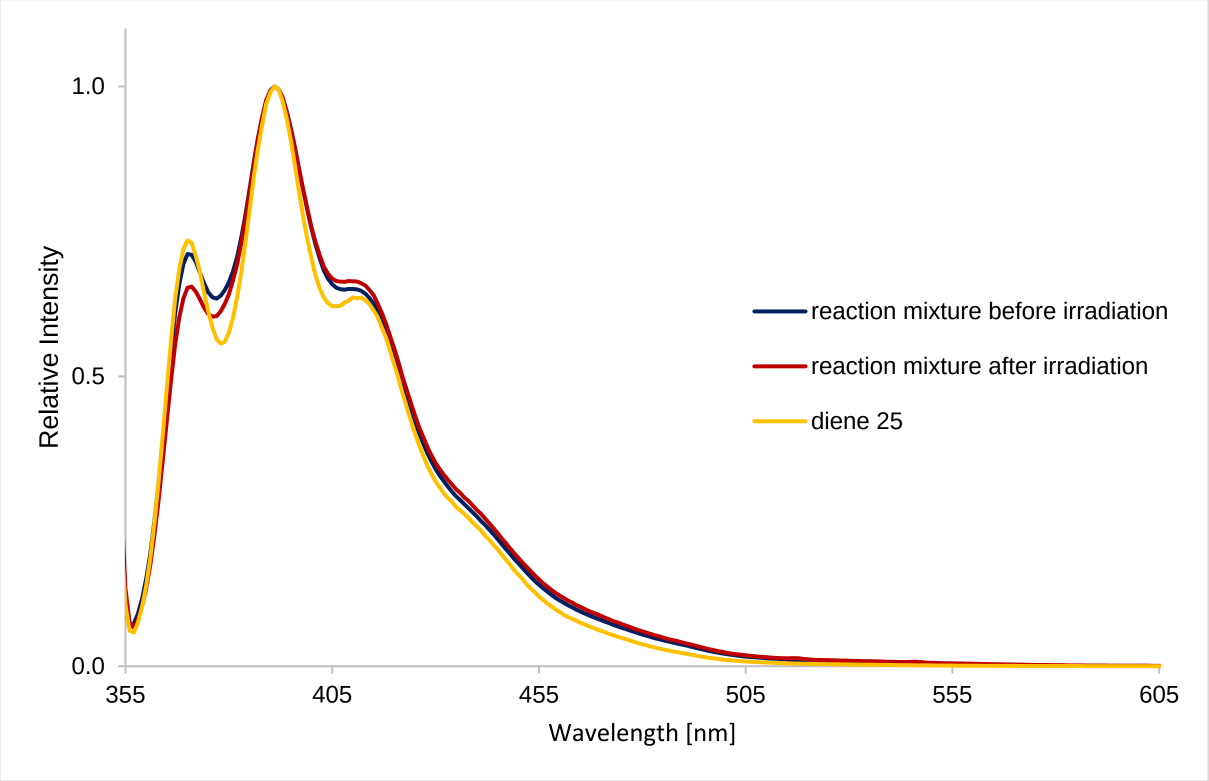

Figure 8: Fluorescence spectra (excitation at λ = 350 nm). Blue: reaction mixture before irradiation; red: reaction mixture after irradiation at λ = 400 nm; grey: alkyne **A-1**; yellow: diene **25**.

Computational Details

Method

All calculation performed in this study were done in Gaussian 16, Revision B.01.[33] Optimisation of all structures was done without geometrical constraints using ωB97X-D long-range-corrected hybrid density functional with atom-atom dispersion corrections[34], known to perform well within the context of structure optimisation containing transition metals.[35] For all calculations a triple zeta basis set def2-TZVP by Ahlrichs and co-workers was used.[36] Dichloromethane solvation was implemented integrating the implicit solvent model which was described to apply the polarisable continuum model (PCM) using the integral equation formalism. Stationary points were investigated *via* frequency analysis; all minima were without the presence of imaginary frequencies. Gibbs free energies were calculated at 298.15 K and 1.00 atm. Singlet and triplet excitation energies were calculated using time-dependent DFT (TD-DFT) with hybrid functional CAM-B3LYP[37] and def2-TZVP basis sets. For visualisation and rendering images of structures the CYLview20[38] software was used.

Results

Structure	E(ωB97X-D) [E_h]	ΔG^{298}_{rel}(ωB97X-D) [kJ/mol]
**(*E*)-Zr(1)**	-3777.19808355	5.5
**(*E*)-Zr(2)**	-3777.19820122	0.0
**(*Z*)-Zr(1)**	-3777.19783407	4.6
**(*Z*)-Zr(2)**	-3777.19272065	22.1

Dihedral angles

Structure	C^1-C^2-C^3-C^4 [°]	C^3-C^4-Zr-Cl [°]
**(*E*)-Zr(1)**	-8.05	-134.5
**(*E*)-Zr(2)**	6.00	6.5
**(*Z*)-Zr(1)**	-20.53	-161.4
**(*Z*)-Zr(2)**	-44.15	-4.4

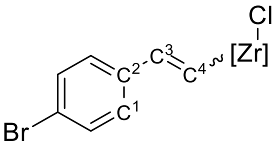

TD-DFT (CAM-B3LYP)

Singlet state transitions only ([nm], oscillator strength)

Transition #	**(*E*)-Zr(1)**	**(*E*)-Zr(2)**	**(*Z*)-Zr(1)**	**(*Z*)-Zr(2)**
1	314.72, 0.5239	311.56, 0.0325	299.59, 0.1580	299.13, 0.0207
2	295.22, 0.1574	283.84, 0.0504	284.82, 0.1530	280.87, 0.0073
3	283.54, 0.0019	275.36, 0.0665	273.14, 0.0189	272.57, 0.0114
4	263.30, 0.0276	273.66, 0.9288	268.79, 0.3438	256.90, 0.0845

Coordinates of optimised structures

(*E*)-Zr(1)

Lowest frequency: 17.46 cm^{-1}

C	4.30166	1.17591	-0.62944
C	2.92768	1.35638	-0.65166
C	2.05699	0.4363	-0.06712
C	2.61604	-0.67922	0.56011
C	3.98472	-0.87474	0.59544
C	4.81952	0.05588	-0.00453
H	4.95651	1.90035	-1.09418
H	2.52168	2.23397	-1.14143
H	1.97633	-1.41098	1.03652
H	4.39821	-1.74443	1.08786
C	0.60337	0.67963	-0.14421
H	0.36265	1.6499	-0.57525
C	-0.38326	-0.14779	0.21223
H	-0.05453	-1.11649	0.60598
Zr	-2.64681	-0.07319	0.11402

C	-1.89777	-1.74713	-1.63769
H	-0.86447	-1.75248	-1.94101
C	-2.45827	-2.50675	-0.58945
C	-2.92162	-0.96521	-2.20472
H	-1.9237	-3.17912	0.06277
C	-3.83504	-2.21406	-0.52605
H	-2.81006	-0.27622	-3.02662
C	-4.12477	-1.25398	-1.51473
H	-4.53603	-2.62062	0.1853
H	-5.0922	-0.82104	-1.71461
C	-2.32407	2.21478	-0.87297
H	-1.57434	2.33268	-1.63959
C	-3.67495	1.88095	-1.08681
C	-2.12975	2.3753	0.51953
H	-4.14031	1.70476	-2.04272
C	-4.30986	1.80101	0.17004
H	-1.20111	2.62503	1.00475
C	-3.35347	2.12263	1.16038
H	-5.34425	1.5468	0.3434
H	-3.52626	2.13769	2.22379
Cl	-3.00777	-0.89108	2.41567
Br	6.69924	-0.21123	0.03622

(*E*)-Zr(2)
Lowest frequency: 10.99 cm^{-1}

C	4.263308	-0.54524	1.151509
C	2.885433	-0.58541	1.296035
C	2.028629	-0.08608	0.314365
C	2.607533	0.470543	-0.82817
C	3.980418	0.519943	-0.99043
C	4.800648	0.008537	0.003602
H	4.906226	-0.94118	1.9259
H	2.464468	-1.01902	2.195733
H	1.979696	0.879417	-1.60967
H	4.408261	0.956444	-1.88284
C	0.569988	-0.1685	0.52861
H	0.300704	-0.54661	1.513159
C	-0.39859	0.151583	-0.33529
H	-0.0693	0.51674	-1.31213
Zr	-2.6268	-0.01156	0.053006
C	-2.1404	-1.71007	-1.75679
H	-1.2261	-1.61625	-2.31905
C	-2.30741	-2.45118	-0.5664
C	-3.37215	-1.10619	-2.07045
H	-1.53433	-2.99559	-0.04787
C	-3.64674	-2.32174	-0.15687
H	-3.57319	-0.47788	-2.92316
C	-4.30716	-1.47879	-1.07364

H	-4.08182	-2.75362	0.729943
H	-5.34459	-1.18709	-1.03402
C	-3.05078	2.043758	-1.3068
H	-2.84929	2.001304	-2.36566
C	-4.27791	1.727131	-0.67914
C	-2.14205	2.447191	-0.30514
H	-5.17665	1.39793	-1.17504
C	-4.11801	1.919973	0.706213
H	-1.12324	2.758356	-0.46302
C	-2.79529	2.356736	0.938145
H	-4.86571	1.735537	1.461913
H	-2.35698	2.56269	1.901286
Cl	-2.48827	-0.62162	2.44396
Br	6.686779	0.074442	-0.21369

(*Z*)-Zr(1)
Lowest frequency: 19.90 cm^{-1}

C	-3.94115	1.60114	0.08065
C	-2.72086	2.243	-0.04636
C	-1.55094	1.54061	-0.34424
C	-1.65624	0.16398	-0.54057
C	-2.86685	-0.49624	-0.42099
C	-4.00276	0.23004	-0.10474
H	-4.83273	2.16439	0.32032
H	-2.67353	3.31601	0.09798
H	-0.77661	-0.40426	-0.80401
H	-2.92418	-1.56456	-0.58011
C	-0.27787	2.27123	-0.44402
H	-0.41101	3.34278	-0.5946
C	0.95824	1.77233	-0.34785
H	1.72457	2.5498	-0.48297
Zr	2.2606	-0.02856	0.06123
C	1.49083	-0.34976	-2.32901
H	0.62432	0.18914	-2.67372
C	2.82753	0.09862	-2.40618
C	1.49335	-1.616	-1.71363
H	3.15431	1.04865	-2.79847
C	3.65725	-0.90318	-1.86492
H	0.63109	-2.23439	-1.52289
C	2.83751	-1.95724	-1.42147
H	4.72921	-0.85031	-1.76403
H	3.17501	-2.87349	-0.96487
C	0.59053	-0.13796	1.95172
H	-0.27979	0.49473	1.91259
C	0.69505	-1.43481	1.41091
C	1.82922	0.18772	2.54056
H	-0.08955	-1.97711	0.90838
C	1.99735	-1.9189	1.68293

H	2.0805	1.12604	3.00879
C	2.69328	-0.91627	2.38305
H	2.38205	-2.88918	1.41383
H	3.72011	-0.96704	2.70793
Cl	4.3194	1.27492	0.51451
Br	-5.66881	-0.66493	0.06356

(*Z*)-Zr(2)
Lowest frequency: 36.61 cm^{-1}

C	-3.72981	1.43162	0.65719
C	-2.53187	1.8073	1.24035
C	-1.48454	0.89919	1.40713
C	-1.69338	-0.41743	1.00442
C	-2.88696	-0.81401	0.42137
C	-3.89375	0.11814	0.24694
H	-4.52498	2.15278	0.52538
H	-2.40289	2.83313	1.56544
H	-0.91975	-1.15712	1.16395
H	-3.02784	-1.84092	0.11275
C	-0.22175	1.34161	2.03211
H	-0.36871	2.00895	2.88267
C	1.01858	1.0325	1.64353
H	1.78352	1.47403	2.29086
Zr	2.01789	-0.06559	-0.11538
C	2.25636	-1.59527	1.89958
H	1.57199	-1.47219	2.72219
C	2.07661	-2.43632	0.78888
C	3.48538	-0.91581	1.73738
H	1.22143	-3.06453	0.59927
C	3.19526	-2.28581	-0.06271
H	3.90285	-0.19027	2.41761
C	4.07275	-1.3614	0.5363
H	3.34689	-2.78701	-1.00575
H	5.0244	-1.04425	0.14293
C	2.54941	2.40932	-0.32058
H	2.21685	3.07753	0.45505
C	3.76543	1.70143	-0.33711
C	1.83238	2.07666	-1.48823
H	4.53415	1.74064	0.41832
C	3.8109	0.9442	-1.53602
H	0.84527	2.42656	-1.74689
C	2.62224	1.18959	-2.24592
H	4.61952	0.30922	-1.85983
H	2.34404	0.73974	-3.18464
Cl	0.4329	-1.10967	-1.72413
Br	-5.53432	-0.41347	-0.55205

Optimised structures

(*E*)-Zr(1)

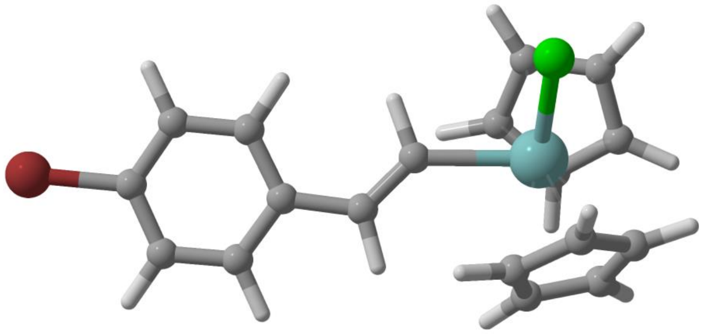

(*E*)-Zr(2)

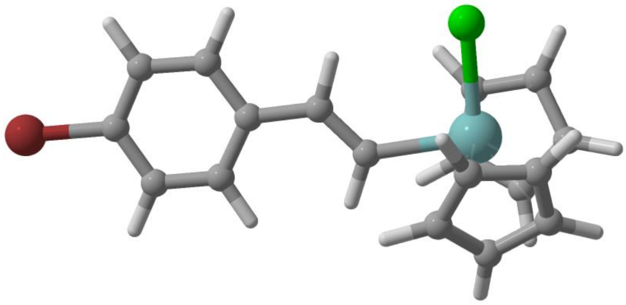

(*Z*)-Zr(1)

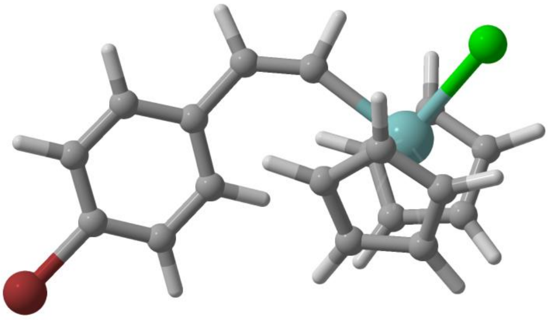

(*Z*)-Zr(2)

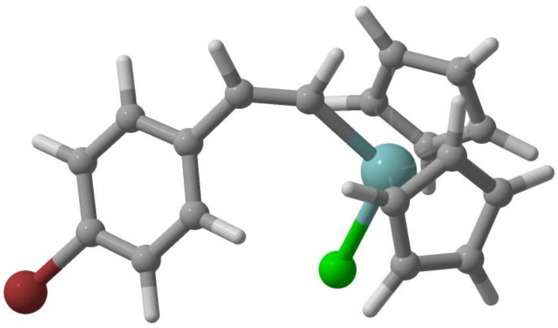

References

[1] Bergueiro, J.; Montenegro, J.; Cambeiro, F.; Saá, C.; López, S. Cross-Coupling Reactions of Organosilicon Compounds in the Stereocontrolled Synthesis of Retinoids. *Chem. Eur. J.* **2012**, *18*, 4401-4410.

[2] Uenishi, J.; Kawahama,R.; Yonemitsu, O.; Tsuji, J. Stereoselective Hydrogenolysis of 1,1-Dibromo-1-alkenes and Stereospecific Synthesis of Conjugated (*Z*)-Alkenyl Compounds. *J. Org. Chem.* **1998**, *63*, 8965-8975.

[3] Rao, M. L. N.; Jadhav, D. N.; Dasgupta, P. Pd-Catalyzed Domino Synthesis of Internal Alkynes Using Triarylbismuths as Multicoupling Organometallic Nucleophiles. *Org. Lett.* **2010**, *12*, 2048-2051.

[4] Pappula, V.; Reddy Donthiri, R.; Mohan, D. C.; Adimurthy, S. H-*β*- Zeolite Catalyzed Synthesis of *β*-Bromostyrenes from Styrenebromohydrins. *Tetrahedron Lett.* **2014**, *55*, 1793-1795.

[5], Xva, W.;Zhang, W.; Zhang, F.; Kuang, C. Stereoselective synthesis of (*Z*)-*β*-arylvinyl bromides from anti-2,3-dibromo-3-arylpropanoic acids. *J. Chem. Res.* **2014**, *38*, 115-117.

[6], Müller, D.; Alexakis, A. Formation of Quaternary Stereogenic Centers by Copper-Catalyzed Asymmetric Conjugate Addition Reactions of Alkenylaluminums to Trisubstituted Enones. *Chem. Eur. J.* **2013**, *19*, 15226-15239.

[7] Ye, Y.; Chen, H.; Yao, K.; Gong, H. Iron-Catalyzed Reductive Vinylation of Tertiary Alkyl Oxalates with Activated Vinyl Halides. *Org. Lett.* **2020**, *22*, 2070-2075.

[8] Lebrun, M.-E.; Le Marquand, P.; Berthelette, C. Stereoselective Synthesis of *Z* Alkenyl Halides *via* Julia Olefination. *J. Org. Chem.* **2006**, *71*, 2009-2013.

[9] Aleksey. V. Shastin, A. V.; Nenajdenko, V. G.; Korotchenko, V. N.; Balenkovab, E. S.New method for the synthesis of *β*-bromostyrenes. *Russ. Chem. Bull. Int. Ed.* **2001**, *50*, 1401-1405.

[10] Ranu, B. C.; Banerjee, S.; and Gupta, J. Ionic Liquid–Promoted Stereoselective Synthesis of (*Z*)-Vinyl Bromides by [bmIm]OH under Organic Solvent–Free Conditions: A Green Approach. *Synth. Commun.* **2017**, *37*, 2869-2876.

[11] Zhang, H.; Xu, Q.; Yu, L.; Yu, S. Photocatalytic Isomerisation of Styrenyl Halides: Stereodivergent Synthesis of Functionalized Alkenes. *Eur. J. Org. Chem*. **2020**, *10*, 1472-1477.

[12] Huang, L.; Rueping, M. Direct Cross-Coupling of Allylic C(sp^3)−H Bonds with Aryl- and Vinylbromides by Combined Nickel and Visible-Light Catalysis. *Angew. Chem. Int. Ed.* **2018**, *57*, 1033-10337.

[13] Kuanga, C.; Yang, Q.; Senbokuc, H.; Tokudac, M. Stereoselective Synthesis of (*E*)-*β*-Arylvinyl Bromides by Microwave-Induced Hunsdiecker-Type Reaction. *Synthesis* **2005**, *8*, 1319-1325.

[14] Hao, T.-T.; Liang, H.-R.; Ou-Yang, Y.-H.; Yin, C.-Z.; Zheng, X.-L.; Yuan, M. L.; Li, R.-X.; Fu, H. Y.; Chen, H. Palladium-Catalyzed Domino Reaction for Stereoselective Synthesis of Multisubstituted Olefins: Construction of Blue Luminogens. *J. Org. Chem.* **2018**, *83*, 4441-4454.

[15] Tang, K. T.; Kent, G. T.; Erden, I.; Wu, W. *cis*-*β*-Bromostyrene derivatives from cinnamic acids via a tandem substitutive bromination-decarboxylation sequence. *Tetrahedron Lett.* **2012**, *58*, 3894-3896.

[16];Camacho-Davila, A. A. Kumada–Corriu Cross Coupling Route to the Anti-Cancer Agent Combretastatin A-4. *Synth. Commun.* **2008**, *38*, 3828-3833.

[17] Herz, H.-G.; Queiroz, M. J. R. P., Maasa, G. 2-Arylvinylation of 1-Methylindole by Palladium-Catalyzed Cross-Coupling Reactions. *Synthesis* **1999**, *6*, 1013-1016.

[18] Chang, D.; Gu, Y.; Shen, Q. Pd-Catalyzed Difluoromethylation of Vinyl Bromides, Triflates, Tosylates, and Nonaflates. *Chem. Eur. J.* **2015**, *21*, 6074-6078.

[19] Bagli, J. F.; Ferdinandi, E. Thiophene Isosters of Phenylethanolamines. *Can. J. Chem*. **1975**, *53*, 2598-2607.

[20] He, L.-Y.; Schulz-Senft, M.; Thiedemann, B.; Linshoeft, J.; Gates, P. J.; Straubitz, A. Nucleophile-Selective Cross-Coupling Reactions with Vinyl and Alkynyl Bromides on a Dinucleophilic Aromatic Substrate. *Eur. J. Org. Chem.* **2015**, *11*, 2498-2502.

[21] Uehling, M. R.; Rucker, R. P.; Lalic, G. Catalytic *Anti*-Markovnikov Hydrobromination of Alkynes. *J. Am. Chem. Soc.* **2014**, *136*, 8799-8803.

[22] Itoh, T.; Kanzaki, Y.; Shimizu, Y.; Kanai, M. Copper(I)-Catalyzed Enantio- and Diastereodivergent Borylative Coupling of Styrenes and Imines. *Angew. Chem. Int. Ed.* **2018**, *57*, 8265-8269.

[23] Matsuda, T.; Suzuki, K.; Miura, N. Rhodium-Catalyzed Cross-Coupling of Alkenyl Halides with Arylboron Compounds. *Adv. Synth. Catal.* **2013**, *355*, 3396-3400.

[24] Pawluć, P.; Hreczycho, G.; Szudkowska, J.; Kubicki, M; Marciniec, B. New One-Pot Synthesis of (*E*)-*β*-Aryl Vinyl Halides from Styrenes. *Org. Lett.* **2009**, *11*, 3390-3393.

[25] Xu, S.; Holst, H. M.; McGuire, S. B.; Race, N. J. Reagent control enables selective and regiodivergent opening of unsymmetrical phenonium ions. *J. Am. Chem. Soc* **2020**, *142*, 8090-8096.

[26] Czisch, P.; Erker, G. Die Bildung (*E*)(*Z*)-Isomerer Alkenylzirconocen-Komplexe. *J. Organomet. Chem*. **1983**, *253*, C9-C11.

[27] Ren, S.; Seki, T.; Necas, D.; Shimizu, H.; Nakajima, K.; Kanno, K.; Song, Z.; Takahashi, T. Selective Dimerization of Aryl-Substituted Terminal Alkynes on Bis(Indenyl)Zirconocene Derivatives. *Chem. Lett.* **2011**, *40*, 1443–1444.

[28] de Orbe, M. E; Zanini, M.; Quinonero, O.; Echavarren, A. M. Gold- or Indium-Catalyzed Cross-Coupling of Bromoalkynes with Allylsilanes through a Concealed Rearrangement. *ACS Catal.* **2019**, *9*, 7817-7822.

[29] Zha, G.-F.; Fang, W.-Y.; Li, Y.-G.; Leng, J.; Chen, X.; Qin, H.-L. SO_2F_2-Mediated Oxidative Dehydrogenation and Dehydration of Alcohols to Alkynes. *J. Am. Chem. Soc.* **2018**, *140*, 17666-17673.

[30] Andrade, C. B.;Carvalho, D. B.; Trefzger, O. S.; Kassab, N. M.; Guerrero, P. G. Jr.; Barbosa, S. L.; Shiguemoto, C. Y. K.; Baroni, A. C. M. One-Pot Synthesis of Unsymmetrical 1,3-Butadiyne Derivatives and their Application in the Synthesis of Unsymmetrical 2,5-Diarylthiophenes. *Eur. J. Org. Chem.* **2019**, *4*, 696-704.

[31] Wang, F.; Wang, X.; Zhang, M-X.; Yang, Y.-H.; Zhu, H.-L. Synthesis, biological evaluation and molecular modeling of 1H-benzo[d]imidazole derivatives as novel anti-tubulin polymerisation agents. *RSC Adv*. **2015**, *5*, 74425-74437.

[32] Iinuma, M.; Moriyama, K.; Togo, H. Oxidation of Alcohols to Aldehydes or Ketones with 1-Acetoxy-1,2-benziodoxole-3(1H)-one Derivatives. *Eur. J. Org. Chem.* **2014**, *4*, 772-780.

[33] Gaussian 16, Revision B.01, Frisch, M. J.; Trucks, G. W.; Schlegel, H. B.; Scuseria, G. E.; Robb, M. A.; Cheeseman, J. R.; Scalmani, G.; Barone, V.; Petersson, G. A.; Nakatsuji, H. ; Li, X.; Caricato, M.; Marenich, A. V.; Bloino, J. ; Janesko, B. G.; Gomperts, R.; Mennucci, B.; Hratchian, H. P.; Ortiz, J. V.; Izmaylov, A. F.; Sonnenberg, J. L.; Williams-Young, D.; Ding, F.; Lipparini, F.; Egidi, F.; Goings, J.; Peng, B.; Petrone, A.; Henderson, T.; Ranasinghe, D.; Zakrzewski, V. G.; Gao, J.; Rega, N.; Zheng, G.; Liang, W.; Hada, M.; Ehara, M.; Toyota, K.; Fukuda, R.; Hasegawa, J.; Ishida, M.; Nakajima, T.; Honda, Y.; Kitao, O.; Nakai, H.; Vreven, T.; Throssell, K.; Montgomery, J. A. Jr.; Peralta, J. E.; Ogliaro, F.; Bearpark, M. J.; Heyd, J. J.; Brothers, E. N.; Kudin, K. N.; Staroverov, V. N.; Keith, T. A.; Kobayashi, R.; Normand, J.; Raghavachari, K.; Rendell, A. P.; Burant, J. C.; Iyengar, S. S.; Tomasi, J.; Cossi, M.; Millam, J. M.; Klene, M.; Adamo, C.; Cammi, R.; Ochterski, J. W.; Martin, R. L.; Morokuma, K.; Farkas, O.; Foresman, J. B.; Fox D. J., Gaussian, Inc., Wallingford CT, 2016.

[34] Chai, J.-D.; Head-Gordon, M. Long-Range Corrected Hybrid Density Functionals with Damped Atom-Atom Dispersion Corrections. *Phys. Chem. Chem. Phys*. **2008**, 10, 6615-6620.

[35] Minenkov, Y.; Singstad, A.; Occhipinti, G.; Jensen, V. R. The Accuracy of DFT-Optimized Geometries of Functional Transition Metal Compounds: A Validation Study of Catalysts for Olefin Metathesis and Other Reactions in the Homogeneous Phase. *Dalton Trans.* **2012**, *41*, 5526-5541.

[36] Weigend, F.; Ahlrichs, R. Balanced Basis Sets of Split Valence, Triple Zeta Valence and Quadruple Zeta Valence Quality for H to R_n: Design and Assessment of Accuracy. *Phys. Chem. Chem. Phys*. **2005**, 7, 3297-3305.

[37] Yanai, T.; Tew, D. P.; Handy, N. C. A New Hybrid Exchange–Correlation Functional Using the Coulomb-Attenuating Method (CAM-B3LYP). *Chem. Phys. Lett*. **2004**, 393, 51-57.

[38] CYLview20; Legault, C. Y., Université de Sherbrooke, 2020 (http://www.cylview.org)

(*E*,*Z*)-1-Bromo-4-(2-vinyl)benzene (*E*,*Z*)-1

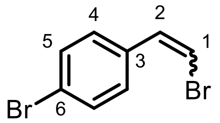

^{1}H NMR (400 MHz, $CDCl_3$), experiment 1

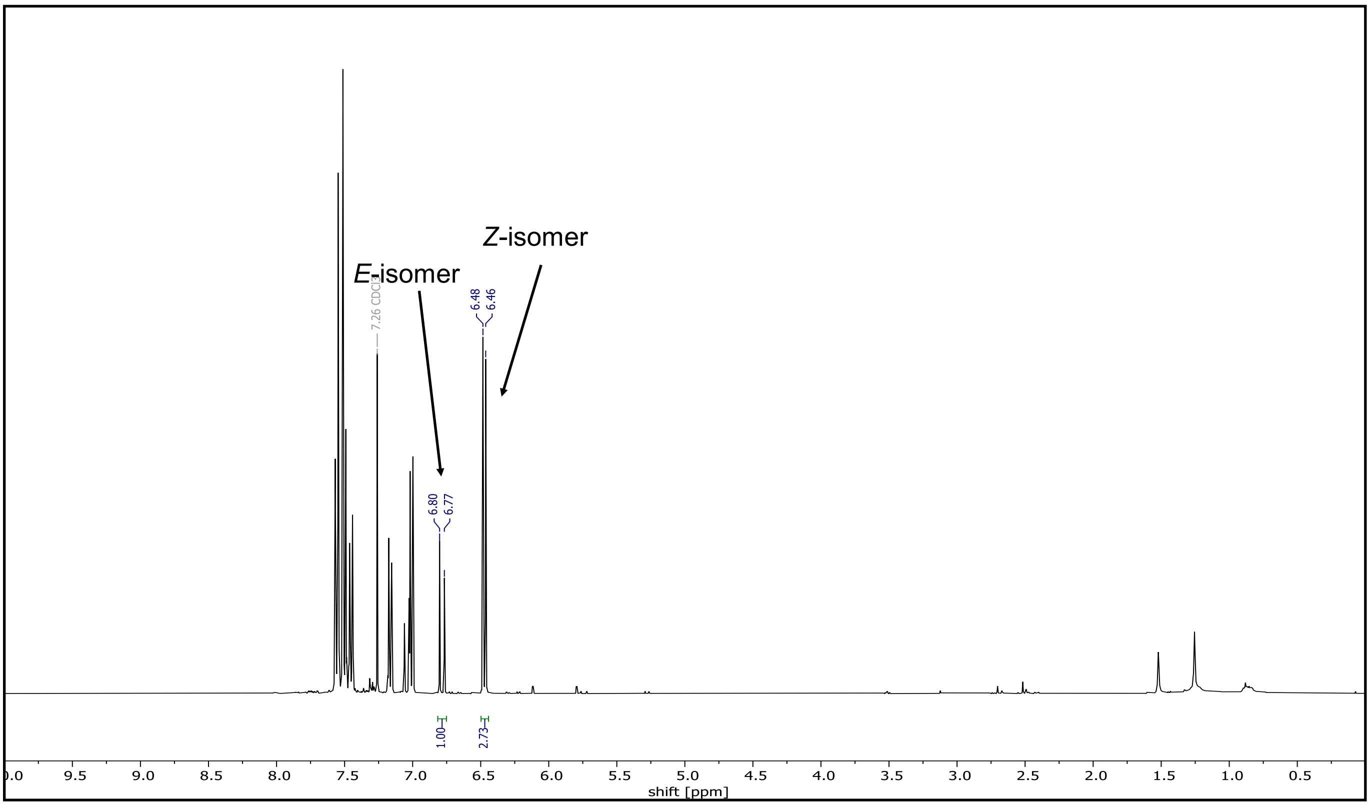

^{1}H NMR (400 MHz, $CDCl_3$), experiment 2

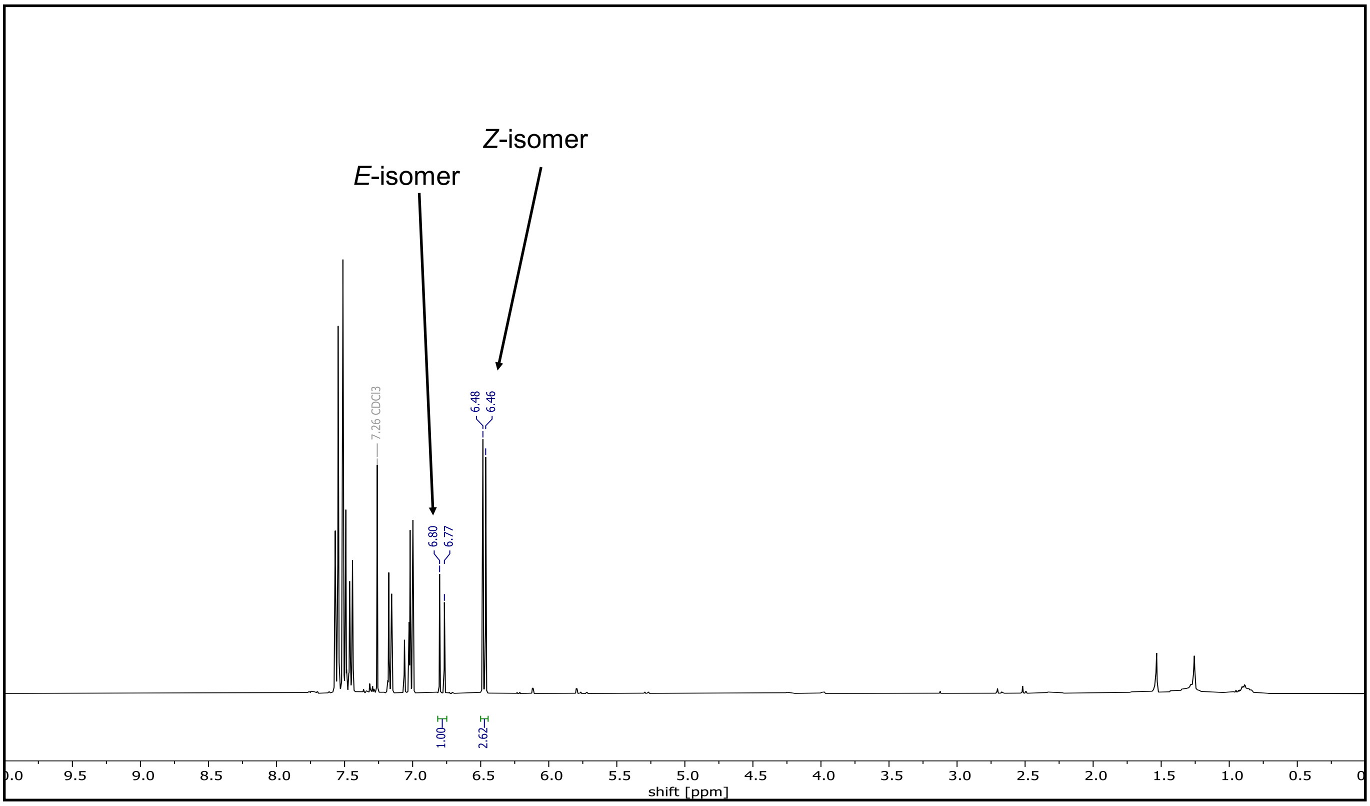

(*E*,*Z*)-1-Bromo-4-(2-vinyl)benzene (*E*,*Z*)-1

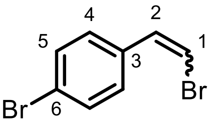

^{1}H NMR ($CDCl_3$), experiment 1

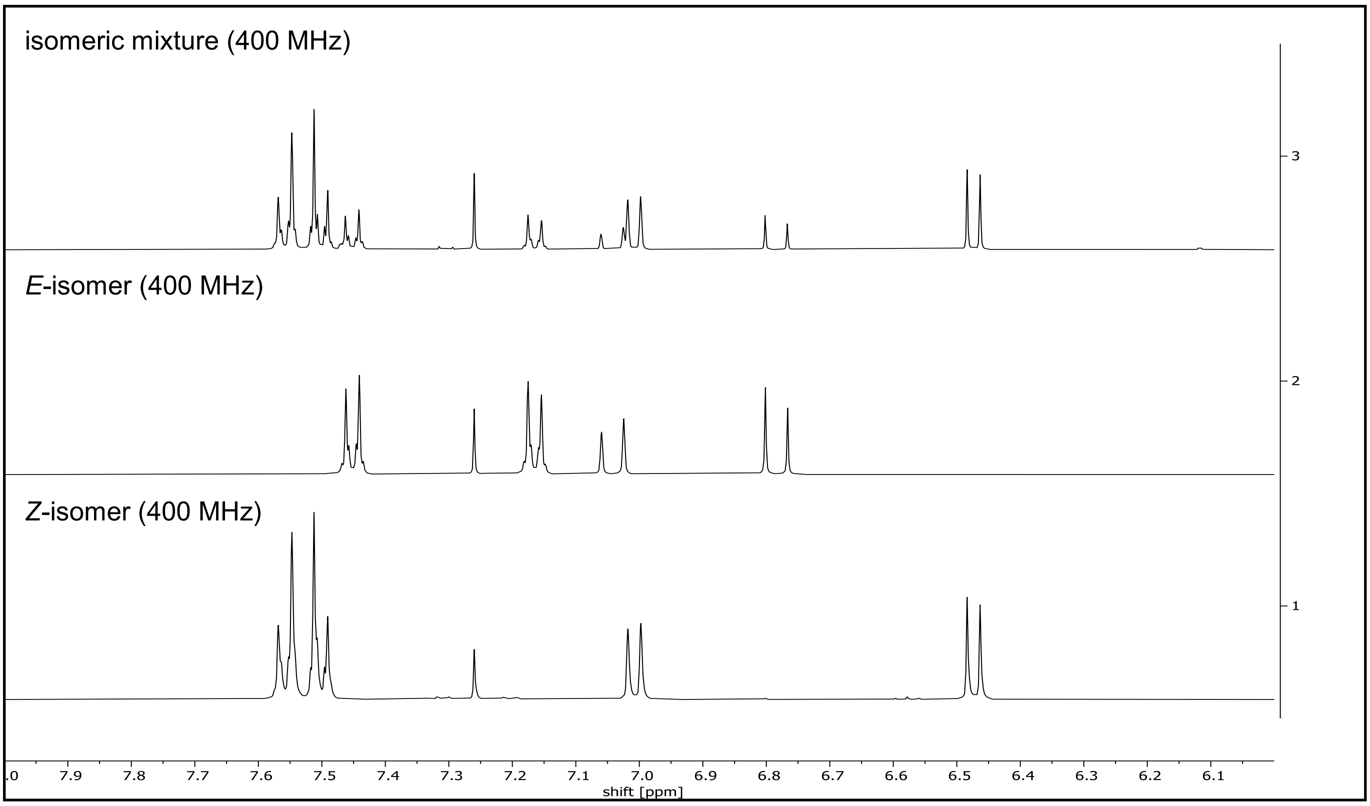

^{1}H NMR ($CDCl_3$), experiment 2

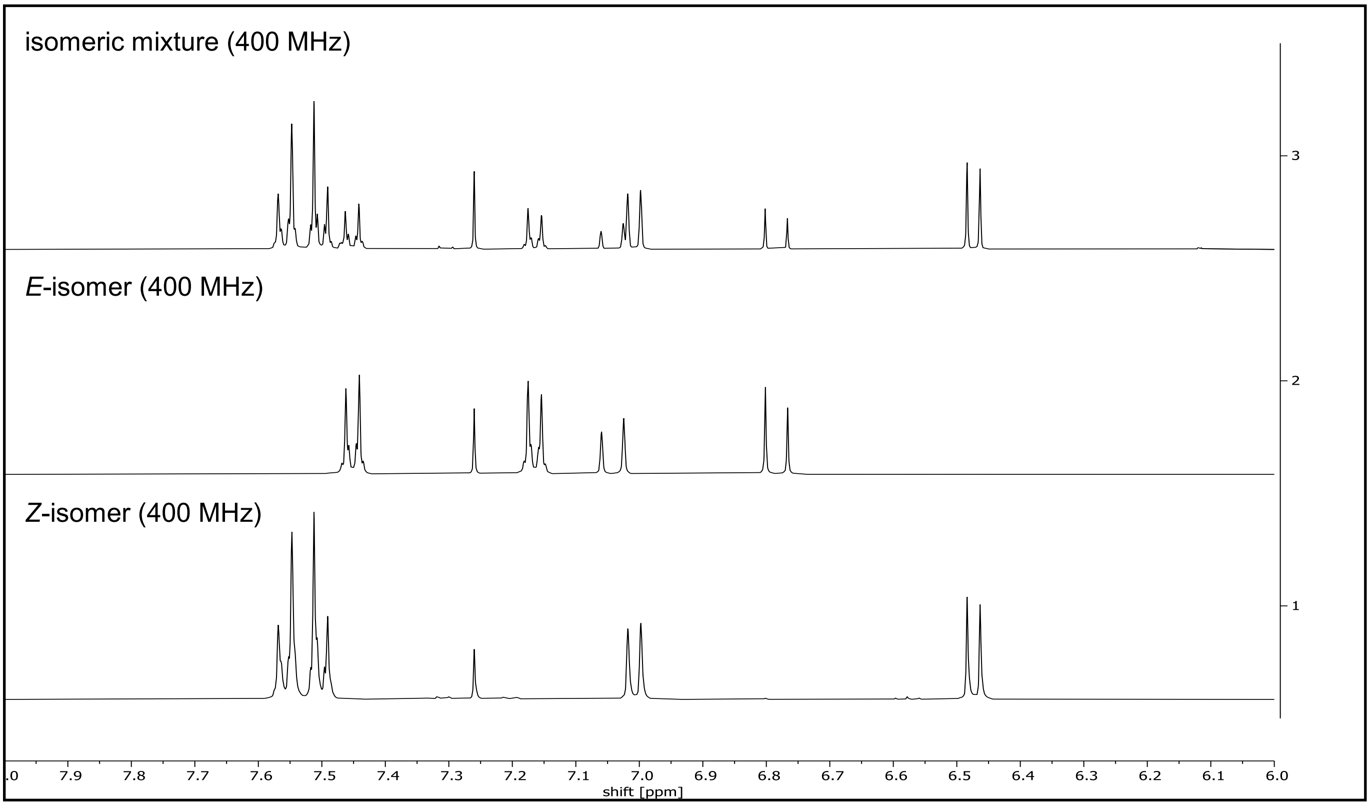

(*E*)-1-Bromo-4-(2-bromovinyl)benzene (*E*)-1

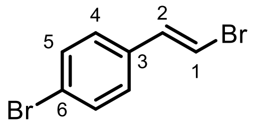

^{1}H NMR (400 MHz,$CDCl_3$)

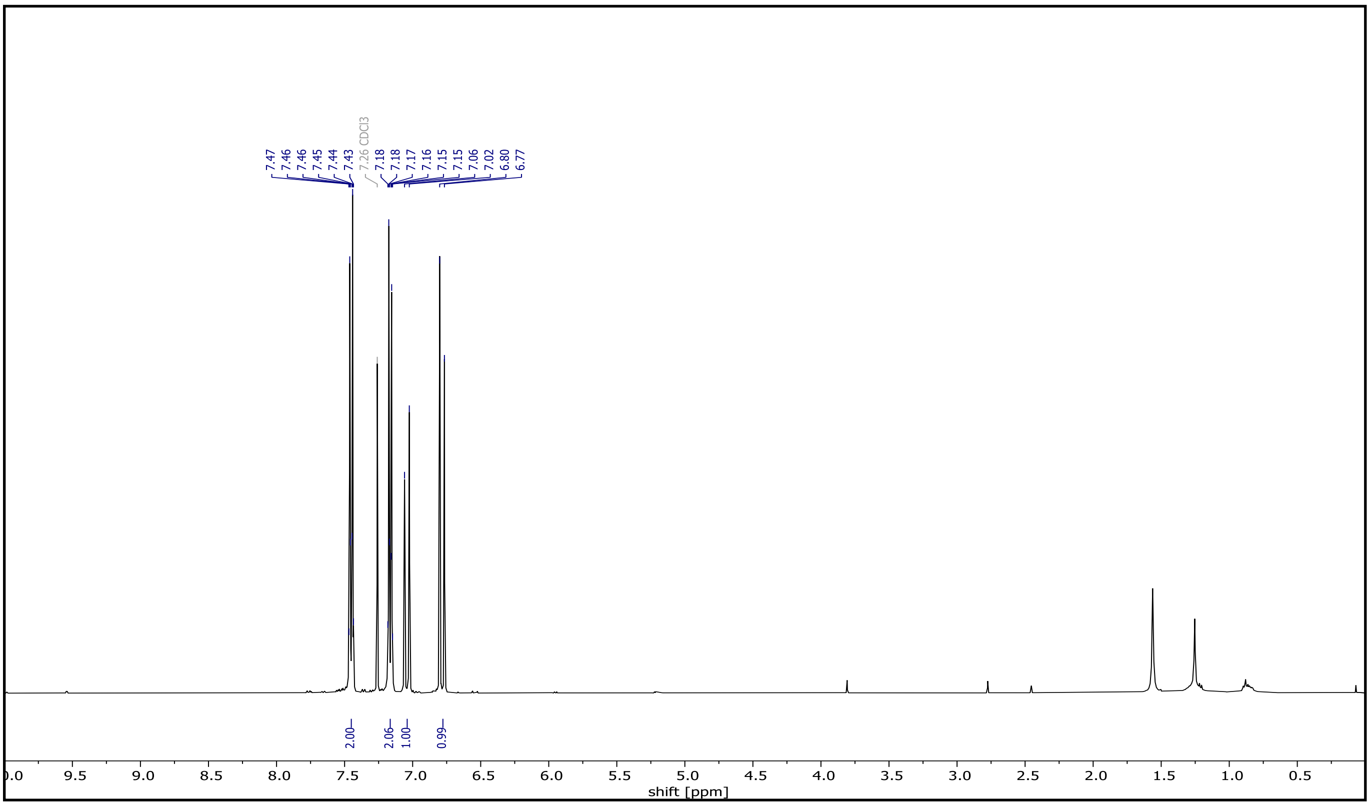

$^{13}C\{^{1}H\}$ NMR (101 MHz,$CDCl_3$)

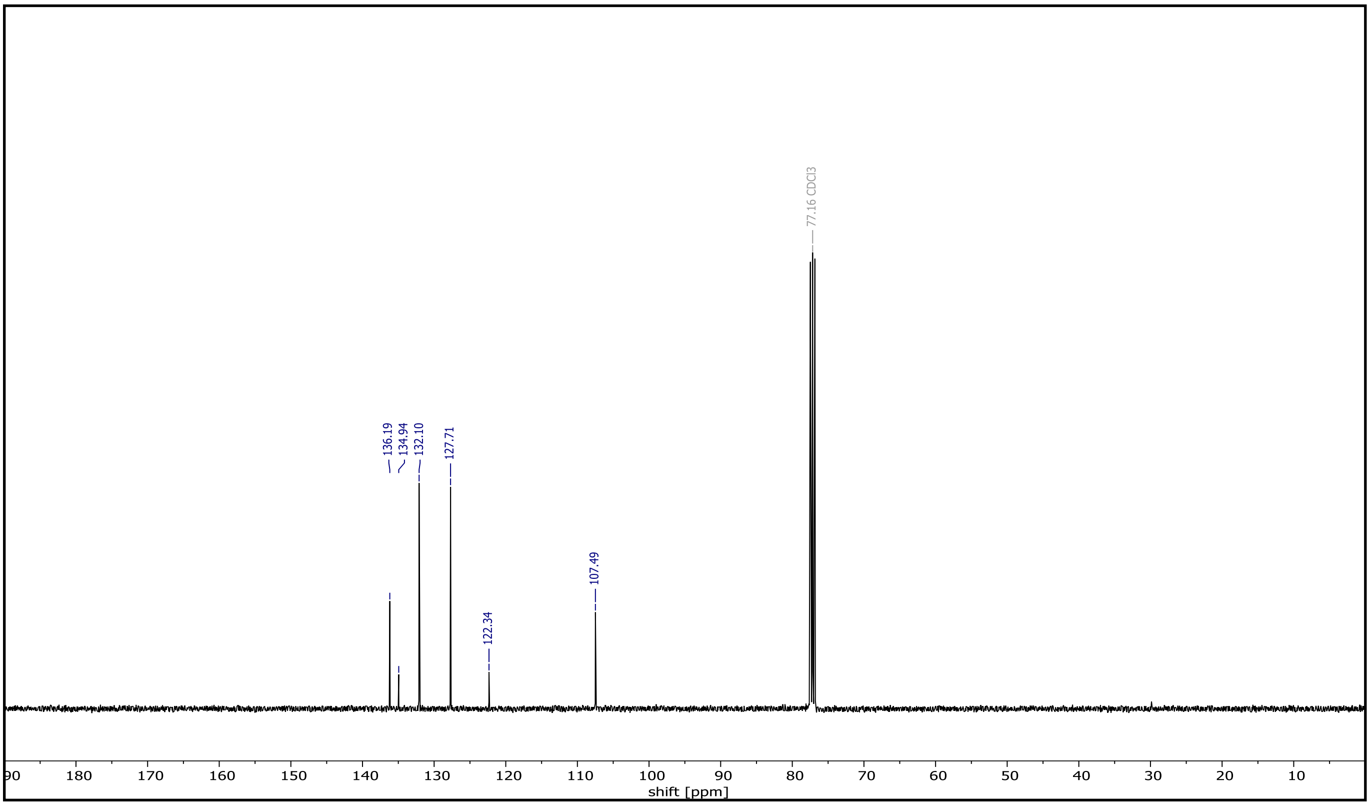

(Z)-1-Bromo-4-(2-bromovinyl)benzene (Z)-1

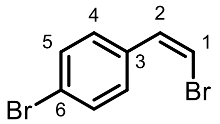

^{1}H NMR (400 MHz,$CDCl_3$)

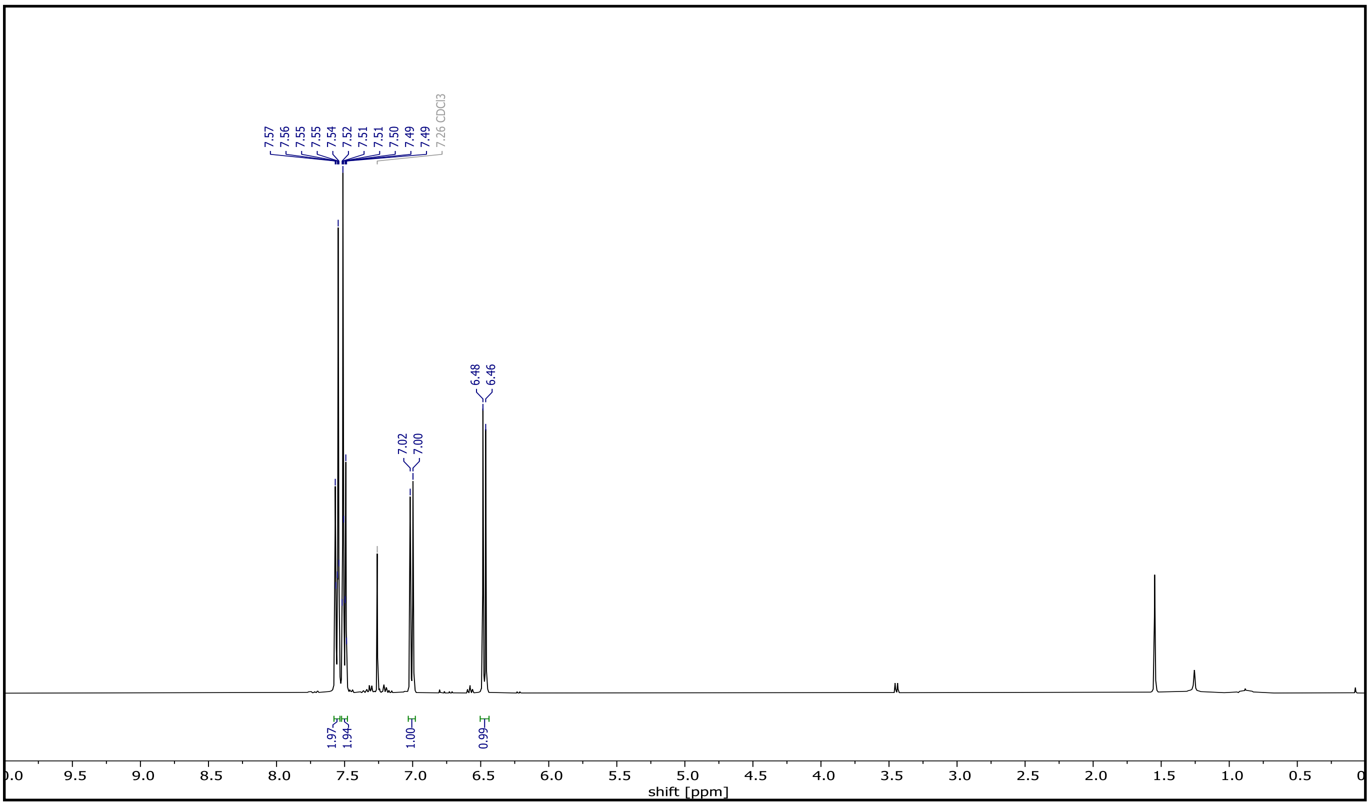

^{13}C{^{1}H} NMR (101 MHz,$CDCl_3$)

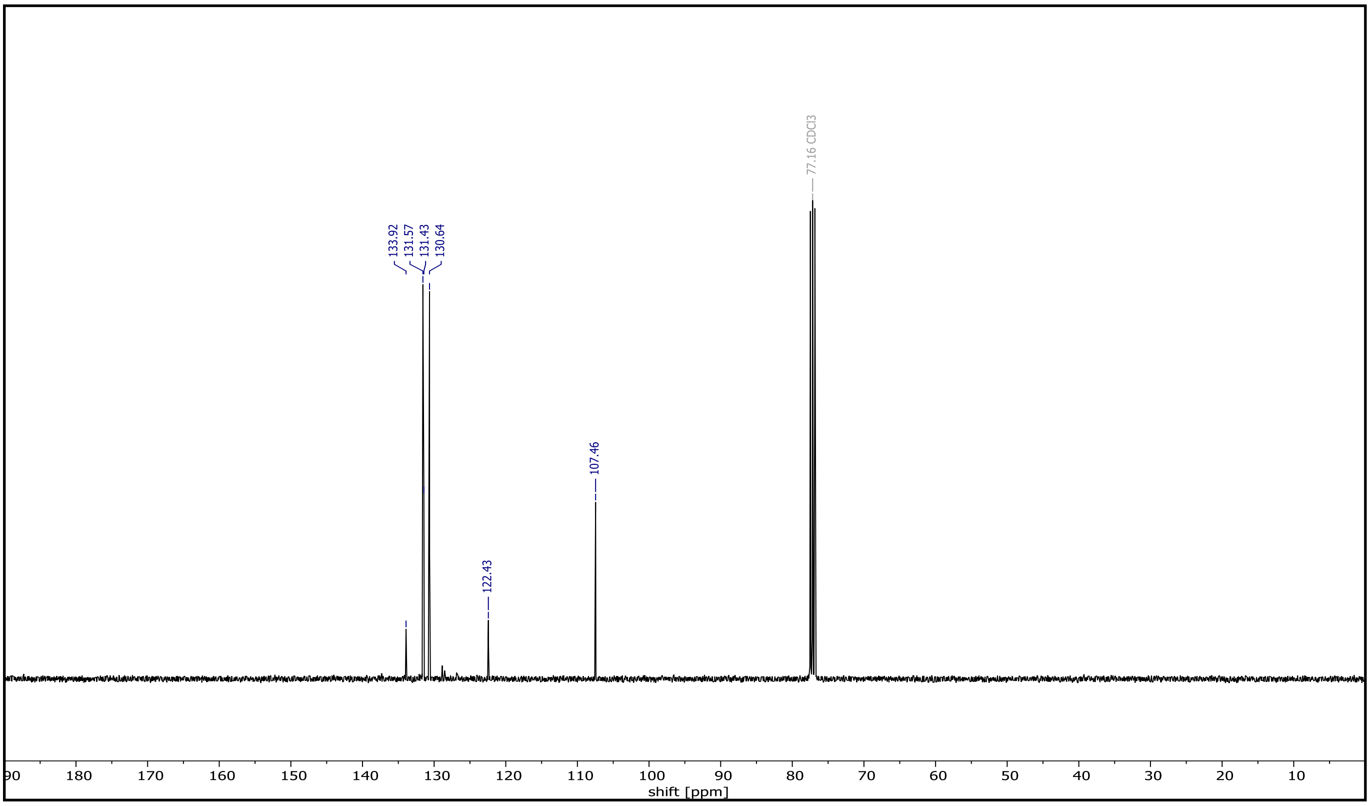

(*E*,*Z*)-1-(2-Bromovinyl)-4-fluorobenzene (*E*,*Z*)-2

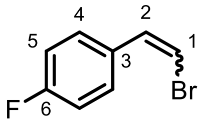

^{1}H NMR (400 MHz, $CDCl_3$), experiment 1

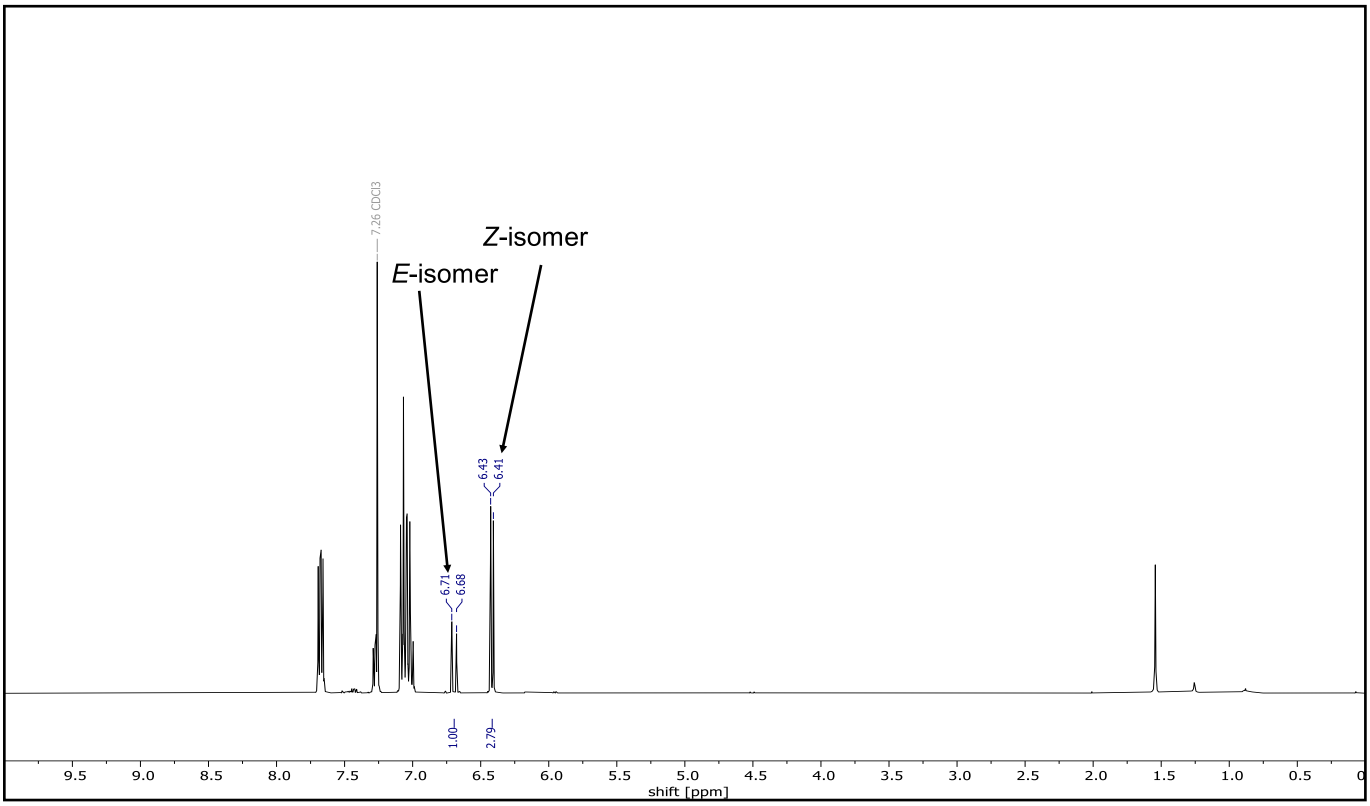

^{1}H NMR (400 MHz, $CDCl_3$), experiment 2

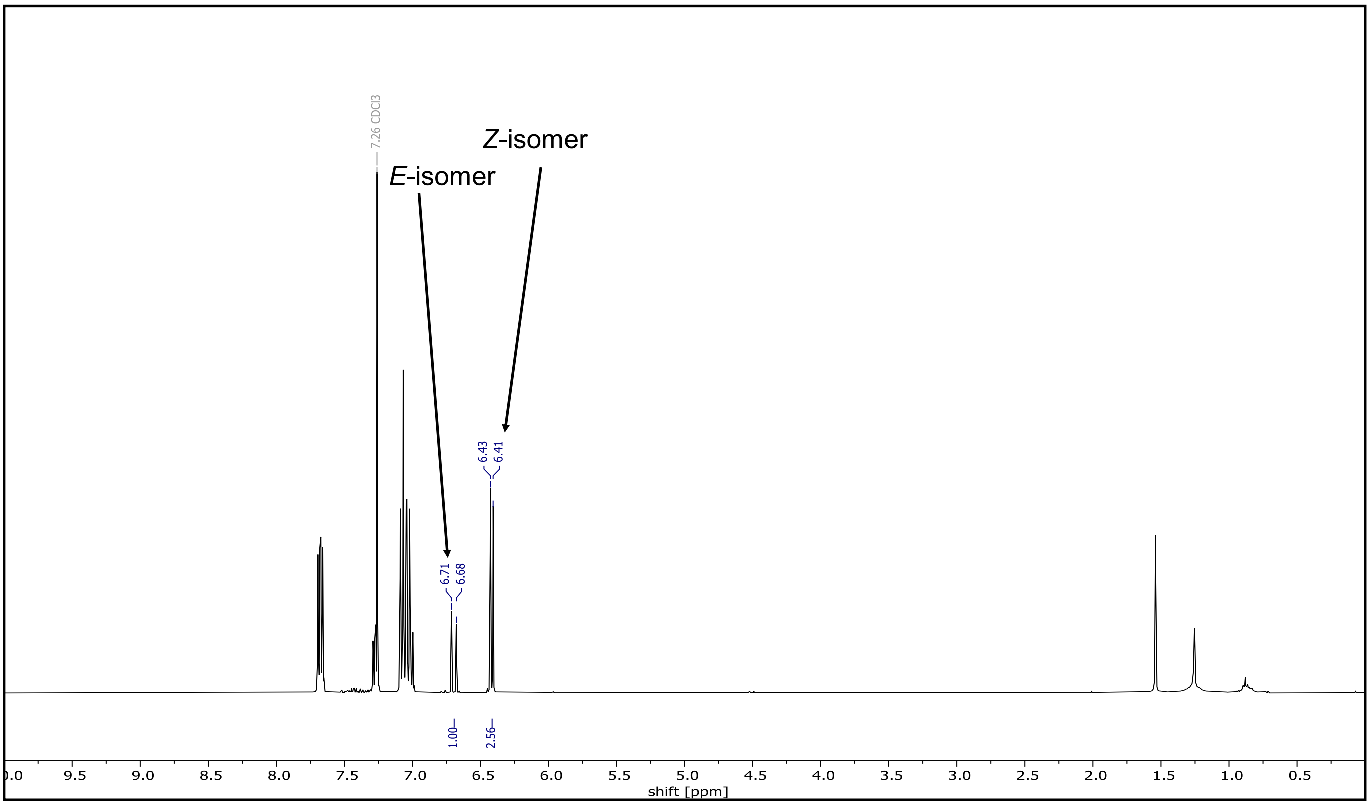

(*E*,*Z*)-1-(2-Bromovinyl)-4-fluorobenzene (*E*,*Z*)-2

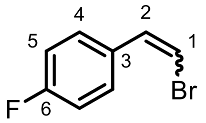

^{1}H NMR ($CDCl_3$), experiment 1

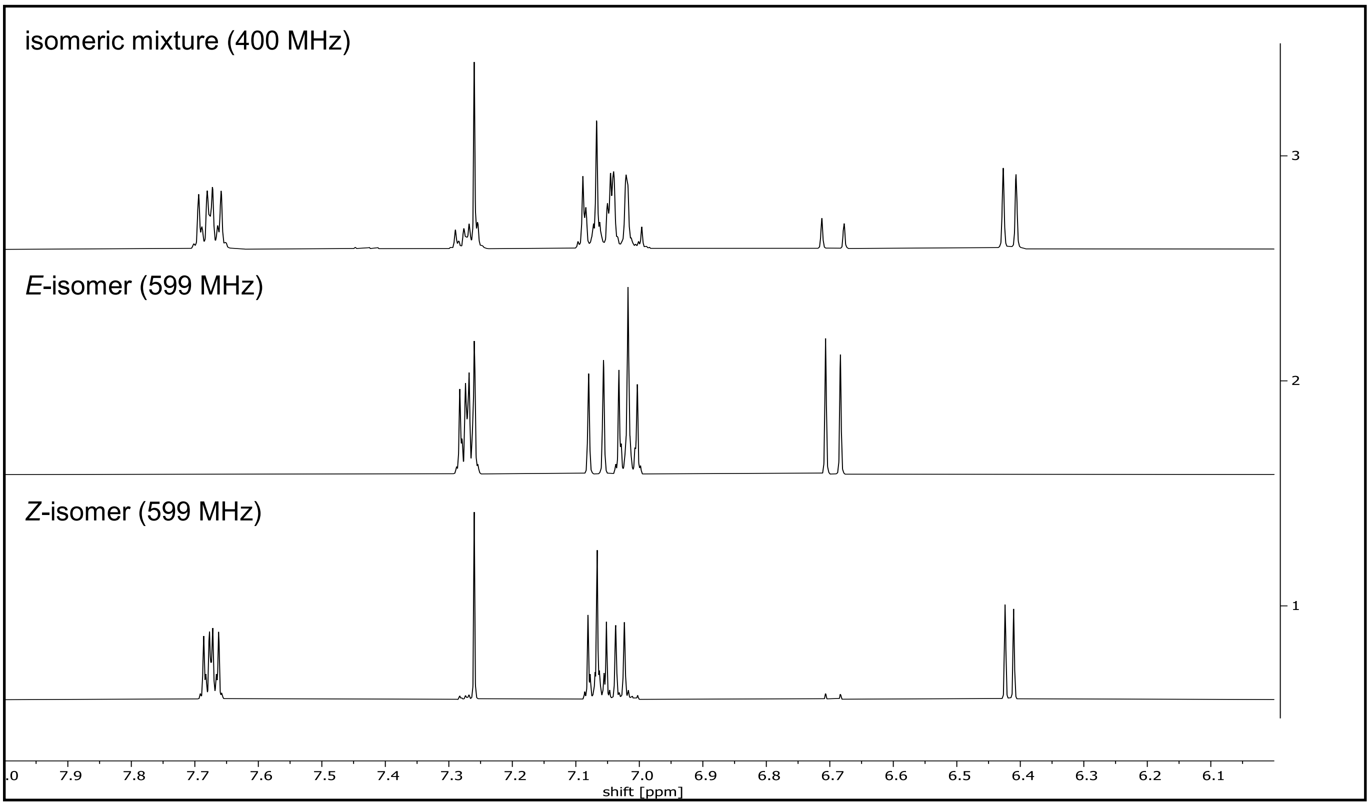

^{1}H NMR ($CDCl_3$), experiment 2

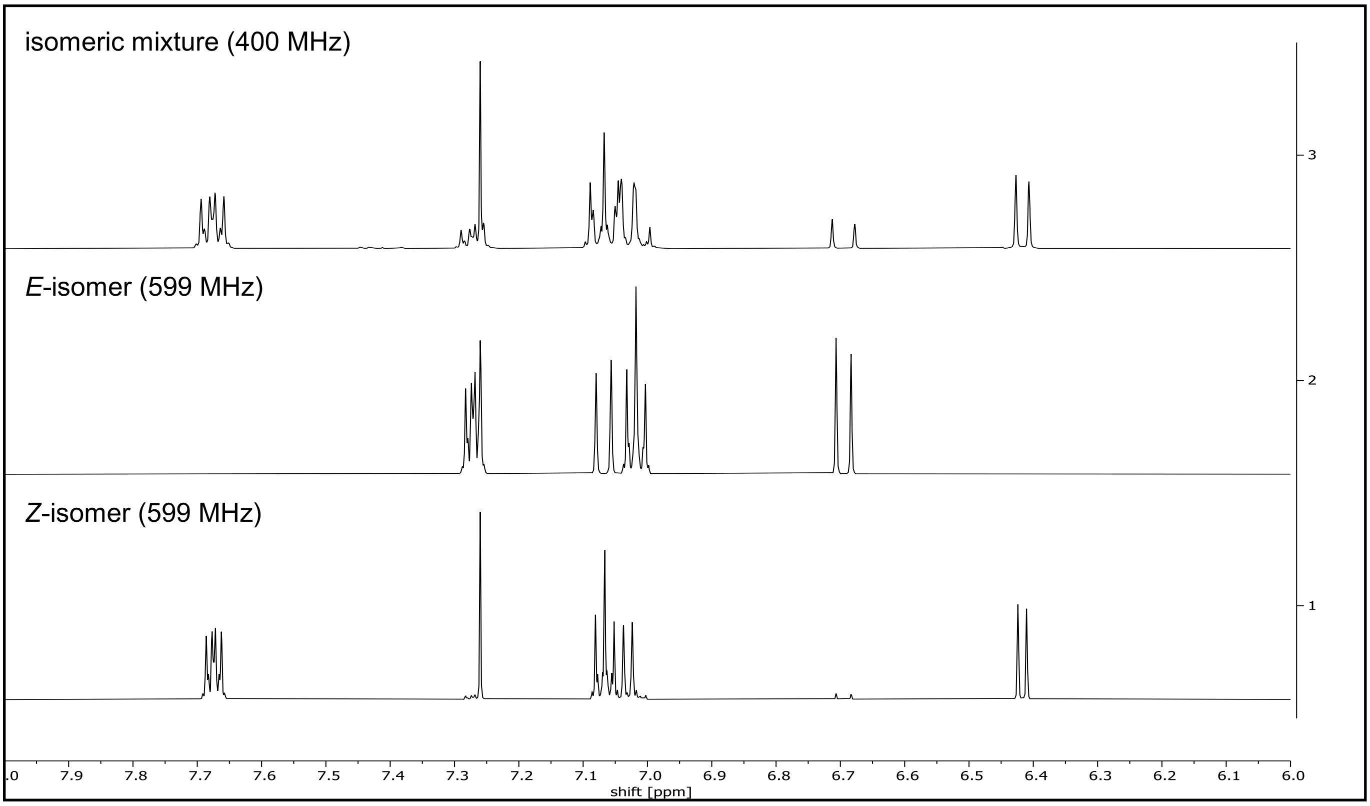

(*E*)-1-(2-Bromovinyl)-4-fluorobenzene (*E*)-2

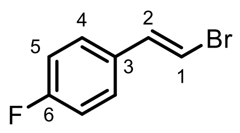

^{1}H NMR (599 MHz, $CDCl_3$)

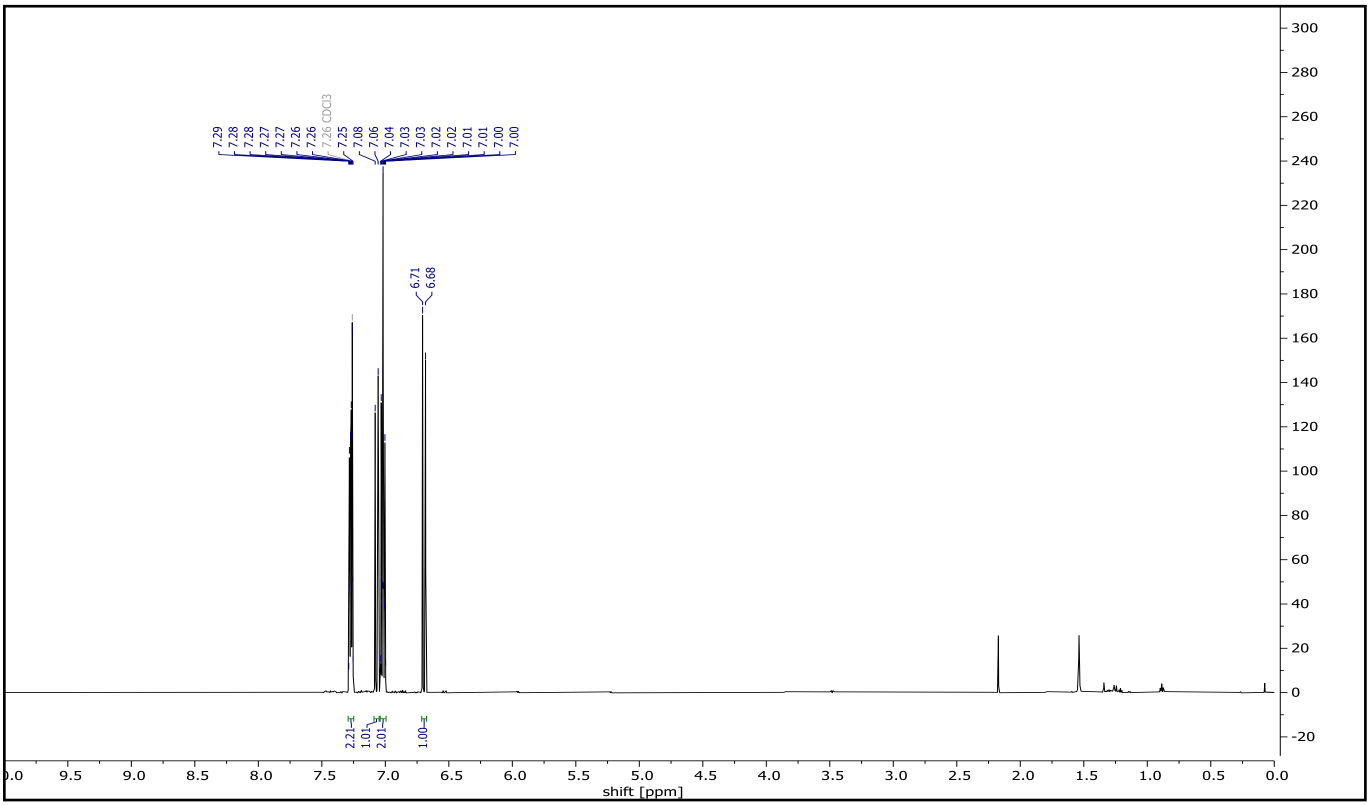

^{13}C{^{1}H} NMR (151 MHz, $CDCl_3$)

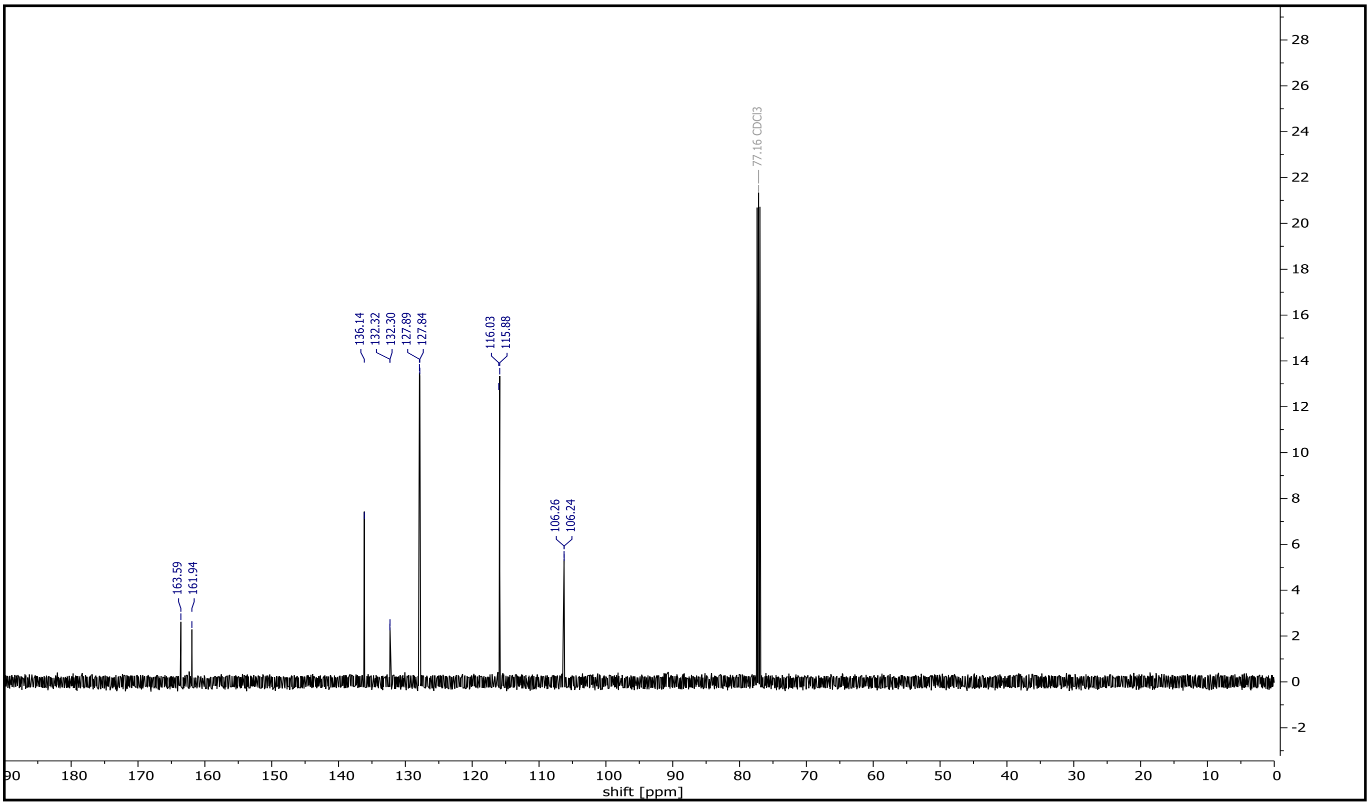

(*E*)-1-(2-Bromovinyl)-4-fluorobenzene (*E*)-2

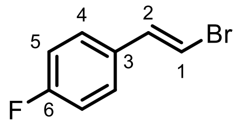

^{19}F NMR (564 MHz, $CDCl_3$)

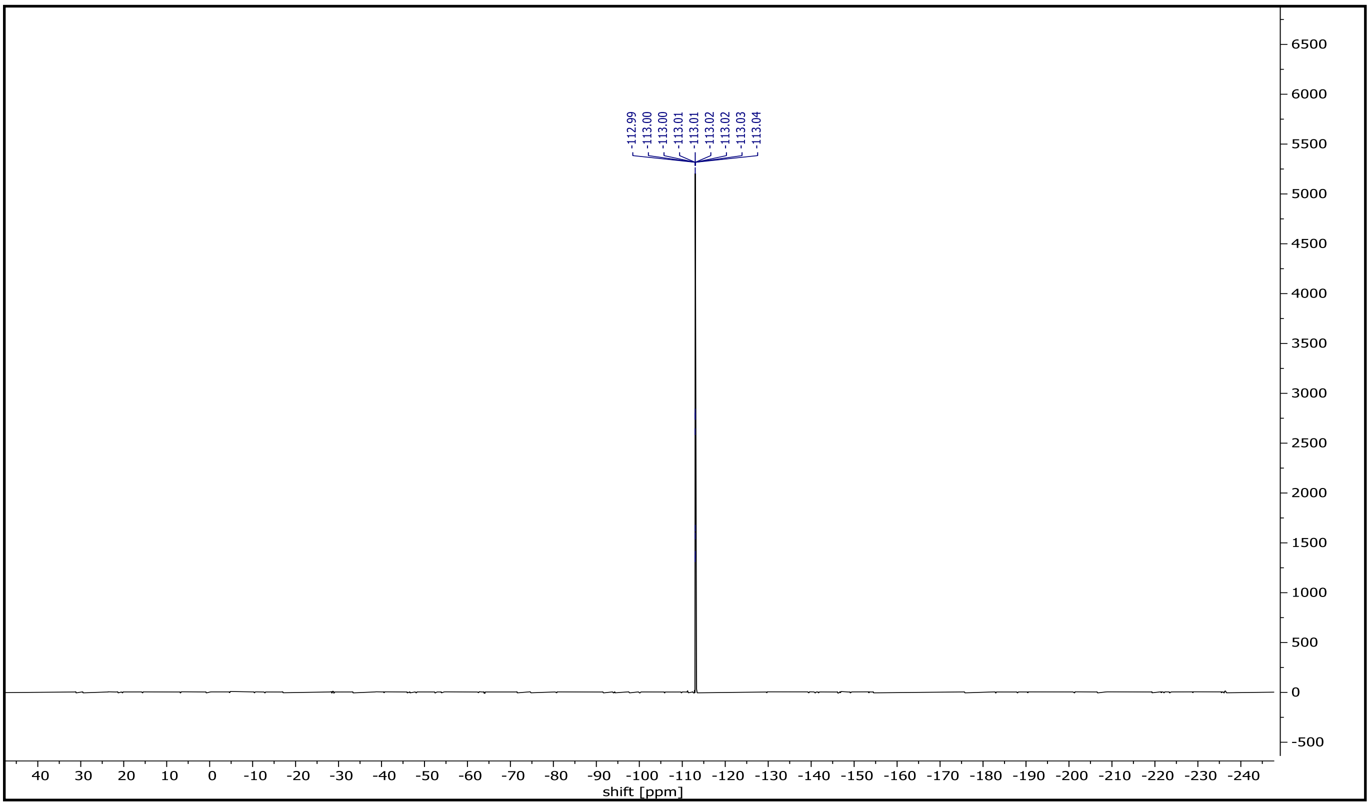

(*Z*)-1-(2-Bromovinyl)-4-fluorobenzene (*Z*)-2

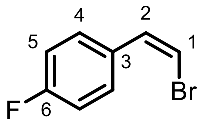

^{1}H NMR (599 MHz, $CDCl_3$)

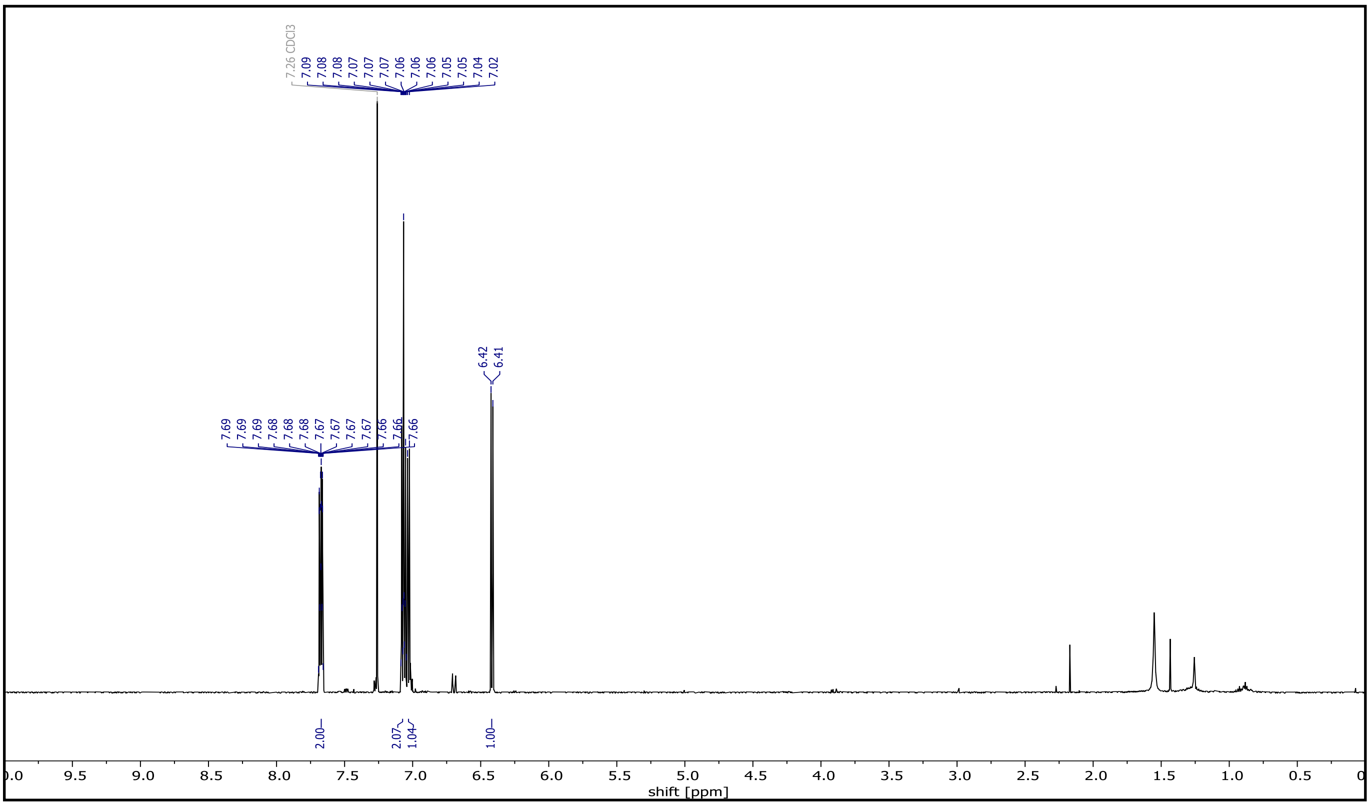

^{13}C{^{1}H} NMR (151 MHz, $CDCl_3$)

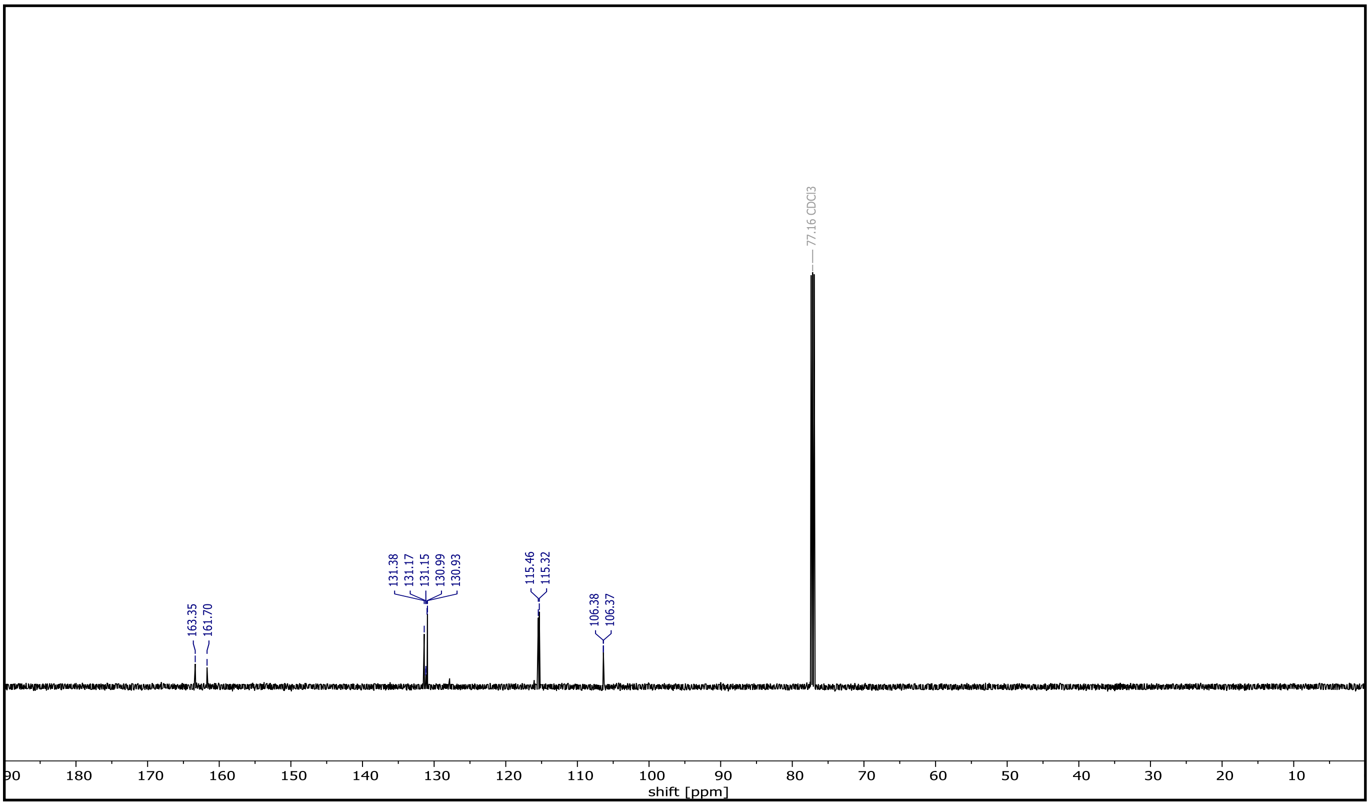

(*Z*)-1-(2-Bromovinyl)-4-fluorobenzene (*Z*)-2

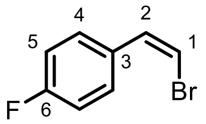

^{19}F NMR (376 MHz, $CDCl_3$)

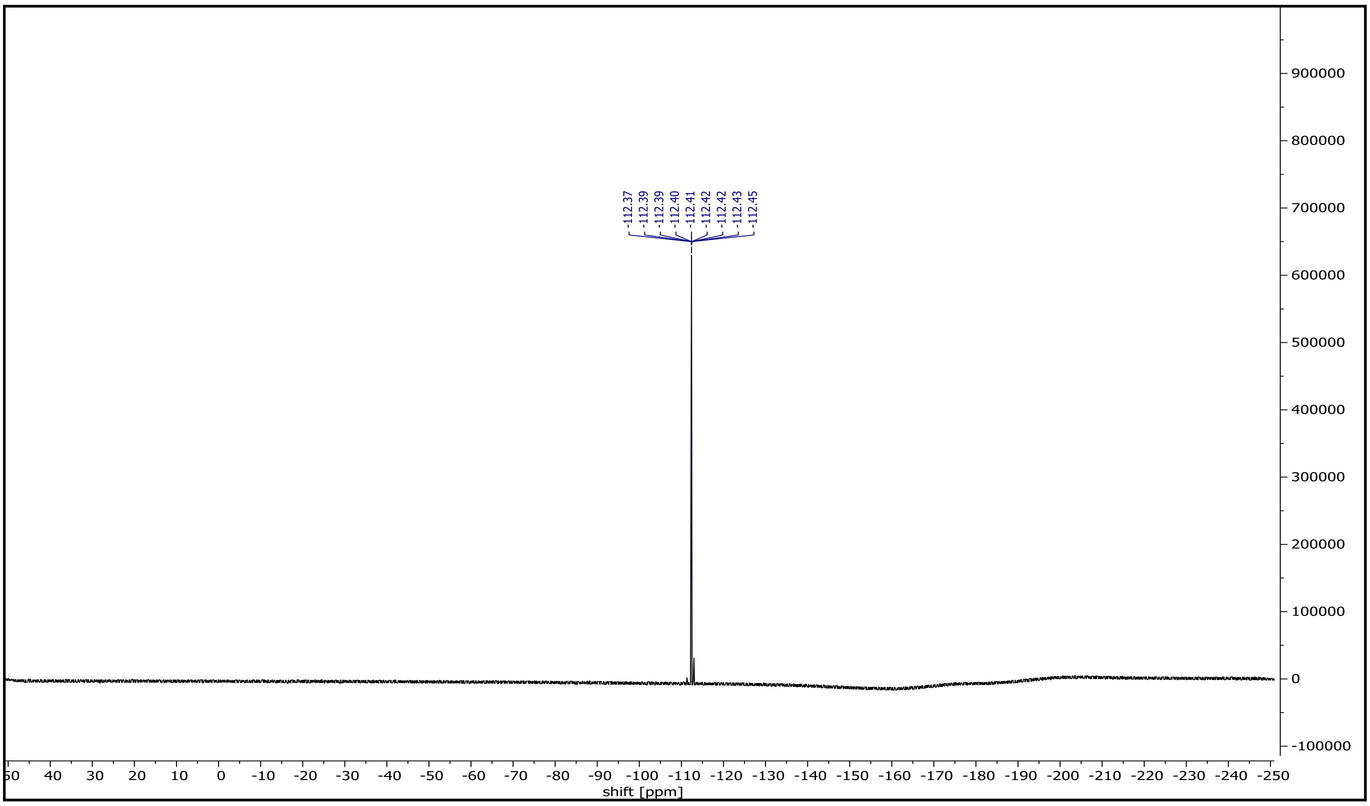

(*E*,*Z*)-1-(2-Bromovinyl)-4-chlorobenzene (*E*,*Z*)-3

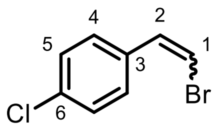

^{1}H NMR (400 MHz, $CDCl_3$), experiment 1

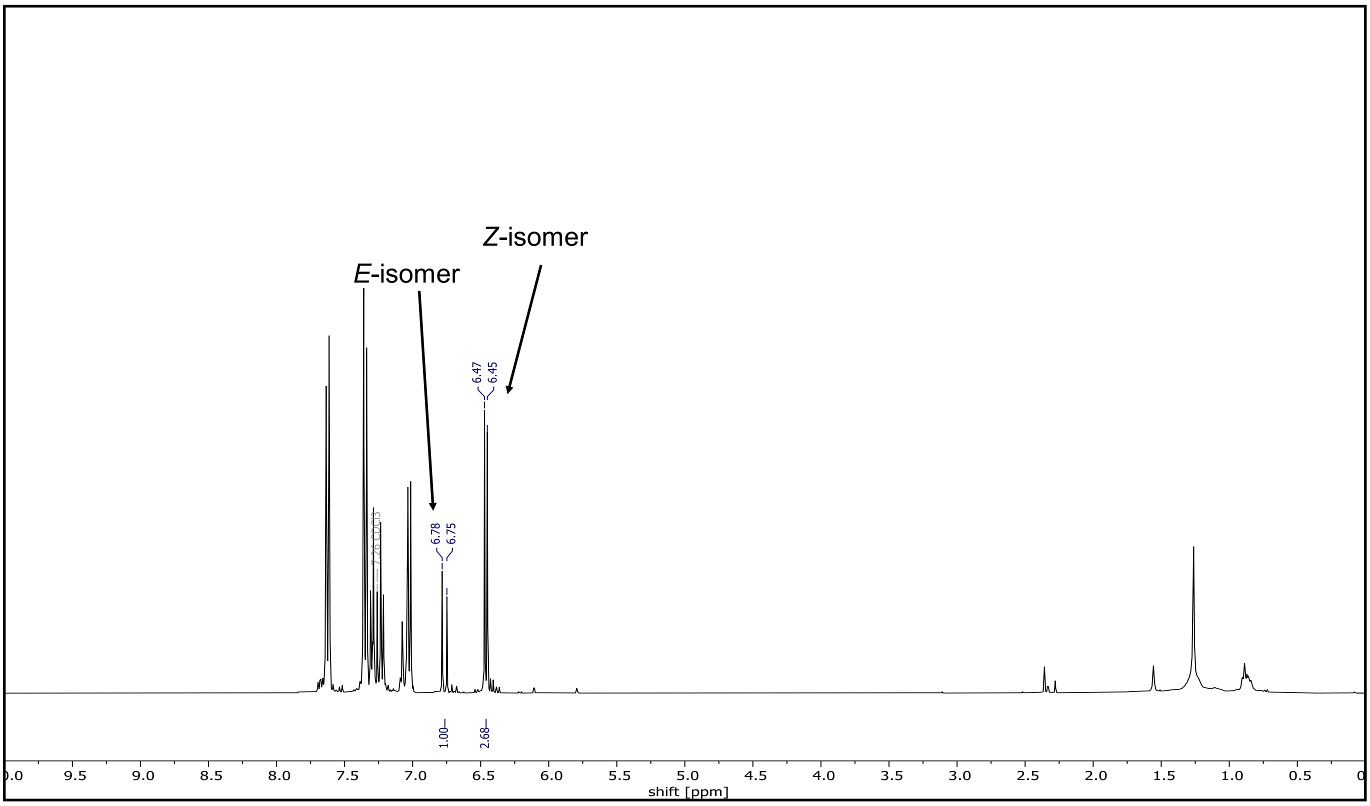

^{1}H NMR (400 MHz, $CDCl_3$), experiment 2

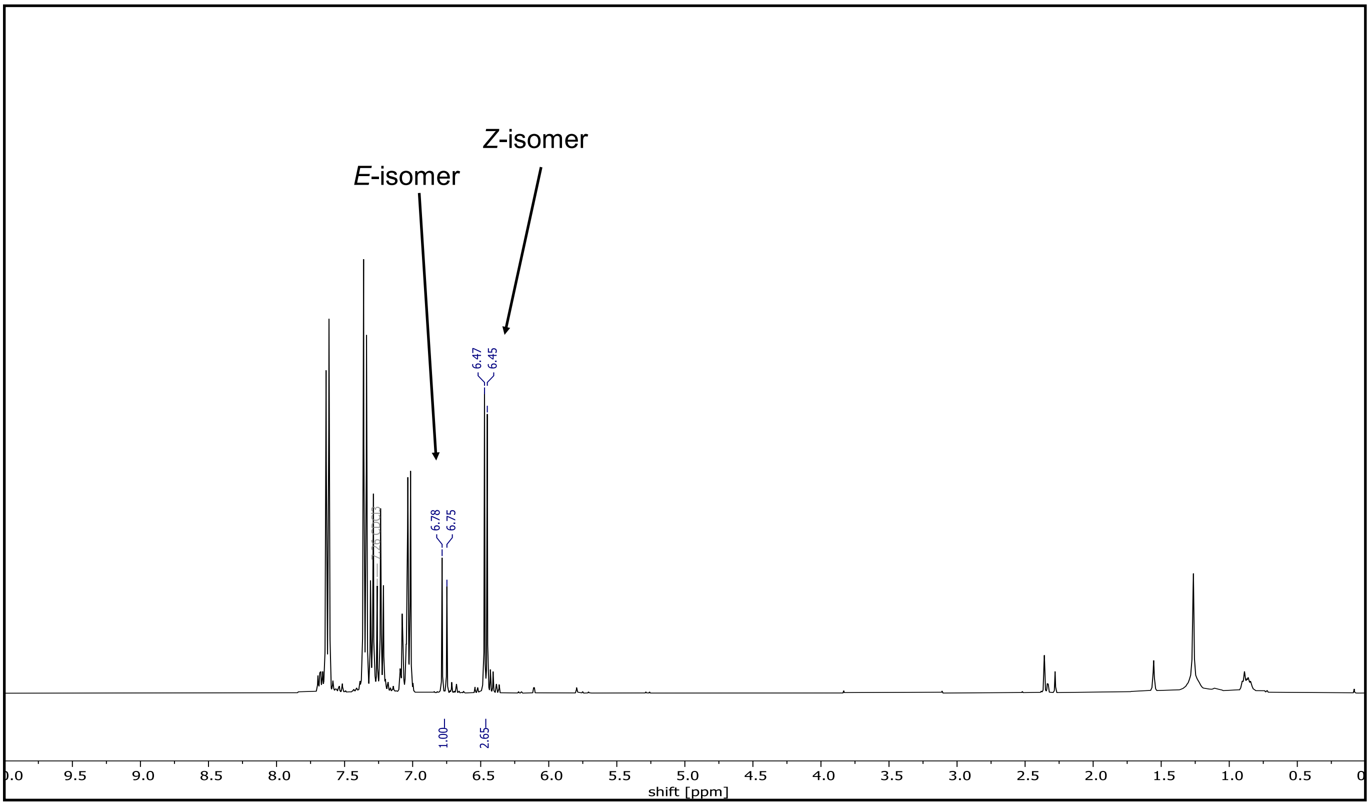

(*E*,*Z*)-1-(2-Bromovinyl)-4-chlorobenzene (*E*,*Z*)-3

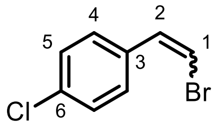

^{1}H NMR ($CDCl_3$), experiment1

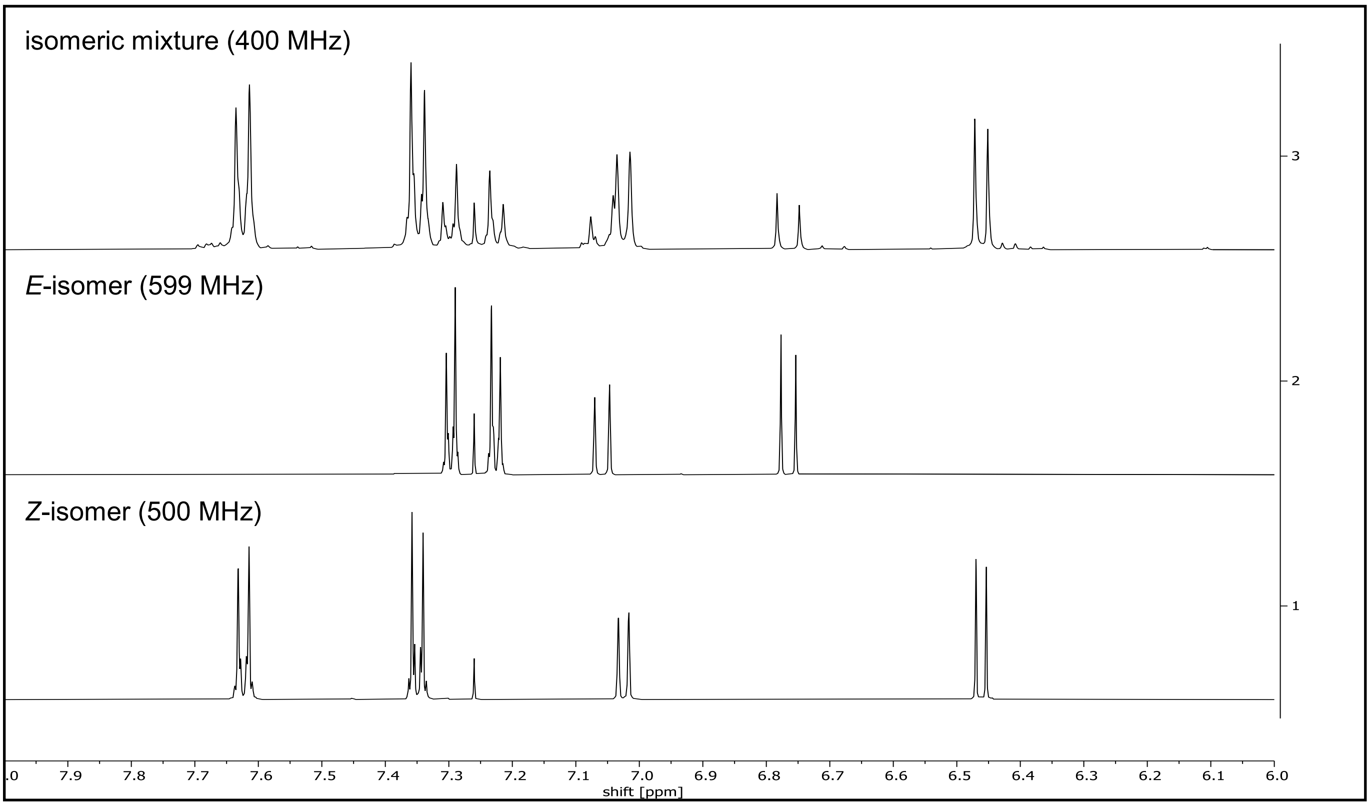

^{1}H NMR ($CDCl_3$), experiment 2

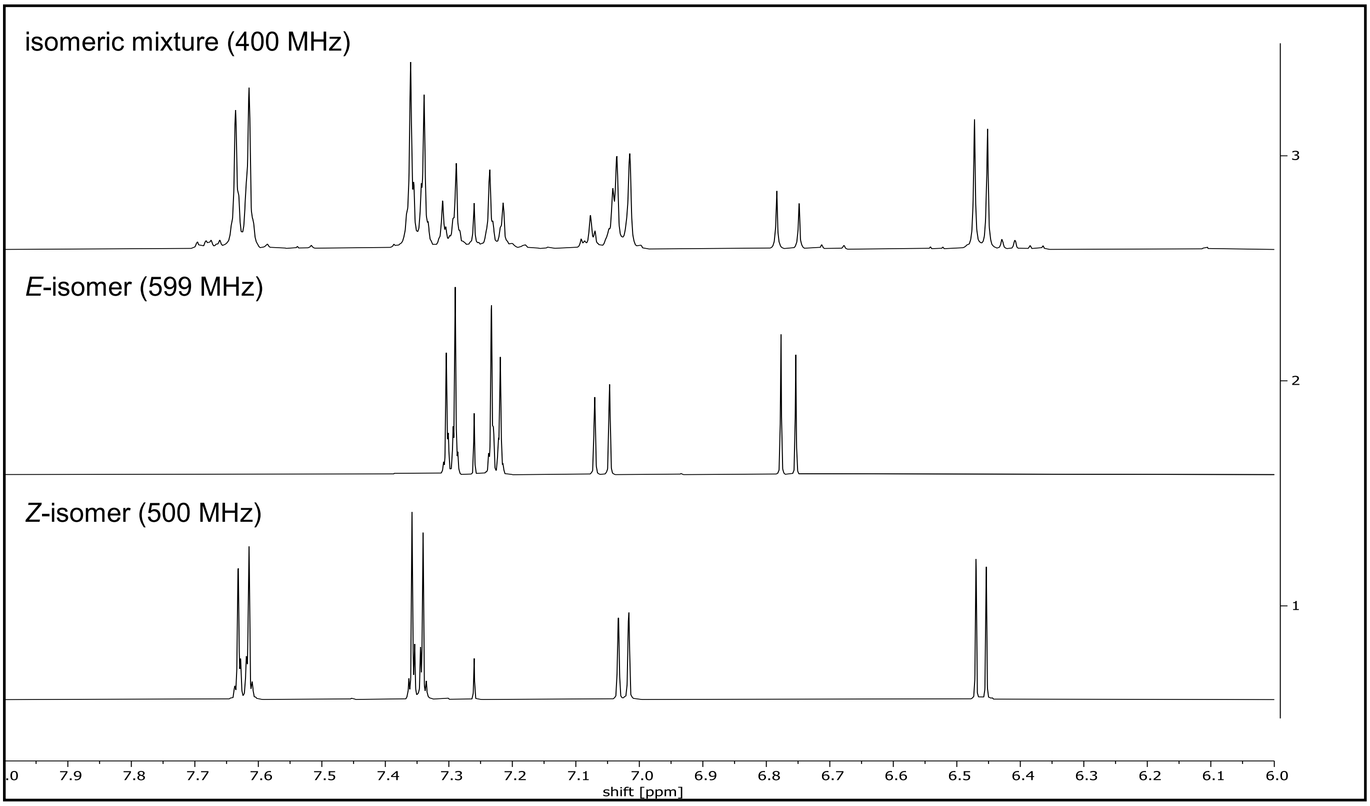

(*E*)-1-(2-Bromovinyl)-4-chlorobenzene (*E*)-3

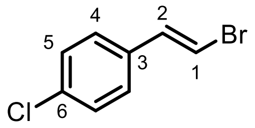

^{1}H NMR (599 MHz, $CDCl_3$)

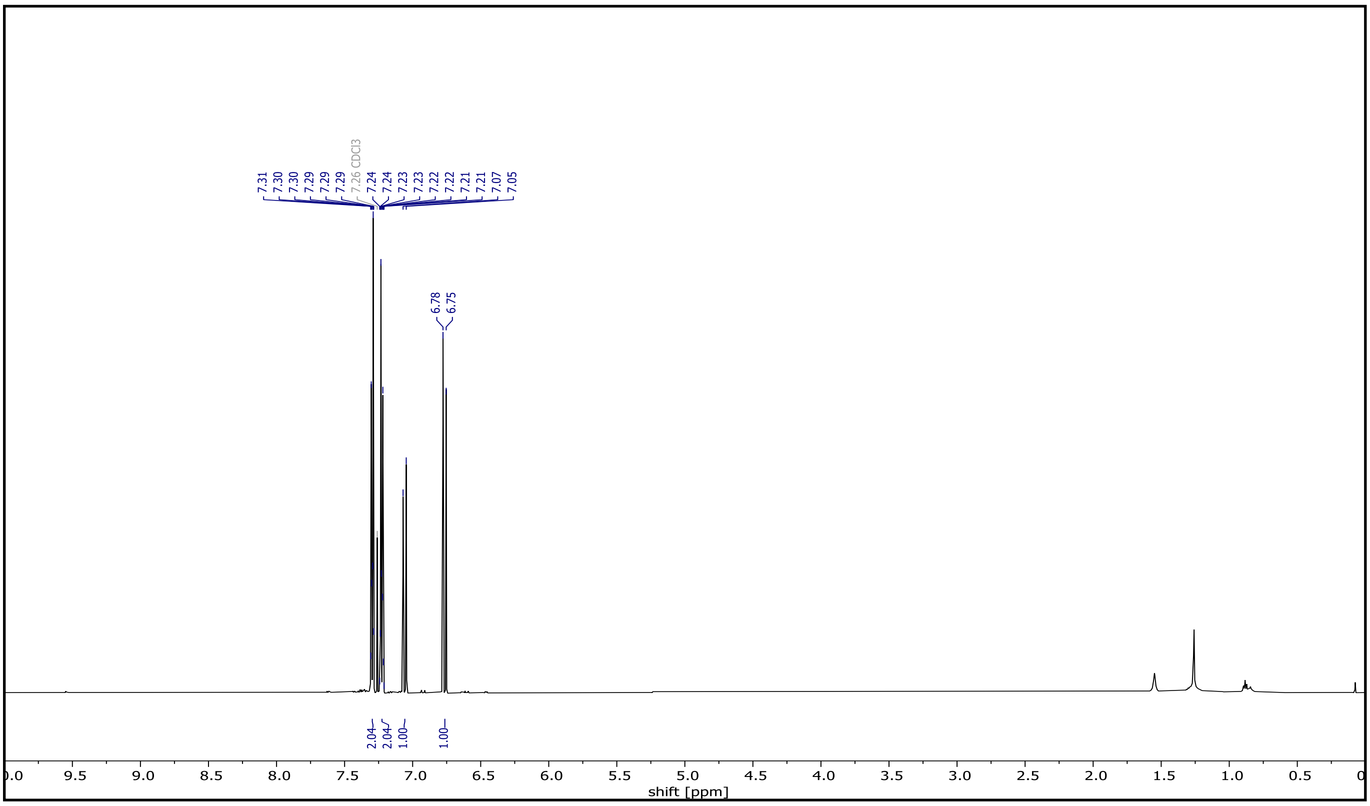

$^{13}C\{^1H\}$ NMR (151 MHz, $CDCl_3$)

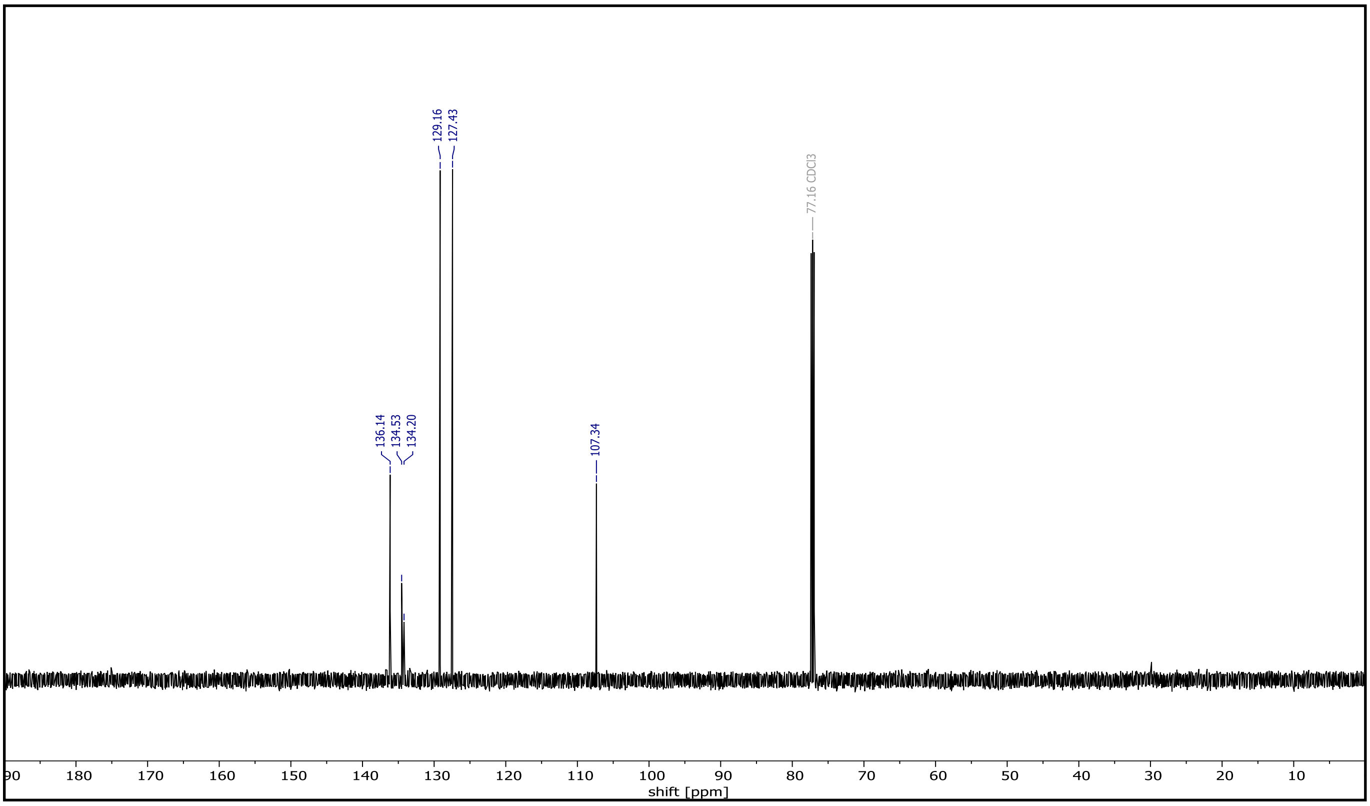

(*Z*)-1-(2-Bromovinyl)-4-chlorobenzene (*Z*)-3

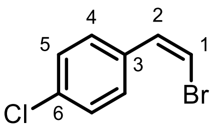

^{1}H NMR (500 MHz, $CDCl_3$)

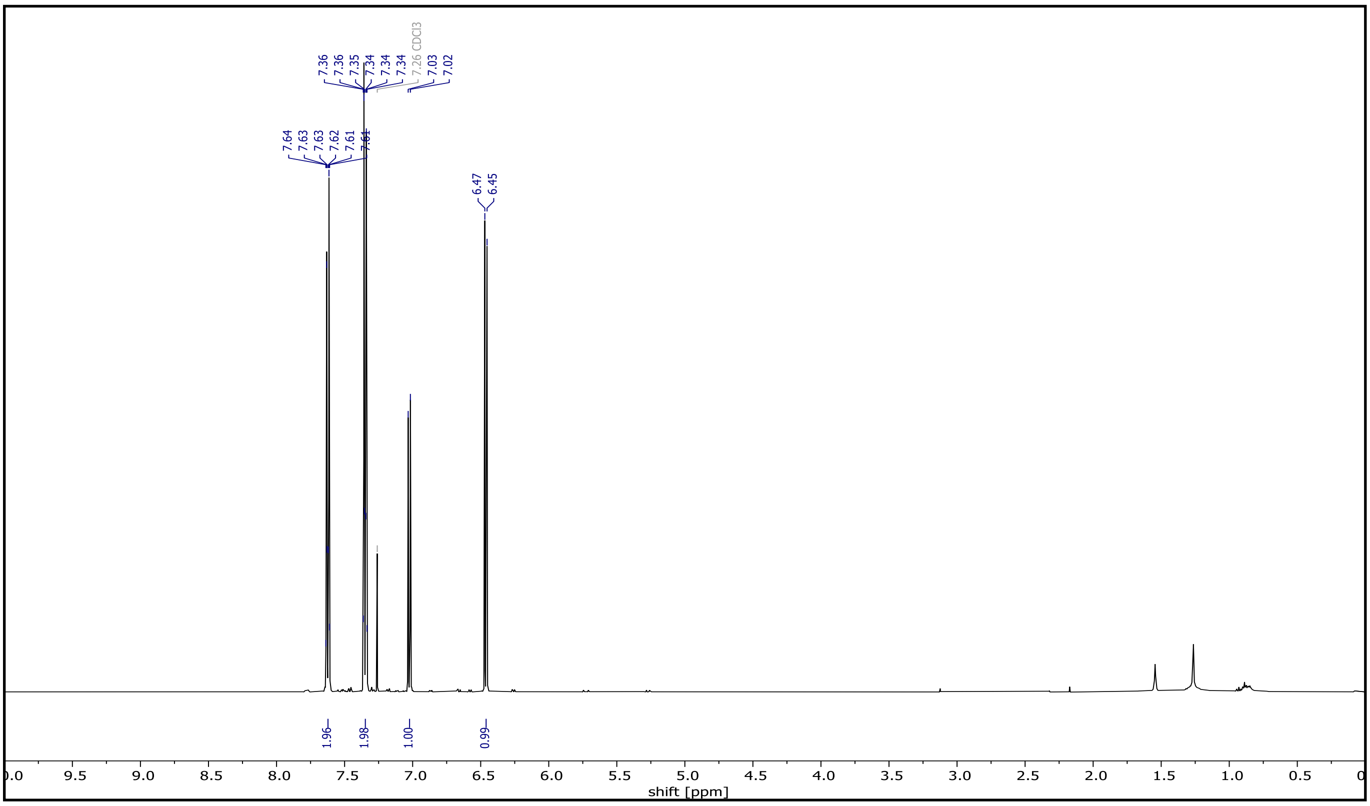

$^{13}C\{^{1}H\}$ NMR (126 MHz, $CDCl_3$)

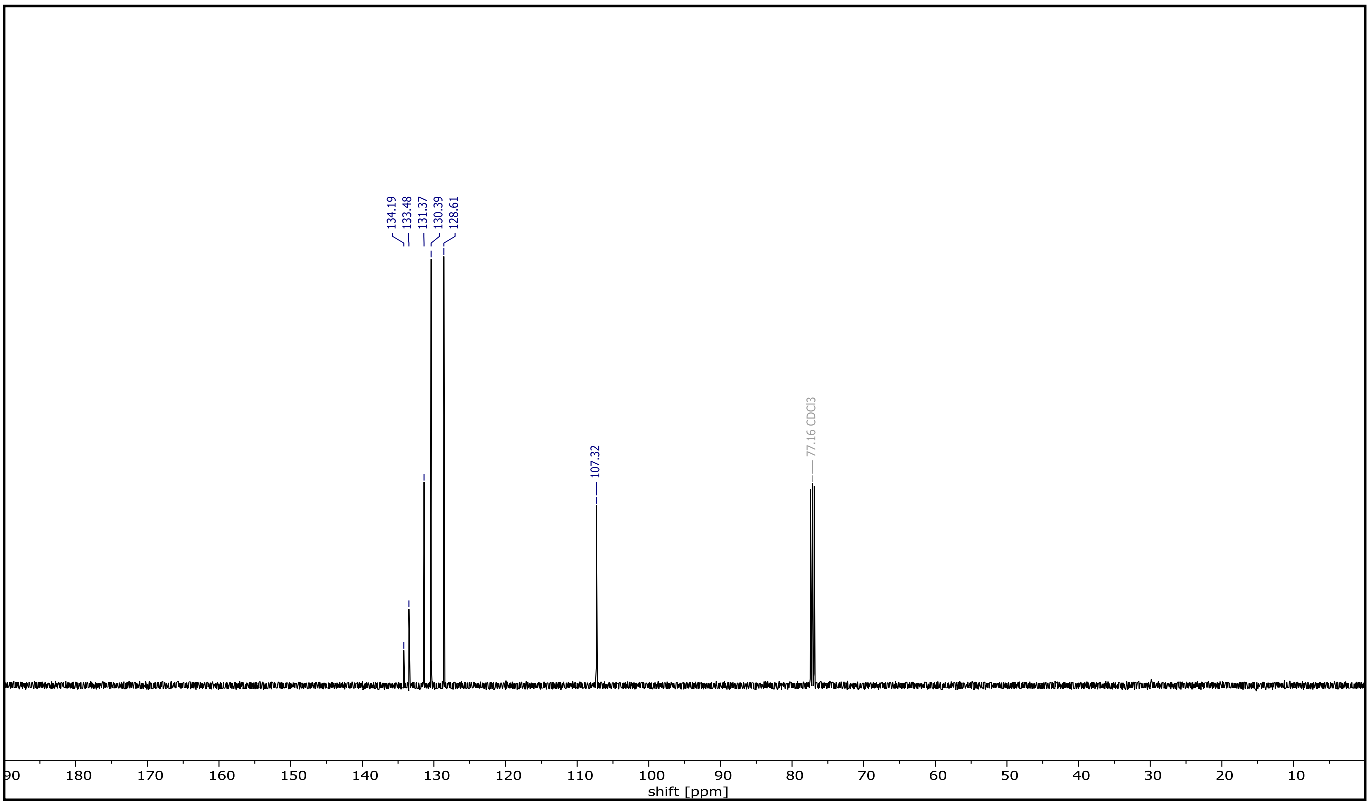

(*E*,*Z*)-1-(2-Bromovinyl)-4-iodobenzene (*E*,*Z*)-4

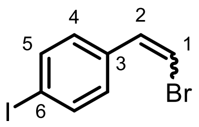

^{1}H NMR (400 MHz, $CDCl_3$), experiment 1

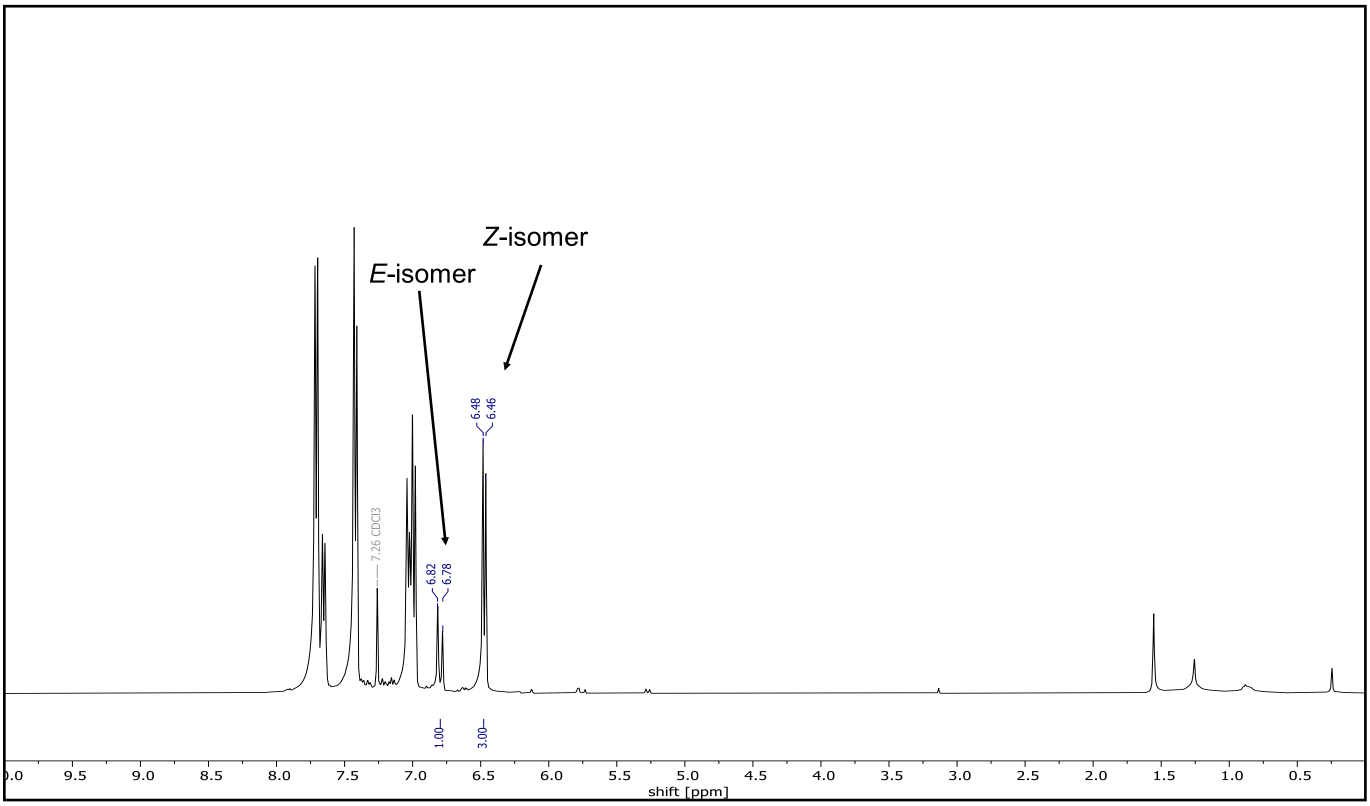

^{1}H NMR (400 MHz, $CDCl_3$), experiment 2

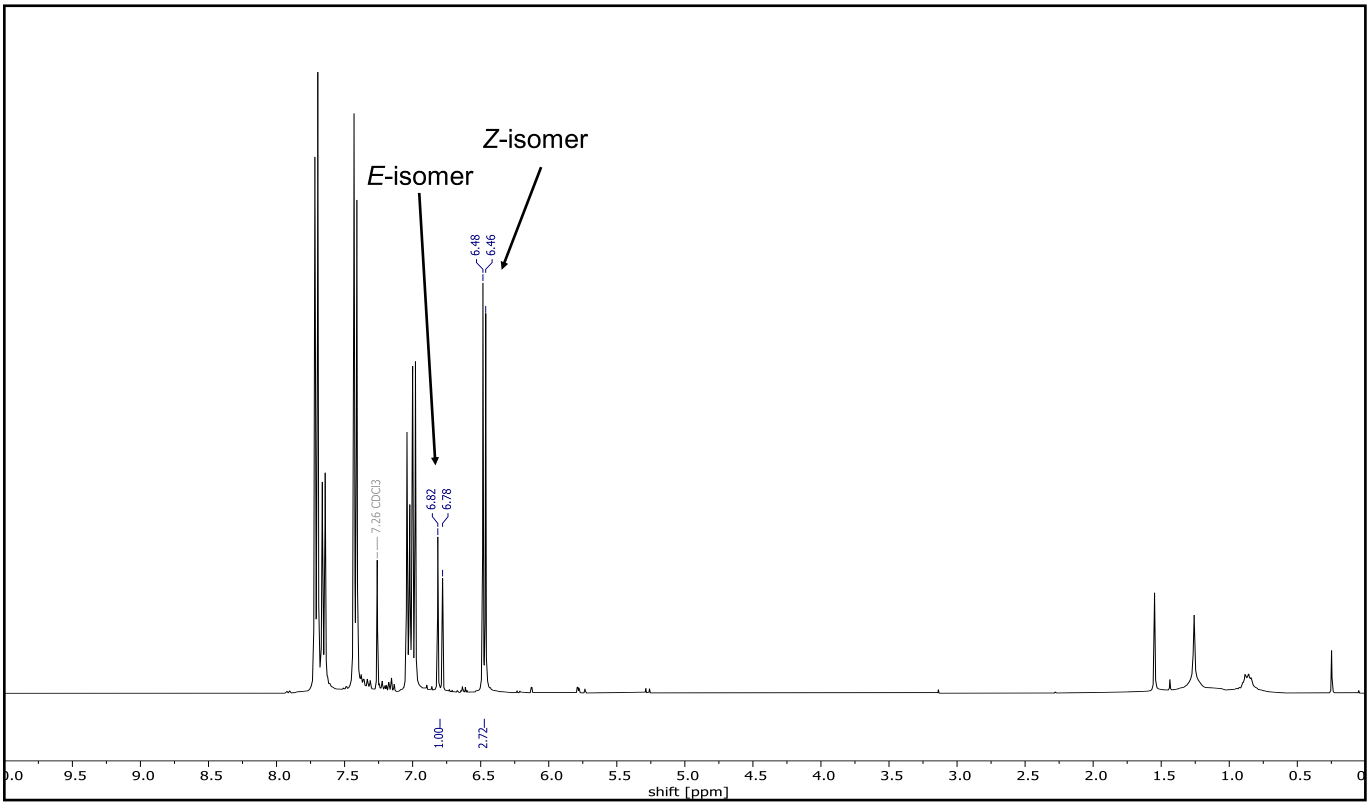

(*E*,*Z*)-1-(2-Bromovinyl)-4-iodobenzene (*E*,*Z*)-4

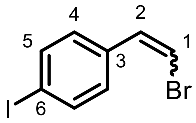

^{1}H NMR ($CDCl_3$), experiment 1

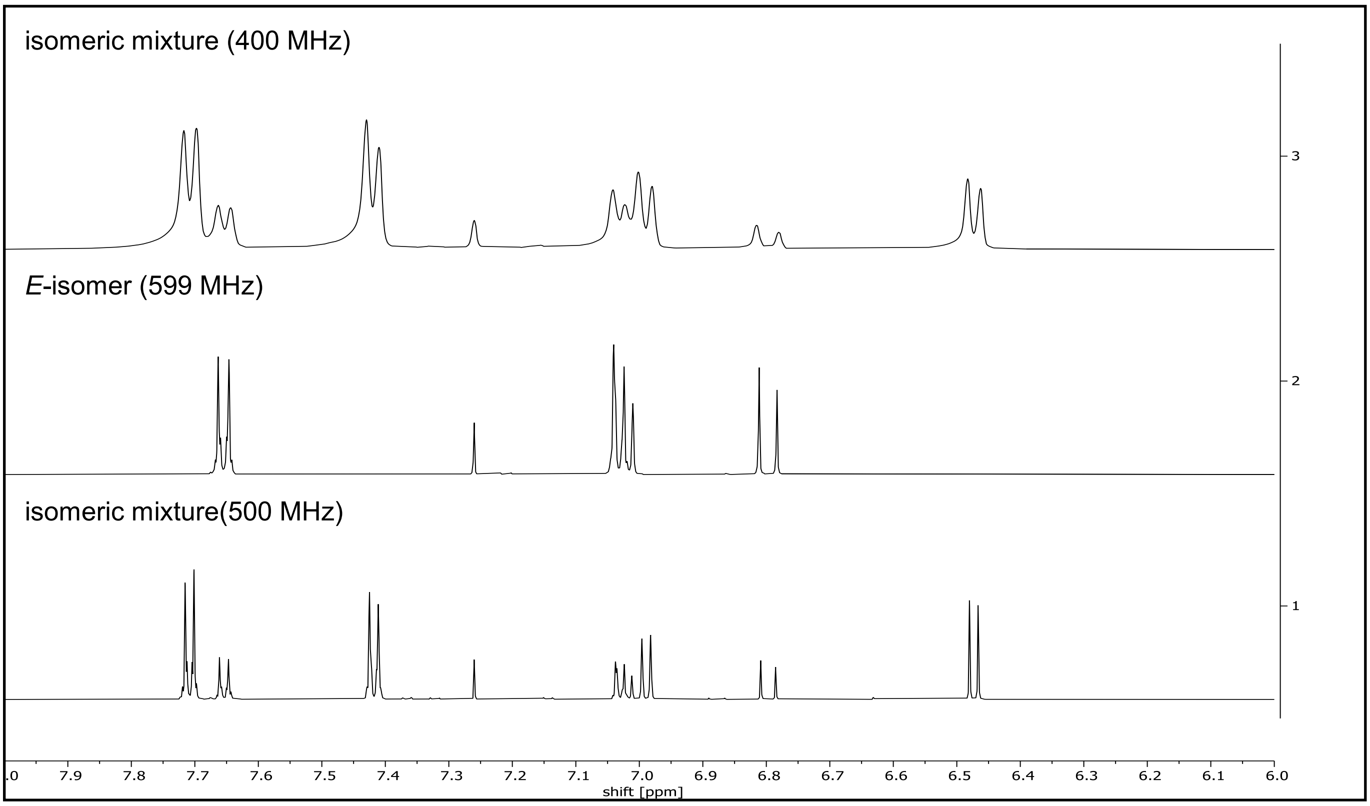

^{1}H NMR ($CDCl_3$), experiment 2

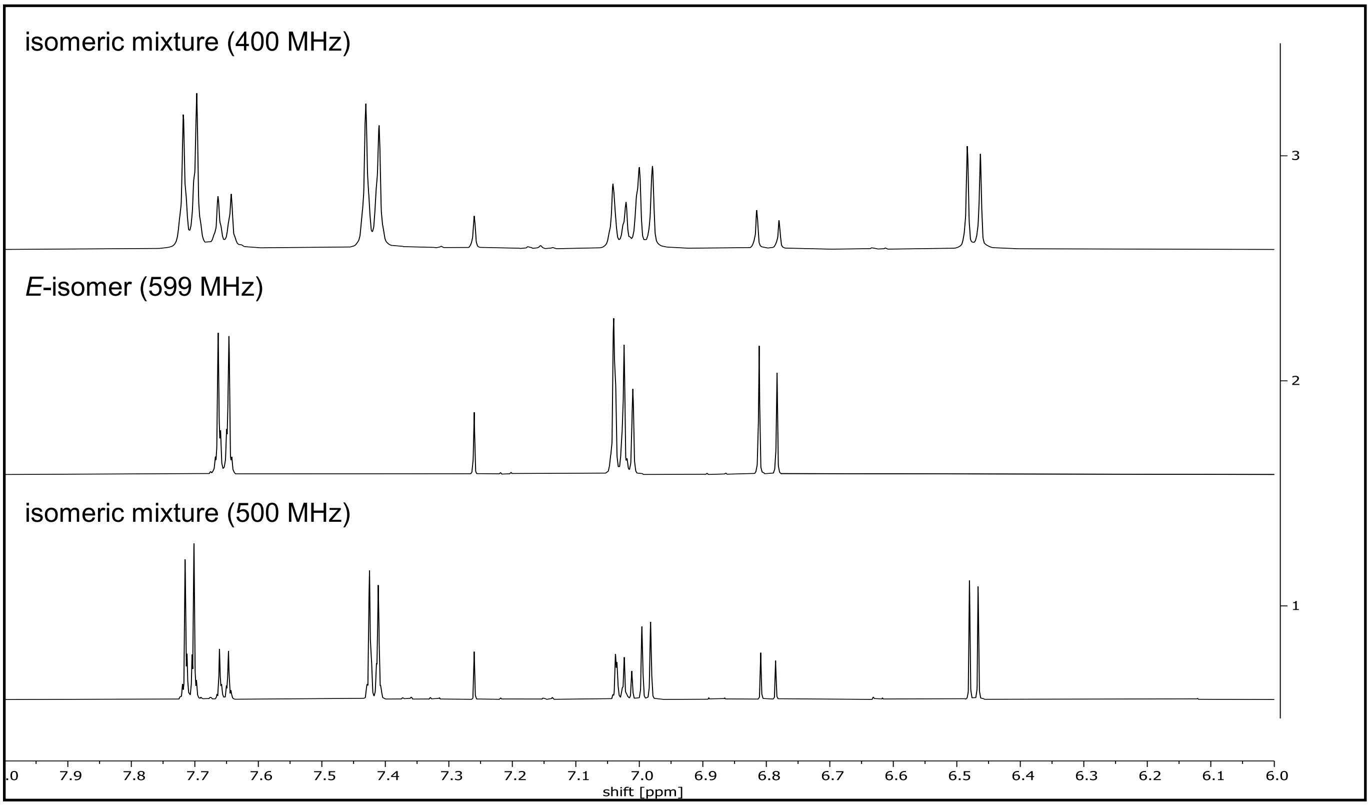

(*E*)-1-(2-Bromovinyl)-4-iodobenzene (*E*)-4

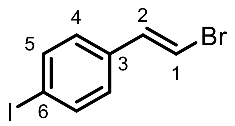

^{1}H NMR (500 MHz, $CDCl_3$)

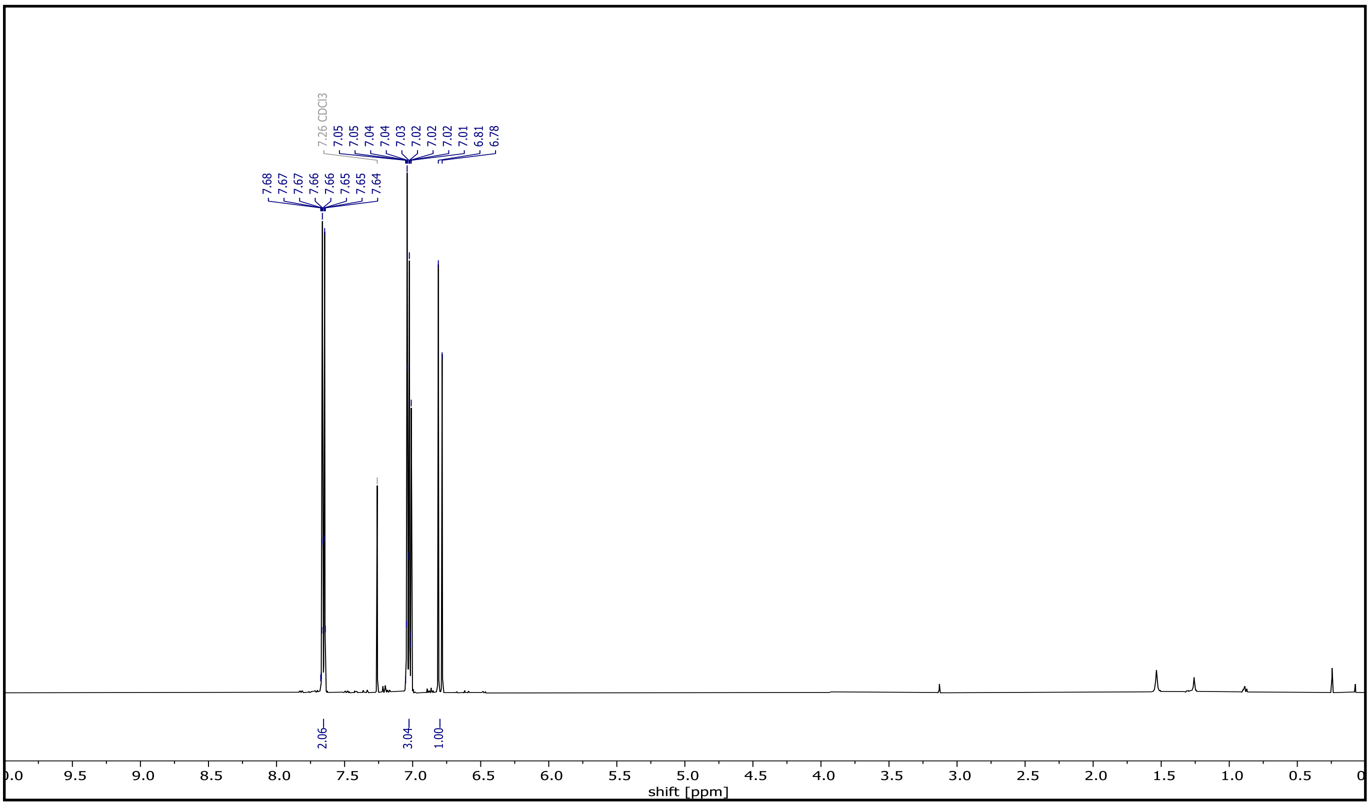

^{13}C NMR (126 MHz, $CDCl_3$)

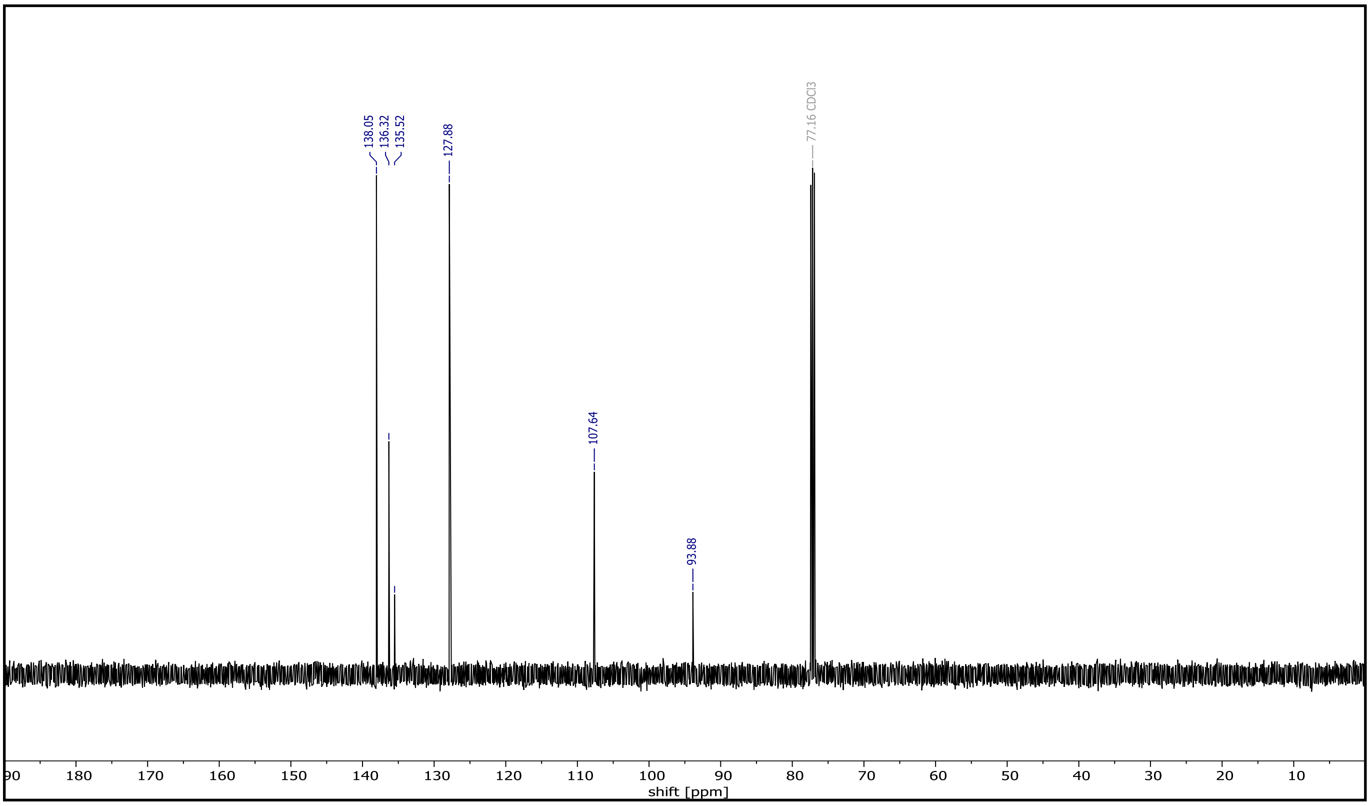

(*Z*,*E*)-1-(2-Bromovinyl)-4-iodobenzene (*Z*,*E*)-4 (*Z*:*E* = 73:27)

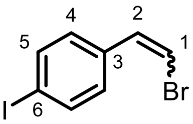

^{1}H NMR (599 MHz, $CDCl_3$) only signals from the *Z*-isomer are labeled

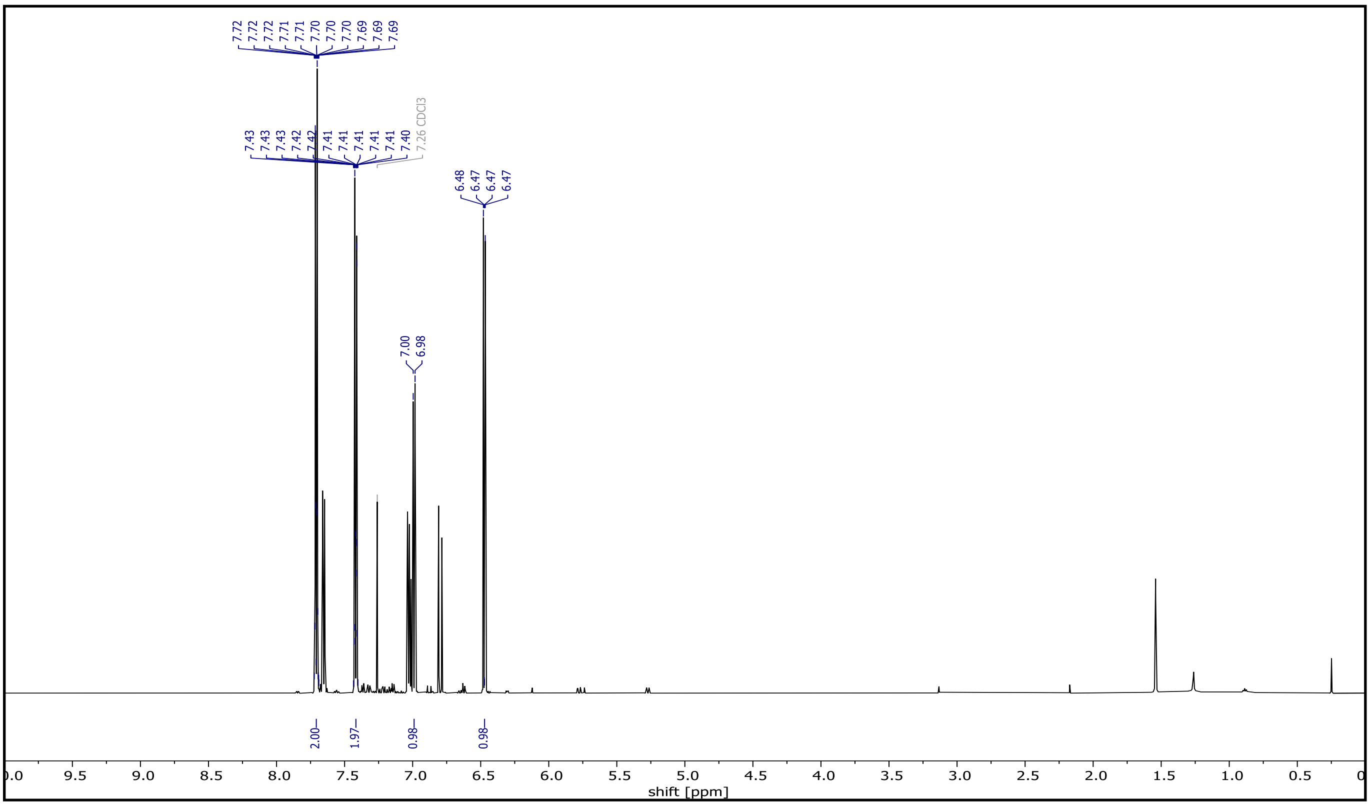

^{13}C NMR (151 MHz, $CDCl_3$) only signals from the *Z* isomer are labeled

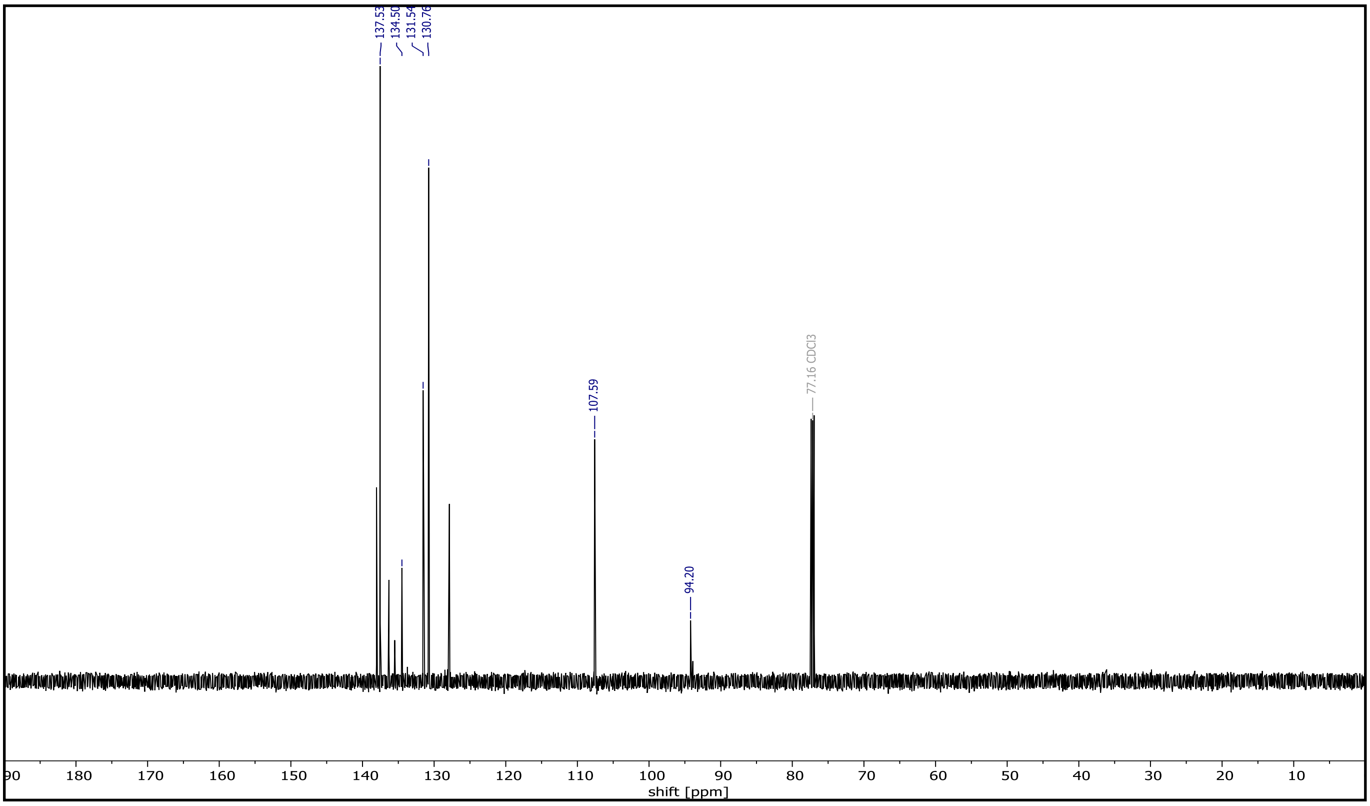

(*E*,*Z*)-1-(2-Bromovinyl)-2-fluorobenzene (*E*,*Z*)-5

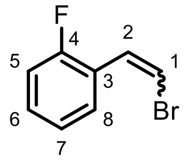

^{1}H NMR (400 MHz, $CDCl_3$), experiment 1

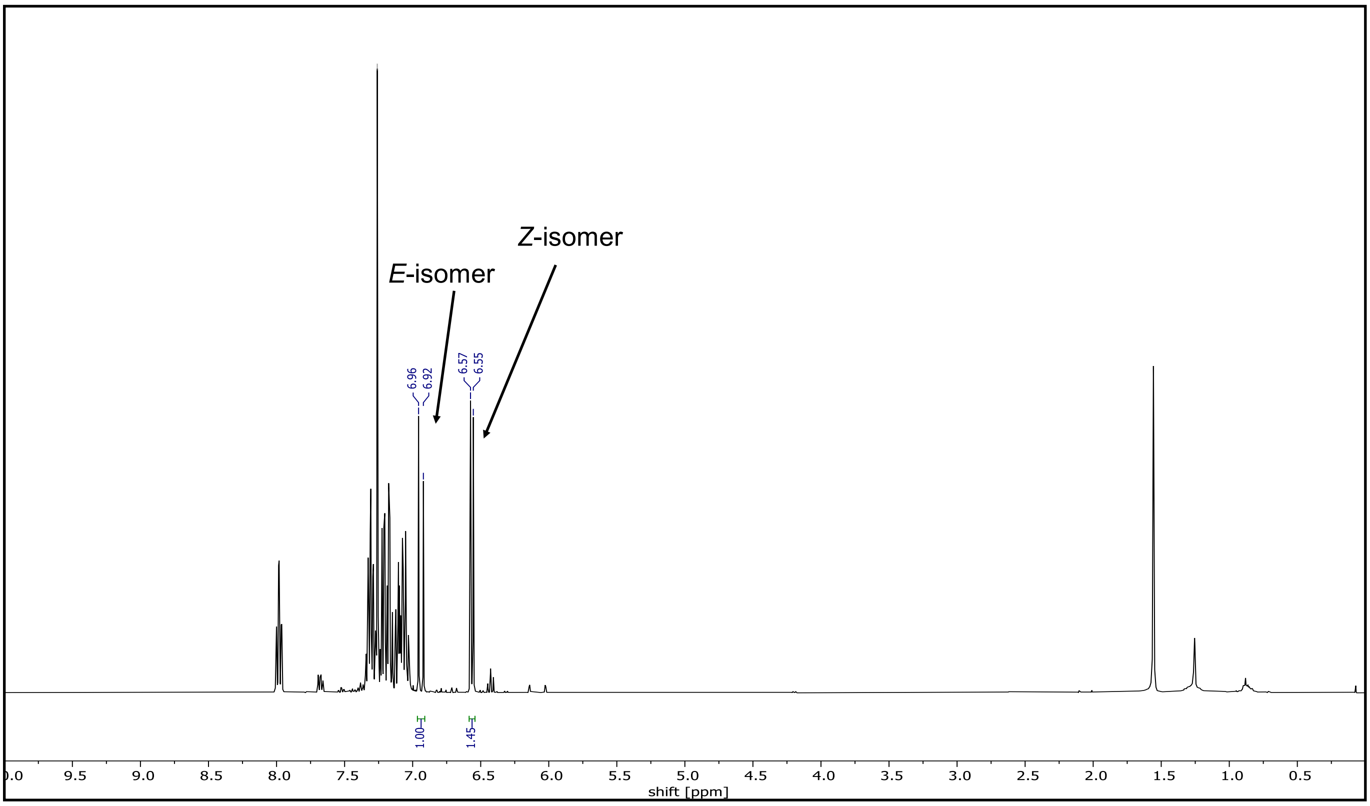

^{1}H NMR (400 MHz, $CDCl_3$), experiment 2

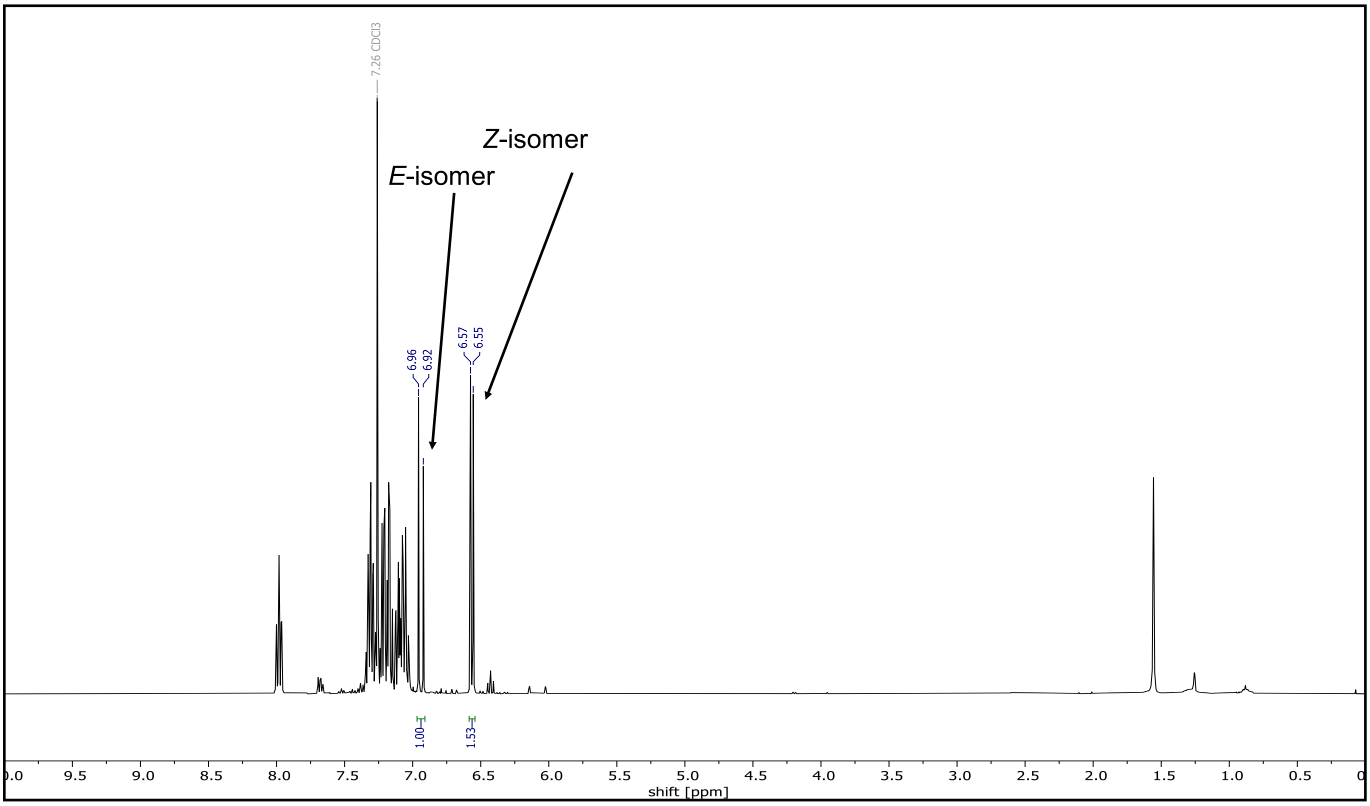

(E,Z)-1-(2-Bromovinyl)-2-fluorobenzene (E,Z)-5

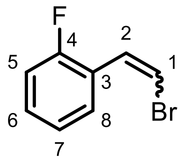

^{1}H NMR (CDCl$_3$), experiment 1

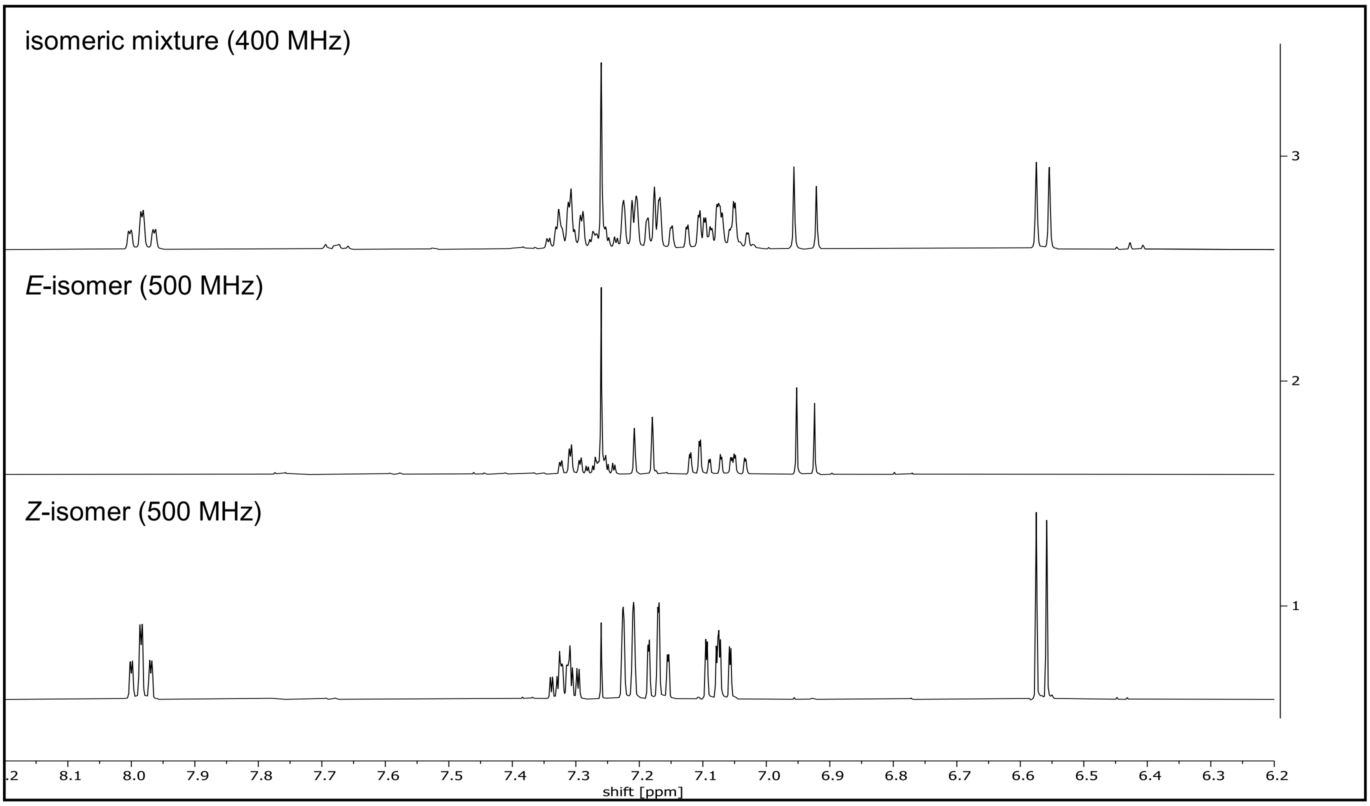

^{1}H NMR (CDCl$_3$), experiment 2

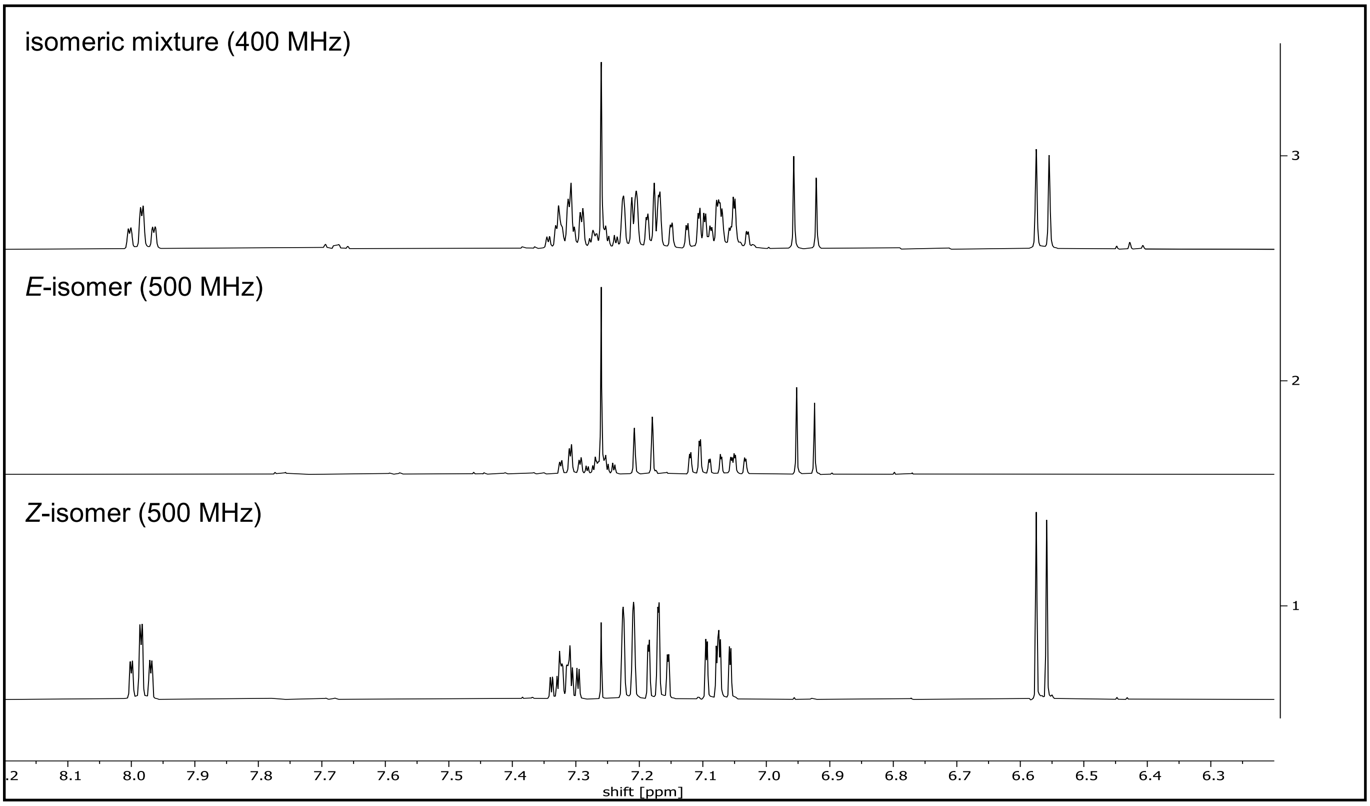

(*E*)-1-(2-Bromovinyl)-2-fluorobenzene (*E*)-5

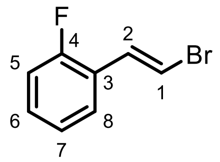

^{1}H NMR (500 MHz, $CDCl_3$)

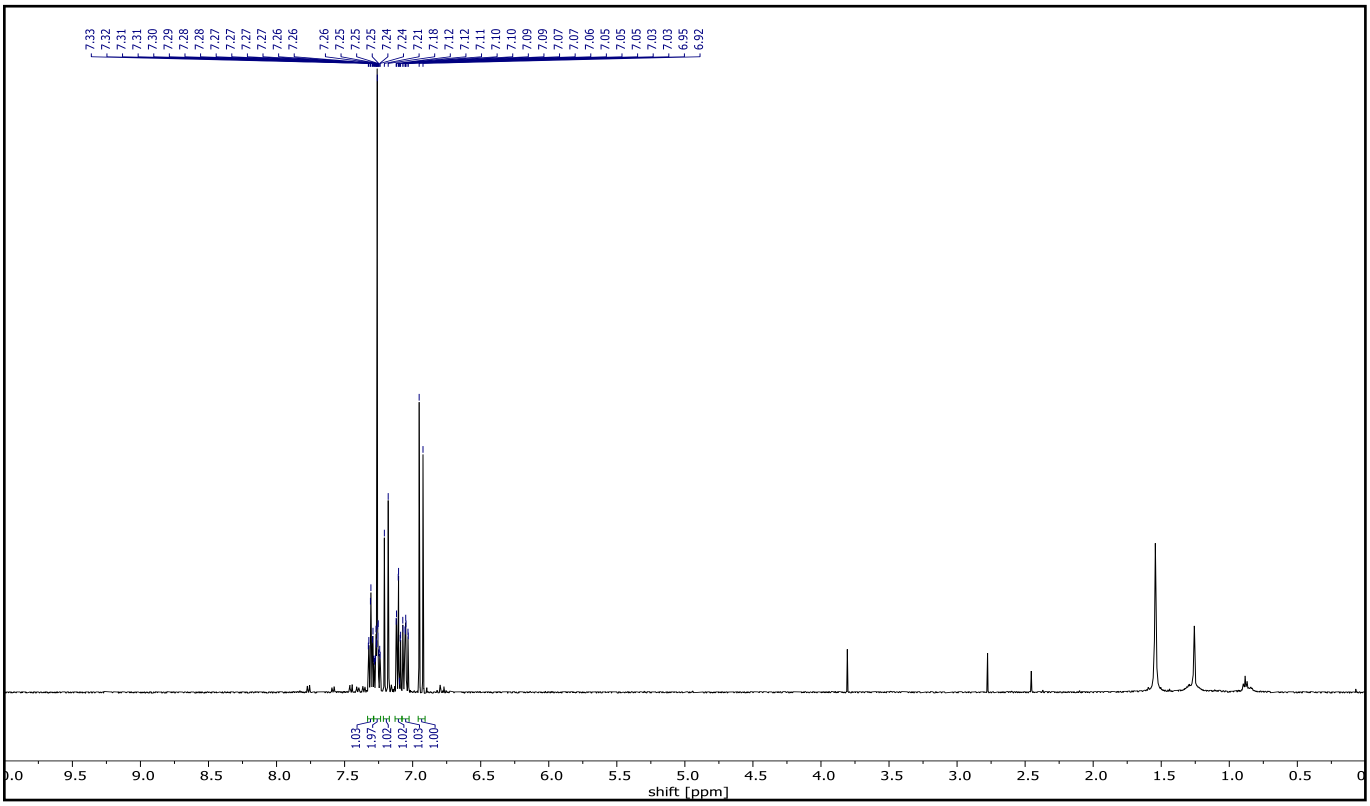

^{13}C{^{1}H} NMR (101 MHz, $CDCl_3$)

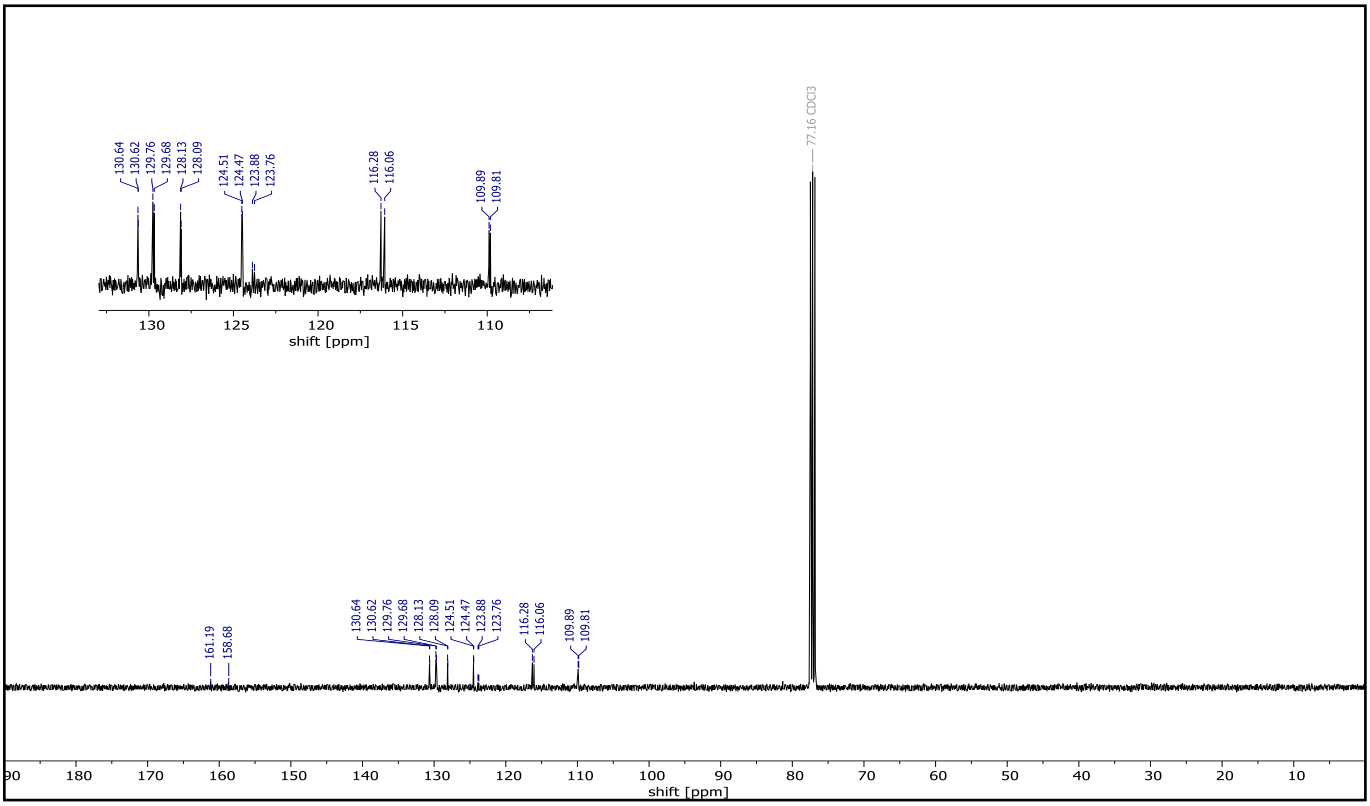

(*E*)-1-(2-Bromovinyl)-2-fluorobenzene (*E*)-5

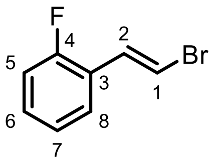

^{19}F NMR (470 MHz, $CDCl_3$)

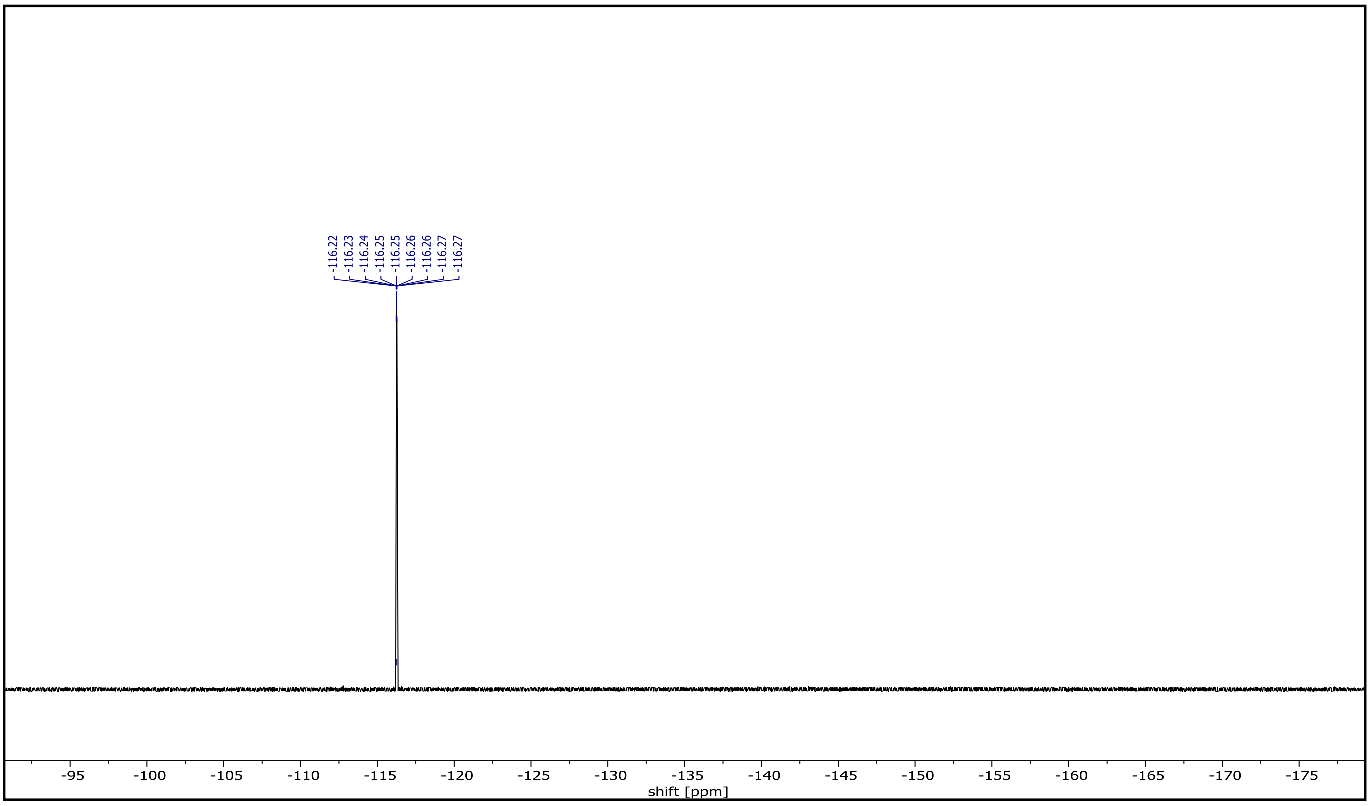

(*Z*)-1-(2-Bromovinyl)-2-fluorobenzene (*E*,*Z*)-5

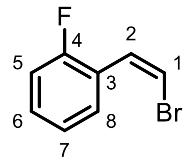

^{1}H NMR (500 MHz, $CDCl_3$)

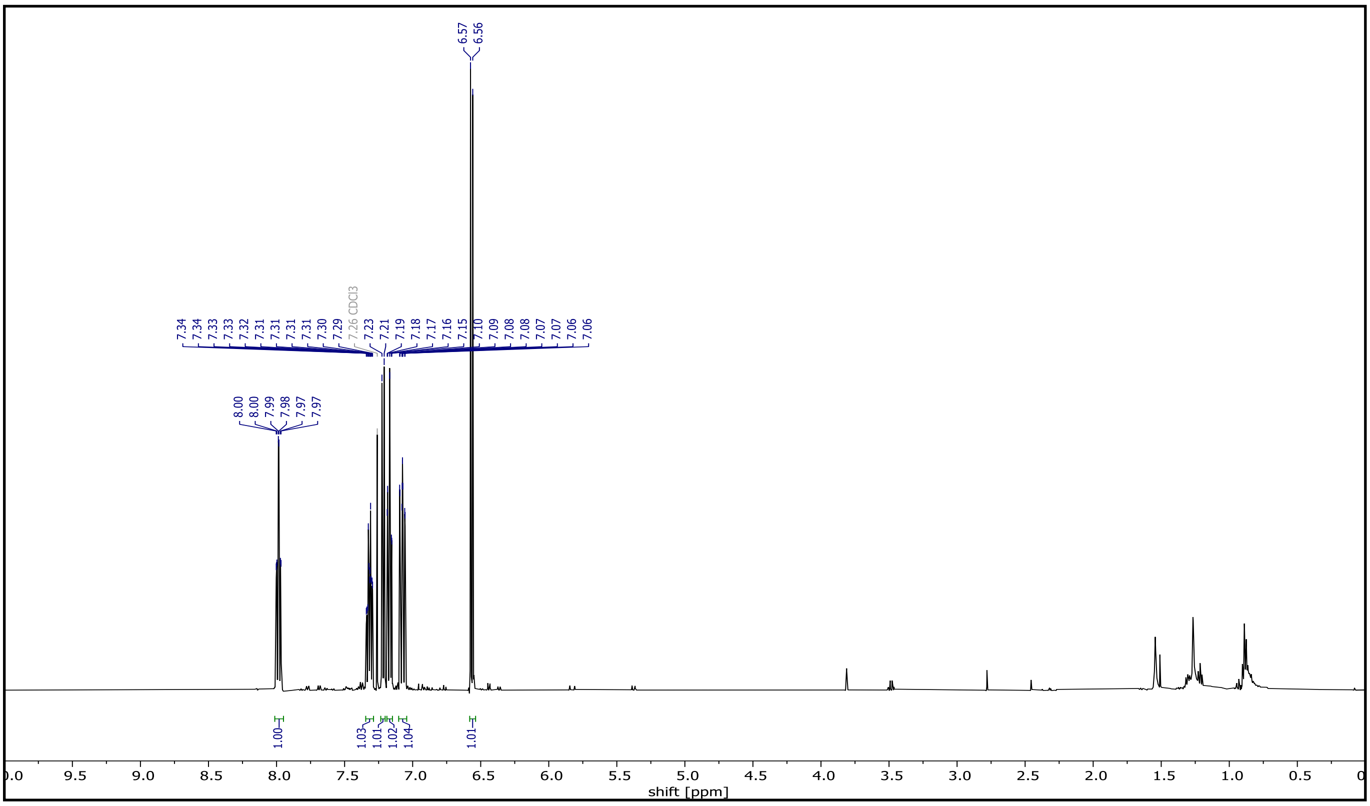

$^{13}C\{^1H\}$ NMR (126 MHz, $CDCl_3$)

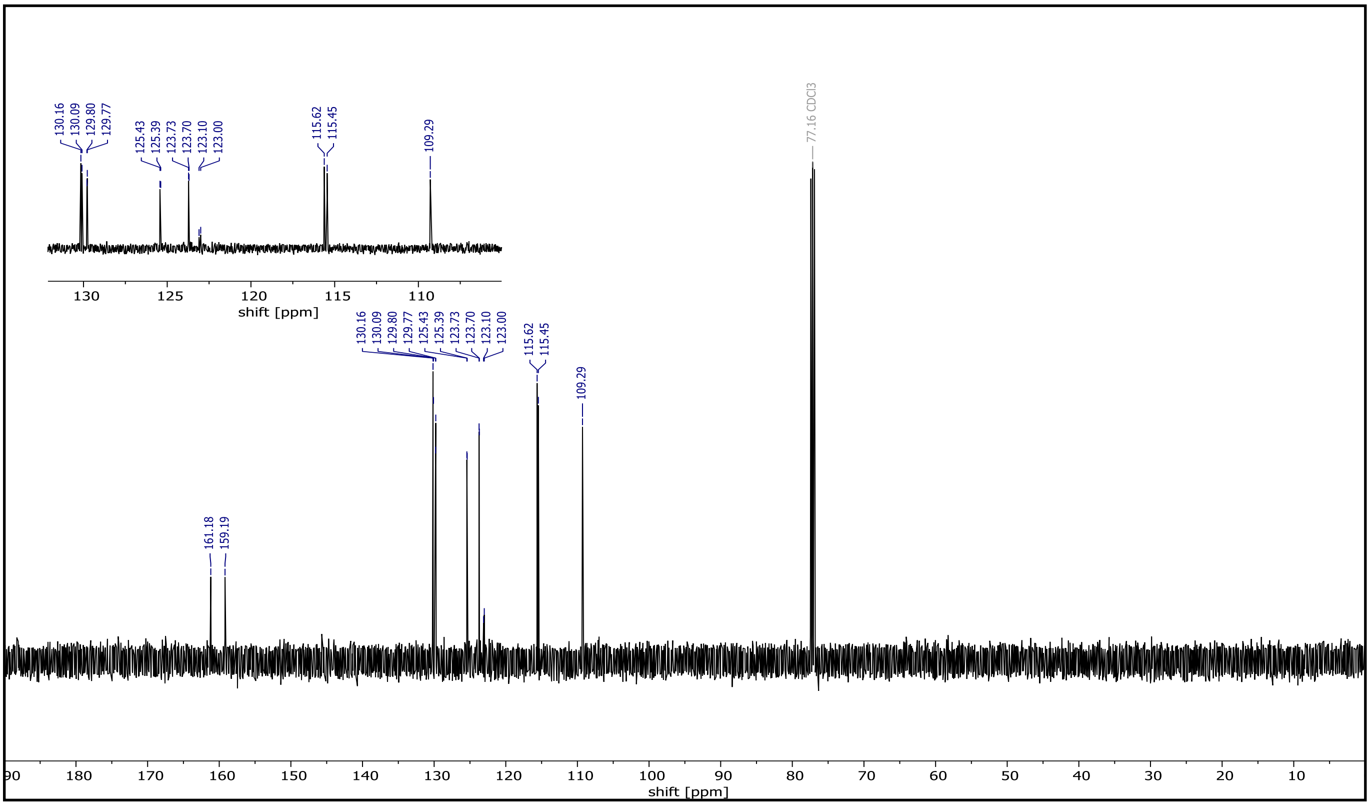

(*Z*)-1-(2-Bromovinyl)-2-fluorobenzene (*E*,*Z*)-5

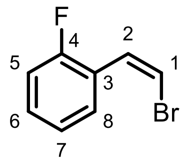

^{19}F NMR (470 MHz, $CDCl_3$)

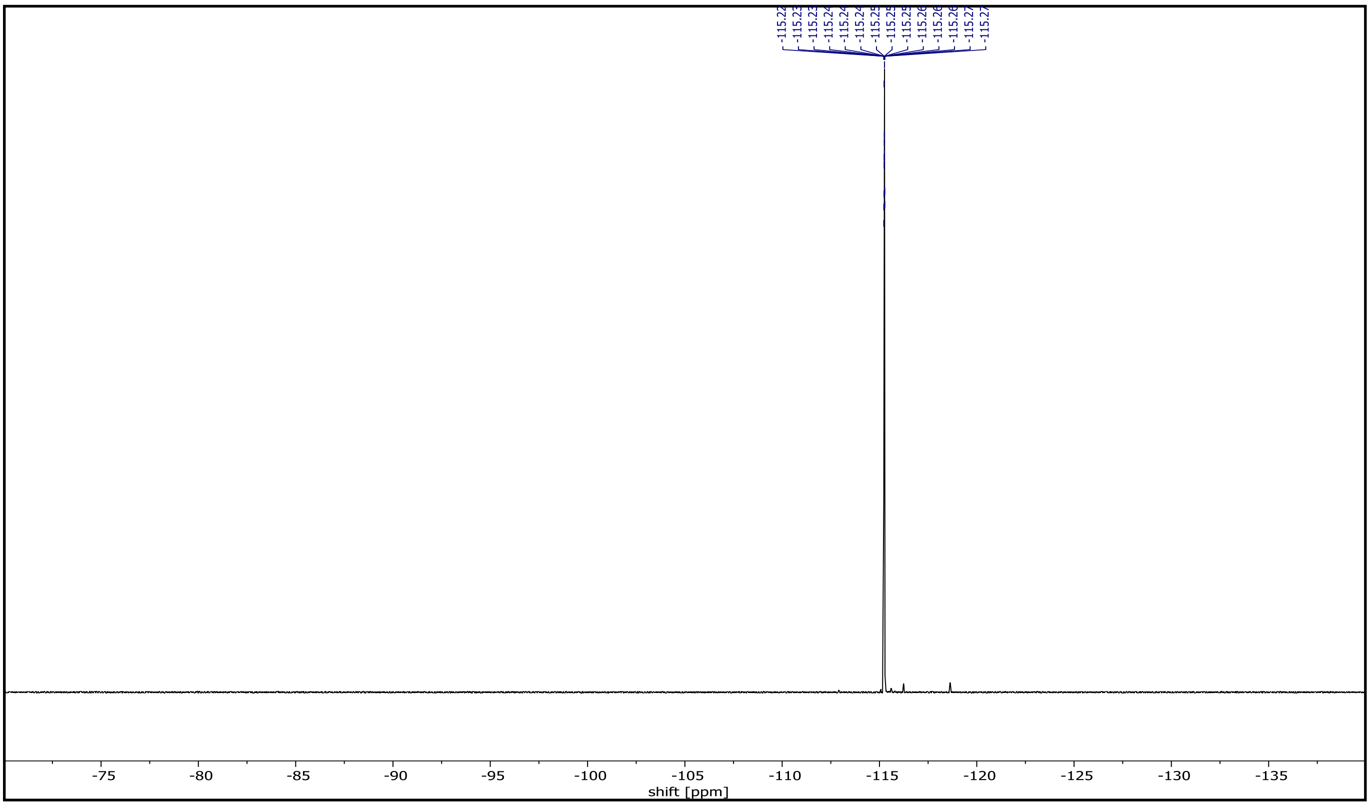

(*E*,*Z*)-1-Bromo-3-(2-bromovinyl)benzene (*E*,*Z*)-6

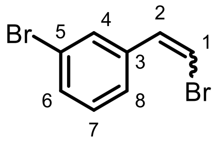

^1H NMR (400 MHz, $CDCl_3$), experiment 1

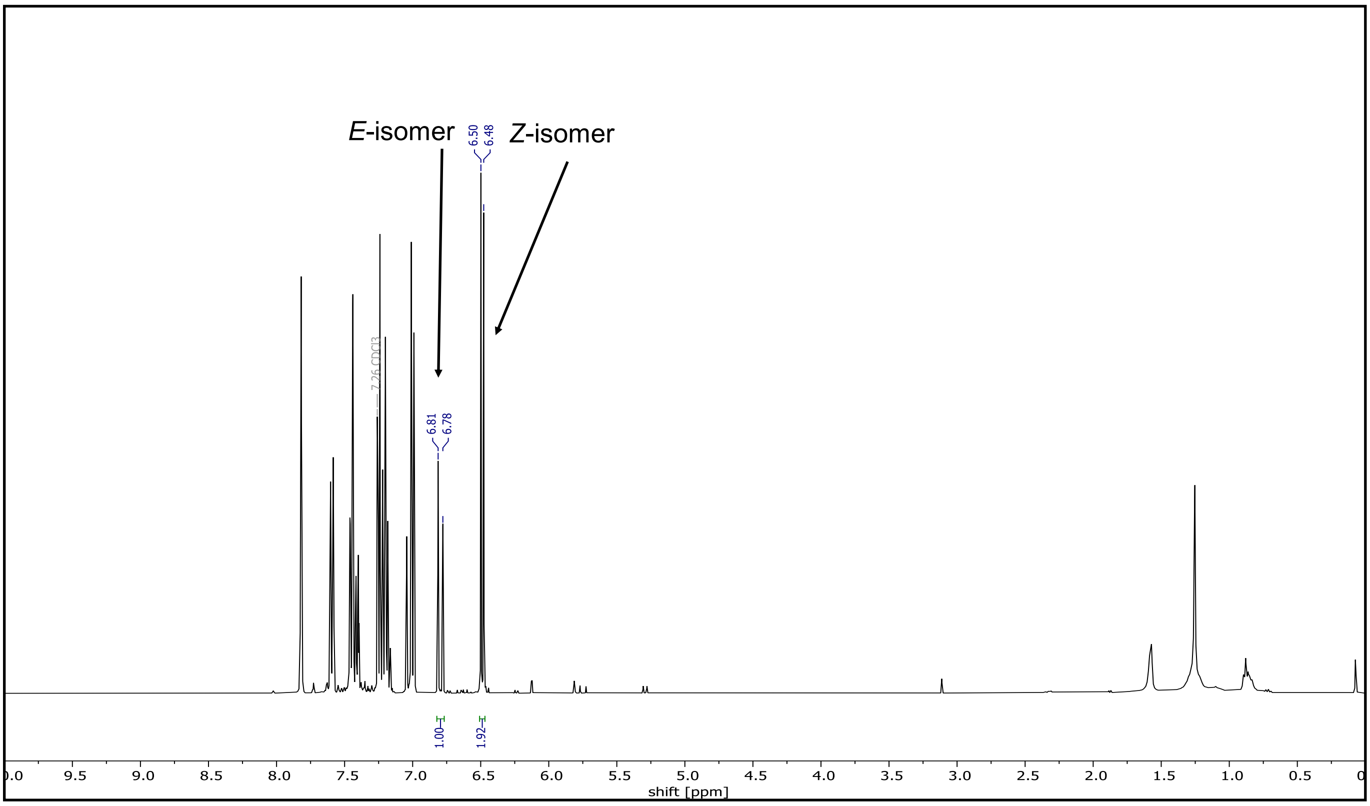

^1H NMR (400 MHz, $CDCl_3$), experiment 2

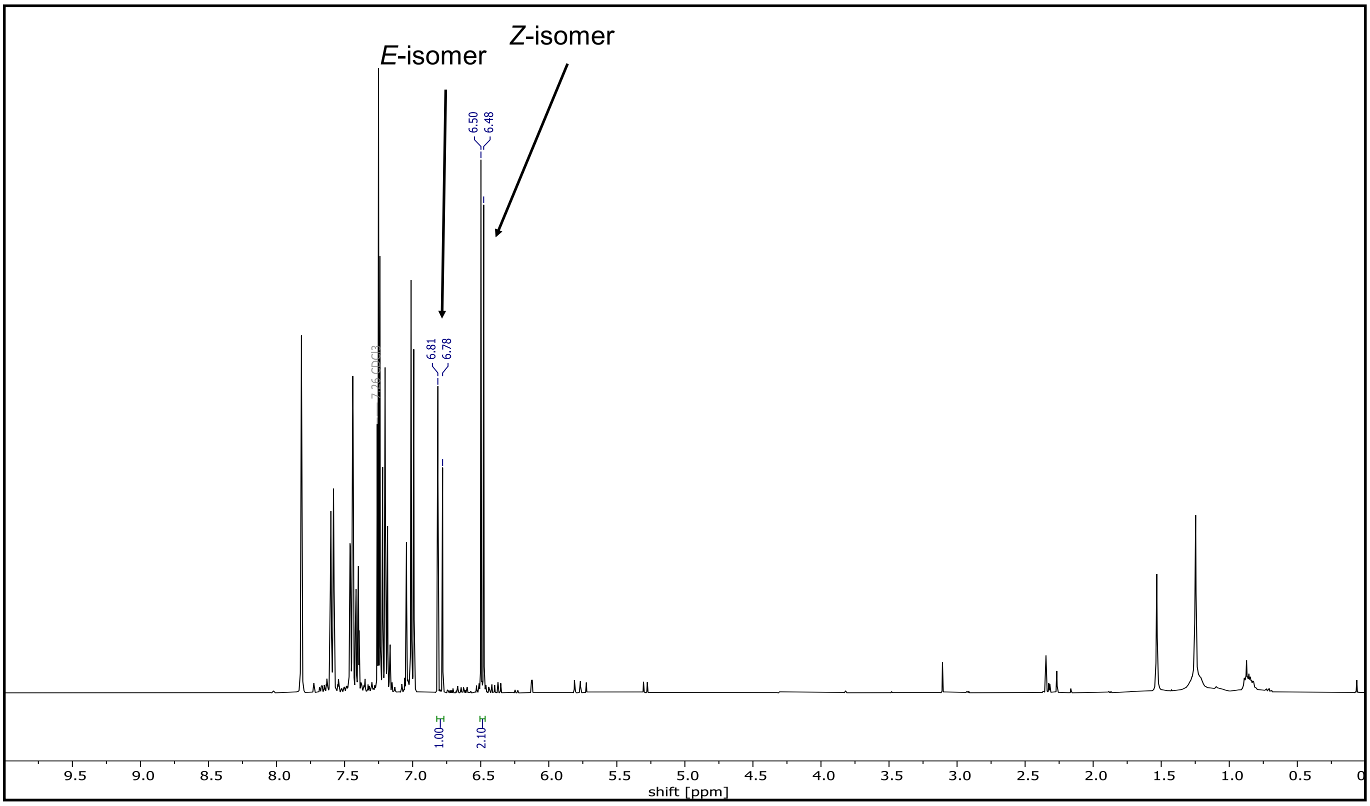

(*E*,*Z*)-1-Bromo-3-(2-bromovinyl)benzene (*E*,*Z*)-6

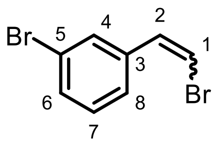

^{1}H NMR ($CDCl_3$), experiment 1

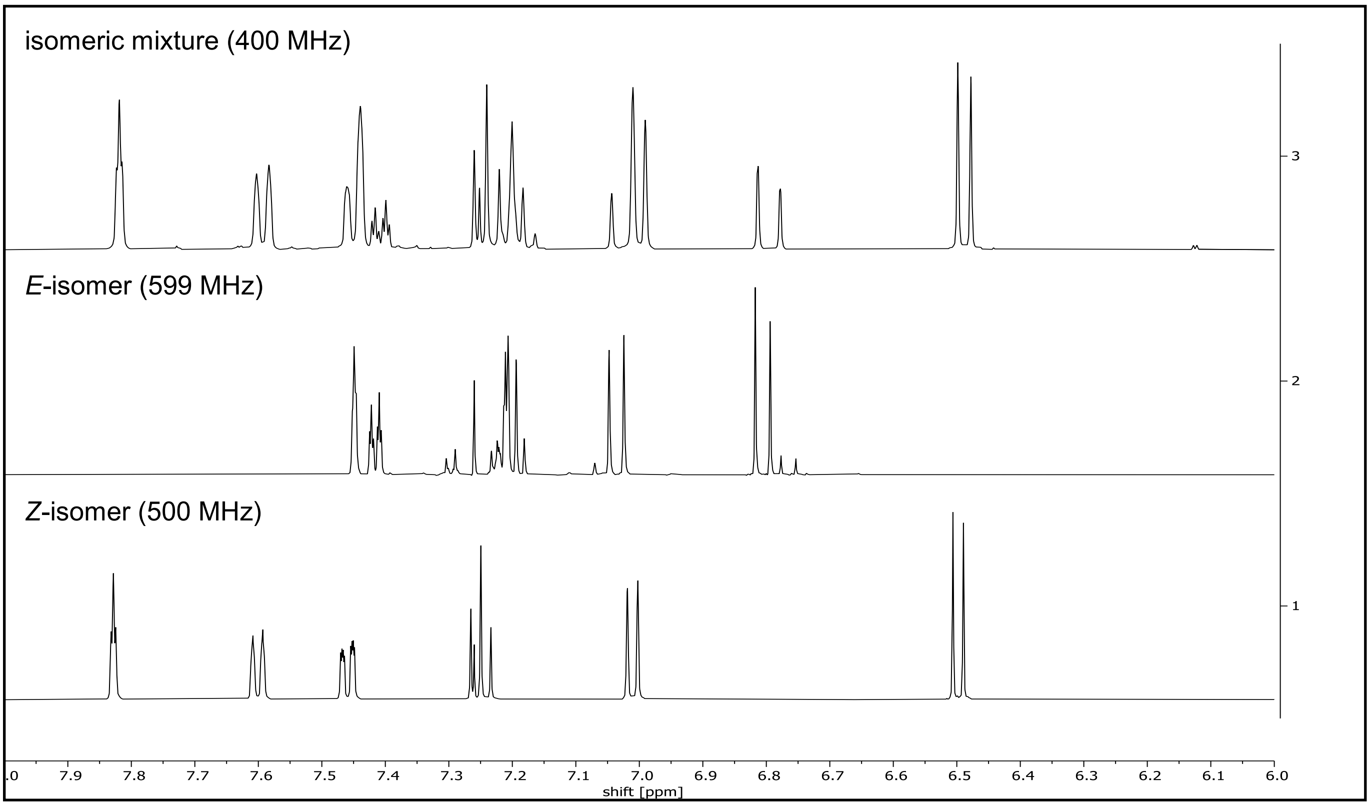

^{1}H NMR ($CDCl_3$), experiment 2

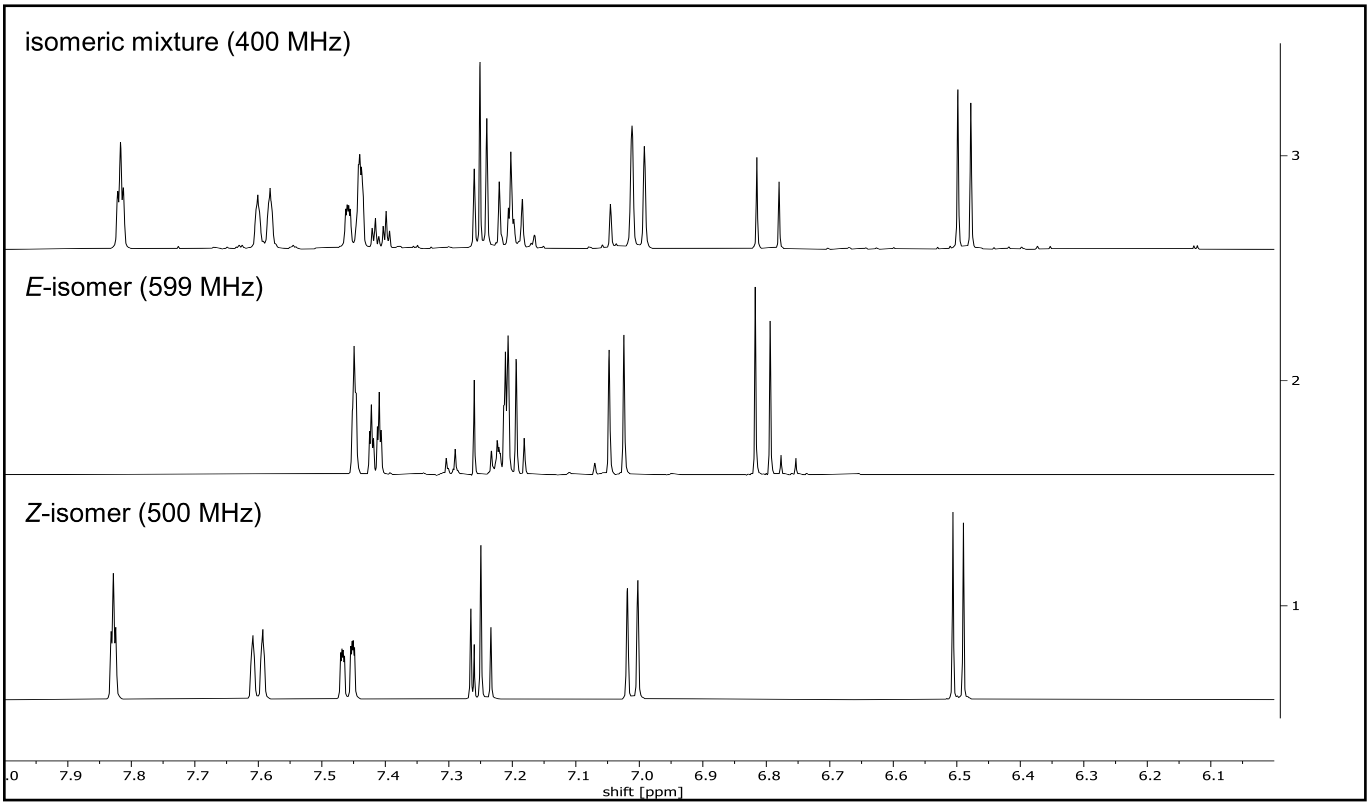

(*E*)-1-Bromo-3-(2-bromovinyl)benzene (*E*)-6

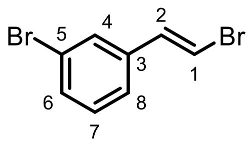

^{1}H NMR (599 MHz, $CDCl_3$)

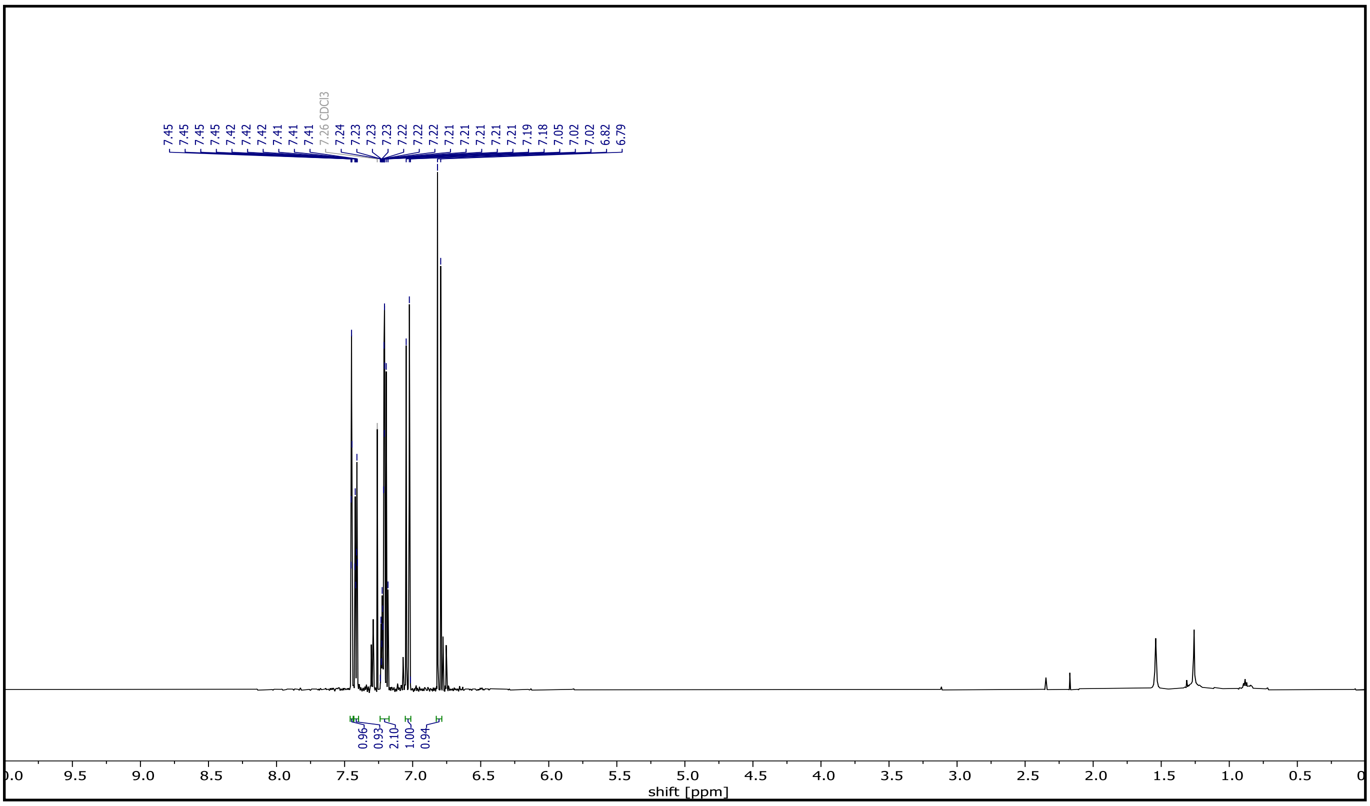

^{13}C{^{1}H} NMR (151 MHz, $CDCl_3$)

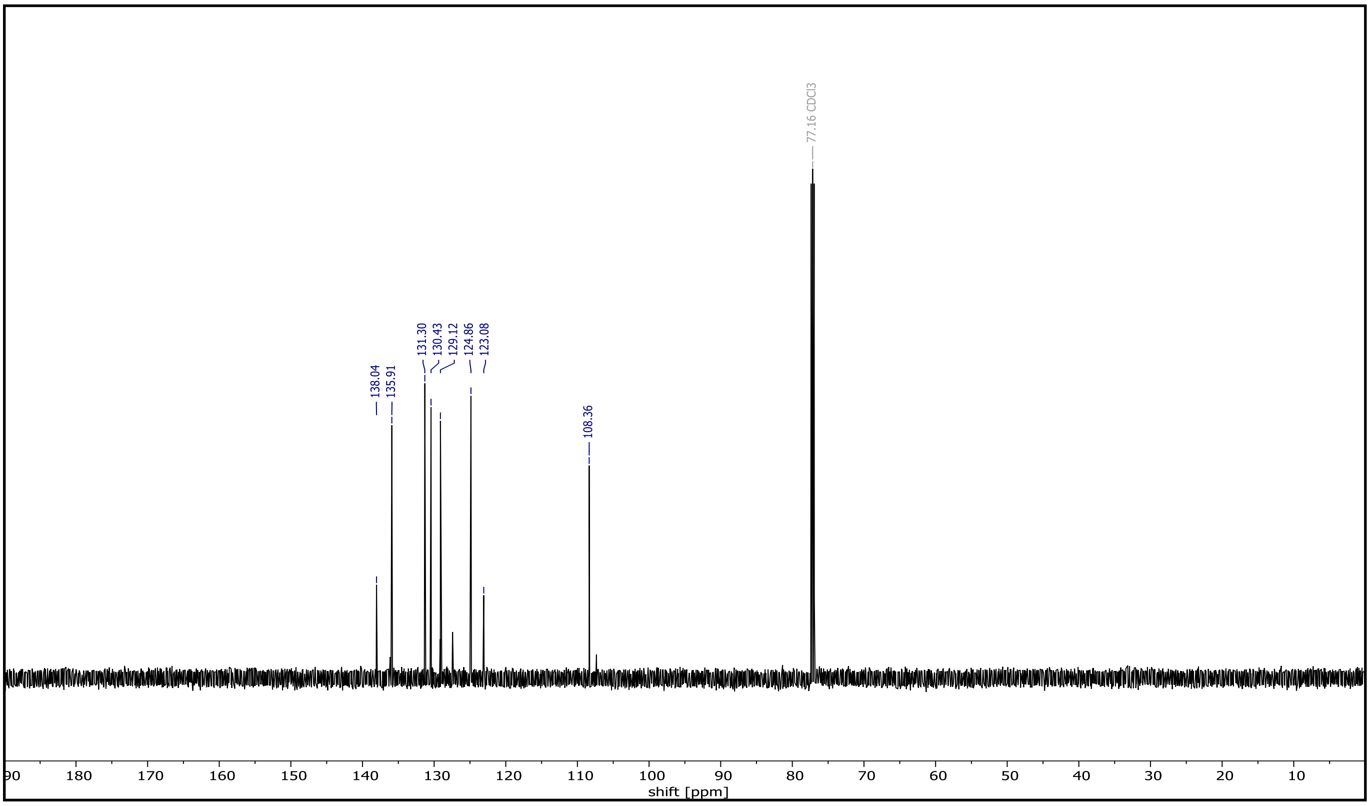

(*Z*)-1-Bromo-3-(2-bromovinyl)benzene (*Z*)-6

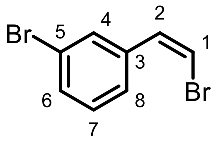

^{1}H-NMR (500 MHz, $CDCl_3$)

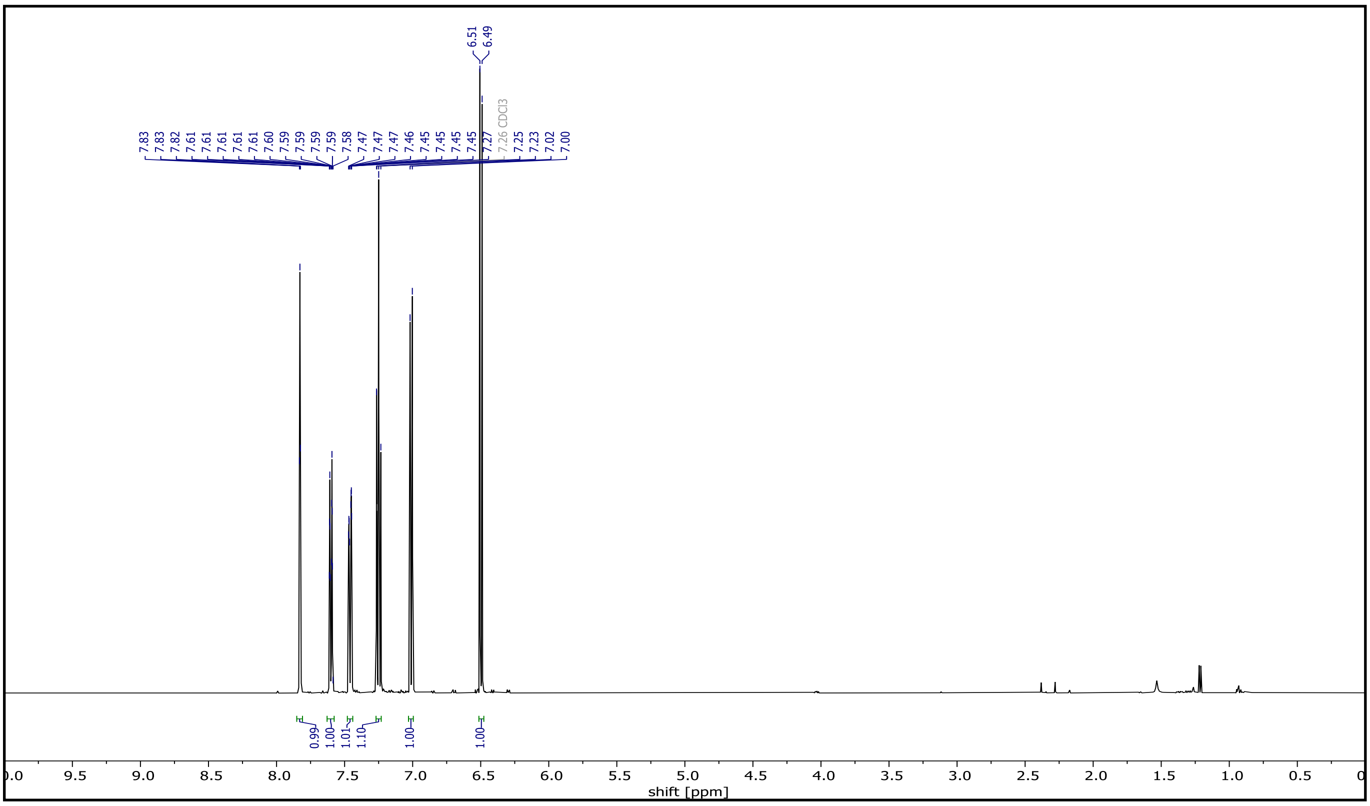

^{13}C^{1}H NMR (126 MHz, $CDCl_3$)

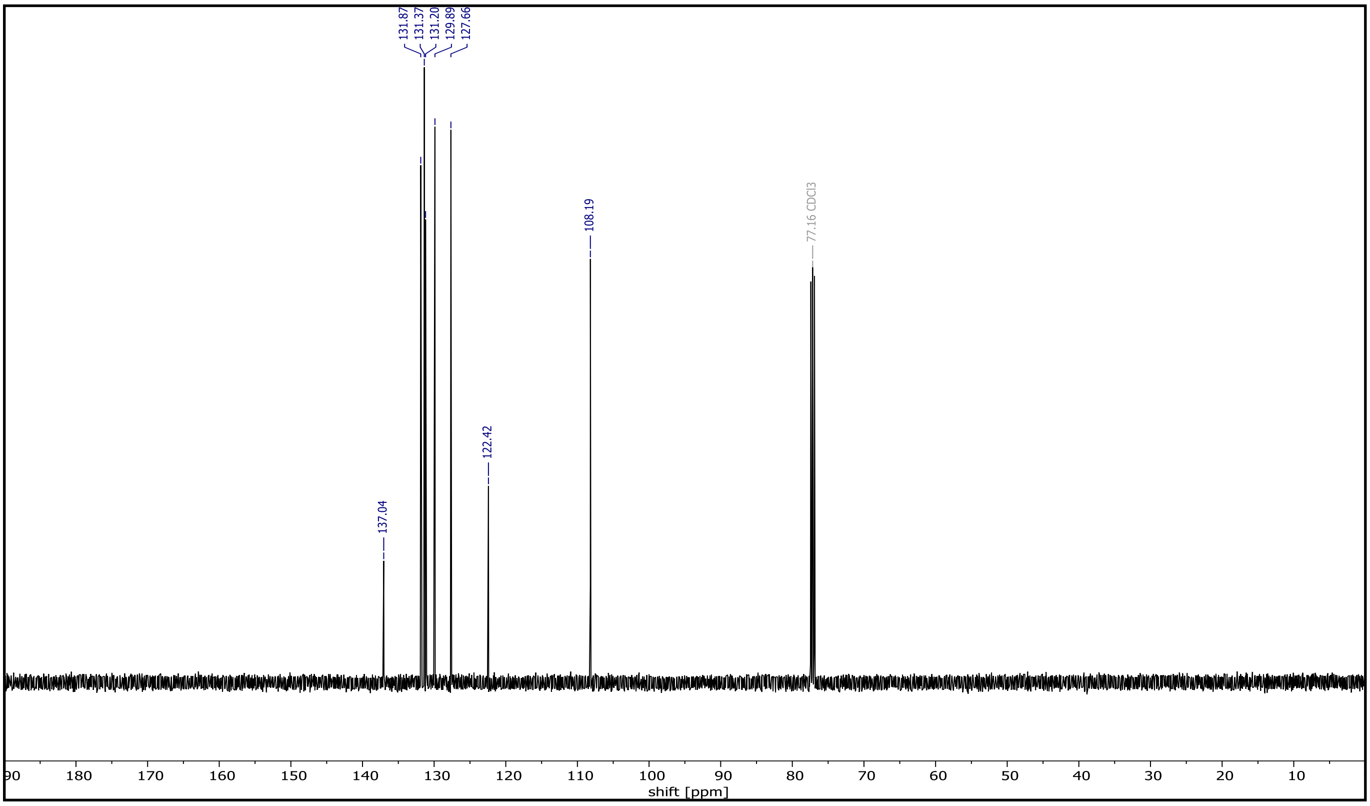

(*E*,*Z*)-(2-Bromovinyl)benzene (*E*,*Z*)-7

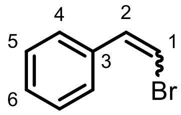

^{1}H NMR (400 MHz, $CDCl_3$), experiment 1

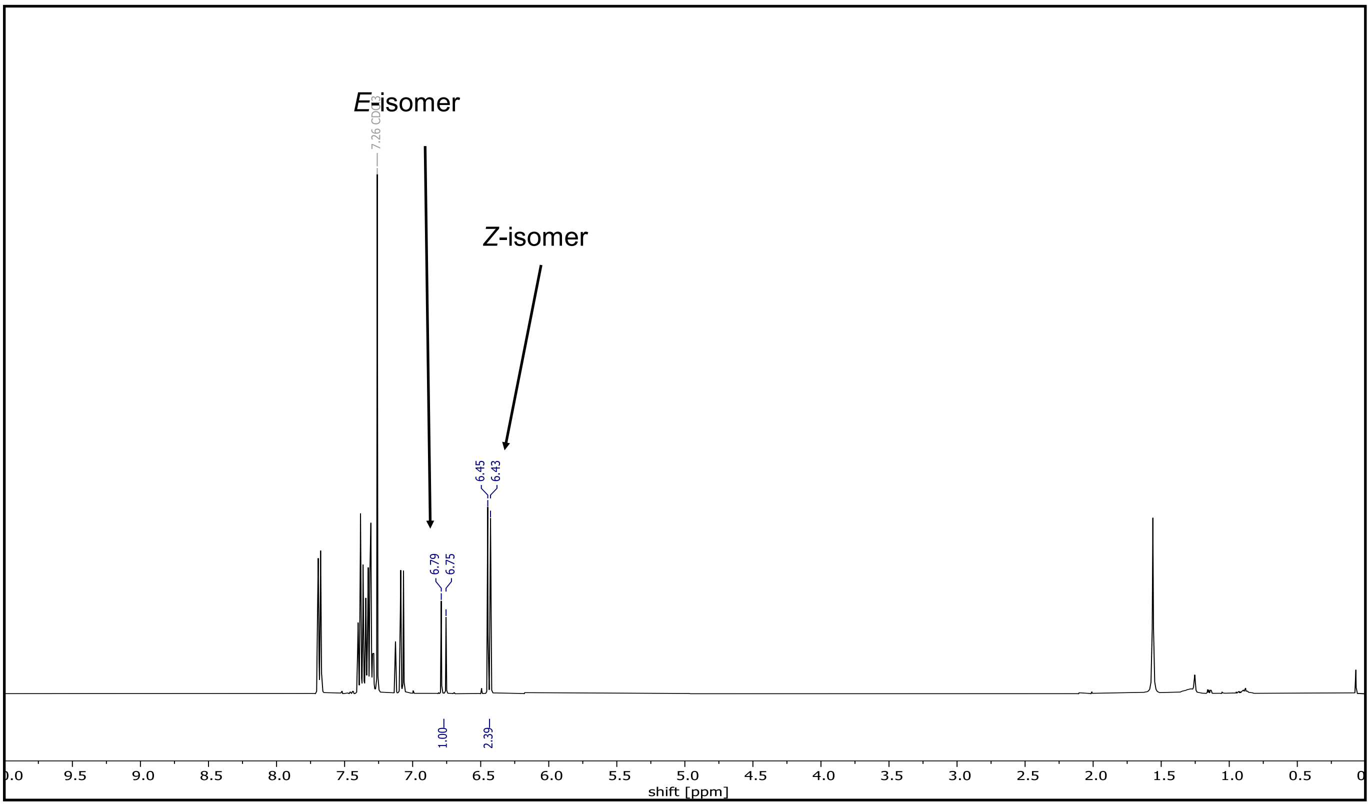

^{1}H NMR (400 MHz, $CDCl_3$), experiment 2

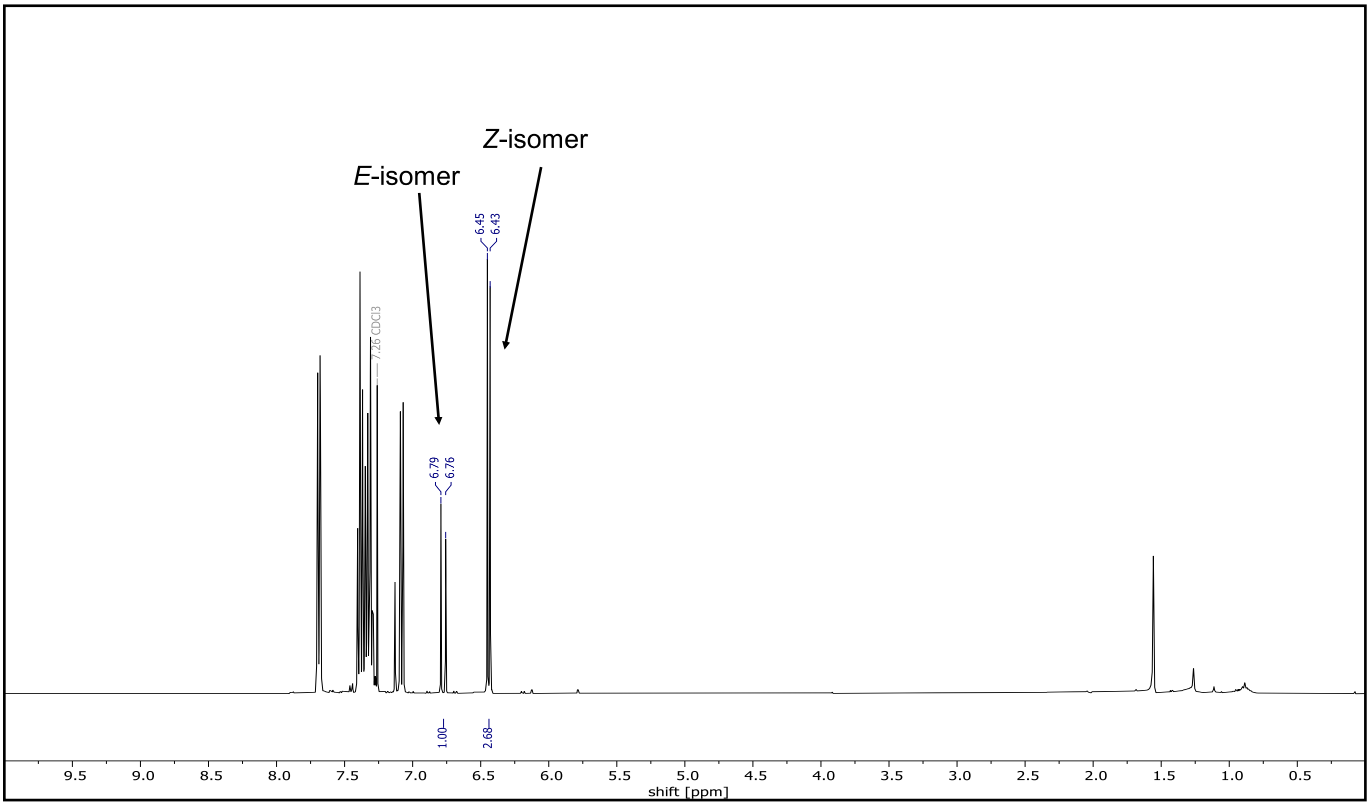

(*E*,*Z*)-(2-Bromovinyl)benzene (*E*,*Z*)-7

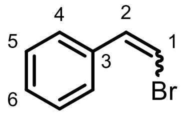

^{1}H NMR ($CDCl_3$), experiment 1

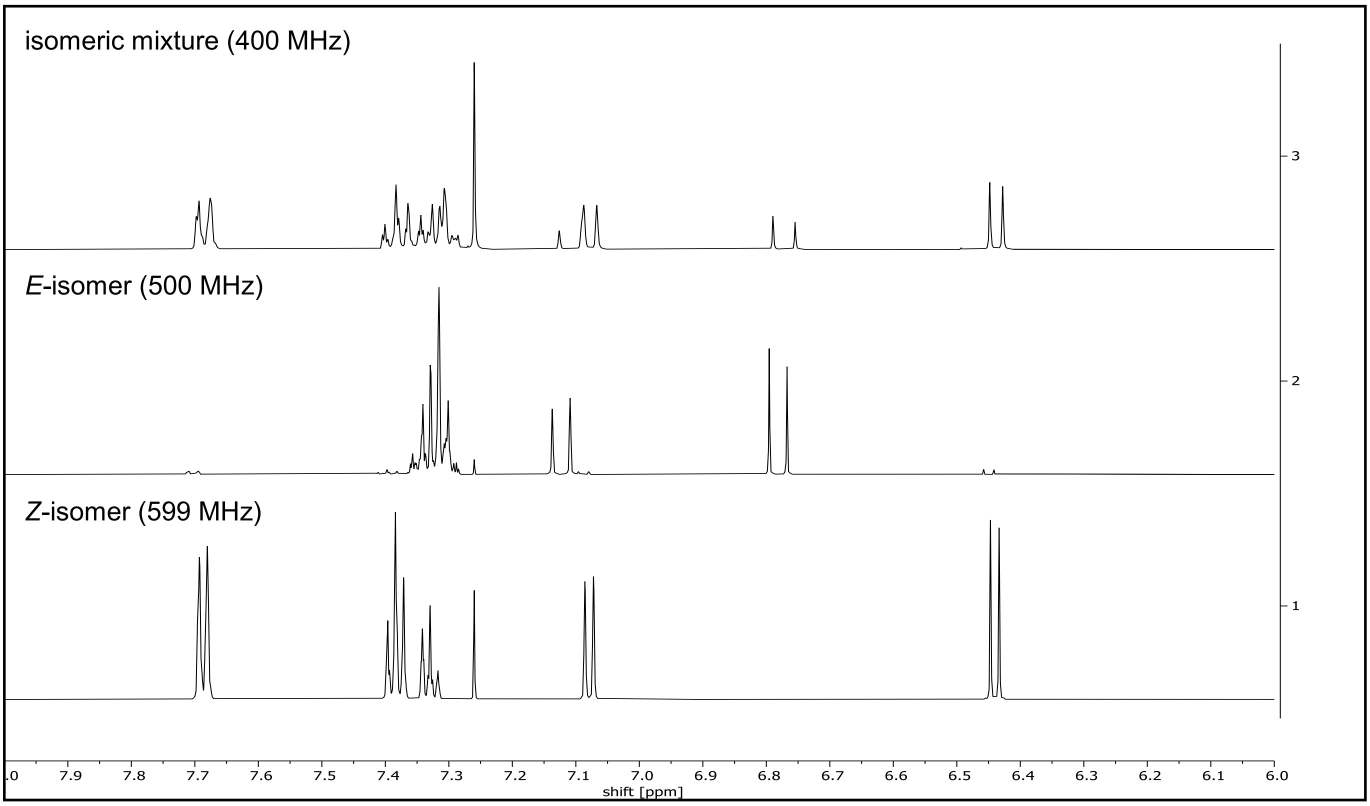

^{1}H NMR ($CDCl_3$), experiment 2

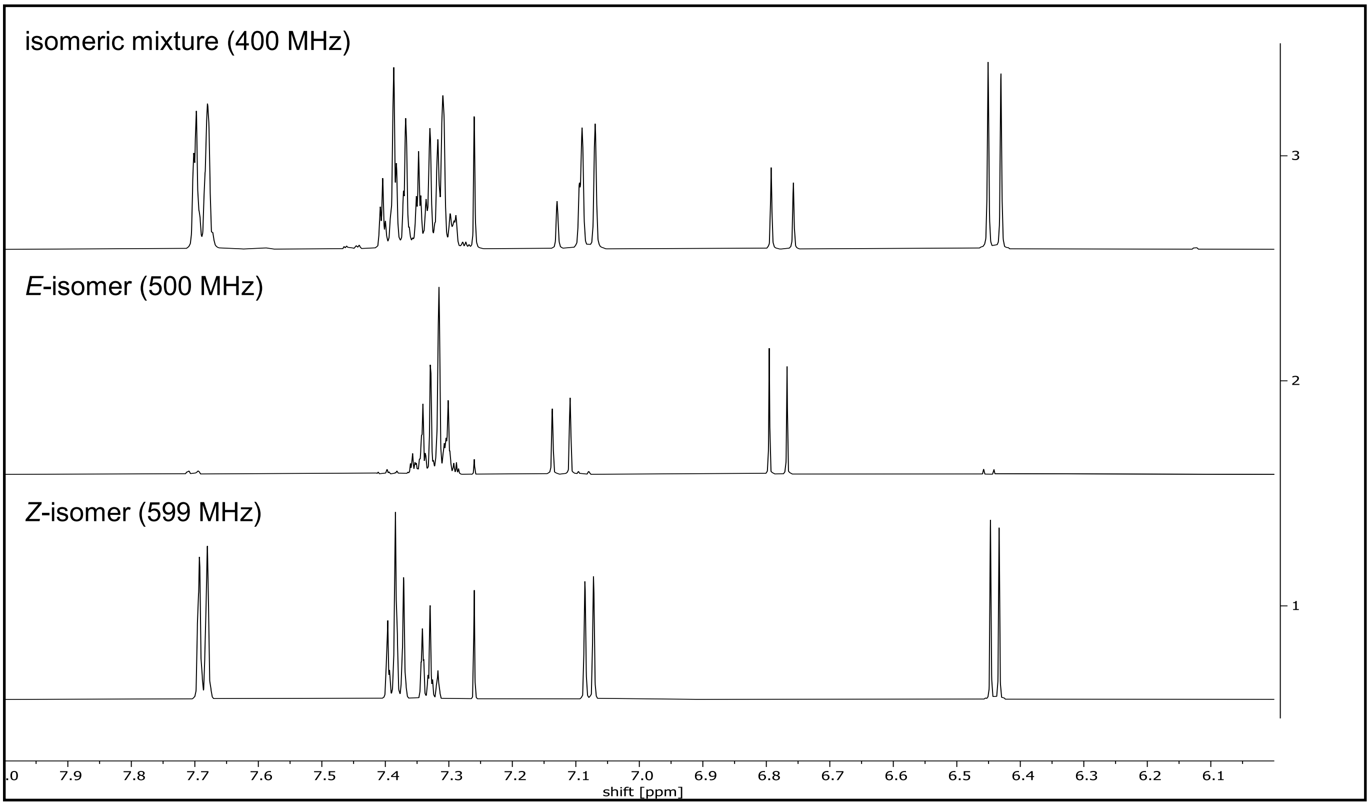

(*E*)-(2-Bromovinyl) (*E*)-7

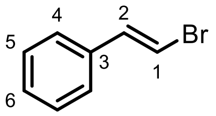

^{1}H-NMR (500 MHz, $CDCl_3$)

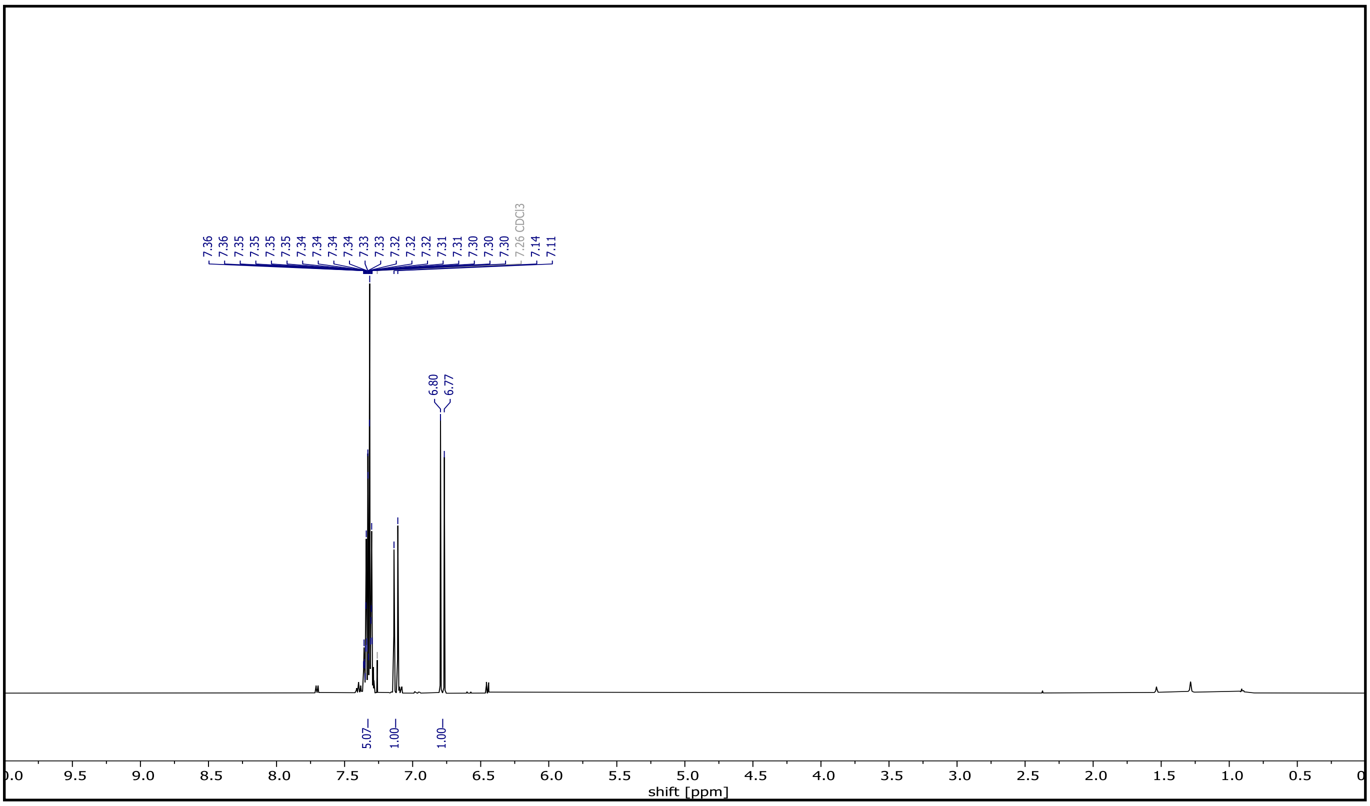

$^{13}C\{^{1}H\}$ NMR (126 MHz, $CDCl_3$)

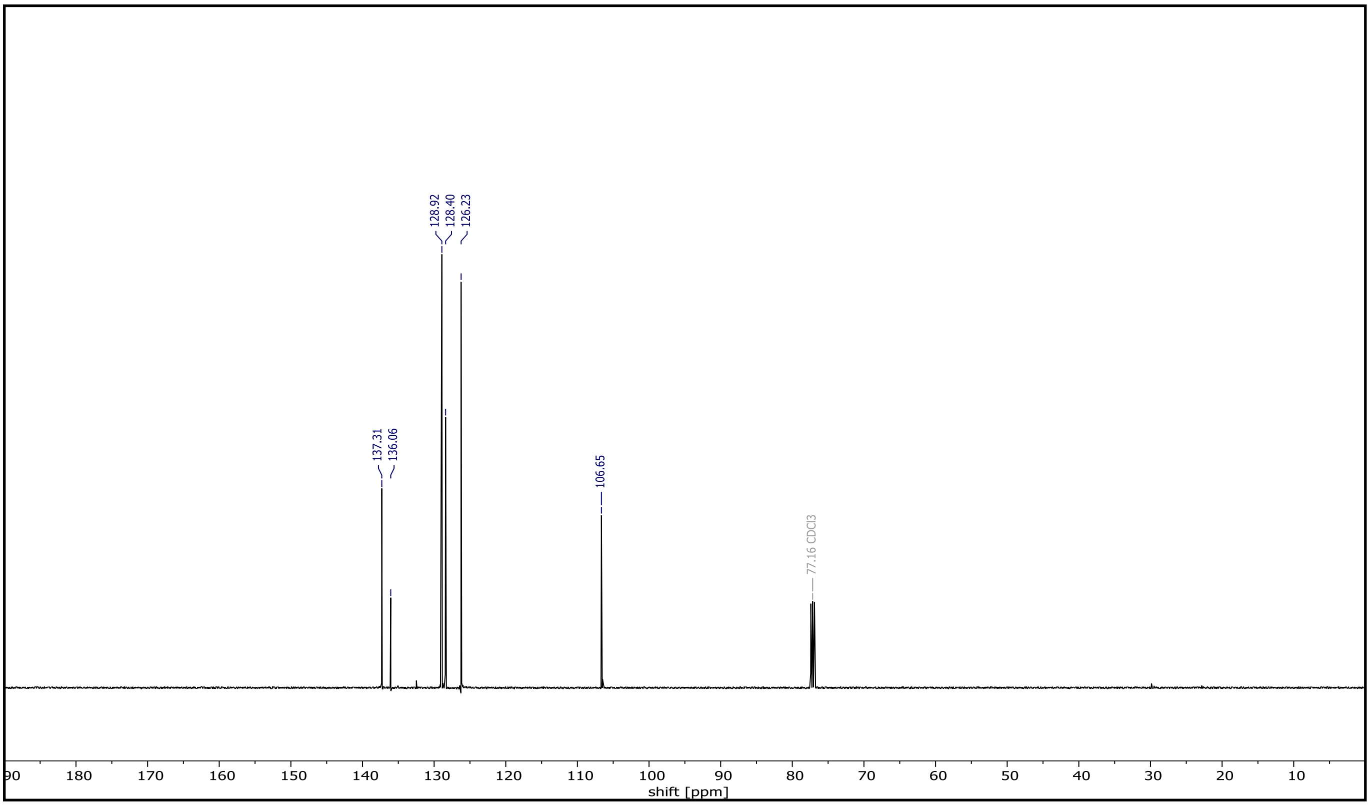

(*Z*)-(2-Bromovinyl) (*Z*)-7

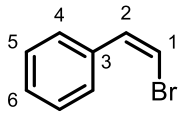

^{1}H NMR (599 MHz, $CDCl_3$)

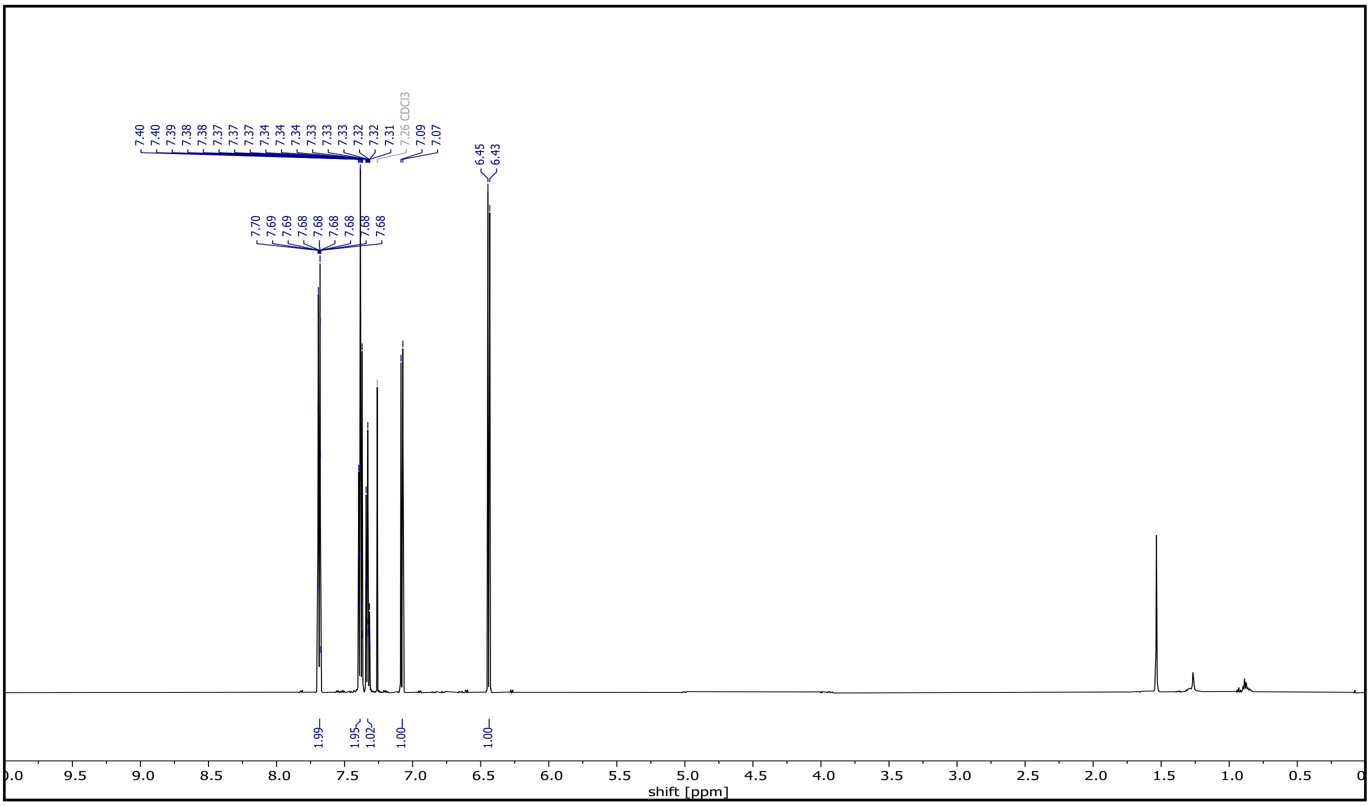

^{13}C{^{1}H} NMR (151 MHz, $CDCl_3$)

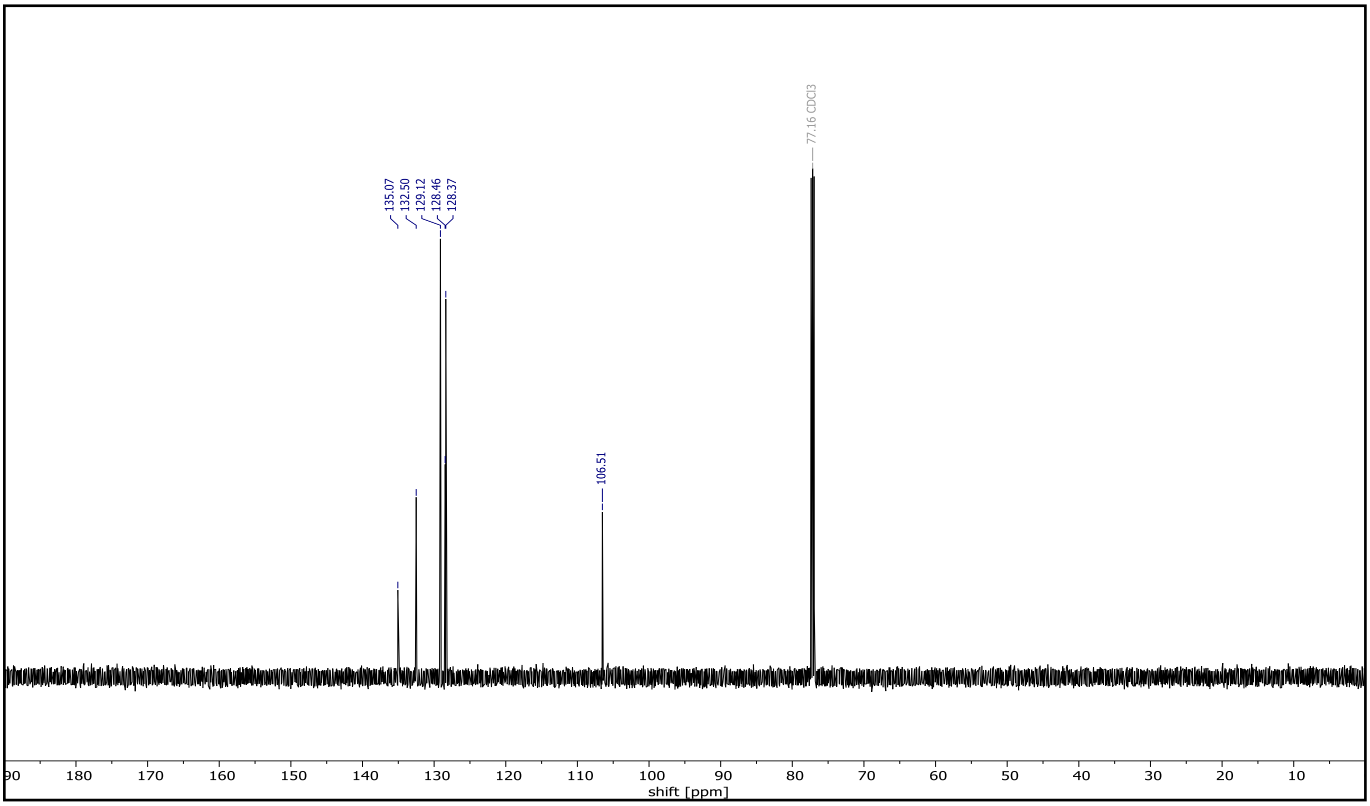

(*E*,*Z*)-1-(2-Bromovinyl)-4-(*tert*-butyl)benzene (*E*,*Z*)-8

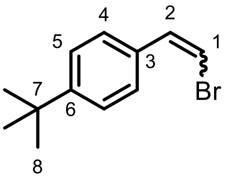

^{1}H NMR (400 MHz, $CDCl_3$), experiment 1

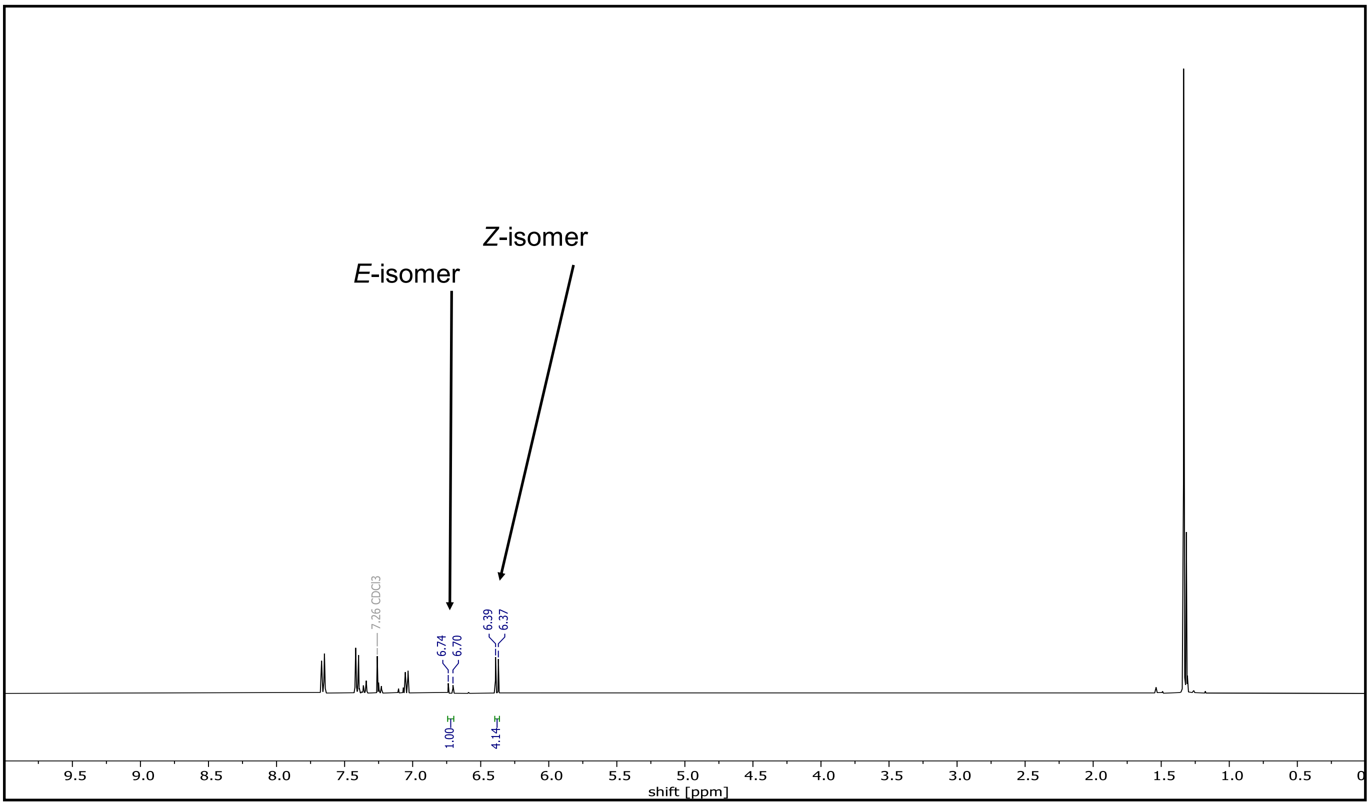

^{1}H NMR (400 MHz, $CDCl_3$), experiment 2

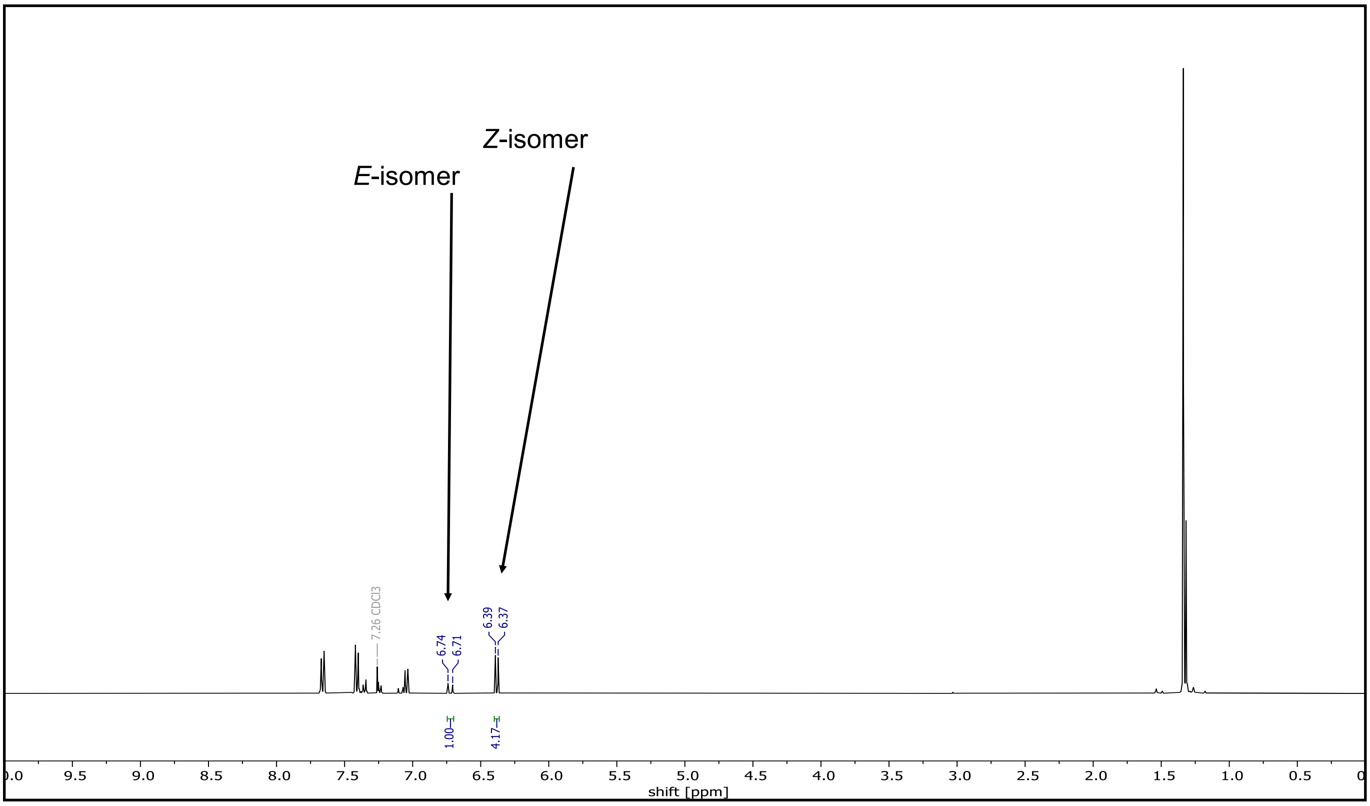

(*E*,*Z*)-1-(2-Bromovinyl)-4-(*tert*-butyl)benzene (*E*,*Z*)-8

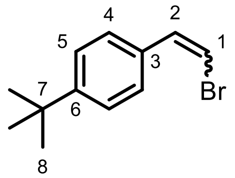

^{1}H NMR ($CDCl_3$), experiment 1

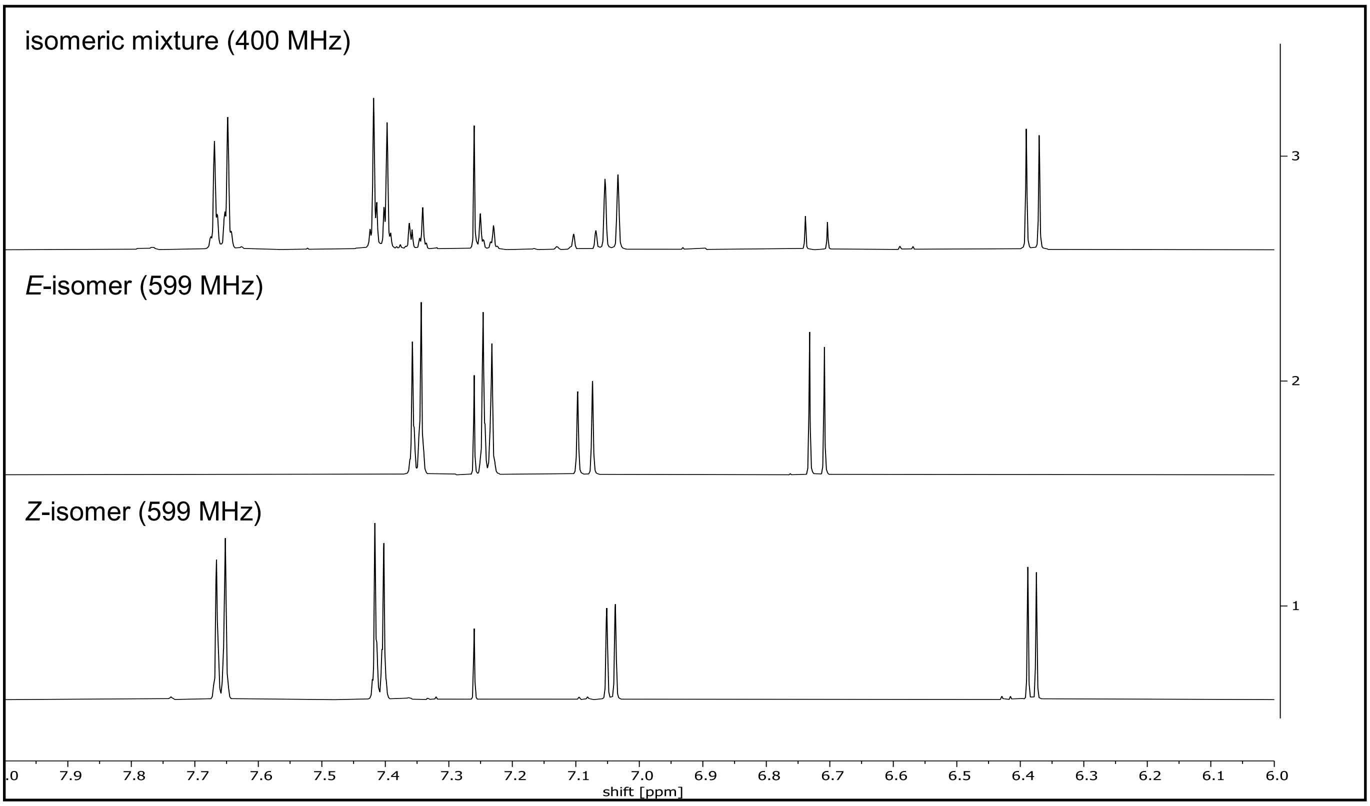

^{1}H NMR ($CDCl_3$), experiment 2

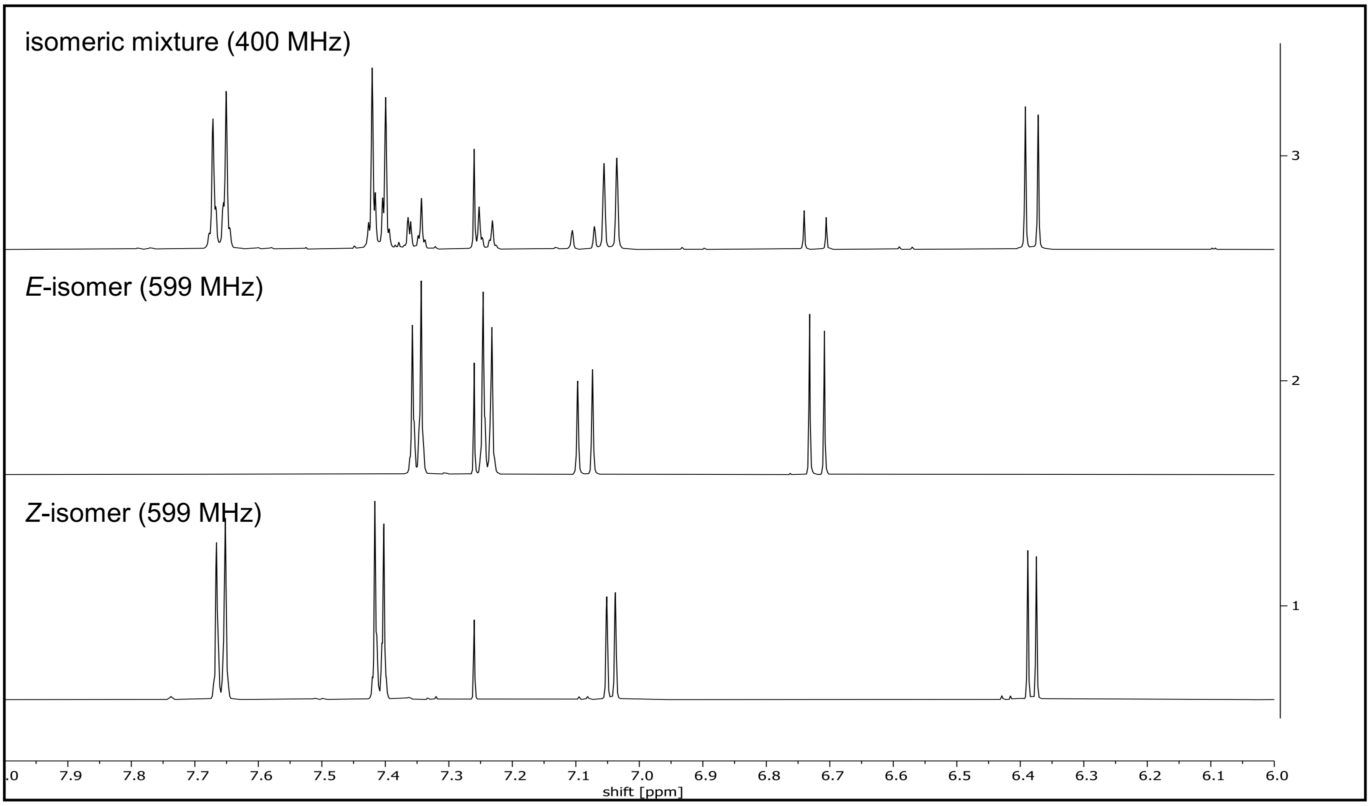

(*E*)-1-(2-Bromovinyl)-4-(*tert*-butyl)benzene (*E*)-8

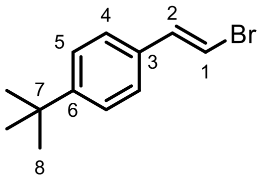

^{1}H NMR (599 MHz, $CDCl_3$)

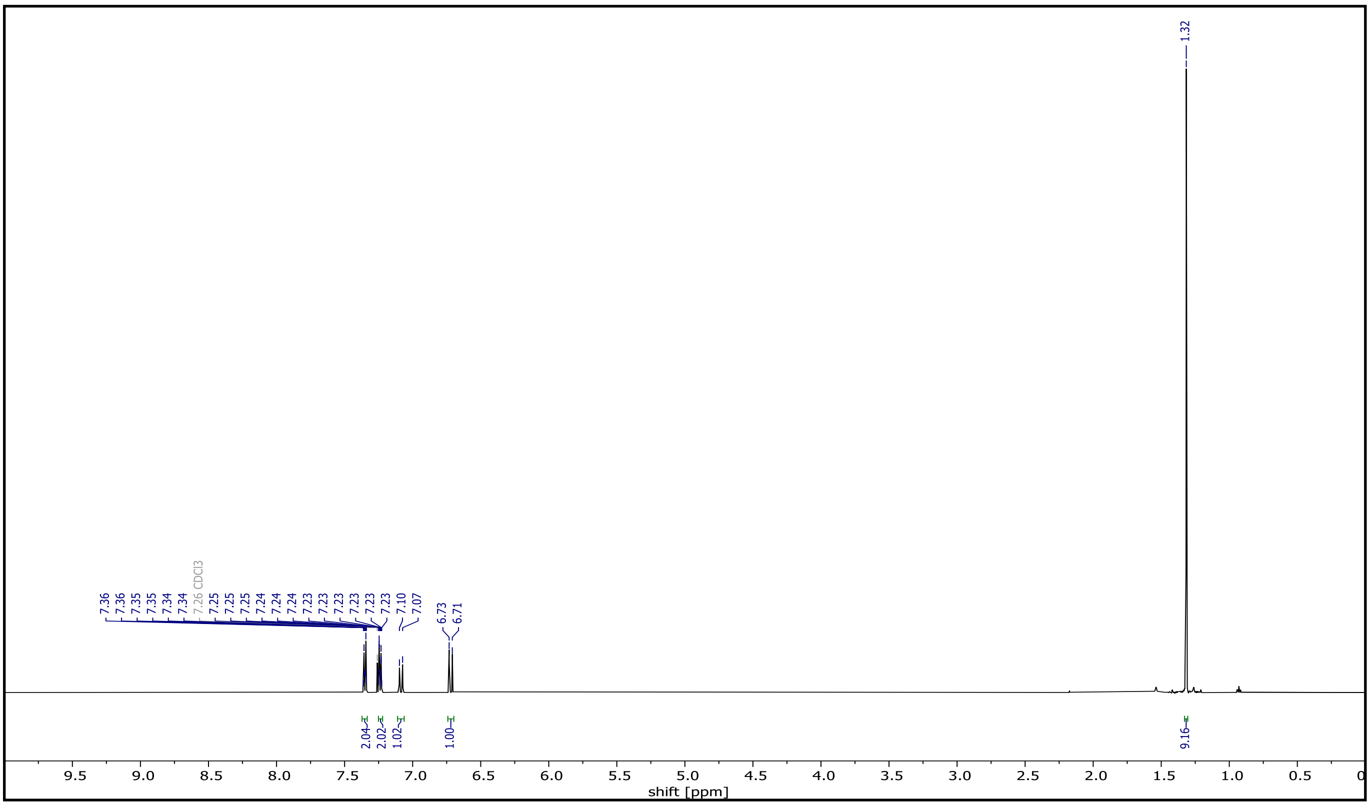

$^{13}C\{^{1}H\}$ NMR (151 MHz, $CDCl_3$)

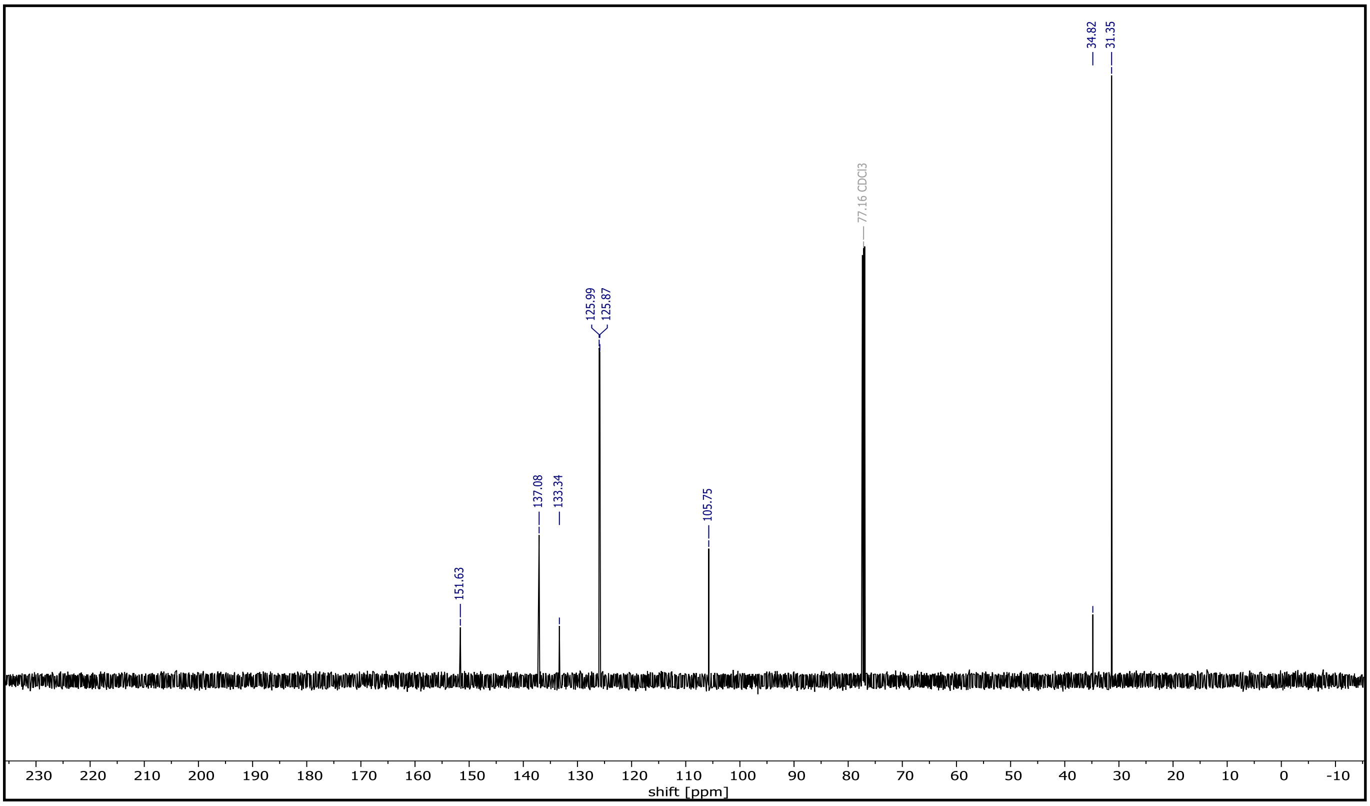

(*Z*)-1-(2-Bromovinyl)-4-(*tert*-butyl)benzene (*Z*)-8

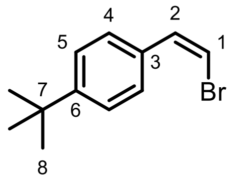

^{1}H NMR (599 MHz, $CDCl_3$)

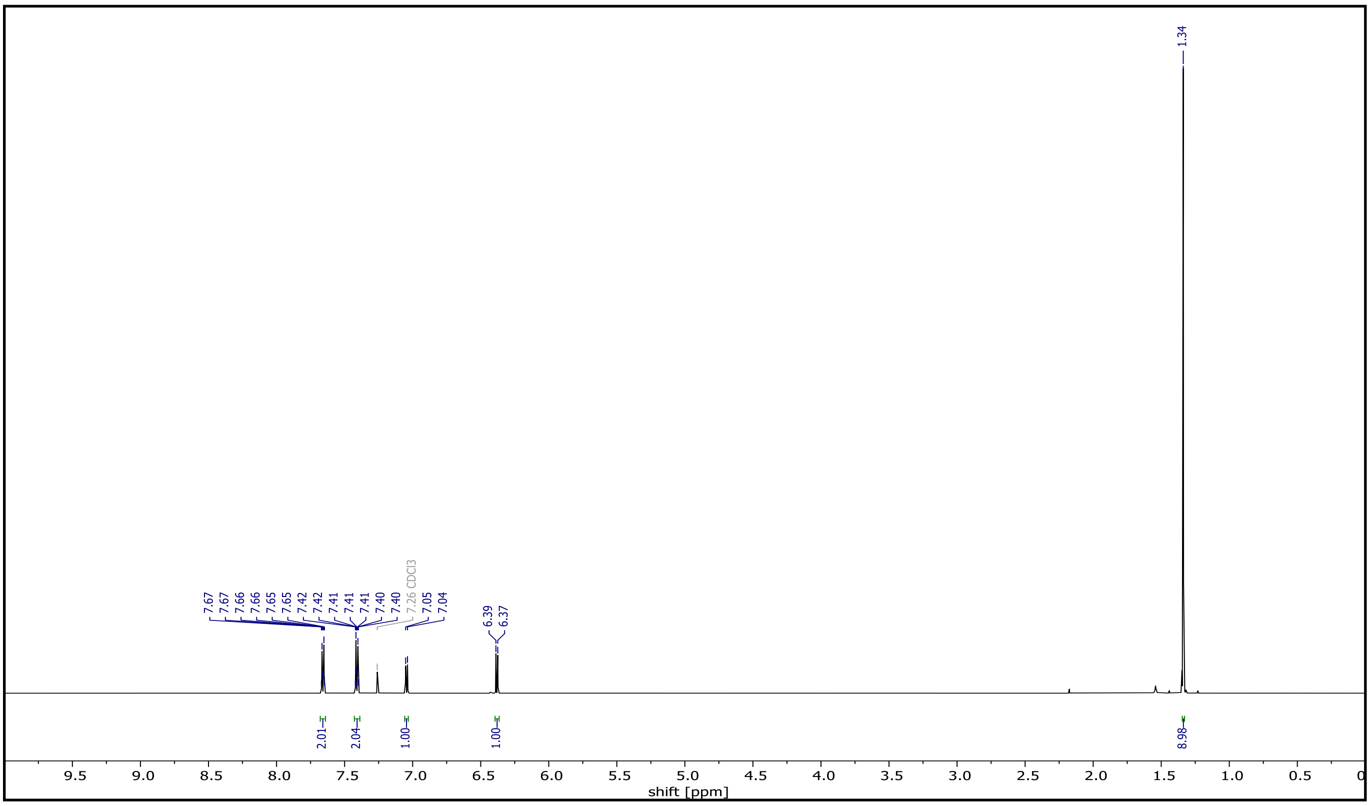

^{13}C{^{1}H} NMR (151 MHz, $CDCl_3$)

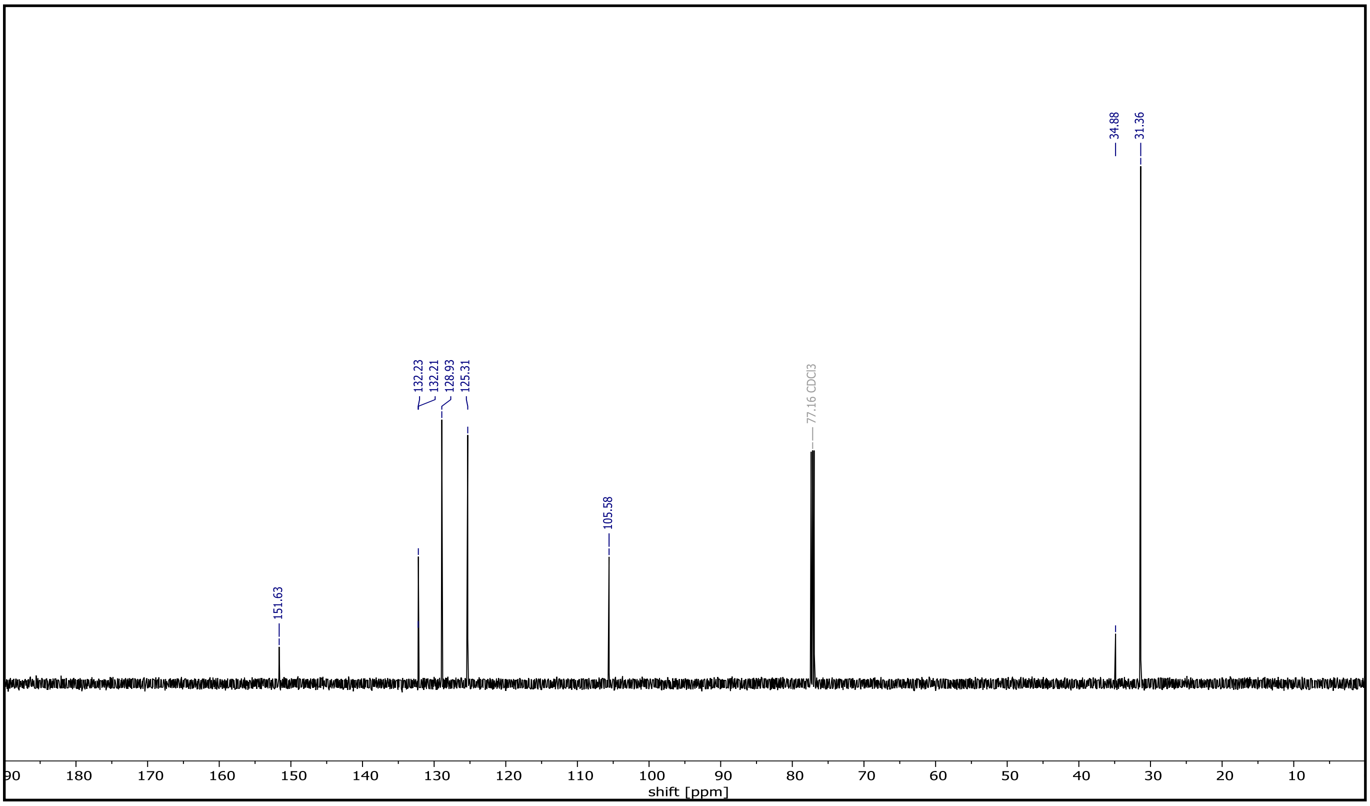

(*E*,*Z*)-4-(2-Bromovinyl)-1,1'-biphenyl (*E*,*Z*)-9

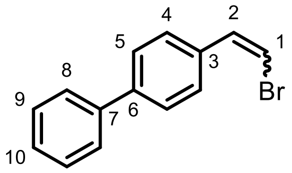

^{1}H NMR (400 MHz, $CDCl_3$), experiment 1

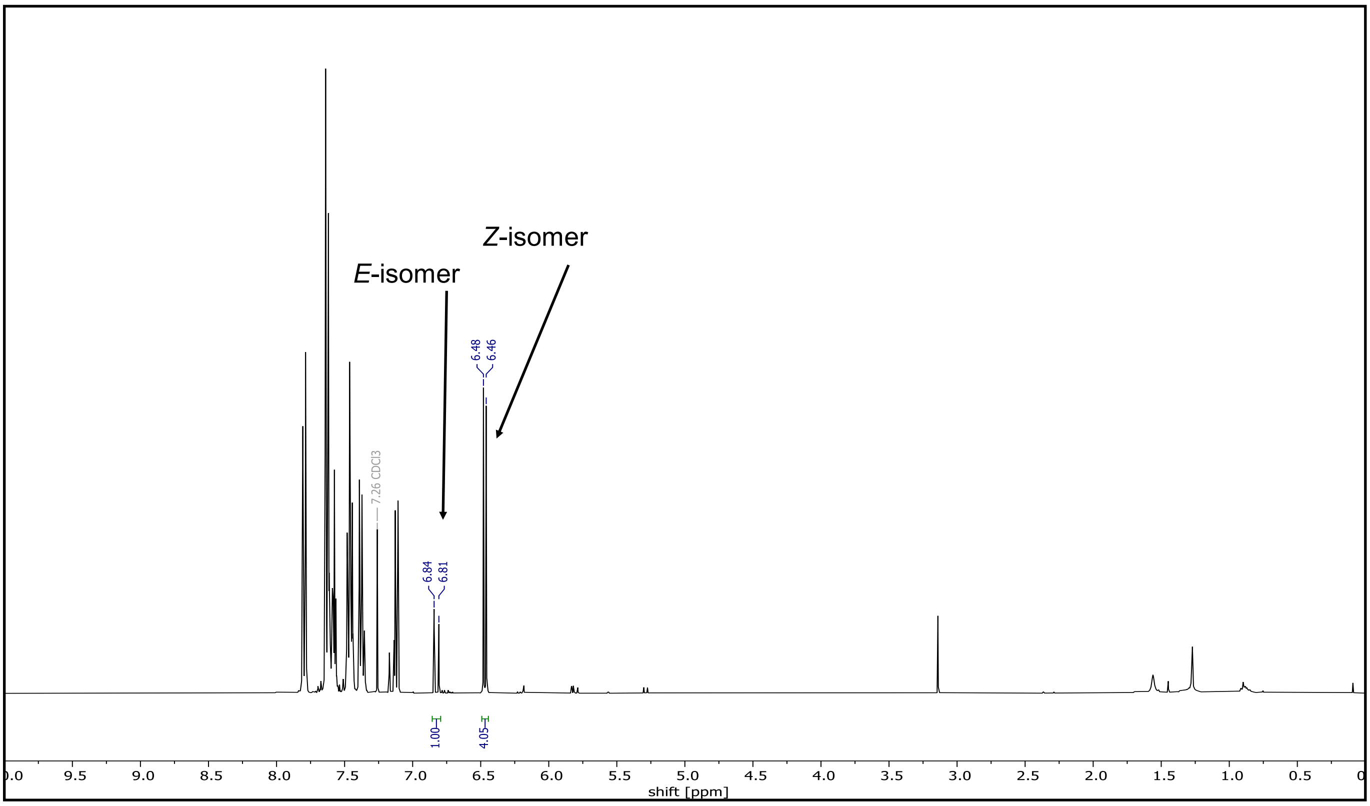

^{1}H NMR (400 MHz, $CDCl_3$), experiment 2

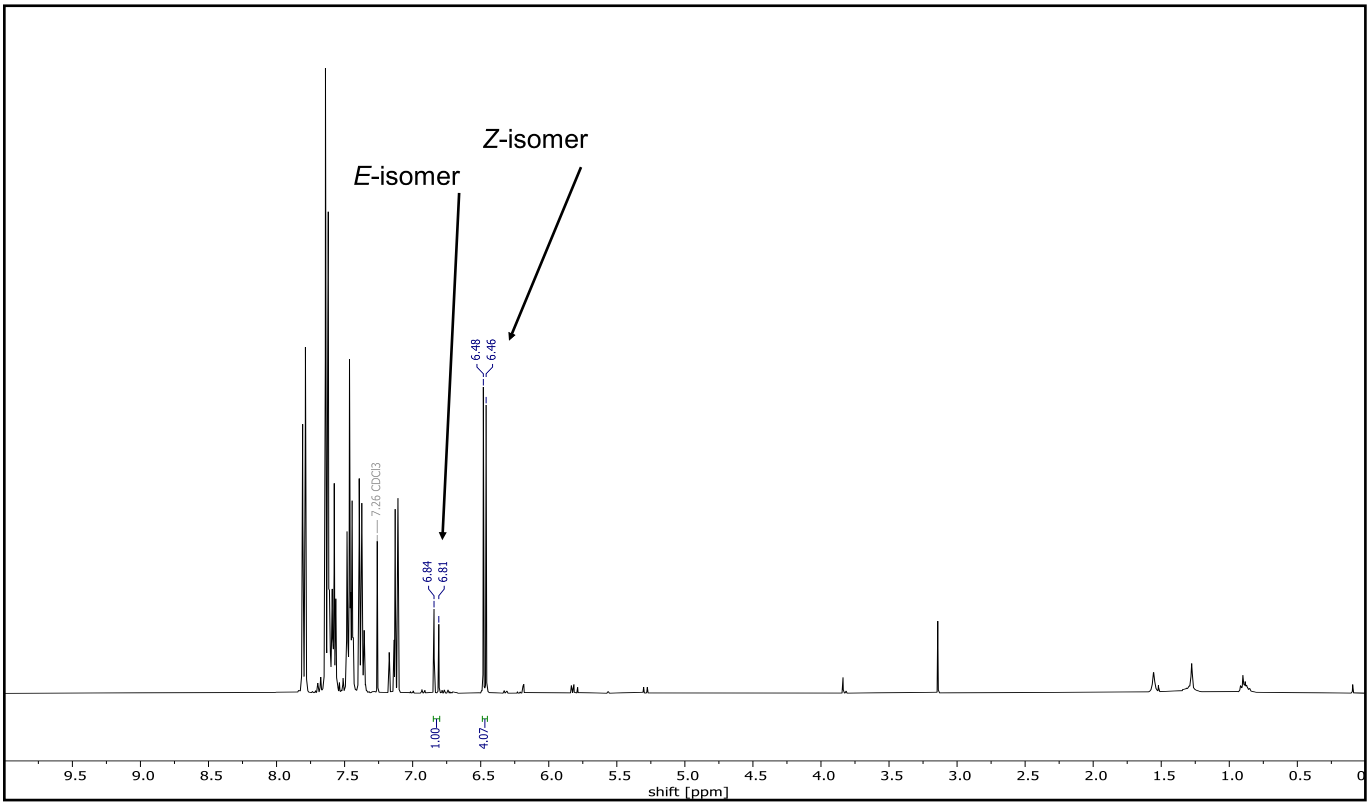

(*E*,*Z*)-4-(2-Bromovinyl)-1,1'-biphenyl (*E*,*Z*)-9

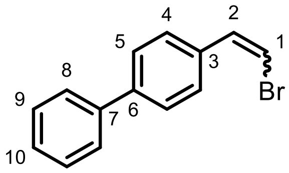

^{1}H NMR ($CDCl_3$), experiment 1

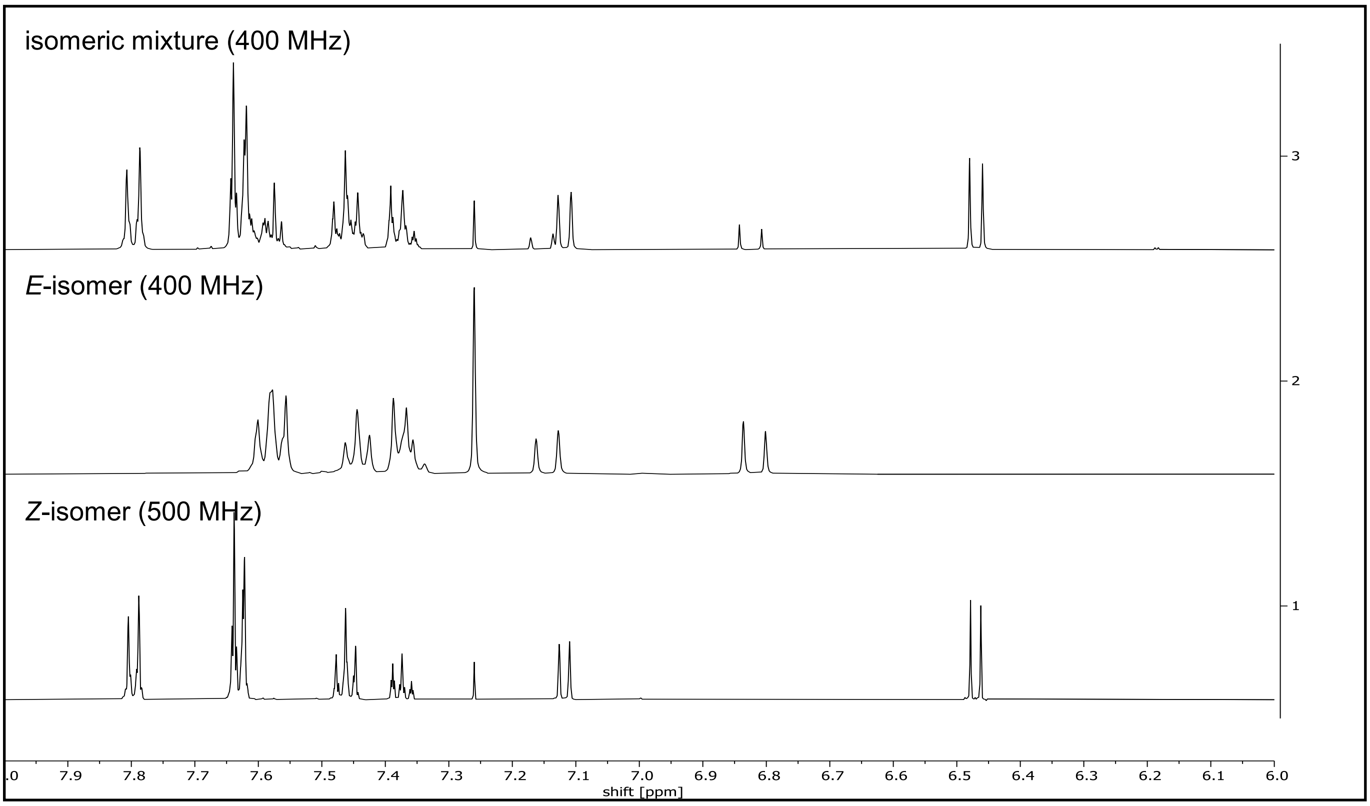

^{1}H NMR ($CDCl_3$), experiment 2

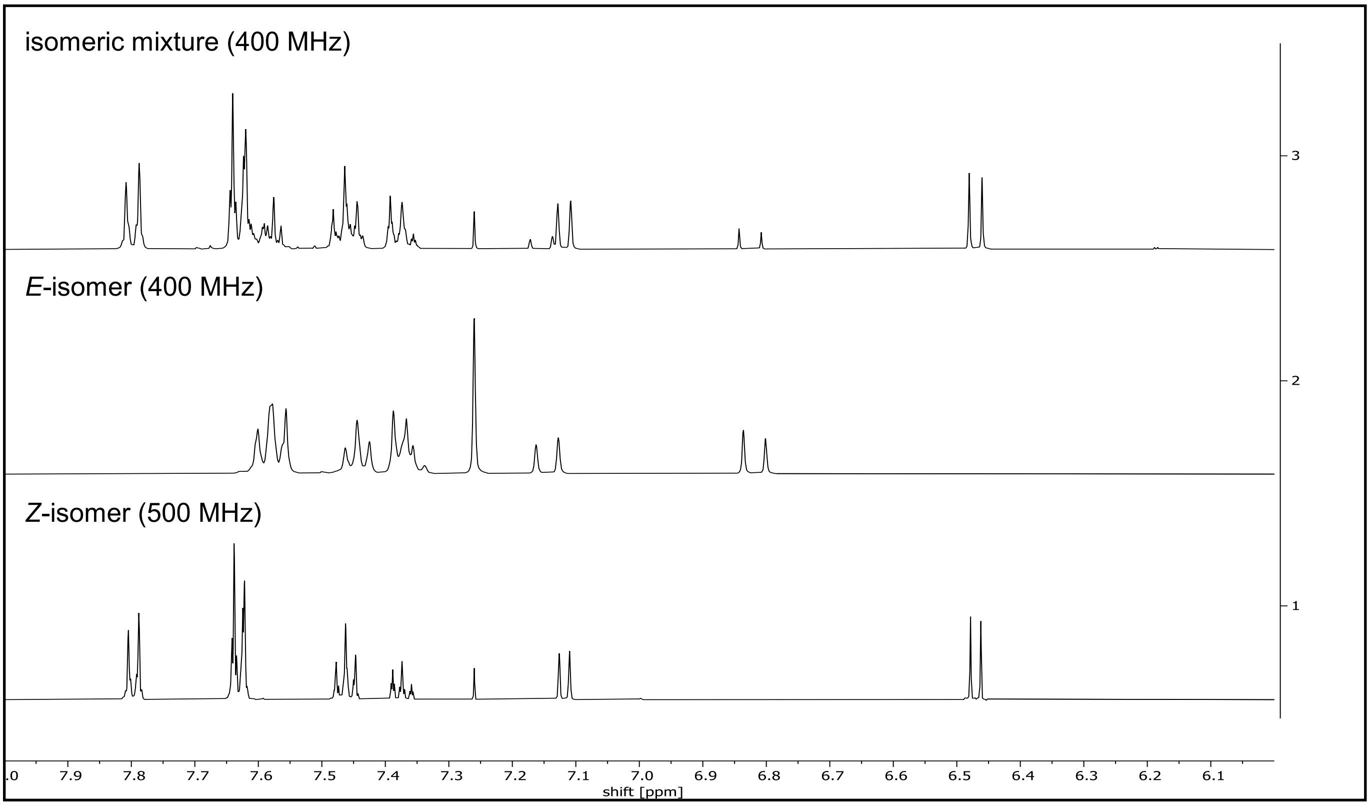

(*E*)-4-(2-Bromovinyl)-1,1‘-biphenyl (*E*)-9

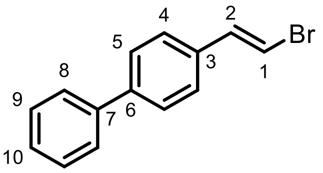

^{1}H NMR (400 MHz, $CDCl_3$)

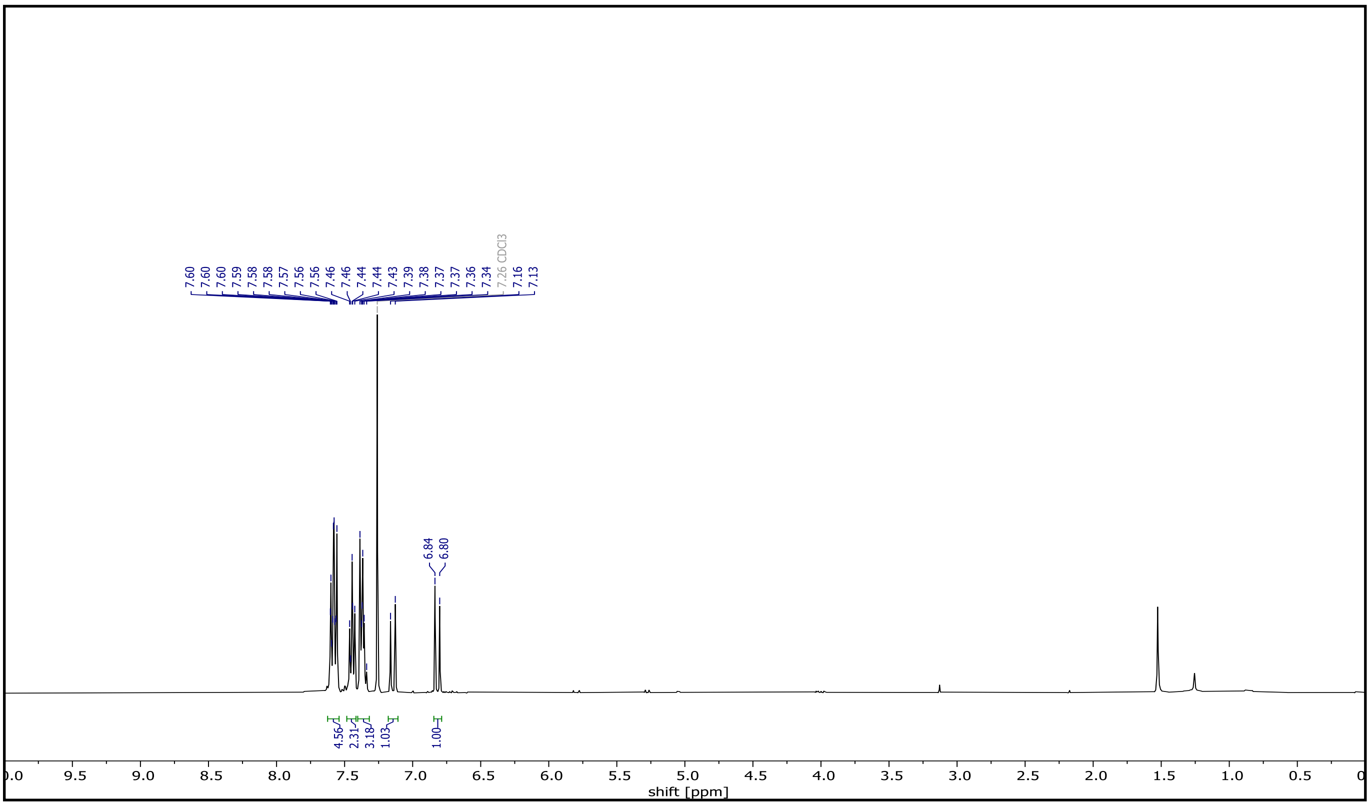

$^{13}C\{^{1}H\}$ NMR (151 MHz, $CDCl_3$)

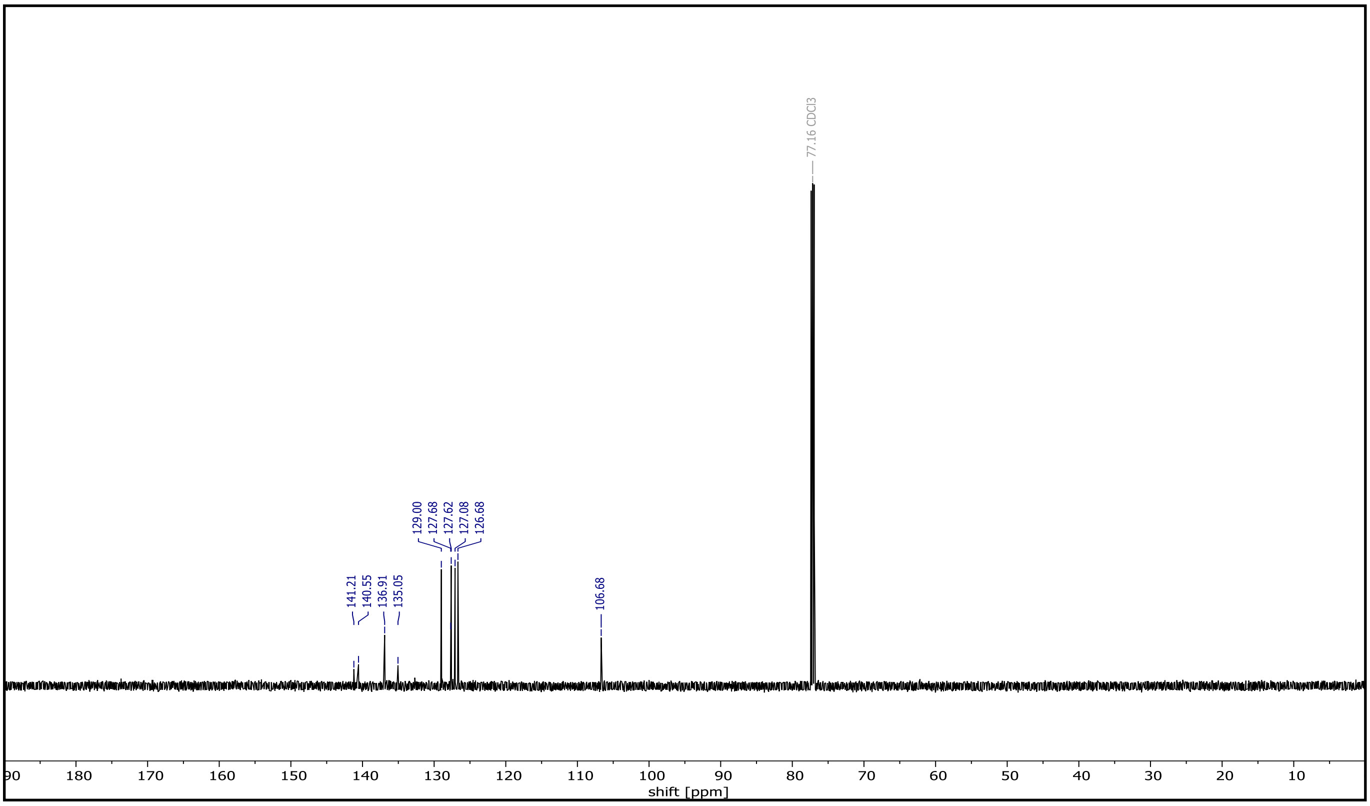

(*Z*)-4-(2-Bromovinyl)-1,1'-biphenyl (*Z*)-9

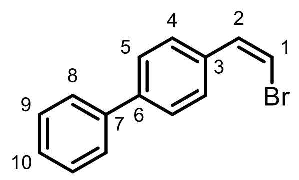

^{1}H NMR (500 MHz, $CDCl_3$)

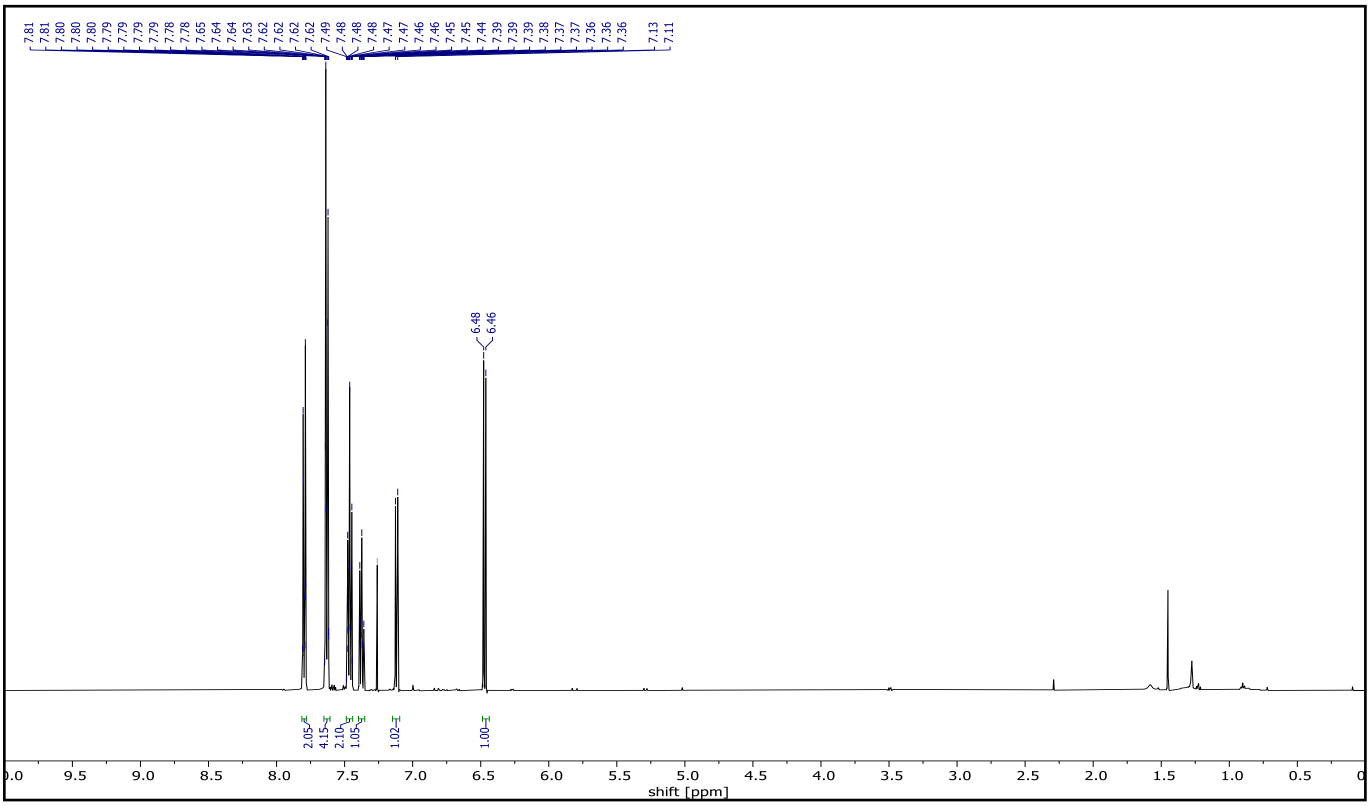

$^{13}C\{^{1}H\}$ NMR (126 MHz, $CDCl_3$)

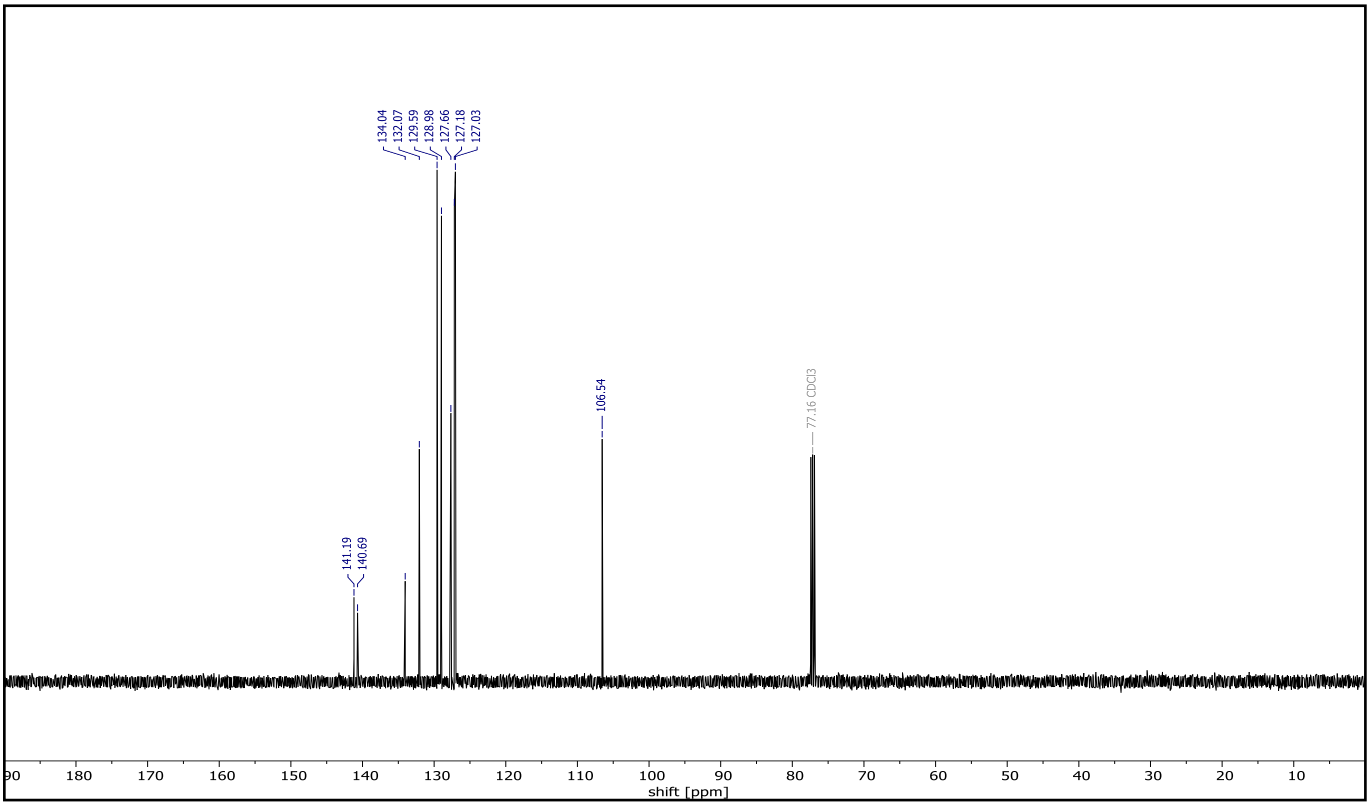

(*E*,*Z*)-1-(2-Bromovinyl)-4-methylbenzene (*E*,*Z*)-10

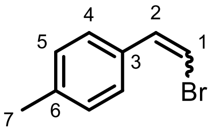

^{1}H NMR (400 MHz, $CDCl_3$), experiment 1

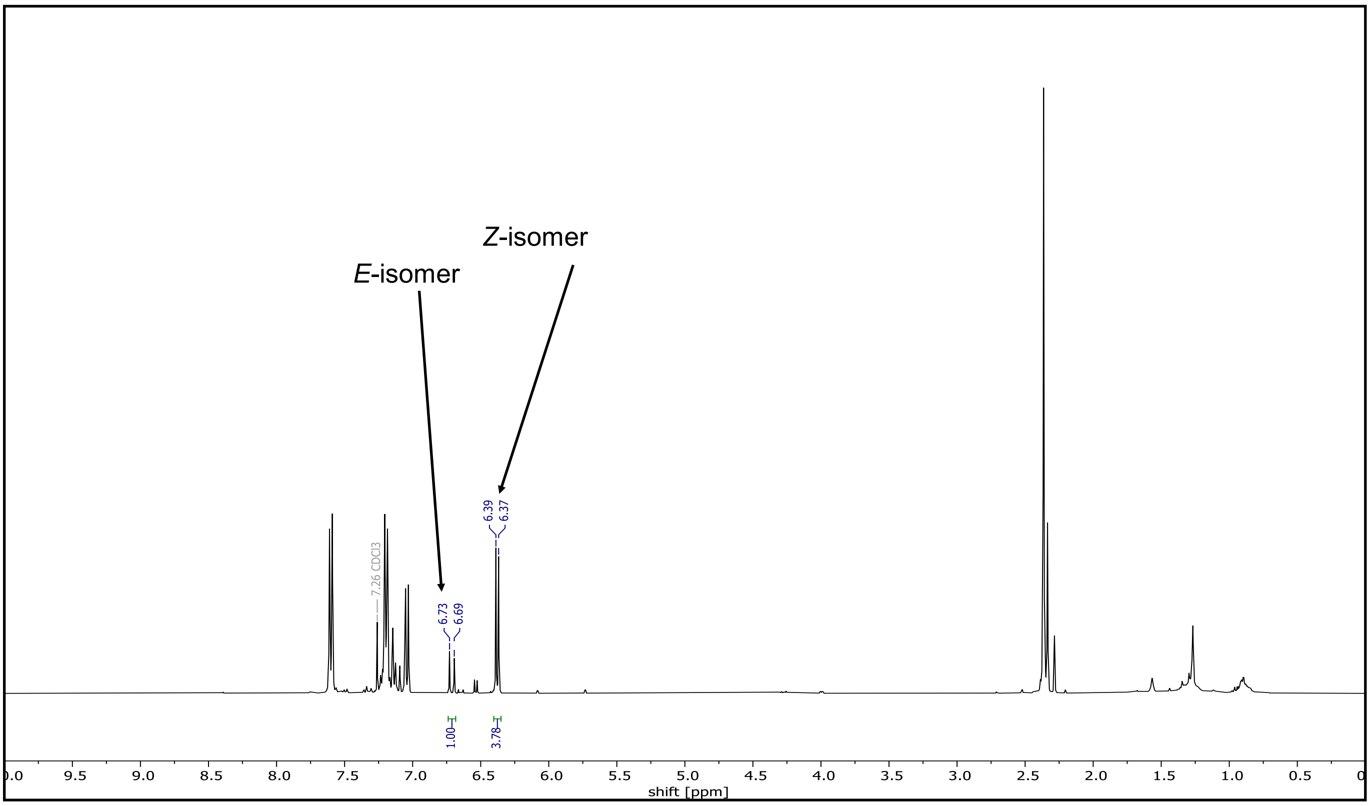

^{1}H NMR (400 MHz, $CDCl_3$), experiment 2

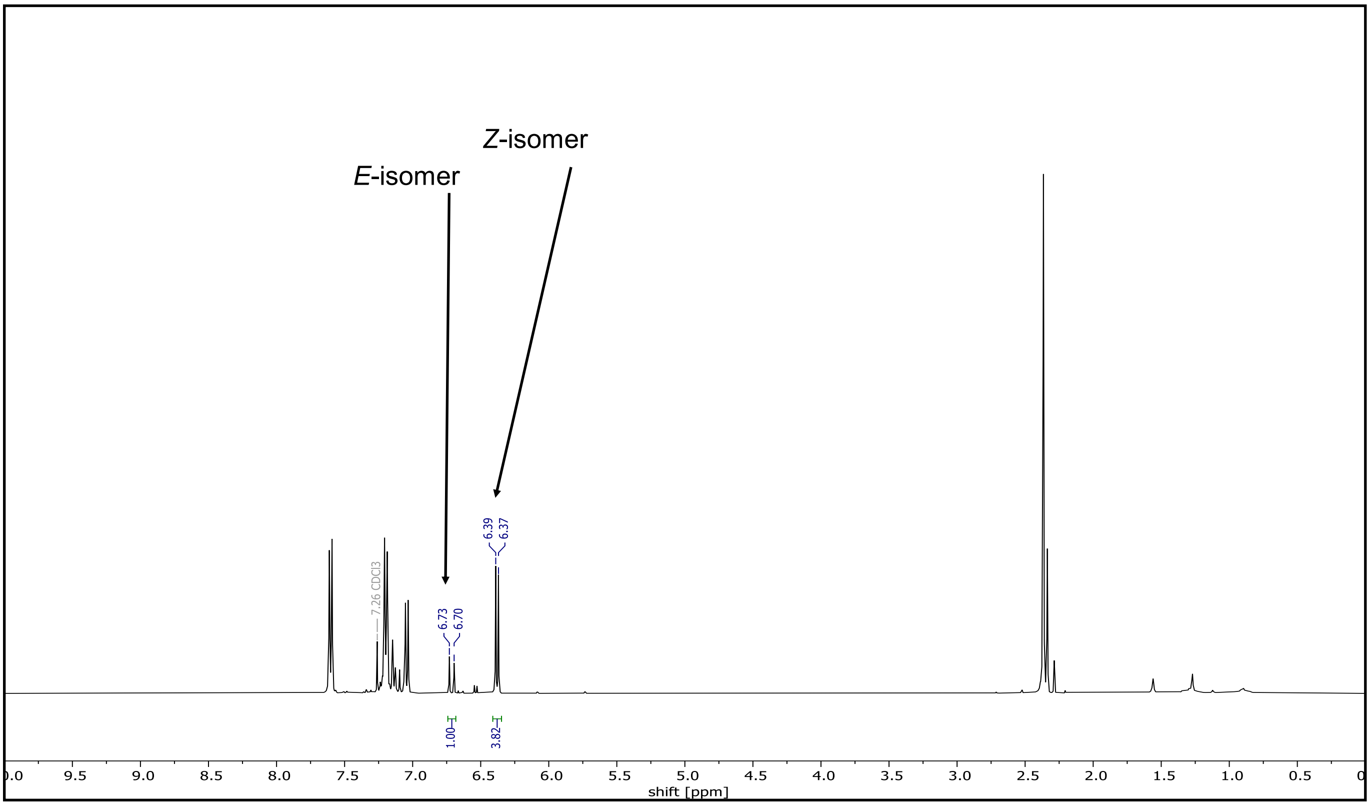

(*E*,*Z*)-1-(2-Bromovinyl)-4-methylbenzene (*E*,*Z*)-10

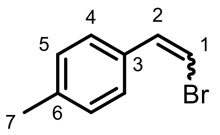

^{1}H NMR ($CDCl_3$), experiment 1

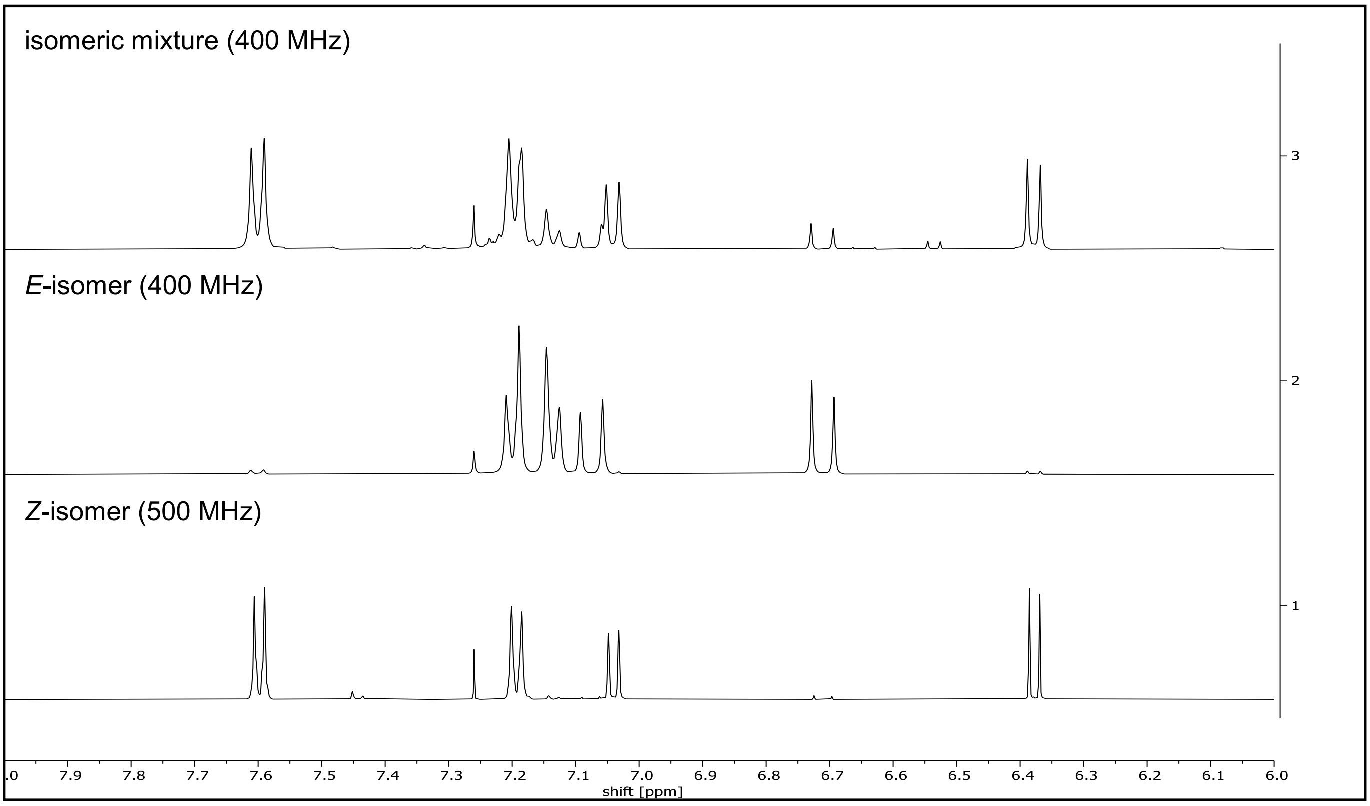

^{1}H NMR ($CDCl_3$), experiment 2

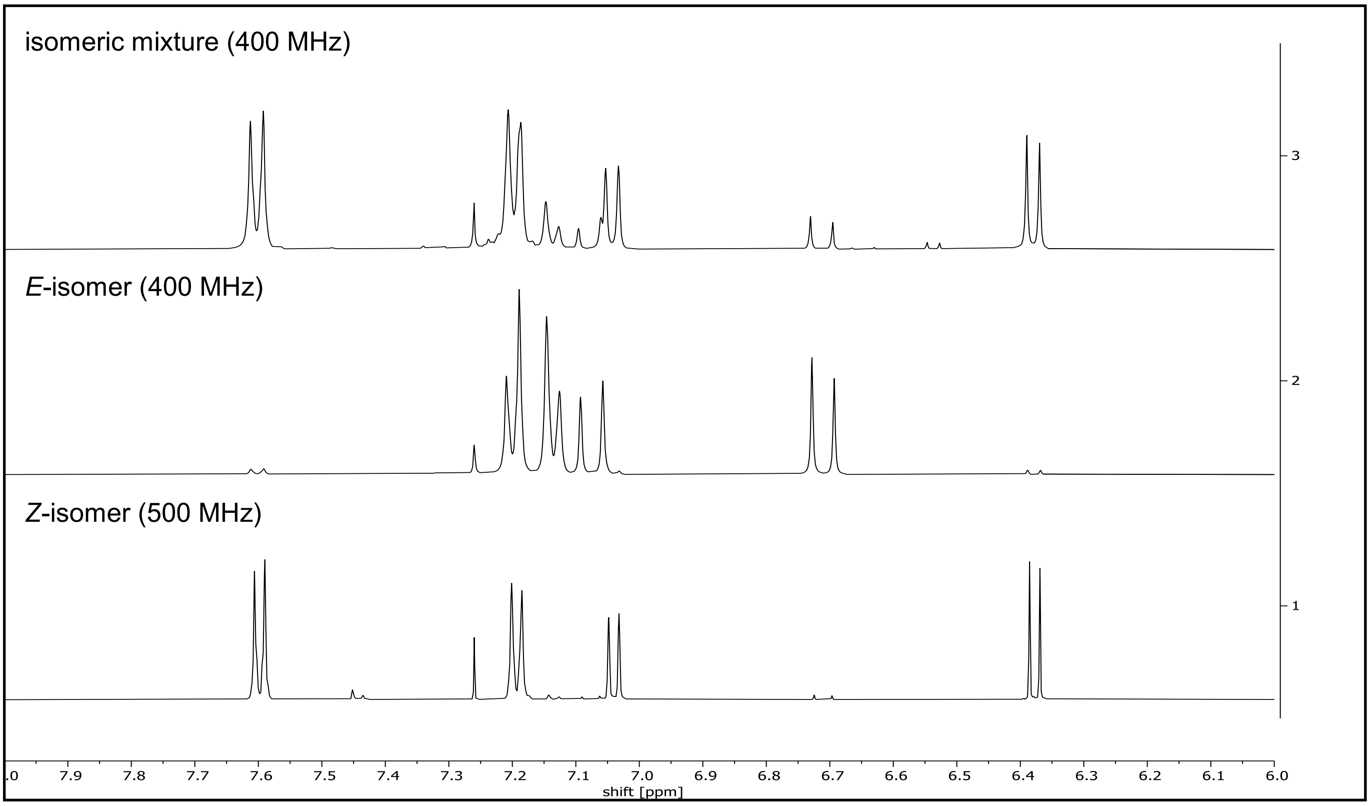

(*E*)-1-(2-Bromovinyl)-4-methylbenzene (*E*)-10

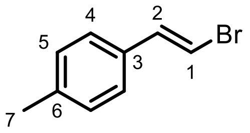

^{1}H NMR (400 MHz, $CDCl_3$)

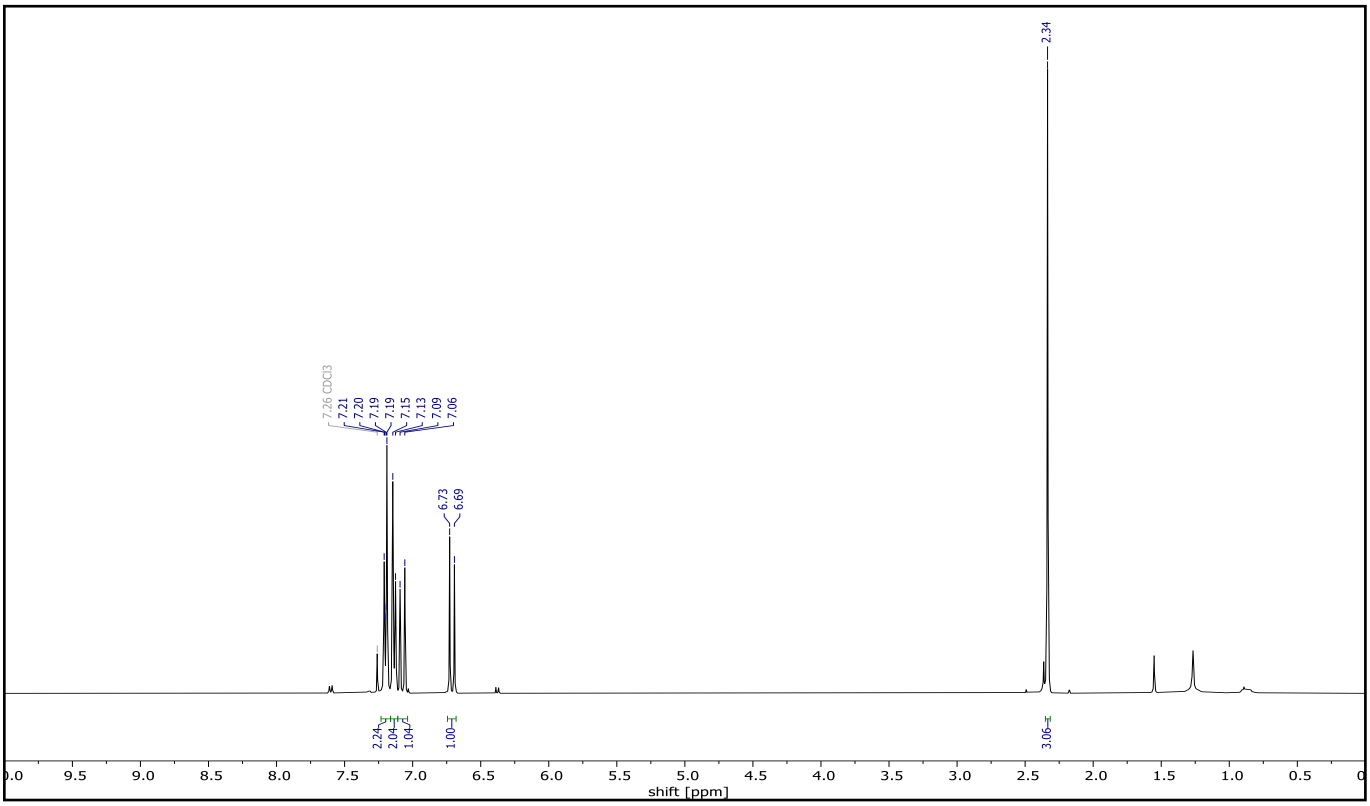

^{13}C NMR (101 MHz, $CDCl_3$)

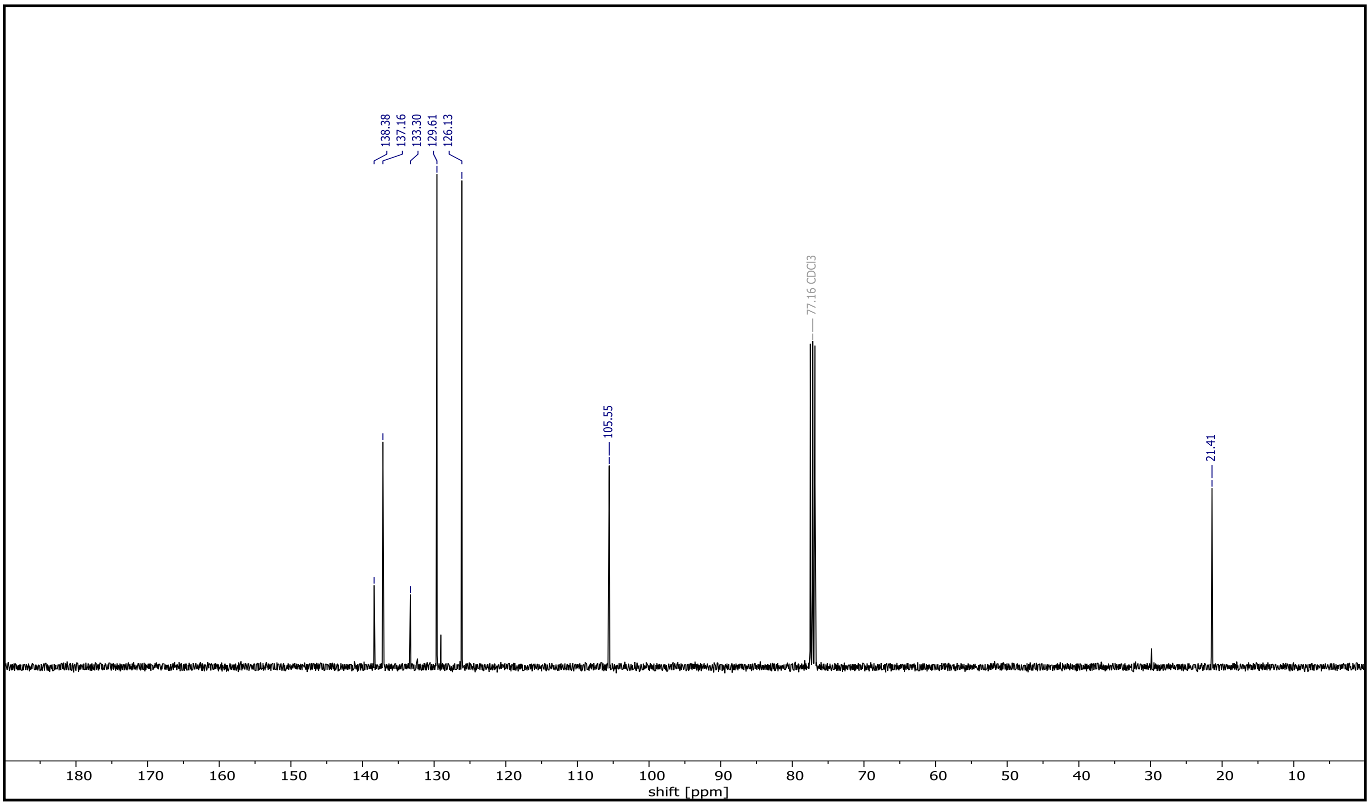

(*Z*)-1-(2-Bromovinyl)-4-methylbenzene (*Z*)-10

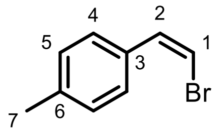

^{1}H NMR (500 MHz, $CDCl_3$)

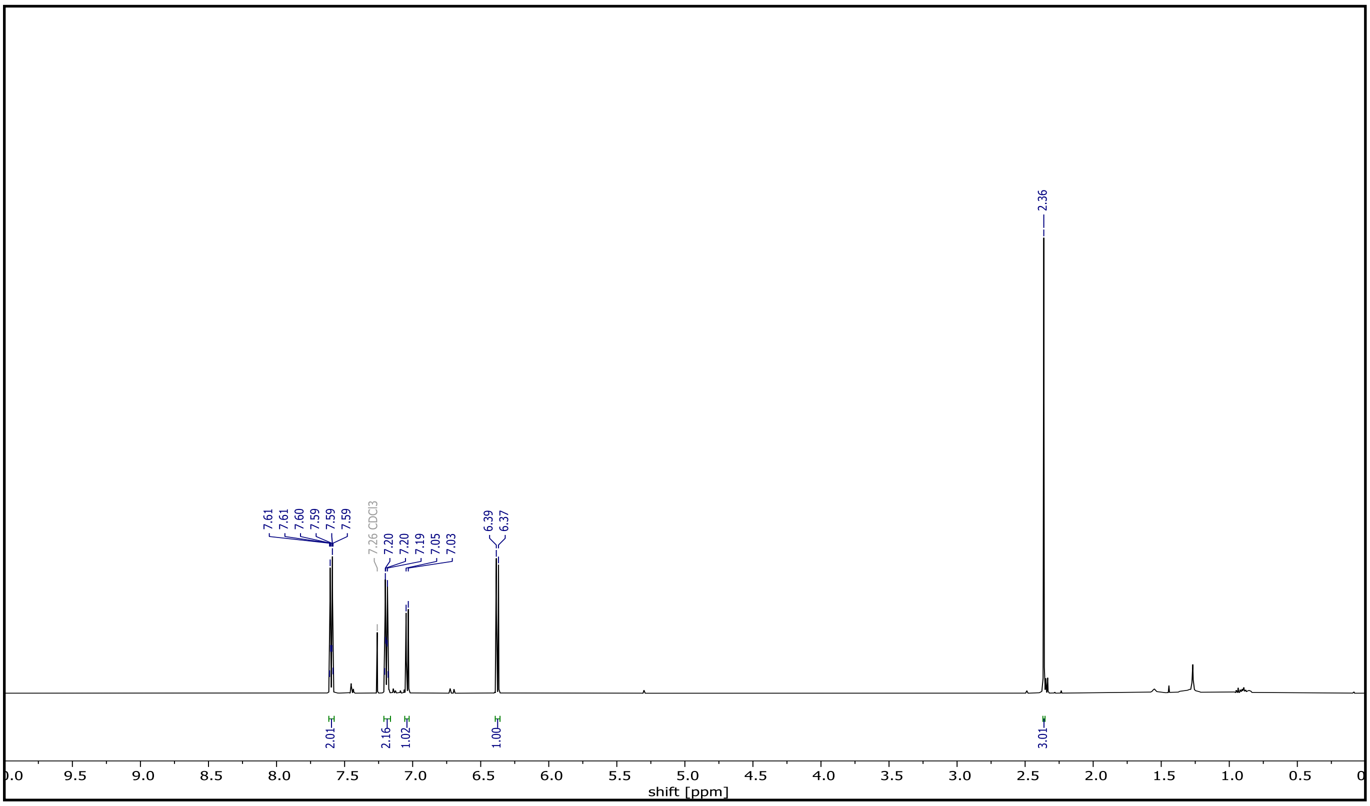

^{13}C NMR (126 MHz, $CDCl_3$)

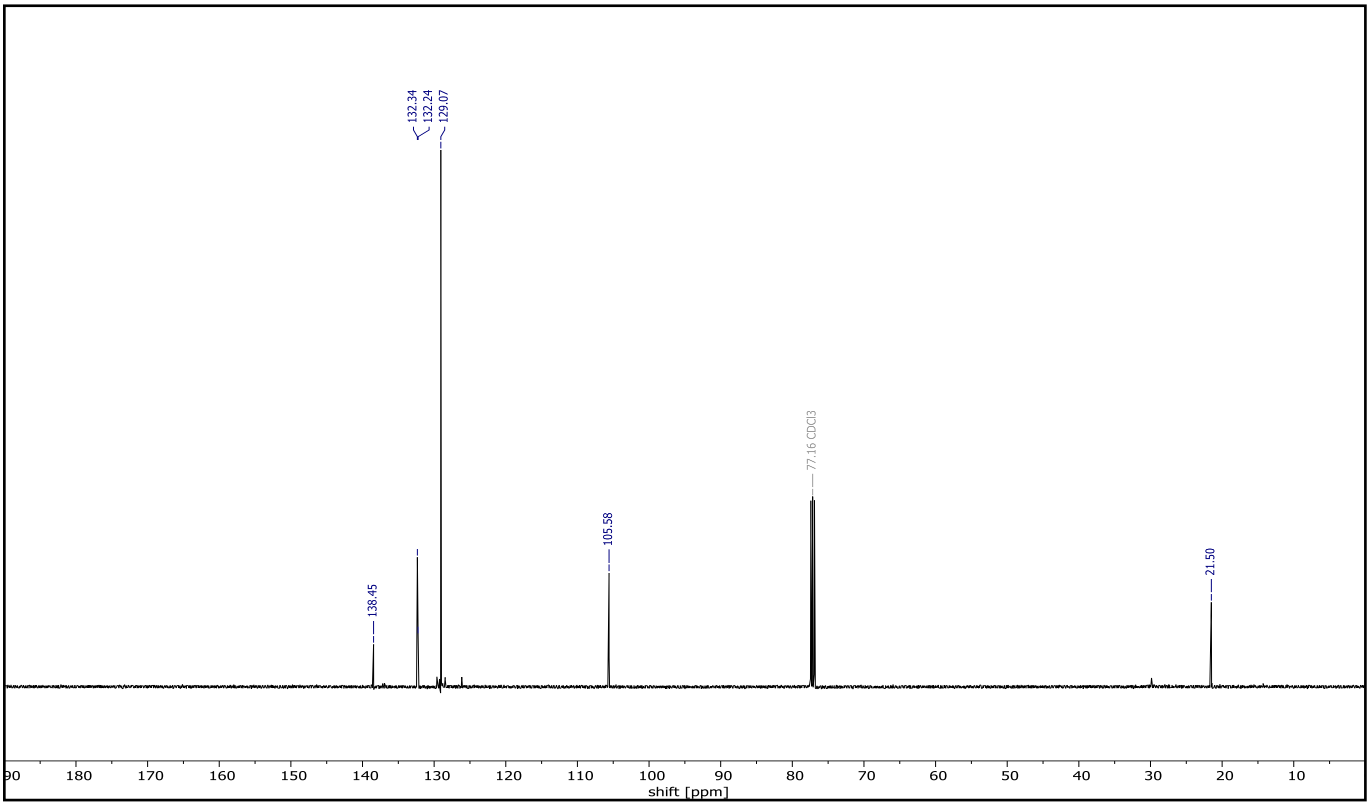

(*E*,*Z*)-1-(2-Bromovinyl)-2-methylbenzene (*E*,*Z*)-11

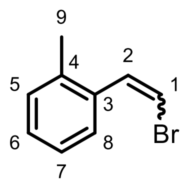

^{1}H NMR (400 MHz, $CDCl_3$), experiment 1

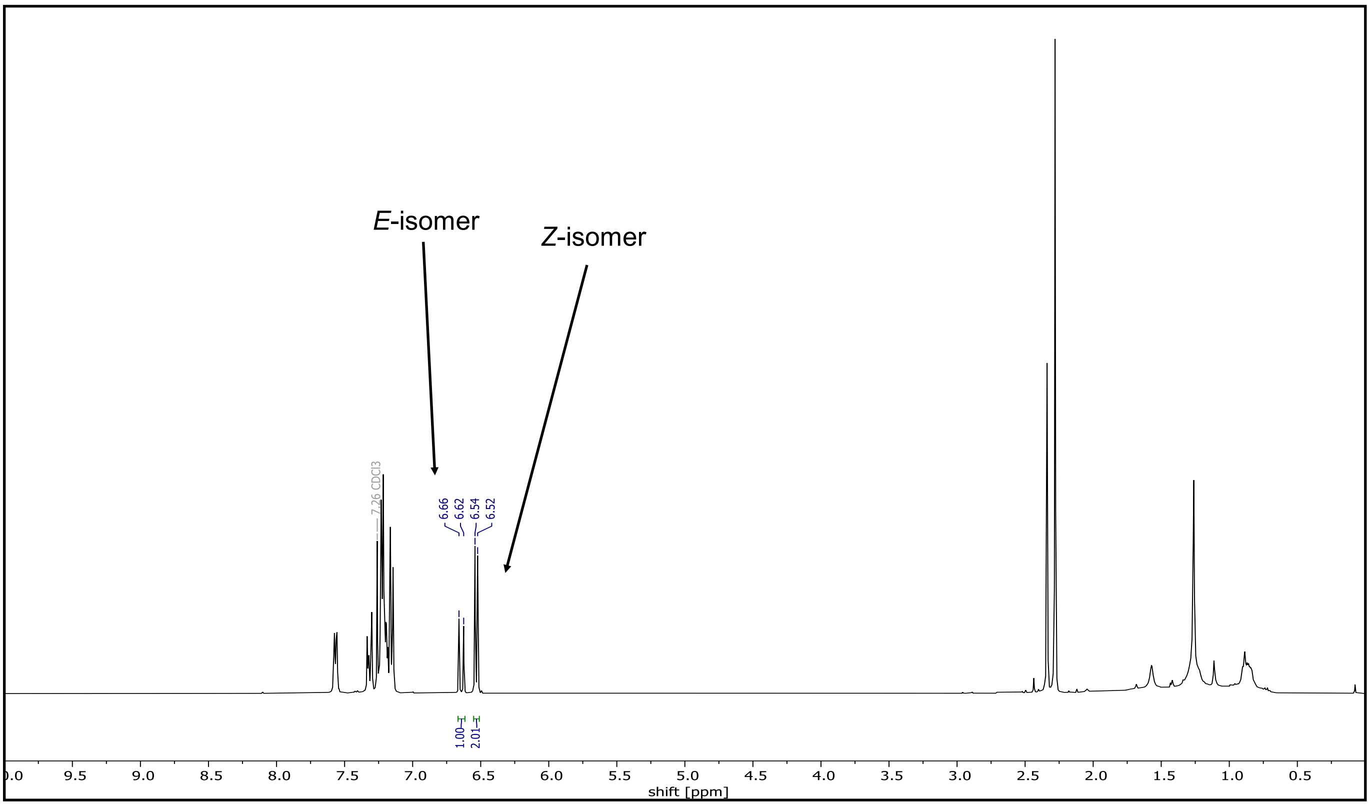

^{1}H NMR (400 MHz, $CDCl_3$), experiment 2

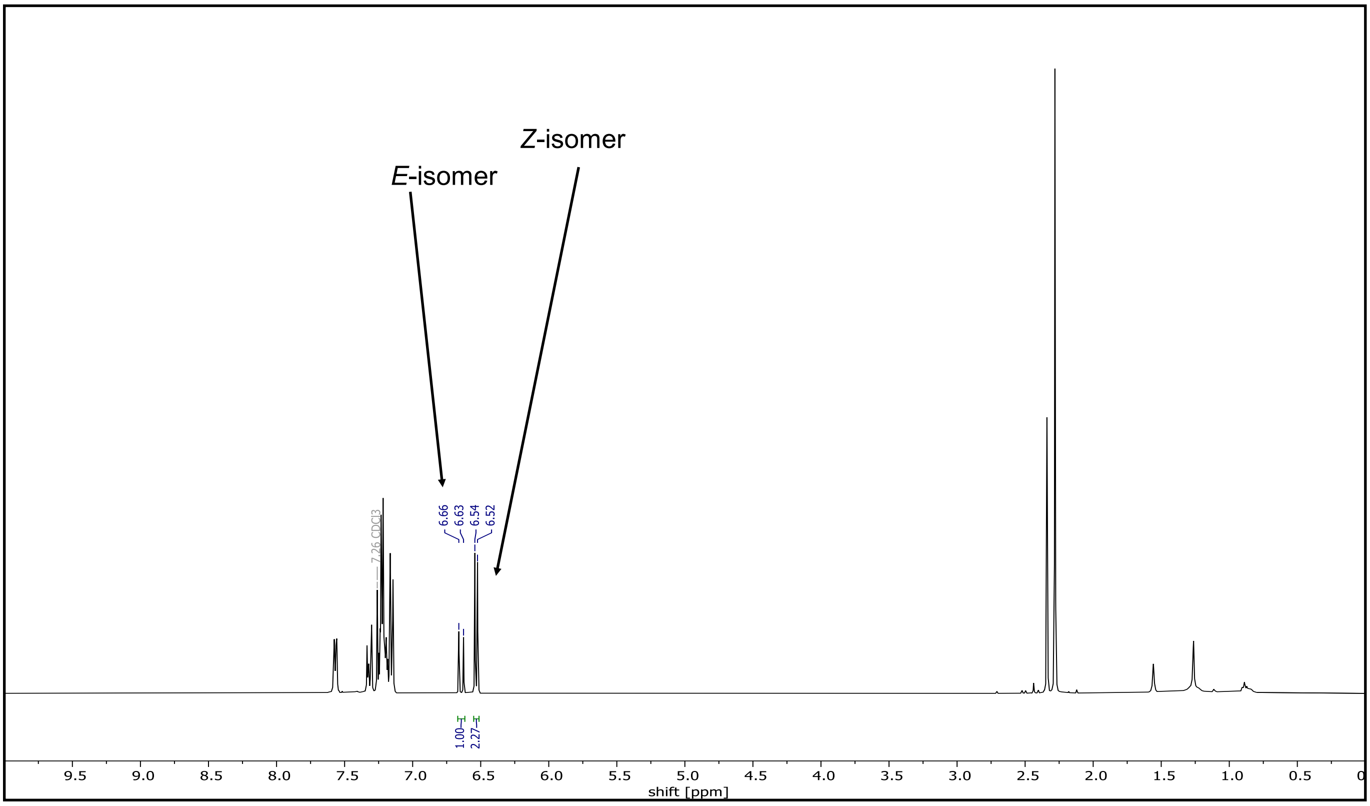

(*E*,*Z*)-1-(2-Bromovinyl)-2-methylbenzene (*E*,*Z*)-11

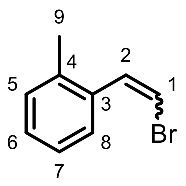

^{1}H NMR ($CDCl_3$), experiment1

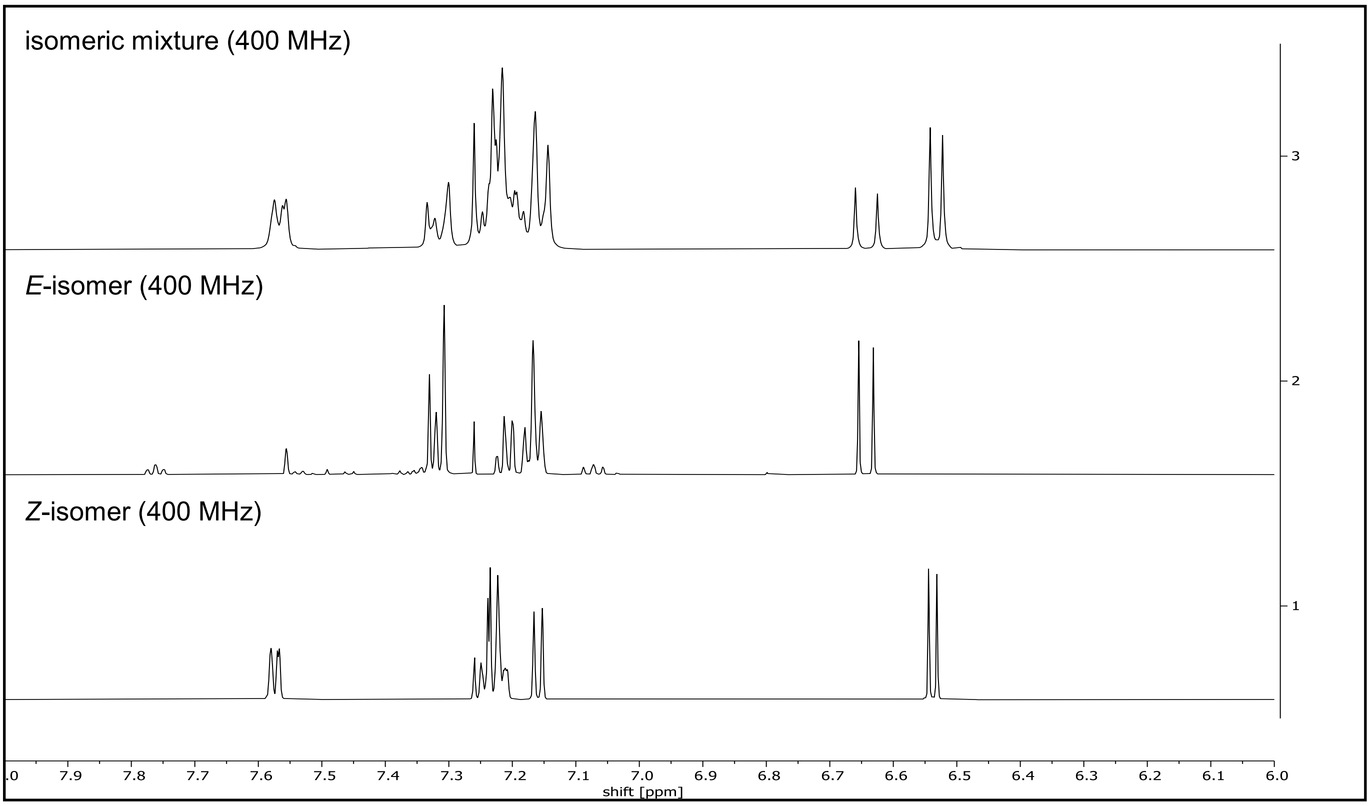

^{1}H NMR ($CDCl_3$), experiment 2

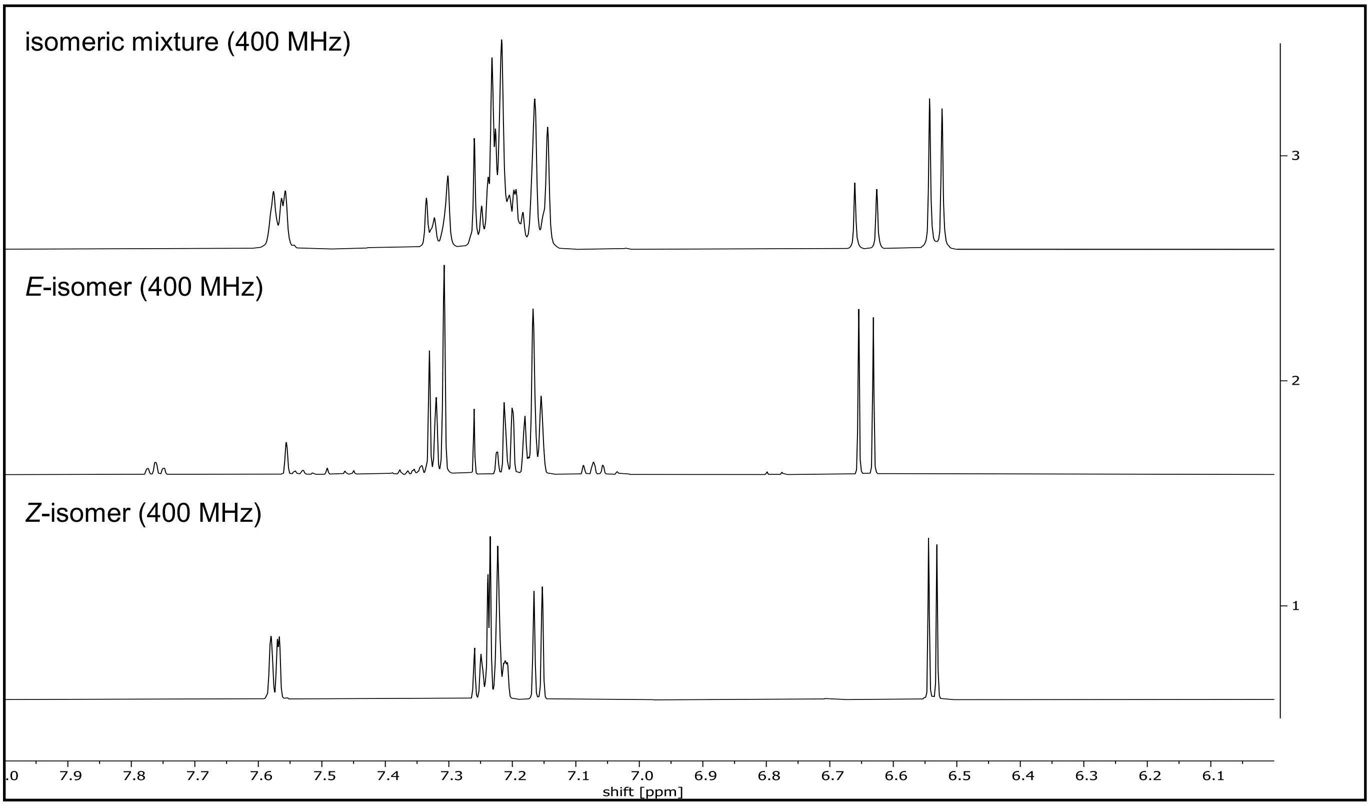

(*E*)-1-(2-Bromovinyl)-2-methylbenzene (*E*)-11

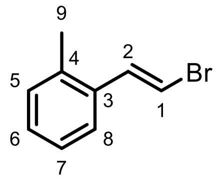

^{1}H NMR (500 MHz, $CDCl_3$)

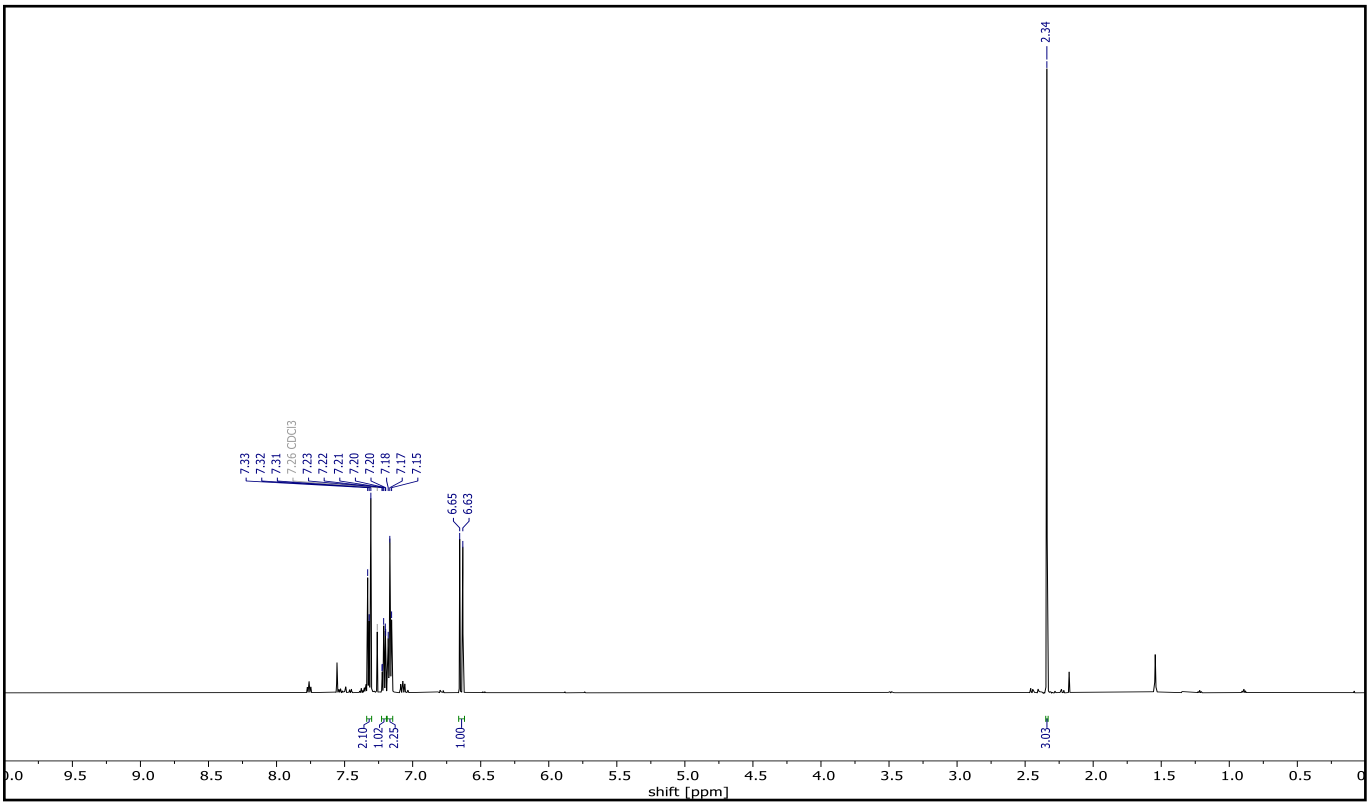

$^{13}C\{^{1}H\}$ NMR (126 MHz, $CDCl_3$)

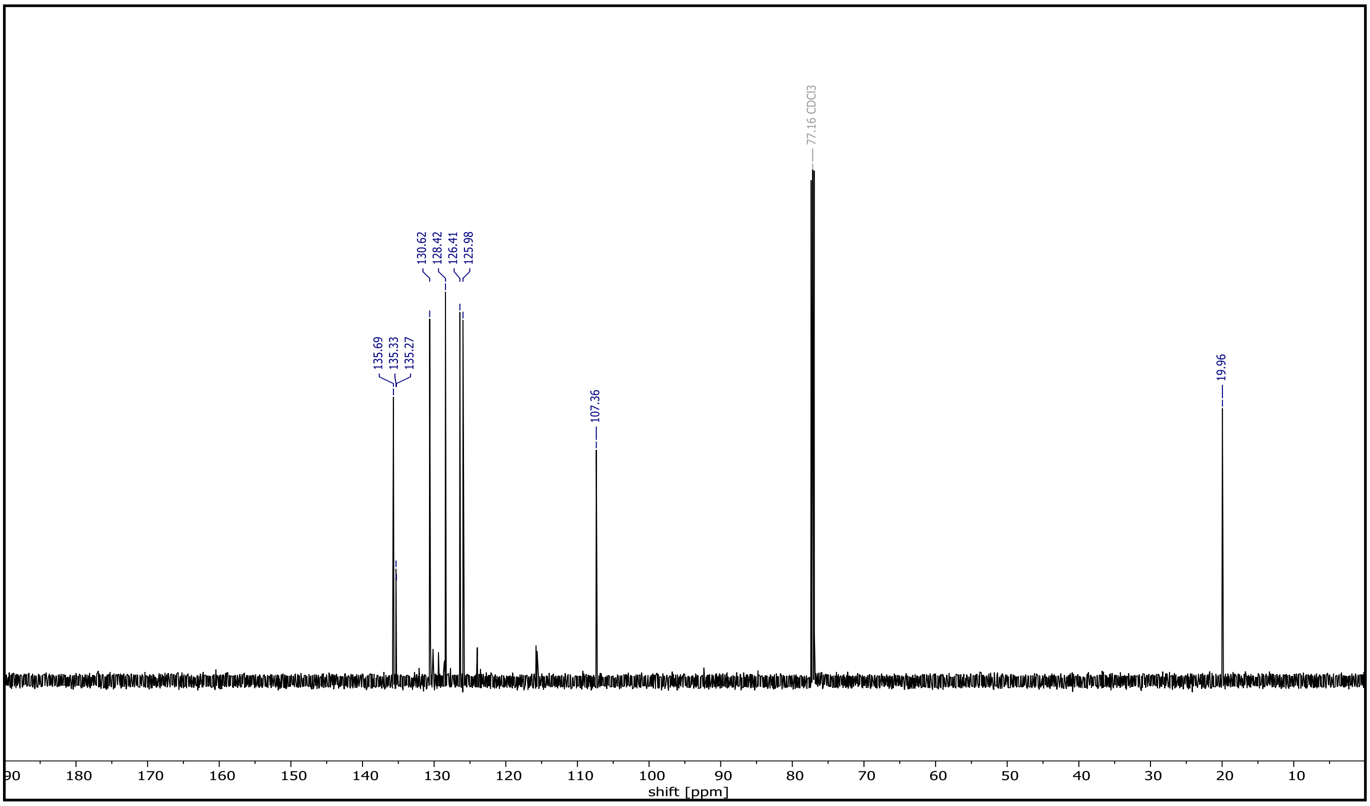

(*Z*)-1-(2-Bromovinyl)-2-methylbenzene (*Z*)-11

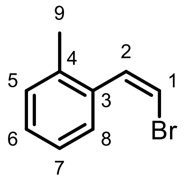

^{1}H NMR (599 MHz, $CDCl_3$)

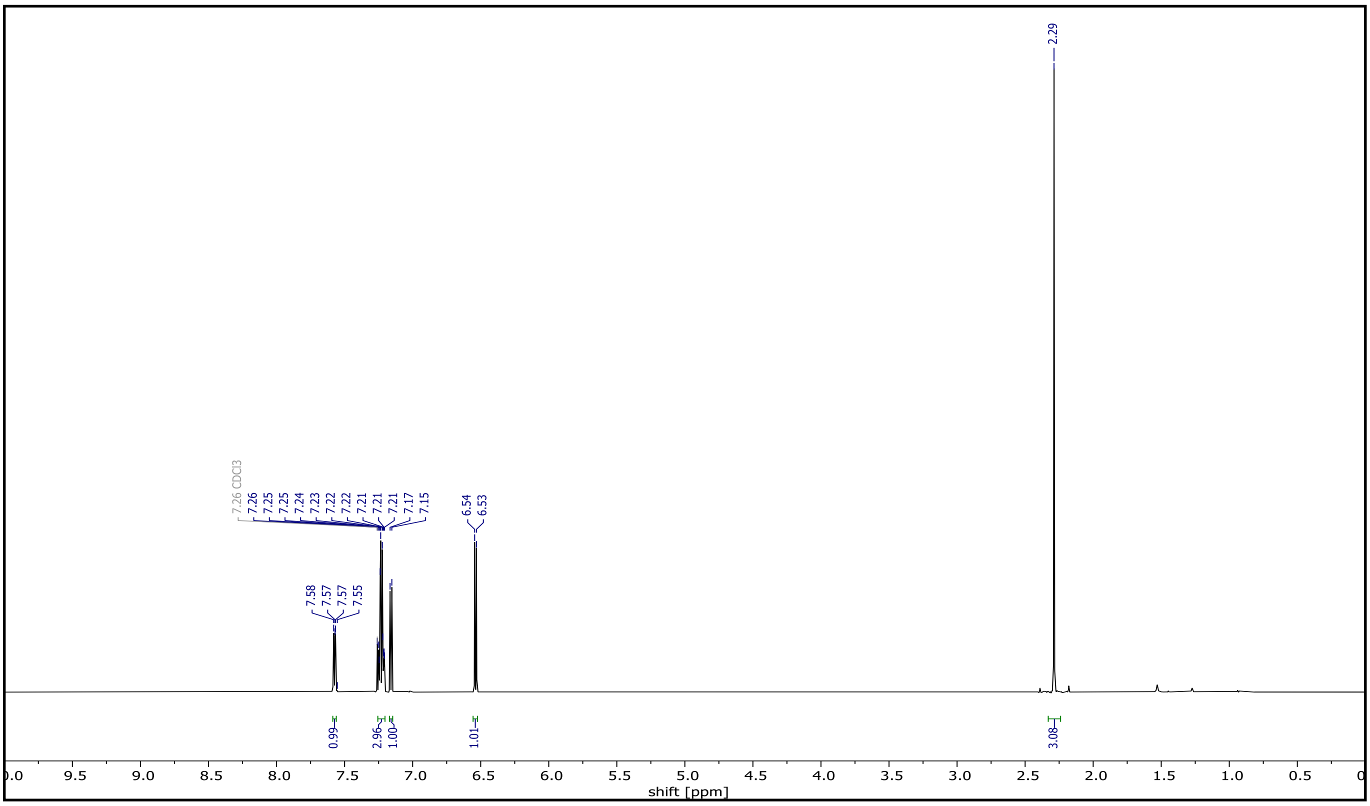

^{13}C{^{1}H} NMR (151 MHz, $CDCl_3$)

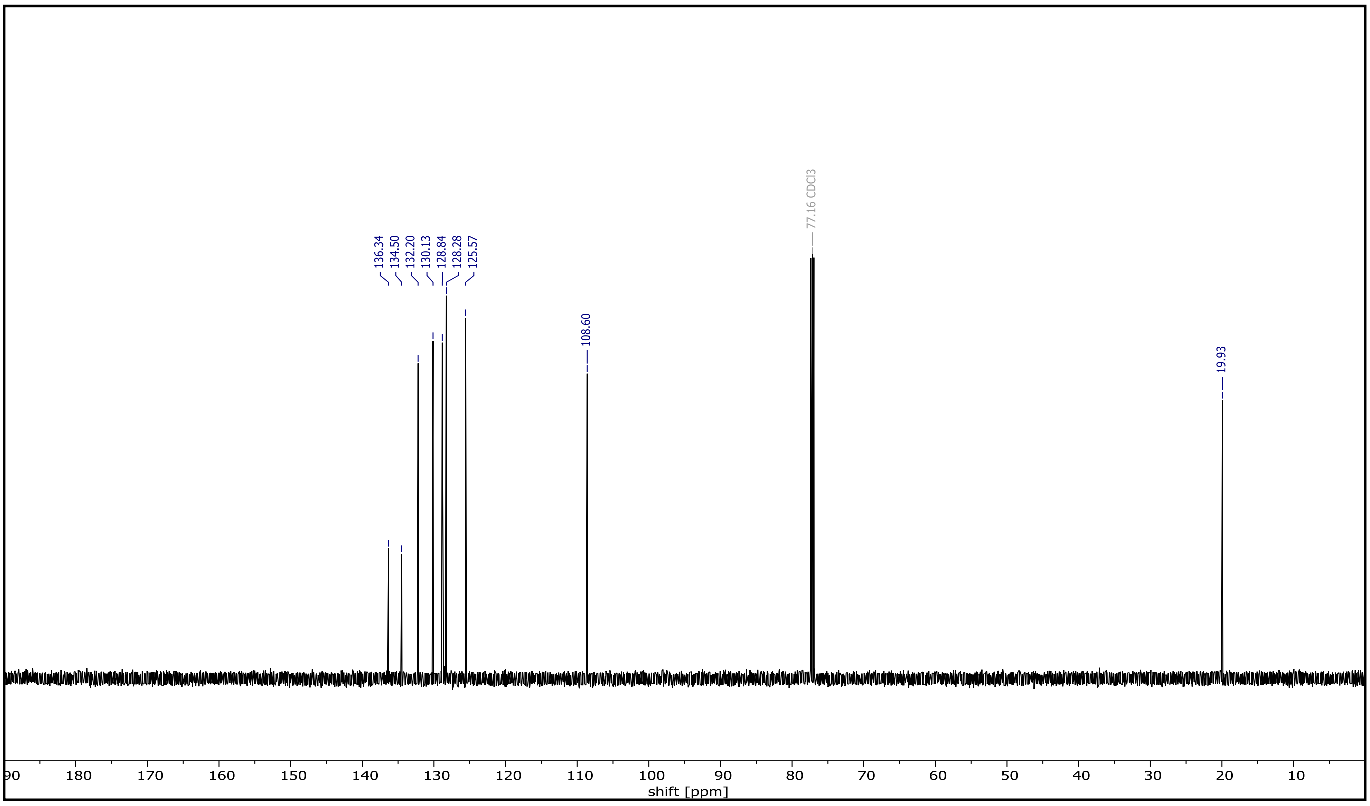

(E,Z)-1-(2-Bromovinyl)-3-methylbenzene (E,Z)-12

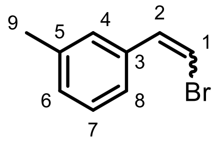

^{1}H NMR (400 MHz, $CDCl_3$), experiment 1

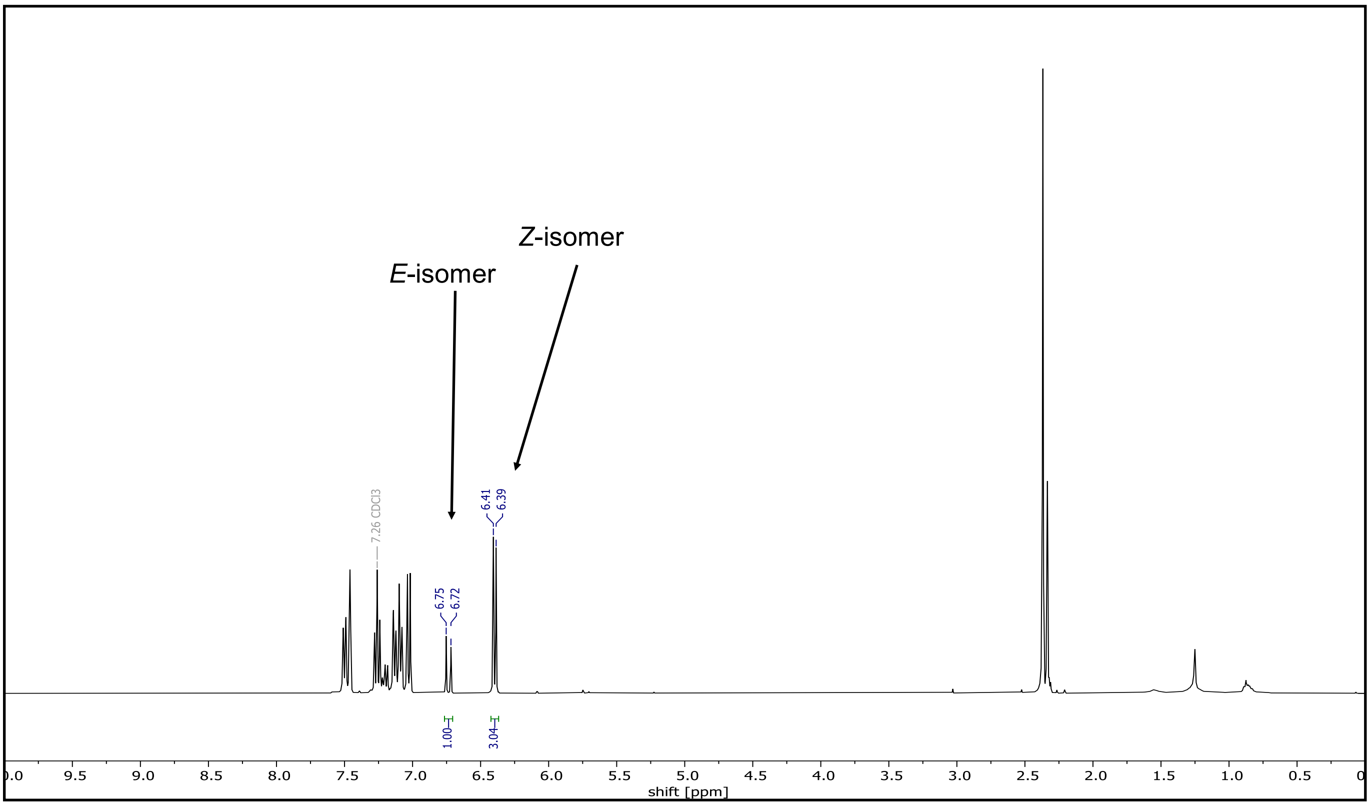

^{1}H NMR (400 MHz, $CDCl_3$), experiment 2

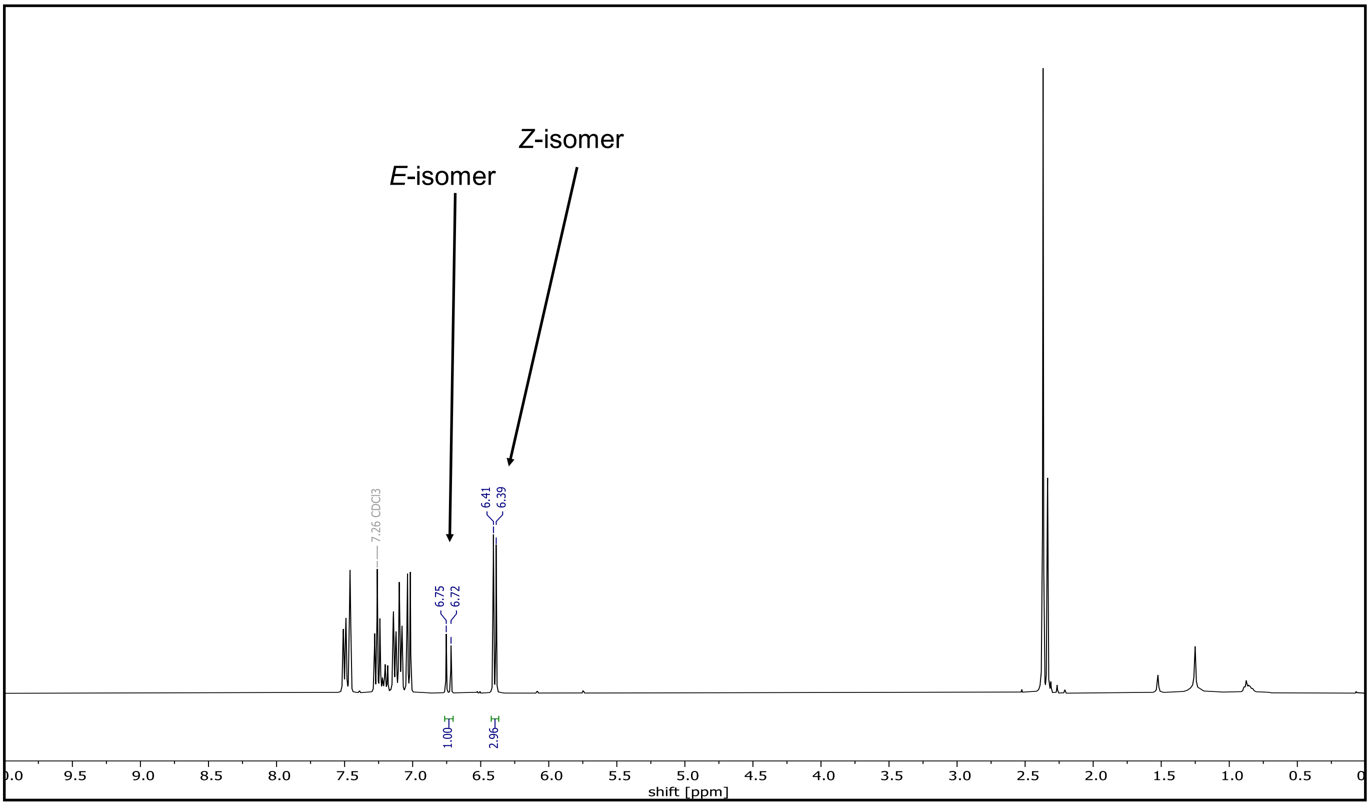

(E,Z)-1-(2-Bromovinyl)-3-methylbenzene (E,Z)-12

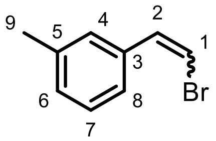

^{1}H NMR ($CDCl_3$), experiment 1

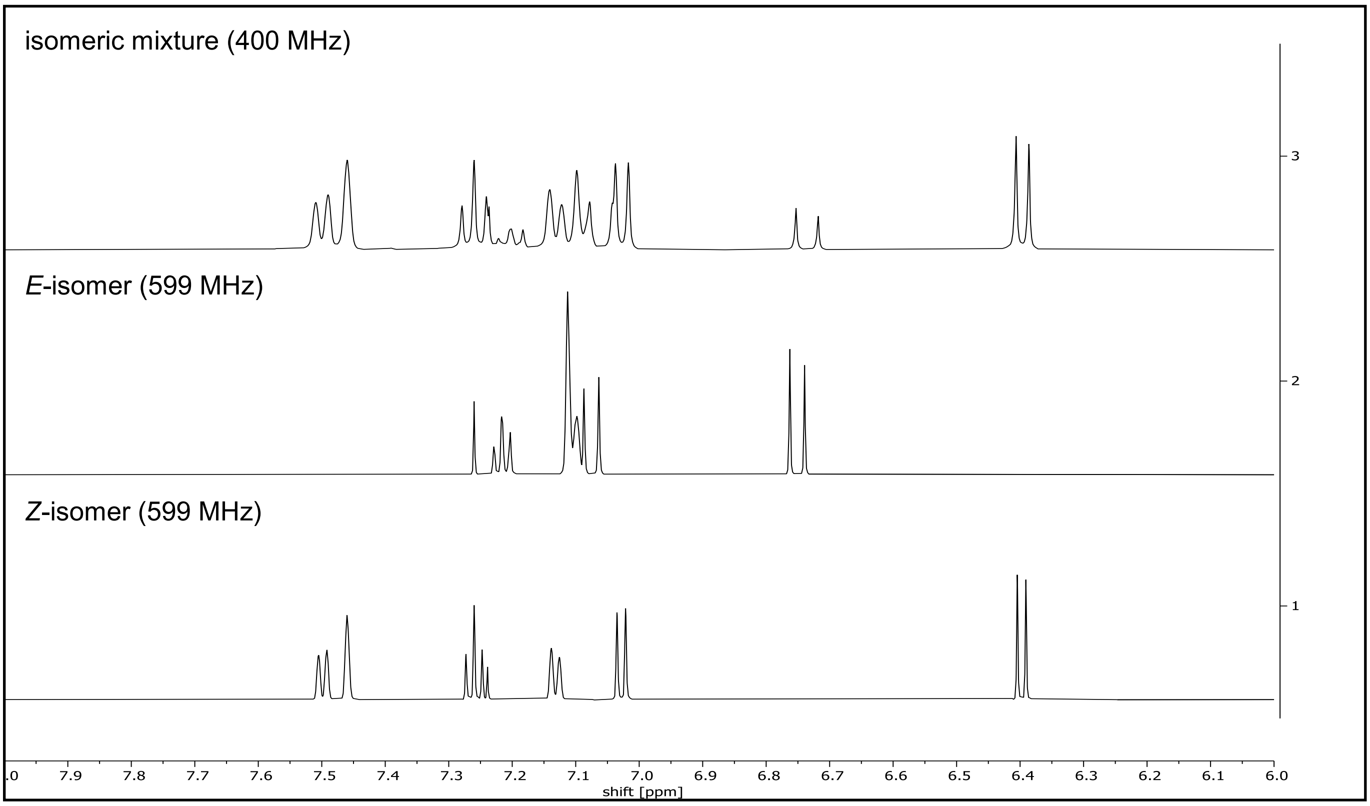

^{1}H NMR ($CDCl_3$), experiment 2

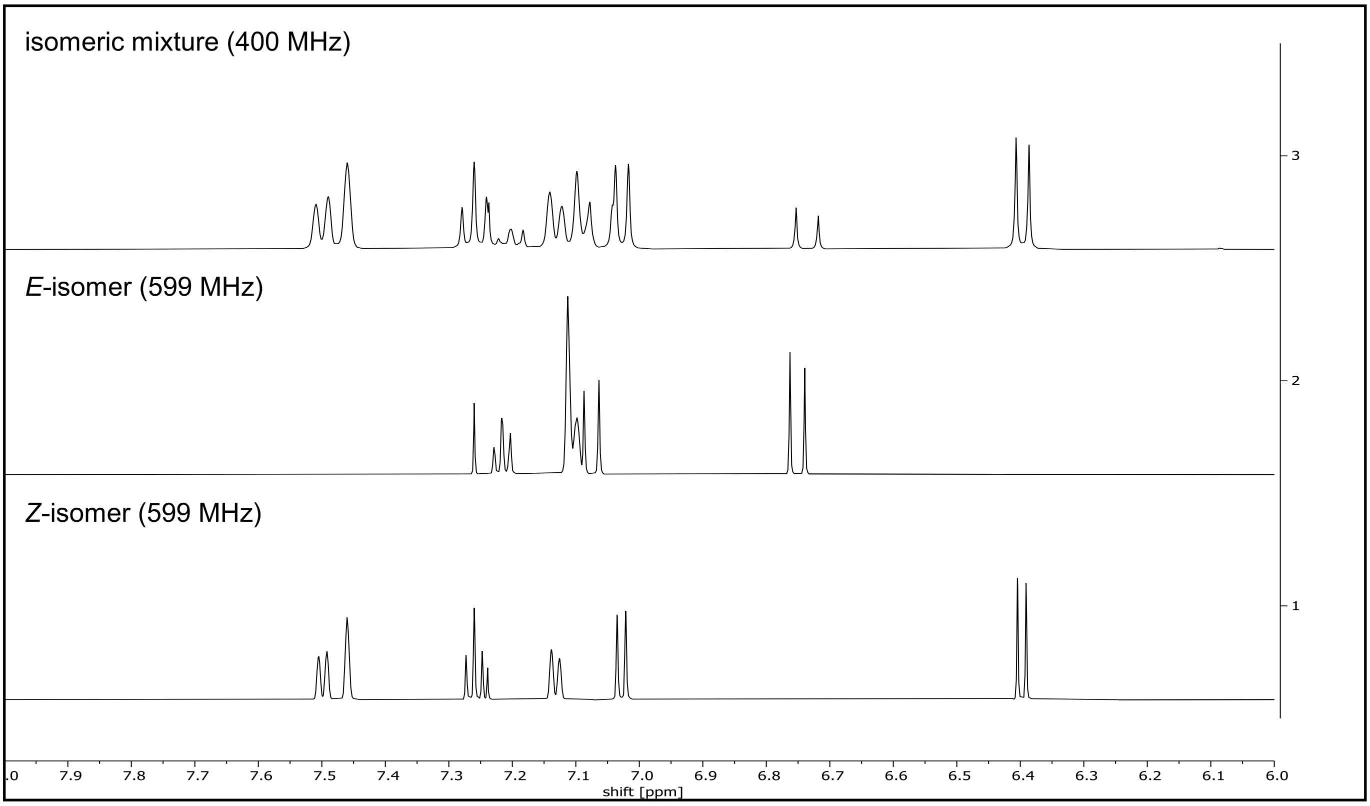

(*E*)-1-(2-Bromovinyl)-3-methylbenzene (*E*)-12

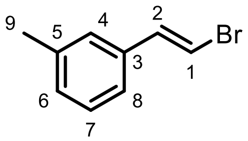

^{1}H NMR (599 MHz, $CDCl_3$)

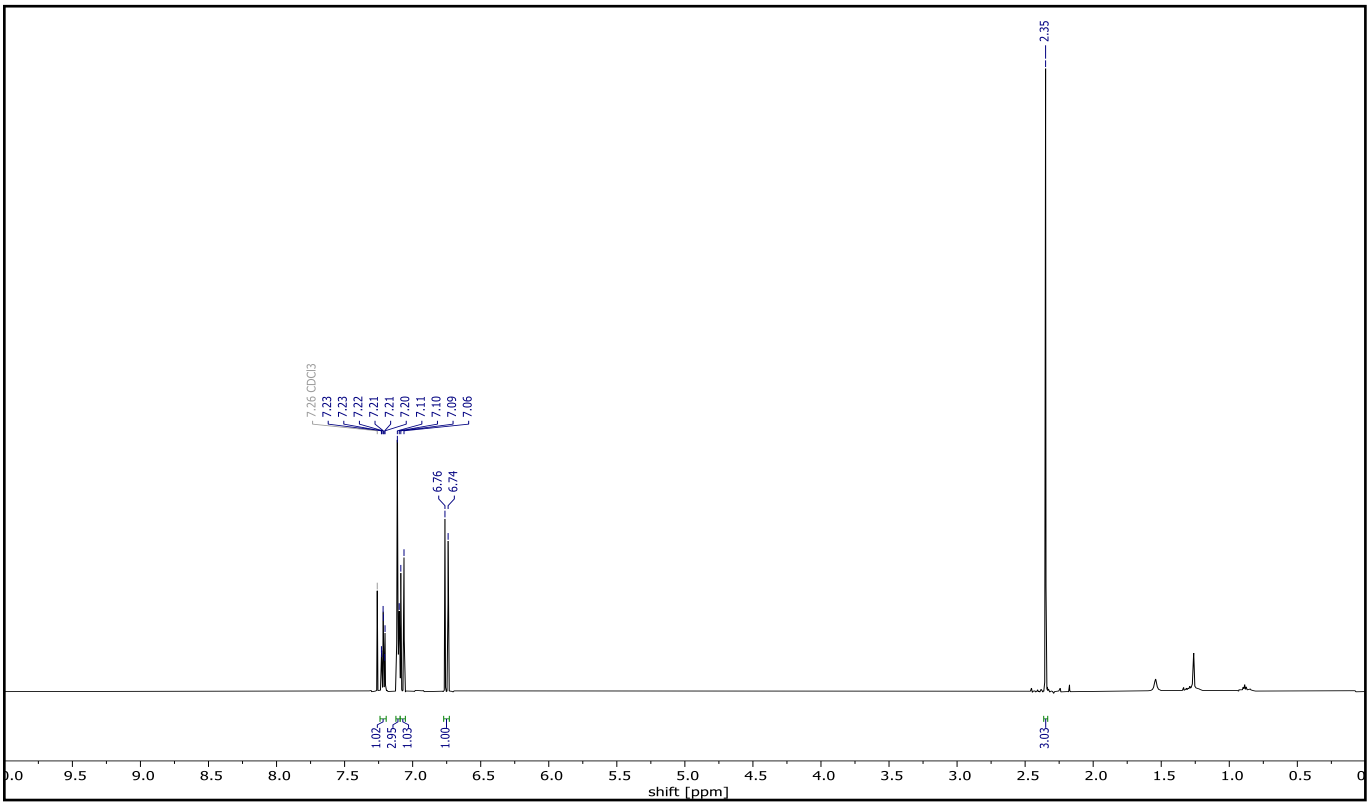

^{13}C{^{1}H} NMR (151 MHz, $CDCl_3$)

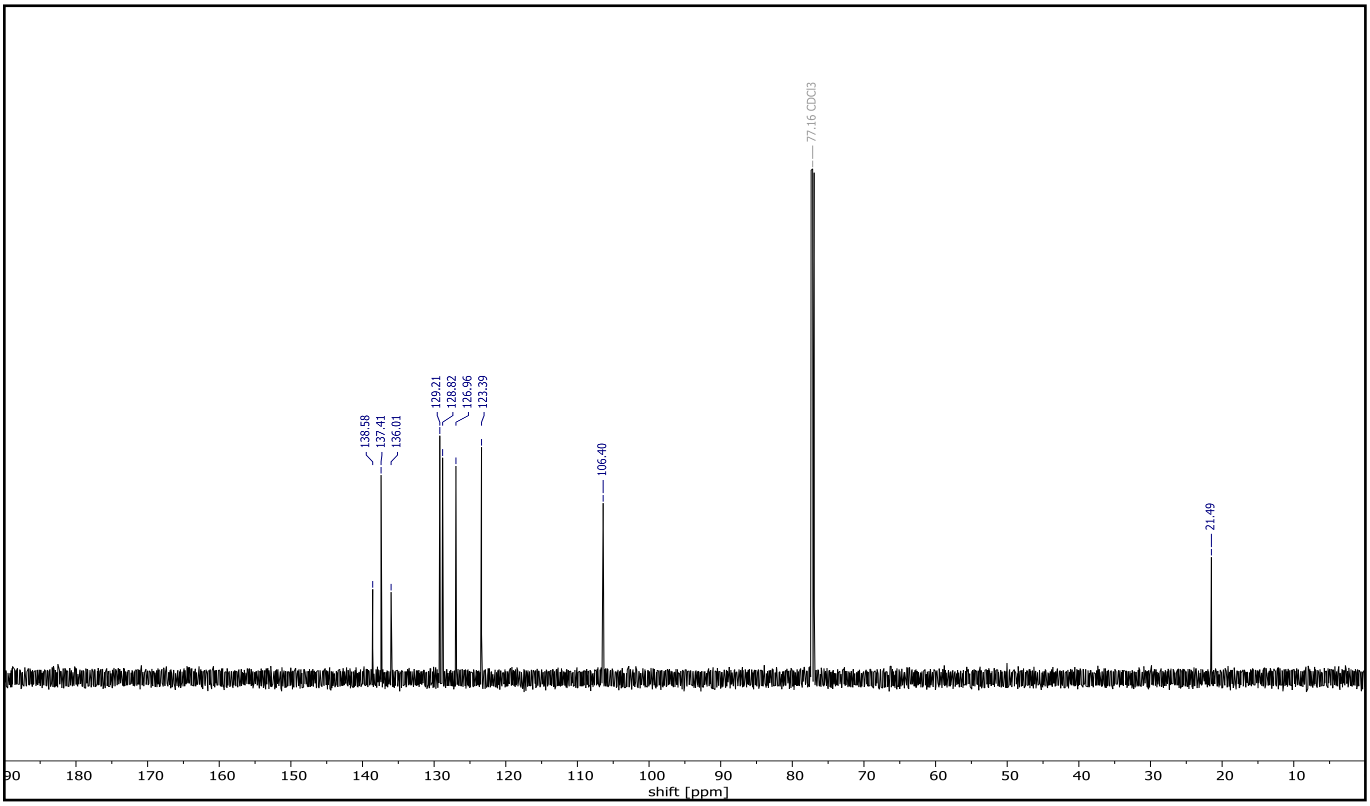

(Z)-1-(2-Bromovinyl)-4-methylbenzene (Z)-12

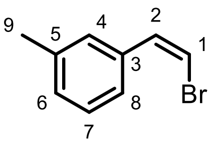

^{1}H NMR (599 MHz, $CDCl_3$)

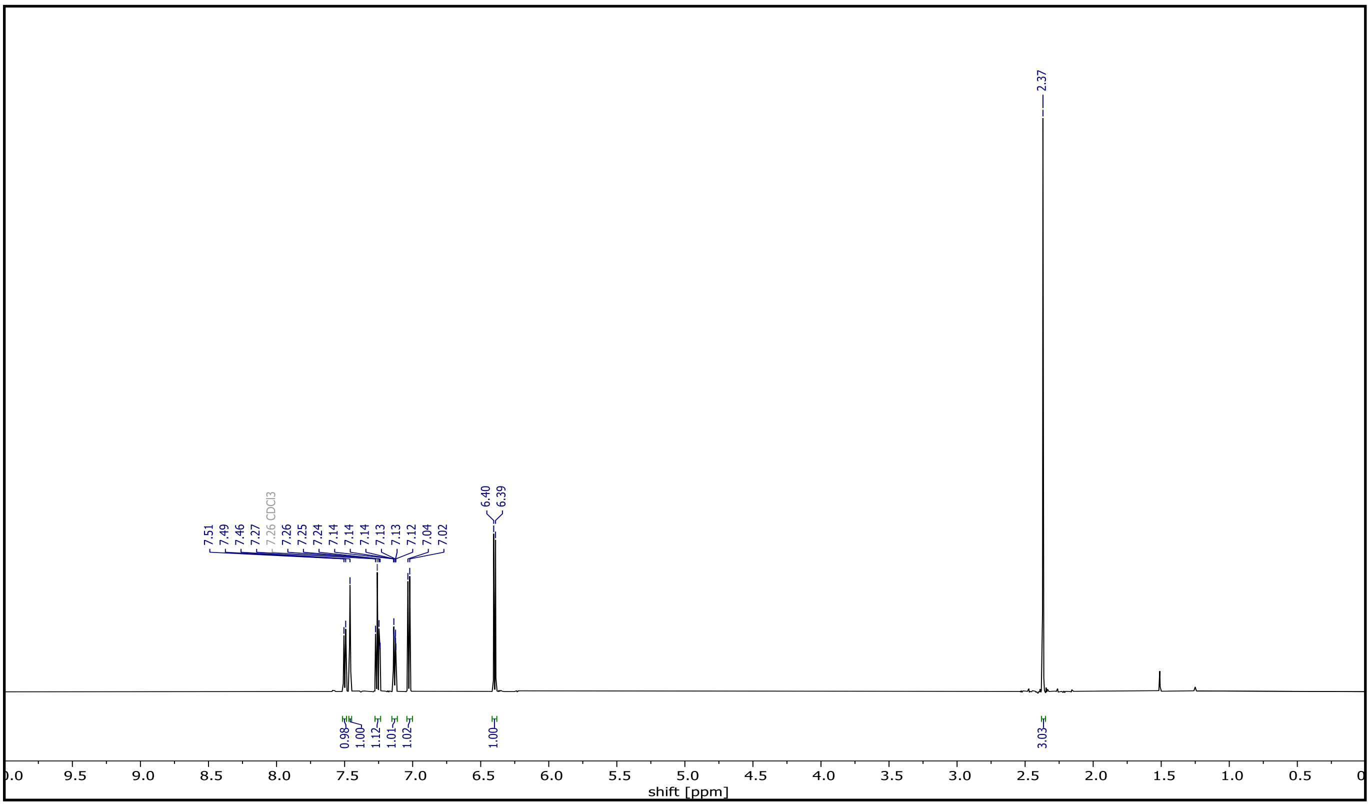

^{13}C{^{1}H} NMR (151 MHz, $CDCl_3$)

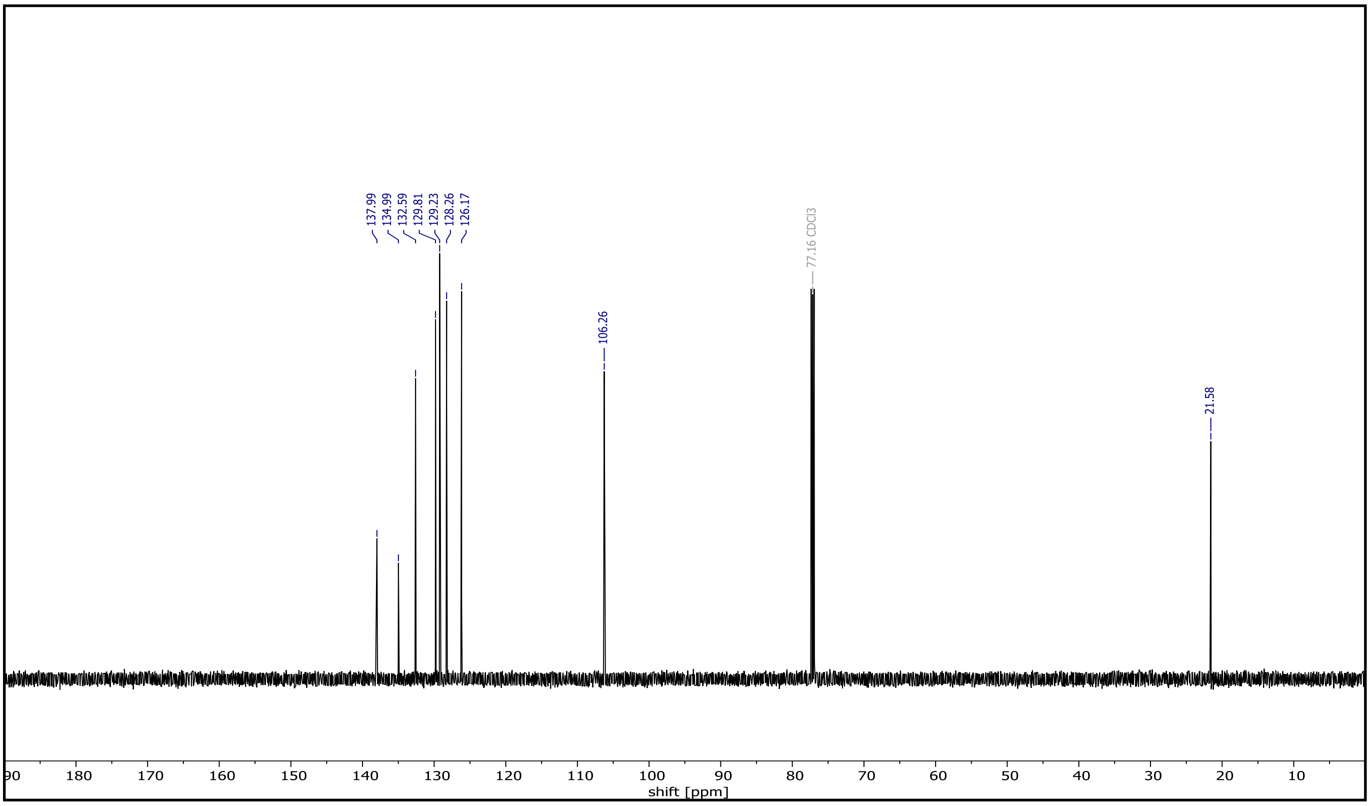

(*E*,*Z*)-2-(2-Bromovinyl)naphthalene (*E*,*Z*)-13

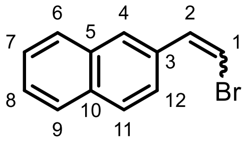

^{1}H NMR (400 MHz, $CDCl_3$), experiment 1

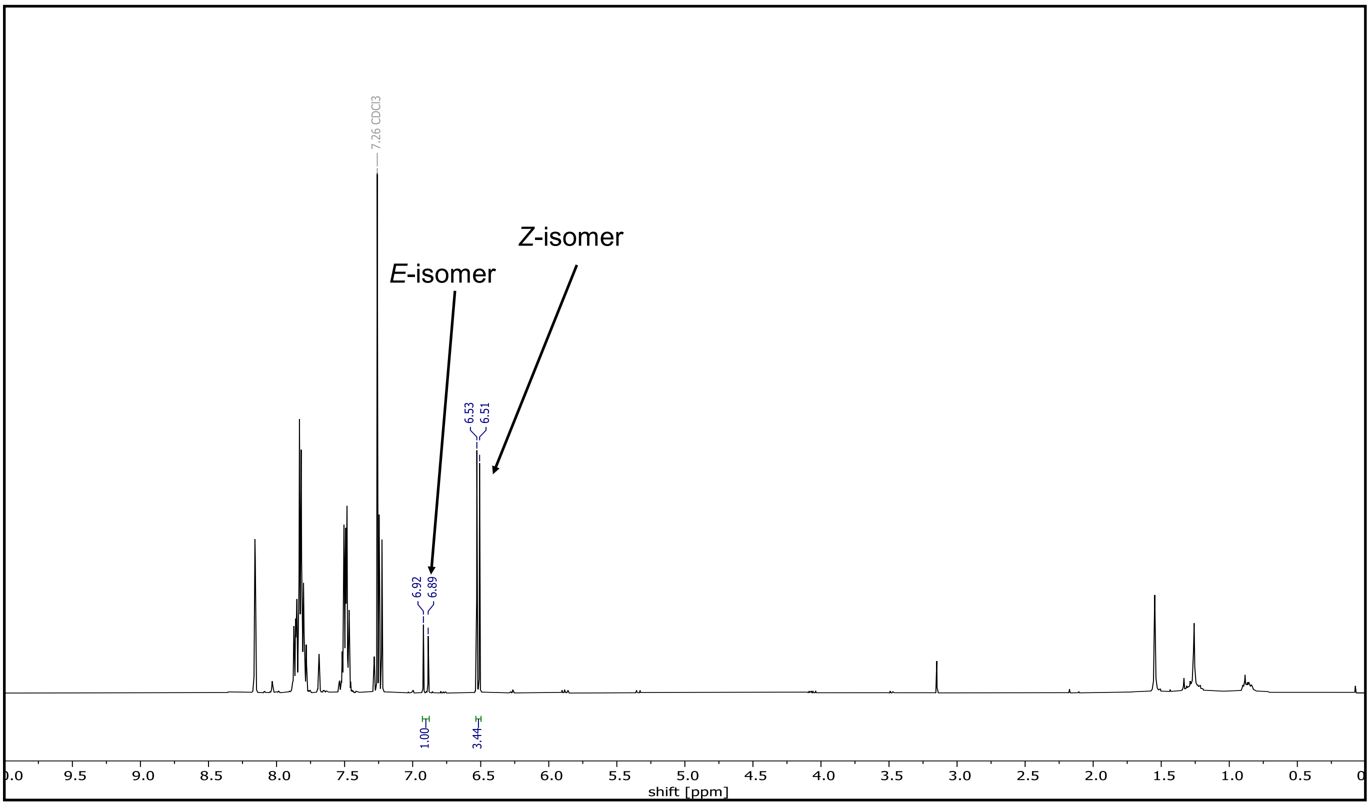

^{1}H NMR (400 MHz, $CDCl_3$), experiment 2

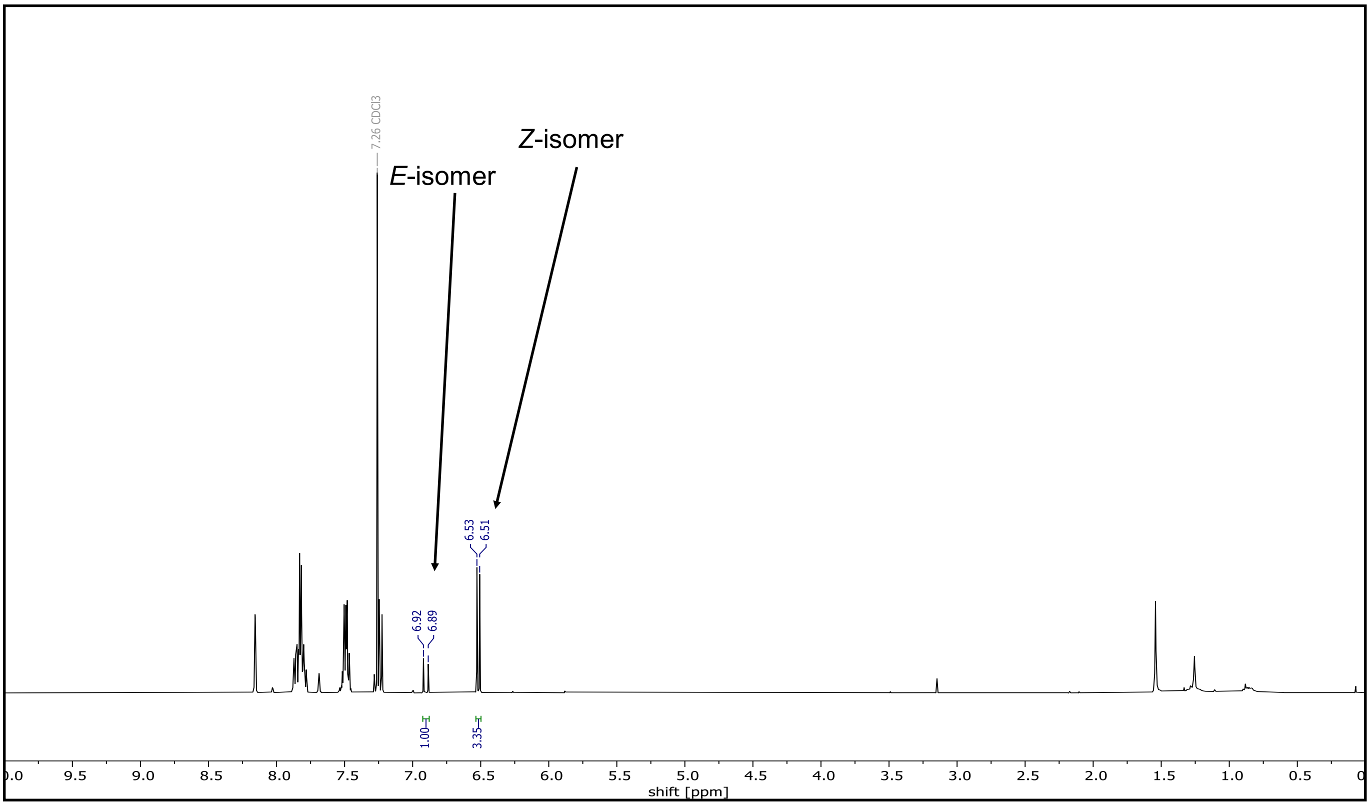

(*E*,*Z*)-2-(2-Bromovinyl)naphthalene (*E*,*Z*)-13

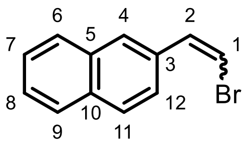

^{1}H NMR ($CDCl_3$), experiment 1

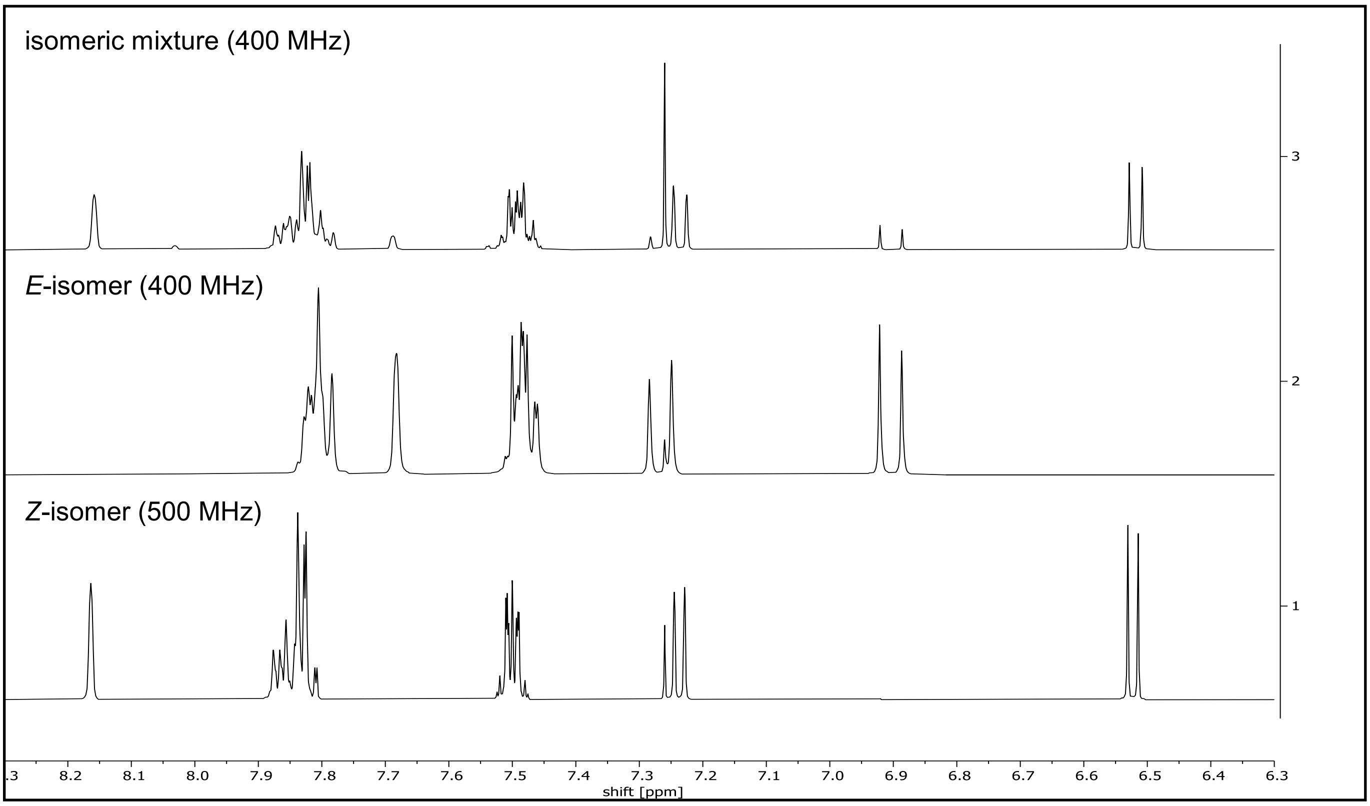

^{1}H NMR ($CDCl_3$), experiment 2

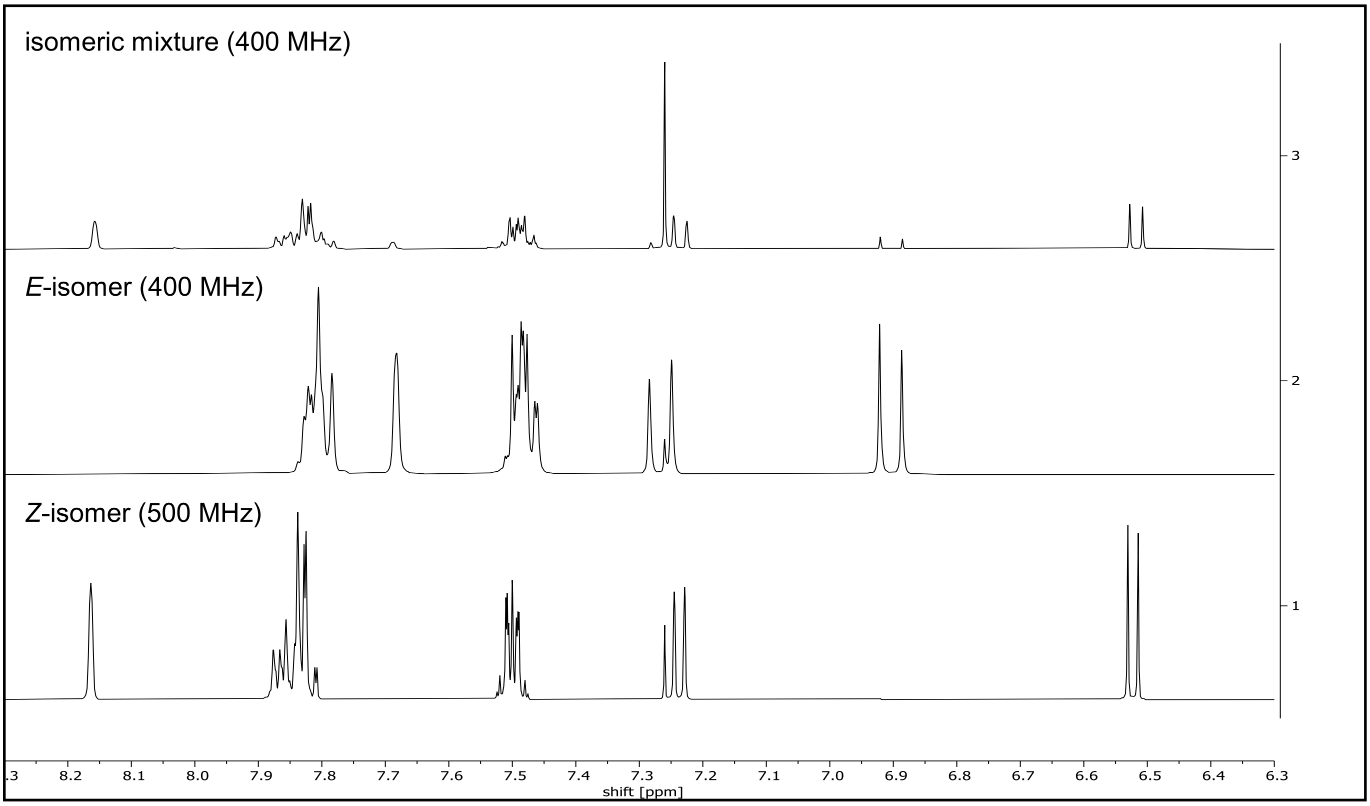

(*E*)-2-(2-Bromovinyl)naphthalene (*E*)-13

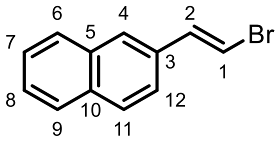

^{1}H NMR (400 MHz, $CDCl_3$)

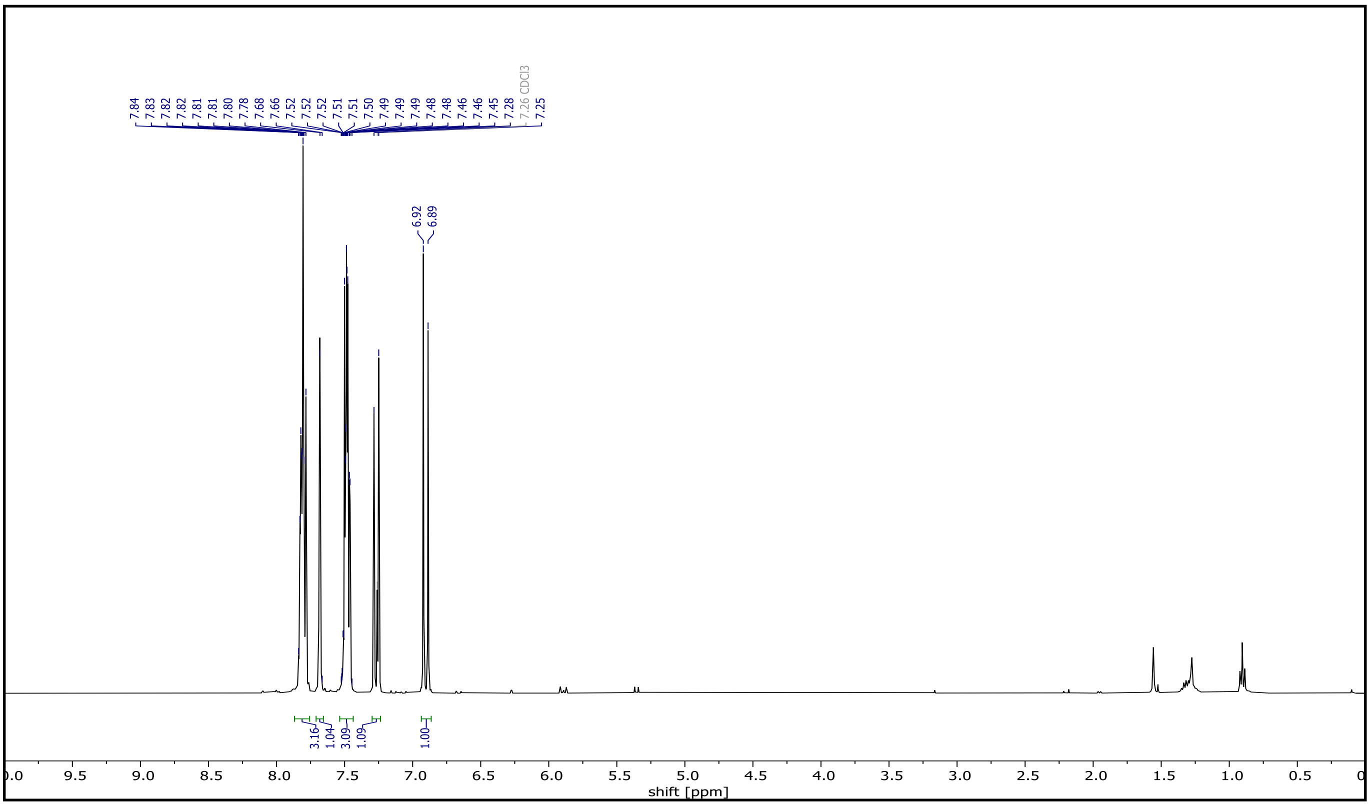

^{13}C{^{1}H} NMR (151 MHz, $CDCl_3$)

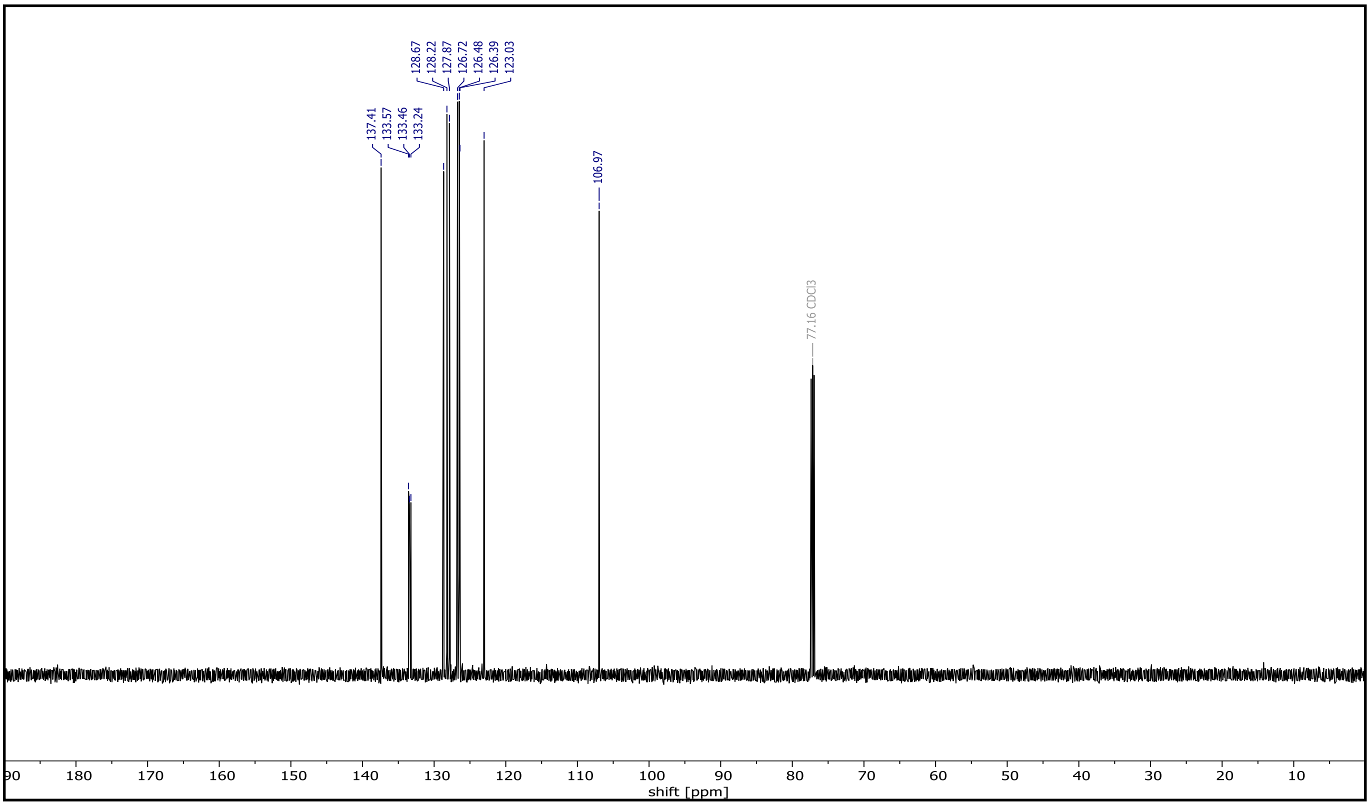

(*Z*)-2-(2-Bromovinyl)naphthalene (*Z*)-13

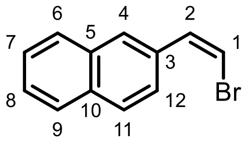

^{1}H NMR (500 MHz, $CDCl_3$)

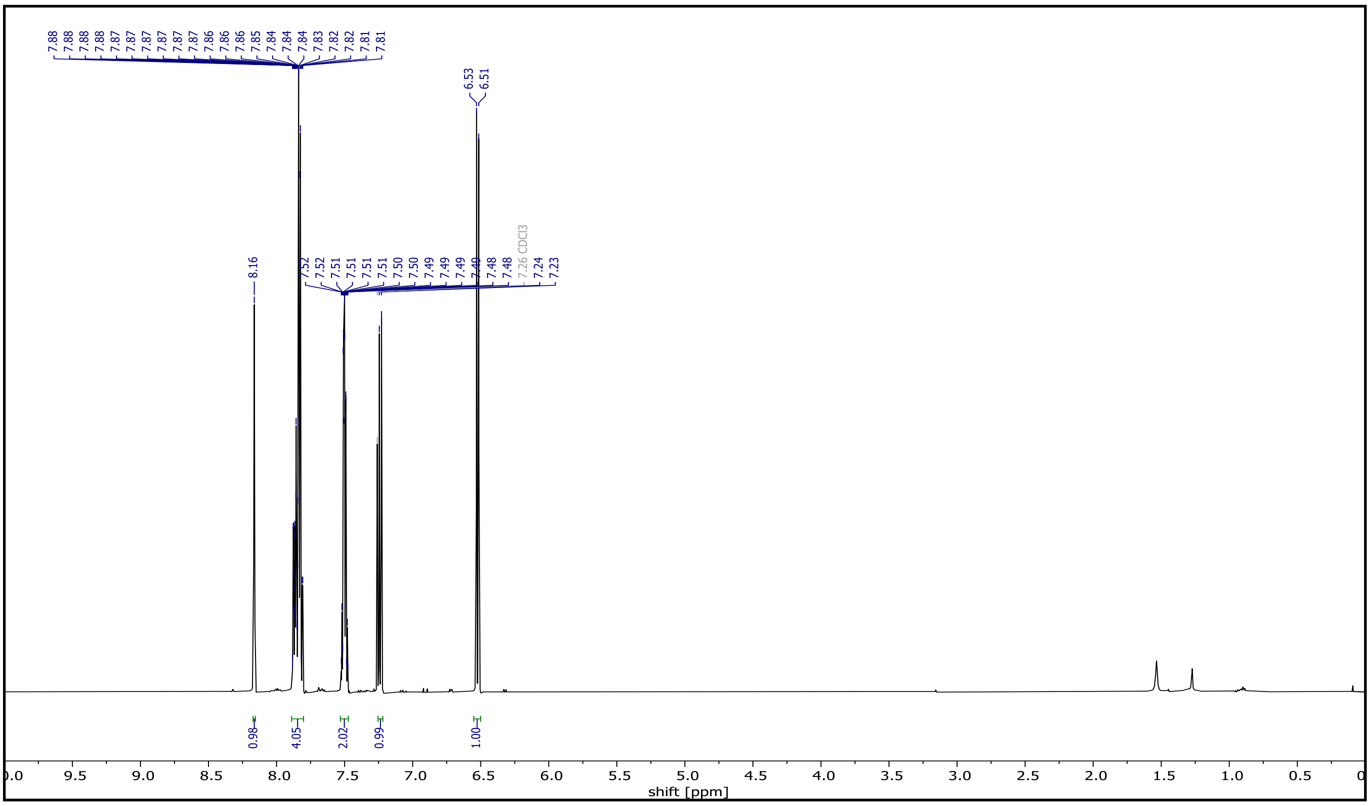

$^{13}C\{^{1}H\}$ NMR (126 MHz, $CDCl_3$)

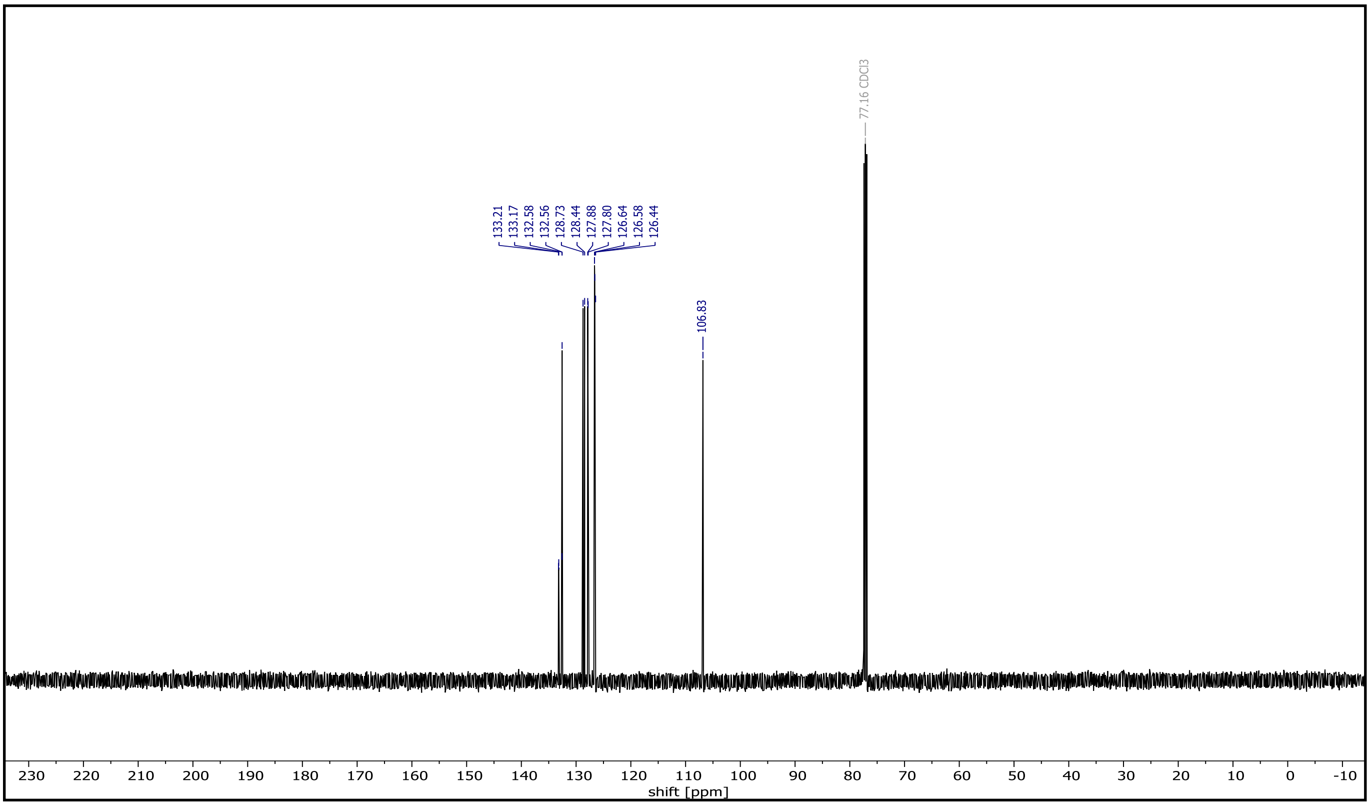

(*E*,*Z*)-1-(2-Bromovinyl)-4-(trifluoromethyl)benzene (*E*,*Z*)-14

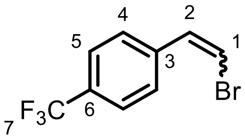

^1H NMR (400 MHz, $CDCl_3$), experiment 1

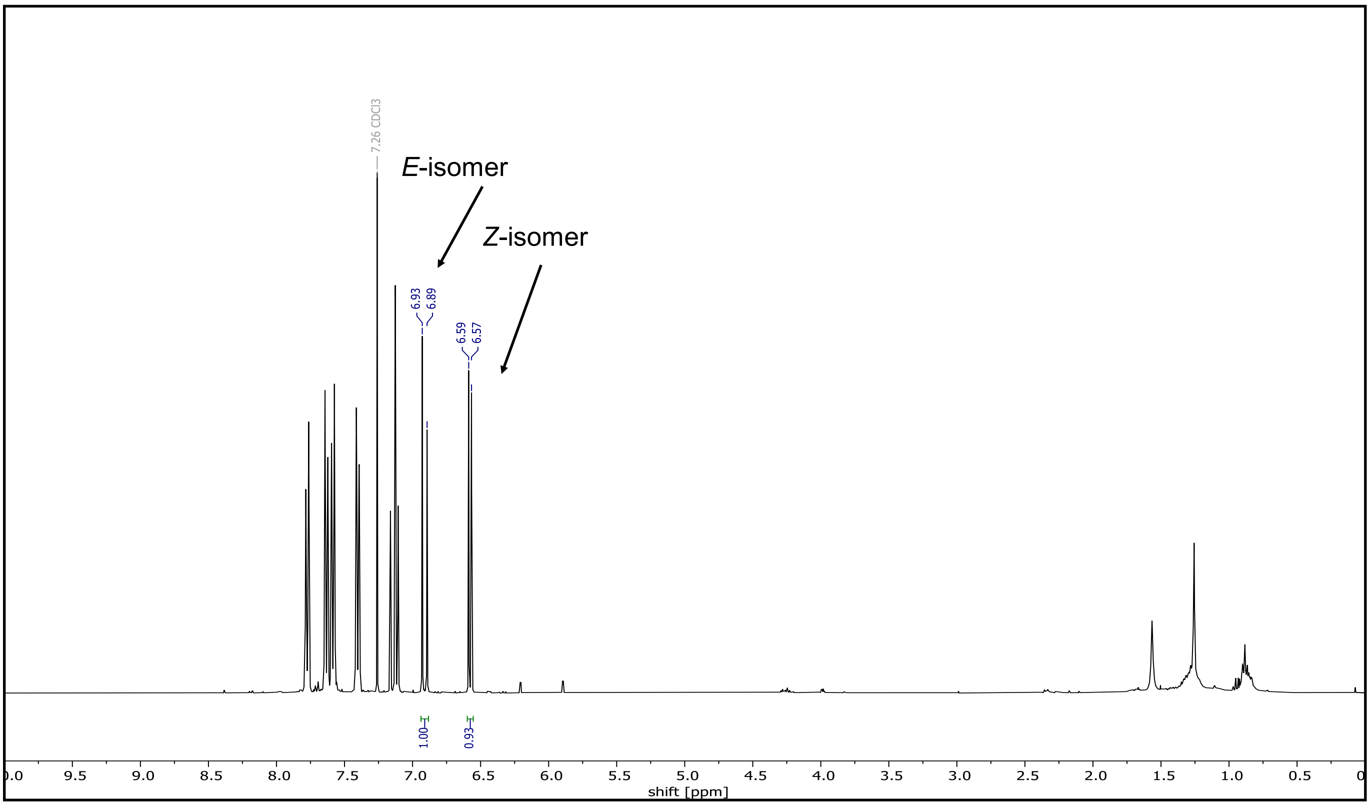

^1H NMR (400 MHz, $CDCl_3$), experiment 2

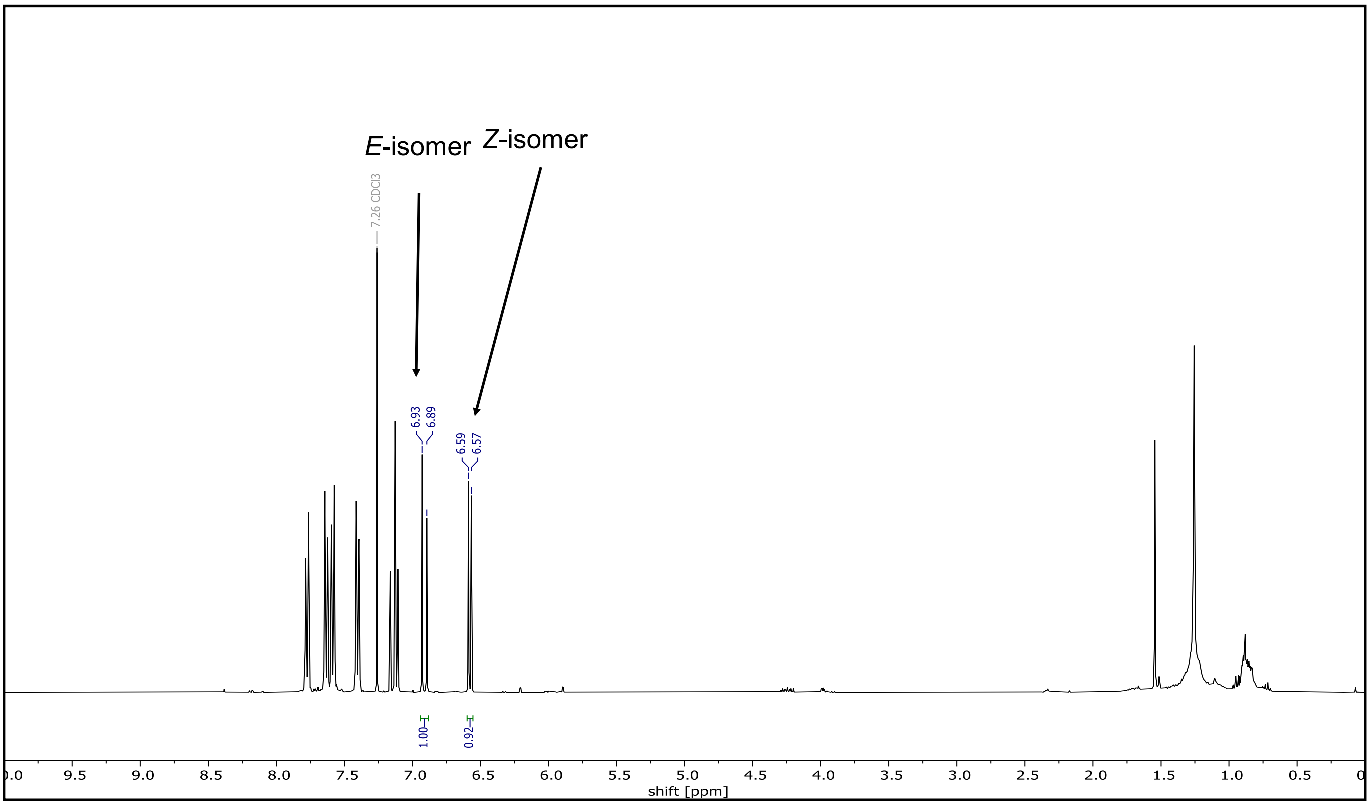

(*E*,*Z*)-1-(2-Bromovinyl)-4-(trifluoromethyl)benzene (*E*,*Z*)-14

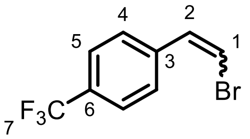

^1H NMR ($CDCl_3$), experiment 1

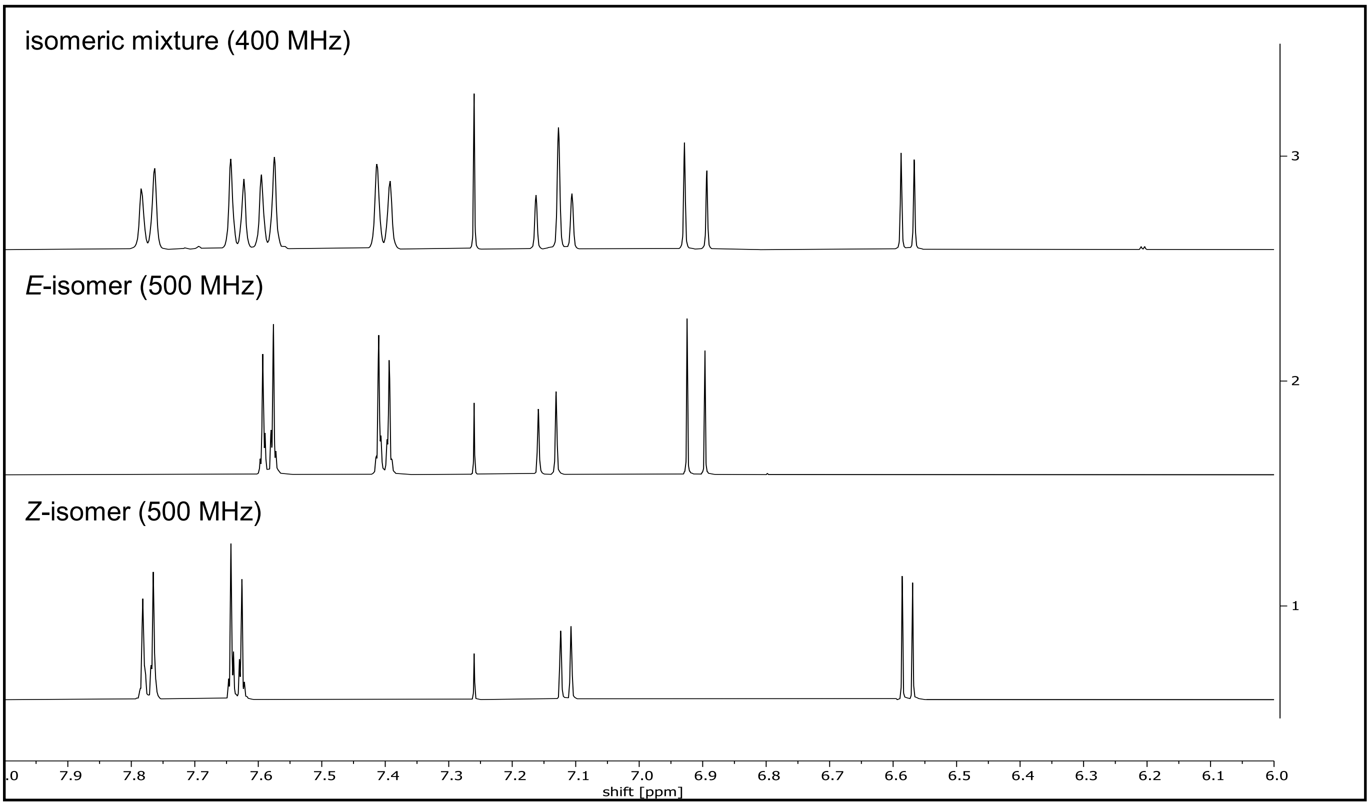

^1H NMR ($CDCl_3$), experiment 2

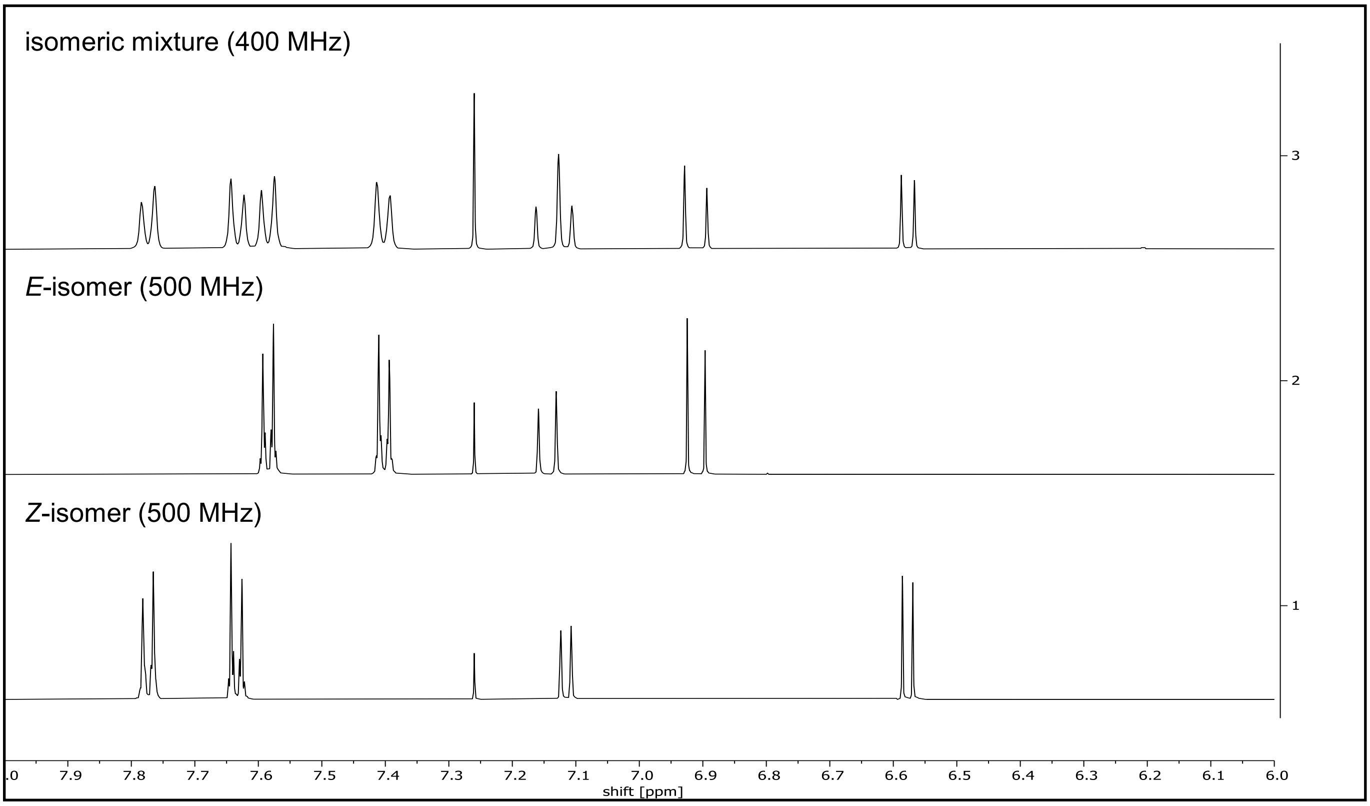

(*E*)-1-(2-Bromovinyl)-4-(trifluoromethyl)benzene (*E*)-14

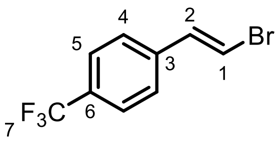

^{1}H NMR (500 MHz, $CDCl_3$)

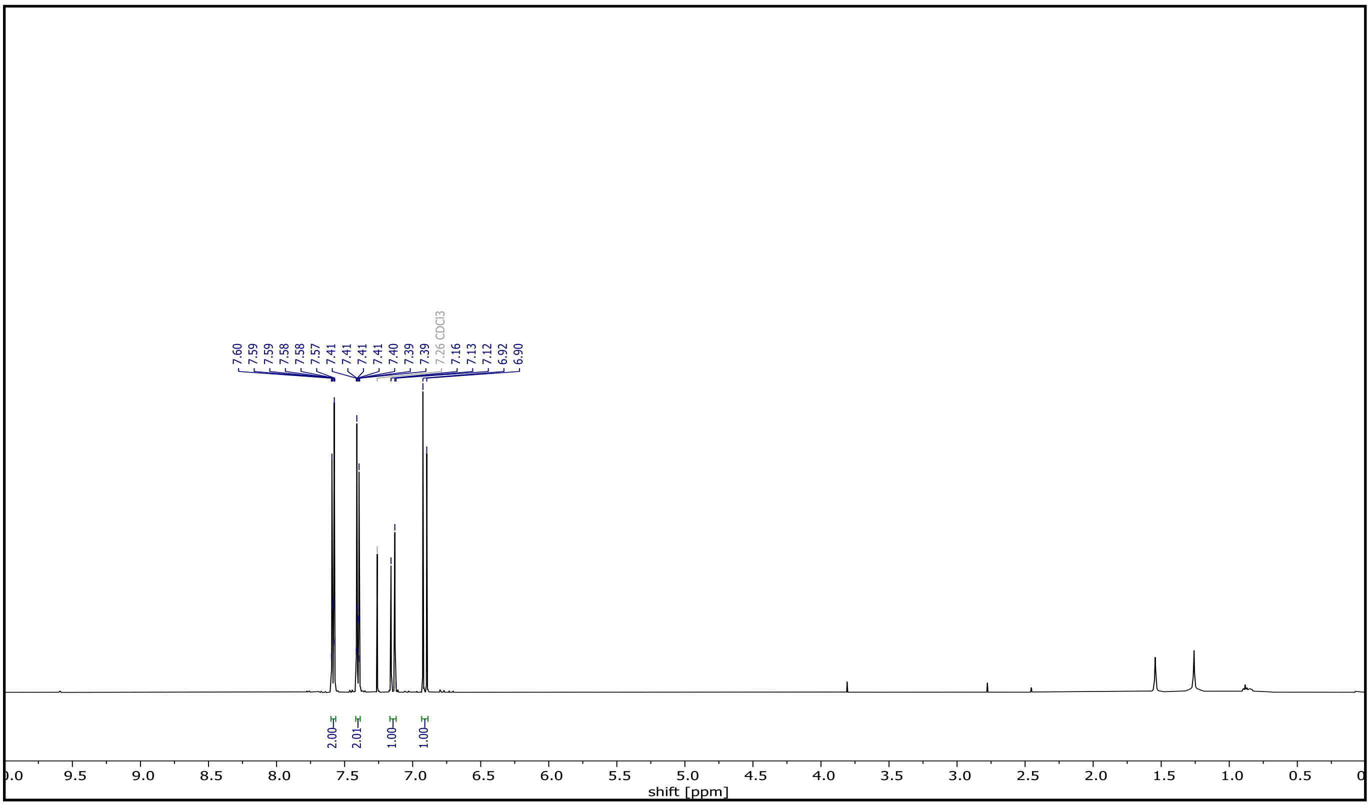

^{13}C{^{1}H} NMR (126 MHz, $CDCl_3$)

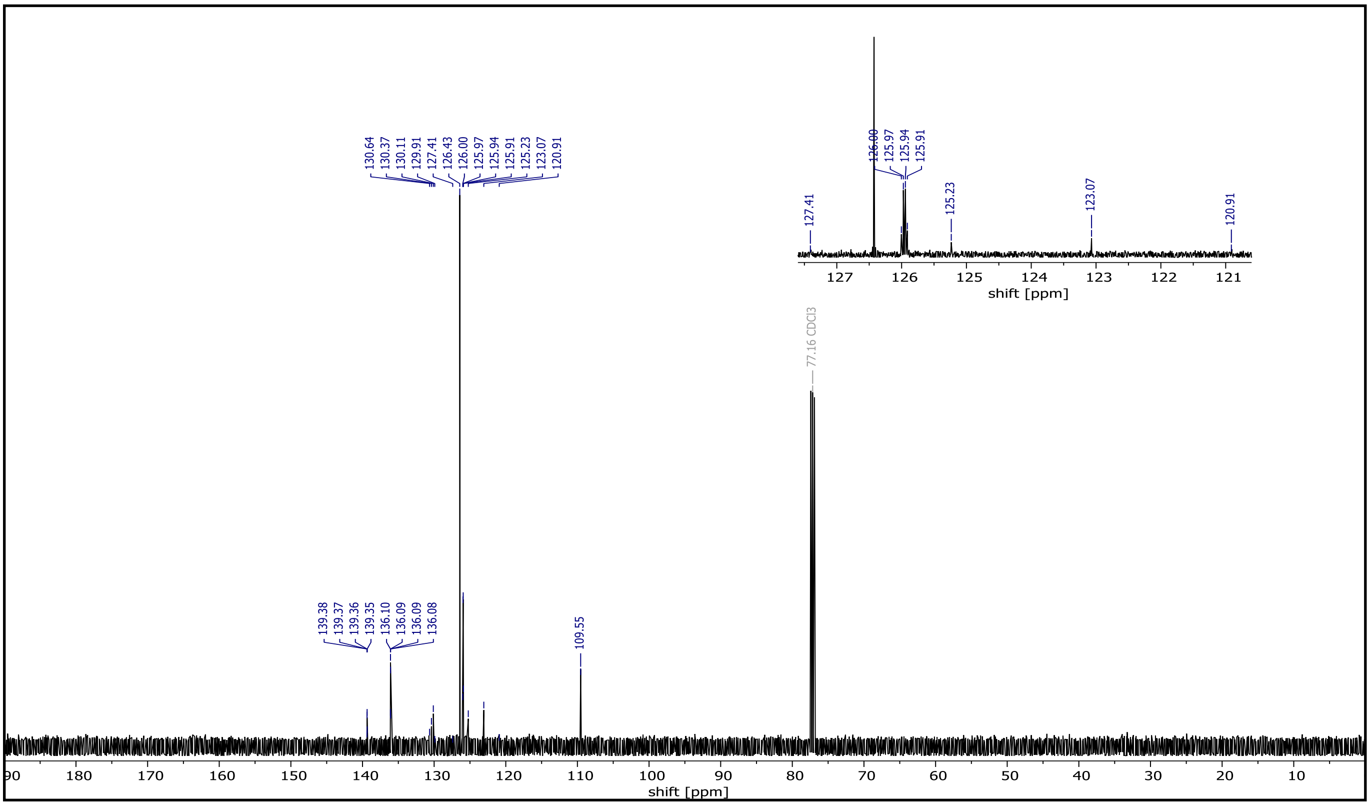

(*E*)-1-(2-Bromovinyl)-4-(trifluoromethyl)benzene (*E*)-14

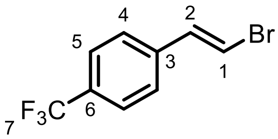

^{19}F NMR (470 MHz, $CDCl_3$)

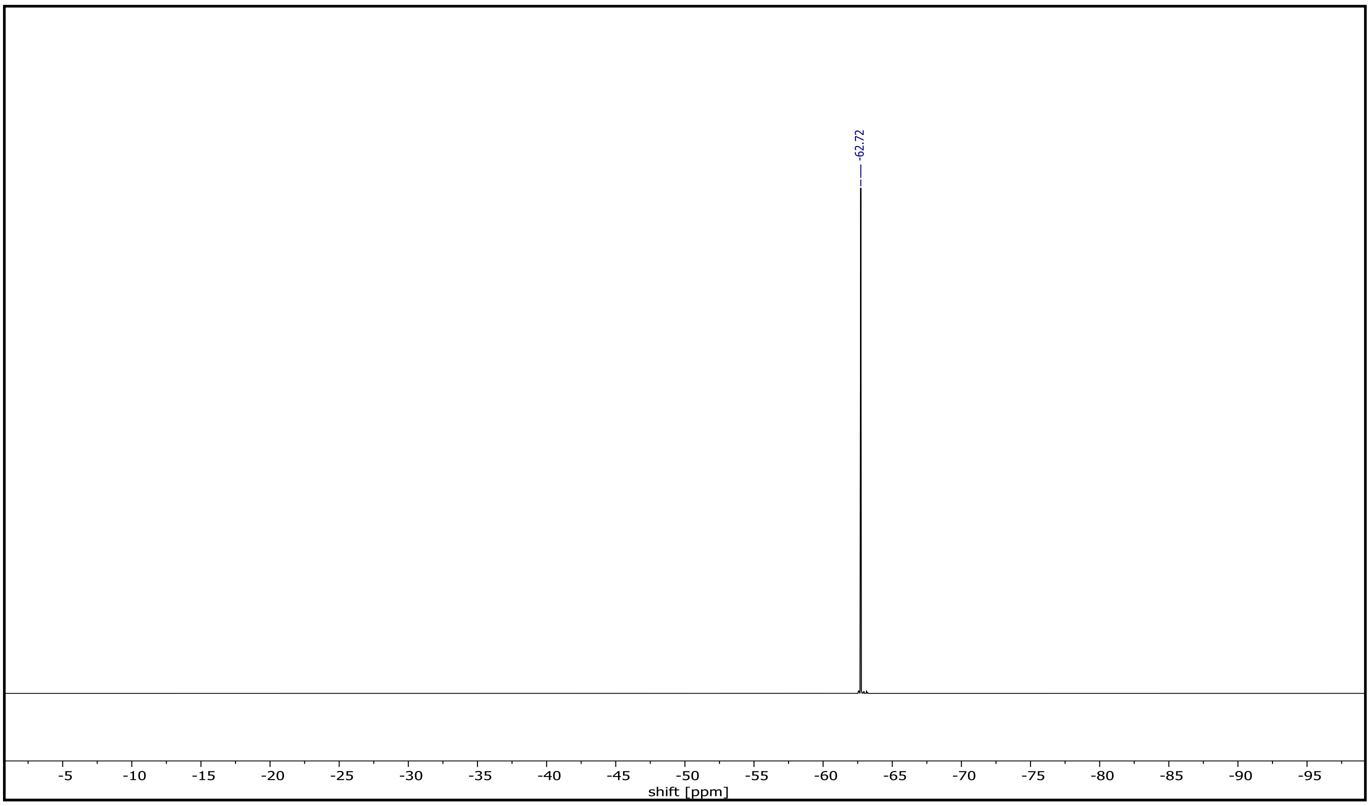

(*Z*)-1-(2-Bromovinyl)-4-(trifluoromethyl)benzene (*Z*)-14

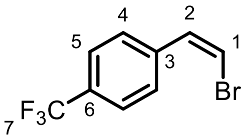

^{1}H NMR (500 MHz, $CDCl_3$)

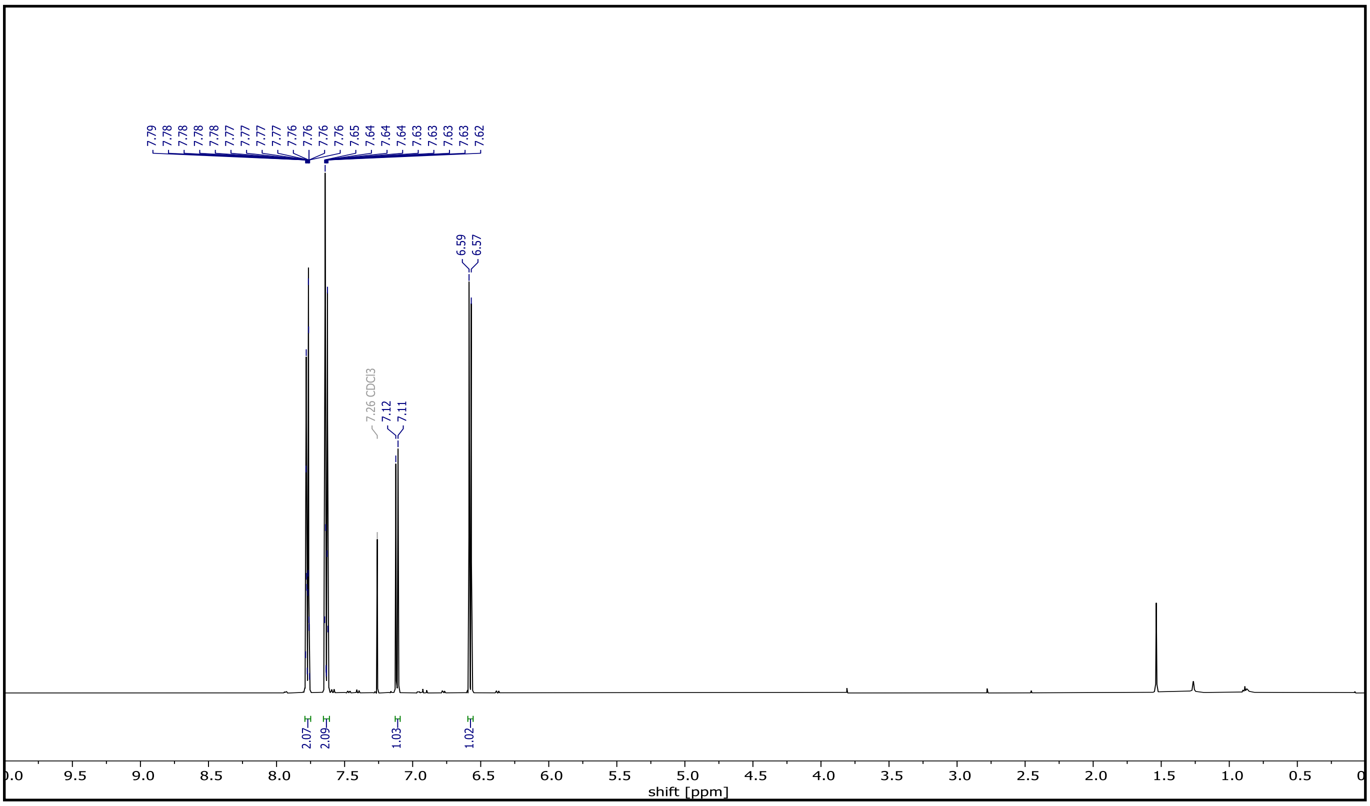

$^{13}C\{^{1}H\}$ NMR (126 MHz, $CDCl_3$)

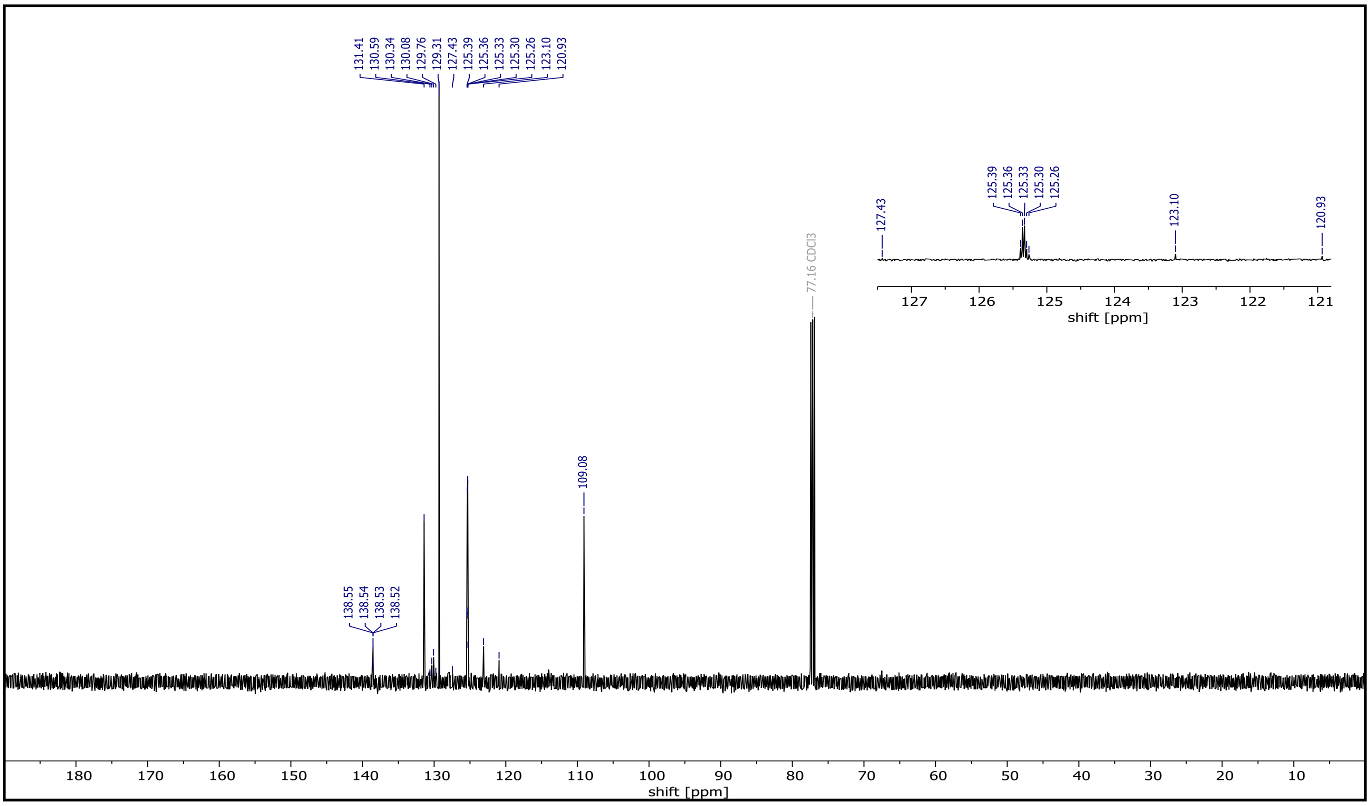

(*Z*)-1-(2-Bromovinyl)-4-(trifluoromethyl)benzene (*Z*)-14

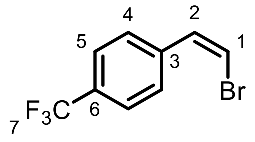

^{19}F NMR (470 MHz, $CDCl_3$)

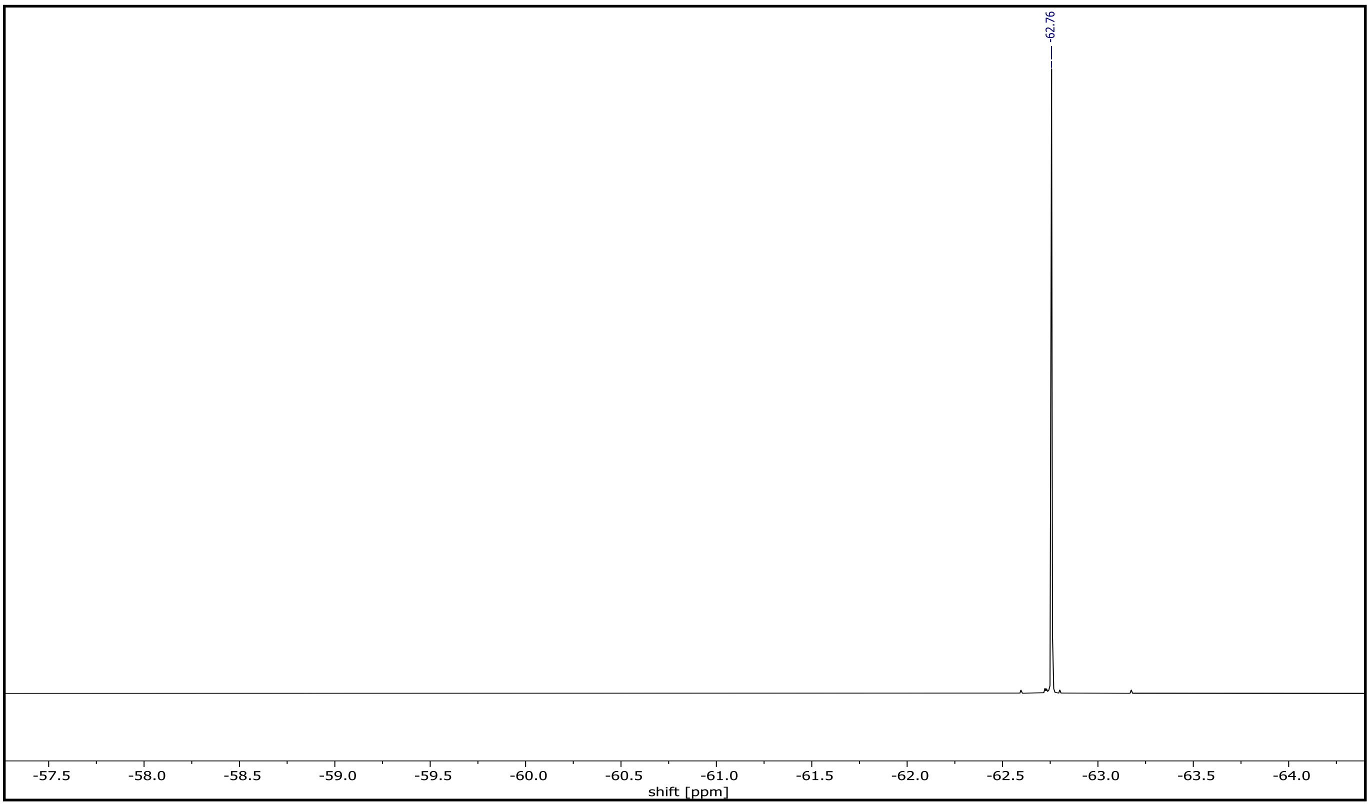

(*E*,*Z*)-1-(2-Bromovinyl)-4-methoxybenzene (*E*,*Z*)-15

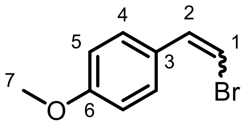

^{1}H NMR (400 MHz, $CDCl_3$), experiment 1

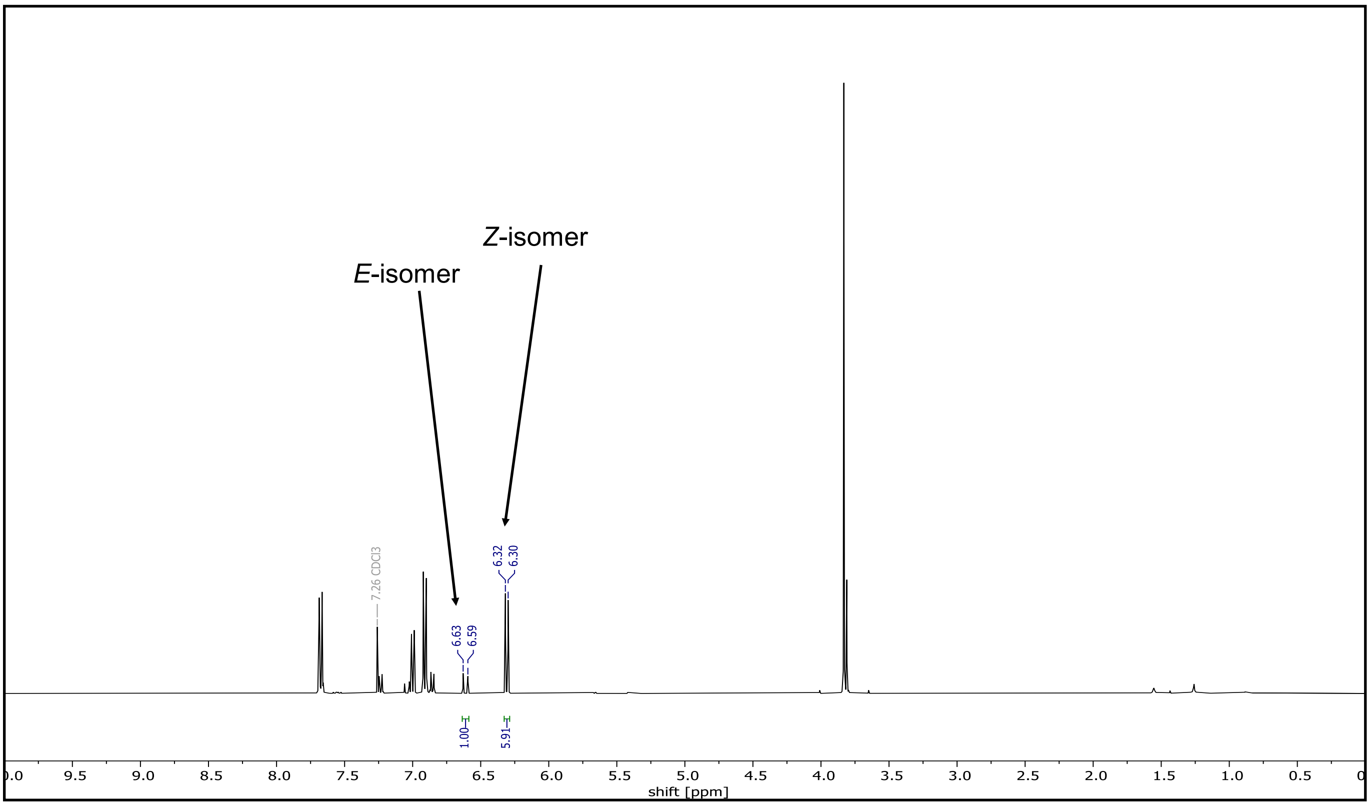

^{1}H NMR (400 MHz, $CDCl_3$), experiment 2

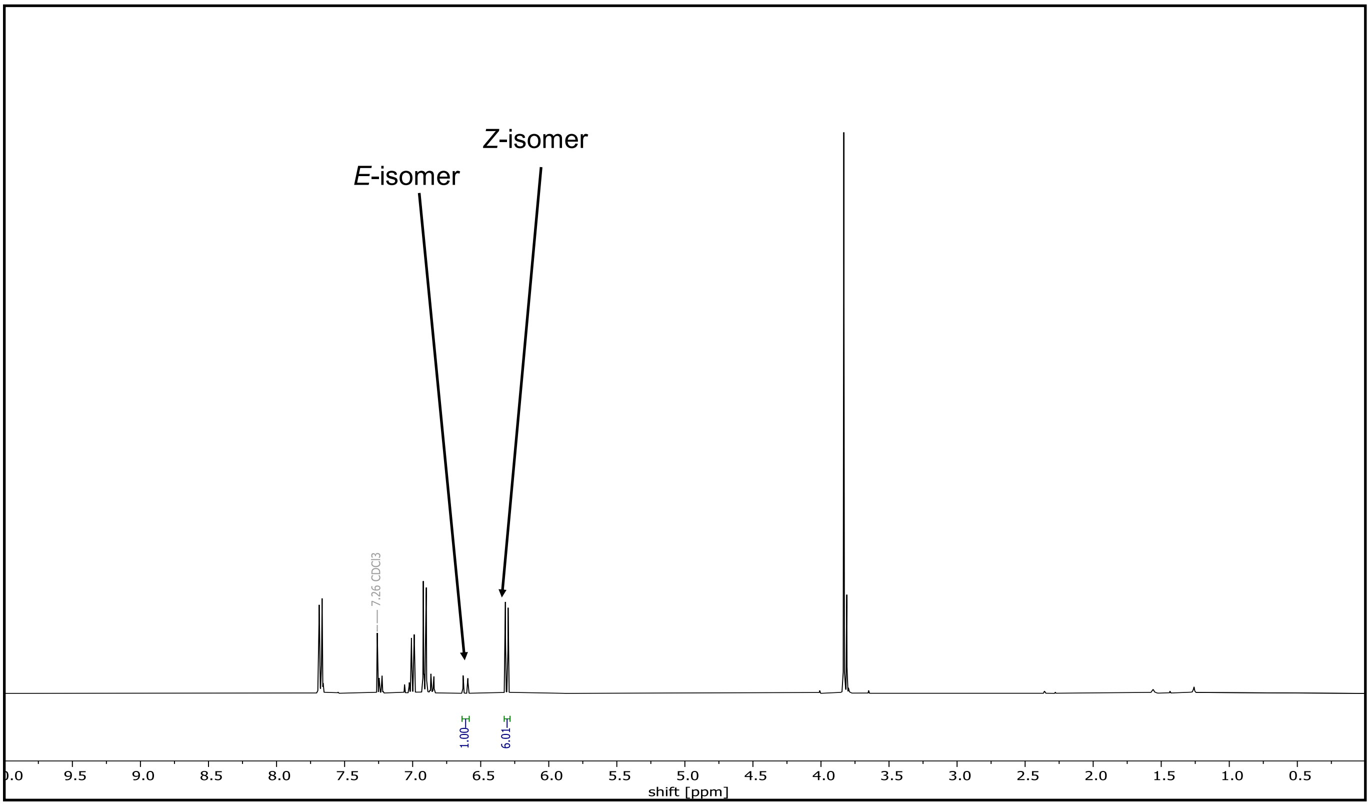

(*E*,*Z*)-1-(2-Bromovinyl)-4-methoxybenzene (*E*,*Z*)-15

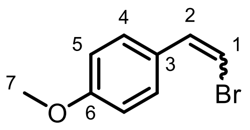

^{1}H NMR ($CDCl_3$), experiment1

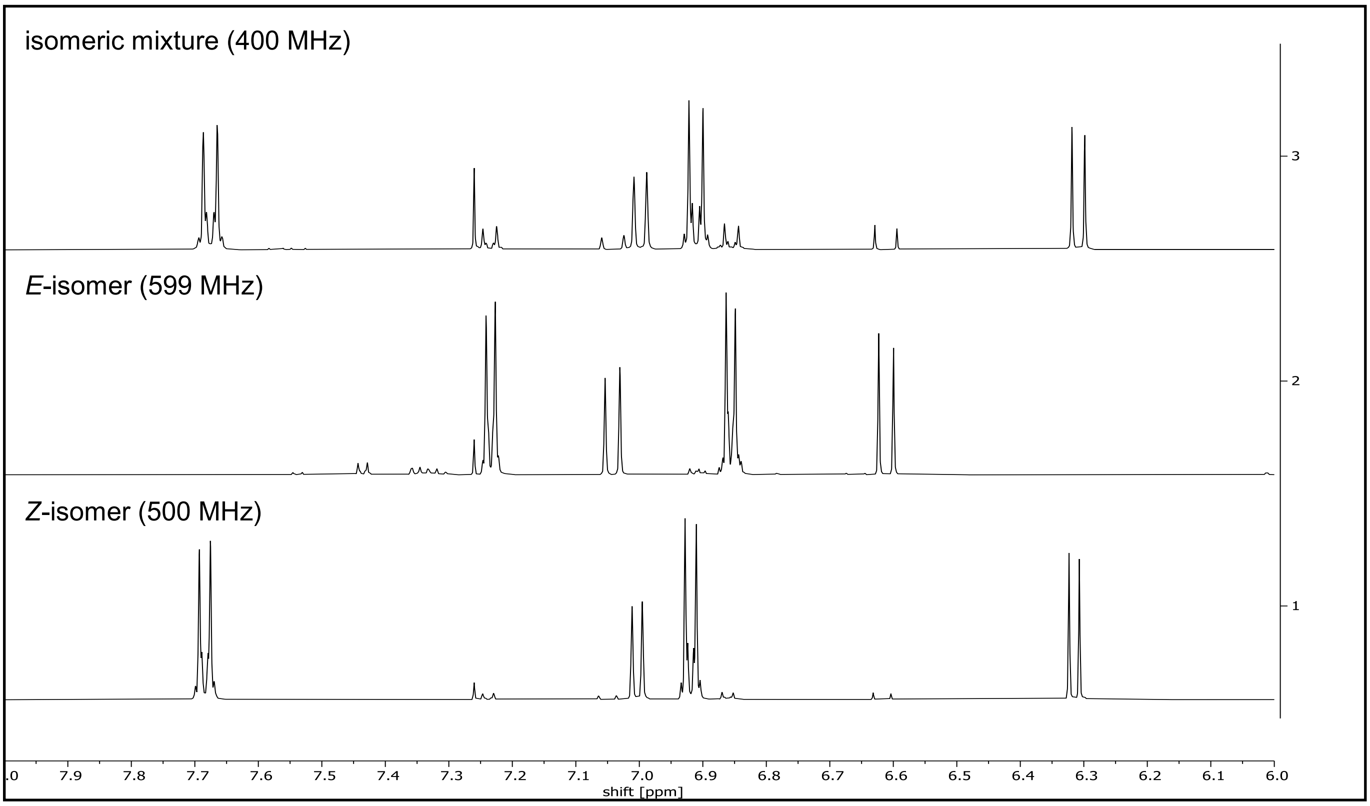

^{1}H NMR ($CDCl_3$), experiment 2

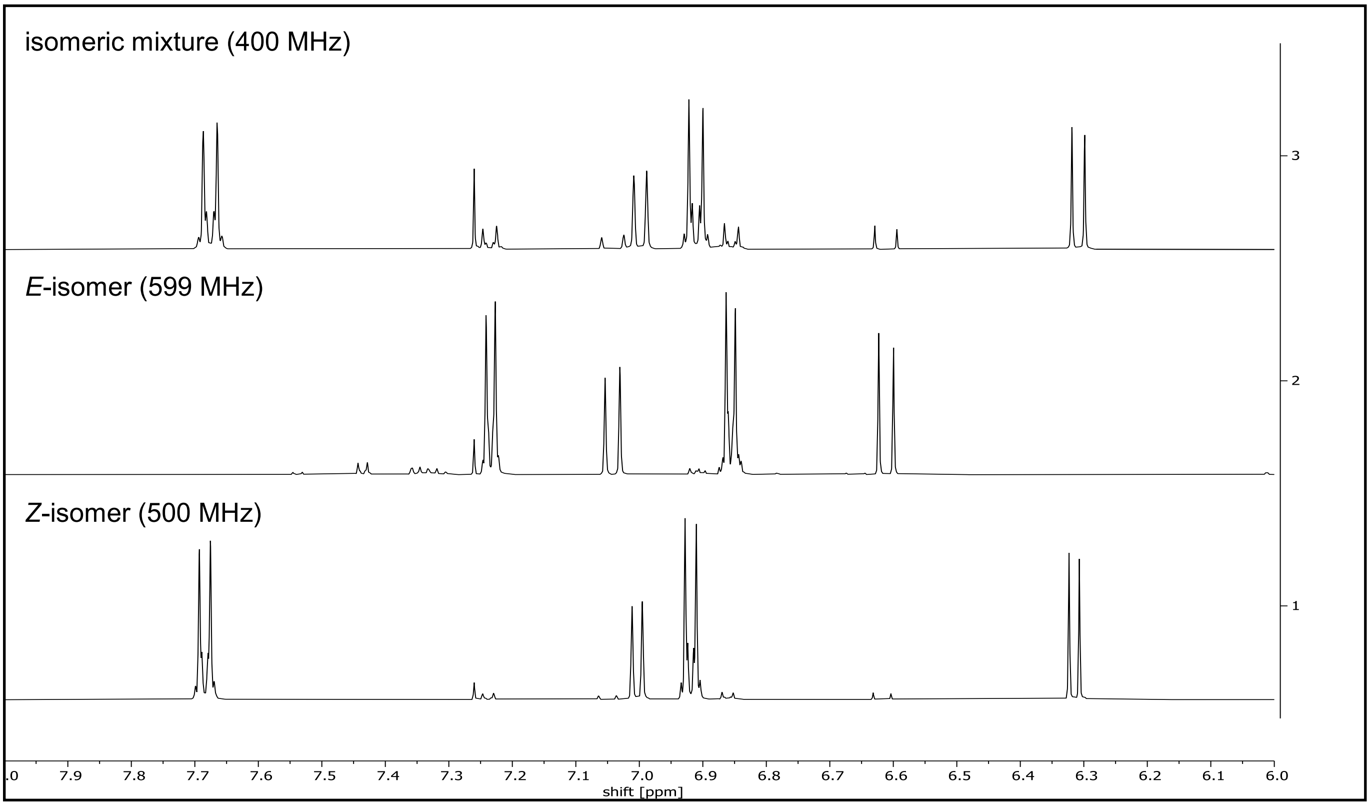

(*E*)-1-(2-Bromovinyl)-4-methoxybenzene (*E*)-15

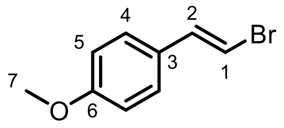

^{1}H NMR (599 MHz, $CDCl_3$)

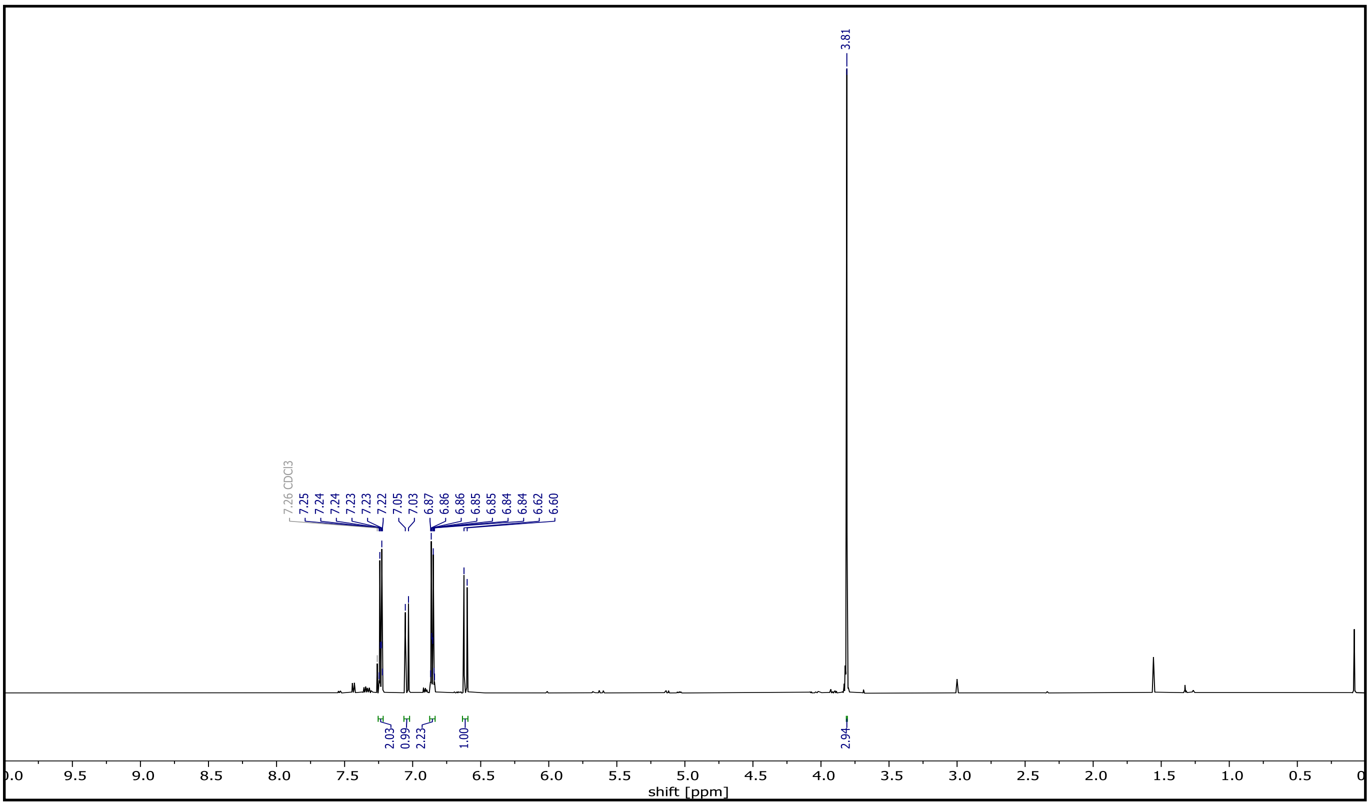

^{13}C NMR (151 MHz, $CDCl_3$)

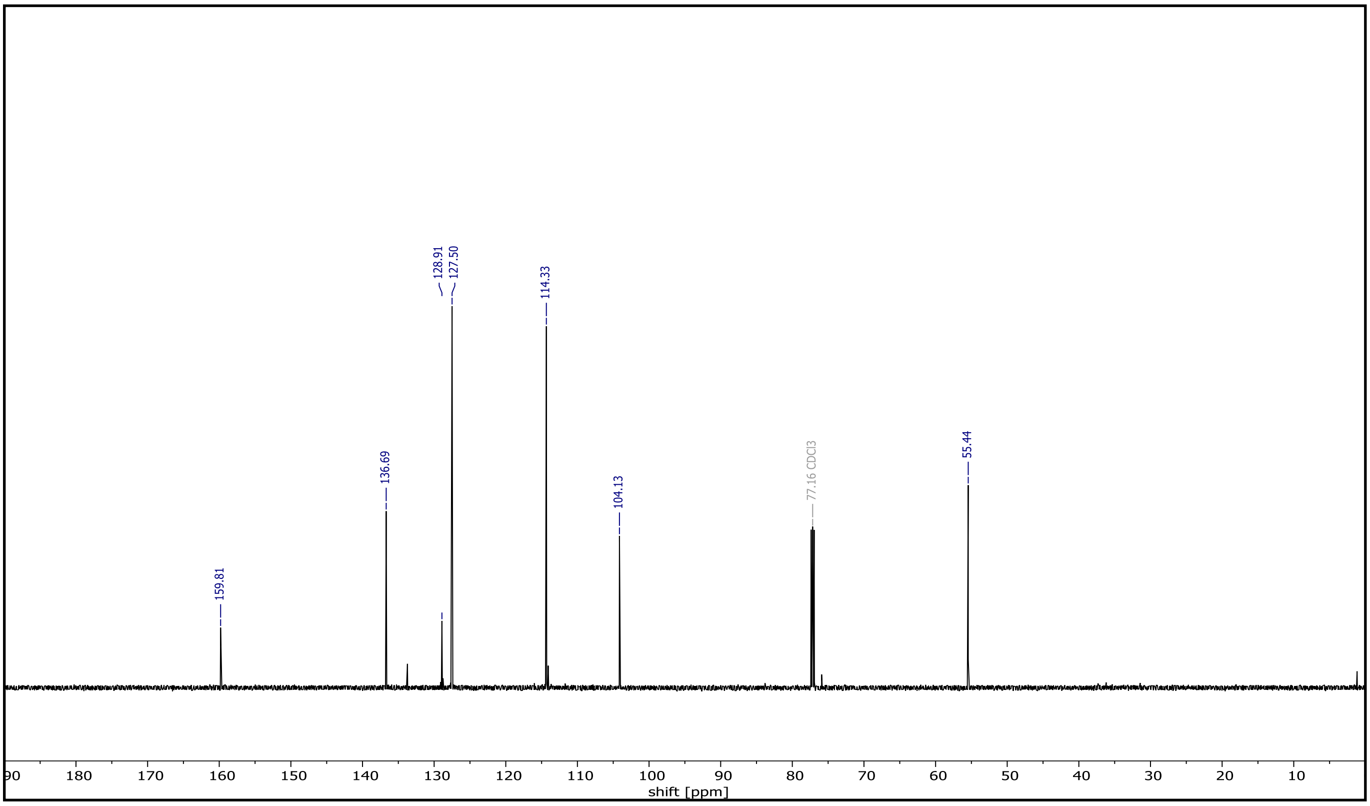

(*Z*)-1-(2-Bromovinyl)-4-methoxybenzene (*Z*)-15

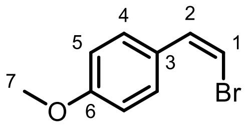

^{1}H NMR (500 MHz, $CDCl_3$)

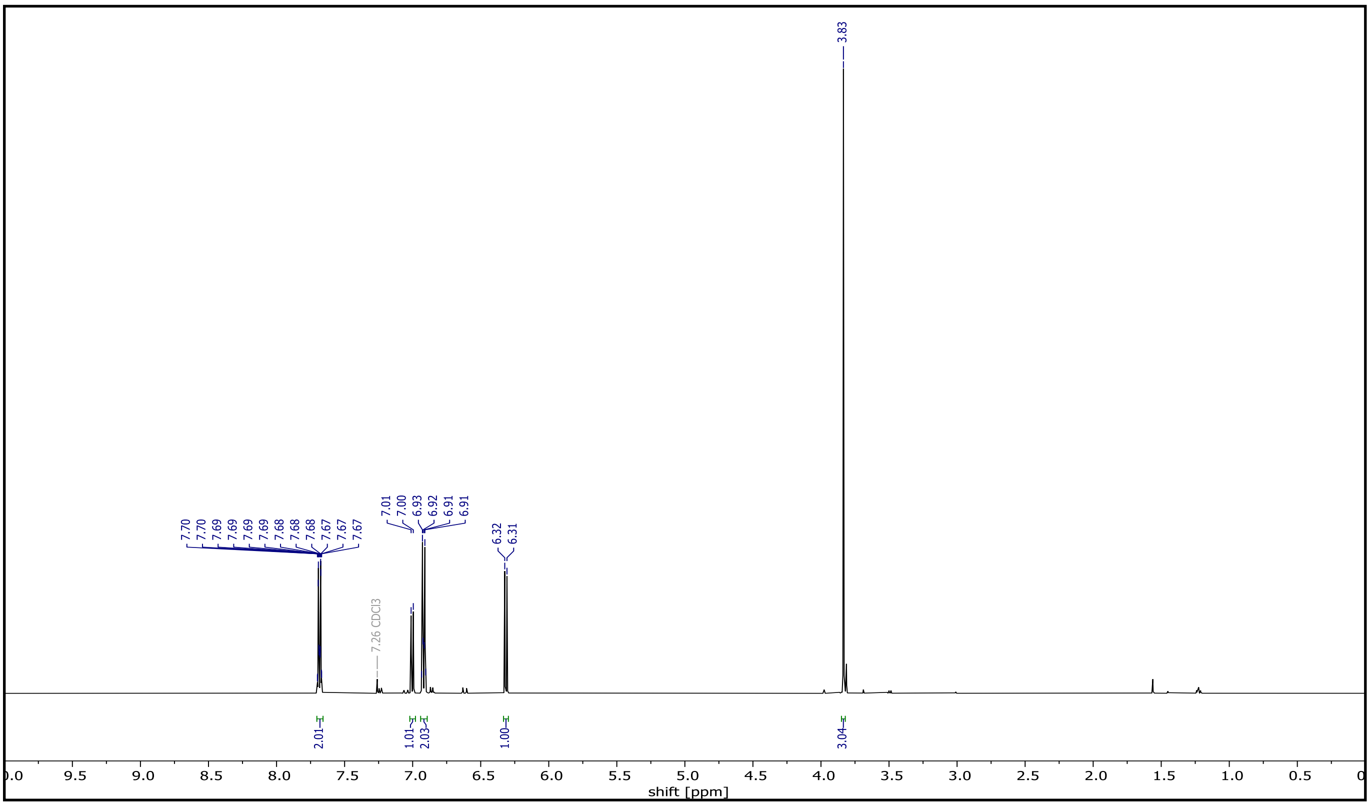

^{13}C NMR (126 MHz, $CDCl_3$)

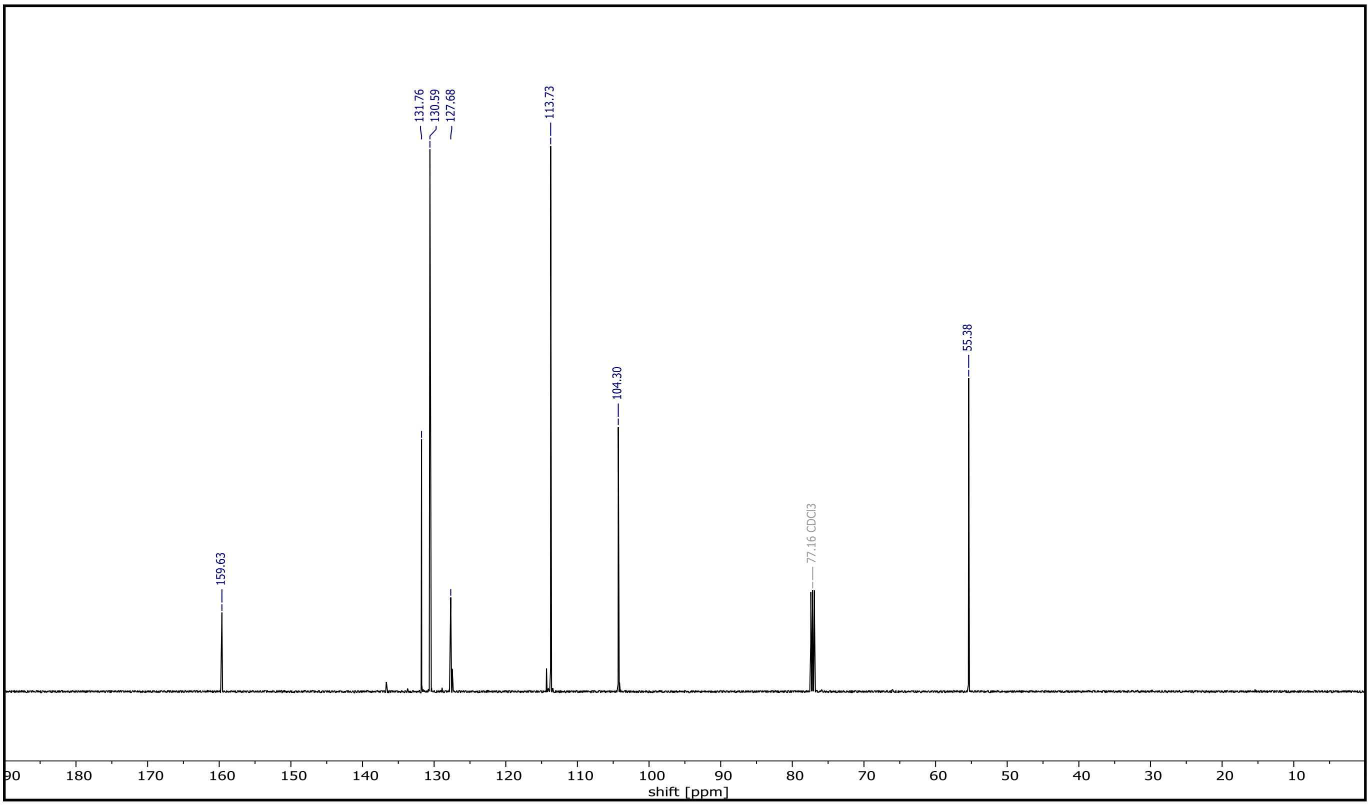

(*E*,*Z*)-5-(2-Bromovinyl)-1,2,3-trimethoxybenzene (*E*,*Z*)-16

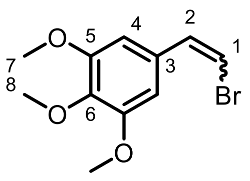

^{1}H NMR (400 MHz, $CDCl_3$), experiment 1

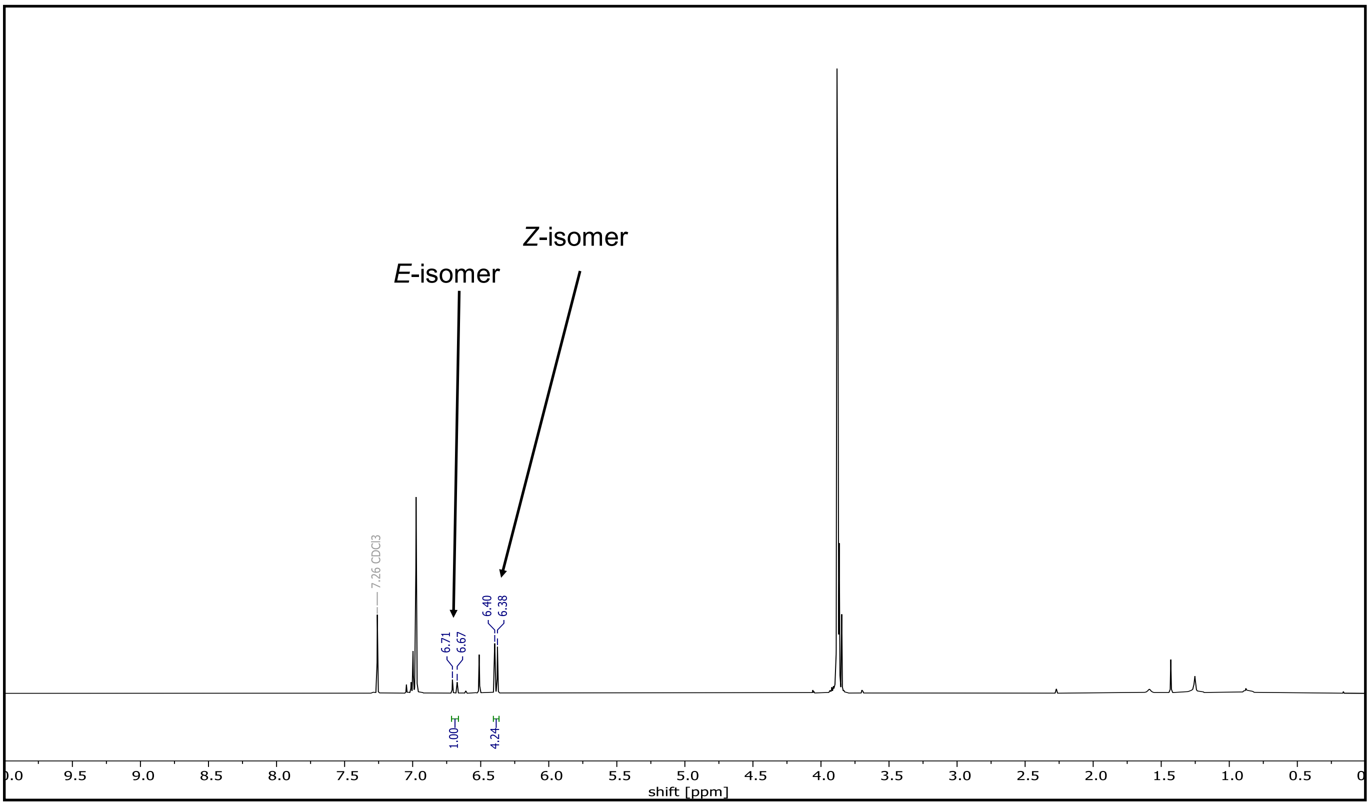

^{1}H NMR (400 MHz, $CDCl_3$), experiment 2

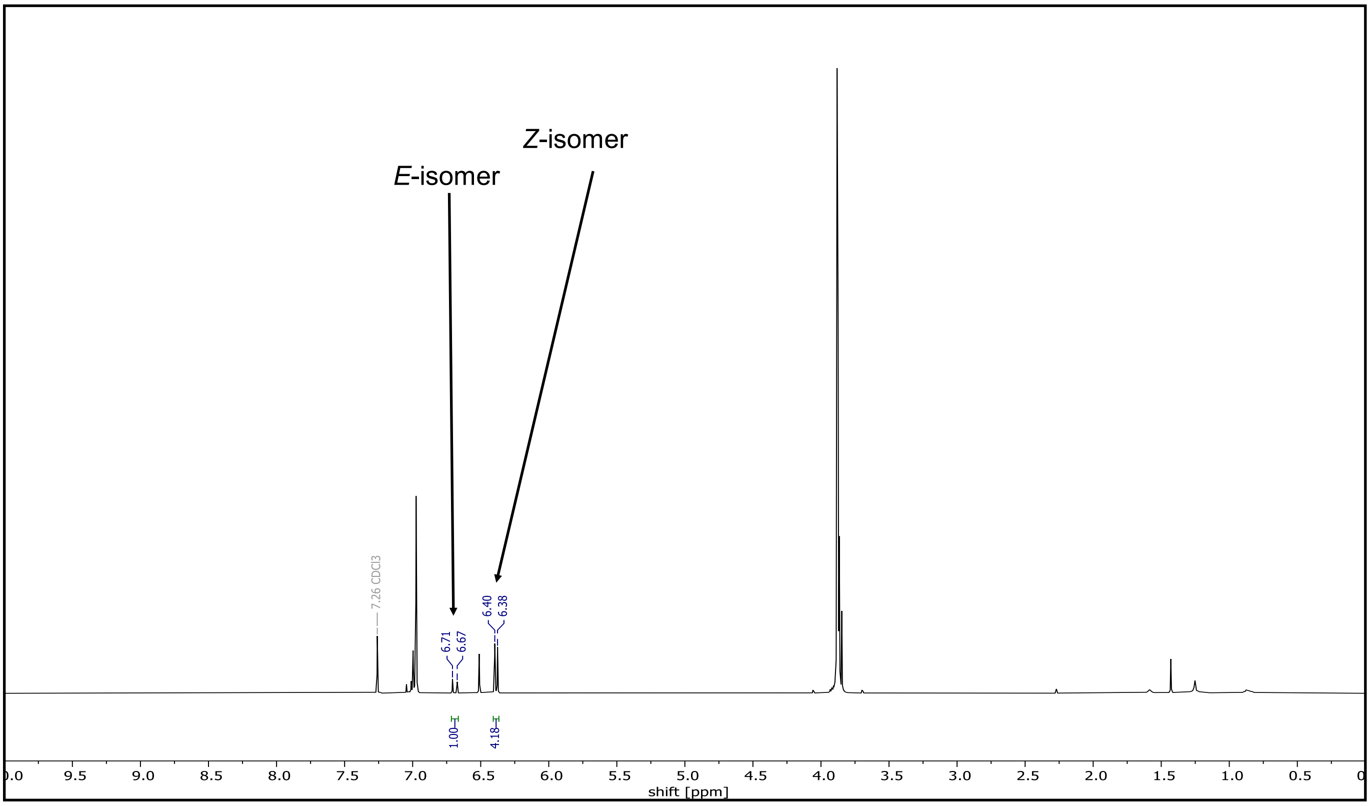

(*E*,*Z*)-5-(2-Bromovinyl)-1,2,3-trimethoxybenzene (*E*,*Z*)-16

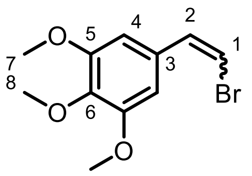

^{1}H NMR ($CDCl_3$), experiment 1

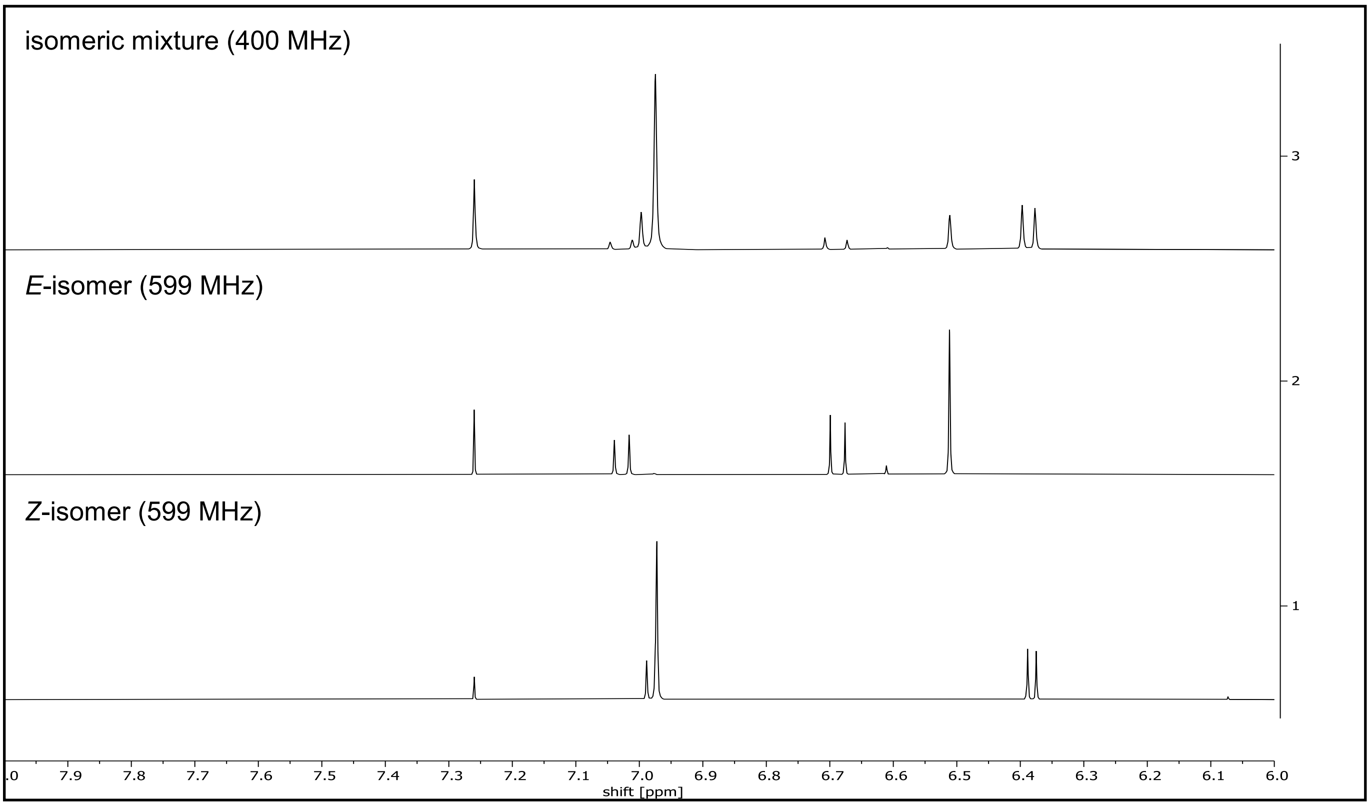

^{1}H NMR ($CDCl_3$), experiment 2

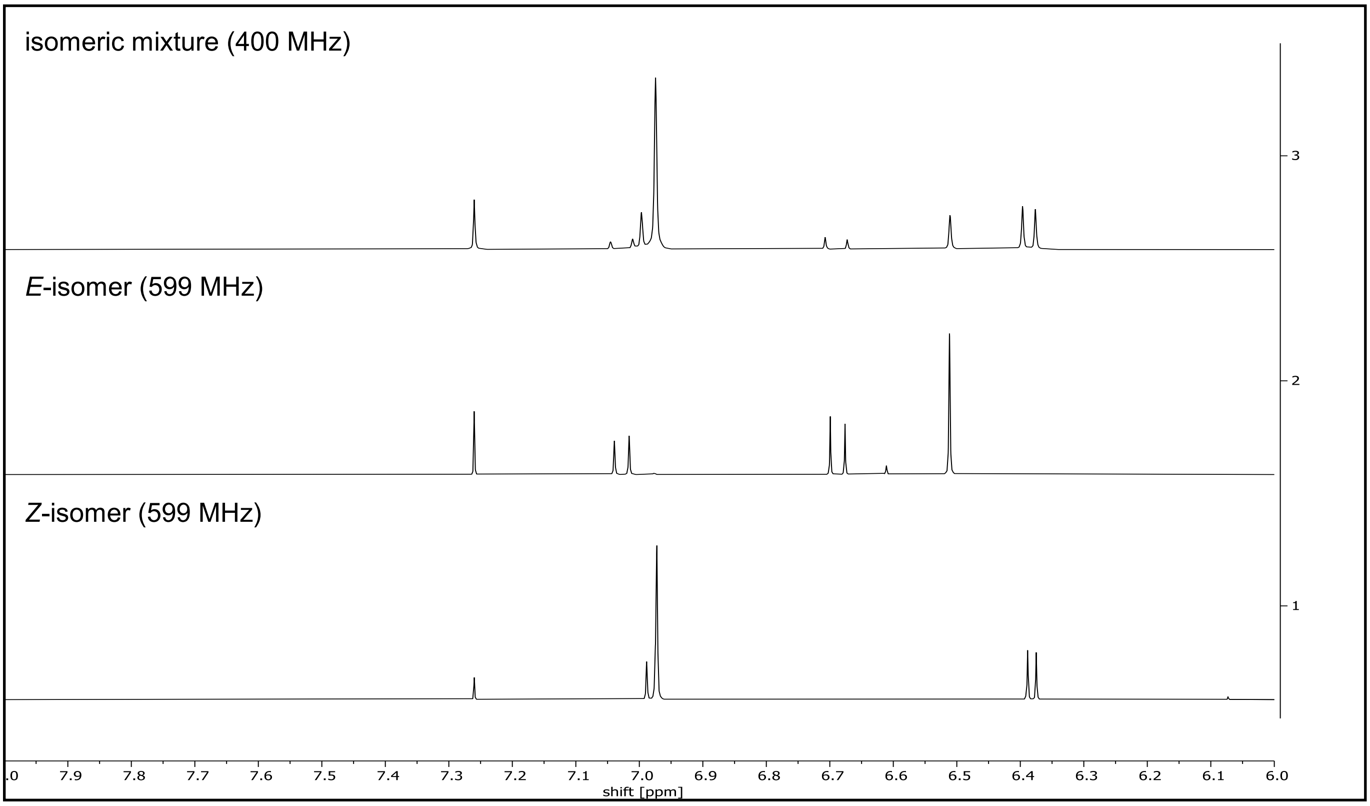

(*E*)-5-(2-Bromovinyl)-1,2,3-trimethoxybenzene (*E*)-16

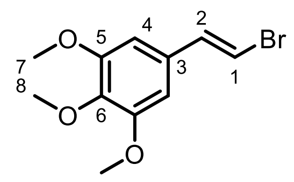

^{1}H NMR (599 MHz, $CDCl_3$)

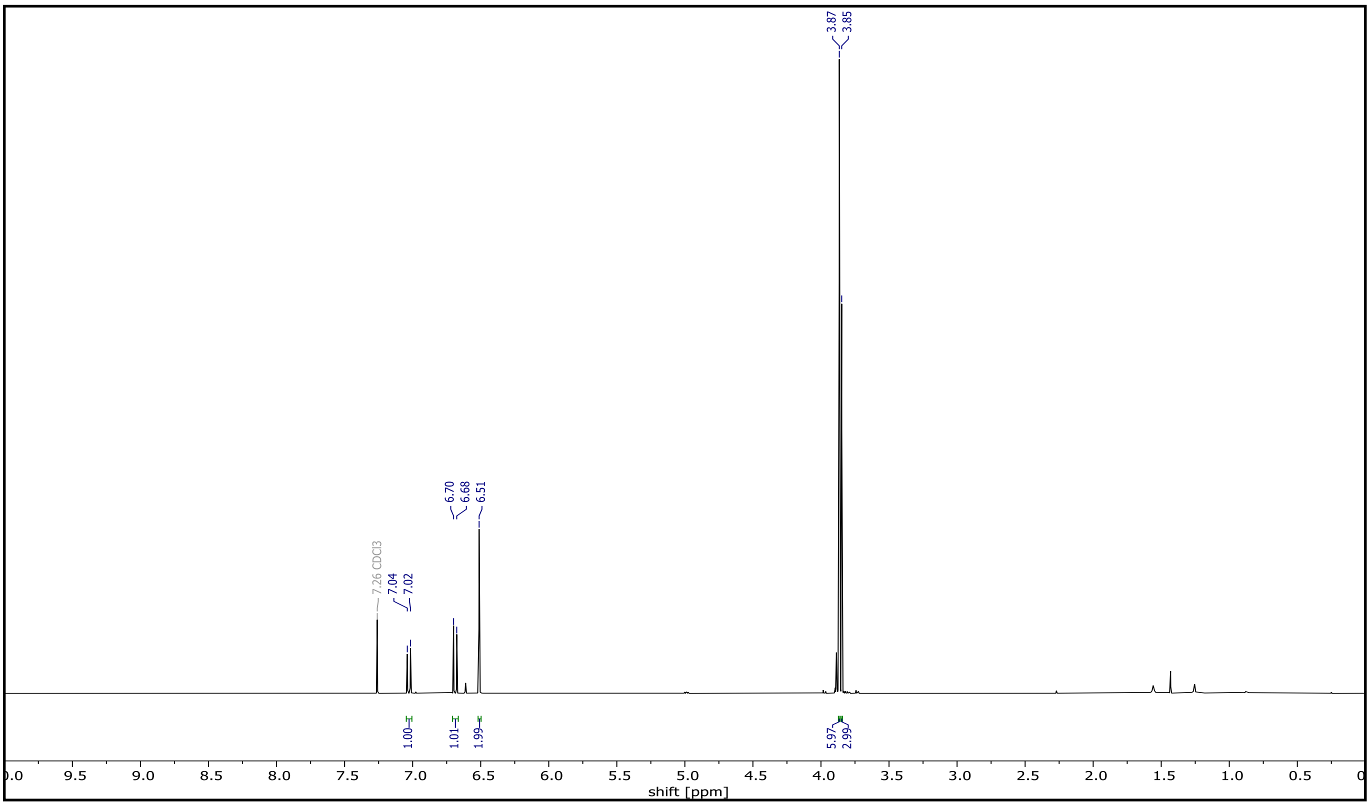

$^{13}C\{^1H\}$ NMR (151 MHz, $CDCl_3$)

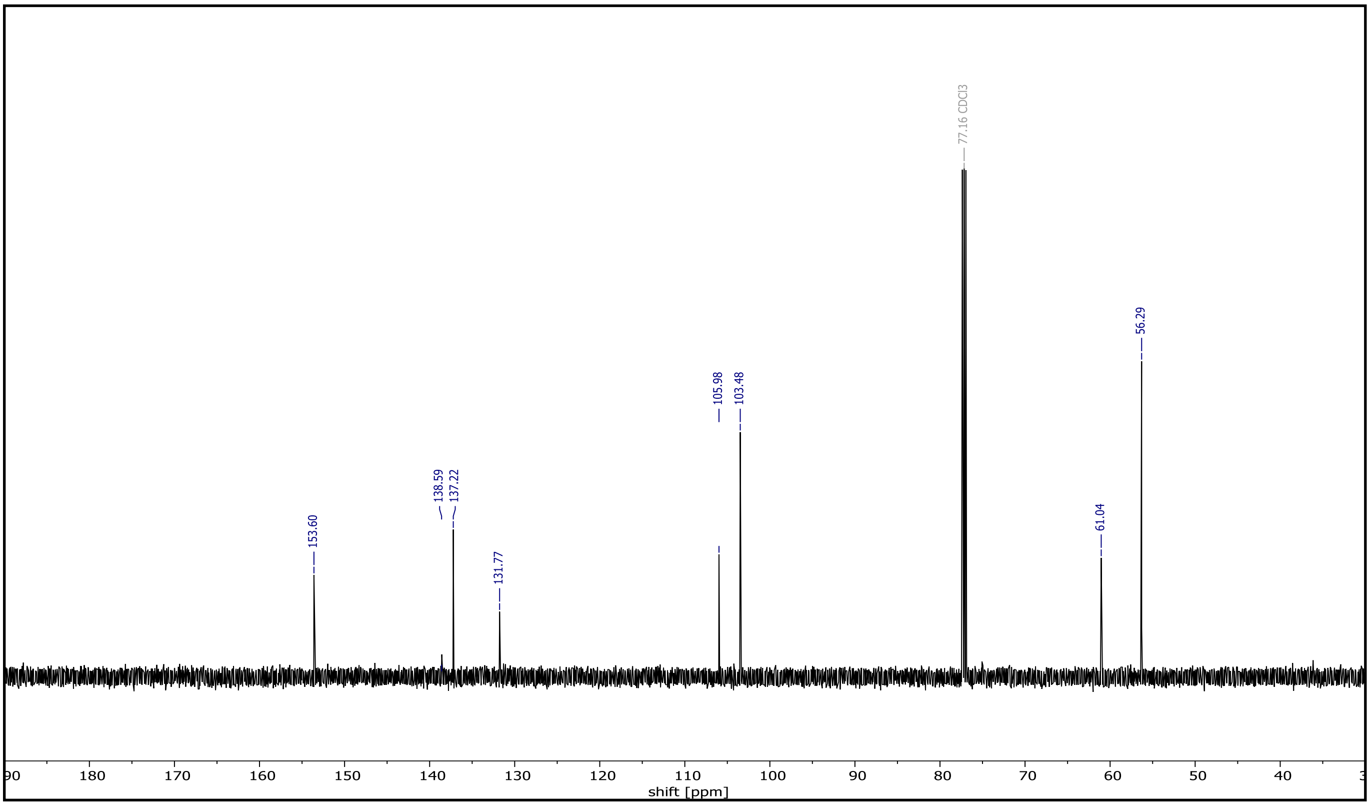

(*Z*)-5-(2-Bromovinyl)-1,2,3-trimethoxybenzene (*Z*)-16

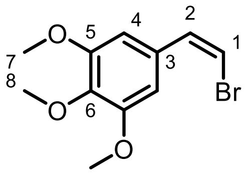

^1H NMR (599 MHz, $CDCl_3$)

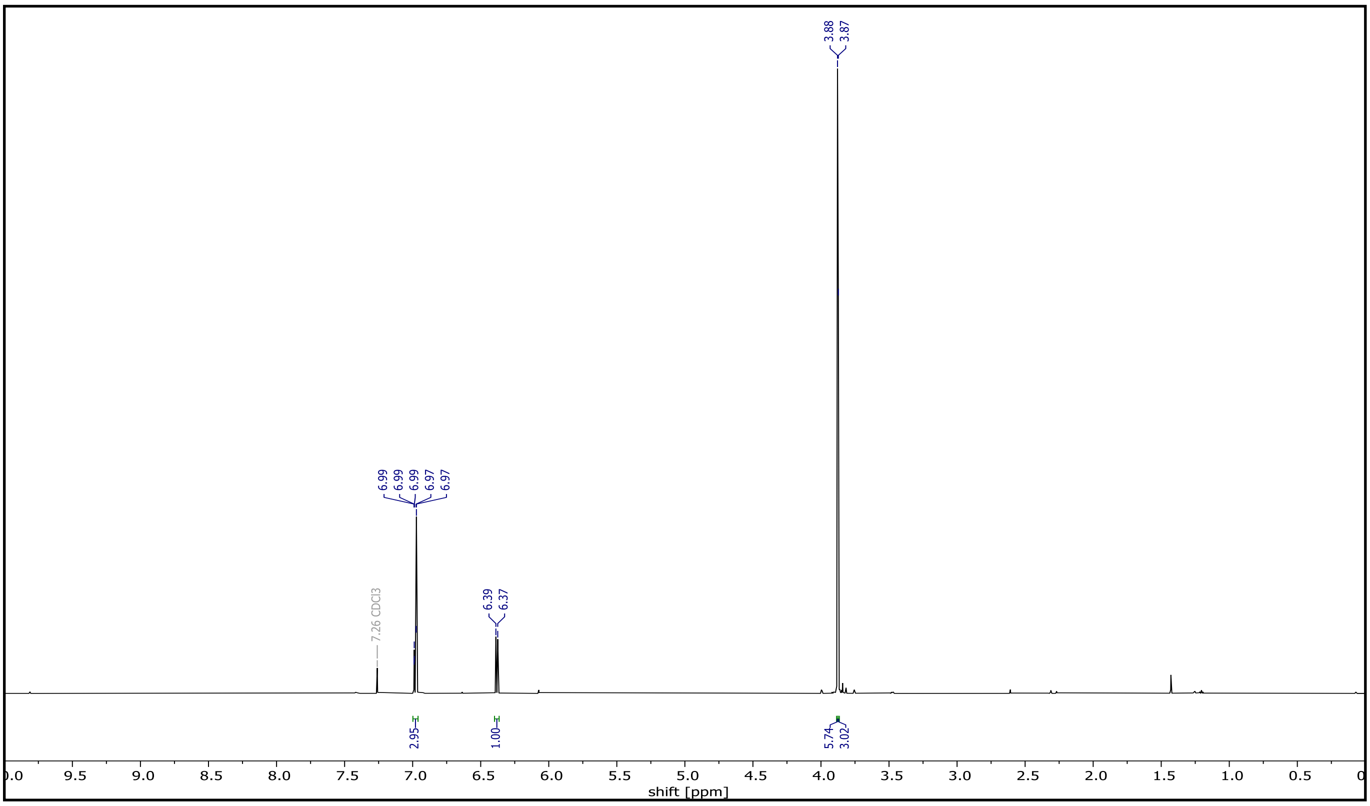

$^{13}C\{^1H\}$ NMR (151 MHz, $CDCl_3$)

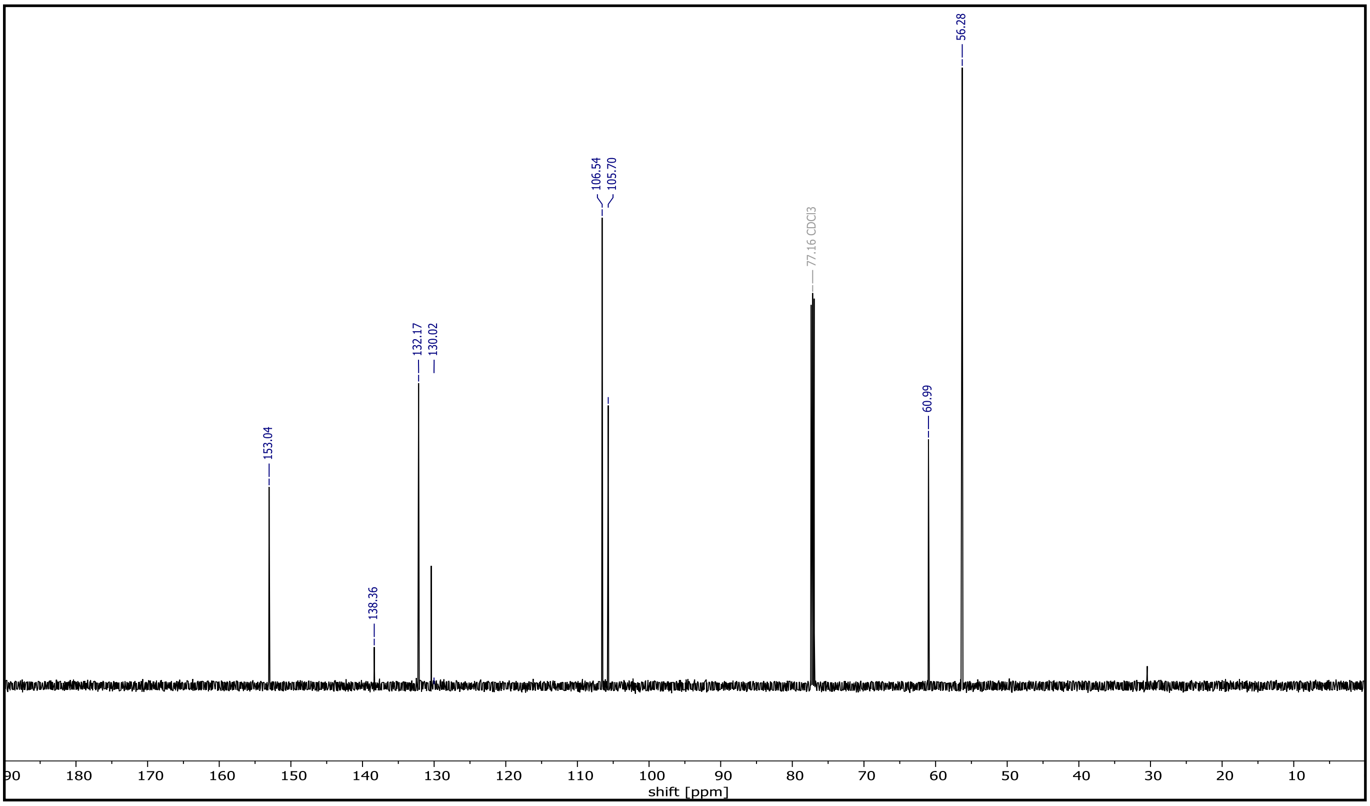

(*E*,*Z*)-5-(2-Bromovinyl)benzo[*d*]1,3]dioxole (*E*,*Z*)-17

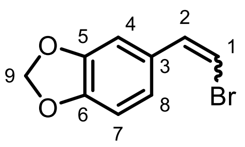

^{1}H NMR (400 MHz, $CDCl_3$), experiment 1

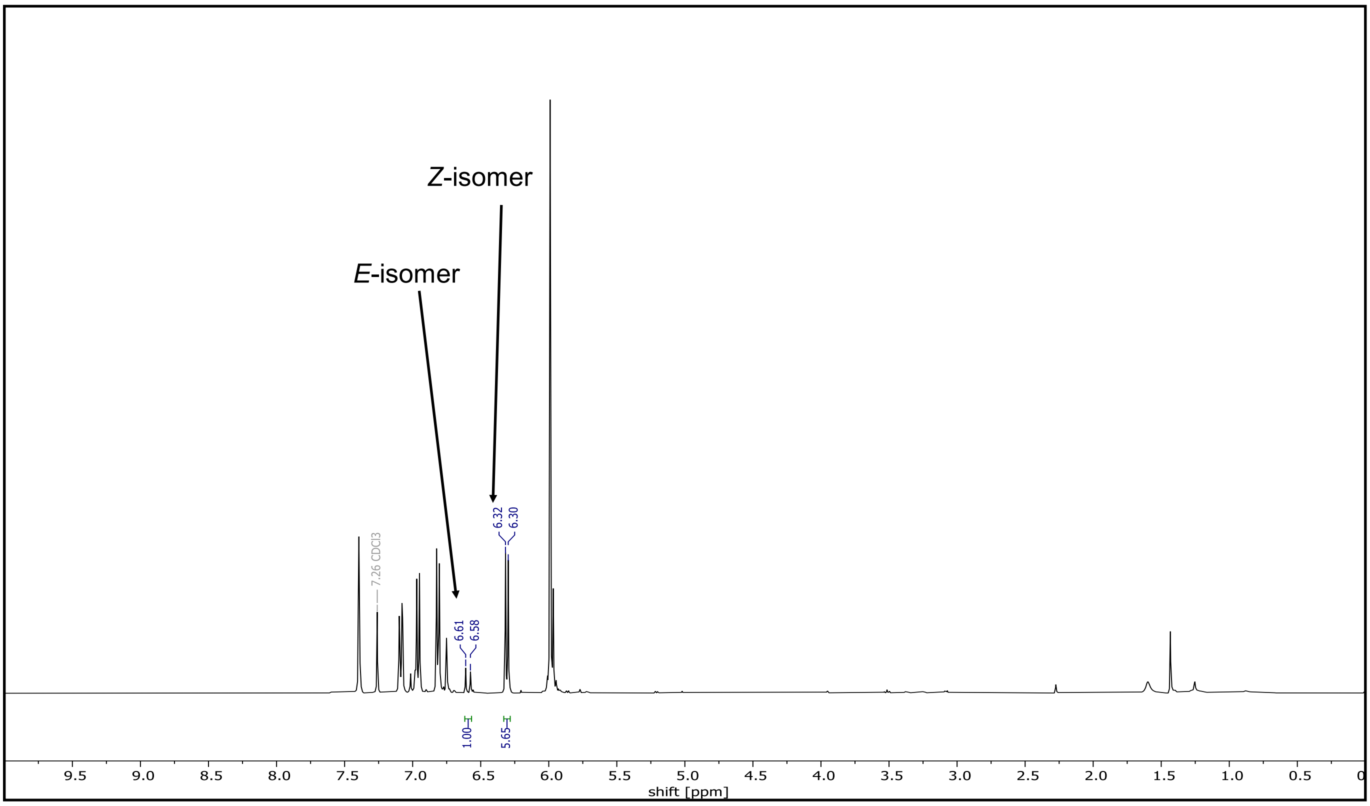

^{1}H NMR (400 MHz, $CDCl_3$), experiment 2

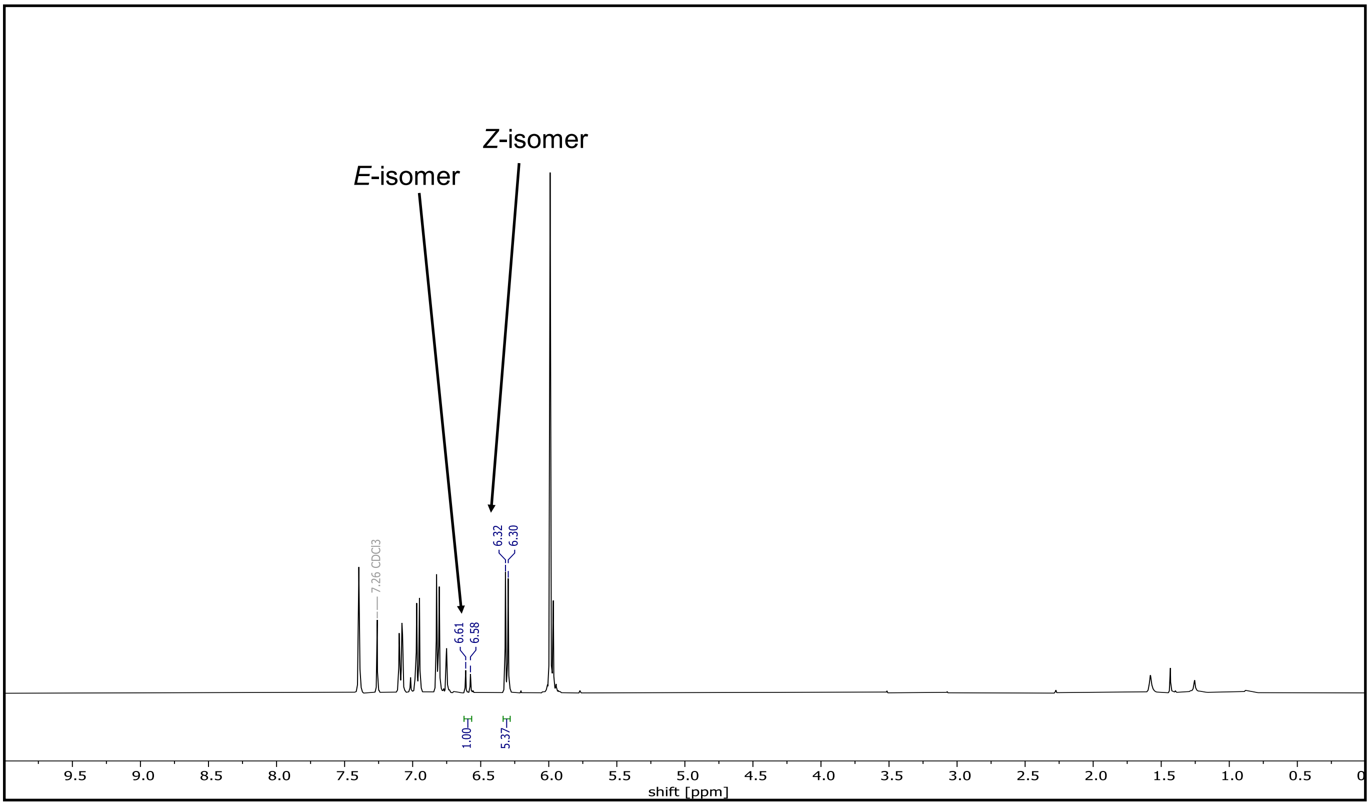

(*E*,*Z*)-5-(2-Bromovinyl)benzo[*d*]1,3]dioxole (*E*,*Z*)-17

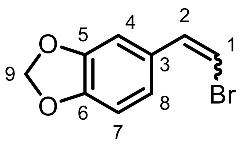

^{1}H NMR ($CDCl_3$), experiment 1

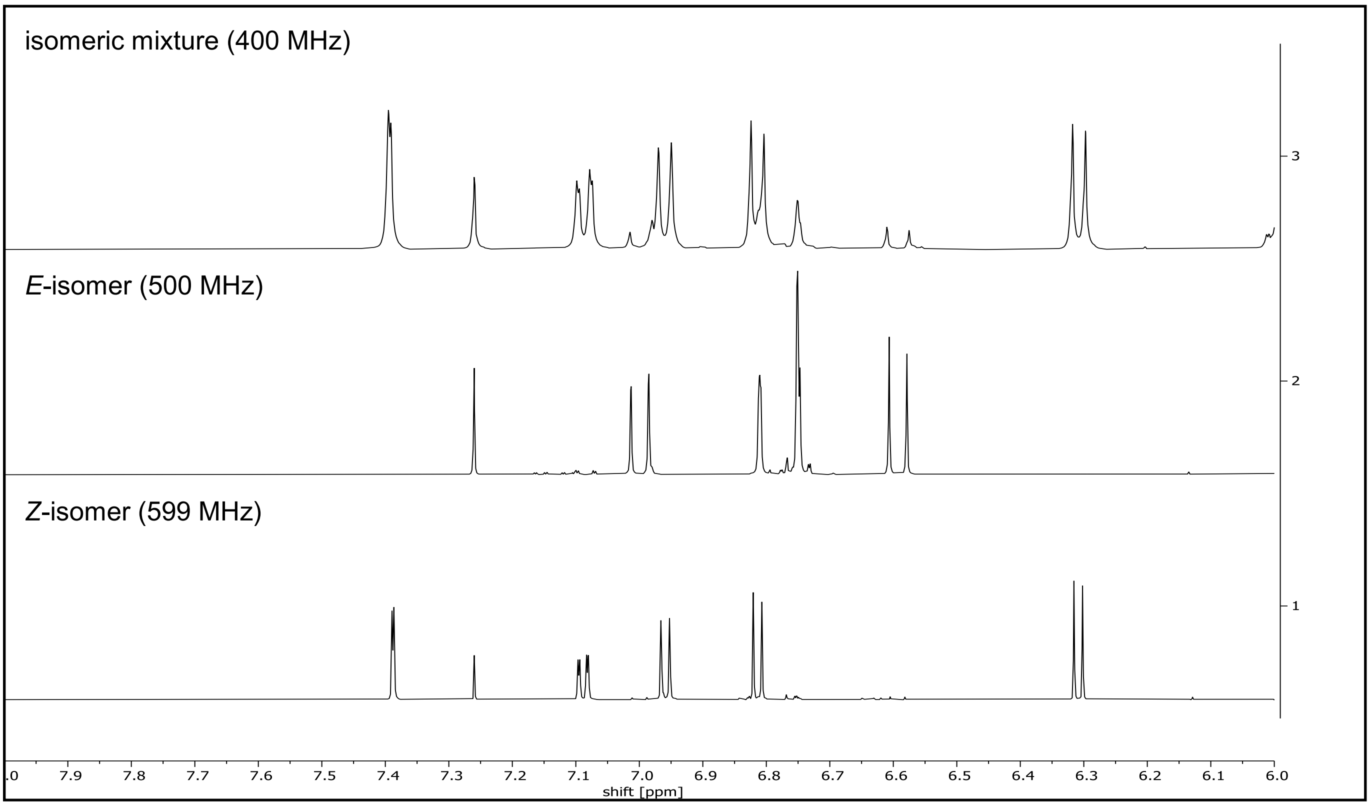

^{1}H NMR ($CDCl_3$), experiment 2

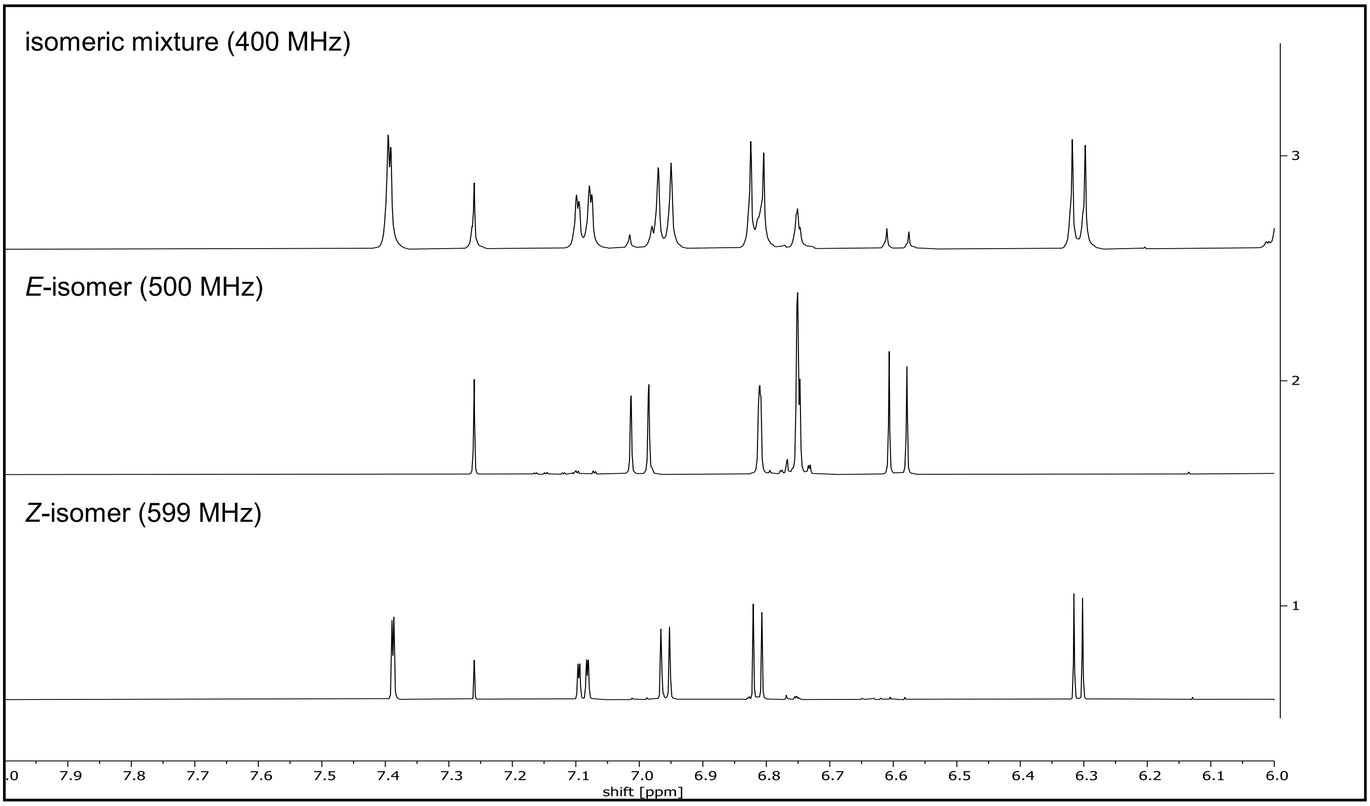

(*E*)-5-(2-Bromovinyl)benzo[*d*][1,3]dioxole (*E*)-17

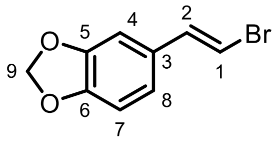

^{1}H NMR (500 MHz, $CDCl_3$)

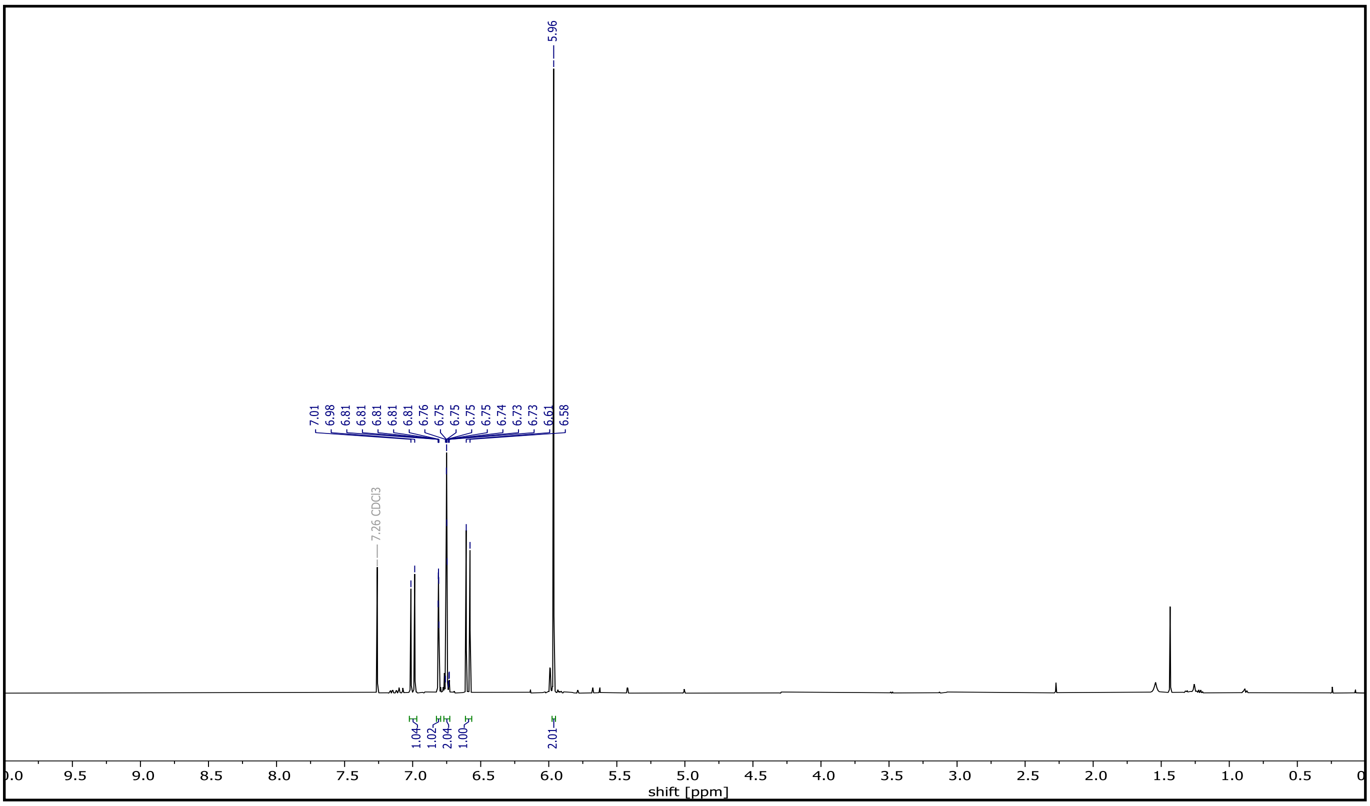

$^{13}C\{^{1}H\}$ NMR (126 MHz, $CDCl_3$)

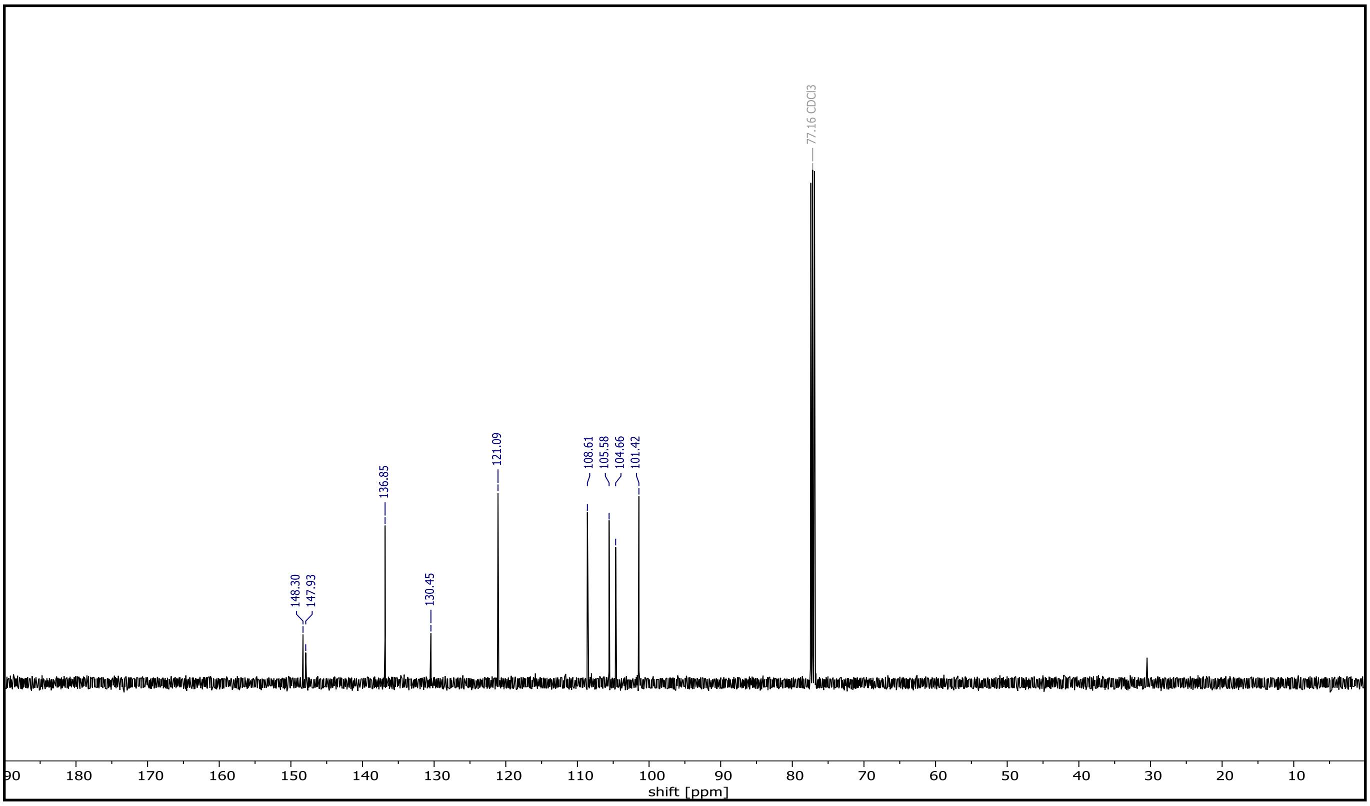

(Z)-5-(2-Bromovinyl)benzo[d][1,3]dioxole (Z)-17

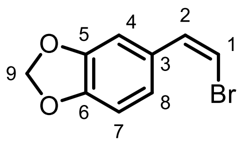

^{1}H NMR (599 MHz, $CDCl_3$)

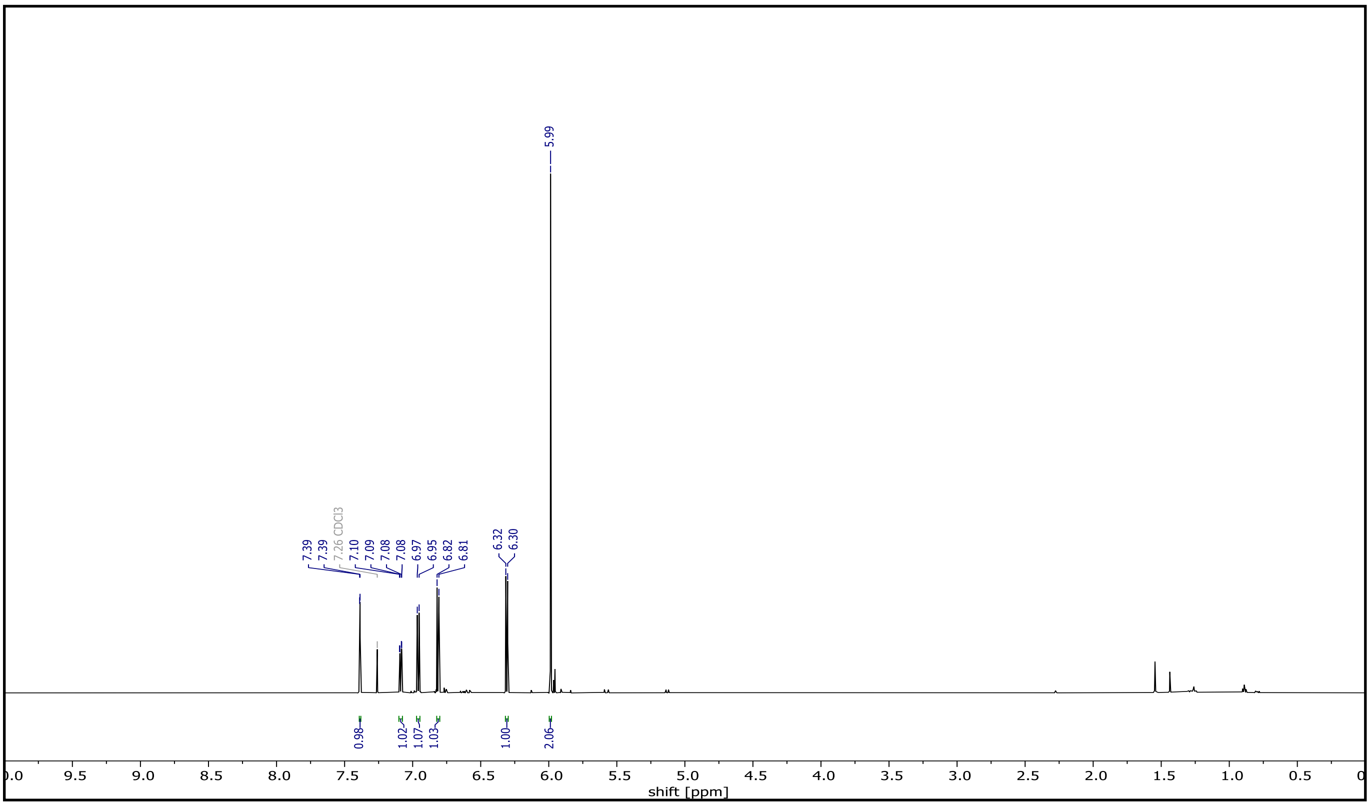

^{13}C{^{1}H} NMR (151 MHz, $CDCl_3$)

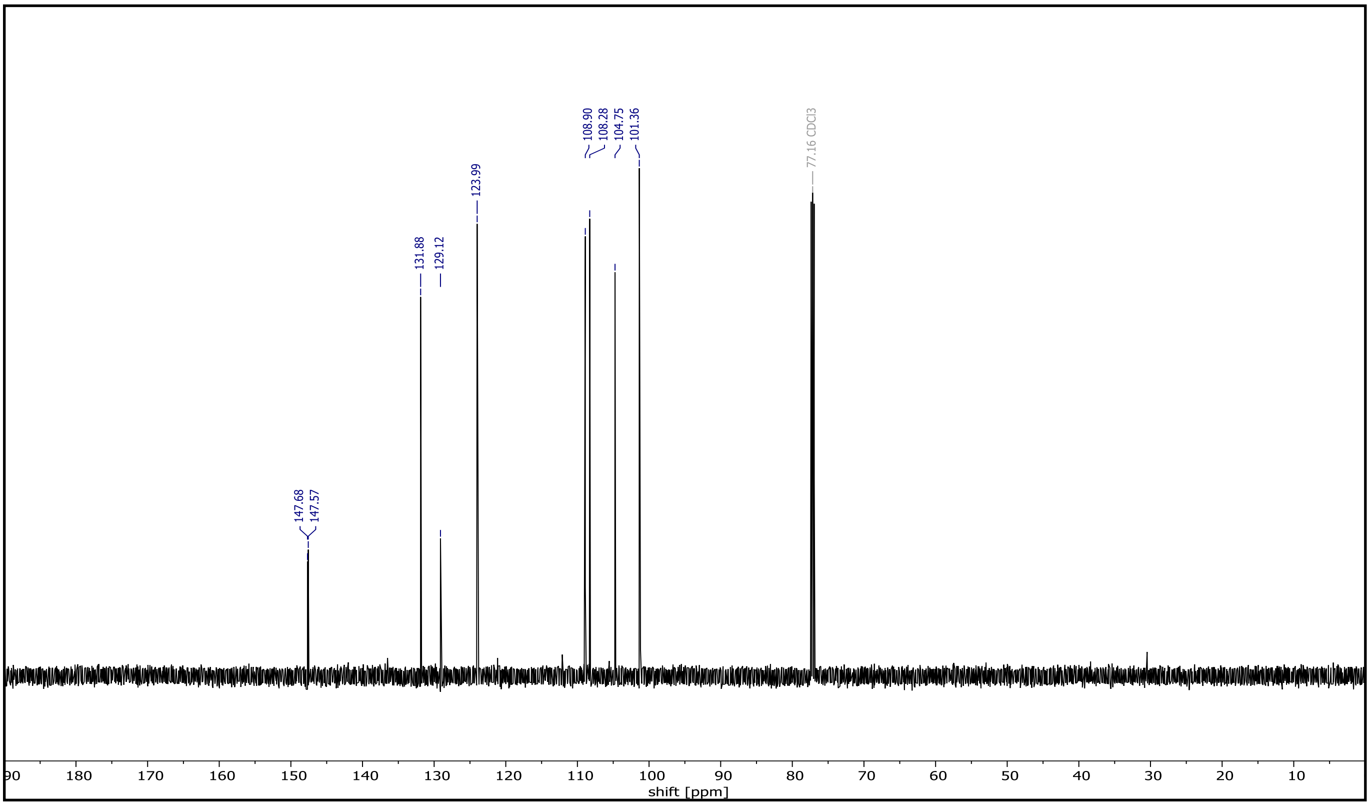

(*Z*)-1-Methoxy-4-(3-phenylprop-1-en-1-yl)benzene (*Z*)-18

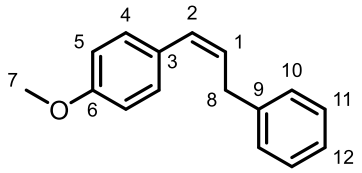

^{1}H-NMR (500 MHz, $CDCl_3$)

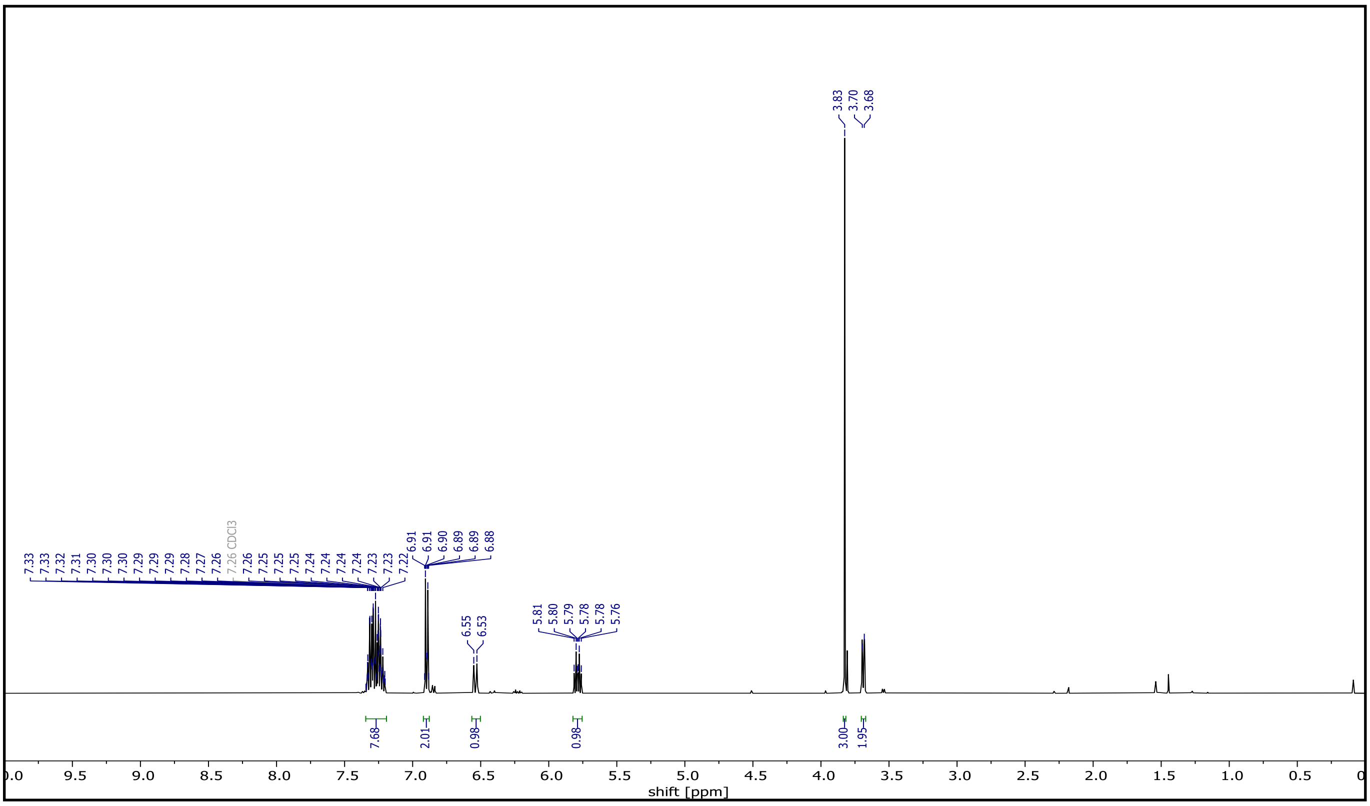

^{13}C-NMR (126 MHz, $CDCl_3$),

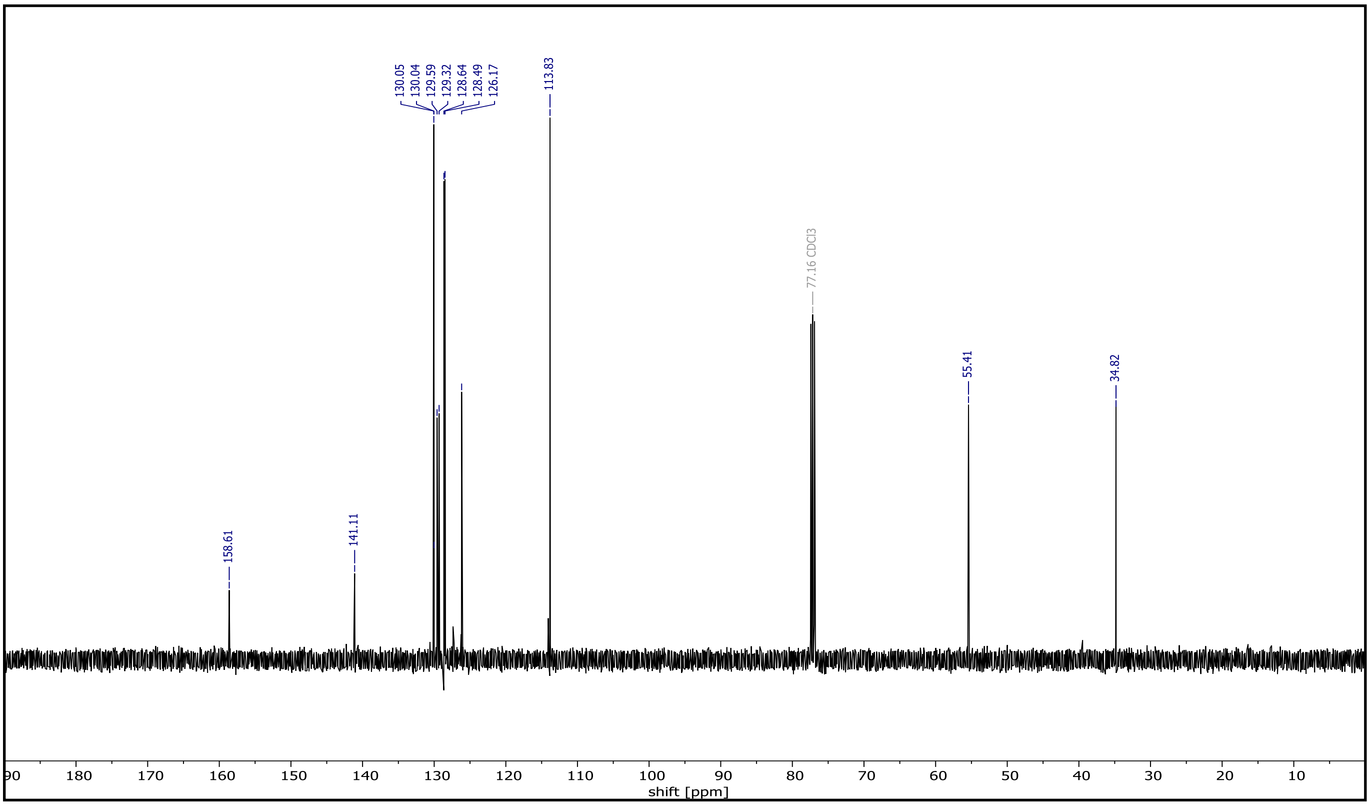

(*Z*,*E*)-4-(Vinyl-2-d)-1,1'-biphenyl (*Z*,*E*)-19 (*Z*:*E* = 77:23)

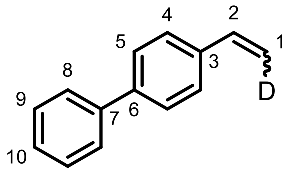

^{1}H-NMR (599 MHz, $CDCl_3$)

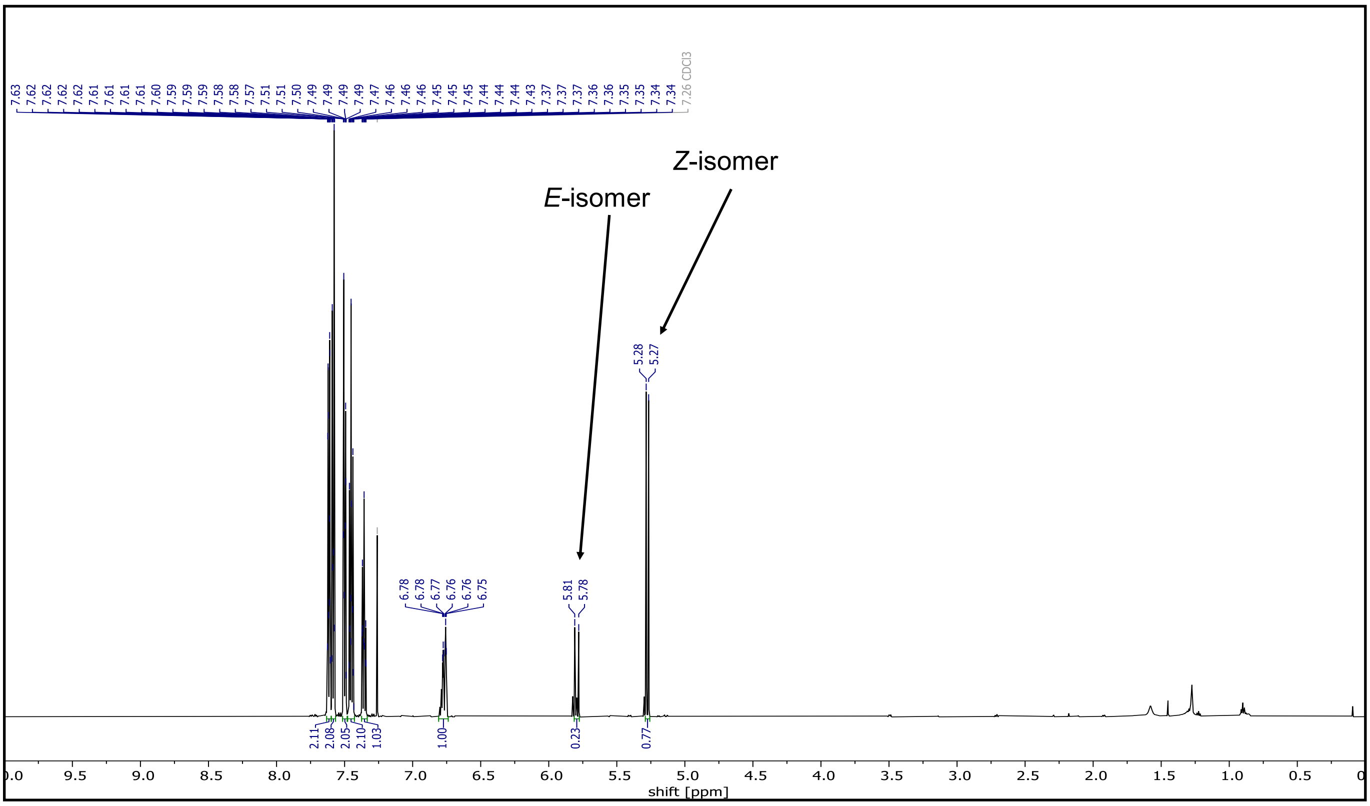

^{13}C-NMR (151 MHz, $CDCl_3$)

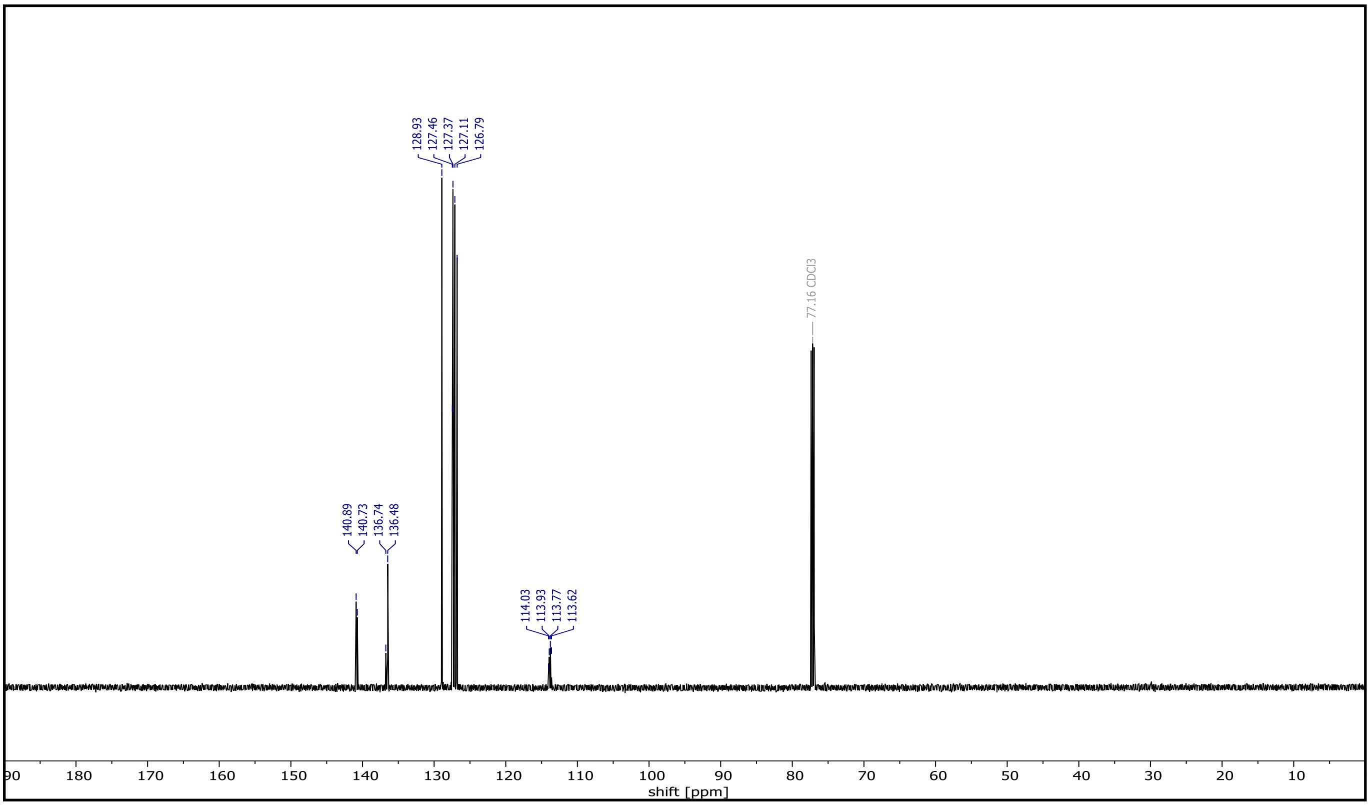

(*Z*,*E*)-4-(2-Chlorovinyl)-1,1'-biphenyl (*Z*,*E*)-20 (*Z*:*E* = 80:20)

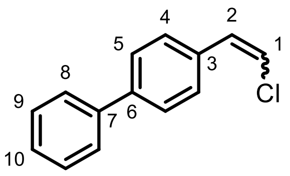

^{1}H-NMR (599 MHz, $CDCl_3$)

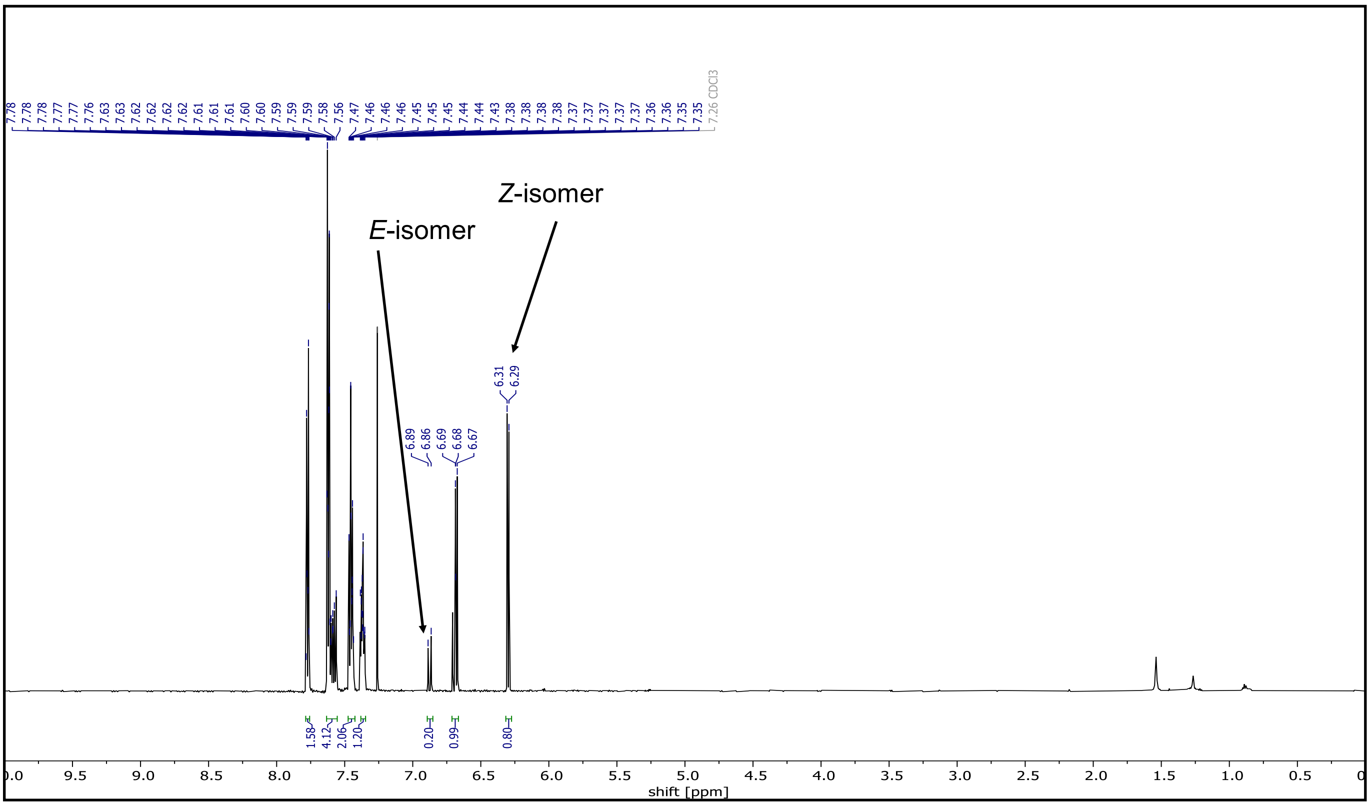

^{13}C-NMR (151 MHz, $CDCl_3$)

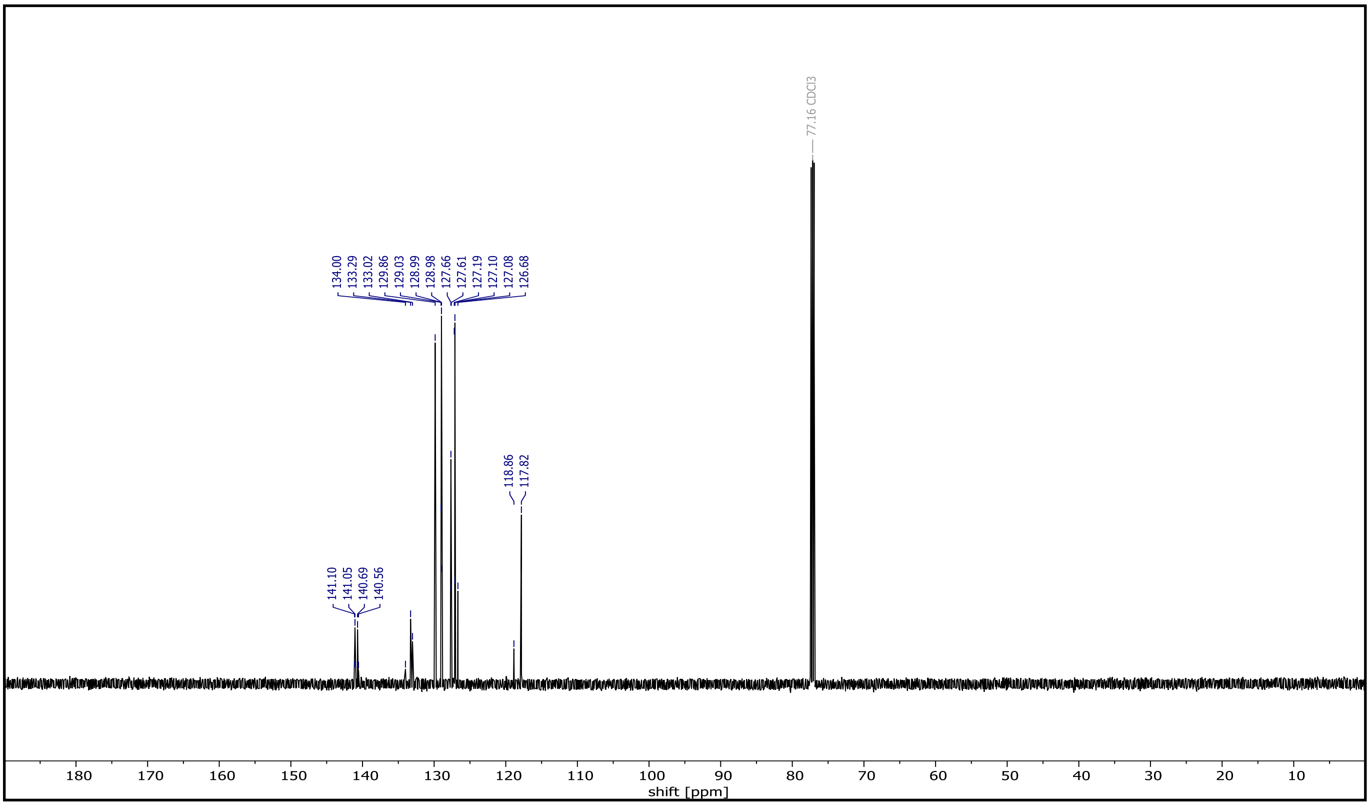

(*Z*,*E*)-4-(2-Iodovinyl)-1,1'-biphenyl (*Z*,*E*)-21 (*Z*:*E* = 80:20)

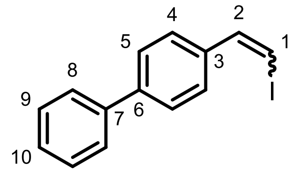

^{1}H-NMR (599 MHz, $CDCl_3$)

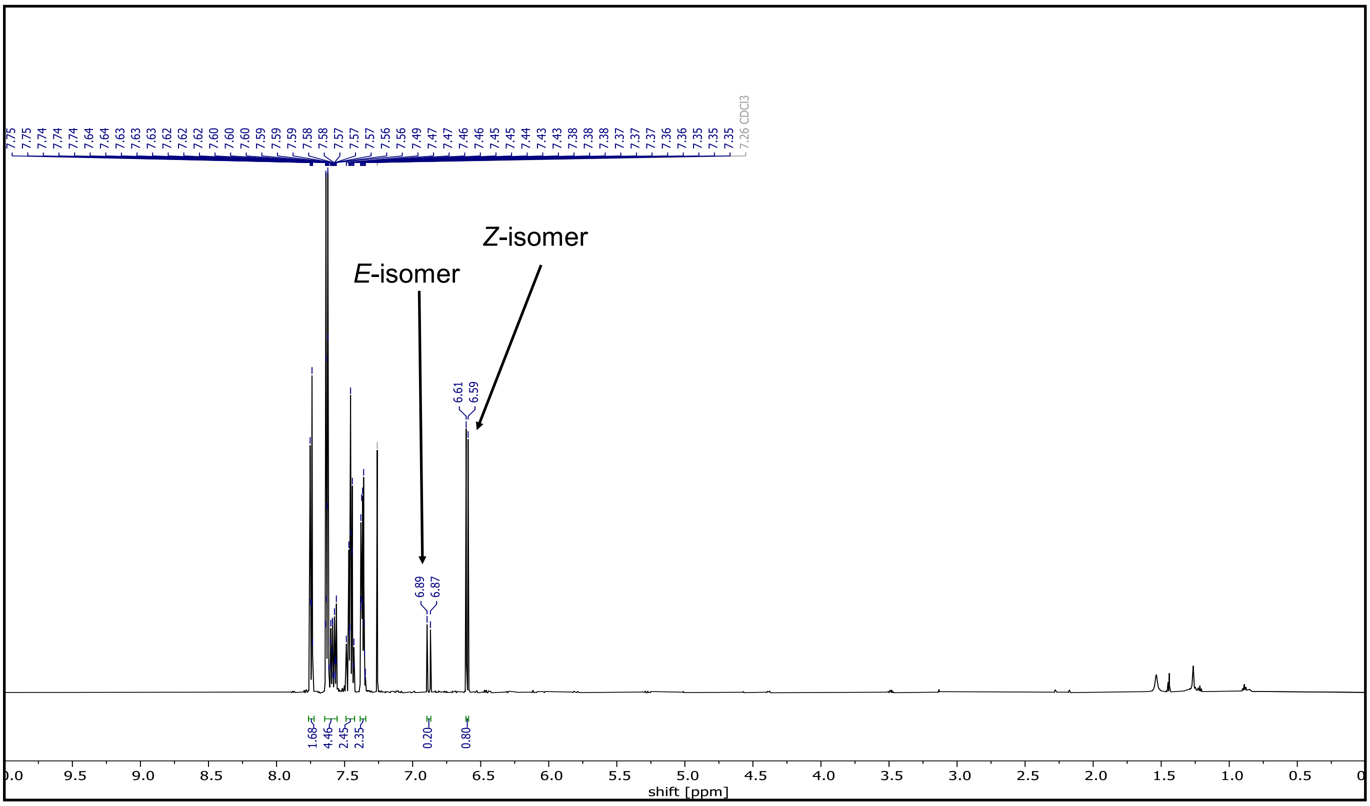

^{13}C-NMR (151 MHz, $CDCl_3$)

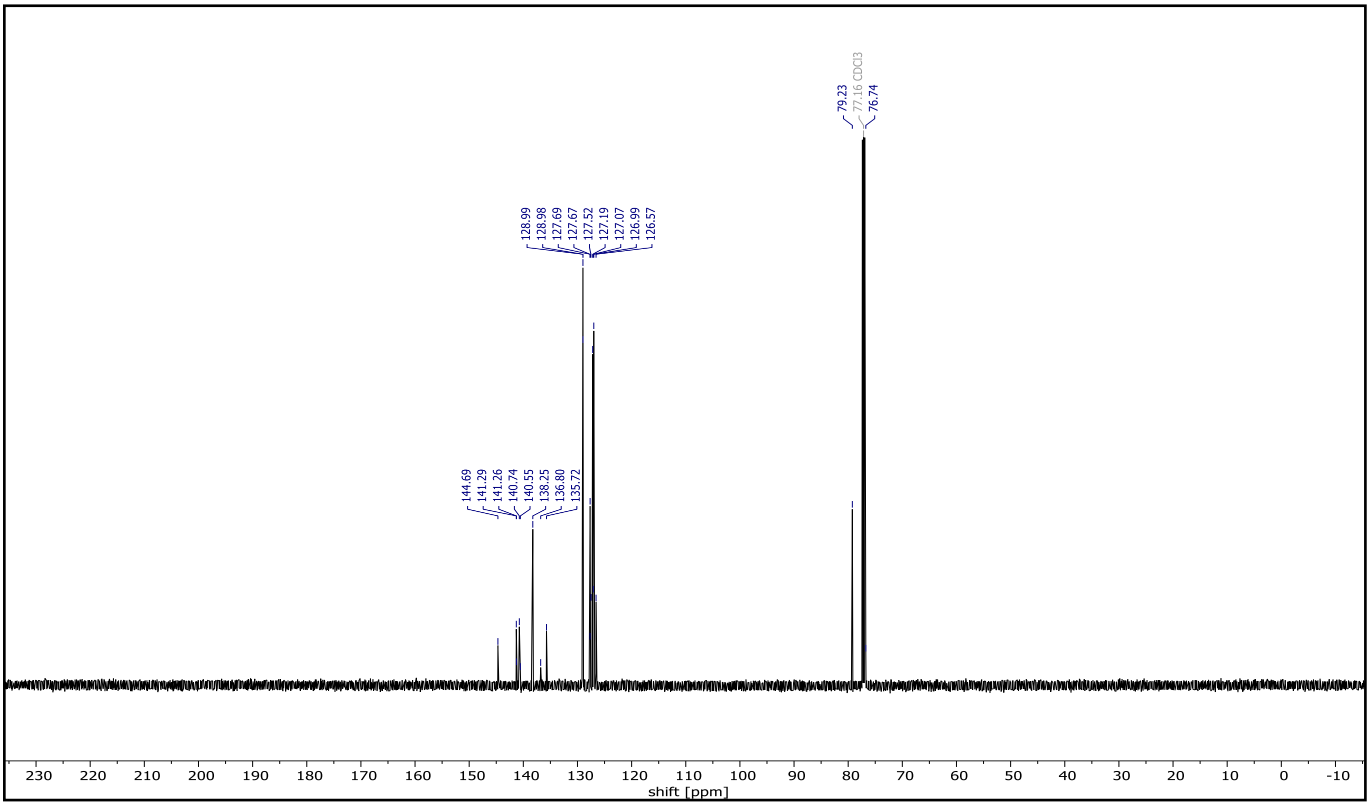

(*E*,*Z*)-2-Bromo-5-(2-bromovinyl)thiophene (*E*,*Z*)-22

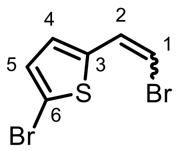

^{1}H-NMR (400 MHz, $CDCl_3$)

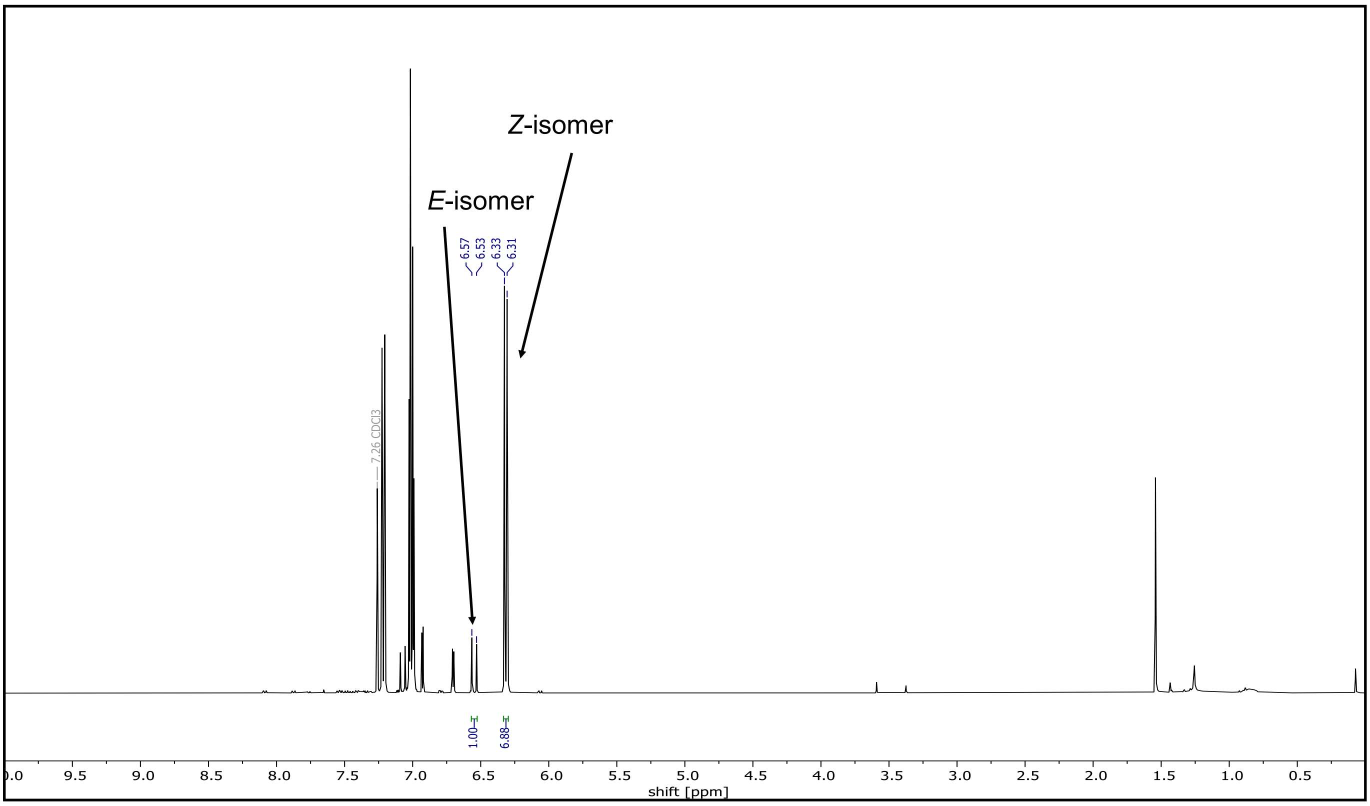

^{1}H-NMR (400 MHz, $CDCl_3$)

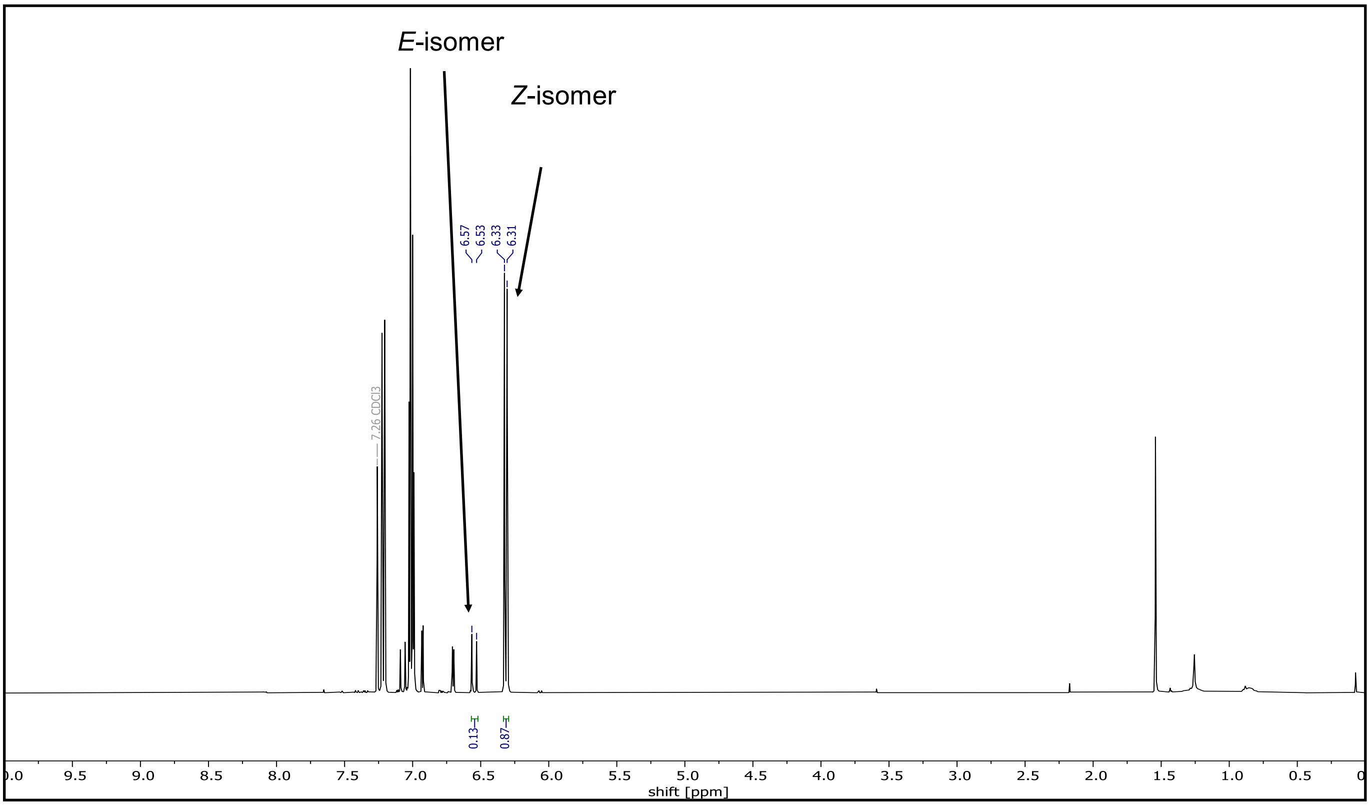

(*E*,*Z*)-2-bromo-5-(2-bromovinyl)thiophene (*E*,*Z*)-22

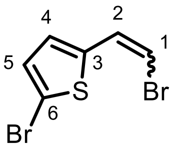

^{1}H-NMR ($CDCl_3$), experiment 1

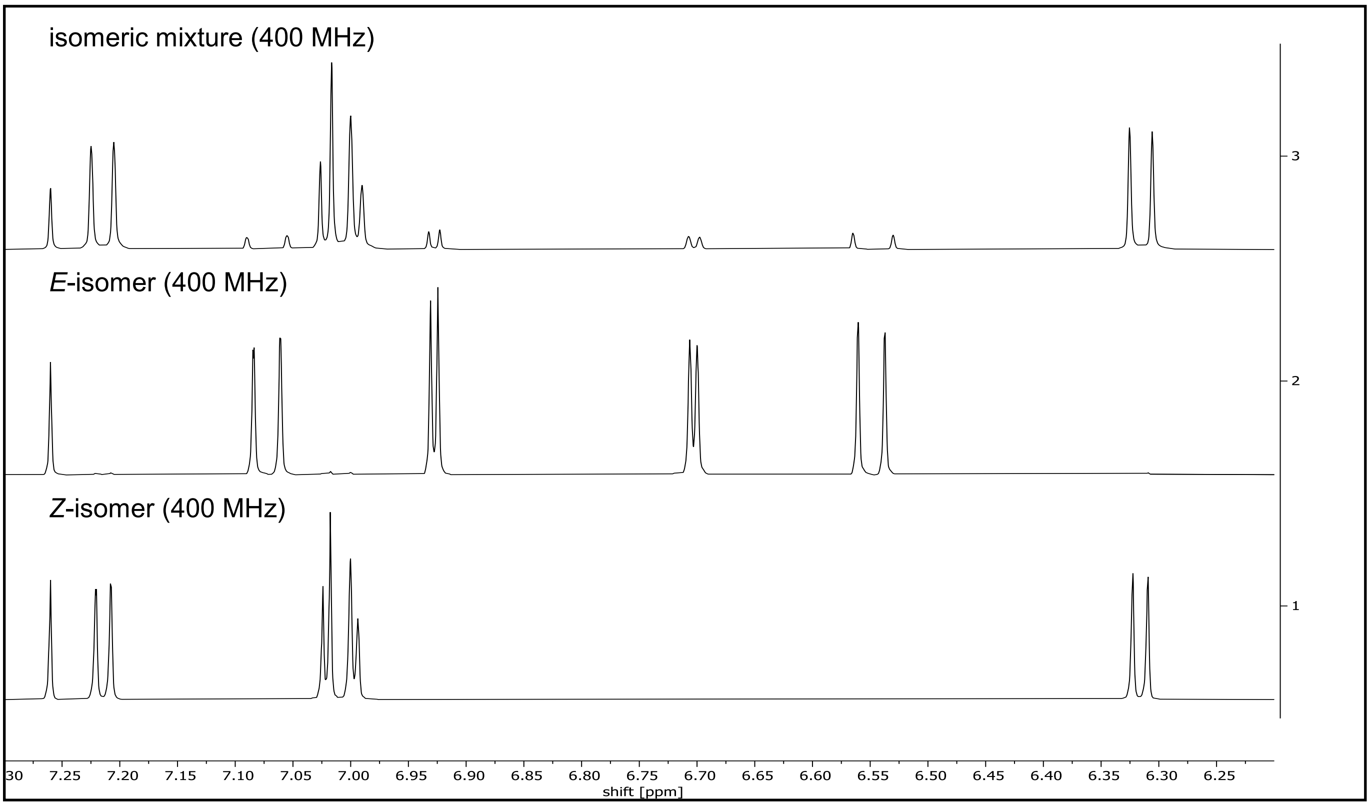

^{1}H-NMR ($CDCl_3$), experiment 2

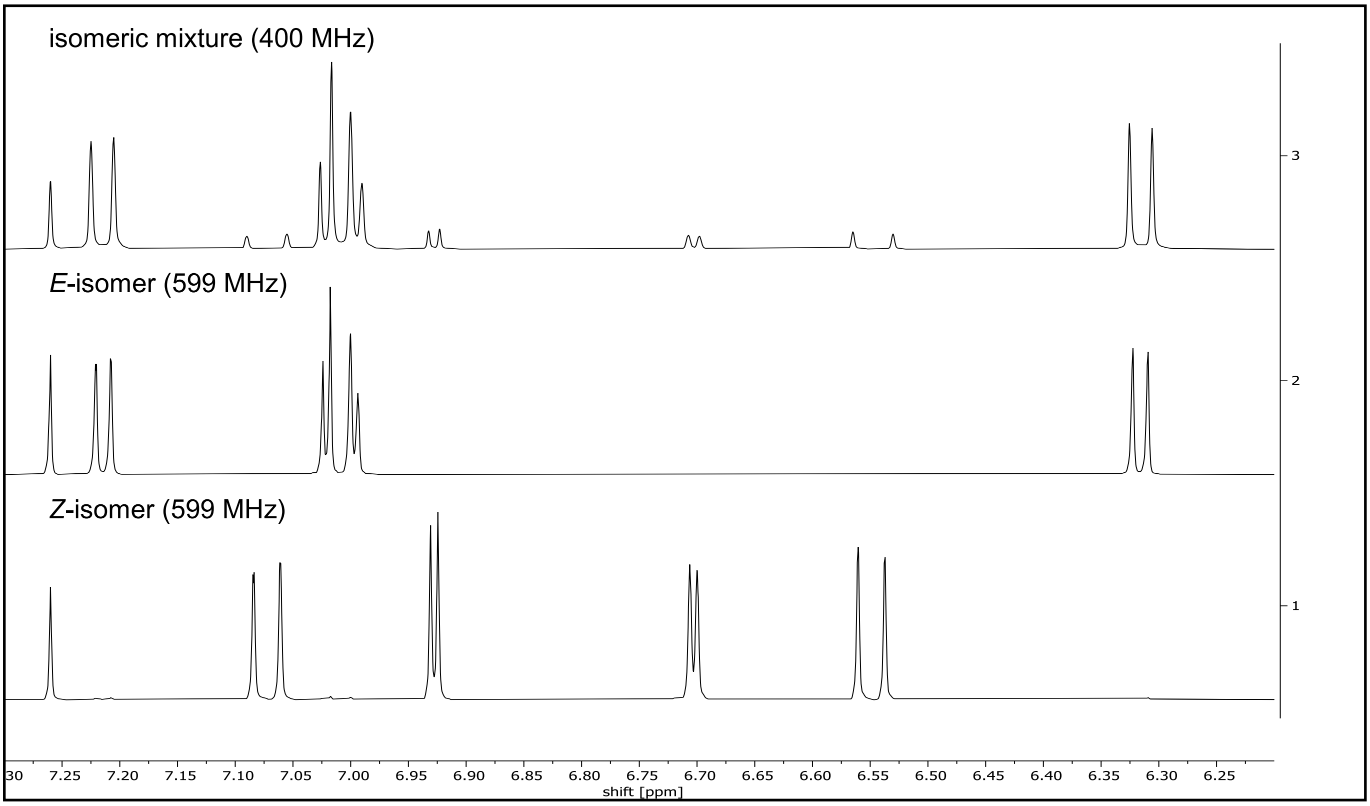

(*E*)-2-bromo-5-(2-bromovinyl)thiophene (*E*)-22

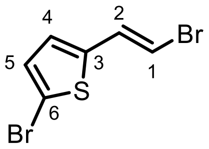

^{1}H-NMR (599 MHz, $CDCl_3$)

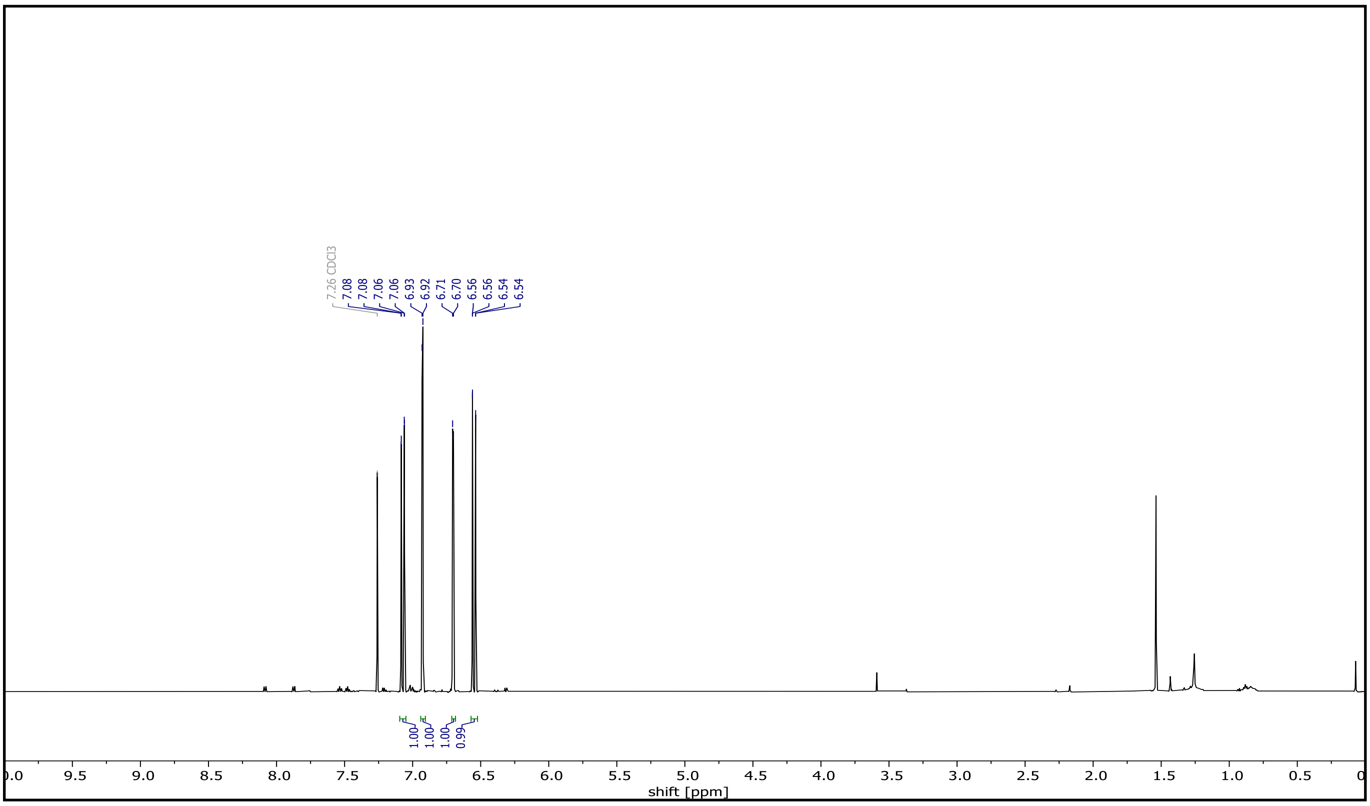

^{13}C-NMR (151 MHz, $CDCl_3$)

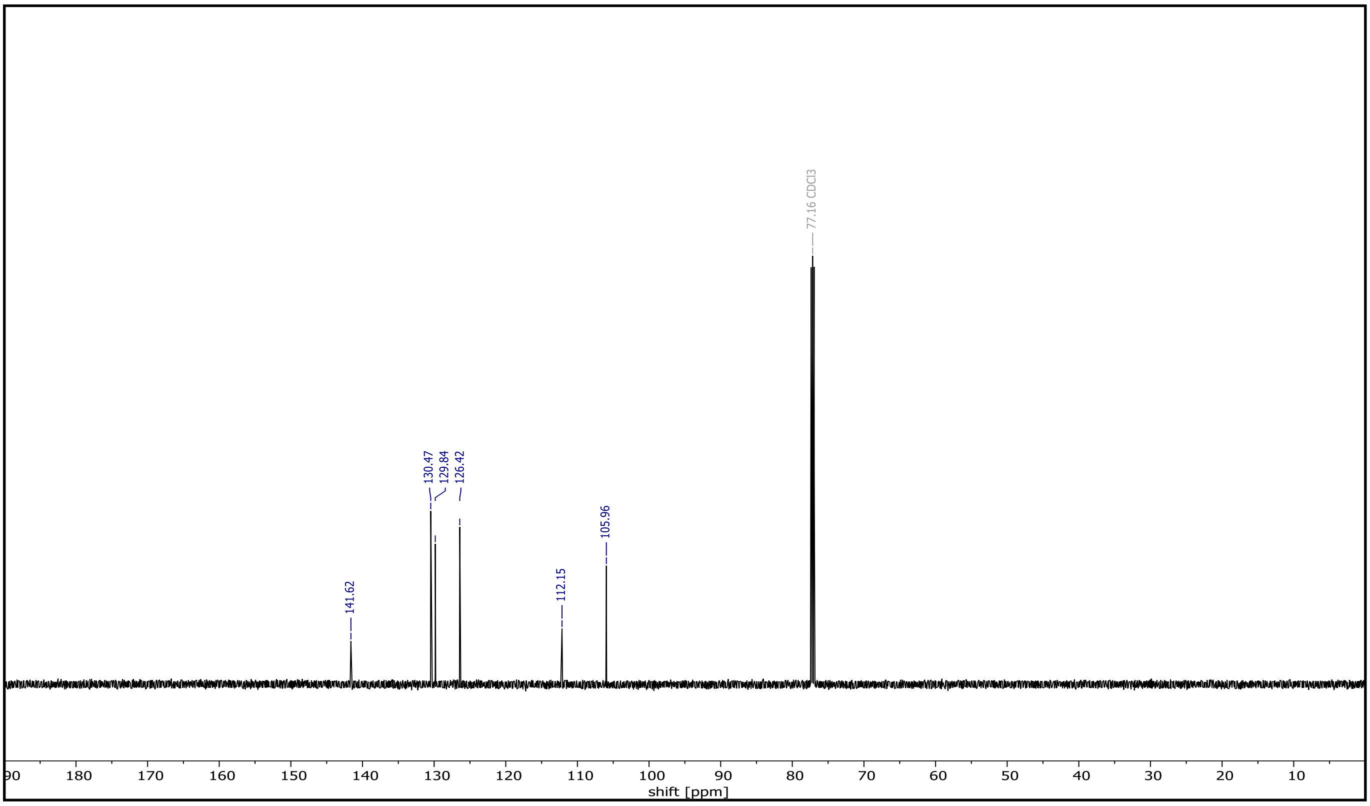

(*Z*)-2-bromo-5-(2-bromovinyl)thiophene (*Z*)-22

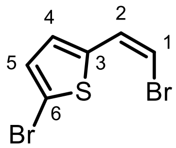

^{1}H-NMR (599 MHz, $CDCl_3$)

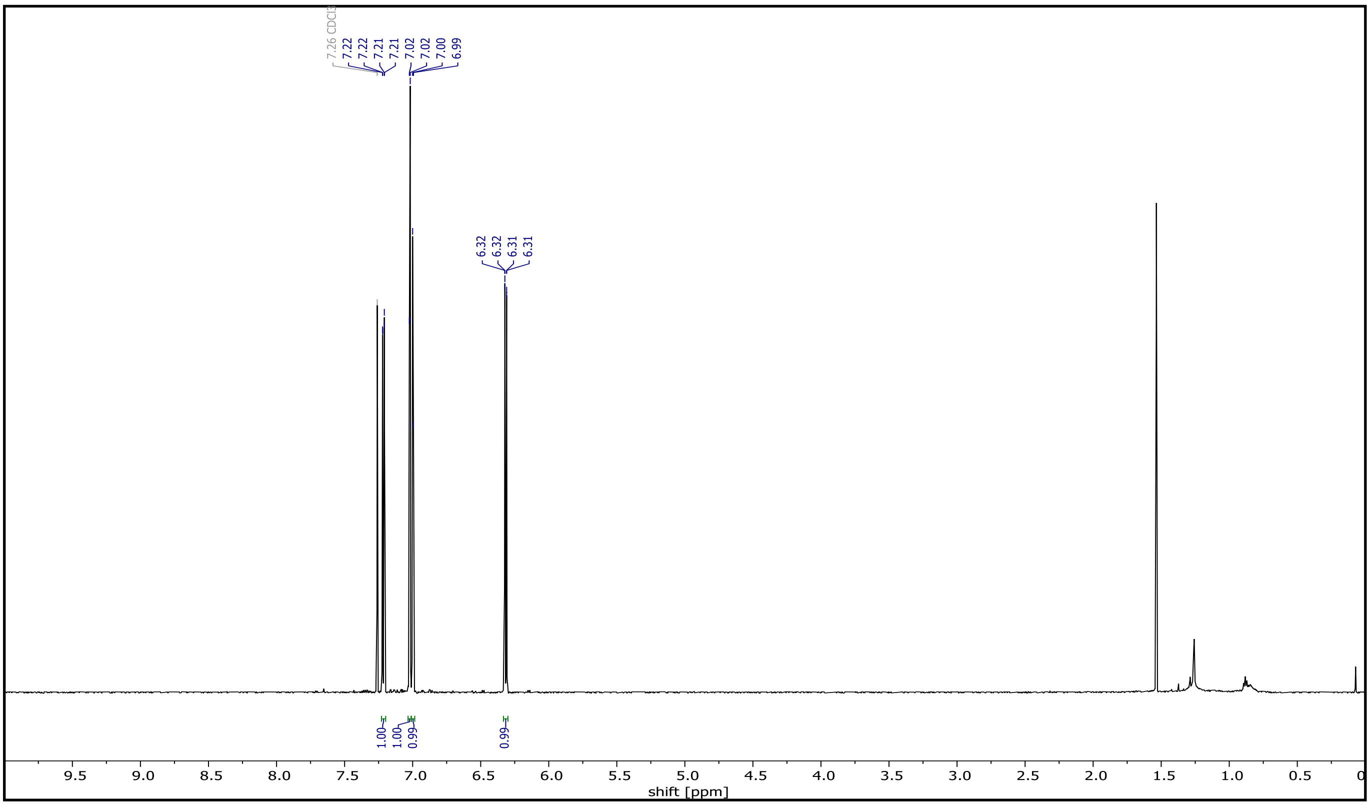

^{13}C-NMR (151 MHz, $CDCl_3$)

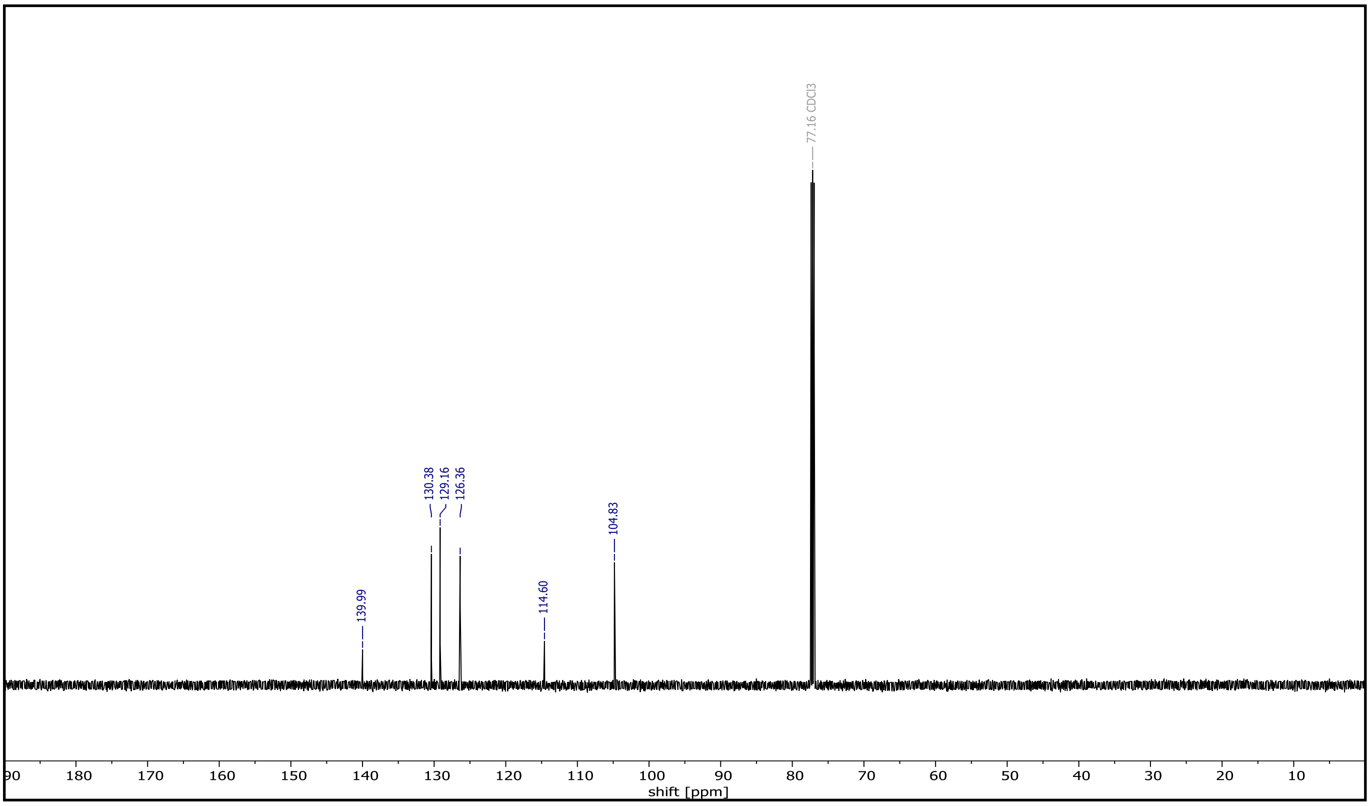

(*E*,*Z*)-2-(2-Bromovinyl)thiophene (*E*,*Z*)-23

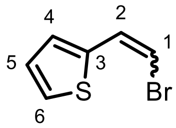

^1H NMR (400 MHz, $CDCl_3$), experiment 1

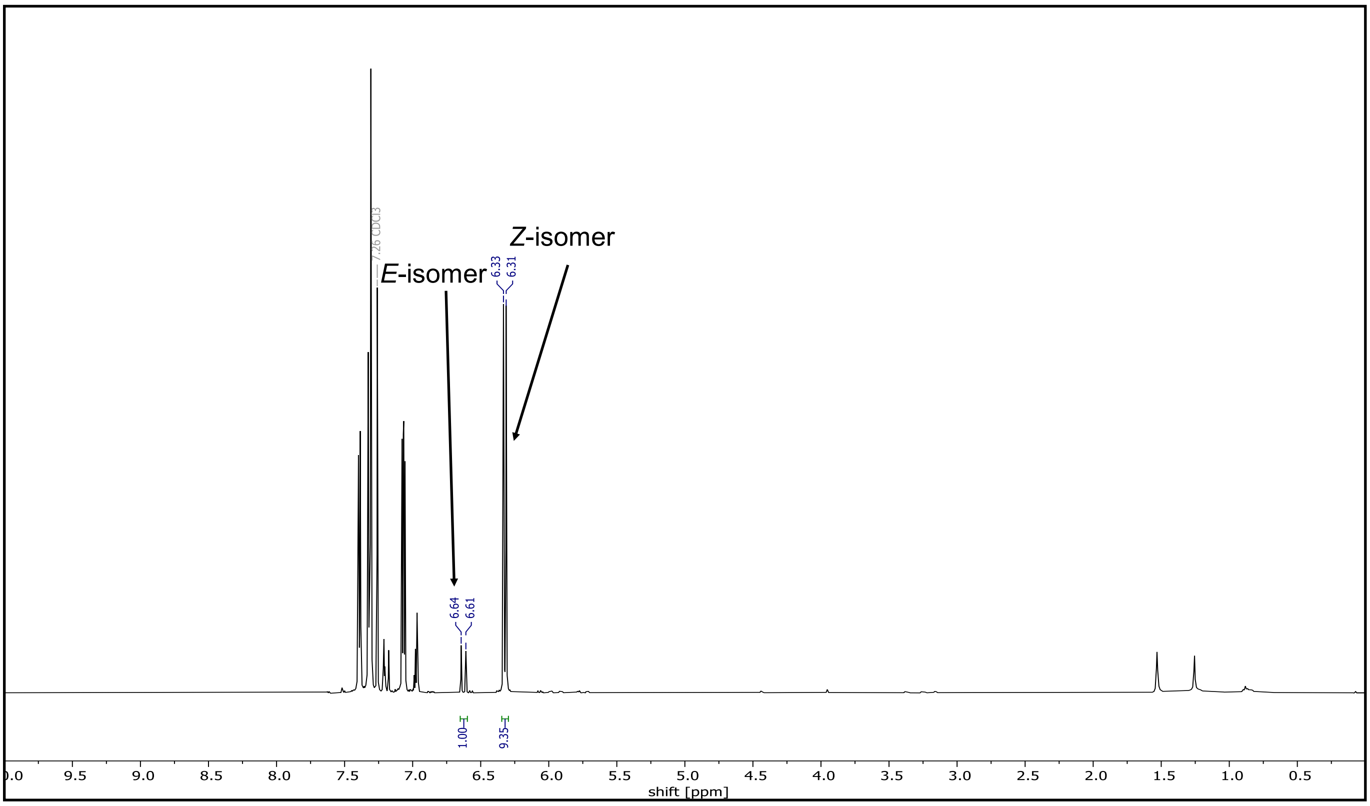

^1H NMR (400 MHz, $CDCl_3$), experiment 2

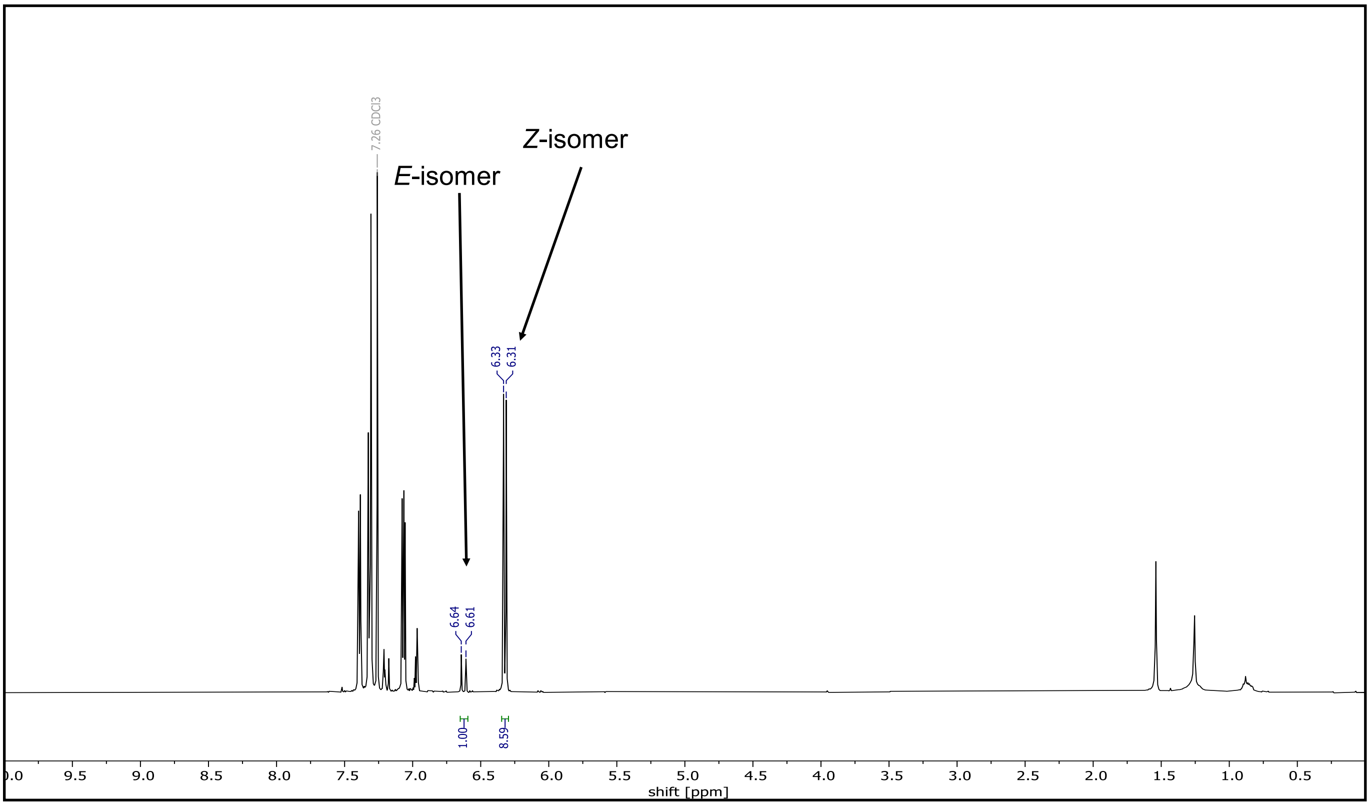

(*E*,*Z*)-2-(2-Bromovinyl)thiophene (*E*,*Z*)-23

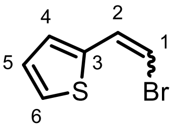

^{1}H NMR ($CDCl_3$), experiment 1

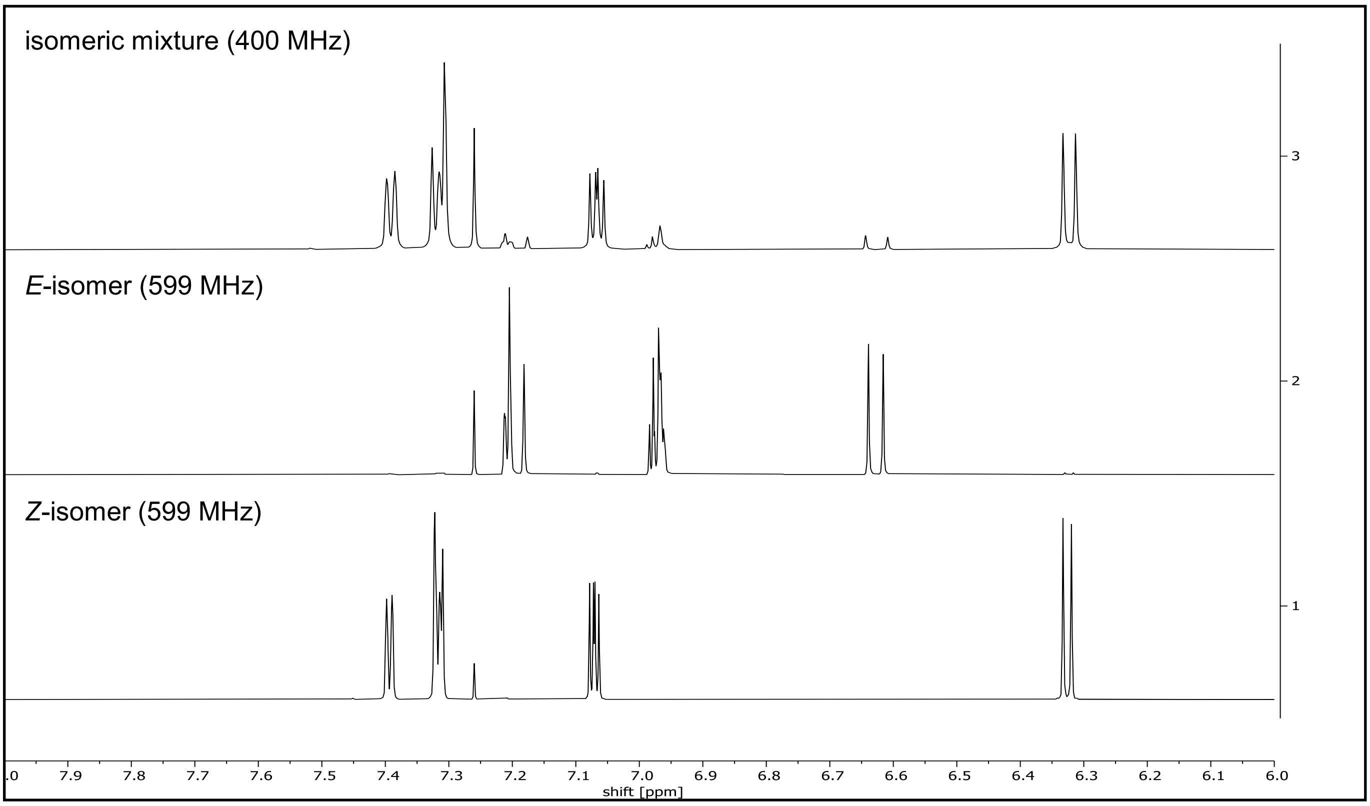

^{1}H NMR ($CDCl_3$), experiment 2

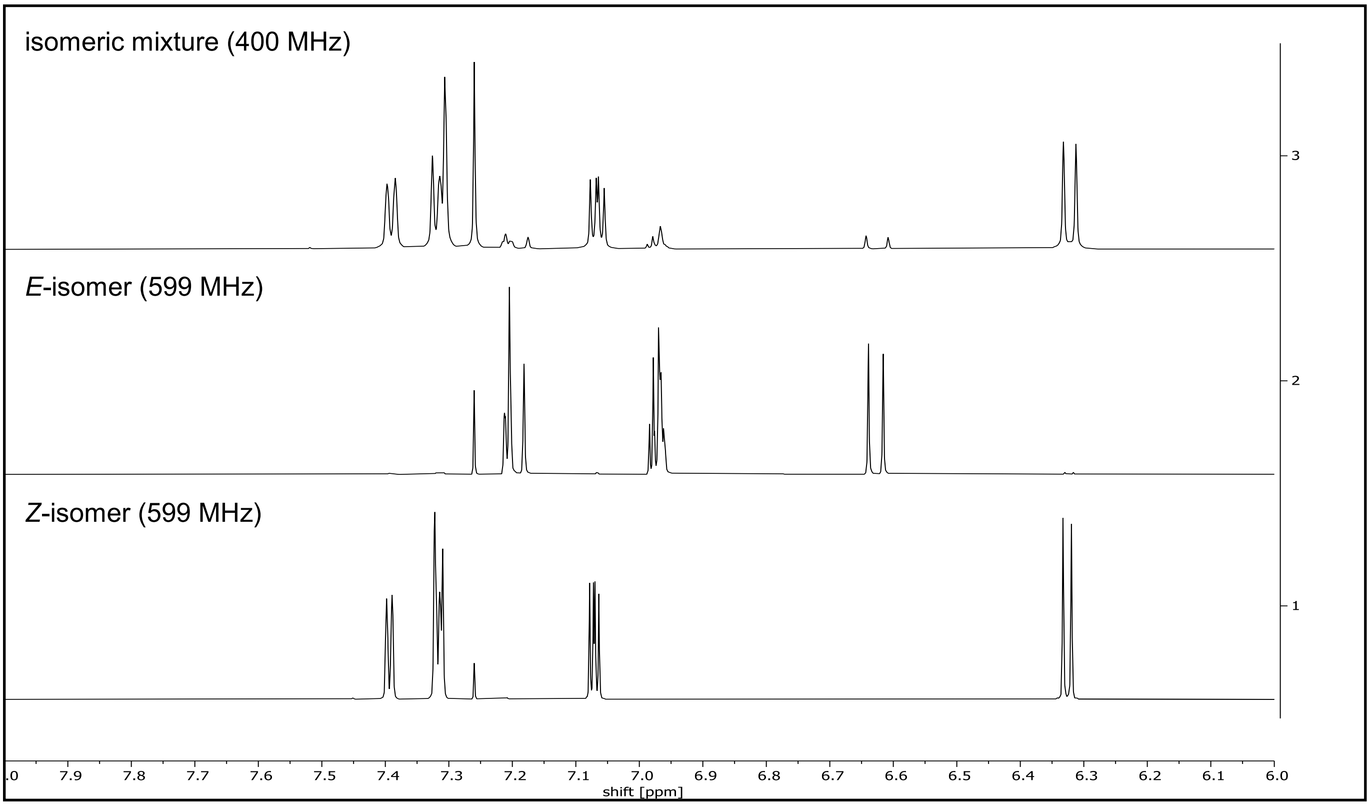

(*E*)-2-(2-Bromovinyl)thiophene (*E*)-23

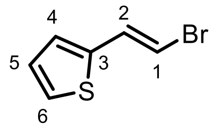

^{1}H NMR (599 MHz, $CDCl_3$)

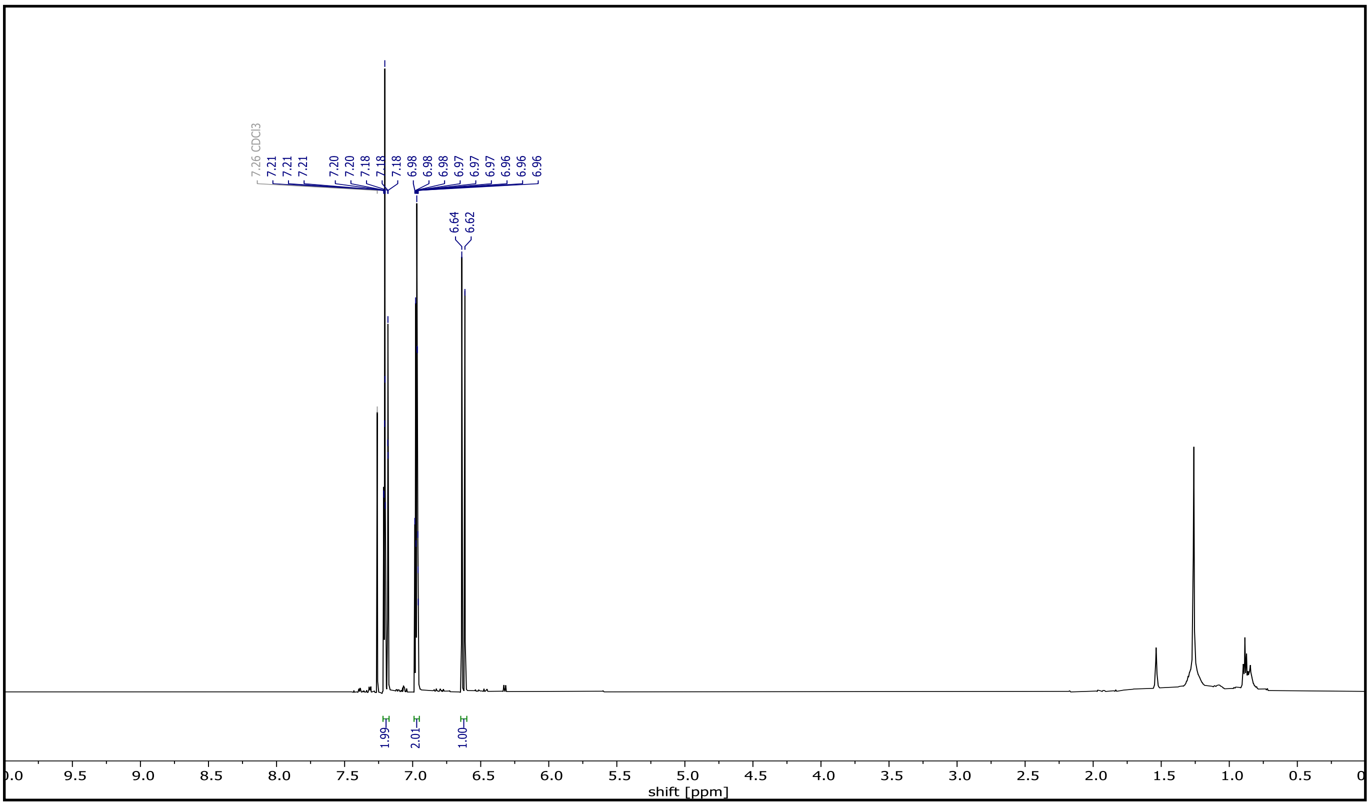

$^{13}C\{^1H\}$ NMR (151 MHz, $CDCl_3$)

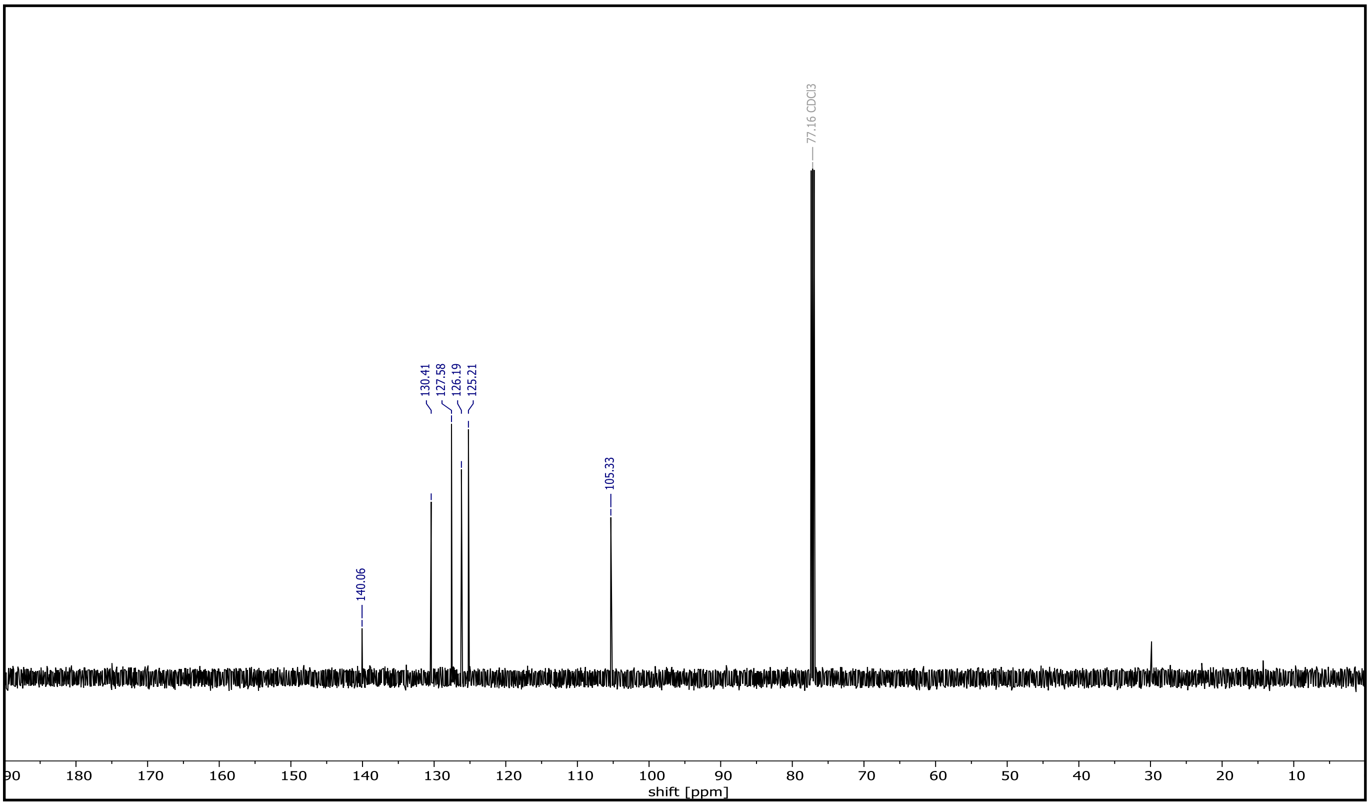

(*Z*)-2-(2-Bromovinyl)thiophene (*Z*)-23

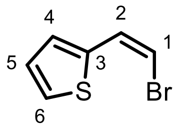

^1H NMR (599 MHz, $CDCl_3$)

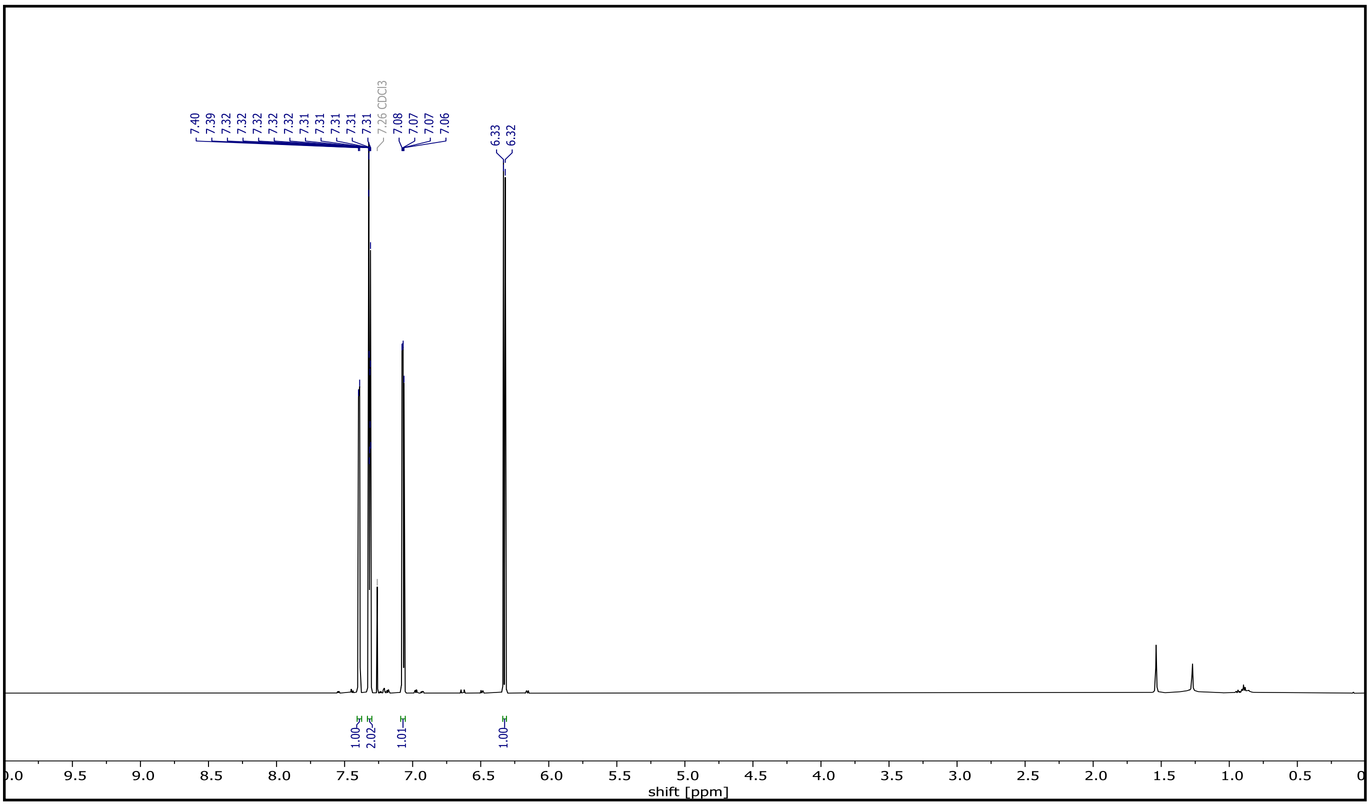

$^{13}C\{^1H\}$ NMR (151 MHz, $CDCl_3$)

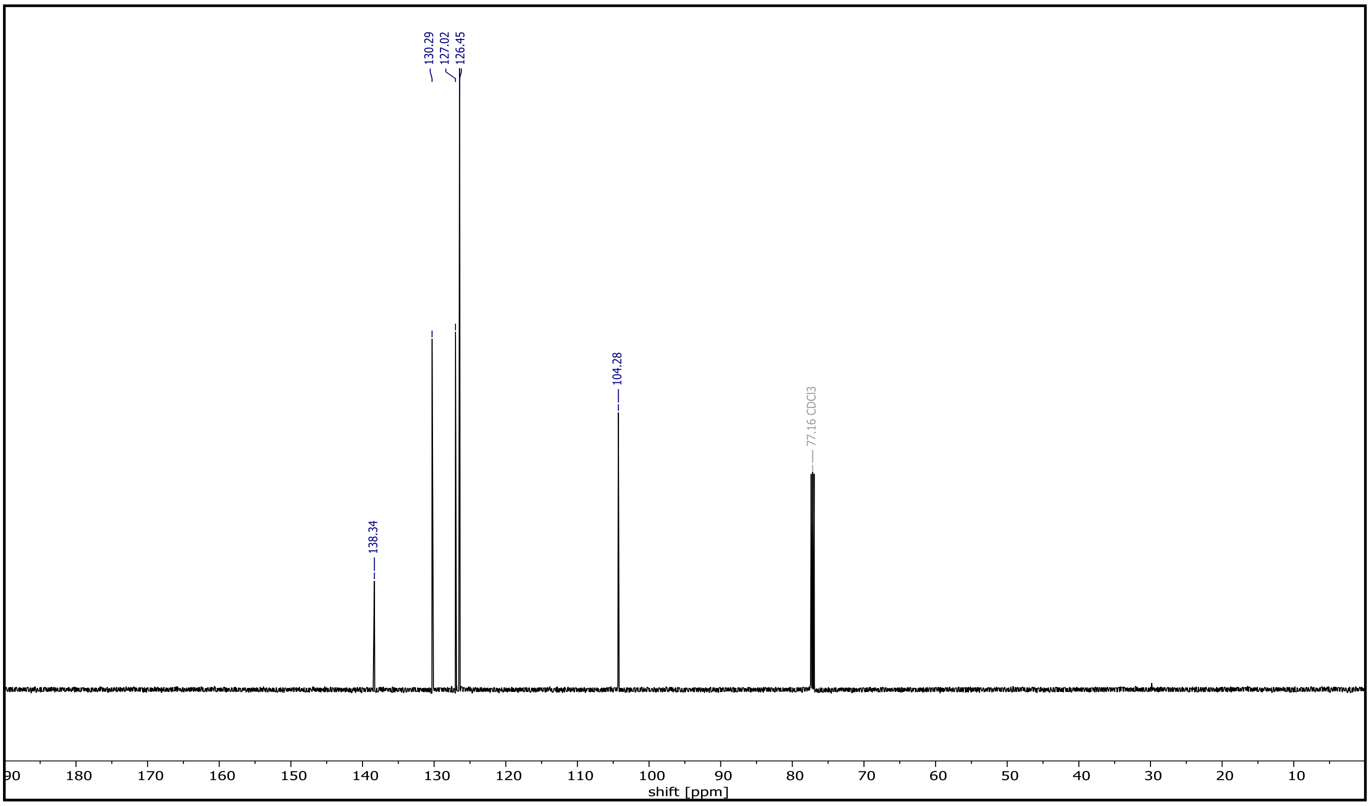

(*E*,*Z*)-3-(2-Bromovinyl)thiophene (*E*,*Z*)-24 (*Z*:*E* = 78:22)

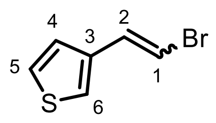

^{1}H-NMR (400 MHz, $CDCl_3$), experiment 1

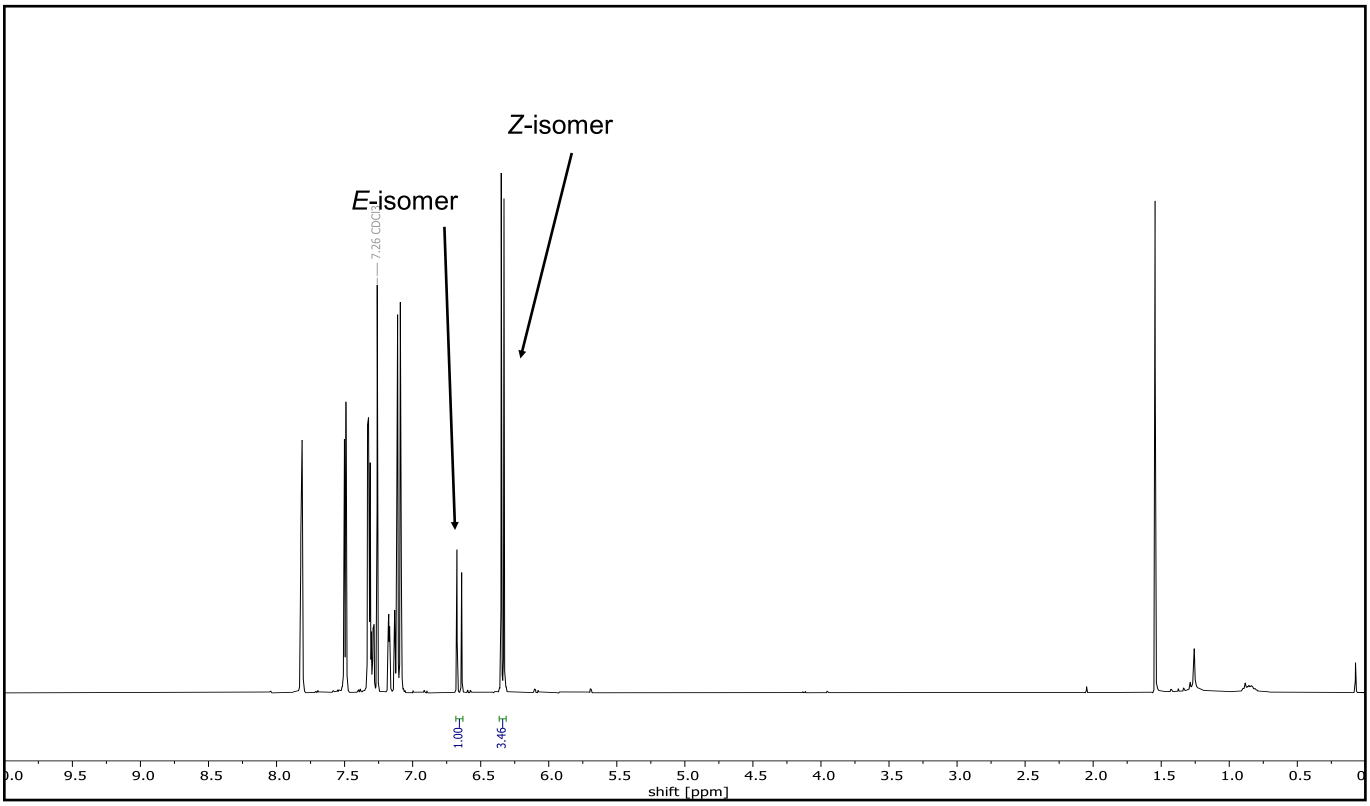

^{1}H-NMR (400 MHz, $CDCl_3$), experiment 2

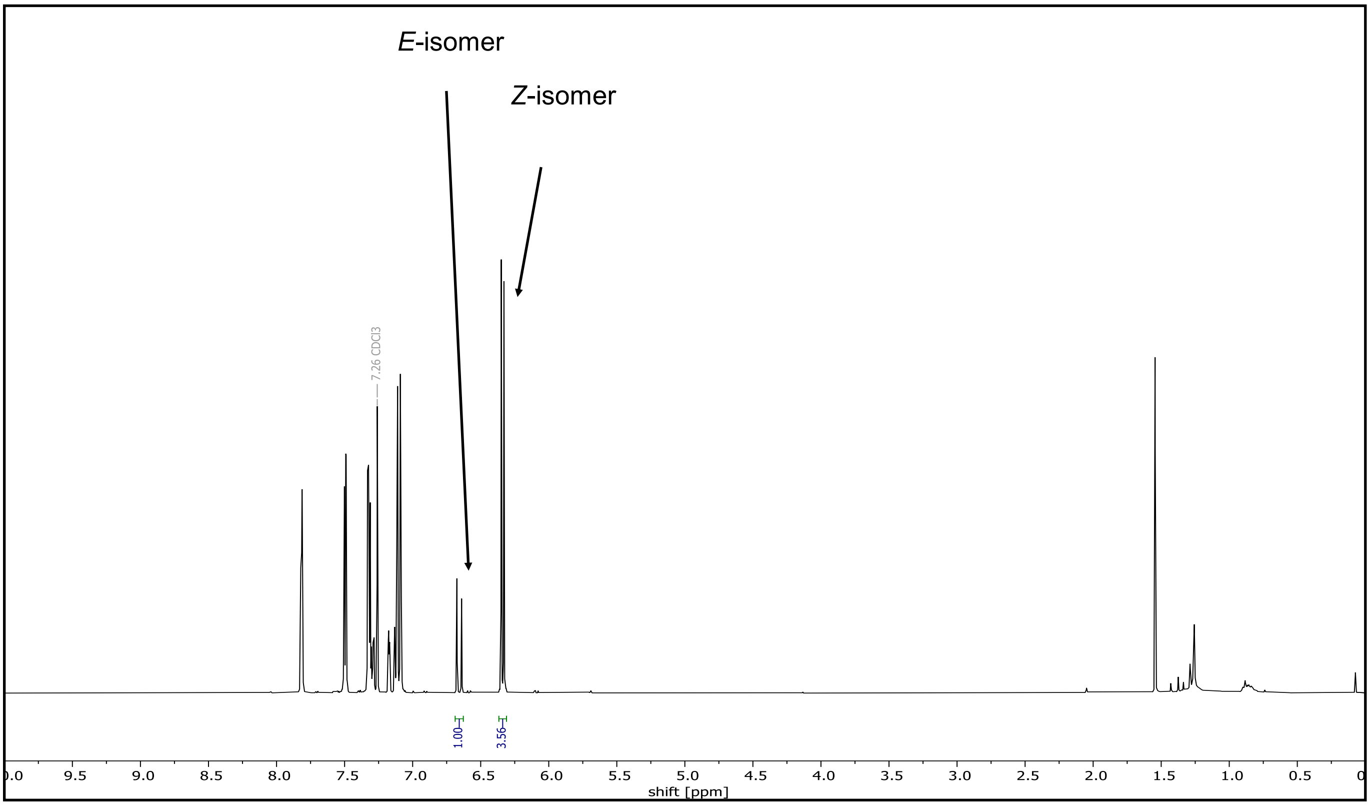

(*E*,*Z*)-3-(2-Bromovinyl)thiophene (*E*,*Z*)-24 (*Z*:*E* = 78:22)

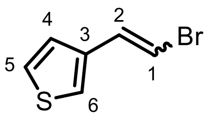

^{1}H-NMR (500 MHz, $CDCl_3$)

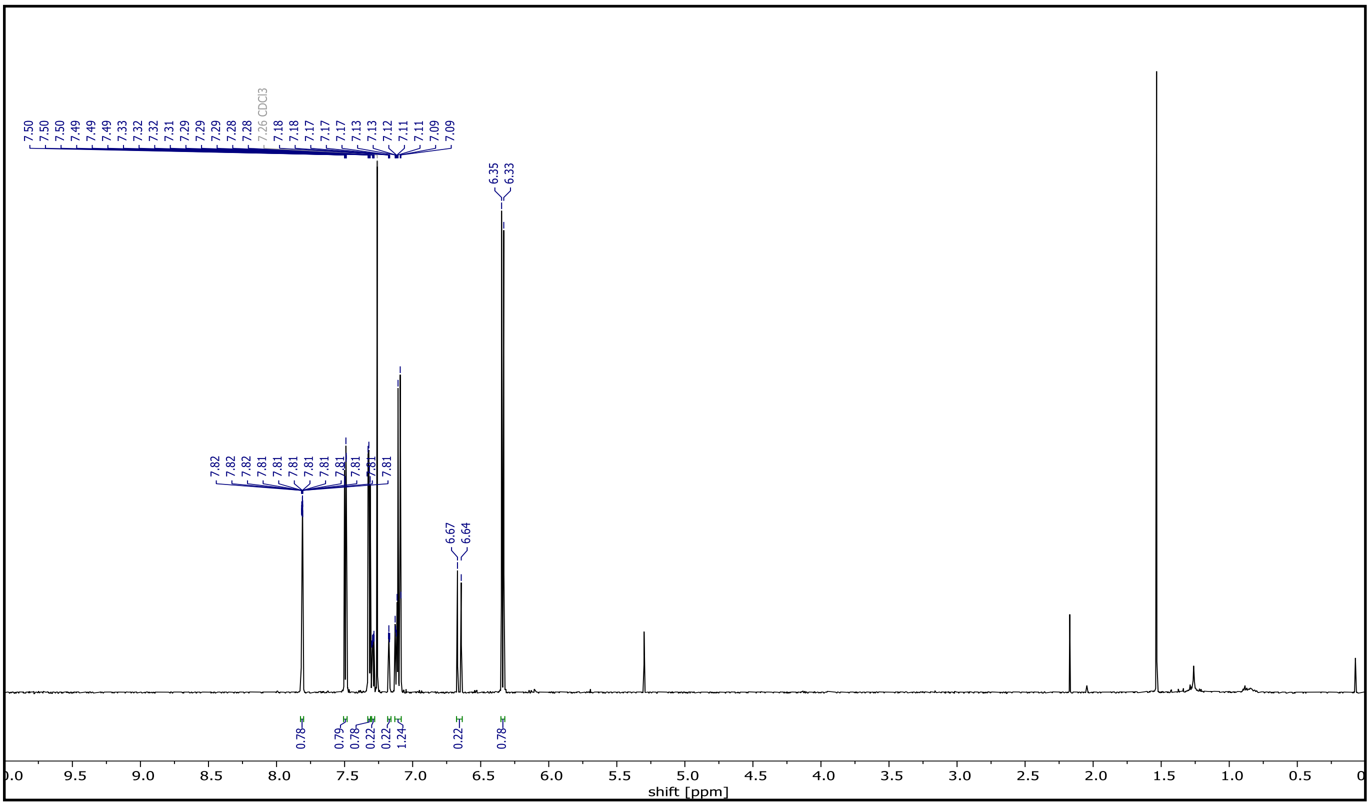

^{13}C-NMR (126 MHz, $CDCl_3$)

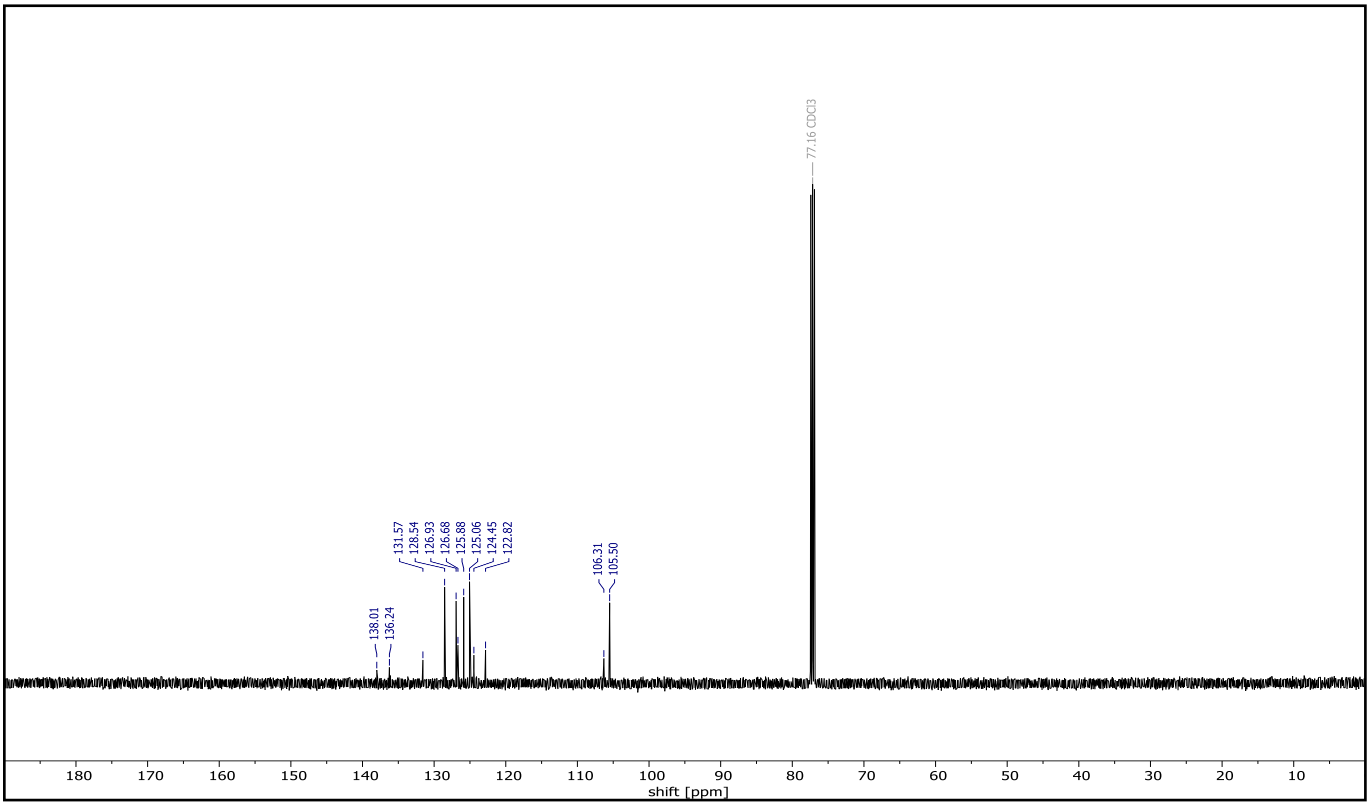

(*E*,*Z*)-(4-Bromostyryl)dicyclopentadienyl zirconium chloride (*E*,*Z*)-Zr1

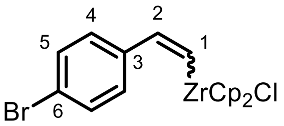

^{1}H-NMR (599 MHz, CD_2Cl_3)

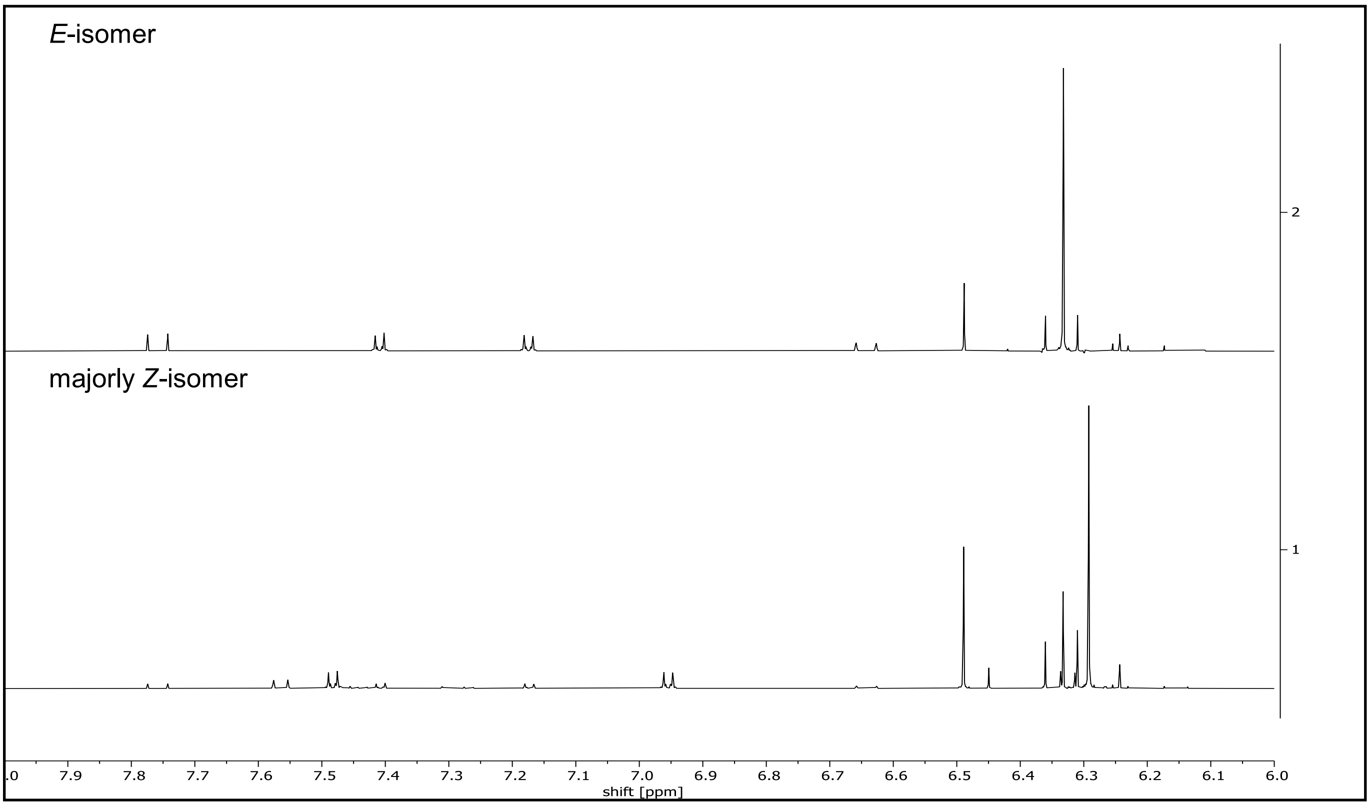

^{13}C-NMR (151 MHz, CD_2Cl_2),

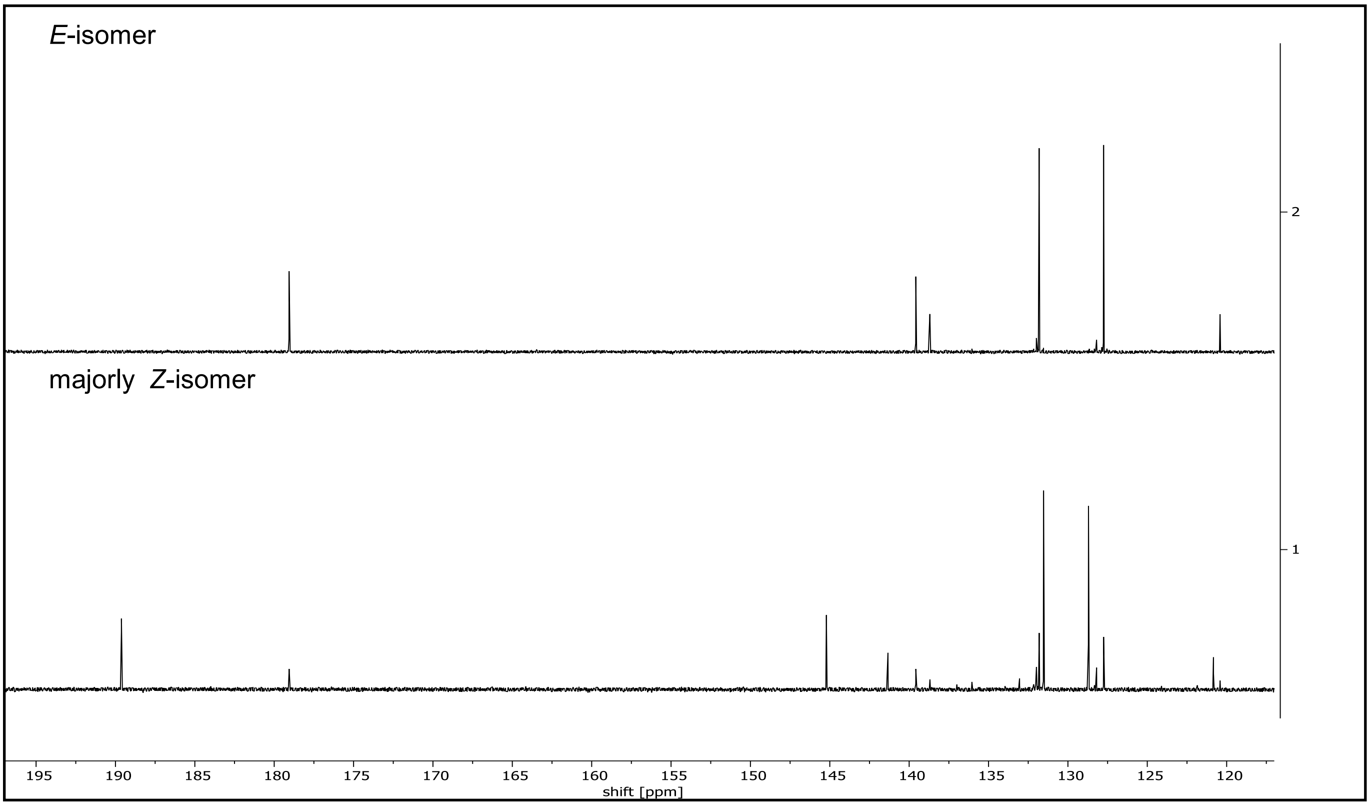

(*E*)-(4-Bromostyryl)dicyclopentadienyl zirconium chloride (*E*)-Zr1

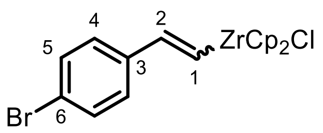

^{1}H-NMR (599 MHz, $CDCl_3$)

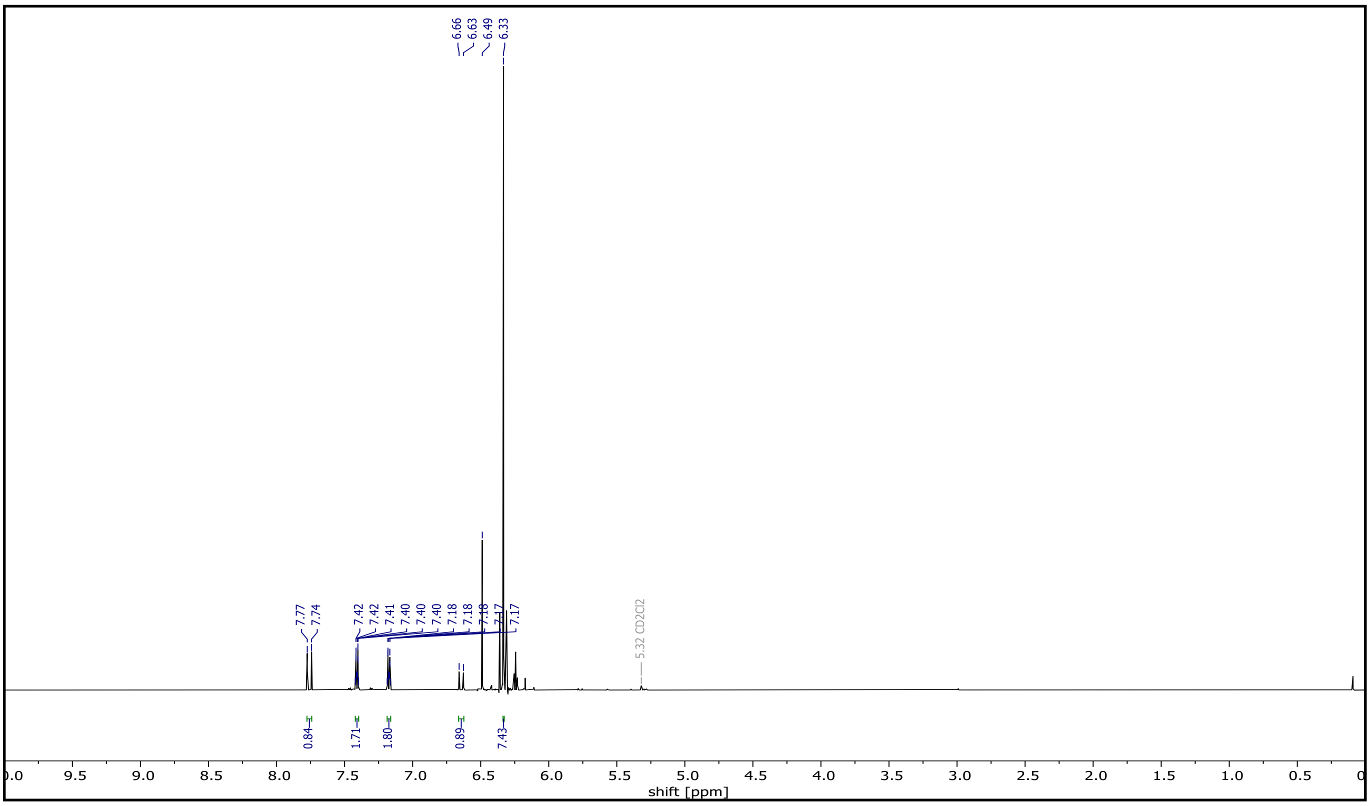

^{13}C-NMR (151 MHz, $CDCl_3$)

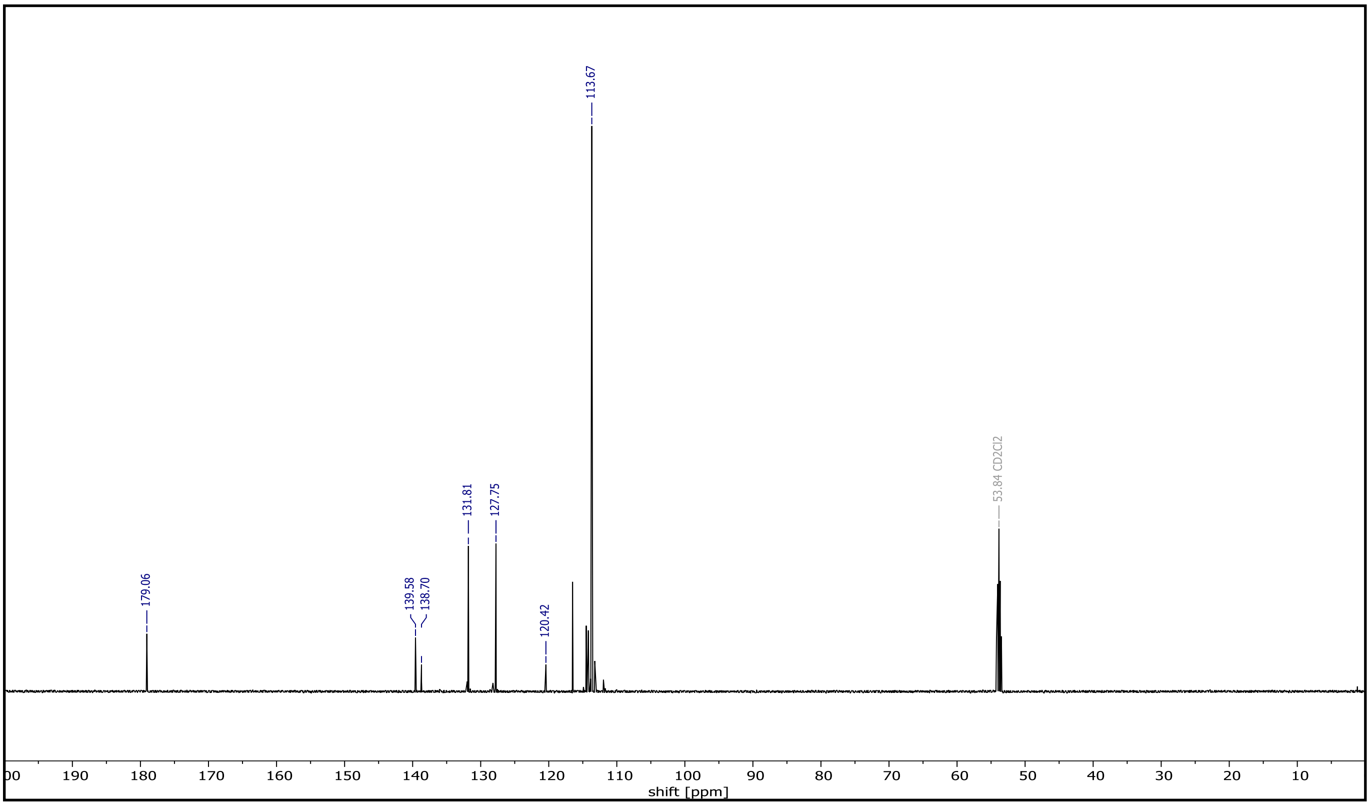

(*Z*)-(4-Bromostyryl)dicyclopentadienyl zirconium chloride (*Z*)-Zr1

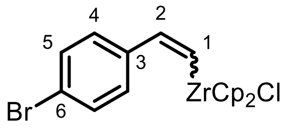

^{1}H-NMR (599 MHz, $CDCl_3$)

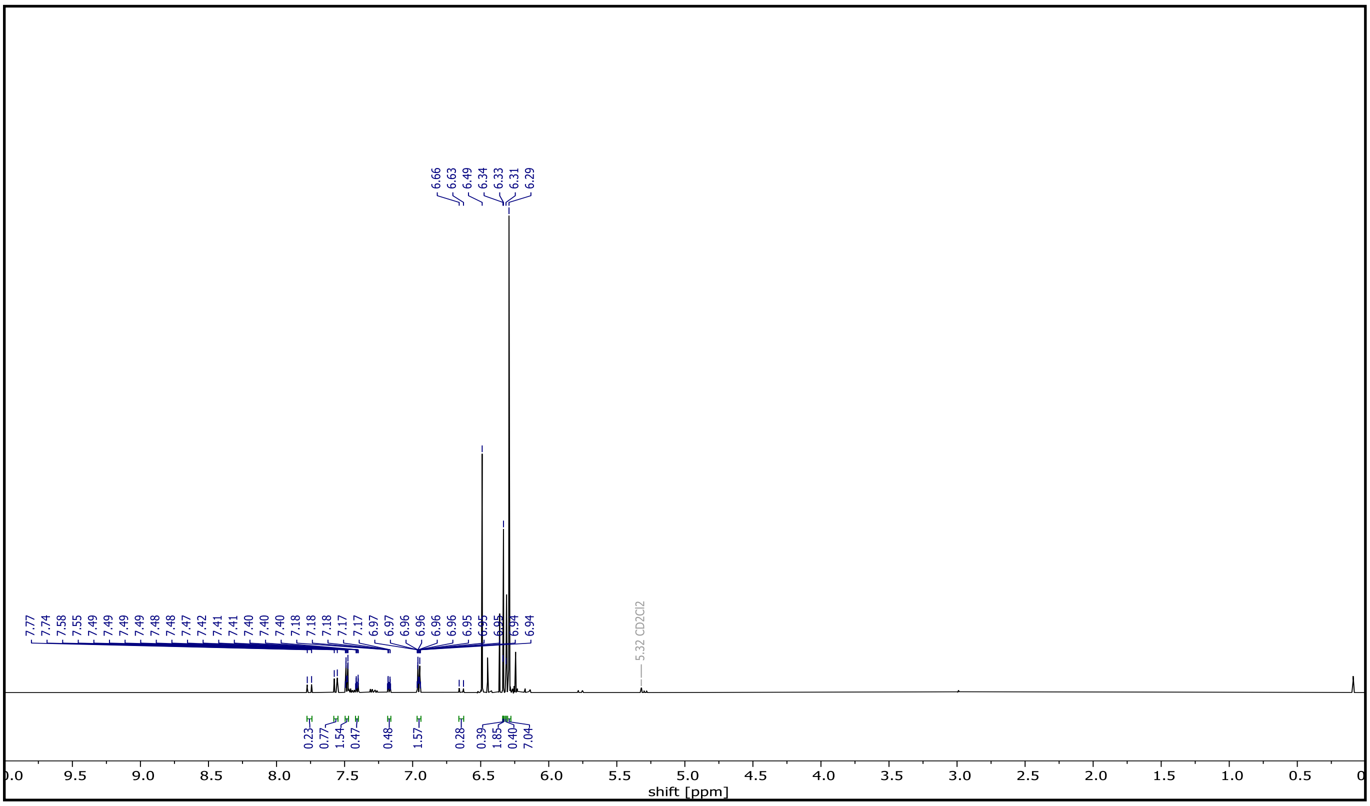

^{13}C-NMR (151 MHz, $CDCl_3$)

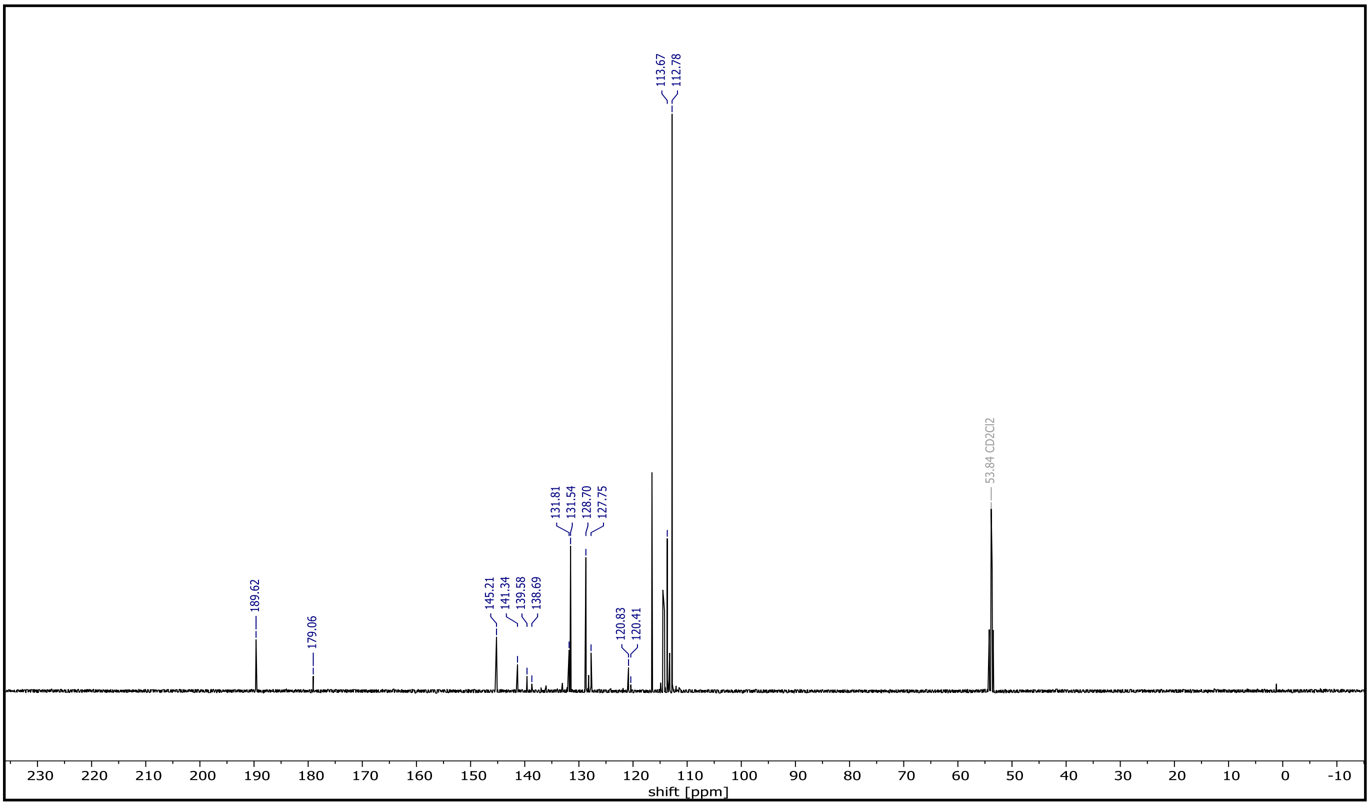

(1*E*,3*E*)-1,4-Bis(4-bromophenyl)buta-1,3-diene 25

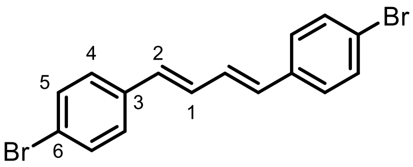

^{1}H-NMR (599 MHz, $CDCl_3$)

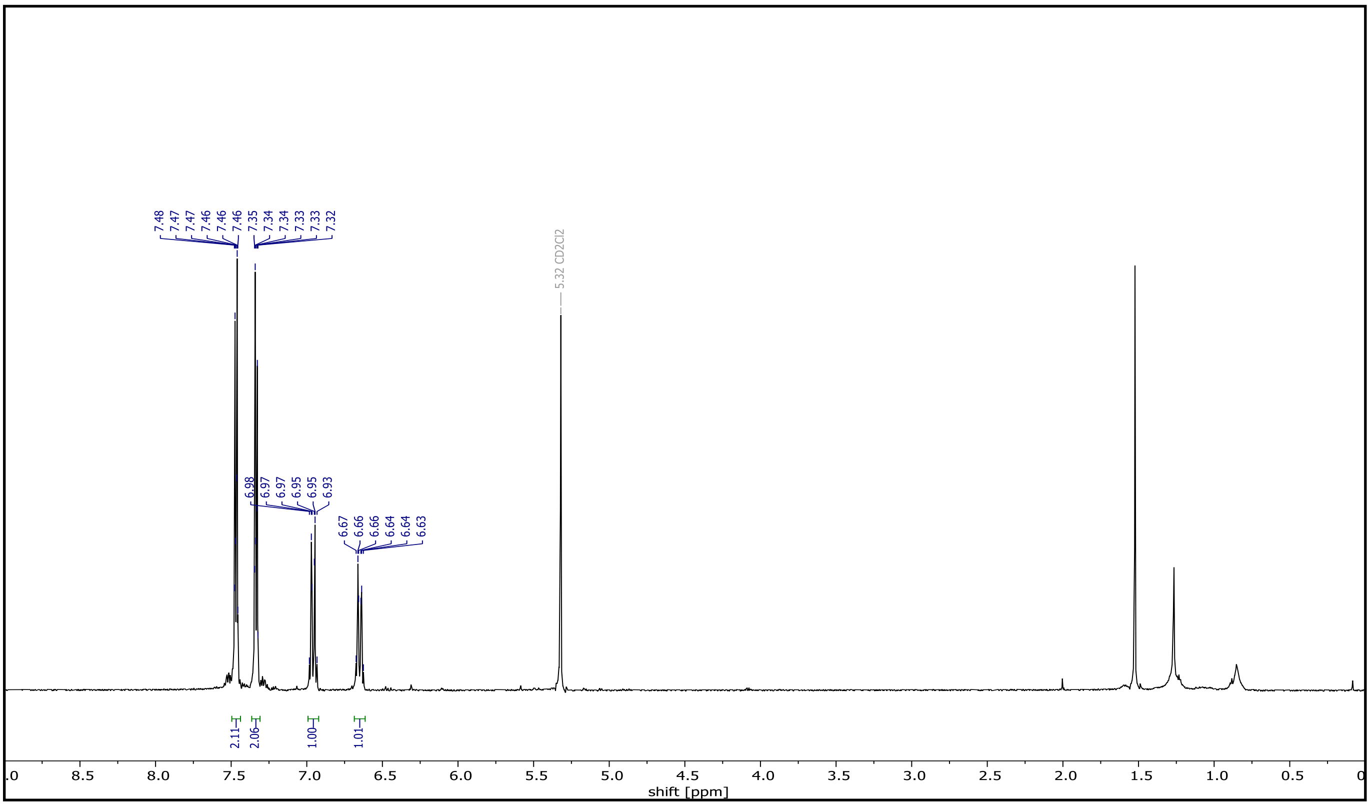

^{13}C-NMR (151 MHz, $CDCl_3$)

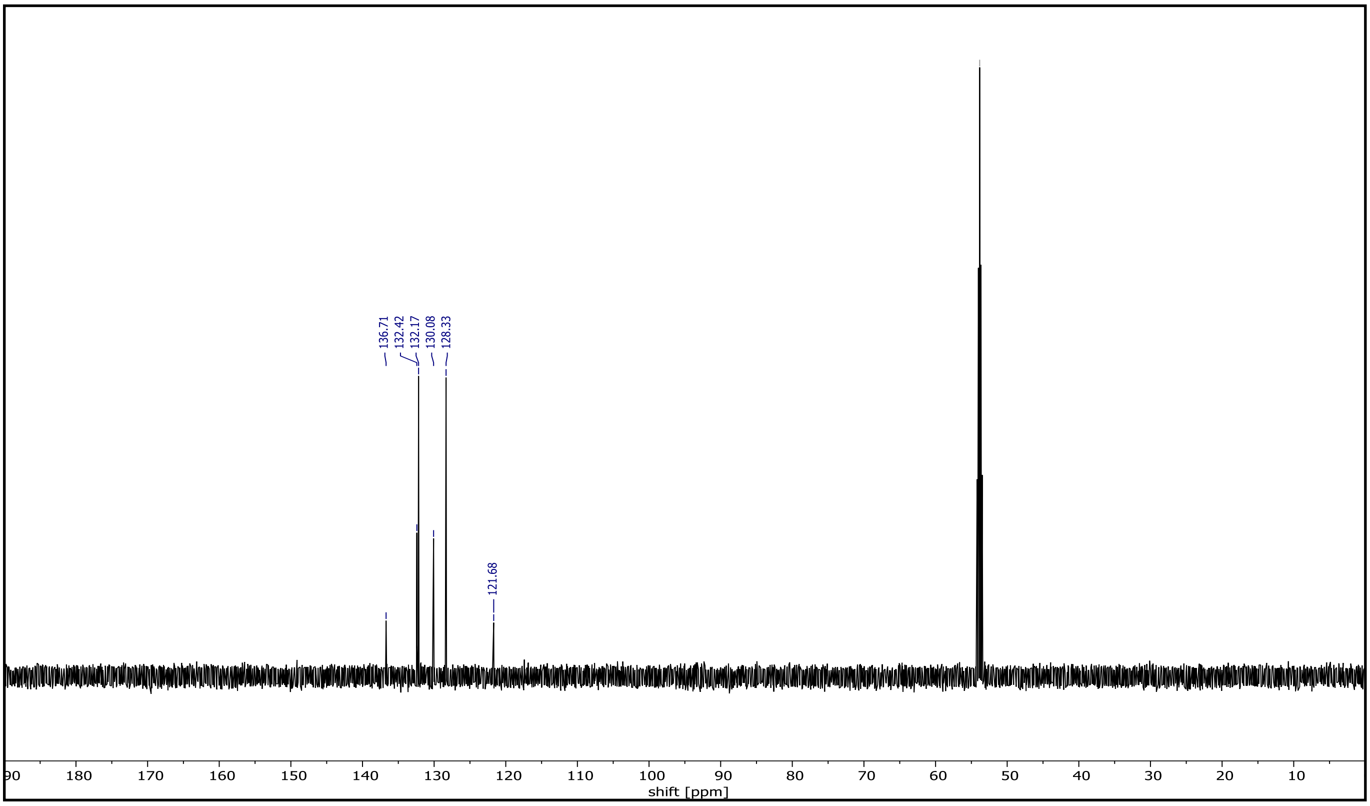

(1*E*,3*E*)-1,4-bis(4-bromophenyl)buta-1,3-diene 25 stacked with (*E*)-(4-Bromostyryl)dicyclopentadienyl zirconium chloride (*E*)-Zr1 and (*Z*,*E*)-(4-Bromostyryl)dicyclopentadienyl zirconium chloride (*Z*,*E*)-Zr1

^{1}H-NMR (599 MHz, $CDCl_3$)

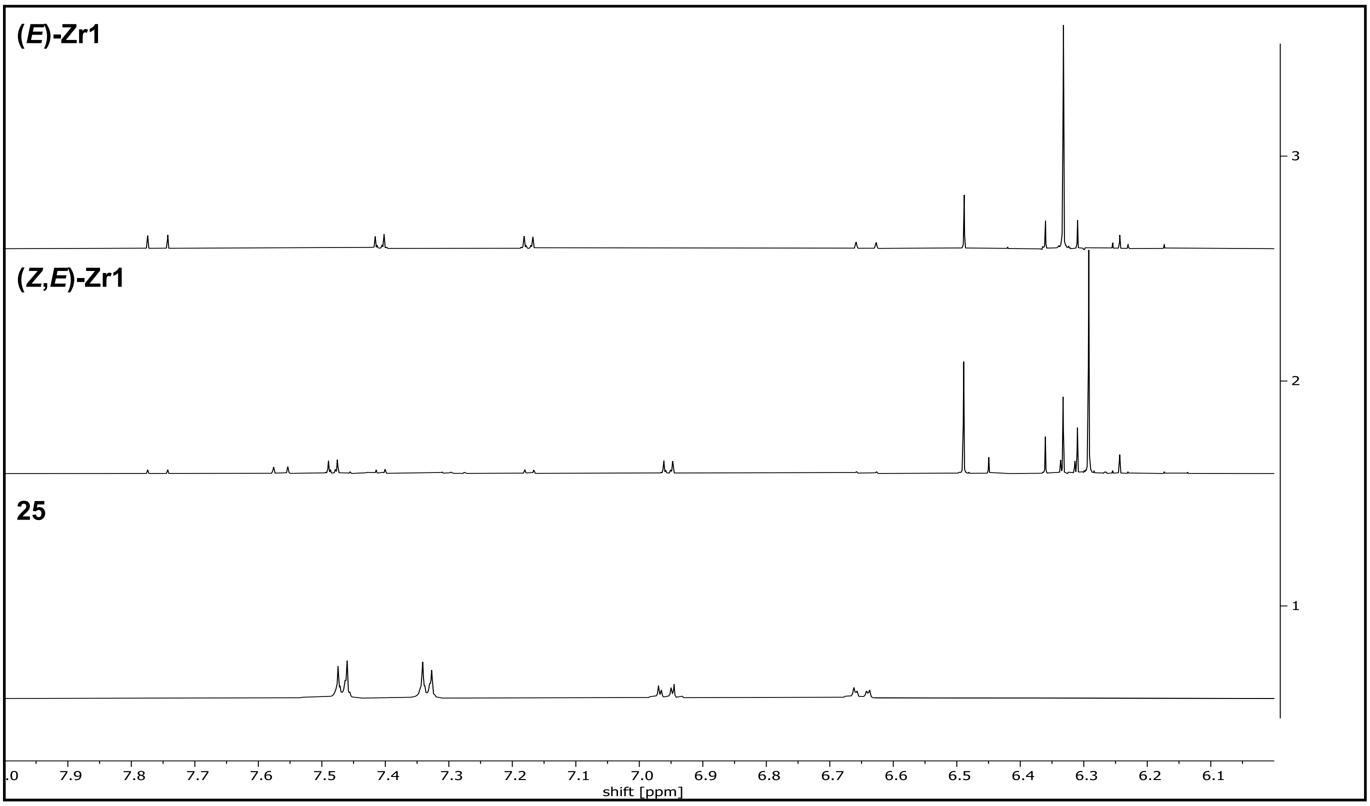

^{13}C-NMR (151 MHz, $CDCl_3$)

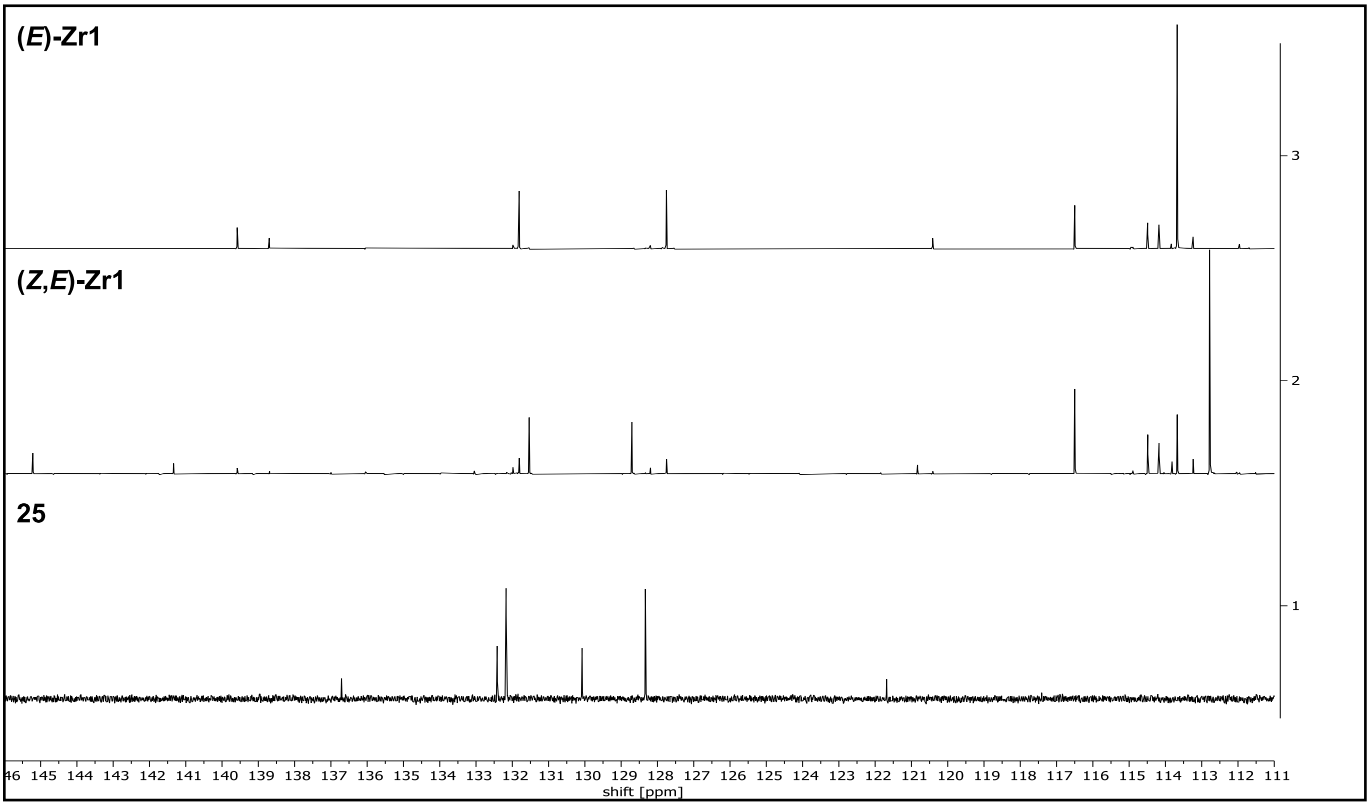

1-Ethynyl-4-iodobenzene A-4

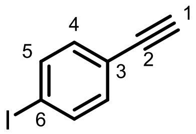

^{1}H-NMR (500 MHz,$CDCl_3$)

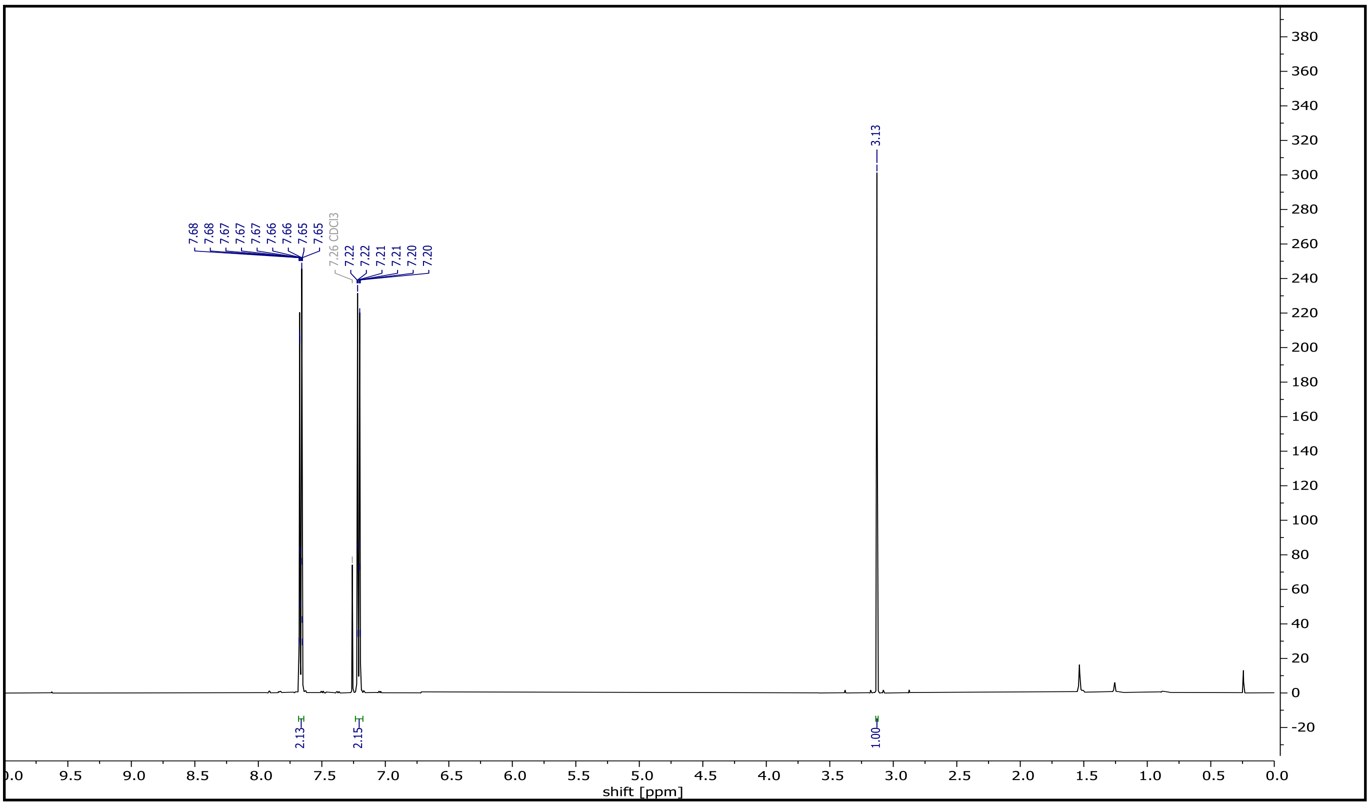

^{13}C-NMR (126 MHz,$CDCl_3$)

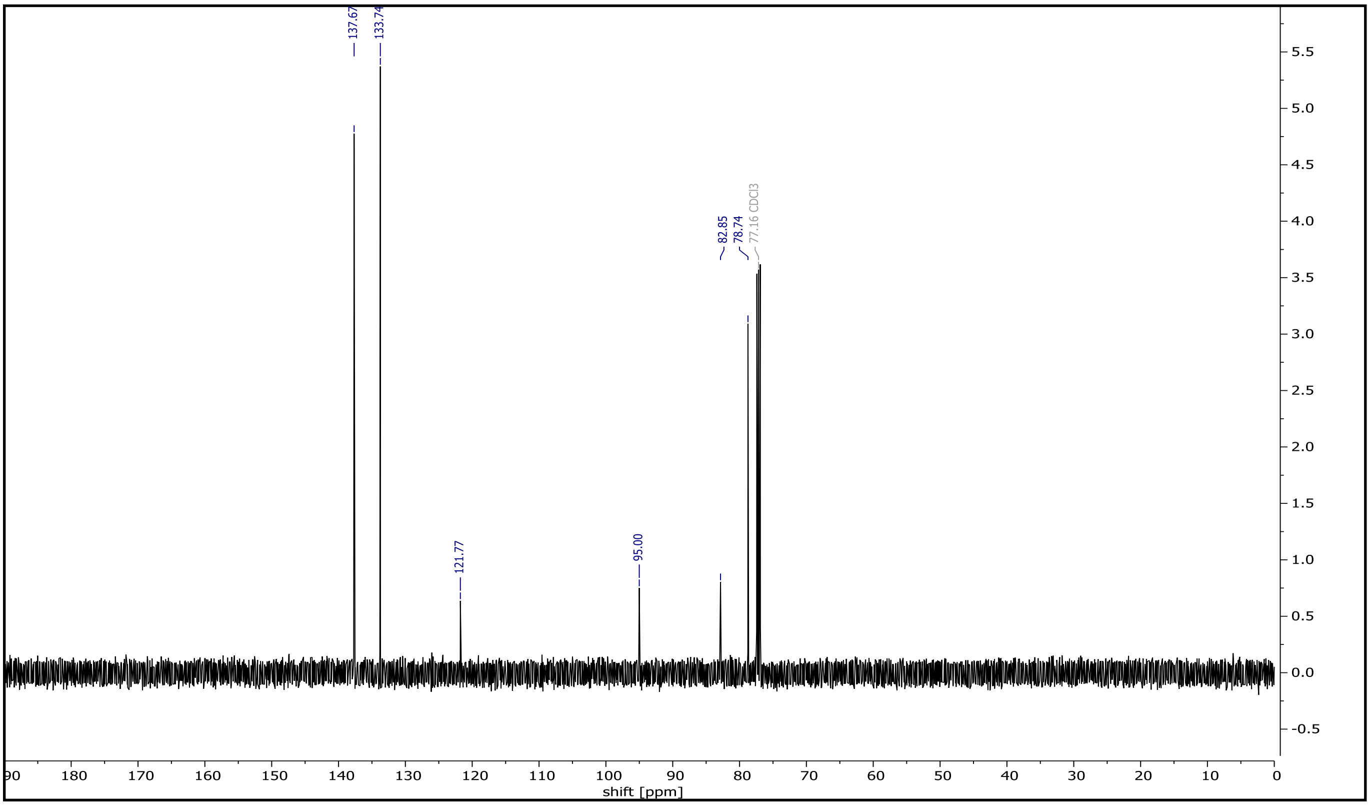

5-Ethynyl-1,2,3-trimethoxybenzene A-16

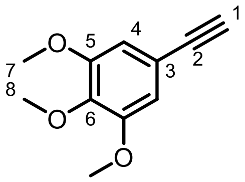

^{1}H-NMR (599 MHz,CDCl$_3$)

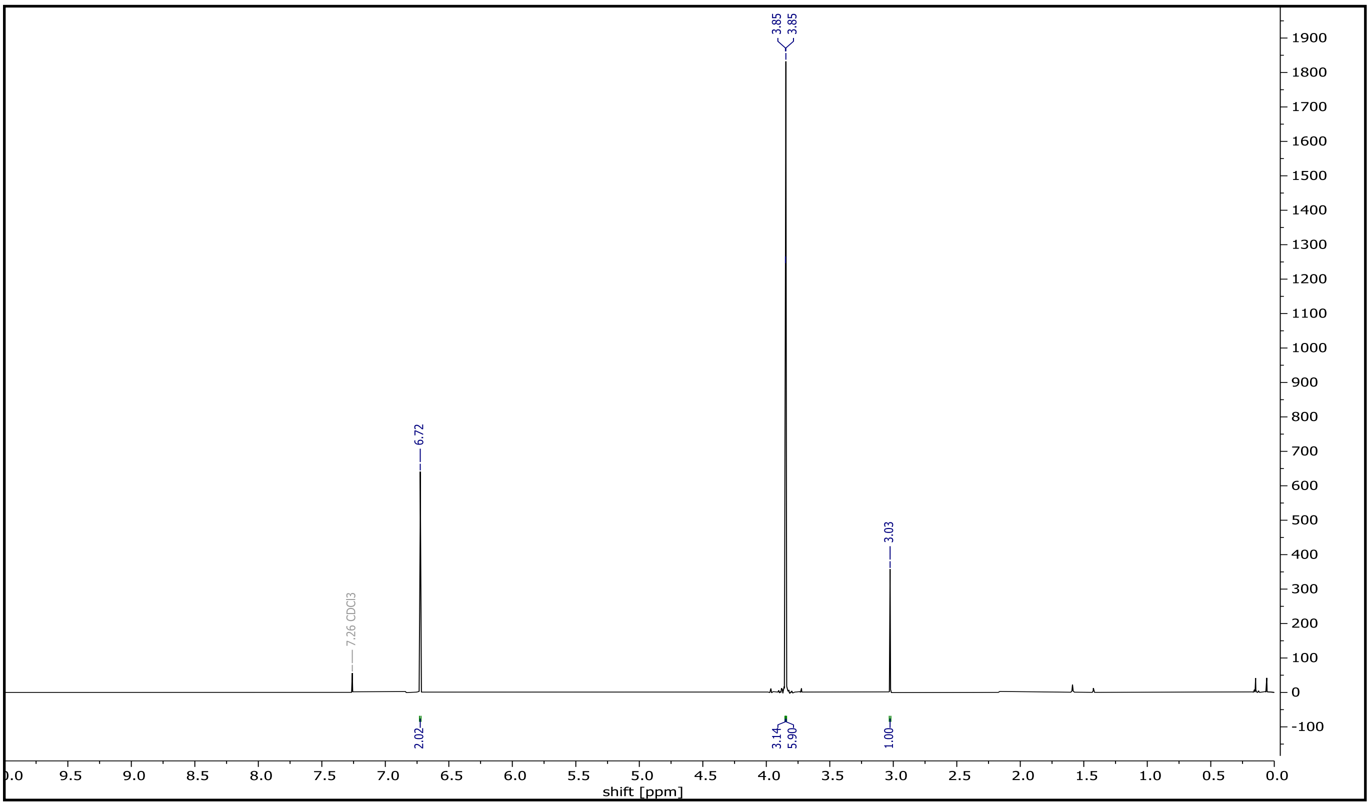

^{13}C-NMR (151 MHz,CDCl$_3$)

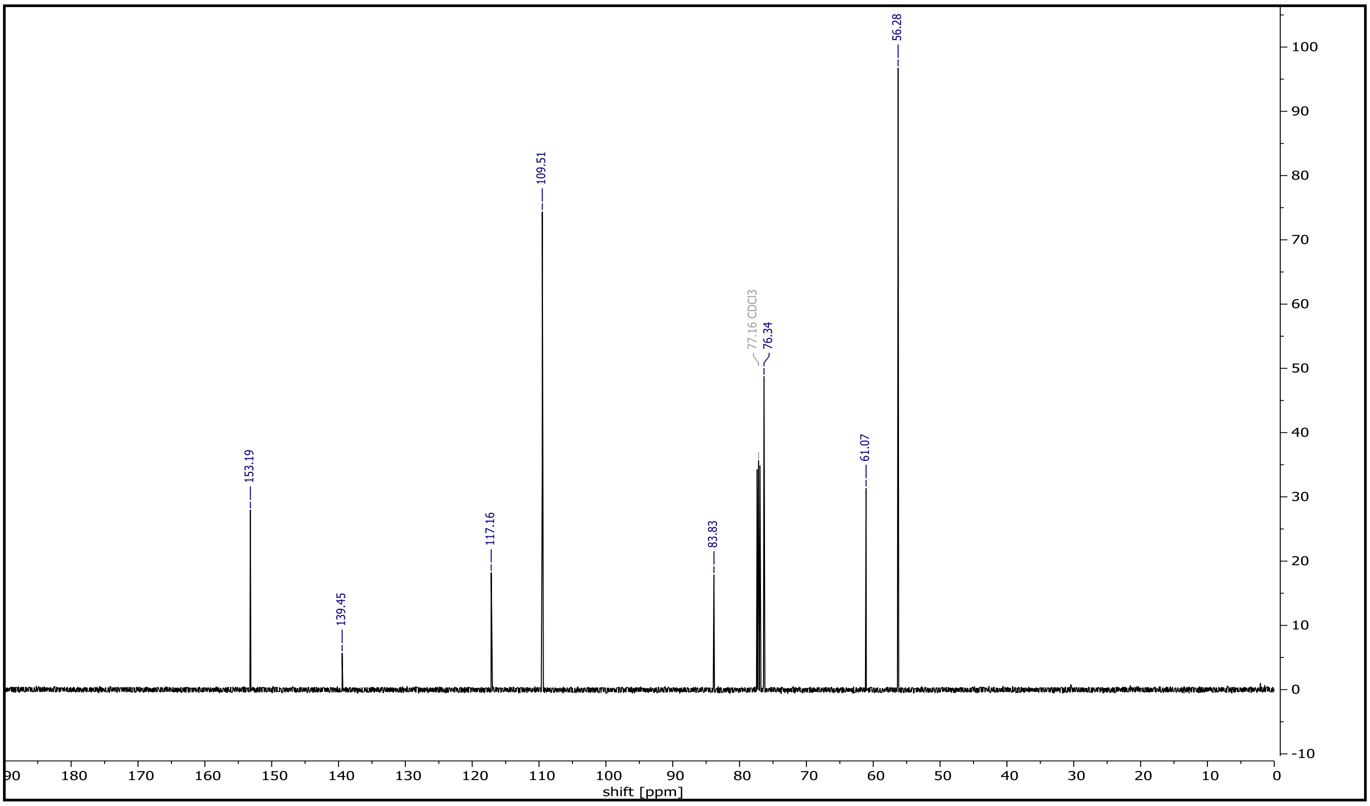

Piperonal S-17

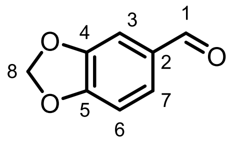

^{1}H-NMR (500 MHz,$CDCl_3$)

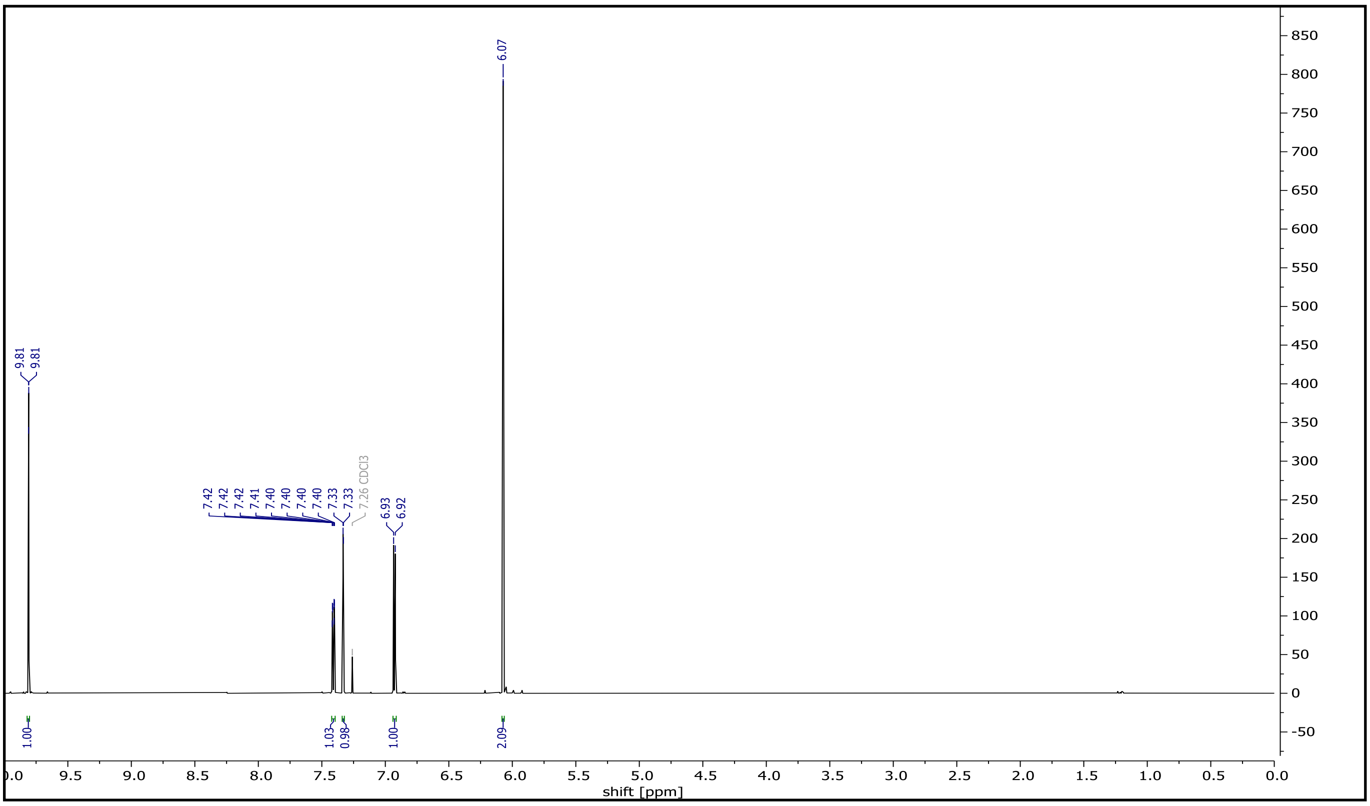

^{13}C-NMR (126 MHz,$CDCl_3$)

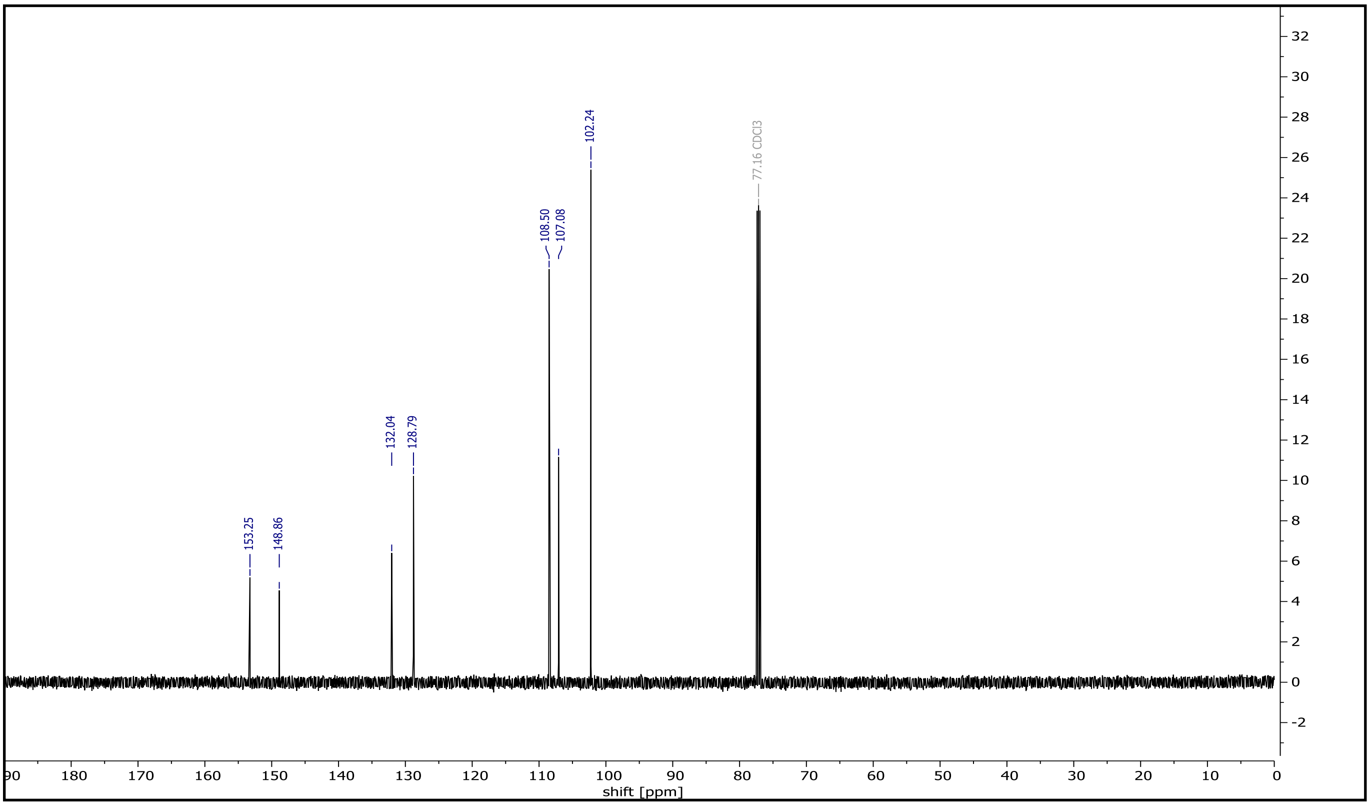

5-Ethynylbenzo[*d*][1,3]dioxole A-17

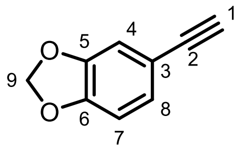

^{1}H-NMR (500 MHz,CDCl$_3$)

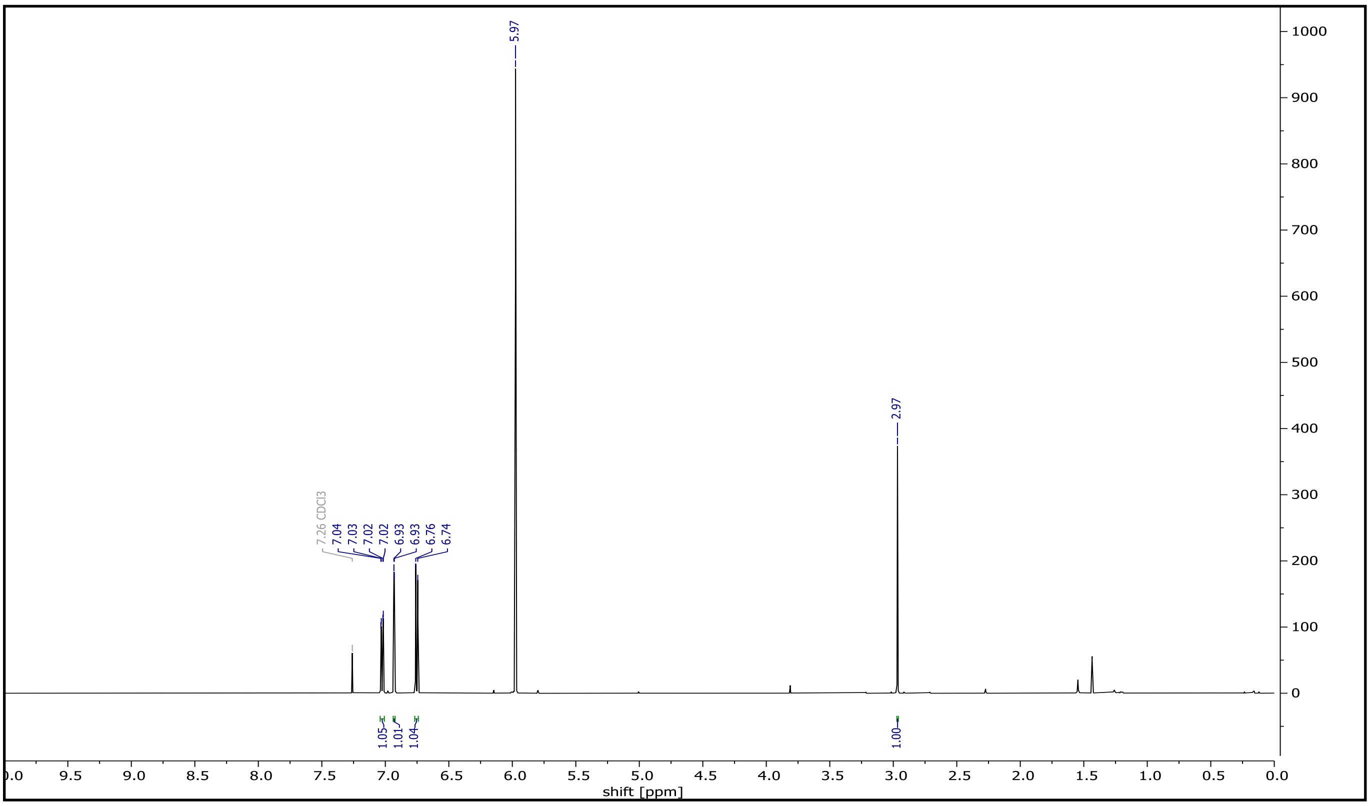

^{13}C-NMR (126 MHz,CDCl$_3$)

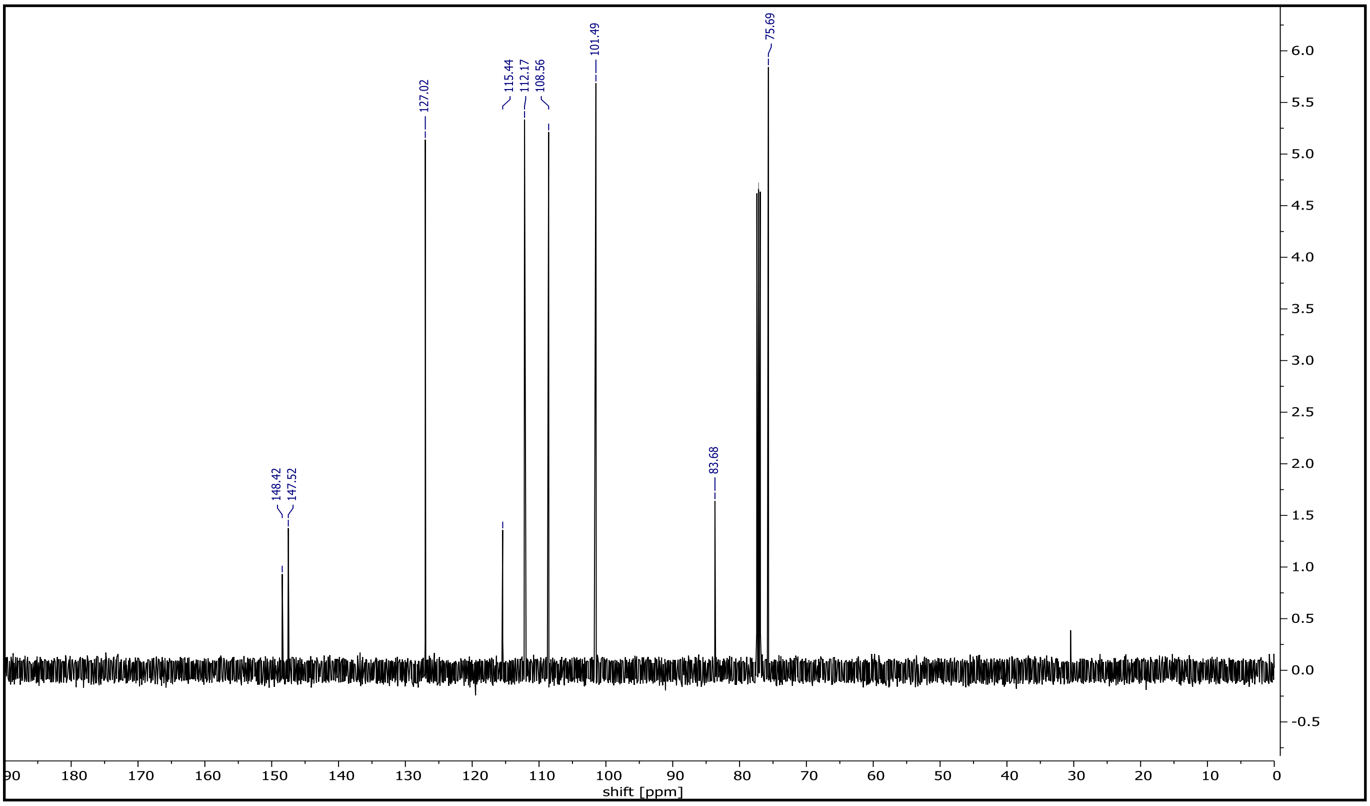

Supplement: SC-012-D1SC02454J-s001 [file SC-012-D1SC02454J-s001.pdf]
